# Evidence Map and Systematic Review of Disinfection Efficacy on Environmental Surfaces in Healthcare Facilities

**DOI:** 10.3390/ijerph182111100

**Published:** 2021-10-22

**Authors:** Elizabeth C. Christenson, Ryan Cronk, Helen Atkinson, Aayush Bhatt, Emilio Berdiel, Michelle Cawley, Grace Cho, Collin Knox Coleman, Cailee Harrington, Kylie Heilferty, Don Fejfar, Emily J. Grant, Karen Grigg, Tanmay Joshi, Suniti Mohan, Grace Pelak, Yuhong Shu, Jamie Bartram

**Affiliations:** 1 The Water Institute, Gillings School of Global Public Health, University of North Carolina, Chapel Hill, NC 27599, USA; eliz@alumni.unc.edu (E.C.C.); rcronk@alumni.unc.edu (R.C.); hpamea@live.unc.edu (H.A.); aayush@live.unc.edu (A.B.); eberdiel@live.unc.edu (E.B.); seohee@live.unc.edu (G.C.); cwrobert@live.unc.edu (C.K.C.); caileeh@live.unc.edu (C.H.); kylblair@live.unc.edu (K.H.); donlukef@live.unc.edu (D.F.); ejgrant@live.unc.edu (E.J.G.); tanmayj3@live.unc.edu (T.J.); suniti@live.unc.edu (S.M.); yuhshu@live.unc.edu (Y.S.); 2 ICF, Durham, NC 27713, USA; 3 Health Sciences Library, University of North Carolina, Chapel Hill, NC 27599, USA; mcawley@unc.edu (M.C.); kgrigg@email.unc.edu (K.G.); pelakg@email.unc.edu (G.P.); 4 School of Civil Engineering, University of Leeds, Leeds LS2 9DY, UK

**Keywords:** disinfection, healthcare facilities, healthcare-associated infections, environmental surfaces, infection prevention and control

## Abstract

Healthcare-associated infections (HAIs) contribute to patient morbidity and mortality with an estimated 1.7 million infections and 99,000 deaths costing USD $28–34 billion annually in the United States alone. There is little understanding as to if current environmental surface disinfection practices reduce pathogen load, and subsequently HAIs, in critical care settings. This evidence map includes a systematic review on the efficacy of disinfecting environmental surfaces in healthcare facilities. We screened 17,064 abstracts, 635 full texts, and included 181 articles for data extraction and study quality assessment. We reviewed ten disinfectant types and compared disinfectants with respect to study design, outcome organism, and fourteen indictors of study quality. We found important areas for improvement and gaps in the research related to study design, implementation, and analysis. Implementation of disinfection, a determinant of disinfection outcomes, was not measured in most studies and few studies assessed fungi or viruses. Assessing and comparing disinfection efficacy was impeded by study heterogeneity; however, we catalogued the outcomes and results for each disinfection type. We concluded that guidelines for disinfectant use are primarily based on laboratory data rather than a systematic review of in situ disinfection efficacy. It is critically important for practitioners and researchers to consider system-level efficacy and not just the efficacy of the disinfectant.

## 1. Introduction

Healthcare-associated infections (HAIs) contribute to patient morbidity and mortality with an estimated 687,000 infections and 72,000 deaths in the United States in 2015 [1] and an additional 2.6 million annual infections in the European Union [2]. The burden of HAIs is higher in low- and middle-income countries [3,4,5]. HAIs are often correlated with the presence of contaminated environmental surfaces and are exacerbated by multi-drug resistance and compounded by spore-producing or biofilm-associated pathogens that are difficult to disinfect [6]. Healthcare-associated pathogens with high morbidity and mortality, including vancomycin-resistant *Enterococci* (VRE), methicillin-resistant *Staphylococcus aureus* (MRSA), *Clostridium difficile*, and *Candida auris*, are especially problematic in the intensive care unit (ICU), where patients are often immunocompromised [7,8].

The environmental transmission pathways of pathogens and HAIs are varied. They include medical devices, air ventilation units, environmental surfaces (e.g., floors, bedrails), water, healthcare workers (e.g., hands), and mobile elements (e.g., wheelchairs, shoes, etc.); floors may play a large role [9,10,11,12]. Meta-analyses support the environment as being a transmission pathway through roommates/prior occupants with HAIs in high-income settings [13,14]. Patients hospitalized in rooms previously occupied by people infected with HAIs are at increased odds of HAI acquisition compared to patients whose prior room occupant was negative for HAIs [15,16,17].

Interventions to reduce the environment as a transmission pathway for HAIs are also varied. Improved cleaning procedures [18,19], training environmental service personnel [20,21,22], hand hygiene [10,11,12,23], and bundled disinfection interventions reduce the concentrations of pathogens on environmental surfaces and reduce HAIs in healthcare facilities [19,24]. However, transmission pathways are poorly disaggregated. For bundled interventions, it is challenging to determine each component’s independent effect and the contribution of potential transmission pathways on HAI acquisition. The literature has focused on multimodal strategies in infection prevention and control (IPC) without analyzing the impact of separate components, such as disinfection implementation or disinfection efficacy [25]. Understanding the efficacy of the individual components of multi-modal strategies may help guide bundle development and may aid in decision-making in low-resource settings.

One systematic review found that most studies that included bundled interventions with an environmental cleaning and disinfection component were more effective than bundled interventions without the component at reducing HAIs [26]. Nevertheless, the extent to which surface disinfection contributes to HAI reductions is unclear.

The hierarchy of studies for assessing the impact of infection control is outcomes from (1) in vitro reduction of reference pathogens → (2) in situ reduction of environmental pathogens → (3) colonization and pathogen transmission to patients → (4) patient HAIs [27,28]. In vitro studies, such as quantitative carrier tests, are appropriate for determining the disinfectant concentration and contact time necessary to provide a log reduction target of pathogens on surfaces [29,30]. Large bodies of in vitro surface disinfection research exist for agriculture, food production and preparation, and biodefense but are not always applicable to pathogens that are regularly associated with HAIs. In vitro studies on surface disinfection provide the necessary disinfection kinetics to justify in situ studies yet lack the variance in surfaces, environmentally derived pathogens, and inadequate terminal cleaning methods. There are reported reductions in disinfection efficacy in the healthcare facility setting in situ when compared to reported in vitro efficacy (see, e.g., [31]). Additionally, pathogens remain viable on porous and non-porous surfaces for extended times in ambient conditions [32,33,34,35].

There is still little understanding as to if current disinfection practices on environmental surfaces reduce pathogen load and subsequently HAIs in critical care settings. There has not been a rigorous systematic review of the efficacy of disinfection interventions in situ. While a prior systematic review [28] and related technical brief [36] identified the disinfection methods used in healthcare facilities on environmental surfaces, the work was restricted to publications in English and to efficacy on specific Gram-positive pathogens (MRSA, VRE, *C. difficile*). The literature primarily concerns multimodal strategies in infection prevention and control (IPC) without analyzing the impact of separate components [25]. This is exemplified in a systematic review assessing the effect of multi-modal interventions on HAIs, which reported that 35%–55% of HAIs are preventable but did not differentiate the multi-faceted components of the interventions [37]. In situ evidence for the efficacy of disinfection interventions are based on non-systematic methods such as narrative review [38], literature reviews [19], commentary [39], and clinical guidance [40]. Furthermore, clinical practice guidance for environmental surface cleaning is disparate between evidence-based or consensus-driven and narrative-based (i.e., logically justified) recommendations. Guidelines vary based on country of origin with government, independent associations, and professional societies issuing 69 separate guidance documents [28]. 

We conducted a systematic review to develop an evidence map that (1) catalogues in situ disinfection interventions on environmental surfaces (excepting UV); (2) identifies gaps in the research and areas for improvement; (3) catalogues the in situ efficacy of environmental surface disinfection interventions in healthcare facilities on all HAI and organism outcomes; and (4) summarizes important components of IPC strategies for the disinfection of environmental surfaces in a proposed framework for ideal disinfection.

## 2. Materials and Methods

Search Strategy and Machine Learning: We searched PubMed, Embase, Scopus, and Web of Science in January 2020 for studies related to healthcare facilities and disinfectants (as described in Supplementary Material 1). Healthcare facility terms included inpatient and outpatient environments and spanned global healthcare facilities in a variety of critical care environments. Disinfection terms included specific chemical disinfectants identified by the Centers for Disease Control and Prevention (CDC) [41] and the World Health Organization (WHO) [42] for use in health care disinfection, such as alcohols, chlorine and demand-release chlorine compounds, formaldehyde, glutaraldehyde, hydrogen peroxide, iodophors, ortho-phthalaldehyde, peracetic acid, phenolics, and quaternary ammonium compounds as well as non-touch interventions such as vapors and antimicrobial surfaces. Disinfection terms also included generic terms such as “decontaminant” and “disinfectant” to identify studies for which we did not specify the disinfectant in the search terms. We excluded reviews and other article types such as commentaries, as specified in Supporting Information Supplementary Material 1. After the duplicates were removed, we used machine learning to prioritize studies to be screened manually for relevance using Document Classification and Topic Extraction Resource (DoCTER) software (ICF, Fairfax, VA, USA). All of the studies that were predicted to be relevant by DoCTER were imported to Covidence reference management software (Veritas Health Innovation, Melbourne, Australia) for title and abstract screening.

We used supervised clustering with an ensemble approach to prioritize studies for manual screening using the text of titles and abstracts (similar to the approach described in [43]). Supervised clustering is a form of semi-supervised learning that uses known relevant studies (i.e., seeds) to identify unclassified studies that are likely to be relevant. Seed studies are a form of training data but require fewer positive studies than typically necessary for machine learning algorithms.

**Figure 1:**
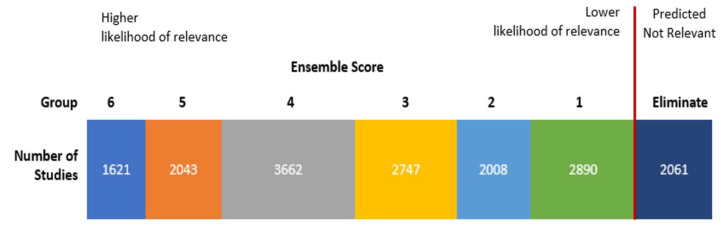
Results of ensemble supervised clustering using 32 seed studies. Studies with an ensemble score of 1 or more were screened manually for relevance.

To identify seeds, we screened 750 randomly selected studies from which 32 qualifying studies served as seeds for supervised clustering. One person reviewed studies for use as seeds, and these studies were confirmed by a subject matter expert. The ensemble approach uses two algorithms: k-means and non-negative matrix factorization, and three cluster sizes: 10, 20, and 30. Using each algorithm with the three different cluster numbers yields six different clustering models (e.g., KM-10 model is the k-means algorithm with 10 clusters, and KM-20 is the k-means algorithm with 20 clusters). The six models were applied to the title and abstract text. The output of supervised clustering with a six-model ensemble approach had an ensemble score ranging from 0 to 6 for each study based on the number of models where the study was found in a relevant cluster (i.e., a cluster with a high proportion of seed studies). We ran supervised clustering with the 32 seed studies, and all non-seed studies were given an ensemble score (Figure 1). We specified at least 90 percent recall of relevant studies from the unclassified corpus in DoCTER but a recall closer to 100 percent was anticipated because all 32 seeds were captured by one or more clusters. Overall, we expected approximately 95 percent recall by reviewing all of the studies with an ensemble score of 1 or higher.

Inclusion Criteria: Titles and abstracts of all of the studies with an ensemble score of 1 or higher for relevance, which included the 32 seed studies, were screened. After the titles and abstracts were screened, the full text was read to determine if the study would be included. Two reviewers independently screened all of the titles and abstracts, and disputes were resolved through discussion. One reviewer independently screened the full texts for inclusion. The 2061 studies not found to be in a relevant cluster by any model (score of 0) were removed from analysis without manual screening (Figure 1).

Inclusion criteria for title and abstract and full text screening were (1) disinfection interventions that did not include UV or other light-based interventions to reduce the scope of the systematic review and excluded any study that had a disinfection component that was part of a bundled or multi-modal intervention package (e.g., a training intervention was implemented simultaneously to disinfection intervention). Studies were excluded if the disinfectant was not specified and if the study was cross-sectional in nature (e.g., no comparator). (2) We excluded articles that did not sample environmental surfaces, which were defined as non-porous surfaces that are either part of the built environment (e.g., walls, toilet) of a healthcare facility or remain in the critical care environment during the patient’s stay (e.g., bedside table), and did not include studies that focused solely on mobile elements such as doctors’ hands, wheelchairs, or medical instruments (e.g., stethoscopes, endoscopes). We excluded equipment surfaces, including studies that focused solely on central-line and dialysis. We excluded studies that focused on sink traps, the inside of showerheads, and porous surfaces (e.g., curtains, linens). If studies included surfaces in addition to environmental surfaces in the sampling protocol, we included the study. (3) The critical care environment included all healthcare facilities except veterinary, long-term residential care, and dental facilities. We excluded areas in healthcare facilities that patients would not visit, such as laboratory, laundry, and preparatory areas. We excluded long-term care facilities because IPC management and implementation may be different than other healthcare facilities. (4) Only original, peer-reviewed research was included. Systematic reviews, meta-analyses, poster abstracts, and any conference proceedings were not included. (5) Outcome measurements had to target organisms from surfaces, rather than from, e.g., air. We included HAI outcomes.

Data Extraction and Risk of Bias: Multiple reviewers independently extracted data from studies meeting the inclusion criteria. All data were reviewed for quality control by one reviewer. Interventions were categorized as being manually applied, antimicrobial surfaces applications, or vapors. Disinfectants with multiple active ingredients were categorized based on the active ingredient with the highest percentage by volume. Antimicrobial surfaces were comprised of inherently antibacterial surfaces (e.g., copper) or were coated with a product that bonded with the surface to inhibit growth. Coatings that were re-applied more than once a week were considered manually applied products rather than surface interventions (e.g., [44]). Outcome organisms were grouped into Gram-positive cocci, Gram-positive bacilli, Gram-negative bacteria, fungi, viruses, and “all viable organisms” (non-specific culture media or outcomes that combined multiple organism types, e.g., multi-drug resistant organisms that combine Gram-negative and Gram-positive organisms). Outcome measurement quality was ranked in descending order from organism concentration followed by percent surfaces positive, followed by adenosine triphosphate (ATP) measurements or qualitative observations; they were then classified according to highest quality outcome. HAI and antibiotic resistance outcomes were also identified. Study design was categorized for studies with outcome organisms (i.e., excluding studies with only HAI outcomes) as crossover design, controlled design (controlled before-after or controlled cohort study design), or uncontrolled study design (studies without a contemporary control). All studies were classified according to the World Bank country income group [45] for study location.

Risk of bias was assessed for each study by two reviewers using a fourteen-point study quality assessment instrument adapted from the National Institutes of Health (NIH) Study Quality Assessment Tool [46]. The study quality instrument included fourteen indicators to assess bias across setting, methods, outcomes, and conclusions of the included studies with heterogeneous study design; for contemporary controls, baseline equivalence, bias due to deviation from protocol, blind evaluation, bias due to missing data, bias in selective reporting, conflicts of interest, and others were considered Supplementary Material 2, Table S5). Each indicator received a score of 0, 0.5, or 1, such that the maximum total score for each study was 14. Twenty-three percent of studies were randomly selected for secondary independent review. Cohen’s kappa statistics and raw percent agreement were calculated to compare inter-rater reliability for each of the indicators [47].

This review was not registered nor was the review protocol registered. This systematic review was based on the Preferred Reporting Items for Systematic Reviews and Meta-Analyses (PRISMA) 2020 Checklist [48] (see Supplementary Material 3).

## 3. Results

**Figure 2:**
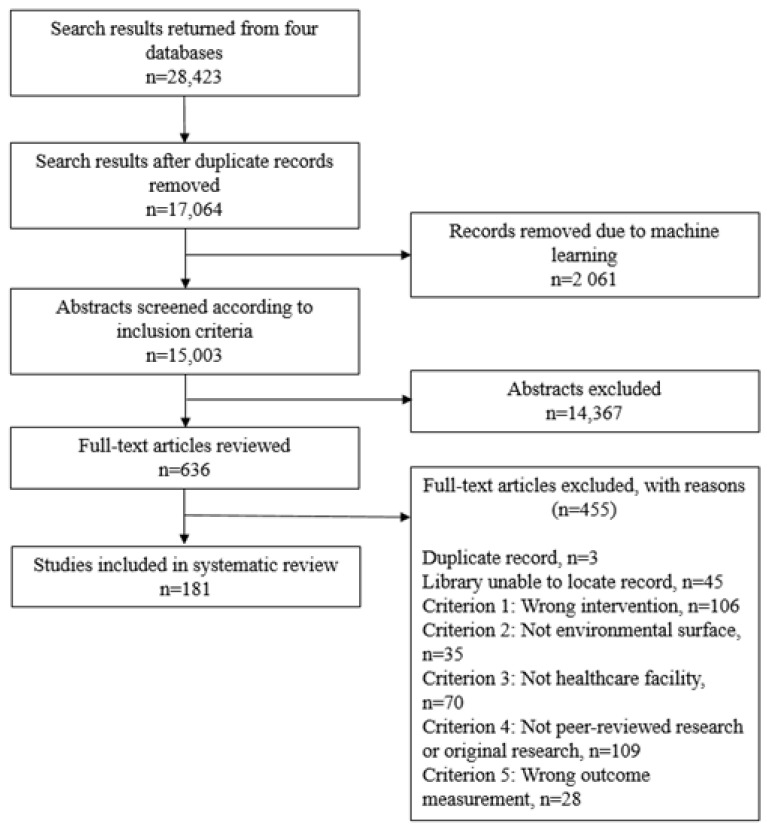
PRISMA flowchart of systematic review.

The initial literature search identified 17,064 studies, of which 2061 were eliminated through machine learning (Figure 2). Of the remaining 15,003 articles, 635 articles were selected for full text review, and were 181 included for data extraction. The included studies are listed in Supplementary Material 2, Table S6. Characteristics of the included studies with respect to disinfection intervention type, outcome HAI or organism assessed, outcome measurement, study design, and World Bank country income group for country of study location are listed in Table 1.

Manually applied interventions included alcohol, peroxygen, quaternary ammonium compounds (QACs), sodium hypochlorite, and other chlorine; surface interventions included copper and other non-copper surface applications or coatings; and vapor interventions included hydrogen peroxide interventions. We identified the target pathogens and/or HAIs measured due to each disinfection intervention and presented an evidence map and summary of the data relating to study design, organism outcome, and disinfection intervention.

Most studies (86%) were conducted in high income countries such as the USA, UK, Italy, and Japan. Studies from upper-middle income countries (10%) were conducted in Turkey, Brazil, South Africa, Russia, Mexico, Indonesia, China, and Bosnia and Herzegovina. Studies from lower-middle income countries (3%) comprised India, Sri Lanka, Pakistan, and Morocco. One study was conducted in a low-income country (Sierra Leone). 

### 3.1. Disinfection Type

Manually applied disinfectant application methods included mopping, wiping, pouring, or spraying, using, e.g., cotton, microfiber, or pre-moistened cloths, wipes, mops. Alcohol disinfection, including some disinfectants with multiple active ingredients (e.g., chlorhexidine gluconate, QAC), was identified in 11% of studies [49,50,51,52,53,54,55,56,57,58,59,60,61,62,63,64,65,66,67,68]. Peroxygen disinfection, including hydrogen peroxide, peracetic acid, or peroxymonosulfate, was identified in 9% of studies [50,56,57,69,70,71,72,73,74,75,76,77,78,79,80,81,82]. QAC disinfection, which included diverse active ingredients such as primarily didecyl dimethyl ammonium chloride and benzyl ammonium chloride, was identified in 25% of studies [20,31,44,49,52,59,63,64,69,73,75,79,80,83,84,85,86,87,88,89,90,91,92,93,94,95,96,97,98,99,100,101,102,103,104,105,106,107,108,109,110,111,112,113]. Sodium hypochlorite disinfection, which comprised any disinfection method specified as bleach or sodium hypochlorite, was identified in 19% of studies [20,44,60,61,75,89,95,97,100,104,109,114,115,116,117,118,119,120,121,122,123,124,125,126,127,128,129,130,131,132,133,134,135]. Other chlorine disinfectants were identified in 14% of studies [65,70,78,101,118,135,136,137,138,139,140,141,142,143,144,145,146,147,148,149,150,151,152,153,154]. Other chlorines included demand-release chlorines such as sodium dichloroisocyanurate, chloramine, chlorine-dioxide, and bromo-chloro-dimethyl-hydantoin, as well as electrolyzed water, hypochlorous acid, and any unspecified chlorine-based disinfectant. All other manually applied disinfectants, which included phenols, hydrochlorides, aldehydes, copper, glucopratamin, triethylene glycol, and grapefruit seed extract, were identified in 10% of studies [49,55,57,84,136,145,155,156,157,158,159,160,161,162,163,164,165,166].

For surface interventions, we found that copper surfaces were in 9% of studies [90,132,152,154,167,168,169,170,171,172,173,174,175,176,177,178,179]. Other non-copper surface applications or coatings comprised 8% of studies [87,102,180,181,182,183,184,185,186,187,188,189,190,191,192]. Other non-copper surfaces included coatings incorporating metals such as titanium oxide and silver ions as well as other coatings comprised of polymers, isopropyl alcohol and organofunctional silane, organosilane products, silicon nano-coating inorganic metal and organic quaternary ammonium, silicone quaternary amine, quaternary ammonium silyl oxide, and titanyl oxide moieties. One study used a probiotic-based cleaning product.

Vapor disinfection includes systems described as producing and dispersing vapors, aerosols, or droplets of disinfectants through spray, mist, or fogging machines. Hydrogen peroxide vapor was identified in 18% of studies [70,72,77,85,86,99,111,117,122,125,129,130,141,142,193,194,195,196,197,198,199,200,201,202,203,204,205,206,207,208,209,210,211]. Other vaporized disinfection methods, which included chlorine dioxide, sodium hypochlorite, essential oils, formalin, QACs, glutaral, beta propiolactone, steam, acidic electrolytic water, ozone, and steam, were identified in 10% of studies [70,84,137,203,212,213,214,215,216,217,218,219,220,221,222,223].

### 3.2. Outcomes

**Figure 3:**
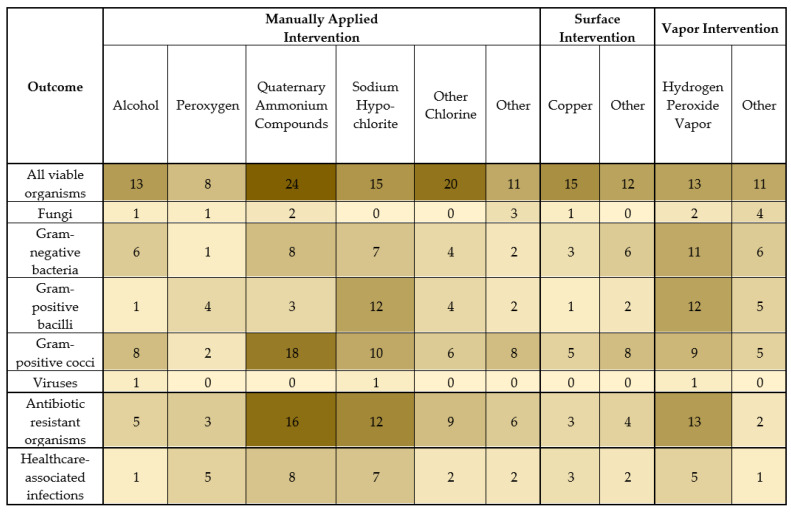
Number of studies with the indicated outcome organism or healthcare-associated infection and the indicated intervention type. A darker shade indicates more studies with the indicated outcome and intervention type.

Of the 181 studies included, 168 (93%) assessed organisms on environmental surfaces (Figure 3). Many studies described multiple outcomes and multiple intervention types. Of the included studies, the outcome organisms that were reported were usually all viable organisms (66%) or Gram-positive cocci (38%), followed by Gram-negative bacteria (25%), Gram-positive bacilli (20%), and fungi (7%). Three studies (2%) assessed the disinfectant efficacy on environmental surfaces for viruses in situ. Antibiotic-resistant organisms were assessed in 33% of the studies, most commonly MRSA, VRE, carbapenem-resistant *Acitenobacter baumannii*, extended-spectrum beta-lactamase (ESBL)-producing organisms, and other antibiotic-resistant Gram-negative organisms. 

Most studies assessing all viable bacteria measured concentration, though when assessing specific organisms, the outcome was more commonly percent surface positive. Overall, 63% of studies reported concentration outcomes, 43% reported percent surface positive, 6% reported ATP or qualitative outcomes, and 2% reported outcomes related to gene abundance.

Of the 181 studies included, 28 (15%) reported HAI outcomes due to an environmental surface disinfection intervention, and 11 of the 28 HAI studies assessed drug-resistant organisms.

### 3.3. Disinfection Efficacy

Efficacy was defined differently among the included studies and was reported by comparing reduction, prevalence ratio, mean, median, range, and/or qualitative assessment. The intervention was not always compared to a control or another intervention with respect to statistical significance nor with respect to measures of variance and confidence intervals. Outcome measurements included concentration, gene abundance, percent surfaces positive, and ATP bioluminescence (Table 1). Studies used different comparators, with some studies comparing a disinfectant to a control without disinfectant and others to another disinfectant. 

Efficacy for each of the ten disinfection interventions is presented by different outcome (Gram-positive organisms (bacilli and cocci), Gram-negative organisms, fungi, all viable organisms, and HAIs) in Supplementary Material 4. The study setting, intervention methods, and results for all studies organized by disinfection type, and outcome organisms are listed in Supplementary Material 5.

### 3.4. Proposed Framework for Ideal Disinfection

In this review we catalogued studies that assessed the in situ efficacy of disinfectants on environmental surfaces. However, the disinfectant efficacy on target organisms is not the only consideration for the effective disinfection of environmental surfaces. Building on the framework identifying properties for the ideal disinfectant [41], we propose an updated framework for ideal disinfection that includes all disinfection types and not only chemical disinfectants. The proposed decision-making framework for the ideal disinfectant includes nine criteria categorized under three themes: fit for purpose, safety, and implementation (Table 2).

The fit for purpose criteria allow the healthcare facility to identify disinfection needs based on, for example, critical care setting or pathogen. This systematic review rigorously catalogues evidence concerning the first question regarding disinfection efficacy. Other questions include the persistence or residual effect of the disinfectants that are more commonly studied among surface and vapor disinfectant interventions than among manually applied disinfectants (see, e.g., [63,64,102,103,132,166,180], the efficacy of the disinfectant when in the presence of increased biofilm or organic material (see, e.g., [49,56,110,145,197,201]), and whether pre-cleaning is needed (see, e.g., [123,141,193]).

Safety criteria ensure that the disinfectant does not have unintended side effects. We identified themes around disinfectants contributing to chemical or antimicrobial resistance (e.g., [44,62,69,110,158]) and toxicity or discomfort to healthcare workers and patients (see, e.g., [49,134,135,137,145,164,165,182,196,213,219,220,222]) as well as the compatibility of the disinfectant on surfaces and clothing (see, e.g., [61,69,83,85,139,145,172,189,220]). 

Many articles included themes around the implementation of disinfection interventions. Specific themes were related to the adherence to the protocol, the appropriate application of the disinfectant, and the costs. Adherence was discussed as being related to monitoring and training. Studies assessing disinfection implementation found that objective measurements of disinfection (e.g., ATP fluorescence or environmental samples rather than visual inspection) improved disinfection practices [28,153]. 

Monitoring for disinfection compliance was primarily conducted through biological indicators for HPV interventions [194,199,201,209] and by using fluorescent markers or random audits [62,69,114,135,153]. Implementation may be affected by the inappropriate application of the protocol related to disinfectant contact time or improper disinfectant concentration (see, e.g., [61,78,153]) or whether implementation improved or worsened due to the method of application (e.g., wipes vs. mop; cotton vs. microfiber; one cloth vs. two cloths; see, e.g., [62,92,104,107,153,165]). Some antimicrobial coatings may not bind appropriately to target surfaces, and this may decrease the apparent efficacy. Training environmental services staff before and during interventions were identified as important for both adherence to protocol and to the appropriate application of the disinfectant (see, e.g., [20,78,120,125]). Few studies mentioned costs although some reported monetary or time costs associated with a disinfectant type (see, e.g., [69,70,92,114,126,131,137,141,160,197,201,223]).

### 3.5. Study Quality

Studies primarily used a before-after design without a simultaneous control (48%) or controlled cohort/controlled before-after study designs (46%). Few studies had crossover designs (5%) (Table 1).

**Figure 4:**
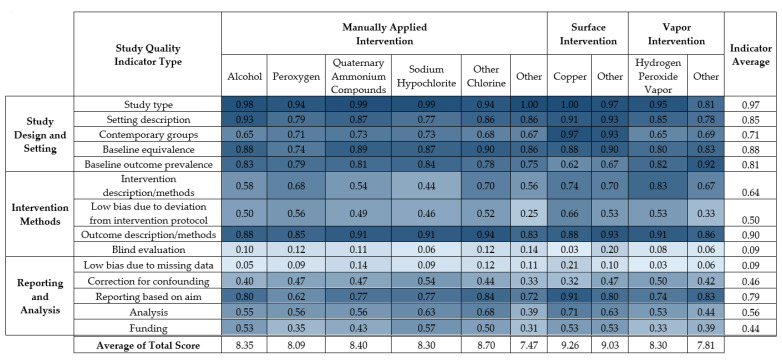
Average score for fourteen study quality indicators individually and in sum for each disinfection intervention type.

The average score for each of fourteen study quality indicators is displayed in Figure 4. Results of the 14-point study quality assessment for each study are listed in Supplementary Material 2, Table S7. A summary table of the proportions of studies that received each study quality criterion for each study quality indicator appears in Supplementary Material 2, Table S5.

The strengths of the disinfection interventions were primarily indicated in study description and study design. The majority (93%) of the studies had natural study designs, with 6% having seeded study designs. Most (73%) studies described the healthcare setting and environmental surfaces, 77% had clearly defined and equivalent healthcare settings for the control and intervention groups, 76% measured the initial burden before disinfection intervention, 85% had well-defined outcome methods, and 62% reported results based on the aim of the study. 

Frequent weaknesses in study quality concerned implementation, reporting, and analysis. Most (90%) studies did not report whether there were missing data in the analyses, and 85% did not report blind evaluation of both healthcare workers and microbiologists. Only 13% reported blind evaluation in either group. Half (52%) of the studies did not sufficiently identify the disinfectant (e.g., product active ingredients and concentration), 67% of studies did not report measures of variance nor conduct a statistical test, 38% of studies measured the implementation of the disinfection intervention through, e.g., ATP assays, 23% indicated that the staff were trained but that intervention was not monitored, and 38% did not discuss monitoring or training during the intervention. Finally, 45% of the studies had funding other than academic or government sources and did not include a statement of influence or conflicts of interest regarding funding contributions to study design, implementation, decision to publish, etc. 

The validation of the study quality instrument revealed a Cohen’s kappa coefficient of 0.75 (95% confidence interval 0.70–0.80) for agreeability between scoring by initial reviewers compared to scoring by the second independent reviewer (i.e., 70–80% of the scores can be attributed to reliable scoring by instrument users, and 20–30% can be attributed to random chance, error, or other factors). The raw percent agreement was calculated since the reviewers were trained, and low randomness due to guessing was expected. The raw percent agreement was 84%. The Cohen’s kappa suggests moderate inter-rater reliability, and the raw percent agreement suggests strong inter-rater reliability for scoring [47]. We interpreted the variability among indicator score variability as the degree to which the indicator could be easily interpreted for the study. Cohen’s kappa and raw percent agreement for each study quality indicator are in Supplementary Material 2, Table S8. 

## 4. Discussion

In this evidence map and systematic review, we identified 181 studies that described disinfection interventions on environmental surfaces across ten types of disinfection groups. We compared disinfectant interventions with respect to study design, outcome organism, and study quality; however, comparing disinfectant efficacy was difficult due to the heterogeneity in the study design and the unmeasured variability in disinfection implementation.

### 4.1. Strengths and Weaknesses

This systematic review identified important gaps in study design and study reporting for studies describing the efficacy of disinfection on environmental surfaces. Studies from low- and lower-middle income countries comprised only 4% of the included studies. Study design flaws affecting many studies included the omission of contemporary controls and only used a historical control. For the studies that did use a contemporary control (e.g., cohort study or controlled before-after), many did not report the initial concentration when comparing reductions or disinfection efficacy across two experimental groups. Among studies reporting initial concentration, few assessed and corrected for different initial concentrations between groups (see, e.g., [126]). Confounders identified in the studies included the differential use of cleaning or disinfection by the experimental group (e.g., researchers vs. healthcare services; trained nurses vs. outsourced cleaning team), differential implementation of disinfection strategy (no monitoring of implementation), differential or unclear sample collection time relative to routine or standard cleaning/disinfection, and no baseline equivalence of the outcome (initial burden not measured on control compared to intervention surfaces). The lack of monitoring and the audit of environmental services and disinfection implementation is a determinant that was not measured in most studies and has been identified in other systematic reviews of IPC as an important determinant for effective disinfection [19,28]. The best study designs compared the concentration of the outcome organism before and after disinfection intervention and before and after a contemporary control in equivalent healthcare settings. 

### 4.2. Disinfection Efficacy

Many studies inadequately described the disinfection intervention (active ingredient, contact times, and final dilutions for disinfectants used in intervention studies). The method of application is important. Contact time may be affected by different methods of implementation (e.g., wet mopping vs. spray mopping; cotton vs. microfiber cloths) (see, e.g., [62,92,107,153,165]). 

The outcomes that were measured were primarily on all viable organisms, specifically bacteria; only three studies assessed viruses, and eleven assessed fungi. Many studies did not assess concentration but rather the prevalence of surfaces that were positive for an organism. For pathogens of concern, most studies reported prevalence rather than concentration, and as a result, many may not have observed reductions, which is probably due to the low initial burden of the pathogen. More studies that reported all of the viable bacteria outcomes found significant effects compared to studies that reported other outcome organisms, which is possibly due to fewer studies assessing concentration among specific pathogens (see, e.g., [182]). Large sample sizes are necessary to assess significant reductions of low-prevalence pathogens; alternatively, studies that inoculate high concentrations of pathogens may elicit a better understanding of disinfectant efficacy.

### 4.3. Healthcare-Associated Infections

The identified studies have provided extensive evidence that environmental surfaces can be colonized with HAI-related pathogens after disinfection and that these surfaces could be an important transmission pathway, with some pathogens surviving prescribed disinfection. HAIs caused by antimicrobial-resistant organisms were assessed less often. It is estimated that 426,277 healthcare-associated infections are caused by antimicrobial-resistant microorganisms every year in the European Union [224]. Antimicrobial-resistant organisms present a challenge for treatment and can lead to increased morbidity and mortality, as they have a higher burden in low and middle income countries due to delayed presentation, low access to microbiological diagnostics and testing, and the low availability of second-line antibiotics [225]. Disinfection interventions on environmental surfaces may reduce HAIs; however, disinfection efficacy is only one component in a larger system of IPC strategies that are applicable to environmental surfaces. 

## 5. Conclusions

Comparing disinfection efficacy was impeded by study heterogeneity and study quality. As such, we conclude that guidelines for disinfectant use are primarily based on laboratory data rather than on a systematic review of in situ disinfection efficacy. We built upon the framework of the criteria for the selection of the ideal disinfectant to review important components for system-level disinfection efficacy as part of infection prevention and control (IPC) strategies.

In addition to disinfection efficacy, bundled interventions, including monitoring and implementation interventions such as measuring environmental bioburden, audit and feedback, training/re-education of environmental services staff, the addition of more cleaning staff or supervisors, and/or the use of implementation or quality checklists can improve IPC efficacy [226]. Monitoring/audit and feedback programs can prevent and control HAIs and antimicrobial resistance by supporting behavior changes during IPC implementation to create a monitoring and learning culture (as recommended in WHO 2018 [226]). Evidence deemed as being high-quality is reported to indicate that surveillance with active feedback may reduce HAIs [25]. A separate systematic review found intermediate-level evidence that standardizing audits and feedback reduces HAIs [227]. Studies reporting the sustainability of implementation interventions highlight the importance of ongoing education, direct feedback, and fiscal commitment to the monitoring/audit and feedback program from administrators [28]. 

Contextual factors for successful disinfection implementation include placing environmental services within the administrative hierarchy of the hospital, the outsourcing of environmental services, and a positive patient safety culture between clinical and environmental services staff and between supervisors and front-line personnel [28]. Multimodal strategies, including team-based, task-oriented, positive, and hands-on training, were considered to be more effective than formal training for IPC program adherence [227]. While implementation research has found that training, monitoring, and feedback of IPC implementation increases adherence to IPC programs, evidence about the long-term efficacy of IPC interventions is still needed [228]. As such, a complex of factors determines IPC effectiveness. While the choice of disinfectant and its efficacy have been dominant considerations in research and IPC programs, it is critically important for practitioners and researchers to consider system-level efficacy in reducing organism load and reducing HAIs in healthcare settings.

## Figures and Tables

**Table 1:** Study characteristics.

Characteristic		*n* (%)
**Number of Studies**	Total number of studies included	181 (100%)
	Studies with organism outcomes	168 (93%)
	Studies with HAI outcomes	28 (15%)
**Country Income**	High income	156 (86%)
	Upper-middle income	18 (10%)
	Lower-middle income	6 (3%)
	Low income	1 (1%)
**Study Design ^2^**	Controlled crossover	9 (5%)
	Other controlled study	78 (46%)
	Uncontrolled (no contemporary control)	81 (48%)
**Outcome Measurement ^1,2^**	Concentration	106 (63%)
	Percent surfaces	72 (43%)
	ATP or qualitative	10 (6%)
	Genes	4 (2%)
**Intervention ^1^**		
Manually Applied	Alcohol	20 (11%)
	Peroxygen	17 (9%)
	Quaternary ammonium compounds	45 (25%)
	Sodium hypochlorite	34 (19%)
	Other chlorine	25 (14%)
	Other manually applied	18 (10%)
Surface	Copper	17 (9%)
	Other surfaces	15 (8%)
Vapor	Hydrogen peroxide vapor	33 (18%)
	Other vapors	18 (10%)
**Outcome Organism ^1,2^**	All viable organisms	111 (66%)
	Gram-positive bacilli	34 (20%)
	Gram-positive cocci/other	63 (38%)
	Gram-negative bacteria	42 (25%)
	Fungi	11 (7%)
	Virus	3 (2%)
	Antibiotic-resistant organism	56 (33%)

^1^ % >100 because multiple organisms, outcomes, and/or interventions can be reported within one study. ^2^ Percentages of studies with outcome organisms only (total *n* = 168).

**Table 2:** Proposed framework for ideal disinfection as part of a larger infection prevention and control strategy.

**Fit for Purpose**
1. Veracity of disinfectant kill claim on target organism.
2. Dry surface persistence and longevity of disinfectant.
3. Efficacy of disinfectant with biofilm/organic material.
**Safety**
4. Chemical or antimicrobial resistance resulting from disinfectant.
5. Toxicity to healthcare workers or patients resulting from disinfectant.
6. Surface degradation resulting from disinfectant.
**Implementation**
7. Adherence to disinfection protocol.
8. Appropriate disinfection application.
9. Costs of disinfectant installation, application, and/or repair.

## Data Availability

All supporting data are in the Supplementary Materials.

## Supplementary Materials

The following are available online at https://www.mdpi.com/article/10.3390/ijerph182111100/s1, Supplementary Material 1: Database Search Strategies; Supplementary Material 2: List of Included Studies and Study Quality Assessment; Supplementary Material 3: PRISMA Checklists; Supplementary Material 4: Summary of Disinfection Intervention Efficacy; Supplementary Material 5: Tables for Disinfection Efficacy by Disinfection Intervention and Outcome
