# Supplementary material for: Evidence Map and Systematic Review of Disinfection Efficacy on Environmental Surfaces in Healthcare Facilities
Source: Int J Environ Res Public Health. 2021 Oct 22;18(21):11100. doi: 10.3390/ijerph182111100 (PMC8582915; doi:10.3390/ijerph182111100)
Supplement: Supplementary file 1 [file ijerph-18-11100-s001.zip › ijerph-1420603-supplementary.pdf]

Supplementary Materials
for
Evidence map and systematic review of disinfection efficacy on environmental surfaces in healthcare facilities

Authors: Elizabeth Christenson[1], Ryan Cronk[1], Helen Atkinson[1], Aayush Bhatt[1], Emilio Berdiel[1], Michelle Cawley[2], Grace Cho[1], Collin Knox Coleman[1], Cailee Harrington[1], Kylie Heilferty[1], Don Fejfar[1], Emily Grant[1], Karen Grigg[2], Tanmay Joshi[1], Suniti Mohan[1], Grace Pelak[2], Yuhong Shu[1], Jamie Bartram[1,3*]

[1]The Water Institute, Gillings School of Global Public Health, University of North Carolina at Chapel Hill
[2]Health Sciences Library, University of North Carolina at Chapel Hill
[3]University of Leeds

*Corresponding author – Jamie Bartram

Table of Contents
Supplementary Material 1 – Database Search Strategies
Supplementary Material 2 – List of Included Studies and Study Quality Assessment
Supplementary Material 3 – PRISMA Checklists
Supplementary Material 4 – Summary of Disinfection Intervention Efficacy
Supplementary Material 5 – Tables for Disinfection Efficacy by Disinfection Intervention and Outcome
References

Abbreviations

ATP – adenosine triphosphate
BCDMH - bromochlorodimethylhydantoin
CFU – colony forming units
CRAB – carbapenem-resistant *Acinetobacter baumannii*
CRE – carbapenem- resistant *Enterobacteriaceae*
ESBL – extended spectrum beta-lactamase
HAI – healthcare-associated infection
MDR – multi-drug resistant
MDRO – multi-drug resistant organism
MRSA – methicillin-resistant *Staphylococcus aureus*
NaDCC – sodium dichloroisocyanurate
QAC – quaternary ammonium compound
RLU – relative light unit
UK – the United Kingdom
USA – the United States of America
UV - ultraviolet
VRE – vancomycin-resistant *Enterococcus*

Supplementary Material 1: Database Search Strategies

Table of Contents:
Table S1: PubMed search terms
Table S2: Web of Science search terms
Table S3: Scopus search terms
Table S4: Embase search terms

Description of Inclusion Criteria

Healthcare facility terms included inpatient and outpatient environments and spanned global healthcare facilities in a variety of critical care environments. Disinfection terms included chemical disinfectants identified by the Centers for Disease Control and Prevention (CDC) (Rutala and Weber 2008) and World Health Organization (WHO) (World Health Organization 2002) for use in health care disinfection such as alcohols, chlorine and demand-release chlorine compounds, formaldehyde, glutaraldehyde, hydrogen peroxide, iodophors, ortho-phthalaldehyde, peracetic acid, phenolics, and quaternary ammonium compounds, as well as non-touch interventions such as vapors and antimicrobial surfaces. We excluded reviews and other article types such as commentaries.

Disinfection interventions did not include UV or other light-based interventions to reduce scope of systematic review and excluded any study that had a disinfection component that was part of a bundled or multi-modal intervention package (e.g. a training intervention was implemented simultaneous to disinfection intervention). Studies were excluded if the disinfectant was not specified and if the study was cross-sectional in nature (e.g. no comparator).

We excluded articles that did not sample environmental surfaces, defined as non-porous surfaces that are either part of the built environment (e.g. walls, toilet) of a healthcare facility or remain in the critical care environment during the patient's stay (e.g. bedside table,) and did not include studies that focused solely on mobile elements such as doctors' hands, wheelchairs, or medical instruments (e.g. stethoscopes, endoscopes). We excluded equipment surfaces including studies that focused solely on central-line and dialysis. We excluded studies that focused on sink traps, inside of showerheads, and porous surfaces (e.g. curtains, linens). If studies included surfaces in addition to environmental surfaces in the sampling protocol, we included the study.

The critical care environment included all healthcare facilities except veterinary, long-term residential care, and dental facilities. We excluded areas in healthcare facilities that patients would not visit such as laboratory, laundry, and preparatory areas. We excluded long-term care facilities because IPC management and implementation may be different than other healthcare facilities.

Only original, peer-reviewed research was included. Systematic reviews, meta-analyses, poster abstracts, and any conference proceedings were not included.

Outcome measurements had to target organisms from surfaces, rather than from, e.g. air. We included HAI outcomes.

Table S1: PubMed search terms

Date of Search: January 14, 2020

Set	Search Strategy
Set 1: Health Facilities	("Ambulatory Care Facilities"[mh] OR "Ambulatory Care Facilities"[tiab] OR “academic medical centers”[mh] OR “academic medical centers”[tiab] OR “acute care”[tiab] OR “ambulatory surgery center”[tiab] OR “ambulatory surgical centre”[tiab] OR “ambulatory surgical facilities”[tiab] OR “ambulatory surgical facility”[tiab] OR “birth center”[tiab] OR “birth centre”[tiab] OR “birth clinic”[tiab] OR “birth facilities”[tiab] OR “birth facility”[tiab] OR “birthing center”[tiab] OR “birthing centers”[mh] OR “birthing centers”[tiab] OR “birthing centre”[tiab] OR “birthing clinic”[tiab] OR “birthing facilities”[tiab] OR “birthing facility”[tiab] OR “bone marrow transplant department”[tiab] OR “bone marrow transplant unit”[tiab] OR “bone marrow unit”[tiab] OR “burn department”[tiab] OR “burn unit”[tiab] OR “burn ward”[tiab] OR “cancer care center”[tiab] OR “cancer care centre”[tiab] OR “cancer care facilities”[tiab] OR “cancer care facility”[tiab] OR “cardiac clinic”[tiab] OR “cardiac department”[tiab] OR “cardiac unit”[tiab] OR “clinical center”[tiab] OR “clinical centre”[tiab] OR “clinical environment”[tiab] OR “clinical facilities”[tiab] OR “clinical facility”[tiab] OR “community health center”[tiab] OR “Community Health Centers”[mh] OR “community health centre”[tiab] OR “community health clinic”[tiab] OR “community health facilities”[tiab] OR “community health facility”[tiab] OR “community health setting”[tiab] OR “community hospital”[tiab] OR “community hospitals”[tiab] OR “coronary unit”[tiab] OR “dialysis center”[tiab] OR “dialysis centre”[tiab] OR “dialysis facilities”[tiab] OR “dialysis facility”[tiab] OR “elderly care department”[tiab] OR “elderly care unit”[tiab] OR “general hospital”[tiab] OR “general hospitals”[tiab] OR “general medicine department”[tiab] OR “general medicine unit”[tiab] OR “geriatric clinic”[tiab] OR “geriatric department”[tiab] OR “geriatric unit”[tiab] OR “health care center”[tiab] OR “health care centre”[tiab] OR “health care clinic”[tiab] OR “health care clinics”[tiab] OR “health care environment”[tiab] OR “health care facilities”[tiab] OR “health care facility”[tiab] OR “health care setting”[tiab] OR “health center”[tiab] OR “health centre”[tiab] OR “health clinic”[tiab] OR “health facilities, proprietary”[mh] OR “health facilities”[tiab] OR “health facility environment”[mh] OR “health facility environment”[tiab] OR “health facility”[tiab] OR “healthcare center”[tiab] OR “healthcare centre”[tiab] OR “healthcare clinic”[tiab] OR “healthcare environment”[tiab] OR “healthcare facilities”[tiab] OR “healthcare facility”[tiab] OR “healthcare setting”[tiab] OR “hematology department”[tiab] OR “hematology unit”[tiab] OR “hospices”[tiab] OR “hospital environment”[tiab] OR “hospital unit”[tiab] OR “hospital units”[mh]

Set	Search Strategy
	OR “hospital units”[tiab] OR “hospitals, community”[mh] OR “hospitals, general”[mh] OR “hospitals, high-volume”[mh] OR “hospitals, low-volume”[mh] OR “hospitals, private”[mh] OR “hospitals, public”[mh] OR “hospitals, rural”[mh] OR “hospitals, satellite”[mh] OR “hospitals, special”[mh] OR “hospitals, urban”[mh] OR “in patient center”[tiab] OR “in patient centre”[tiab] OR “in patient facilities”[tiab] OR “in patient facility”[tiab] OR “in patient setting”[tiab] OR “inpatient center”[tiab] OR “inpatient centre”[tiab] OR “inpatient facilities”[tiab] OR “inpatient facility”[tiab] OR “inpatient setting”[tiab] OR “isolation hospital”[tiab] OR “isolation room”[tiab] OR “isolation ward”[tiab] OR “medical center”[tiab] OR “medical centre”[tiab] OR “medical clinic”[tiab] OR “medical environment”[tiab] OR “medical facilities”[tiab] OR “medical facility”[tiab] OR “medical setting”[tiab] OR “neonatal department”[tiab] OR “neonatal unit”[tiab] OR “nephrology department”[tiab] OR “nephrology unit”[tiab] OR “neurosurgical department”[tiab] OR “neurosurgical unit”[tiab] OR “NICU”[tiab] OR “oncology department”[tiab] OR “oncology unit”[tiab] OR “open ward area”[tiab] OR “operating department”[tiab] OR “operating room”[tiab] OR “operating suite”[tiab] OR “operating unit”[tiab] OR “operation theater”[tiab] OR “operation theatre”[tiab] OR “out patient center”[tiab] OR “out patient centre”[tiab] OR “out patient clinic”[tiab] OR “out patient facilities”[tiab] OR “out patient facility”[tiab] OR “out patient setting”[tiab] OR “outpatient center”[tiab] OR “outpatient centre”[tiab] OR “outpatient clinic”[tiab] OR “outpatient clinics, hospital”[mh] OR “outpatient facilities”[tiab] OR “outpatient facility”[tiab] OR “outpatient setting”[tiab] OR “patient care area”[tiab] OR “patient care center”[tiab] OR “patient care centre”[tiab] OR “patient care clinic”[tiab] OR “patient care facilities”[tiab] OR “patient care facility”[tiab] OR “patient room”[tiab] OR “PICU”[tiab] OR “private hospital”[tiab] OR “private hospitals”[tiab] OR “proprietary health facilities”[tiab] OR “proprietary health facility”[tiab] OR “public hospital”[tiab] OR “public hospitals”[tiab] OR “recovery room”[tiab] OR “recovery ward”[tiab] OR “satellite hospital”[tiab] OR “satellite hospitals”[tiab] OR “surgical department”[tiab] OR “surgical unit”[tiab] OR “teaching hospital”[tiab] OR “terminal care”[tiab] OR “terminal room”[tiab] OR “urgent care”[tiab] OR “ward bay”[tiab] OR “ward side room”[tiab] OR ”clinical setting”[tiab] OR "critical care"[tiab] OR dispensaries[tiab] OR dispensary[tiab] OR hospice[tiab] OR Hospices[mh] OR Hospices[tiab] OR "intensive care"[tiab] OR policlinic*[tiab] OR surgicenter[tiab])
Set 2: Disinfectants	("1-(diamino methylidene)guanidine"[tiab] OR "1,2,3-triimidodicarbonic diamide"[tiab] OR "1,2-benzisothiazolin-3-one"[tiab] OR "1,2-benzisothiazoline-3-one"[tiab] OR "1,4-butane dialdehyde"[tiab] OR "1,5-Pentanedial"[tiab] OR "2

Set	Search Strategy
	Aminoethanol"[tiab] OR "2-(2-Methoxyethoxy)ethanol"[tiab] OR "2,4,4'-Trichloro-2'-Hydroxydiphenyl Ether"[tiab] OR "2-Aminoethanol"[tiab] OR "2-benzyl-4-chlorophenol"[tiab] OR "2-butoxyethanol"[tiab] OR "2-chloro-5-hydroxy-1,3-dimethylbenzene"[tiab] OR "2-Hydroxy-2',4,4'-trichlorodiphenyl Ether"[tiab] OR "2-hydroxybiphenyl"[tiab] OR "2-hydroxydiphenyl"[tiab] OR "2-phenylphenol"[tiab] OR "3,5-dimethyl-4-chlorophenol"[tiab] OR "3-1 benzoisothiazolin"[tiab] OR "4-(t-butyl)phenol"[tiab] OR "4-chloro-3,5-dimethylphenol sulfonate"[tiab] OR "4-chloro-3,5-dimethylphenol"[tiab] OR "4-tert-butylphenol"[tiab] OR "4-tertiary-butylphenol"[tiab] OR "4-tert-pentyphenol"[tiab] OR "absolute alcohol"[tiab] OR "acetic acid"[tiab] OR "acetyl hydroperoxide"[tiab] OR "active oxygen"[tiab] OR "alcohol based"[tiab] OR "alcohol-based"[tiab] OR "alcoholic solution"[tiab] OR "aldehyde"[tiab] OR "alkyl didecyl dimethyl ammonium chloride"[tiab] OR "alkyl dimethyl benzyl ammonium chloride"[tiab] OR "Alkyldimethylbenzylammonium"[tiab] OR "amidosulfanic acid"[tiab] OR "amino acid disinfectant"[tiab] OR "aminoethanol"[tiab] OR "aminoformamidine hydrochloride"[tiab] OR "aminomethanamidine hydrochloride"[tiab] OR "aminosulfonic acid"[tiab] OR "aminosulfuric acid"[tiab] OR "ammonium chloride"[tiab] OR "ammonium compound"[tiab] OR "ammonium compounds"[tiab] OR "ammonium hydroxide"[tiab] OR "ammonium sulfamate"[tiab] OR "ammonium sulfate"[tiab] OR "amphiprotic disinfectant"[tiab] OR "amphiprotic"[tiab] OR "anthium doxide"[tiab] OR "antiformin"[tiab] OR "antimicrobial coating"[tiab] OR "antimicrobial surface"[tiab] OR "automated cleaning"[tiab] OR "automated decontamination"[tiab] OR "automated disinfectant"[tiab] OR "automated disinfectants"[tiab] OR "automated disinfection"[tiab] OR "bactericidal"[tiab] OR "benzalkonium"[tiab] OR "Benzene-1,2-dicarboxaldehyde"[tiab] OR "Benzenesulfonates"[mh] OR "benzenesulfonic acid"[tiab] OR "benzisothiazolinone"[tiab] OR "benzisothiazolone"[tiab] OR "benzylchlorophenol"[tiab] OR "biguanide"[tiab] OR "biocidal"[tiab] OR "biocide"[tiab] OR "bleach containing"[tiab] OR "bleach"[tiab] OR "bleach-containing"[tiab] OR "bleaching powder"[tiab] OR "buformin"[tiab] OR "butanedial"[tiab] OR "butoxyethanol"[tiab] OR "butyl glycol"[tiab] OR "butylcellosolve"[tiab] OR "butylphen"[tiab] OR "calcium chlorohypochloride"[tiab] OR "calcium chlorohypochlorite"[tiab] OR "calcium dihypochlorite"[tiab] OR "calcium hypochlorite"[tiab] OR "calcium oxychloride"[tiab] OR "carbamidine hydrochloride"[tiab] OR "carbinol"[tiab] OR "carbol"[tiab] OR "carbolic acid"[tiab] OR "carrel-dakin solution"[tiab] OR "caustic soda"[tiab] OR "cetrimide"[tiab] OR "chemical agent"[tiab] OR "chemical agents"[tiab] OR "chloramine T"[tiab] OR "chloramine-T"[tiab] OR "chlorcyanurate"[tiab] OR "chlordesine"[tiab] OR

Set	Search Strategy
	"chlordezine"[tiab] OR "chlorhexidine bigluconate"[tiab] OR "chlorhexidine digluconate"[tiab] OR "chlorinated lime"[tiab] OR "chlorination"[tiab] OR "chlorine based"[tiab] OR "chlorine containing"[tiab] OR "chlorine dioxide"[tiab] OR "chlorine disinfectant"[tiab] OR "chlorine peroxide"[tiab] OR "chlorine releasing"[tiab] OR "chlorine-based"[tiab] OR "chlorine-containing"[tiab] OR "chlorine-releasing"[tiab] OR "chloro-based"[tiab] OR "chlorofene"[tiab] OR "chloroperoxyl"[tiab] OR "chlorophene"[tiab] OR "chlorosyloxidanyl"[tiab] OR "chloroxylenol"[tiab] OR "clorofene"[tiab] OR "clorophene potassium salt"[tiab] OR "clorophene sodium salt"[tiab] OR "clorophene"[tiab] OR "Colamine"[tiab] OR "copper coating"[tiab] OR "copper surface"[tiab] OR "copper-coated"[tiab] OR "copper-silver ionization"[tiab] OR "dakin's solution"[tiab] OR "dakins solution"[tiab] OR "decontaminant"[tiab] OR "decontaminants"[tiab] OR "decontamination"[tiab] OR "dehydrated alcohol"[tiab] OR "dehydrated ethanol"[tiab] OR "denatured alcohol"[tiab] OR "denatured ethanol"[tiab] OR "desoxone 1"[tiab] OR "desoxone1"[tiab] OR "desoxone-1"[tiab] OR "detergent"[tiab] OR "detergents"[tiab] OR "dialkyl dimethyl ammonium chloride"[tiab] OR "dialkyl quaternaires"[tiab] OR "dichloroisocyanuric acid"[tiab] OR "dichlorophenoxy"[tiab] OR "dichloro-s-triazinetrione sodium"[tiab] OR "didecyl dimethyl ammonium bromide"[tiab] OR "didecyldimethylammonium"[tiab] OR "Diethylene glycol methyl ether"[tiab] OR "diethylene glycol monomethyl ether"[tiab] OR "diguanide"[tiab] OR "dihydrogen dioxide"[tiab] OR "dimethylcarbinol"[tiab] OR "dioctyl dimethyl ammonium bromide"[tiab] OR "disinfectant"[tiab] OR "disinfectant-detergent"[tiab] OR "disinfectants"[tiab] OR "Disinfection"[mh] OR "disinfection"[tiab] OR "dodecyl benzene sodium sulfonate"[tiab] OR "dodecyl benzenesulfonic"[tiab] OR "dodecylbenzenesulfonic acid"[tiab] OR "domiphen bromide"[tiab] OR "electrolysed strong acid water"[tiab] OR "electrolysed weak acid water"[tiab] OR "electrolyzed strong acid water"[tiab] OR "electrolyzed water"[tiab] OR "electrolyzed weak acid water"[tiab] OR "environmental cleaning"[tiab] OR "ethanoic acid"[tiab] OR "ethanol"[tiab] OR "ethanolamine"[tiab] OR "ethyl alcohol"[tiab] OR "ethyl hydrate"[tiab] OR "ethyl hydroxide"[tiab] OR "ethyl-alcohol"[tiab] OR "ethylene glycol"[tiab] OR "ethyleneglycol monobutyl ether"[tiab] OR "ethylic acid"[tiab] OR "ethylic alcohol"[tiab] OR "Formaldehyde"[mh] OR "formaldehyde"[tiab] OR "formalin"[tiab] OR "formic aldehyde"[tiab] OR "formol"[tiab] OR "free chlorine"[tiab] OR "fungicidal"[tiab] OR "fungicide"[tiab] OR "germicidal"[tiab] OR "germicide"[tiab] OR "glutaral"[mh] OR "glutaral"[tiab] OR "glutaraldehyde"[tiab] OR "glutardialdehyde"[tiab] OR "glutaric acid dialdehyde"[tiab] OR "glutaric aldehyde"[tiab] OR

Set	Search Strategy
	"glutaric dialdehyde"[tiab] OR "grain alcohol"[tiab] OR "guanidine hydrochloride"[tiab] OR "Guanidine Monohydrate"[tiab] OR "Guanidine Monohydrobromide"[tiab] OR "Guanidine Monohydrochloride"[tiab] OR "Guanidine Monohydroiodine"[tiab] OR "Guanidine Nitrate"[tiab] OR "Guanidine Phosphate"[tiab] OR "Guanidine Sulfate"[tiab] OR "Guanidine Sulfite"[tiab] OR "guanidine"[tiab] OR "Guanidinium Chloride"[tiab] OR "Guanidinium"[tiab] OR "Guanidium Chloride"[tiab] OR "guanylguanidine"[tiab] OR "heavy metal coating"[tiab] OR "heavy metal surface"[tiab] OR "hydrochloride"[tiab] OR "hydrogen peroxide"[mh] OR "hydrogen peroxide"[tiab] OR "hydroperoxide"[tiab] OR "hydroxybenzene"[tiab] OR "hydroxyethane"[tiab] OR "hydroxylamine"[tiab] OR "hypochloride"[tiab] OR "hypochlorite sodium"[tiab] OR "hypochlorite"[tiab] OR "hypochlorous acid"[tiab] OR "imidodicarbonimidic diamide"[tiab] OR "iodophor"[tiab] OR "iodophors"[mh] OR "isopropanol"[tiab] OR "isopropyl alcohol"[tiab] OR "javel water"[tiab] OR "javelle water"[tiab] OR "light activated coating"[tiab] OR "light activated disinfection"[tiab] OR "light activated surface"[tiab] OR "light-activated coating"[tiab] OR "light-activated disinfection"[tiab] OR "light-activated surface"[tiab] OR "lunar caustic"[tiab] OR "metal alloy coating"[tiab] OR "metal alloy surface"[tiab] OR "metformin"[tiab] OR "methanal"[tiab] OR "methanecarboxylic acid"[tiab] OR "methanol"[tiab] OR "methoxydiglycol"[tiab] OR "methyl alcohol"[tiab] OR "methyl aldehyde"[tiab] OR "methyl carbitol"[tiab] OR "methyl dioxitol"[tiab] OR "methyl hydrate"[tiab] OR "methyl hydroxide"[tiab] OR "methylcarbinol"[tiab] OR "methylene oxide"[tiab] OR "methylethyl alcohol"[tiab] OR "methylol"[tiab] OR "mono peracetic acid"[tiab] OR "monochloramine T"[tiab] OR "monoethanolamine"[tiab] OR "monohydrochloride"[tiab] OR "monohydroxymethane"[tiab] OR "monoperacetic acid"[tiab] OR "mycobactericidal"[tiab] OR "N,N'-(1,10-decanediyldi-1-(4H)-pyridinyl-4-ylidene)bis-(1-octamine) dihydrochloride"[tiab] OR "no touch cleaning"[tiab] OR "no touch decontamination"[tiab] OR "no touch disinfectant"[tiab] OR "no touch disinfectants"[tiab] OR "no touch disinfection"[tiab] OR "nonsporicidal"[tiab] OR "no-touch cleaning"[tiab] OR "no-touch decontamination"[tiab] OR "no-touch disinfectant"[tiab] OR "no-touch disinfectants"[tiab] OR "no-touch disinfection"[tiab] OR "o Phthalaldehyde"[tiab] OR "o Phthaldialdehyde"[tiab] OR "o-benzyl-p-chlorophenol"[tiab] OR "octanamine"[tiab] OR "octenidine hydrochloride"[tiab] OR "octenidine"[tiab] OR "o-phenylphenate"[tiab] OR "o-phenylphenol"[tiab] OR "o-phthalaldehyde"[mh] OR "o-Phthaldialdehyde"[tiab] OR "o-phthalic dicarboxaldehyde"[tiab] OR "organosilane"[tiab] OR "organosilane-

Set	Search Strategy
	treated"[tiab] OR "organosilicon"[tiab] OR "ortho Phthalaldehyde"[tiab] OR "ortho Phthalic Aldehyde"[tiab] OR "ortho-benzyl parachlorophenol"[tiab] OR "orthobenzylparachlorophenol"[tiab] OR "ortho-benzyl-para-chlorophenol"[tiab] OR "ortho-phenyl phenol"[tiab] OR "ortho-phenylphenate"[tiab] OR "orthophenylphenol"[tiab] OR "ortho-phenylphenol"[tiab] OR "orthophthalaldehyde"[tiab] OR "ortho-Phthalaldehyde"[tiab] OR "Orthophthaldialdehyde"[tiab] OR "ortho-Phthalic Aldehyde"[tiab] OR "oxidizing agent"[tiab] OR "oxomethane"[tiab] OR "oxomethylene"[tiab] OR "oxymethylene"[tiab] OR "parachlorometaxylenol"[tiab] OR "paraform"[tiab] OR "paraformaldehyde"[tiab] OR "para-tertiary amylphenol"[tiab] OR "para-tertiary butylphenol"[tiab] OR "para-tertiary-amylphenol"[tiab] OR "para-tertiary-amyl-phenol"[tiab] OR "para-tertiary-butylphenol"[tiab] OR "p-chloro-m-xylenol"[tiab] OR "PCMX"[tiab] OR "peracetic acid"[tiab] OR "perhydrol"[tiab] OR "peroxyacetic acid"[tiab] OR "peroxyethanoic acid"[tiab] OR "peroxygen"[tiab] OR "phenformin"[tiab] OR "phenol"[tiab] OR "phenolate sodium"[tiab] OR "phenolic"[tiab] OR "phenolics"[tiab] OR "phthalaldehyde"[tiab] OR "polyvinylpyrrolidone iodine"[tiab] OR "potassium dichloro-s-triazinetrione"[tiab] OR "povidone iodine"[tiab] OR "povidone-iodine"[tiab] OR "pressurized steam"[tiab] OR "pro oxidant"[tiab] OR "proguanil"[tiab] OR "pro-oxidant"[tiab] OR "propanol"[tiab] OR "propyl alcohol"[tiab] OR "*Pseudomonas*"[tiab] OR "p-tert-amylphenol"[tiab] OR "p-tert-butylphenol"[tiab] OR "pulsed ultrasound"[tiab] OR "pulsed ultraviolet"[tiab] OR "pulsed xenon"[tiab] OR "pvp iodine"[tiab] OR "PVP-I"[tiab] OR "PVP-iodine"[tiab] OR "pyroxylic"[tiab] OR "quaternary amine"[tiab] OR "quaternary ammonium compounds"[mh] OR "quaternary ammonium"[tiab] OR "reactive oxygen species"[mh] OR "reactive oxygen"[tiab] OR "rubbing alcohol"[tiab] OR "self disinfecting coating"[tiab] OR "self disinfecting surface"[tiab] OR "self-disinfecting coating"[tiab] OR "self-disinfecting surface"[tiab] OR "silver coating"[tiab] OR "silver nitrate"[tiab] OR "silver surface"[tiab] OR "silver-coated"[tiab] OR "sodium dichloroisocyanurate"[tiab] OR "sodium dichloro-s-triazine-2,4,6(1H,3H,5H)-trione"[tiab] OR "sodium dodecyl benzene sulfonate"[tiab] OR "sodium dodecylbenzenesulfonate"[tiab] OR "sodium hydroxide"[tiab] OR "sodium hypochlorite"[mh] OR "sodium laurylbenzenesulfonate"[tiab] OR "sodium methoxide"[tiab] OR "sodium o-phenylphenoate"[tiab] OR "sodium ortho-phenylphenate"[tiab] OR "sodium oxychloride"[tiab] OR "sodium peracetate"[tiab] OR "sodium phenolate"[tiab] OR "sodium p-toluenesulfonchloramide"[tiab] OR "sodium tosylchloramide"[tiab] OR "sodium ortho-phenylphenol"[tiab] OR "sporicidal"[tiab] OR "sporicide"[tiab] OR "steam cleaner"[tiab] OR "succinaldehyde"[tiab] OR "succinic aldehyde"[tiab] OR "succinic dialdehyde"[tiab] OR

Set	Search Strategy
	"sulfamate"[tiab] OR "sulfamic acid"[tiab] OR "sulfaminic acid"[tiab] OR "sulfonol"[tiab] OR "super oxidized solution"[tiab] OR "super oxidized water"[tiab] OR "superoxidized solution"[tiab] OR "super-oxidized solution"[tiab] OR "superoxidized water"[tiab] OR "surgical chlorinated soda solution"[tiab] OR "tamed iodine"[tiab] OR "tosylchloramide sodium"[tiab] OR "touchless cleaning"[tiab] OR "touchless decontamination"[tiab] OR "touchless disinfectant"[tiab] OR "touchless disinfectants"[tiab] OR "touchless disinfection"[tiab] OR "triclosan"[tiab] OR "trimethylammonium"[tiab] OR "troclosene"[tiab] OR "troclosenum natricum"[tiab] OR "tuberculocidal"[tiab] OR "tuberculocide"[tiab] OR "twin-chain quaternaires"[tiab] OR "ultraviolet cleaning"[tiab] OR "ultraviolet decontamination"[tiab] OR "ultraviolet disinfectant"[tiab] OR "ultraviolet disinfectants"[tiab] OR "ultraviolet disinfection"[tiab] OR "UV cleaning"[tiab] OR "UV decontamination"[tiab] OR "UV disinfectant"[tiab] OR "UV disinfectants"[tiab] OR "UV disinfection"[tiab] OR "UVC cleaning"[tiab] OR "UV-C cleaning"[tiab] OR "UVC decontamination"[tiab] OR "UV-C decontamination"[tiab] OR "UVC disinfectant"[tiab] OR "UV-C disinfectant"[tiab] OR "UVC disinfectants"[tiab] OR "UV-C disinfectants"[tiab] OR "UVC disinfection"[tiab] OR "UV-C disinfection"[tiab] OR "vinegar"[tiab] OR "virucidal"[tiab] OR "virucide"[tiab] OR "wood alcohol"[tiab] OR "wood naphtha"[tiab] OR "wood spirit"[tiab] OR "zinc peracetate"[tiab])
Additional Limits	NOT (“comment”[Publication Type] OR “editorial”[Publication Type] OR “review”[Publication Type])

<u>Table S2: Web of Science search terms</u>

Date of Search: January 15, 2020

Set	Search Strategy
Set 1: Health Facilities	("Ambulatory Care Facilities" OR “academic medical centers” OR “acute care” OR “ambulatory surgery center” OR “ambulatory surgical centre” OR “ambulatory surgical facilities” OR “ambulatory surgical facility” OR “birth center” OR “birth centre” OR “birth clinic” OR “birth facilities” OR “birth facility” OR “birthing center” OR “birthing centers” OR “birthing centre” OR “birthing clinic” OR “birthing facilities” OR “birthing facility” OR “bone marrow transplant department” OR “bone marrow transplant unit” OR “bone marrow unit” OR “burn department” OR “burn unit” OR “burn ward” OR “cancer care center” OR “cancer care centre” OR “cancer care facilities” OR “cancer care facility” OR “cardiac clinic” OR “cardiac department” OR “cardiac unit” OR “clinical center” OR “clinical centre” OR “clinical environment” OR “clinical facilities” OR “clinical facility” OR “community health center” OR “community health centre” OR

Set	Search Strategy
	“community health clinic” OR “community health facilities” OR “community health facility” OR “community health setting” OR “community hospital” OR “community hospitals” OR “coronary unit” OR “dialysis center” OR “dialysis centre” OR “dialysis facilities” OR “dialysis facility” OR “elderly care department” OR “elderly care unit” OR “general hospital” OR “general hospitals” OR “general medicine department” OR “general medicine unit” OR “geriatric clinic” OR “geriatric department” OR “geriatric unit” OR “health care center” OR “health care centre” OR “health care clinic” OR “health care clinics” OR “health care environment” OR “health care facilities” OR “health care facility” OR “health care setting” OR “health center” OR “health centre” OR “health clinic” OR “health facilities” OR “health facility environment” OR “health facility” OR “healthcare center” OR “healthcare centre” OR “healthcare clinic” OR “healthcare environment” OR “healthcare facilities” OR “healthcare facility” OR “healthcare setting” OR “hematology department” OR “hematology unit” OR “hospices” OR “hospital environment” OR “hospital unit” OR “hospital units” OR “in patient center” OR “in patient centre” OR “in patient facilities” OR “in patient facility” OR “in patient setting” OR “inpatient center” OR “inpatient centre” OR “inpatient facilities” OR “inpatient facility” OR “inpatient setting” OR “isolation hospital” OR “isolation room” OR “isolation ward” OR “medical center” OR “medical centre” OR “medical clinic” OR “medical environment” OR “medical facilities” OR “medical facility” OR “medical setting” OR “neonatal department” OR “neonatal unit” OR “nephrology department” OR “nephrology unit” OR “neurosurgical department” OR “neurosurgical unit” OR “NICU” OR “oncology department” OR “oncology unit” OR “open ward area” OR “operating department” OR “operating room” OR “operating suite” OR “operating unit” OR “operation theater” OR “operation theatre” OR “out patient center” OR “out patient centre” OR “out patient clinic” OR “out patient facilities” OR “out patient facility” OR “out patient setting” OR “outpatient center” OR “outpatient centre” OR “outpatient clinic” OR “outpatient facilities” OR “outpatient facility” OR “outpatient setting” OR “patient care area” OR “patient care center” OR “patient care centre” OR “patient care clinic” OR “patient care facilities” OR “patient care facility” OR “patient room” OR “PICU” OR “private hospital” OR “private hospitals” OR “proprietary health facilities” OR “proprietary health facility” OR “public hospital” OR “public hospitals” OR “recovery room” OR “recovery ward” OR “satellite hospital” OR “satellite hospitals” OR “surgical department” OR “surgical unit” OR “teaching hospital” OR “terminal care” OR “terminal room” OR “urgent care” OR “ward bay” OR “ward side room” OR ”clinical setting” OR "critical care" OR dispensaries OR dispensary OR hospice OR Hospices OR "intensive care" OR policlinic* OR surgicenter)

Set	Search Strategy
Set 2: Disinfectants	("1-(diamino methylidene)guanidine" OR "1,2,3-triimidodicarbonic diamide" OR "1,2-benzisothiazolin-3-one" OR "1,2-benzisothiazoline-3-one" OR "1,4-butane dialdehyde" OR "1,5-Pentanedial" OR "2 Aminoethanol" OR "2-(2-Methoxyethoxy)ethanol" OR "2,4,4'-Trichloro-2'-Hydroxydiphenyl Ether" OR "2-Aminoethanol" OR "2-benzyl-4-chlorophenol" OR "2-butoxyethanol" OR "2-chloro-5-hydroxy-1,3-dimethylbenzene" OR "2-Hydroxy-2',4,4'-trichlorodiphenyl Ether" OR "2-hydroxybiphenyl" OR "2-hydroxydiphenyl" OR "2-phenylphenol" OR "3,5-dimethyl-4-chlorophenol" OR "3-1 benzoisothiazolin" OR "4-(t-butyl)phenol" OR "4-chloro-3,5-dimethylphenol sulfonate" OR "4-chloro-3,5-dimethylphenol" OR "4-tert-butylphenol" OR "4-tertiary-butylphenol" OR "4-tert-pentyphenol" OR "absolute alcohol" OR "acetic acid" OR "acetyl hydroperoxide" OR "active oxygen" OR "alcohol based" OR "alcohol-based" OR "alcoholic solution" OR "aldehyde" OR "alkyl didecyl dimethyl ammonium chloride" OR "alkyl dimethyl benzyl ammonium chloride" OR "Alkyldimethylbenzylammonium" OR "amidosulfanic acid" OR "amino acid disinfectant" OR "aminoethanol" OR "aminoformamidine hydrochloride" OR "aminomethanamidine hydrochloride" OR "aminosulfonic acid" OR "aminosulfuric acid" OR "ammonium chloride" OR "ammonium compound" OR "ammonium compounds" OR "ammonium hydroxide" OR "ammonium sulfamate" OR "ammonium sulfate" OR "amphiprotic disinfectant" OR "amphiprotic" OR "anthium doxide" OR "antiformin" OR "antimicrobial coating" OR "antimicrobial surface" OR "automated cleaning" OR "automated decontamination" OR "automated disinfectant" OR "automated disinfectants" OR "automated disinfection" OR "bactericidal" OR "benzalkonium" OR "Benzene-1,2-dicarboxaldehyde" OR "benzenesulfonic acid" OR "benzisothiazolinone" OR "benzisothiazolone" OR "benzylchlorophenol" OR "biguanide" OR "biocidal" OR "biocide" OR "bleach containing" OR "bleach" OR "bleach-containing" OR "bleaching powder" OR "buformin" OR "butanedial" OR "butoxyethanol" OR "butyl glycol" OR "butylcellosolve" OR "butylphen" OR "calcium chlorohypochloride" OR "calcium chlorohypochlorite" OR "calcium dihypochlorite" OR "calcium hypochlorite" OR "calcium oxychloride" OR "carbamidine hydrochloride" OR "carbinol" OR "carbol" OR "carbolic acid" OR "carrel-dakin solution" OR "caustic soda" OR "cetrimide" OR "chemical agent" OR "chemical agents" OR "chloramine T" OR "chloramine-T" OR "chlorcyanurate" OR "chlordesine" OR "chlordezine" OR "chlorhexidine bigluconate" OR "chlorhexidine digluconate" OR "chlorinated lime" OR "chlorination" OR "chlorine based" OR "chlorine containing" OR "chlorine dioxide" OR "chlorine disinfectant" OR "chlorine peroxide" OR "chlorine releasing" OR "chlorine-based" OR

Set	Search Strategy
	"chlorine-containing" OR "chlorine-releasing" OR "chloro-based" OR "chlorofene" OR "chloroperoxyl" OR "chlorophene" OR "chlorosyloxidanyl" OR "chloroxylenol" OR "clorofene" OR "clorophene potassium salt" OR "clorophene sodium salt" OR "clorophene" OR "Colamine" OR "copper coating" OR "copper surface" OR "copper-coated" OR "copper-silver ionization" OR "dakin's solution" OR "dakins solution" OR "decontaminant" OR "decontaminants" OR "decontamination" OR "dehydrated alcohol" OR "dehydrated ethanol" OR "denatured alcohol" OR "denatured ethanol" OR "desoxone 1" OR "desoxone1" OR "desoxone-1" OR "detergent" OR "detergents" OR "dialkyl dimethyl ammonium chloride" OR "dialkyl quaternaires" OR "dichloroisocyanuric acid" OR "dichlorophenoxy" OR "dichloro-s-triazinetrione sodium" OR "didecyl dimethyl ammonium bromide" OR "didecyldimethylammonium" OR "Diethylene glycol methyl ether" OR "diethylene glycol monomethyl ether" OR "diguanide" OR "dihydrogen dioxide" OR "dimethylcarbinol" OR "dioctyl dimethyl ammonium bromide" OR "disinfectant" OR "disinfectant-detergent" OR "disinfectants" OR "disinfection" OR "dodecyl benzene sodium sulfonate" OR "dodecyl benzenesulfonic" OR "dodecylbenzenesulfonic acid" OR "domiphen bromide" OR "electrolysed strong acid water" OR "electrolysed weak acid water" OR "electrolyzed strong acid water" OR "electrolyzed water" OR "electrolyzed weak acid water" OR "environmental cleaning" OR "ethanoic acid" OR "ethanol" OR "ethanolamine" OR "ethyl alcohol" OR "ethyl hydrate" OR "ethyl hydroxide" OR "ethyl-alcohol" OR "ethylene glycol" OR "ethyleneglycol monobutyl ether" OR "ethylic acid" OR "ethylic alcohol" OR "formaldehyde" OR "formalin" OR "formic aldehyde" OR "formol" OR "free chlorine" OR "fungicidal" OR "fungicide" OR "germicidal" OR "germicide" OR "glutaral" OR "glutaraldehyde" OR "glutardialdehyde" OR "glutaric acid dialdehyde" OR "glutaric aldehyde" OR "glutaric dialdehyde" OR "grain alcohol" OR "guanidine hydrochloride" OR "Guanidine Monohydrate" OR "Guanidine Monohydrobromide" OR "Guanidine Monohydrochloride" OR "Guanidine Monohydroiodine" OR "Guanidine Nitrate" OR "Guanidine Phosphate" OR "Guanidine Sulfate" OR "Guanidine Sulfite" OR "guanidine" OR "Guanidinium Chloride" OR "Guanidinium" OR "Guanidium Chloride" OR "guanylguanidine" OR "heavy metal coating" OR "heavy metal surface" OR "hydrochloride" OR "hydrogen peroxide" OR "hydroperoxide" OR "hydroxybenzene" OR "hydroxyethane" OR "hydroxylamine" OR "hypochloride" OR "hypochlorite sodium" OR "hypochlorite" OR "hypochlorous acid" OR "imidodicarbonimidic diamide" OR "iodophor" OR "isopropanol" OR "isopropyl alcohol" OR "javel water" OR "javelle water" OR "light activated coating" OR "light activated disinfection" OR "light activated surface" OR "light-activated

Set	Search Strategy
	coating" OR "light-activated disinfection" OR "light-activated surface" OR "lunar caustic" OR "metal alloy coating" OR "metal alloy surface" OR "metformin" OR "methanal" OR "methanecarboxylic acid" OR "methanol" OR "methoxydiglycol" OR "methyl alcohol" OR "methyl aldehyde" OR "methyl carbitol" OR "methyl dioxitol" OR "methyl hydrate" OR "methyl hydroxide" OR "methylcarbinol" OR "methylene oxide" OR "methylethyl alcohol" OR "methylol" OR "mono peracetic acid" OR "monochloramine T" OR "monoethanolamine" OR "monohydrochloride" OR "monohydroxymethane" OR "monoperacetic acid" OR "mycobactericidal" OR "N,N'-(1,10-decanediyldi-1-(4H)-pyridinyl-4-ylidene)bis-(1-octamine) dihydrochloride" OR "no touch cleaning" OR "no touch decontamination" OR "no touch disinfectant" OR "no touch disinfectants" OR "no touch disinfection" OR "nonsporicidal" OR "no-touch cleaning" OR "no-touch decontamination" OR "no-touch disinfectant" OR "no-touch disinfectants" OR "no-touch disinfection" OR "o Phthalaldehyde" OR "o Phthaldialdehyde" OR "o-benzyl-p-chlorophenol" OR "octanamine" OR "octenidine hydrochloride" OR "octenidine" OR "o-phenylphenate" OR "o-phenylphenol" OR "o-Phthaldialdehyde" OR "o-phthalic dicarboxaldehyde" OR "organosilane" OR "organosilane-treated" OR "organosilicon" OR "ortho Phthalaldehyde" OR "ortho Phthalic Aldehyde" OR "ortho-benzyl parachlorophenol" OR "orthobenzylparachlorophenol" OR "ortho-benzyl-para-chlorophenol" OR "ortho-phenyl phenol" OR "ortho-phenylphenate" OR "orthophenylphenol" OR "ortho-phenylphenol" OR "orthophthalaldehyde" OR "ortho-Phthalaldehyde" OR "Orthophthaldialdehyde" OR "ortho-Phthalic Aldehyde" OR "oxidizing agent" OR "oxomethane" OR "oxomethylene" OR "oxymethylene" OR "parachlorometaxylenol" OR "paraform" OR "paraformaldehyde" OR "para-tertiary amylphenol" OR "para-tertiary butylphenol" OR "para-tertiary-amylphenol" OR "para-tertiary-amyl-phenol" OR "para-tertiary-butylphenol" OR "p-chloro-m-xylenol" OR "PCMX" OR "peracetic acid" OR "perhydrol" OR "peroxyacetic acid" OR "peroxyethanoic acid" OR "peroxygen" OR "phenformin" OR "phenol" OR "phenolate sodium" OR "phenolic" OR "phenolics" OR "phthalaldehyde" OR "polyvinylpyrrolidone iodine" OR "potassium dichloro-s-triazinetrione" OR "povidone iodine" OR "povidone-iodine" OR "pressurized steam" OR "pro oxidant" OR "proguanil" OR "pro-oxidant" OR "propanol" OR "propyl alcohol" OR "*Pseudomonas*" OR "p-tert-amylphenol" OR "p-tert-butylphenol" OR "pulsed ultrasound" OR "pulsed ultraviolet" OR "pulsed xenon" OR "pvp iodine" OR "PVP-I" OR "PVP-iodine" OR "pyroxylic" OR "quaternary amine" OR "quaternary ammonium" OR "reactive oxygen" OR "rubbing alcohol" OR "self disinfecting coating" OR "self disinfecting surface" OR "self-disinfecting coating" OR "self-disinfecting surface" OR "silver coating" OR "silver nitrate" OR "silver

Set	Search Strategy
	surface" OR "silver-coated" OR "sodium dichloroisocyanurate" OR "sodium dichloro-s-triazine-2,4,6(1H,3H,5H)-trione" OR "sodium dodecyl benzene sulfonate" OR "sodium dodecylbenzenesulfonate" OR "sodium hydroxide" OR "sodium laurylbenzenesulfonate" OR "sodium methoxide" OR "sodium o-phenylphenoate" OR "sodium ortho-phenylphenate" OR "sodium oxychloride" OR "sodium peracetate" OR "sodium phenolate" OR "sodium p-toluenesulfonchloramide" OR "sodium tosylchloramide" OR "sodium ortho-phenylphenol" OR "sporicidal" OR "sporicide" OR "steam cleaner" OR "succinaldehyde" OR "succinic aldehyde" OR "succinic dialdehyde" OR "sulfamate" OR "sulfamic acid" OR "sulfaminic acid" OR "sulfonol" OR "super oxidized solution" OR "super oxidized water" OR "superoxidized solution" OR "super-oxidized solution" OR "superoxidized water" OR "surgical chlorinated soda solution" OR "tamed iodine" OR "tosylchloramide sodium" OR "touchless cleaning" OR "touchless decontamination" OR "touchless disinfectant" OR "touchless disinfectants" OR "touchless disinfection" OR "triclosan" OR "trimethylammonium" OR "troclosene" OR "troclosenum natricum" OR "tuberculocidal" OR "tuberculocide" OR "twin-chain quaternaires" OR "ultraviolet cleaning" OR "ultraviolet decontamination" OR "ultraviolet disinfectant" OR "ultraviolet disinfectants" OR "ultraviolet disinfection" OR "UV cleaning" OR "UV decontamination" OR "UV disinfectant" OR "UV disinfectants" OR "UV disinfection" OR "UVC cleaning" OR "UV-C cleaning" OR "UVC decontamination" OR "UV-C decontamination" OR "UVC disinfectant" OR "UV-C disinfectant" OR "UVC disinfectants" OR "UV-C disinfectants" OR "UVC disinfection" OR "UV-C disinfection" OR "vinegar" OR "virucidal" OR "virucide" OR "wood alcohol" OR "wood naphtha" OR "wood spirit" OR "zinc peracetate")
Additional limits	Articles, Conference Papers, Conference Abstracts last 2 years; exclude reviews, commentaries, etc.

Table S3: Scopus search terms

Date of Search: January 15, 2020

Set	Search Strategy
Set 1: Health Facilities	("Ambulatory Care Facilities" OR “academic medical centers” OR “acute care” OR “ambulatory surgery center” OR “ambulatory surgical centre” OR “ambulatory surgical facilities” OR “ambulatory surgical facility” OR “birth center” OR “birth centre” OR “birth clinic” OR “birth facilities” OR “birth facility” OR “birthing center” OR “birthing centers” OR “birthing centre” OR “birthing clinic” OR “birthing facilities” OR “birthing facility” OR “bone marrow transplant department” OR “bone marrow transplant unit” OR “bone marrow unit” OR “burn department” OR “burn unit” OR “burn ward” OR “cancer care center” OR “cancer care centre” OR “cancer care facilities” OR

Set	Search Strategy
	"cancer care facility" OR "cardiac clinic" OR "cardiac department" OR "cardiac unit" OR "clinical center" OR "clinical centre" OR "clinical environment" OR "clinical facilities" OR "clinical facility" OR "community health center" OR "community health centre" OR "community health clinic" OR "community health facilities" OR "community health facility" OR "community health setting" OR "community hospital" OR "community hospitals" OR "coronary unit" OR "dialysis center" OR "dialysis centre" OR "dialysis facilities" OR "dialysis facility" OR "elderly care department" OR "elderly care unit" OR "general hospital" OR "general hospitals" OR "general medicine department" OR "general medicine unit" OR "geriatric clinic" OR "geriatric department" OR "geriatric unit" OR "health care center" OR "health care centre" OR "health care clinic" OR "health care clinics" OR "health care environment" OR "health care facilities" OR "health care facility" OR "health care setting" OR "health center" OR "health centre" OR "health clinic" OR "health facilities" OR "health facility environment" OR "health facility" OR "healthcare center" OR "healthcare centre" OR "healthcare clinic" OR "healthcare environment" OR "healthcare facilities" OR "healthcare facility" OR "healthcare setting" OR "hematology department" OR "hematology unit" OR "hospices" OR "hospital environment" OR "hospital unit" OR "hospital units" OR "in patient center" OR "in patient centre" OR "in patient facilities" OR "in patient facility" OR "in patient setting" OR "inpatient center" OR "inpatient centre" OR "inpatient facilities" OR "inpatient facility" OR "inpatient setting" OR "isolation hospital" OR "isolation room" OR "isolation ward" OR "medical center" OR "medical centre" OR "medical clinic" OR "medical environment" OR "medical facilities" OR "medical facility" OR "medical setting" OR "neonatal department" OR "neonatal unit" OR "nephrology department" OR "nephrology unit" OR "neurosurgical department" OR "neurosurgical unit" OR "NICU" OR "oncology department" OR "oncology unit" OR "open ward area" OR "operating department" OR "operating room" OR "operating suite" OR "operating unit" OR "operation theater" OR "operation theatre" OR "out patient center" OR "out patient centre" OR "out patient clinic" OR "out patient facilities" OR "out patient facility" OR "out patient setting" OR "outpatient center" OR "outpatient centre" OR "outpatient clinic" OR "outpatient facilities" OR "outpatient facility" OR "outpatient setting" OR "patient care area" OR "patient care center" OR "patient care centre" OR "patient care clinic" OR "patient care facilities" OR "patient care facility" OR "patient room" OR "PICU" OR "private hospital" OR "private hospitals" OR "proprietary health facilities" OR "proprietary health facility" OR "public hospital" OR "public hospitals" OR "recovery room" OR "recovery ward" OR "satellite hospital" OR "satellite hospitals" OR "surgical department" OR "surgical unit" OR

Set	Search Strategy
	“teaching hospital” OR “terminal care” OR “terminal room” OR “urgent care” OR “ward bay” OR “ward side room” OR ”clinical setting” OR "critical care" OR dispensaries OR dispensary OR hospice OR Hospices OR "intensive care" OR policlinic* OR surgicenter)
Set 2: Disinfectants	("1-(diamino methylidene)guanidine" OR "1,2,3-triimidodicarbonic diamide" OR "1,2-benzisothiazolin-3-one" OR "1,2-benzisothiazoline-3-one" OR "1,4-butane dialdehyde" OR "1,5-Pentanedial" OR "2 Aminoethanol" OR "2-(2-Methoxyethoxy)ethanol" OR "2,4,4'-Trichloro-2'-Hydroxydiphenyl Ether" OR "2-Aminoethanol" OR "2-benzyl-4-chlorophenol" OR "2-butoxyethanol" OR "2-chloro-5-hydroxy-1,3-dimethylbenzene" OR "2-Hydroxy-2',4,4'-trichlorodiphenyl Ether" OR "2-hydroxybiphenyl" OR "2-hydroxydiphenyl" OR "2-phenylphenol" OR "3,5-dimethyl-4-chlorophenol" OR "3-1 benzoisothiazolin" OR "4-(t-butyl)phenol" OR "4-chloro-3,5-dimethylphenol sulfonate" OR "4-chloro-3,5-dimethylphenol" OR "4-tert-butylphenol" OR "4-tertiary-butylphenol" OR "4-tert-pentyphenol" OR "absolute alcohol" OR "acetic acid" OR "acetyl hydroperoxide" OR "active oxygen" OR "alcohol based" OR "alcohol-based" OR "alcoholic solution" OR "aldehyde" OR "alkyl didecyl dimethyl ammonium chloride" OR "alkyl dimethyl benzyl ammonium chloride" OR "Alkyldimethylbenzylammonium" OR "amidosulfanic acid" OR "amino acid disinfectant" OR "aminoethanol" OR "aminoformamidine hydrochloride" OR "aminomethanamidine hydrochloride" OR "aminosulfonic acid" OR "aminosulfuric acid" OR "ammonium chloride" OR "ammonium compound" OR "ammonium compounds" OR "ammonium hydroxide" OR "ammonium sulfamate" OR "ammonium sulfate" OR "amphiprotic disinfectant" OR "amphiprotic" OR "anthium doxide" OR "antiformin" OR "antimicrobial coating" OR "antimicrobial surface" OR "automated cleaning" OR "automated decontamination" OR "automated disinfectant" OR "automated disinfectants" OR "automated disinfection" OR "bactericidal" OR "benzalkonium" OR "Benzene-1,2-dicarboxaldehyde" OR "benzenesulfonic acid" OR "benzisothiazolinone" OR "benzisothiazolone" OR "benzylchlorophenol" OR "biguanide" OR "biocidal" OR "biocide" OR "bleach containing" OR "bleach" OR "bleach-containing" OR "bleaching powder" OR "buformin" OR "butanedial" OR "butoxyethanol" OR "butyl glycol" OR "butylcellosolve" OR "butylphen" OR "calcium chlorohypochloride" OR "calcium chlorohypochlorite" OR "calcium dihypochlorite" OR "calcium hypochlorite" OR "calcium oxychloride" OR "carbamidine hydrochloride" OR "carbinol" OR "carbol" OR "carbolic acid" OR "carrel-dakin solution" OR "caustic soda" OR "cetrimide" OR "chemical agent" OR "chemical agents" OR "chloramine T" OR "chloramine-T" OR "chlorcyanurate" OR "chlordesine" OR "chlordezine" OR

Set	Search Strategy
	"chlorhexidine bigluconate" OR "chlorhexidine digluconate" OR "chlorinated lime" OR "chlorination" OR "chlorine based" OR "chlorine containing" OR "chlorine dioxide" OR "chlorine disinfectant" OR "chlorine peroxide" OR "chlorine releasing" OR "chlorine-based" OR "chlorine-containing" OR "chlorine-releasing" OR "chloro-based" OR "chlorofene" OR "chloroperoxyl" OR "chlorophene" OR "chlorosyloxidanyl" OR "chloroxylenol" OR "clorofene" OR "clorophene potassium salt" OR "clorophene sodium salt" OR "clorophene" OR "Colamine" OR "copper coating" OR "copper surface" OR "copper-coated" OR "copper-silver ionization" OR "dakin’s solution" OR "dakins solution" OR "decontaminant" OR "decontaminants" OR "decontamination" OR "dehydrated alcohol" OR "dehydrated ethanol" OR "denatured alcohol" OR "denatured ethanol" OR "desoxone 1" OR "desoxone1" OR "desoxone-1" OR "detergent" OR "detergents" OR "dialkyl dimethyl ammonium chloride" OR "dialkyl quaternaires" OR "dichloroisocyanuric acid" OR "dichlorophenoxy" OR "dichloro-s-triazinetrione sodium" OR "didecyl dimethyl ammonium bromide" OR "didecyldimethylammonium" OR "Diethylene glycol methyl ether" OR "diethylene glycol monomethyl ether" OR "diguanide" OR "dihydrogen dioxide" OR "dimethylcarbinol" OR "dioctyl dimethyl ammonium bromide" OR "disinfectant" OR "disinfectant-detergent" OR "disinfectants" OR "disinfection" OR "dodecyl benzene sodium sulfonate" OR "dodecyl benzenesulfonic" OR "dodecylbenzenesulfonic acid" OR "domiphen bromide" OR "electrolysed strong acid water" OR "electrolysed weak acid water" OR "electrolyzed strong acid water" OR "electrolyzed water" OR "electrolyzed weak acid water" OR "environmental cleaning" OR "ethanoic acid" OR "ethanol" OR "ethanolamine" OR "ethyl alcohol" OR "ethyl hydrate" OR "ethyl hydroxide" OR "ethyl-alcohol" OR "ethylene glycol" OR "ethyleneglycol monobutyl ether" OR "ethylic acid" OR "ethylic alcohol" OR "formaldehyde" OR "formalin" OR "formic aldehyde" OR "formol" OR "free chlorine" OR "fungicidal" OR "fungicide" OR "germicidal" OR "germicide" OR "glutaral" OR "glutaraldehyde" OR "glutardialdehyde" OR "glutaric acid dialdehyde" OR "glutaric aldehyde" OR "glutaric dialdehyde" OR "grain alcohol" OR "guanidine hydrochloride" OR "Guanidine Monohydrate" OR "Guanidine Monohydrobromide" OR "Guanidine Monohydrochloride" OR "Guanidine Monohydroiodine" OR "Guanidine Nitrate" OR "Guanidine Phosphate" OR "Guanidine Sulfate" OR "Guanidine Sulfite" OR "guanidine" OR "Guanidinium Chloride" OR "Guanidinium" OR "Guanidium Chloride" OR "guanylguanidine" OR "heavy metal coating" OR "heavy metal surface" OR "hydrochloride" OR "hydrogen peroxide" OR "hydroperoxide" OR "hydroxybenzene" OR "hydroxyethane" OR "hydroxylamine" OR "hypochloride" OR "hypochlorite sodium" OR

Set	Search Strategy
	"hypochlorite" OR "hypochlorous acid" OR "imidodicarbonimidic diamide" OR "iodophor" OR "isopropanol" OR "isopropyl alcohol" OR "javel water" OR "javelle water" OR "light activated coating" OR "light activated disinfection" OR "light activated surface" OR "light-activated coating" OR "light-activated disinfection" OR "light-activated surface" OR "lunar caustic" OR "metal alloy coating" OR "metal alloy surface" OR "metformin" OR "methanal" OR "methanecarboxylic acid" OR "methanol" OR "methoxydiglycol" OR "methyl alcohol" OR "methyl aldehyde" OR "methyl carbitol" OR "methyl dioxitol" OR "methyl hydrate" OR "methyl hydroxide" OR "methylcarbinol" OR "methylene oxide" OR "methylethyl alcohol" OR "methylol" OR "mono peracetic acid" OR "monochloramine T" OR "monoethanolamine" OR "monohydrochloride" OR "monohydroxymethane" OR "monoperacetic acid" OR "mycobactericidal" OR "N,N'-(1,10-decanediyldi-1-(4H)-pyridinyl-4-ylidene)bis-(1-octamine) dihydrochloride" OR "no touch cleaning" OR "no touch decontamination" OR "no touch disinfectant" OR "no touch disinfectants" OR "no touch disinfection" OR "nonsporicidal" OR "no-touch cleaning" OR "no-touch decontamination" OR "no-touch disinfectant" OR "no-touch disinfectants" OR "no-touch disinfection" OR "o Phthalaldehyde" OR "o Phthaldialdehyde" OR "o-benzyl-p-chlorophenol" OR "octanamine" OR "octenidine hydrochloride" OR "octenidine" OR "o-phenylphenate" OR "o-phenylphenol" OR "o-Phthaldialdehyde" OR "o-phthalic dicarboxaldehyde" OR "organosilane" OR "organosilane-treated" OR "organosilicon" OR "ortho Phthalaldehyde" OR "ortho Phthalic Aldehyde" OR "ortho-benzyl parachlorophenol" OR "orthobenzylparachlorophenol" OR "ortho-benzyl-para-chlorophenol" OR "ortho-phenyl phenol" OR "ortho-phenylphenate" OR "orthophenylphenol" OR "ortho-phenylphenol" OR "orthophthalaldehyde" OR "ortho-Phthalaldehyde" OR "Orthophthaldialdehyde" OR "ortho-Phthalic Aldehyde" OR "oxidizing agent" OR "oxomethane" OR "oxomethylene" OR "oxymethylene" OR "parachlorometaxylenol" OR "paraform" OR "paraformaldehyde" OR "para-tertiary amylphenol" OR "para-tertiary butylphenol" OR "para-tertiary-amylphenol" OR "para-tertiary-amyl-phenol" OR "para-tertiary-butylphenol" OR "p-chloro-m-xylenol" OR "PCMX" OR "peracetic acid" OR "perhydrol" OR "peroxyacetic acid" OR "peroxyethanoic acid" OR "peroxygen" OR "phenformin" OR "phenol" OR "phenolate sodium" OR "phenolic" OR "phenolics" OR "phthalaldehyde" OR "polyvinylpyrrolidone iodine" OR "potassium dichloro-s-triazinetrione" OR "povidone iodine" OR "povidone-iodine" OR "pressurized steam" OR "pro oxidant" OR "proguanil" OR "pro-oxidant" OR "propanol" OR "propyl alcohol" OR "*Pseudomonas*" OR "p-tert-amylphenol" OR "p-tert-butylphenol" OR "pulsed ultrasound" OR "pulsed ultraviolet" OR "pulsed xenon" OR "pvp iodine" OR "PVP-I" OR "PVP-iodine" OR

Set	Search Strategy
	"pyroxylic" OR "quaternary amine" OR "quaternary ammonium" OR "reactive oxygen" OR "rubbing alcohol" OR "self disinfecting coating" OR "self disinfecting surface" OR "self-disinfecting coating" OR "self-disinfecting surface" OR "silver coating" OR "silver nitrate" OR "silver surface" OR "silver-coated" OR "sodium dichloroisocyanurate" OR "sodium dichloro-s-triazine-2,4,6(1H,3H,5H)-trione" OR "sodium dodecyl benzene sulfonate" OR "sodium dodecylbenzenesulfonate" OR "sodium hydroxide" OR "sodium laurylbenzenesulfonate" OR "sodium methoxide" OR "sodium o-phenylphenoate" OR "sodium ortho-phenylphenate" OR "sodium oxychloride" OR "sodium peracetate" OR "sodium phenolate" OR "sodium p-toluenesulfonchloramide" OR "sodium tosylchloramide" OR "sodium ortho-phenylphenol" OR "sporicidal" OR "sporicide" OR "steam cleaner" OR "succinaldehyde" OR "succinic aldehyde" OR "succinic dialdehyde" OR "sulfamate" OR "sulfamic acid" OR "sulfaminic acid" OR "sulfonol" OR "super oxidized solution" OR "super oxidized water" OR "superoxidized solution" OR "super-oxidized solution" OR "superoxidized water" OR "surgical chlorinated soda solution" OR "tamed iodine" OR "tosylchloramide sodium" OR "touchless cleaning" OR "touchless decontamination" OR "touchless disinfectant" OR "touchless disinfectants" OR "touchless disinfection" OR "triclosan" OR "trimethylammonium" OR "troclosene" OR "troclosenum natricum" OR "tuberculocidal" OR "tuberculocide" OR "twin-chain quaternaires" OR "ultraviolet cleaning" OR "ultraviolet decontamination" OR "ultraviolet disinfectant" OR "ultraviolet disinfectants" OR "ultraviolet disinfection" OR "UV cleaning" OR "UV decontamination" OR "UV disinfectant" OR "UV disinfectants" OR "UV disinfection" OR "UVC cleaning" OR "UV-C cleaning" OR "UVC decontamination" OR "UV-C decontamination" OR "UVC disinfectant" OR "UV-C disinfectant" OR "UVC disinfectants" OR "UV-C disinfectants" OR "UVC disinfection" OR "UV-C disinfection" OR "vinegar" OR "virucidal" OR "virucide" OR "wood alcohol" OR "wood naphtha" OR "wood spirit" OR "zinc peracetate")
Additional limits	Articles, Conference Papers; exclude reviews, commentaries, etc. (Limited to TI-AB)

<u>Table S4: Embase search terms</u>

Date of Search: January 15, 2020

Set	Search Strategy
Set 1: Health Facilities	('ambulatory care facilities':ab,ti OR 'university hospital'/exp OR 'academic medical centers':ab,ti OR 'acute care':ab,ti OR 'ambulatory surgery center':ab,ti OR 'ambulatory surgical centre':ab,ti OR 'ambulatory surgical facilities':ab,ti OR 'ambulatory surgical facility':ab,ti OR 'birth center':ab,ti OR 'birth centre':ab,ti OR 'birth clinic':ab,ti OR 'birth facilities':ab,ti OR 'birth facility':ab,ti OR

Set	Search Strategy
	'birthing center':ab,ti OR 'maternity ward'/exp OR 'birthing centers':ab,ti OR 'birthing centre':ab,ti OR 'birthing clinic':ab,ti OR 'birthing facilities':ab,ti OR 'birthing facility':ab,ti OR 'bone marrow transplant department':ab,ti OR 'bone marrow transplant unit':ab,ti OR 'bone marrow unit':ab,ti OR 'burn department':ab,ti OR 'burn unit':ab,ti OR 'burn ward':ab,ti OR 'cancer care center':ab,ti OR 'cancer care centre':ab,ti OR 'cancer care facilities':ab,ti OR 'cancer care facility':ab,ti OR 'cardiac clinic':ab,ti OR 'cardiac department':ab,ti OR 'cardiac unit':ab,ti OR 'clinical center':ab,ti OR 'clinical centre':ab,ti OR 'clinical environment':ab,ti OR 'clinical facilities':ab,ti OR 'clinical facility':ab,ti OR 'community health center':ab,ti OR 'health center'/exp OR 'community health centre':ab,ti OR 'community health clinic':ab,ti OR 'community health facilities':ab,ti OR 'community health facility':ab,ti OR 'community health setting':ab,ti OR 'community hospital':ab,ti OR 'community hospitals':ab,ti OR 'coronary unit':ab,ti OR 'dialysis center':ab,ti OR 'dialysis centre':ab,ti OR 'dialysis facilities':ab,ti OR 'dialysis facility':ab,ti OR 'elderly care department':ab,ti OR 'elderly care unit':ab,ti OR 'general hospital':ab,ti OR 'general hospitals':ab,ti OR 'general medicine department':ab,ti OR 'general medicine unit':ab,ti OR 'geriatric clinic':ab,ti OR 'geriatric department':ab,ti OR 'geriatric unit':ab,ti OR 'health care center':ab,ti OR 'health care centre':ab,ti OR 'health care clinic':ab,ti OR 'health care clinics':ab,ti OR 'health care environment':ab,ti OR 'health care facilities':ab,ti OR 'health care facility':ab,ti OR 'health care setting':ab,ti OR 'health center':ab,ti OR 'health centre':ab,ti OR 'health clinic':ab,ti OR 'health care facility'/exp OR 'health facilities':ab,ti OR 'health facility environment':ab,ti OR 'health facility':ab,ti OR 'healthcare center':ab,ti OR 'healthcare centre':ab,ti OR 'healthcare clinic':ab,ti OR 'healthcare environment':ab,ti OR 'healthcare facilities':ab,ti OR 'healthcare facility':ab,ti OR 'healthcare setting':ab,ti OR 'hematology department':ab,ti OR 'hematology unit':ab,ti OR 'hospices':ab,ti OR 'hospital environment':ab,ti OR 'hospital unit':ab,ti OR 'hospital subdivisions and components'/exp OR 'hospital units':ab,ti OR 'community hospital'/exp OR 'general hospital'/exp OR 'high volume hospital'/exp OR 'low volume hospital'/exp OR 'private hospital'/exp OR 'public hospital'/exp OR 'rural hospital'/exp OR 'hospital'/exp OR 'in patient center':ab,ti OR 'in patient centre':ab,ti OR 'in patient facilities':ab,ti OR 'in patient facility':ab,ti OR 'in patient setting':ab,ti OR 'inpatient center':ab,ti OR 'inpatient centre':ab,ti OR 'inpatient facilities':ab,ti OR 'inpatient facility':ab,ti OR 'inpatient setting':ab,ti OR 'isolation hospital':ab,ti OR 'isolation room':ab,ti OR 'isolation ward':ab,ti OR 'medical center':ab,ti OR 'medical centre':ab,ti OR 'medical clinic':ab,ti OR 'medical environment':ab,ti OR 'medical facilities':ab,ti OR 'medical facility':ab,ti OR 'medical setting':ab,ti OR 'neonatal department':ab,ti OR 'neonatal unit':ab,ti OR 'nephrology

Set	Search Strategy
	department':ab,ti OR 'nephrology unit':ab,ti OR 'neurosurgical department':ab,ti OR 'neurosurgical unit':ab,ti OR 'nicu':ab,ti OR 'oncology department':ab,ti OR 'oncology unit':ab,ti OR 'open ward area':ab,ti OR 'operating department':ab,ti OR 'operating room':ab,ti OR 'operating suite':ab,ti OR 'operating unit':ab,ti OR 'operation theater':ab,ti OR 'operation theatre':ab,ti OR 'out patient center':ab,ti OR 'out patient centre':ab,ti OR 'out patient clinic':ab,ti OR 'out patient facilities':ab,ti OR 'out patient facility':ab,ti OR 'out patient setting':ab,ti OR 'outpatient center':ab,ti OR 'outpatient centre':ab,ti OR 'outpatient clinic':ab,ti OR 'outpatient department'/exp OR 'outpatient facilities':ab,ti OR 'outpatient facility':ab,ti OR 'outpatient setting':ab,ti OR 'patient care area':ab,ti OR 'patient care center':ab,ti OR 'patient care centre':ab,ti OR 'patient care clinic':ab,ti OR 'patient care facilities':ab,ti OR 'patient care facility':ab,ti OR 'patient room':ab,ti OR 'picu':ab,ti OR 'private hospital':ab,ti OR 'private hospitals':ab,ti OR 'proprietary health facilities':ab,ti OR 'proprietary health facility':ab,ti OR 'public hospital':ab,ti OR 'public hospitals':ab,ti OR 'recovery room':ab,ti OR 'recovery ward':ab,ti OR 'satellite hospital':ab,ti OR 'satellite hospitals':ab,ti OR 'surgical department':ab,ti OR 'surgical unit':ab,ti OR 'teaching hospital':ab,ti OR 'terminal care':ab,ti OR 'terminal room':ab,ti OR 'urgent care':ab,ti OR 'ward bay':ab,ti OR 'ward side room':ab,ti OR 'clinical setting':ab,ti OR 'critical care':ab,ti OR dispensaries:ab,ti OR dispensary:ab,ti OR hospice:ab,ti OR 'hospice'/exp OR hospices:ab,ti OR 'intensive care':ab,ti OR policlinic*:ab,ti OR surgicenter:ab,ti) AND [embase]/lim
Set 2: Disinfectants	("1-(diamino methylidene)guanidine":ab,ti OR "1,2,3-triimidodicarbonic diamide":ab,ti OR "1,2-benzisothiazolin-3-one":ab,ti OR "1,2-benzisothiazoline-3-one":ab,ti OR "1,4-butane dialdehyde":ab,ti OR "1,5-Pentanedial":ab,ti OR "2 Aminoethanol":ab,ti OR "2-(2-Methoxyethoxy)ethanol":ab,ti OR "2-Aminoethanol":ab,ti OR "2-benzyl-4-chlorophenol":ab,ti OR "2-butoxyethanol":ab,ti OR "2-chloro-5-hydroxy-1,3-dimethylbenzene":ab,ti OR "2-hydroxybiphenyl":ab,ti OR "2-hydroxydiphenyl":ab,ti OR "2-phenylphenol":ab,ti OR "3,5-dimethyl-4-chlorophenol":ab,ti OR "3-1 benzoisothiazolin":ab,ti OR "4-(t-butyl)phenol":ab,ti OR "4-chloro-3,5-dimethylphenol sulfonate":ab,ti OR "4-chloro-3,5-dimethylphenol":ab,ti OR "4-tert-butylphenol":ab,ti OR "4-tertiary-butylphenol":ab,ti OR "4-tert-pentyphenol":ab,ti OR "absolute alcohol":ab,ti OR "acetic acid":ab,ti OR "acetyl hydroperoxide":ab,ti OR "active oxygen":ab,ti OR "alcohol based":ab,ti OR "alcohol-based":ab,ti OR "alcoholic solution":ab,ti OR "aldehyde":ab,ti OR "alkyl didecyl dimethyl ammonium chloride":ab,ti OR "alkyl dimethyl benzyl ammonium chloride":ab,ti OR "Alkyldimethylbenzylammonium":ab,ti OR "amidosulfanic acid":ab,ti OR "amino acid disinfectant":ab,ti OR "aminoethanol":ab,ti OR

Set	Search Strategy
	"aminoformamidine hydrochloride":ab,ti OR "aminomethanamidine hydrochloride":ab,ti OR "aminosulfonic acid":ab,ti OR "aminosulfuric acid":ab,ti OR "ammonium chloride":ab,ti OR "ammonium compound":ab,ti OR "ammonium compounds":ab,ti OR "ammonium hydroxide":ab,ti OR "ammonium sulfamate":ab,ti OR "ammonium sulfate":ab,ti OR "amphiprotic disinfectant":ab,ti OR "amphiprotic":ab,ti OR "anthium doxide":ab,ti OR "antiformin":ab,ti OR "antimicrobial coating":ab,ti OR "antimicrobial surface":ab,ti OR "automated cleaning":ab,ti OR "automated decontamination":ab,ti OR "automated disinfectant":ab,ti OR "automated disinfectants":ab,ti OR "automated disinfection":ab,ti OR "bactericidal":ab,ti OR "benzalkonium":ab,ti OR "Benzene-1,2-dicarboxaldehyde":ab,ti OR "benzenesulfonic acid derivative"/exp OR "benzenesulfonic acid":ab,ti OR "benzisothiazolinone":ab,ti OR "benzisothiazolone":ab,ti OR "benzylchlorophenol":ab,ti OR "biguanide":ab,ti OR "biocidal":ab,ti OR "biocide":ab,ti OR "bleach containing":ab,ti OR "bleach":ab,ti OR "bleach-containing":ab,ti OR "bleaching powder":ab,ti OR "buformin":ab,ti OR "butanedial":ab,ti OR "butoxyethanol":ab,ti OR "butyl glycol":ab,ti OR "butylcellosolve":ab,ti OR "butylphen":ab,ti OR "calcium chlorohypochloride":ab,ti OR "calcium chlorohypochlorite":ab,ti OR "calcium dihypochlorite":ab,ti OR "calcium hypochlorite":ab,ti OR "calcium oxychloride":ab,ti OR "carbamidine hydrochloride":ab,ti OR "carbinol":ab,ti OR "carbol":ab,ti OR "carbolic acid":ab,ti OR "carrel-dakin solution":ab,ti OR "caustic soda":ab,ti OR "cetrimide":ab,ti OR "chemical agent":ab,ti OR "chemical agents":ab,ti OR "chloramine T":ab,ti OR "chloramine-T":ab,ti OR "chlorcyanurate":ab,ti OR "chlordesine":ab,ti OR "chlordezine":ab,ti OR "chlorhexidine bigluconate":ab,ti OR "chlorhexidine digluconate":ab,ti OR "chlorinated lime":ab,ti OR "chlorination":ab,ti OR "chlorine based":ab,ti OR "chlorine containing":ab,ti OR "chlorine dioxide":ab,ti OR "chlorine disinfectant":ab,ti OR "chlorine peroxide":ab,ti OR "chlorine releasing":ab,ti OR "chlorine-based":ab,ti OR "chlorine-containing":ab,ti OR "chlorine-releasing":ab,ti OR "chloro-based":ab,ti OR "chlorofene":ab,ti OR "chloroperoxyl":ab,ti OR "chlorophene":ab,ti OR "chlorosyloxidanyl":ab,ti OR "chloroxylenol":ab,ti OR "clorofene":ab,ti OR "clorophene potassium salt":ab,ti OR "clorophene sodium salt":ab,ti OR "clorophene":ab,ti OR "Colamine":ab,ti OR "copper coating":ab,ti OR "copper surface":ab,ti OR "copper-coated":ab,ti OR "copper-silver ionization":ab,ti OR "dakins solution":ab,ti OR "decontaminant":ab,ti OR "decontaminants":ab,ti OR "decontamination":ab,ti OR "dehydrated alcohol":ab,ti OR "dehydrated ethanol":ab,ti OR "denatured alcohol":ab,ti OR "denatured ethanol":ab,ti OR "desoxone 1":ab,ti OR "desoxone1":ab,ti OR "desoxone-1":ab,ti OR "detergent":ab,ti OR

Set	Search Strategy
	"detergents":ab,ti OR "dialkyl dimethyl ammonium chloride":ab,ti OR "dialkyl quaternaires":ab,ti OR "dichloroisocyanuric acid":ab,ti OR "dichlorophenoxy":ab,ti OR "dichloro-s-triazinetrione sodium":ab,ti OR "didecyl dimethyl ammonium bromide":ab,ti OR "didecyldimethylammonium":ab,ti OR "Diethylene glycol methyl ether":ab,ti OR "diethylene glycol monomethyl ether":ab,ti OR "diguanide":ab,ti OR "dihydrogen dioxide":ab,ti OR "dimethylcarbinol":ab,ti OR "dioctyl dimethyl ammonium bromide":ab,ti OR "disinfectant":ab,ti OR "disinfectant-detergent":ab,ti OR "disinfectants":ab,ti OR "disinfection"/exp OR "disinfection":ab,ti OR "dodecyl benzene sodium sulfonate":ab,ti OR "dodecyl benzenesulfonic":ab,ti OR "dodecylbenzenesulfonic acid":ab,ti OR "domiphen bromide":ab,ti OR "electrolysed strong acid water":ab,ti OR "electrolysed weak acid water":ab,ti OR "electrolyzed strong acid water":ab,ti OR "electrolyzed water":ab,ti OR "electrolyzed weak acid water":ab,ti OR "environmental cleaning":ab,ti OR "ethanoic acid":ab,ti OR "ethanol":ab,ti OR "ethanolamine":ab,ti OR "ethyl alcohol":ab,ti OR "ethyl hydrate":ab,ti OR "ethyl hydroxide":ab,ti OR "ethyl-alcohol":ab,ti OR "ethylene glycol":ab,ti OR "ethyleneglycol monobutyl ether":ab,ti OR "ethylic acid":ab,ti OR "ethylic alcohol":ab,ti OR "formaldehyde"/exp OR "formaldehyde":ab,ti OR "formalin":ab,ti OR "formic aldehyde":ab,ti OR "formol":ab,ti OR "free chlorine":ab,ti OR "fungicidal":ab,ti OR "fungicide":ab,ti OR "germicidal":ab,ti OR "germicide":ab,ti OR "glutaraldehyde"/exp OR "glutaral":ab,ti OR "glutaraldehyde":ab,ti OR "glutardialdehyde":ab,ti OR "glutaric acid dialdehyde":ab,ti OR "glutaric aldehyde":ab,ti OR "glutaric dialdehyde":ab,ti OR "grain alcohol":ab,ti OR "guanidine hydrochloride":ab,ti OR "Guanidine Monohydrate":ab,ti OR "Guanidine Monohydrobromide":ab,ti OR "Guanidine Monohydrochloride":ab,ti OR "Guanidine Monohydroiodine":ab,ti OR "Guanidine Nitrate":ab,ti OR "Guanidine Phosphate":ab,ti OR "Guanidine Sulfate":ab,ti OR "Guanidine Sulfite":ab,ti OR "guanidine":ab,ti OR "Guanidinium Chloride":ab,ti OR "Guanidinium":ab,ti OR "Guanidium Chloride":ab,ti OR "guanylguanidine":ab,ti OR "heavy metal coating":ab,ti OR "heavy metal surface":ab,ti OR "hydrochloride":ab,ti OR "hydrogen peroxide"/exp OR "hydrogen peroxide":ab,ti OR "hydroperoxide":ab,ti OR "hydroxybenzene":ab,ti OR "hydroxyethane":ab,ti OR "hydroxylamine":ab,ti OR "hypochloride":ab,ti OR "hypochlorite sodium":ab,ti OR "hypochlorite":ab,ti OR "hypochlorous acid":ab,ti OR "imidodicarbonimidic diamide":ab,ti OR "iodophor":ab,ti OR "iodophor"/exp OR "isopropanol":ab,ti OR "isopropyl alcohol":ab,ti OR "javel water":ab,ti OR "javelle water":ab,ti OR "light activated coating":ab,ti OR "light activated disinfection":ab,ti OR "light

Set	Search Strategy
	activated surface":ab,ti OR "light-activated coating":ab,ti OR "light-activated disinfection":ab,ti OR "light-activated surface":ab,ti OR "lunar caustic":ab,ti OR "metal alloy coating":ab,ti OR "metal alloy surface":ab,ti OR "metformin":ab,ti OR "methanal":ab,ti OR "methanecarboxylic acid":ab,ti OR "methanol":ab,ti OR "methoxydiglycol":ab,ti OR "methyl alcohol":ab,ti OR "methyl aldehyde":ab,ti OR "methyl carbitol":ab,ti OR "methyl dioxitol":ab,ti OR "methyl hydrate":ab,ti OR "methyl hydroxide":ab,ti OR "methylcarbinol":ab,ti OR "methylene oxide":ab,ti OR "methylethyl alcohol":ab,ti OR "methylol":ab,ti OR "mono peracetic acid":ab,ti OR "monochloramine T":ab,ti OR "monoethanolamine":ab,ti OR "monohydrochloride":ab,ti OR "monohydroxymethane":ab,ti OR "monoperacetic acid":ab,ti OR "mycobactericidal":ab,ti OR "no touch cleaning":ab,ti OR "no touch decontamination":ab,ti OR "no touch disinfectant":ab,ti OR "no touch disinfectants":ab,ti OR "no touch disinfection":ab,ti OR "nonsporicidal":ab,ti OR "no-touch cleaning":ab,ti OR "no-touch decontamination":ab,ti OR "no-touch disinfectant":ab,ti OR "no-touch disinfectants":ab,ti OR "no-touch disinfection":ab,ti OR "o Phthalaldehyde":ab,ti OR "o Phthaldialdehyde":ab,ti OR "o-benzyl-p-chlorophenol":ab,ti OR "octanamine":ab,ti OR "octenidine hydrochloride":ab,ti OR "octenidine":ab,ti OR "o-phenylphenate":ab,ti OR "o-phenylphenol":ab,ti OR "phthalaldehyde"/exp OR "o-Phthaldialdehyde":ab,ti OR "o-phthalic dicarboxaldehyde":ab,ti OR "organosilane":ab,ti OR "organosilane-treated":ab,ti OR "organosilicon":ab,ti OR "ortho Phthalaldehyde":ab,ti OR "ortho Phthalic Aldehyde":ab,ti OR "ortho-benzyl parachlorophenol":ab,ti OR "orthobenzylparachlorophenol":ab,ti OR "ortho-benzyl-para-chlorophenol":ab,ti OR "ortho-phenyl phenol":ab,ti OR "ortho-phenylphenate":ab,ti OR "orthophenylphenol":ab,ti OR "ortho-phenylphenol":ab,ti OR "orthophthalaldehyde":ab,ti OR "ortho-Phthalaldehyde":ab,ti OR "Orthophthaldialdehyde":ab,ti OR "ortho-Phthalic Aldehyde":ab,ti OR "oxidizing agent":ab,ti OR "oxomethane":ab,ti OR "oxomethylene":ab,ti OR "oxymethylene":ab,ti OR "parachlorometaxylenol":ab,ti OR "paraform":ab,ti OR "paraformaldehyde":ab,ti OR "para-tertiary amylphenol":ab,ti OR "para-tertiary butylphenol":ab,ti OR "para-tertiary-amylphenol":ab,ti OR "para-tertiary-amyl-phenol":ab,ti OR "para-tertiary-butylphenol":ab,ti OR "p-chloro-m-xylenol":ab,ti OR "PCMX":ab,ti OR "peracetic acid":ab,ti OR "perhydrol":ab,ti OR "peroxyacetic acid":ab,ti OR "peroxyethanoic acid":ab,ti OR "peroxygen":ab,ti OR "phenformin":ab,ti OR "phenol":ab,ti OR "phenolate sodium":ab,ti OR "phenolic":ab,ti OR "phenolics":ab,ti OR "phthalaldehyde":ab,ti OR "polyvinylpyrrolidone iodine":ab,ti OR "potassium dichloro-s-triazinetrione":ab,ti OR "povidone iodine":ab,ti OR "povidone-

Set	Search Strategy
	iodine":ab,ti OR "pressurized steam":ab,ti OR "pro oxidant":ab,ti OR "proguanil":ab,ti OR "pro-oxidant":ab,ti OR "propanol":ab,ti OR "propyl alcohol":ab,ti OR "*Pseudomonas*":ab,ti OR "p-tert-amylphenol":ab,ti OR "p-tert-butylphenol":ab,ti OR "pulsed ultrasound":ab,ti OR "pulsed ultraviolet":ab,ti OR "pulsed xenon":ab,ti OR "pvp iodine":ab,ti OR "PVP-I":ab,ti OR "PVP-iodine":ab,ti OR "pyroxylic":ab,ti OR "quaternary amine":ab,ti OR "quaternary ammonium derivative"/exp OR "quaternary ammonium":ab,ti OR "reactive oxygen metabolite"/exp OR "reactive oxygen":ab,ti OR "rubbing alcohol":ab,ti OR "self disinfecting coating":ab,ti OR "self disinfecting surface":ab,ti OR "self-disinfecting coating":ab,ti OR "self-disinfecting surface":ab,ti OR "silver coating":ab,ti OR "silver nitrate":ab,ti OR "silver surface":ab,ti OR "silver-coated":ab,ti OR "sodium dichloroisocyanurate":ab,ti OR "sodium dichloro-s-triazine-2,4,6(1H,3H,5H)-trione":ab,ti OR "sodium dodecyl benzene sulfonate":ab,ti OR "sodium dodecylbenzenesulfonate":ab,ti OR "sodium hydroxide":ab,ti OR "hypochlorite sodium"/exp OR "sodium laurylbenzenesulfonate":ab,ti OR "sodium methoxide":ab,ti OR "sodium o-phenylphenoate":ab,ti OR "sodium ortho-phenylphenate":ab,ti OR "sodium oxychloride":ab,ti OR "sodium peracetate":ab,ti OR "sodium phenolate":ab,ti OR "sodium p-toluenesulfonchloramide":ab,ti OR "sodium tosylchloramide":ab,ti OR "sodium ortho-phenylphenol":ab,ti OR "sporicidal":ab,ti OR "sporicide":ab,ti OR "steam cleaner":ab,ti OR "succinaldehyde":ab,ti OR "succinic aldehyde":ab,ti OR "succinic dialdehyde":ab,ti OR "sulfamate":ab,ti OR "sulfamic acid":ab,ti OR "sulfaminic acid":ab,ti OR "sulfonol":ab,ti OR "super oxidized solution":ab,ti OR "super oxidized water":ab,ti OR "superoxidized solution":ab,ti OR "super-oxidized solution":ab,ti OR "superoxidized water":ab,ti OR "surgical chlorinated soda solution":ab,ti OR "tamed iodine":ab,ti OR "tosylchloramide sodium":ab,ti OR "touchless cleaning":ab,ti OR "touchless decontamination":ab,ti OR "touchless disinfectant":ab,ti OR "touchless disinfectants":ab,ti OR "touchless disinfection":ab,ti OR "triclosan":ab,ti OR "trimethylammonium":ab,ti OR "troclosene":ab,ti OR "troclosenum natricum":ab,ti OR "tuberculocidal":ab,ti OR "tuberculocide":ab,ti OR "twin-chain quaternaires":ab,ti OR "ultraviolet cleaning":ab,ti OR "ultraviolet decontamination":ab,ti OR "ultraviolet disinfectant":ab,ti OR "ultraviolet disinfectants":ab,ti OR "ultraviolet disinfection":ab,ti OR "UV cleaning":ab,ti OR "UV decontamination":ab,ti OR "UV disinfectant":ab,ti OR "UV disinfectants":ab,ti OR "UV disinfection":ab,ti OR "UVC cleaning":ab,ti OR "UV-C cleaning":ab,ti OR "UVC decontamination":ab,ti OR "UV-C decontamination":ab,ti OR "UVC disinfectant":ab,ti OR "UV-C disinfectant":ab,ti OR "UVC disinfectants":ab,ti OR "UV-C disinfectants":ab,ti OR "UVC

Set	Search Strategy
	disinfection":ab,ti OR "UV-C disinfection":ab,ti OR "vinegar":ab,ti OR "virucidal":ab,ti OR "virucide":ab,ti OR "wood alcohol":ab,ti OR "wood naphtha":ab,ti OR "wood spirit":ab,ti OR "zinc peracetate":ab,ti)
Additional Limits	(#5 AND [embase]/lim NOT ([embase]/lim AND [medline]/lim) AND (2018:py OR 2019:py OR 2020:py) AND 'conference abstract'/it) OR (#5 AND [embase]/lim NOT ([embase]/lim AND [medline]/lim) AND ('article'/it OR 'article in press'/it OR 'conference paper'/it))<br><br>We limited conference abstracts to those published in 2018, 2019, or 2020. We combined these results with other results. Reviews were excluded.

Supplementary Material 2: List of Included Studies and Study Quality Assessment

Table of Contents

Table S5: Study quality indicator definitions for 14-point study quality assessment and average values for each indicator
Table S6: References for all included studies and study ID
Table S7: Individual study quality assessment data
Table S8: Cohen's kappa and raw percent agreement for inter-rater variability

Table S5: Study quality indicator definitions for 14-point study quality assessment and average values for each indicator

Study Quality Indicator	Bias Type	Question	Criteria for 1	Criteria for 0.5	Criteria for 0	% criteria 1	% criteria 0.5	% criteria 0
1	Study type	Is the aim of the study natural, synthetic, or not well-defined?	Natural study	Seeded study *in situ*	Not well defined	93%	6%	0%
2	Setting	Was the setting clearly specified and defined (w.r.t. department/unit type and surface/patient)?	Has both	Misses one	Misses more than two	73%	23%	3%
3	Contemporary groups	Control group included and measured at same time as comparator?	Contemporary/ simultaneous comparison	No contemporary comparison (e.g. "before" is the control)	No comparison at all	45%	49%	3%
4	Baseline equivalence	Were surfaces or patients in experimental groups selected from similar settings?	Department/unit and surfaces/patients are the same (e.g. any intensive care/operating room) for all interventions?	There is a question as to whether they are comparable	Not comparable (e.g. MICU vs. out-patient)	77%	20%	2%
5	Baseline outcome prevalence	Was exposure to outcome (bacteria or HAI) measured prior to interventions?	Measured baseline health of patient or surface bioburden either as longitudinal natural study or amount prior to treatment	External/hospital baseline data	Not measured	76%	8%	15%
6	Intervention description, methods	Were interventions clearly defined, valid, reliable?	Pre-cleaning methods described, disinfection methods described including concentration (or trade name), contact time, surface type	Missing one of the above	Missing more than one above	44%	38%	17%
7	Low bias due to deviation from protocol	Were disinfectant methods implemented consistently across all sites/groups?	Measured implementation (e.g. fluorescence)	Updated staff/re-training	Not mentioned	38%	23%	38%

8	Outcome methods	Was outcome clearly defined, valid, measured reliably, implemented consistently across study?	Environmental sampling to confirm pathogen. Clearly defined and consistent.	Mixed methods: e.g. molecular vs. culture; OR outcome measurements with ATP or fluorescence	Outcome described but poor methods description	85%	9%	6%
9	Blind evaluation	Were healthcare workers/microbiologists aware of intervention for a given surface?	Both were blinded	Healthcare workers OR microbiologists were blinded	Not reported or neither	3%	12%	85%
10	Low bias due to missing data	Low loss of follow up/outcomes	Reported whether there were missing data and a follow-up analysis if >20% loss consistent across groups	Reported whether there were missing data; no follow-up analysis if >20% loss consistent across groups	Not reported	8%	3%	90%
11	Correction for confounding	Were confounding variables measured or adjusted for their impact ?	Considering confounding from, e.g., initial organic/bacterial load, antibiotic use, etc., the study attempts to control for these	Major confounder not accounted for in study design or external data	More than one major confounder not accounted for	27%	36%	35%
12	Reporting based on aim	Was there bias in selection of the reported results and do conclusions follow from aim?	Results show all surface types, all measured bacteria/disease AND outcomes are logical and in-line with study aims	Outcomes are mostly reported OR outcomes tangential to purpose of study	Multiple outcomes of study aims are unreported, null results unreported, OR outcomes NOT to related to study aim	62%	32%	4%
13	Analysis	Were statistical analyses described and variance/effect estimates provided?	Credible statistical analysis including confidence intervals or p-values AND measure of variance or power calculation	Valid statistical test was performed with p-value OR CIs/variance included	Intervention not compared to control	30%	49%	18%
14	Funding conflict of interest	Did a disinfection manufacturer fund the study? If yes, is there a statement identifying	Academic/government funding	Commercial/corporate/non-profit (anything else) funding WITH statement of influence clarified	Commercial/corporate/other funding WITHOUT statement that funders did	34%	20%	45%

		role in study design and reporting of results?			not play role in design/implementation			

Table S6: References for included studies and systematic review ID

Study ID	Full Author List	Year	Title
67	Afinogenova, A. G.; Kraeva, L. A.; Afinogenov, G. E.; Veretennikov, V. V.	2017	Probiotic-based sanitation as alternatives to chemical disinfectants
111	Carling, P. C.; Perkins, J.; Ferguson, J.; Thomasser, A.	2014	Evaluating a new paradigm for comparing surface disinfection in clinical practice
125	Casey, A. L.; Adams, D.; Karpanen, T. J.; Lambert, P. A.; Cookson, B. D.; Nightingale, P.; Miruszenko, L.; Shillam, R.; Christian, P.; Elliott, T. S.	2010	Role of copper in reducing hospital environment contamination
128	Casini, B.; Righi, A.; De Feo, N.; Totaro, M.; Giorgi, S.; Zezza, L.; Valentini, P.; Tagliaferri, E.; Costa, A. L.; Barnini, S.; Baggiani, A.; Lopalco, P. L.; Malacarne, P.; Privitera, G. P.	2018	Improving Cleaning and Disinfection of High-Touch Surfaces in Intensive Care during Carbapenem-Resistant *Acinetobacter baumannii* Endemo-Epidemic Situations
129	Casini, B.; Selvi, C.; Cristina, M. L.; Totaro, M.; Costa, A. L.; Valentini, P.; Barnini, S.; Baggiani, A.; Tagliaferri, E.; Privitera, G.	2017	Evaluation of a modified cleaning procedure in the prevention of carbapenem-resistant *Acinetobacter baumannii* clonal spread in a burn intensive care unit using a high-sensitivity luminometer
130	Casini, B.; Tuvo, B.; Cristina, M. L.; Spagnolo, A. M.; Totaro, M.; Baggiani, A.; Privitera, G. P.	2019	Evaluation of an Ultraviolet C (UVC) Light-Emitting Device for Disinfection of High-touch Surfaces in Hospital Critical Areas
131	Casini, B.; Tuvo, B.; Totaro, M.; Aquino, F.; Baggiani, A.; Privitera, G.	2018	Evaluation of the Cleaning Procedure Efficacy in Prevention of Nosocomial Infections in Healthcare Facilities Using Cultural Method Associated with High Sensitivity Luminometer for ATP Detection
177	Chen, C. H.; Tu, C. C.; Kuo, H. Y.; Zeng, R. F.; Yu, C. S.; Lu, H. H.; Liou, M. L.	2017	Dynamic change of surface microbiota with different environmental cleaning methods between two wards in a hospital
280	Coppin, J. D.; Villamaria, F. C.; Williams, M. D.; Copeland, L. A.; Zeber, J. E.; Jinadatha, C.	2017	Self-sanitizing copper-impregnated surfaces for bioburden reduction in patient rooms
313	Danforth, D.; Nicolle, L. E.; Hume, K.; Alfieri, N.; Sims, H.	1987	Nosocomial infections on nursing units with floors cleaned with a disinfectant compared with detergent
346	de Jong, B.; Meeder, A. M.; Koekkoek, Kwac; Schouten, M. A.; Westers, P.; van Zanten, A. R. H.	2018	Pre-post evaluation of effects of a titanium dioxide coating on environmental contamination of an intensive care unit: the TITANIC study
393	Dharan, S.; Mourouga, P.; Copin, P.; Bessmer, G.; Tschanz, B.; Pittet, D.	1999	Routine disinfection of patients' environmental surfaces. Myth or reality?
414	Doan, L.; Forrest, H.; Fakis, A.; Craig, J.; Claxton, L.; Khare, M.	2012	Clinical and cost effectiveness of eight disinfection methods for terminal disinfection of hospital isolation rooms contaminated with *Clostridium difficile* 027
442	Dramowski, A.; Whitelaw, A.; Cotton, M. F.	2016	Assessment of terminal cleaning in pediatric isolation rooms: Options for low-resource settings
463	Dunklin, E. W.; Lester, W., Jr.	1959	Residual surface disinfection. II. The effect of orthophenylphenol treatment of the floor on bacterial contamination in a recovery room
484	Aimiya, K.; Sato, T.; Hishida, H.; Yamaguchi, K.	1989	Primary decontamination treatments and the control of microbial contamination in a new ward
516	Al-Hamad, A.; Maxwell, S.	2008	How clean is clean? Proposed methods for hospital cleaning assessment

521	Ali, S.; Muzslay, M.; Bruce, M.; Jeanes, A.; Moore, G.; Wilson, A. P. R.	2016	Efficacy of two hydrogen peroxide vapour aerial decontamination systems for enhanced disinfection of meticillin-resistant *Staphylococcus aureus*, *Klebsiella pneumoniae* and *Clostridium difficile* in single isolation rooms
536	Allen, O.; Jadkauskaite, L.; Shafi, N. T.; Jackson, A.; Athithan, V.; Chiu, Y. D.; Ies, E.; Floto, R. A.; Haworth, C. S.	2019	Microbiological evaluation of UV disinfection effectiveness in a specialist cystic fibrosis clinic
567	Andersen, B. M.; Banrud, H.; Boe, E.; Bjordal, O.; Drangsholt, F.	2006	Comparison of UV C light and chemicals for disinfection of surfaces in hospital isolation units
568	Andersen, B. M.; Rasch, M.; Kvist, J.; Tollefsen, T.; Lukkassen, R.; Sandvik, L.; Welo, A.	2009	Floor cleaning: effect on bacteria and organic materials in hospital rooms
606	Armellino, D.; Goldstein, K.; Thomas, L.; Walsh, T. J.; Petraitis, V.	2020	Comparative evaluation of operating room terminal cleaning by two methods: Focused multivector ultraviolet (FMUV) versus manual-chemical disinfection
621	Attaway, H. H., 3rd; Fairey, S.; Steed, L. L.; Salgado, C. D.; Michels, H. T.; Schmidt, M. G.	2012	Intrinsic bacterial burden associated with intensive care unit hospital beds: effects of disinfection on population recovery and mitigation of potential infection risk
686	Barbut, F.; Menuet, D.; Verachten, M.; Girou, E.	2009	Comparison of the efficacy of a hydrogen peroxide dry-mist disinfection system and sodium hypochlorite solution for eradication of *Clostridium difficile* spores
766	Blazejewski, C.; Wallet, F.; Rouze, A.; Le Guern, R.; Ponthieux, S.; Salleron, J.; Nseir, S.	2015	Efficiency of hydrogen peroxide in improving disinfection of ICU rooms
808	Boyce, J. M.; Guercia, K. A.; Sullivan, L.; Havill, N. L.; Fekieta, R.; Kozakiewicz, J.; Goffman, D.	2017	Prospective cluster controlled crossover trial to compare the impact of an improved hydrogen peroxide disinfectant and a quaternary ammonium-based disinfectant on surface contamination and health care outcomes
809	Boyce, J. M.; Havill, N. L.	2013	Evaluation of a new hydrogen peroxide wipe disinfectant
810	Boyce, J. M.; Havill, N. L.; Guercia, K. A.; Schweon, S. J.; Moore, B. A.	2014	Evaluation of two organosilane products for sustained antimicrobial activity on high-touch surfaces in patient rooms
813	Boyce, J. M.; Havill, N. L.; Otter, J. A.; McDonald, L. C.; Adams, N. M.; Cooper, T.; Thompson, A.; Wiggs, L.; Killgore, G.; Tauman, A.; Noble-Wang, J.	2008	Impact of hydrogen peroxide vapor room decontamination on *Clostridium difficile* environmental contamination and transmission in a healthcare setting
882	Byers, K. E.; Durbin, L. J.; Simonton, B. M.; Anglim, A. M.; Adal, K. A.; Farr, B. M.	1998	Disinfection of hospital rooms contaminated with vancomycin-resistant *Enterococcus* faecium
899	Eckstein, B. C.; Adams, D. A.; Eckstein, E. C.; Rao, A.; Sethi, A. K.; Yadavalli, G. K.; Donskey, C. J.	2007	Reduction of *Clostridium difficile* and vancomycin-resistant *Enterococcus* contamination of environmental surfaces after an intervention to improve cleaning methods
904	Edmiston, C. E., Jr.; Spencer, M.; Lewis, B. D.; Rossi, P. J.; Brown, K. R.; Malinowski, M.; Seabrook, G. R.; Leaper, D.	2020	Assessment of a novel antimicrobial surface disinfectant on inert surfaces in the intensive care unit environment using ATP-bioluminesence assay

975	Fattorini, M.; Buonocore, G.; Lenzi, D.; Burgassi, S.; Cardaci, R. M. R.; Biermann, K. P.; Cevenini, G.; Messina, G.	2018	Public Health since the beginning: Neonatal incubators safety in a clinical setting
997	Ferreira, A. M.; de Andrade, D.; Rigotti, M. A.; de Almeida, M. T.; Guerra, O. G.; dos Santos Junior, A. G.	2015	Assessment of disinfection of hospital surfaces using different monitoring methods
1011	Fitton, K.; Barber, K. R.; Karamon, A.; Zuehlke, N.; Atwell, S.; Enright, S.	2017	Long-acting water-stable organosilane agent and its sustained effect on reducing microbial load in an intensive care unit
1024	Frabetti, A.; Vandini, A.; Balboni, P.; Triolo, F.; Mazzacane, S.	2009	Experimental evaluation of the efficacy of sanitation procedures in operating rooms
1045	Frota, O. P.; Ferreira, A. M.; Guerra, O. G.; Rigotti, M. A.; de Andrade, D.; Borges, N. M. A.; de Almeida, M. T. G.	2017	Efficiency of cleaning and disinfection of surfaces: correlation between assessment methods
1059	Fukada, T.; Tsuchiya, Y.; Iwakiri, H.; Ozaki, M.	2015	Adenosine triphosphate bioluminescence assay for monitoring contamination of the working environment of anaesthetists and cleanliness of the operating room
1065	Furlan, M. C. R.; Ferreira, A. M.; Rigotti, M. A.; Guerra, O. G.; Frota, O. P.; De Sousa, A. F. L.; De Andrade, D.	2019	Correlation among monitoring methods of surface cleaning and disinfection in outpatient facilities
1073	Gable, T. S.	1966	Bactericidal effectiveness of floor cleaning methods in a hospital environment
1079	Galván Contreras, R.; Ruiz Tapia, R. A.; Segura Cervantes, E.; Cortés Aguilar, R. M. A.	2016	Comparative study on the effectiveness of 6% sodium hypochlorite solution vs a bromine-chloro-dimethylhydantoin solution for disinfecting hospital environments
1081	Gan, T.; Xu, H.; Wu, J.; Zhu, Y.; Wang, L.; Jin, H.; Wei, L.; Shen, L.; Ni, X.; Cao, J.; Zhang, Y.	2017	Sequential enhanced cleaning eliminates multidrug-resistant organisms in general intensive care unit of a traditional Chinese medicine hospital
1096	Garvey, M. I.; Bradley, C. W.; Jumaa, P.	2016	Environmental decontamination following occupancy of a burns patient with multiple carbapenemase-producing organisms
1105	Gelmini, F.; Belotti, L.; Vecchi, S.; Testa, C.; Beretta, G.	2016	Air dispersed essential oils combined with standard sanitization procedures for environmental microbiota control in nosocomial hospitalization rooms
1171	Ghantoji, S. S.; Stibich, M.; Stachowiak, J.; Cantu, S.; Adachi, J. A.; Raad, I. I.; Chemaly, R. F.	2015	Non-inferiority of pulsed xenon UV light versus bleach for reducing environmental *Clostridium difficile* contamination on high-touch surfaces in *Clostridium difficile* infection isolation rooms
1205	Hedin, G.; Rynback, J.; Lore, B.	2010	Reduction of bacterial surface contamination in the hospital environment by application of a new product with persistent effect
1245	Hinsa-Leasure, S. M.; Nartey, Q.; Vaverka, J.; Schmidt, M. G.	2016	Copper alloy surfaces sustain terminal cleaning levels in a rural hospital
1268	Holmdahl, T.; Walder, M.; Uzcategui, N.; Odenholt, I.; Lanbeck, P.; Medstrand, P.; Widell, A.	2016	Hydrogen Peroxide Vapor Decontamination in a Patient Room Using Feline Calicivirus and Murine Norovirus as Surrogate Markers for Human Norovirus
1280	Hosein, I.; Madeloso, R.; Nagaratnam, W.; Villamaria, F.; Stock, E.; Jinadatha, C.	2016	Evaluation of a pulsed xenon ultraviolet light device for isolation room disinfection in a United Kingdom hospital

1311	Huang, Y. S.; Chen, Y. C.; Chen, M. L.; Cheng, A.; Hung, I. C.; Wang, J. T.; Sheng, W. H.; Chang, S. C.	2015	Comparing visual inspection, aerobic colony counts, and adenosine triphosphate bioluminescence assay for evaluating surface cleanliness at a medical center
1316	Humayun, T.; Qureshi, A.; Al Roweily, S. F.; Carig, J.; Humayun, F.	2019	Efficacy Of Hydrogen Peroxide Fumigation In Improving Disinfection Of Hospital Rooms And Reducing The Number Of Microorganisms
1416	Jinadatha, C.; Quezada, R.; Huber, T. W.; Williams, J. B.; Zeber, J. E.; Copeland, L. A.	2014	Evaluation of a pulsed-xenon ultraviolet room disinfection device for impact on contamination levels of methicillin-resistant *Staphylococcus aureus*
1548	Lee, W. S.; Hsieh, T. C.; Shiau, J. C.; Ou, T. Y.; Chen, F. L.; Liu, Y. H.; Yen, M. Y.; Hsueh, P. R.	2017	Bio-Kil, a nano-based disinfectant, reduces environmental bacterial burden and multidrug-resistant organisms in intensive care units
1574	Lerner, A. O.; Abu-Hanna, J.; Carmeli, Y.; Schechner, V.	2019	Environmental contamination by carbapenem-resistant *Acinetobacter baumannii*: The effects of room type and cleaning methods
1585	Lewis, B. D.; Spencer, M.; Rossi, P. J.; Lee, C. J.; Brown, K. R.; Malinowski, M.; Seabrook, G. R.; Edmiston, C. E., Jr.	2015	Assessment of an innovative antimicrobial surface disinfectant in the operating room environment using adenosine triphosphate bioluminescence assay
1626	Gonzalez, S.; Illescas, A.; Escarzaga, E.	1963	[REDUCTION OF BACTERIAL CONTAMINATION OF THE ENVIRONMENT IN A GENERAL HOSPITAL, BY THE USE OF A NEW GERMICIDE: BIOMET 66]
1723	Hall, T. J.; Jeanes, A.; McKain, L. W.; Jepson, M. J.; Coen, P. G.; Hickok, S. S.; Gant, V. A.	2011	A UK district general hospital cleaning study: A comparison of the performance of ultramicrofibre technology with or without addition of a novel copper-based biocide with standard hypochlorite-based cleaning
1886	Mosci, D.; Marmo, G. W.; Sciolino, L.; Zaccaro, C.; Antonellini, R.; Accogli, L.; Lazzarotto, T.; Mongardi, M.; Landini, M. P.	2017	Automatic environmental disinfection with hydrogen peroxide and silver ions versus manual environmental disinfection with sodium hypochlorite: a multicentre randomized before-and-after trial
1979	Johnson, A.; Weston, L.; Grisewood, L.; Kyffin, M.	2016	Evaluation of the Ultra-V (ultraviolet) decontamination system as an adjunct to cleaning in a district general hospital
1991	Jones, R.; Hutton, A.; Mariyaselvam, M.; Hodges, E.; Wong, K.; Blunt, M.; Young, P.	2015	Keyboard cleanliness: a controlled study of the residual effect of chlorhexidine gluconate
2035	Karunanayake, L. I.; Waniganayake, Y. C.; Gunawardena, K. D. N.; Padmaraja, S. A. D.; Peter, D.; Jayasekera, R.; Karunanayake, P.	2019	Use of silicon nanoparticle surface coating in infection control: Experience in a tropical healthcare setting
2228	Ojajärvi &, J.; Mäkelä, P.	1976	Evaluation of Chlorine Compounds for Surface Disinfection by Laboratory and ln-use Testing
2254	Oon, A.; Reading, E.; Ferguson, J. K.; Dancer, S. J.; Mitchell, B. G.	2020	Measuring environmental contamination in critical care using dilute hydrogen peroxide (DHP) technology: An observational cross-over study
2261	Ortí-Lucas, R. M.; Muñoz-Miguel, J.	2017	Effectiveness of surface coatings containing silver ions in bacterial decontamination in a recovery unit
2287	Özpolat, B.; Çavuşoğlu, T.; Yilmaz, S.; Büyükkoçak, Ü.;	2011	Clinical and laboratory evaluation of anti-microbial efficacy of photocatalysts

	Günaydin, S.		
2288	Oztoprak, N.; Kizilates, F.; Percin, D.	2019	Comparison of steam technology and a two-step cleaning (water/detergent) and disinfecting (1,000 resp. 5,000 ppm hypochlorite) method using microfiber cloth for environmental control of multidrug-resistant organisms in an intensive care unit
2322	Passaretti, C. L.; Otter, J. A.; Reich, N. G.; Myers, J.; Shepard, J.; Ross, T.; Carroll, K. C.; Lipsett, P.; Perl, T. M.	2013	An Evaluation of Environmental Decontamination With Hydrogen Peroxide Vapor for Reducing the Risk of Patient Acquisition of Multidrug-Resistant Organisms
2323	Patel, S. S.; Pevalin, D. J.; Prosser, R.; Couchman, A.	2007	Comparison of detergent-based cleaning, disinfectant-based cleaning, and detergent-based cleaning after enhanced domestic staff training within a source isolation facility
2427	Lowe, J. J.; Gibbs, S. G.; Iwen, P. C.; Smith, P. W.; Hewlett, A. L.	2013	Impact of chlorine dioxide gas sterilization on nosocomial organism viability in a hospital room
2592	Popov, D. A.; Anuchina, N. M.	2016	Microbiological Efficacy of Hospital Environment Decontamination by Hydrogen Peroxide Aerosol
2616	Prindis, V.; Michalek, J.; Kubatova, I.	2018	Application of photocatalytic nanolayers SmartCoat in health care
2653	Schmidt, M. G.; Attaway, H. H.; Fairey, S. E.; Howard, J.; Mohr, D.; Craig, S.	2019	Self-Disinfecting Copper Beds Sustain Terminal Cleaning and Disinfection Effects throughout Patient Care
2654	Schmidt, M. G.; Attaway, H. H.; Sharpe, P. A.; John, J., Jr.; Sepkowitz, K. A.; Morgan, A.; Fairey, S. E.; Singh, S.; Steed, L. L.; Cantey, J. R.; Freeman, K. D.; Michels, H. T.; Salgado, C. D.	2012	Sustained reduction of microbial burden on common hospital surfaces through introduction of copper
2655	Schmidt, M. G.; Fairey, S. E.; Attaway, H. H.	2019	In situ evaluation of a persistent disinfectant provides continuous decontamination within the clinical environment
2656	Schmidt, M. G.; von Dessauer, B.; Benavente, C.; Benadof, D.; Cifuentes, P.; Elgueta, A.; Duran, C.; Navarrete, M. S.	2016	Copper surfaces are associated with significantly lower concentrations of bacteria on selected surfaces within a pediatric intensive care unit
2707	Sexton, J. D.; Tanner, B. D.; Maxwell, S. L.; Gerba, C. P.	2011	Reduction in the microbial load on high-touch surfaces in hospital rooms by treatment with a portable saturated steam vapor disinfection system
2730	Shapey, S.; Machin, K.; Levi, K.; Boswell, T. C.	2008	Activity of a dry mist hydrogen peroxide system against environmental *Clostridium difficile* contamination in elderly care wards
2745	Shekhawat, P. S.; Singh, R. N.; Shekhawat, R.; Joshi, K. R.	1992	Fumigation of neonatal nursery: how effective in reducing the environmental pathogens?
2781	Sigler, V.; Hensley, S.	2013	Persistence of mixed staphylococci assemblages following disinfection of hospital room surfaces
2864	Smith, T. L.; Iwen, P. C.; Olson, S. B.; Rupp, M. E.	1998	Environmental contamination with vancomycin-resistant enterococci in an outpatient setting
2906	Stibich, M.; Stachowiak, J.; Tanner, B.; Berkheiser, M.; Moore, L.; Raad, I.; Chemaly, R. F.	2011	Evaluation of a pulsed-xenon ultraviolet room disinfection device for impact on hospital operations and microbial reduction
2922	Strassle, P.; Thom, K. A.; Johnson, J. K.; Leekha, S.; Lissauer, M.; Zhu, J.; Harris, A. D.	2012	The effect of terminal cleaning on environmental contamination rates of multidrug-resistant *Acinetobacter baumannii*

2926	Styaningsih, N.; Suwondo, A.; Adi, M. S.	2019	Effectiveness of disinfectant a and b on the growth of bacteria in the area of central surgical installation of hospital x in kudus city
2944	Suzuki, A.; Namba, Y.; Matsuura, M.; Horisawa, A.	1984	Bacterial contamination of floors and other surfaces in operating rooms: a five-year survey
3071	Reid, M.; Whatley, V.; Spooner, E.; Nevill, A. M.; Cooper, M.; Ramsden, J. J.; Dancer, S. J.	2018	How Does a Photocatalytic Antimicrobial Coating Affect Environmental Bioburden in Hospitals?
3084	Reynolds, K. A.; Sexton, J. D.; Pivo, T.; Humphrey, K.; Leslie, R. A.; Gerba, C. P.	2019	Microbial transmission in an outpatient clinic and impact of an intervention with an ethanol-based disinfectant
3147	Roux, D.; Aubier, B.; Cochard, H.; Quentin, R.; van der Mee-Marquet, N.	2013	Contaminated sinks in intensive care units: an underestimated source of extended-spectrum beta-lactamase-producing *Enterobacteriaceae* in the patient environment
3190	Saha, A.; Botha, S. L.; Weaving, P.; Satta, G.	2016	A pilot study to assess the effectiveness and cost of routine universal use of peracetic acid sporicidal wipes in a real clinical environment
3236	Santos-Junior, A. G.; Ferreira, A. M.; Frota, O. P.; Rigotti, M. A.; Barcelos, L. D. S.; Lopes de Sousa, A. F.; de Andrade, D.; Guerra, O. G.; Mc, R. Furlan	2018	Effectiveness of Surface Cleaning and Disinfection in a Brazilian Healthcare Facility
3432	Youkee, D.; Brown, C. S.; Lilburn, P.; Shetty, N.; Brooks, T.; Simpson, A.; Bentley, N.; Lado, M.; Kamara, T. B.; Walker, N. F.; Johnson, O.	2015	Assessment of Environmental Contamination and Environmental Decontamination Practices within an Ebola Holding Unit, Freetown, Sierra Leone
3444	Yui, S.; Ali, S.; Muzslay, M.; Jeanes, A.; Wilson, A. P. R.	2017	Identification of *Clostridium difficile* Reservoirs in The Patient Environment and Efficacy of Aerial Hydrogen Peroxide Decontamination
3507	Rathod, S. N.; Beauvais, K.; Sullivan, L. K.; Sudikoff, S. N.; Peaper, D. R.; Martinello, R. A.	2019	The effectiveness of a novel colorant additive in the daily cleaning of patient rooms
3614	Vesley, D.; Klapes, N. A.; Benzow, K.; Le, C. T.	1987	Microbiological evaluation of wet and dry floor sanitization systems in hospital patient rooms
3666	Zhang, A.; Nerandzic, M. M.; Kundrapu, S.; Donskey, C. J.	2013	Does organic material on hospital surfaces reduce the effectiveness of hypochlorite and UV radiation for disinfection of *Clostridium difficile*?
3699	Zubair, M.; Imtiaz, S.; Zafar, A.; Javed, H.; Atif, M.; Abosalif, K. O. A. A.; Ejaz, H.	2018	Role of hospital surfaces in transmission of infectious diseases
3854	Wilcox, M. H.; Fawley, W. N.; Wigglesworth, N.; Parnell, P.; Verity, P.; Freeman, J.	2003	Comparison of the effect of detergent versus hypochlorite cleaning on environmental contamination and incidence of *Clostridium difficile* infection
3858	Wiemken, T. L.; Curran, D. R.; Kelley, R. R.; Pacholski, E. B.; Carrico, R. M.; Peyrani, P.; Khan, M. S.; Ramirez, J. A.	2014	Evaluation of the effectiveness of improved hydrogen peroxide in the operating room
4060	Turner, A. G.; Higgins, M. M.; Craddock, J. G.	1974	Disinfection of immersion tanks (Hubbard) in a hospital burn unit
4132	Thom, K. A.; Standiford, H. C.; Johnson, J. K.; Hanna, N.; Furuno, J. P.	2014	Effectiveness of an antimicrobial polymer to decrease contamination of environmental surfaces in the clinical setting

4146	Tekin, A.; Dal, T.; Selcuk, C. T.; Deveci, Ö; Tekin, R.; Mete, M.; Dayan, S.; Hosoglu, S.	2013	Orthophenylphenol in healthcare environments: A trial related to a new administration method and a review of the literature
4152	Taylor, L.; Phillips, P.; Hastings, R.	2009	Reduction of bacterial contamination in a healthcare environment by silver antimicrobial technology
4220	Sui, Y. S.; Wan, G. H.; Chen, Y. W.; Ku, H. L.; Li, L. P.; Liu, C. H.; Mau, H. S.	2012	Effectiveness of bacterial disinfectants on surfaces of mechanical ventilator systems
4505	Sjoberg, M.; Eriksson, M.; Andersson, J.; Noren, T.	2014	Transmission of *Clostridium difficile* spores in isolation room environments and through hospital beds
4519	Singh, H.; Kumar, R.; Singh, K.; Attri, J.	2017	INFECTION CONTROL IN ISOLATION UNITS/HDUS/ICUS- A COMPARATIVE STUDY USING THREE DIFFERENT DISINFECTANTS WITH FOGGER FOR ENVIRONMENTAL DECONTAMINATION
4540	Siani, H.; Wesgate, R.; Maillard, J. Y.	2018	Impact of antimicrobial wipes compared with hypochlorite solution on environmental surface contamination in a health care setting: A double-crossover study
4655	Schmidt, M. G.; Attaway Iii, H. H.; Fairey, S. E.; Steed, L. L.; Michels, H. T.; Salgado, C. D.	2013	Copper continuously limits the concentration of bacteria resident on bed rails within the intensive care unit
4733	Munster, A. M.; Ostrander, W. E.	1974	Terminal disinfection of contaminated patient care areas: to fog or not to fog?
4861	McCord, J.; Prewitt, M.; Dyakova, E.; Mookerjee, S.; Otter, J. A.	2016	Reduction in *Clostridium difficile* infection associated with the introduction of hydrogen peroxide vapour automated room disinfection
4960	Panknin, H. T.	2014	Diversity of the ambient flora and effectiveness of surface disinfection measures in the neonatal unit
4992	Otter, J. A.; Mepham, S.; Athan, B.; Mack, D.; Smith, R.; Jacobs, M.; Hopkins, S.	2016	Terminal decontamination of the Royal Free London's high-level isolation unit after a case of Ebola virus disease using hydrogen peroxide vapor
5106	Marais, F.; Mehtar, S.; Chalkley, L.	2010	Antimicrobial efficacy of copper touch surfaces in reducing environmental bioburden in a South African community healthcare facility
5113	Manian, F. A.; Griesnauer, S.; Bryant, A.	2013	Implementation of hospital-wide enhanced terminal cleaning of targeted patient rooms and its impact on endemic *Clostridium difficile* infection rates
5183	Lowe, J. J.; Gibbs, S. G.; Iwen, P. C.; Smith, P. W.; Hewlett, A. L.	2013	Decontamination of a hospital room using gaseous chlorine dioxide: Bacillus anthracis, Francisella tularensis, and Yersinia pestis
5485	Le Coutour, X.; Oblin, I.	1991	Disinfection of surfaces in hospital: Comparison between theoric and real efficiency of three comercial products
5623	Kitagawa, H.; Mori, M.; Kashiyama, S.; Sasabe, Y.; Ukon, K.; Shimokawa, N.; Shime, N.; Ohge, H.	2020	Effect of pulsed xenon ultraviolet disinfection on methicillin-resistant *Staphylococcus aureus* contamination of high-touch surfaces in a Japanese hospital
5698	Strat, E.	1971	[Research on the efficiency of disinfectants of the tensio-active group in sterilization of the hospital environment]

5792	Havill, N. L.; Moore, B. A.; Boyce, J. M.	2012	Comparison of the microbiological efficacy of hydrogen peroxide vapor and ultraviolet light processes for room decontamination
5832	Hamilton, D.; Foster, A.; Ballantyne, L.; Kingsmore, P.; Bedwell, D.; Hall, T. J.; Hickok, S. S.; Jeanes, A.; Coen, P. G.; Gant, V. A.	2010	Performance of ultramicrofibre cleaning technology with or without addition of a novel copper-based biocide
5852	Hacek, D. M.; Ogle, A. M.; Fisher, A.; Robicsek, A.; Peterson, L. R.	2010	Significant impact of terminal room cleaning with bleach on reducing nosocomial *Clostridium difficile*
5957	Goldenberg, S. D.; Patel, A.; Tucker, D.; French, G. L.	2012	Lack of enhanced effect of a chlorine dioxide-based cleaning regimen on environmental contamination with *Clostridium difficile* spores
6163	Ho, Y. H.; Wang, L. S.; Jiang, H. L.; Chang, C. H.; Hsieh, C. J.; Chang, D. C.; Tu, H. Y.; Chiu, T. Y.; Chao, H. J.; Tseng, C. C.	2016	Use of a Sampling Area-Adjusted Adenosine Triphosphate Bioluminescence Assay Based on Digital Image Quantification to Assess the Cleanliness of Hospital Surfaces
6199	Chan, H. T.; White, P.; Sheorey, H.; Cocks, J.; Waters, M. J.	2011	Evaluation of the biological efficacy of hydrogen peroxide vapour decontamination in wards of an Australian hospital
6269	Čamdžić, A.; Dedeić-Ljubović, A.; Madacki-Todorović, K.	2019	Using desinfection devices in intensive care units
6287	Butin, M.; Dumont, Y.; Monteix, A.; Raphard, A.; Roques, C.; Martins Simoes, P.; Picaud, J. C.; Laurent, F.	2019	Sources and reservoirs of *Staphylococcus* capitis NRCS-A inside a NICU
6368	Bokulich, N. A.; Mills, D. A.; Underwood, M. A.	2013	Surface microbes in the neonatal intensive care unit: changes with routine cleaning and over time
6414	Karpanen, T. J.; Casey, A. L.; Lambert, P. A.; Cookson, B. D.; Nightingale, P.; Miruszenko, L.; Elliott, T. S. J.	2012	The Antimicrobial Efficacy of Copper Alloy Furnishing in the Clinical Environment: A crossover study
6482	Garvey, M. I.; Wilkinson, M. A. C.; Bradley, C. W.; Holden, K. L.; Holden, E.	2018	Wiping out MRSA: effect of introducing a universal disinfection wipe in a large UK teaching hospital
6651	Daschner, F.; Rabbenstein, G.; Langmaack, H.	1980	Surface decontamination in the control of hospital infections: comparison of different methods (author's transl)
6885	Anderson, D. J.; Moehring, R. W.; Weber, D. J.; Lewis, S. S.; Chen, L. F.; Schwab, J. C.; Becherer, P.; Blocker, M.; Triplett, P. F.; Knelson, L. P.; Lokhnygina, Y.; Rutala, W. A.; Sexton, D. J.	2018	Effectiveness of targeted enhanced terminal room disinfection on hospital-wide acquisition and infection with multidrug-resistant organisms and *Clostridium difficile*: a secondary analysis of a multicentre cluster randomised controlled trial with crossover
6887	Anderson, D. J.; Chen, L. F.; Weber, D. J.; Moehring, R. W.; Lewis, S. S.; Triplett, P. F.; Blocker, M.; Becherer, P.; Schwab, J. C.; Knelson, L. P.; Lokhnygina, Y.; Rutala, W. A.; Kanamori, H.; Gergen, M. F.; Sexton, D. J.	2017	Enhanced terminal room disinfection and acquisition and infection caused by multidrug-resistant organisms and *Clostridium difficile* (the Benefits of Enhanced Terminal Room Disinfection study): a cluster-randomised, multicentre, crossover study
6888	Andersen, B. M.; Rasch, M.; Hochlin, K.; Jensen, F. H.; Wismar, P.; Fredriksen, J. E.	2006	Decontamination of rooms, medical equipment and ambulances using an aerosol of hydrogen peroxide disinfectant
6931	Alhmidi, H.; Koganti, S.; Cadnum, J. L.; Rai, H.; Jencson, A. L.; Donskey, C. J.	2017	Evaluation of a Novel Alcohol-Based Surface Disinfectant for Disinfection of Hard and Soft Surfaces in Healthcare Facilities

6936	Alekseeva, M. I.; Tsetlin, V. M.; Savel'eva, A. R.; Mal'kov, O. S.; Zakomyrdin, A. A.; Fediaev, B. P.; Iarnykh, V. S.; Bochenin Iu, I.; Chkoniia, T. T.	1969	[Disinfection in antituberculous institutions]
7047	Deshpande, A.; Mana, T. S. C.; Cadnum, J. L.; Jencson, A. C.; Sitzlar, B.; Fertelli, D.; Hurless, K.; Kundrapu, S.; Sunkesula, V. C. K.; Donskey, C. J.	2014	Evaluation of a sporicidal peracetic acid/hydrogen peroxideâ€“based daily disinfectant cleaner
7122	Best, E. L.; Parnell, P.; Thirkell, G.; Verity, P.; Copland, M.; Else, P.; Denton, M.; Hobson, R. P.; Wilcox, M. H.	2014	Effectiveness of deep cleaning followed by hydrogen peroxide decontamination during high *Clostridium difficile* infection incidence
7455	Exner, M.; Vogel, F.; Hamann, R.	1982	Surface disinfection in a medical intensive care unit
7468	Esolen, L. M.; Thakur, L.; Layon, A. J.; Fuller, T. A.; Harrington, D. J.; Jha, K.; Kariyawasam, S.	2018	The efficacy of self-disinfecting bedrail covers in an intensive care unit
7829	Taneja, N.; Biswal, M.; Kumar, A.; Edwin, A.; Sunita, T.; Emmanuel, R.; Gupta, A. K.; Sharma, M.	2011	Hydrogen peroxide vapour for decontaminating air-conditioning ducts and rooms of an emergency complex in northern India: time to move on
7891	Stewart, M.; Bogusz, A.; Hunter, J.; Devanny, I.; Yip, B.; Reid, D.; Robertson, C.; Dancer, S. J.	2014	Evaluating use of neutral electrolyzed water for cleaning near-patient surfaces
7928	Souli, M.; Antoniadou, A.; Katsarolis, I.; Mavrou, I.; Paramythiotou, E.; Papadomichelakis, E.; Drogari-Apiranthitou, M.; Panagea, T.; Giamarellou, H.; Petrikkos, G.; Armaganidis, A.	2017	Reduction of Environmental Contamination With Multidrug-Resistant Bacteria by Copper-Alloy Coating of Surfaces in a Highly Endemic Setting
7960	Simon Garcia, M. J.; Gonzalez Sanchez, J. A.; Alcudia Perez, F.; Sanchez Sanchez, C.; Gomez Mayoral, B.; Merino Martinez, M. R.	2009	Evaluation of the effect of a cleaning/disinfection intervention on the rate of multiresistant microorganism infections in the Intensive Care Unit
7971	Sifri, C. D.; Burke, G. H.; Enfield, K. B.	2016	Reduced health care-associated infections in an acute care community hospital using a combination of self-disinfecting copper-impregnated composite hard surfaces and linens
8042	Orenstein, R.; Aronhalt, K. C.; McManus, J. E., Jr.; Fedraw, L. A.	2011	A targeted strategy to wipe out *Clostridium difficile*
8147	Nakata, S.; Ikeda, T.; Nakatani, H.; Sakamoto, M.; Higashidutsumi, M.; Honda, T.; Kawayoshi, A.; Iwamura, Y.	2001	Evaluation of an automatic fogging disinfection unit
8154	Nagai, I.; Kadota, M.; Matsuoka, K.; Jitsukawa, S.	1983	Evaluation of chemical aerosol spray disinfection in the operating room
8312	Rai, S.; Hirsch, B. E.; Attaway, H. H.; Nadan, R.; Fairey, S.; Hardy, J.; Miller, G.; Armellino, D.; Moran, W. R.; Sharpe, P.; Estelle, A.; Michel, J. H.; Michels, H. T.; Schmidt, M. G.	2012	Evaluation of the antimicrobial properties of copper surfaces in an outpatient infectious disease practice
8380	Mayfield, J. L.; Leet, T.; Miller, J.; Mundy, L. M.	2000	Environmental control to reduce transmission of *Clostridium difficile*
8687	Meinke, R.; Meyer, B.; Frei, R.; Passweg, J.; Widmer, A. F.	2012	Equal efficacy of glucoprotamin and an aldehyde product for environmental disinfection in a

			hematologic transplant unit: a prospective crossover trial
8890	Inkinen, J.; Mäkinen, R.; Keinänen-Toivola, M. M.; Nordström, K.; Ahonen, M.	2017	Copper as an antibacterial material in different facilities
9254	Fukada, T.; Iwakiri, H.; Ozaki, M.	2008	Anaesthetists' role in computer keyboard contamination in an operating room
9347	Evans, M. W.; Breshears, J.; Campbell, A.; Husbands, C.; Rupert, R.	2007	Assessment and risk reduction of infectious pathogens on chiropractic treatment tables
9616	Barbeito, M. S.	1966	Emergency disinfection of operating room and patient ward with beta-propiolactone
9651	Dyas, A.; Boughton, B. J.; Das, B. C.	1983	Ozone killing action against bacterial and fungal species; microbiological testing of a domestic ozone generator
9825	Codish, S.; Toledano, R.; Novack, V.; Sherf, M.; Borer, A.	2015	Effectiveness of stringent decontamination of computer input devices in the era of electronic medical records and bedside computing: a randomized controlled trial
10160	Yuen, J. W.; Chung, T. W.; Loke, A. Y.	2015	Methicillin-resistant *Staphylococcus aureus* (MRSA) contamination in bedside surfaces of a hospital ward and the potential effectiveness of enhanced disinfection with an antimicrobial polymer surfactant
10314	von Dessauer, B.; Navarrete, M. S.; Benadof, D.; Benavente, C.; Schmidt, M. G.	2016	Potential effectiveness of copper surfaces in reducing health careâ€“associated infection rates in a pediatric intensive and intermediate care unit: A nonrandomized controlled trial
10463	Shelly, M. J.; Scanlon, T. G.; Ruddy, R.; Hannan, M. M.; Murray, J. G.	2011	Meticillin-resistant *Staphylococcus aureus* (MRSA) environmental contamination in a radiology department
10553	Rutala, W. A.; Kanamori, H.; Gergen, M. F.; Knelson, L. P.; Sickbert-Bennett, E. E.; Chen, L. F.; Anderson, D. J.; Sexton, D. J.; Weber, D. J.	2018	Enhanced disinfection leads to reduction of microbial contamination and a decrease in patient colonization and infection
10625	Ray, A.; Perez, F.; Beltramini, A. M.; Jakubowycz, M.; Dimick, P.; Jacobs, M. R.; Roman, K.; Bonomo, R. A.; Salata, R. A.	2010	Use of vaporized hydrogen peroxide decontamination during an outbreak of multidrug-resistant *Acinetobacter baumannii* infection at a long-term acute care hospital
10851	Tamimi, A. H.; Carlino, S.; Gerba, C. P.	2014	Long-term efficacy of a self-disinfecting coating in an intensive care unit
10984	Otter, J. A.; Yezli, S.; Schouten, M. A.; van Zanten, A. R.; Houmes-Zielman, G.; Nohlmans-Paulssen, M. K.	2010	Hydrogen peroxide vapor decontamination of an intensive care unit to remove environmental reservoirs of multidrug-resistant gram-negative rods during an outbreak
10993	Ory, J.; Cazaban, M.; Richaud-Morel, B.; Di Maio, M.; Dunyach-Remy, C.; Pantel, A.; Sotto, A.; Laurent, F.; Lavigne, J. P.; Butin, M.	2019	Successful implementation of infection control measure in a neonatal intensive care unit to combat the spread of pathogenic multidrug resistant *Staphylococcus* capitis
11015	Oie, S.; Yanagi, C.; Matsui, H.; Nishida, T.; Tomita, M.; Kamiya, A.	2005	Contamination of environmental surfaces by *Staphylococcus aureus* in a dermatological ward and its preventive measures
11022	Ogino, J.; Fujimori, I.; Goto, R.; Hisamastu, K.; Murakami, Y.; Yamada, T.; Kikushima, K.	1995	Efficacy of pyoktanin and DF-100 for prevention of nosocomial MRSA infection

11135	Salgado, C. D.; Sepkowitz, K. A.; John, J. F.; Cantey, J. R.; Attaway, H. H.; Freeman, K. D.; Sharpe, P. A.; Michels, H. T.; Schmidt, M. G.	2013	Copper surfaces reduce the rate of healthcare-acquired infections in the intensive care unit
11965	Fujii, M.; Yasuhara, S.; Ohmoto, Y.; Sugiyama, S.; Nagatsugu, Y.; Katoh, S.; Yamashita, T.; Ito, H.; Oie, S.; Kamiya, A.	1996	[Prevention of MRSA spread in the neurosurgical field]
12022	Kim, M. H.; Lee, S. G.; Kim, K. S.; Heo, Y. J.; Oh, J. E.; Jeong, S. J.	2018	Environmental disinfection with photocatalyst as an adjunctive measure to control transmission of methicillin-resistant *Staphylococcus aureus*: a prospective cohort study in a high-incidence setting
12244	Doidge, M.; Allworth, A. M.; Woods, M.; Marshall, P.; Terry, M.; O'Brien, K.; Goh, H. M.; George, N.; Nimmo, G. R.; Schembri, M. A.; Lipman, J.; Paterson, D. L.	2010	Control of an outbreak of carbapenem-resistant *Acinetobacter baumannii* in Australia after introduction of environmental cleaning with a commercial oxidizing disinfectant
12491	Barbut, F.; Yezli, S.; Mimoun, M.; Pham, J.; Chaouat, M.; Otter, J. A.	2013	Reducing the spread of *Acinetobacter baumannii* and methicillin-resistant *Staphylococcus aureus* on a burns unit through the intervention of an infection control bundle
12894	Biswal, M.; Rudramurthy, S. M.; Jain, N.; Shamanth, A. S.; Sharma, D.; Jain, K.; Yaddanapudi, L. N.; Chakrabarti, A.	2017	Controlling a possible outbreak of *Candida auris* infection: lessons learnt from multiple interventions
12952	Bates, C. J.; Pearse, R.	2005	Use of hydrogen peroxide vapour for environmental control during a Serratia outbreak in a neonatal intensive care unit
13449	Otter, J. A.; Cummins, M.; Ahmad, F.; van Tonder, C.; Drabu, Y. J.	2007	Assessing the biological efficacy and rate of recontamination following hydrogen peroxide vapour decontamination
13703	Montero, D. A.; Arellano, C.; Pardo, M.; Vera, R.; Gálvez, R.; Cifuentes, M.; Berasain, M. A.; Gómez, M.; Ramírez, C.; Vidal, R. M.	2019	Antimicrobial properties of a novel copper-based composite coating with potential for use in healthcare facilities 06 Biological Sciences 0605 Microbiology 11 Medical and Health Sciences 1117 Public Health and Health Services
13718	Mitchell, B. G.; Digney, W.; Locket, P.; Dancer, S. J.	2014	Controlling methicillin-resistant *Staphylococcus aureus* (MRSA) in a hospital and the role of hydrogen peroxide decontamination: an interrupted time series analysis
14089	Hardy, K. J.; Gossain, S.; Henderson, N.; Drugan, C.; Oppenheim, B. A.; Gao, F.; Hawkey, P. M.	2007	Rapid recontamination with MRSA of the environment of an intensive care unit after decontamination with hydrogen peroxide vapour
14130	Manian, F. A.; Griesenauer, S.; Senkel, D.; Setzer, J. M.; Doll, S. A.; Perry, A. M.; Wiechens, M.	2011	Isolation of *Acinetobacter baumannii* complex and methicillin-resistant *Staphylococcus aureus* from hospital rooms following terminal cleaning and disinfection: can we do better?
14269	French, G. L.; Otter, J. A.; Shannon, K. P.; Adams, N. M.; Watling, D.; Parks, M. J.	2004	Tackling contamination of the hospital environment by methicillin-resistant *Staphylococcus aureus* (MRSA): a comparison between conventional terminal cleaning and hydrogen peroxide vapour decontamination
14394	Kaatz, G. W.; Gitlin, S. D.; Schaberg, D. R.; Wilson, K. H.; Kauffman, C. A.; Seo, S. M.; Fekety, R.	1988	Acquisition of *Clostridium difficile* from the hospital environment

14746	Bogusz, A.; Stewart, M.; Hunter, J.; Yip, B.; Reid, D.; Robertson, C.; Dancer, S. J.	2013	How quickly do hospital surfaces become contaminated after detergent cleaning?
14850	Aucella, F.; Vigilante, M.; Valente, G. L.; Stallone, C.	2000	Systematic monitor disinfection is effective in limiting HCV spread in hemodialysis
14913	Alfa, M. J.; Lo, E.; Olson, N.; Macrae, M.; Buelow-Smith, L.	2015	Use of a daily disinfectant cleaner instead of a daily cleaner reduced hospital-Acquired infection rates

Table S7: Individual study quality assessment data

Reference			Study Quality Indicator														Total Score
Study ID	Author	Year	1	2	3	4	5	6	7	8	9	10	11	12	13	14	
67	Afinogenova	2017	1	1	1	1	0	1	1	1	0	0	0	1	0	0	8
111	Carling	2014	1	0.5	0.5	1	1	0.5	1	1	0.5	0.5	1	0.5	0.5	0.5	10
125	Casey	2010	1	1	1	1	0	1	0.5	1	0	0	0.5	1	1	0.5	9.5
128	Casini	2018	1	1	1	1	1	0.5	0	1	0	0	0.5	0.5	0.5	1	9
129	Casini	2017	1	1	1	1	1	0	1	1	0.5	0	0	0.5	0	1	8
130	Casini	2019	1	1	0.5	1	1	0.5	0	1	0	0	1	0.5	0.5	0.5	8.5
131	Casini	2018	1	0.5	0.5	1	1	0.5	0.5	1	0	0	1	1	0.5	0	7.5
177	Chen	2017	1	1	1	1	1	0.5	0.5	1	0	0	0.5	1	0.5	1	9
280	Coppin	2017	1	0	1	0.5	1	0.5	0	1	0	0	0.5	0.5	1	1	8
313	Danforth	1987	1	1	1	1	0.5	0.5	0	1	0.5	0	0.5	0.5	0.5	0	8
346	de Jong	2018	1	1	1	1	1	0.5	0.5	1	0	0	1	1	0.5	0	8.5
393	Dharan	1999	1	1	1	1	1	0.5	1	1	0	0	0	1	1	0	9.5
414	Doan	2012	0.5	1	1	0.5	1	1	0	1	1	0	1	0.5	1	1	10
442	Dramowski	2016	1	1	0.5	1	1	0.5	1	1	0	0	1	1	1	1	11
463	Dunklin	1959	1	1	0.5	1	1	0.5	0	1	0	0	0.5	0.5	0	1	8
484	Aimiya	1989	1	1	0.5	1	1	1	0	1	0	0	1	1	1	1	9.5
516	Al-Hamad	2008	1	1	0.5	1	1	0	0	1	0	0	1	1	0.5	1	8
521	Ali	2016	1	1	0.5	1	1	1	0.5	1	0	0	0.5	0.5	0.5	0.5	9
536	Allen	2019	1	1	0.5	1	1	0.5	0.5	0.5	0	0	0.5	0.5	1	0	8
567	Andersen	2006	1	1	0.5	1	1	1	0.5	1	0	1	0.5	0.5	1	0	10
568	Andersen	2009	1	1	0.5	1	1	0.5	0.5	1	0	0	0.5	1	1	1	10
606	Armellino	2020	1	1	0.5	1	1	1	1	1	0.5	0	1	0.5	0.5	0	10
621	Attaway	2012	1	1	0.5	1	1	1	1	1	0.5	0	0.5	0.5	0.5	1	10.5
686	Barbut	2009	1	0.5	0.5	1	1	1	0.5	0.5	0	0	0.5	0.5	0.5	0.5	8
766	Blazejewski	2015	1	1	1	1	1	0.5	0.5	1	0.5	0	0.5	1	0.5	0	8.5
808	Boyce	2017	1	1	1	1	0.5	0.5	0.5	1	0	1	1	0.5	0.5	0	9.5
809	Boyce	2013	1	0.5	0.5	0	1	0.5	0	0.5	0	0	0	0.5	0.5	0	5
810	Boyce	2014	1	1	1	1	0	1	0.5	1	0	0.5	0	0.5	0.5	0.5	8.5
813	Boyce	2008	1	0.5	0.5	0.5	1	0.5	0	1	0	0	0.5	0.5	0.5	0	6.5
882	Byers	1998	1	1	1	1	1	1	1	1	0	0	0	1	0.5	0	9.5
899	Eckstein	2007	1	1	0.5	1	1	1	0	1	0	0	0.5	1	0.5	1	8.5
904	Edmiston	2020	1	1	1	1	1	0	0	1	0	0	0.5	1	1	0	8.5
975	Fattorini	2018	1	1	0.5	1	1	0.5	0.5	1	0	0	1	1	0.5	1	9
997	Ferreira	2015	1	1	0.5	1	1	0.5	1	1	0	0	1	1	1	1	10
1011	Fitton	2017	1	1	1	1	0	1	0.5	1	1	0	0.5	0	0.5	0	7.5
1024	Frabetti	2009	1	1	0.5	1	1	0	0.5	1	0	0	0.5	1	1	1	8.5
1045	Frota	2017	1	0.5	0.5	0.5	1	0	1	1	0	0	0.5	1	1	1	8.5
1059	Fukada	2015	1	0.5	1	1	1	1	1	1	0	0	1	1	1	0	9.5
1065	Furlan	2019	1	0.5	0.5	0.5	1	0.5	0.5	1	0	0	0.5	1	0.5	0	7

1073	Gable	1966	1	1	0.5	1	1	1	0	1	0.5	0	0	0.5	0.5	0	8
1079	Galván Contreras	2016	1	1	1	0.5	1	0.5	0	1	0	0	1	0.5	0.5	1	8.5
1081	Gan	2017	1	0.5	1	1	1	0.5	1	1	0	0	0.5	1	1	1	9.5
1096	Garvey	2016	1	1	0	1	0	1	1	1	0	0	0	1	0	1	8
1105	Gelmini	2016	1	0	1	0.5	1	0	0	1	0	0	0.5	0	0.5	1	6.5
1171	Ghantoji	2015	1	1	1	1	1	1	1	1	0	0	0	1	0.5	0.5	10
1205	Hedin	2010	1	1	1	0.5	0	1	0.5	1	0.5	1	0.5	0.5	1	1	10
1245	Hinsa-Leasure	2016	1	1	1	0.5	1	0	0	1	0	1	0	1	0.5	0	8
1268	Holmdahl	2016	0.5	0.5	1	1	1	0.5	1	1	0	0	1	1	0.5	0	9
1280	Hosein	2016	1	1	0.5	1	1	1	1	1	0.5	1	0	1	1	0.5	11.5
1311	Huang	2015	1	1	0.5	0.5	1	0.5	0	1	0	0	1	0.5	1	0	7.5
1316	Humayun	2019	1	1	0.5	0.5	1	1	0	1	0	0	0.5	0	0.5	0	7
1416	Jinadatha	2014	1	1	1	1	1	0.5	0.5	1	0.5	0	0.5	1	1	0.5	9.5
1548	Lee	2017	1	1	1	1	1	0.5	0.5	1	0	0	1	0.5	0.5	1	10
1574	Lerner	2019	1	1	1	1	1	1	0.5	1	0	0	0.5	1	0.5	0	9.5
1585	Lewis	2015	1	1	1	1	1	0.5	0.5	1	0.5	0	1	1	1	0.5	11
1626	Gonzalez	1963	1	1	0.5	1	1	0.5	1	1	0	0	0	1	0.5	0	8.5
1723	Hall	2011	1	1	1	1	1	0.5	1	1	0	0	1	1	1	0.5	10
1886	Mosci	2017	1	1	0.5	0.5	1	1	0	1	0	0	1	1	0.5	0	8
1979	Johnson	2016	1	1	0.5	1	1	1	1	1	0	0	0	1	0	0.5	9
1991	Jones	2015	1	1	1	0.5	0.5	1	1	1	0.5	0	0.5	1	1	0	10
2035	Karunanayake	2019	1	1	1	0.5	1	1	1	1	1	0	0.5	0.5	0.5	0.5	10.5
2228	Ojajärvi	1976	1	0.5	0.5	1	0	0.5	0	0	0	0	0	0.5	0.5	0	4.5
2254	Oon	2020	1	1	0.5	1	1	1	0	1	0	0	0.5	0.5	0.5	0	8
2261	Ortí-Lucas	2017	1	1	1	1	0	1	0	1	0.5	0	1	1	1	0.5	9
2287	Özpolat	2011	0.5	1	1	1	1	0	1	0.5	0	0	0	0.5	0.5	0	7
2288	Oztoprak	2019	0.5	0.5	0.5	1	1	1	0	1	0	0	0.5	0.5	1	0	6.5
2322	Passaretti	2013	1	1	1	0.5	0	1	0.5	1	0	0	0.5	0.5	1	0	7.5
2323	Patel	2007	1	0.5	0.5	1	1	0	0	1	0	1	0.5	1	1	0	8.5
2427	Lowe	2013	0.5	1	1	1	1	0.5	1	1	0	0	1	0.5	0.5	1	10
2592	Popov	2016	1	1	0.5	1	1	1	1	1	0	0	0	1	1	0	9.5
2616	Prindis	2018	1	0.5	1	0.5	0	0.5	0	0.5	0	0	0	0	0.5	1	5.5
2653	Schmidt	2019	1	1	0.5	1	1	1	0	1	0	0	0.5	1	0.5	0.5	9
2654	Schmidt	2012	1	1	1	0.5	1	1	1	1	0	0	0	1	0.5	0.5	9.5
2655	Schmidt	2019	1	1	1	1	1	0.5	0	0.5	0.5	0	0.5	1	0.5	0.5	9
2656	Schmidt	2016	1	1	1	1	0.5	0	0.5	0	0	0	0	0	0.5	0.5	6
2707	Sexton	2011	1	1	0.5	1	1	0.5	0	1	0	0	0	1	0.5	0	7.5
2730	Shapey	2008	1	1	0.5	1	1	1	1	0.5	0.5	0	1	0.5	0	0	8
2745	Shekhawat	1992	1	1	0.5	1	1	1	1	1	0	0	0	1	0.5	0	9
2781	Sigler	2013	1	1	0.5	1	1	0	0.5	0.5	0	0	1	0.5	0.5	1	7.5
2864	Smith	1998	1	0	1	1	1	1	0	1	0	0	0	1	0	0	6
2906	Stibich	2011	1	1	0.5	1	1	1	1	1	0	0	0	1	0.5	0.5	9.5
2922	Strassle	2012	1	1	0.5	1	1	1	0	1	0.5	0	0.5	1	0.5	0	9

2926	Styaningsih	2019	1	0.5	1	1	1	0	0	1	0	0	1	0.5	0.5	1	7.5
2944	Suzuki	1984	1	0.5	0.5	1	0	1	0	1	0	0	0.5	0.5	0.5	0	6.5
3071	Reid	2018	1	0.5	1	1	1	0.5	0	1	0	0	1	1	1	1	9
3084	Reynolds	2019	0.5	1	0.5	1	0.5	0.5	0	1	0	0	0	0.5	0.5	0	6
3147	Roux	2013	1	0.5	0	1	0	0	0	1	0	0.5	0	0.5	0.5	0.5	5.5
3190	Saha	2016	1	0.5	1	0.5	1	1	1	1	0	0	0.5	0	0.5	0	7.5
3236	Santos-Junior	2018	1	1	0.5	1	1	1	0	0.5	0	0	1	0.5	1	1	8.5
3432	Youkee	2015	1	1	0.5	1	1	0.5	0.5	1	0.5	0.5	0.5	1	0	1	9.5
3444	Yui	2017	1	1	1	1	0.5	1	0.5	1	0	0	0	1	1	1	10
3507	Rathod	2019	1	1	0.5	1	0.5	0	1	0.5	0	0	0.5	0.5	0.5	0.5	7.5
3614	Vesley	1987	1	0.5	1	1	1	1	0.5	1	0	0	1	1	0.5	0.5	10
3666	Zhang	2013	1	0	0.5	0.5	1	0	0	1	0	0	0	0.5	0.5	1	6
3699	Zubair	2018	1	0.5	0.5	1	1	0.5	0	1	0	0	0	1	0	0	6.5
3854	Wilcox	2003	1	1	1	1	0	0.5	0.5	1	0	0	0.5	1	0	1	7.5
3858	Wiemken	2014	1	0.5	0.5	1	1	1	1	0.5	0	0	0.5	0.5	0.5	0	8
4060	Turner	1974	1	1	1	1	1	1	1	1	0	0	0	1	1	0	10
4132	Thom	2014	1	1	1	1	1	1	1	1	1	0	0	1	0.5	1	11.5
4146	Tekin	2013	1	1	0.5	1	1	0.5	1	1	0	0	0	1	0.5	0	8.5
4152	Taylor	2009	1	1	1	0.5	0	1	1	1	0	0	0	1	0.5	0.5	8.5
4220	Sui	2012	1	1	1	1	1	1	0	1	0	0	1	1	1	1	10
4505	Sjoberg	2014	1	0.5	0.5	0.5	0.5	0.5	0	1	0	0	0	1	0	0	5.5
4519	Singh	2017	1	0.5	1	1	1	0.5	0.5	0	0	0	0.5	0.5	0.5	0	7
4540	Siani	2018	1	0.5	1	1	1	0.5	0.5	1	0	0	0.5	0.5	0.5	0	8
4655	Schmidt	2013	1	1	1	1	1	1	1	1	0	0	0	1	1	0.5	10.5
4733	Munster	1974	1	1	0.5	1	1	1	1	1	0	1	0	1	0.5	0	10
4861	McCord	2016	1	1	0.5	1	1	1	1	1	0	1	0	1	0.5	0.5	10.5
4960	Panknin	2014	1	1	0.5	1	1	0.5	0	0.5	0	0	1	0.5	0.5	0	6.5
4992	Otter	2016	1	1	0.5	1	0	1	1	1	0	0	0	1	0	1	8.5
5106	Marais	2010	1	1	1	1	1	1	1	1	0	0	0	1	0.5	0.5	10
5113	Manian	2013	1	1	0.5	1	0.5	0.5	0	1	0	0	1	0.5	1	0	8
5183	Lowe	2013	0.5	1	1	1	1	0.5	1	1	0	0	0.5	1	0.5	1	9
5485	Le Coutour	1991	1	1	0.5	1	1	0.5	1	1	0	0	0	1	0.5	0	8.5
5623	Kitagawa	2020	1	1	0.5	1	1	1	1	1	0.5	0	0	1	1	0	10
5698	Strat	1971	1	0.5	1	0.5	1	0.5	0	1	0	0	0	1	0	0	6.5
5792	Havill	2012	1	0.5	1	1	1	1	1	1	0	0	1	1	1	0	9.5
5832	Hamilton	2010	1	1	1	1	1	0.5	0	1	0.5	0	1	1	1	1	10
5852	Hacek	2010	1	1	0.5	0.5	1	1	1	1	0	0	0	1	0.5	0	8.5
5957	Goldenberg	2012	1	1	0.5	1	1	1	1	1	0	0	0	1	1	1	10.5
6163	Ho	2016	1	0.5	0.5	0.5	1	0.5	1	1	0	0	0.5	1	1	1	9.5
6199	Chan	2011	0.5	0.5	1	0.5	1	1	0	1	0	0	0.5	1	0.5	0	7.5
6269	Čamdžić	2019	1	1	0.5	1	1	1	1	1	0	0	0	1	0	0	8.5
6287	Butin	2019	1	1	0.5	1	1	1	0.5	1	0	0	1	1	0	1	10
6368	Bokulich	2013	1	1	0.5	1	1	0.5	0	1	0	0	0.5	1	1	1	9.5

6414	Karpanen	2012	1	1	1	1	0	1	1	1	0	0	0	1	1	0.5	9.5
6482	Garvey	2018	1	1	0.5	1	0.5	0.5	0.5	0.5	0	0	0.5	1	0.5	1	8.5
6651	Daschner	1980	1	1	0.5	1	1	0	0	0	0.5	0	0.5	0.5	0	0	6
6885	Anderson	2018	1	0.5	1	0.5	0.5	0	0	1	0	1	0.5	1	1	1	9
6887	Anderson	2017	1	1	1	1	1	0	1	1	0	0	1	1	1	0	9
6888	Andersen	2006	1	0.5	0	0.5	0	1	0.5	1	0.5	0	0	1	0.5	0	6
6931	Alhmidi	2017	1	0.5	1	0.5	0	1	0.5	1	0	0	0.5	0.5	1	0.5	8
6936	Alekseeva	1969	0.5	1	0.5	0.5	1	1	0	1	0	0	0.5	1	0.5	0	7
7047	Deshpande	2014	0.5	1	1	1	1	0.5	1	0.5	0	0	0	1	0.5	0.5	8.5
7122	Best	2014	1	1	0.5	1	1	1	0.5	1	0	0	1	1	0	1	10
7455	Exner	1982	1	1	0.5	0.5	1	0	0	1	0	0	0.5	0.5	0	0	6
7468	Esolen	2018	1	1	1	1	0	1	1	1	0	0	0	1	0.5	0	8.5
7829	Taneja	2011	1	1	0.5	0.5	1	1	1	1	0	0	0.5	1	0.5	1	10
7891	Stewart	2014	1	0.5	0.5	1	1	0.5	0	1	0	0	0.5	1	0.5	1	8.5
7928	Souli	2017	1	1	1	1	1	1	1	1	0	0	0	1	1	0.5	10.5
7960	Simon Garcia	2009	1	1	0.5	1	1	0	0.5	0.5	0	0	0.5	1	1	0.5	8.5
7971	Sifri	2016	1	1	1	1	1	1	1	0	0	0	1	1	1	1	11
8042	Orenstein	2011	1	0.5	0.5	1	1	0.5	1	0	0	0	1	0.5	0.5	0	6.5
8147	Nakata	2001	1	0.5	1	0.5	1	0.5	0	0.5	0	0	0.5	1	0.5	0	7
8154	Nagai	1983	1	1	1	1	0	0.5	0	1	0	0	0.5	1	0.5	0	6.5
8312	Rai	2012	1	1	1	1	0	1	1	1	0	1	0	1	0.5	0.5	10
8380	Mayfield	2000	1	0.5	0.5	1	1	0	0.5	1	0	1	0.5	0.5	0.5	0	7
8687	Meinke	2012	1	0.5	1	1	0	0.5	0	1	0	0	0	1	1	0.5	6.5
8890	Inkinen	2017	1	0.5	1	0.5	0	1	0.5	1	0	0	0.5	1	0.5	1	8.5
9254	Fukada	2008	1	1	0	1	1	0.5	1	1	0	0	0	1	0	0	7.5
9347	Evans	2007	1	1	1	1	1	0.5	1	1	0	0	0	1	0	1	9.5
9616	Barbeito	1966	0.5	1	0.5	1	1	1	0.5	1	0	0	1	1	0	1	9.5
9651	Dyas	1983	0.5	0	0	0.5	1	0.5	0	0.5	0	0	0	1	0	0	4
9825	Codish	2015	1	1	1	0.5	1	0	0	0	0	0	0	0.5	1	0	5.5
10160	Yuen	2015	1	0.5	0.5	1	1	0.5	0.5	1	0.5	0	1	0.5	1	1	10
10314	von Dessauer	2016	1	1	1	1	1	0	0	1	0	0.5	1	1	0.5	1	9
10463	Shelly	2011	1	1	0.5	1	0	0.5	0	1	1	0	0	1	0	0	6
10553	Rutala	2018	1	1	1	1	0	0	0	1	0	0	0	1	0.5	1	6.5
10625	Ray	2010	1	0.5	0.5	1	1	0.5	1	1	0	0	1	1	1	1	9.5
10851	Tamimi	2014	1	1	0.5	1	1	1	1	1	0	1	0	1	0.5	0.5	10.5
10984	Otter	2010	1	0.5	0.5	0.5	0.5	1	0.5	0	0	0	0.5	0.5	0	0	5.5
10993	Ory	2019	1	1	0.5	1	0.5	0	0	0.5	0	0	0	1	0	1	6.5
11015	Oie	2005	1	1	0.5	1	1	1	1	1	0	0	0	1	0.5	0	9
11022	Ogino	1995	1	1	0.5	1	1	0.5	0	0	0	0	1	0.5	0	0	5.5
11135	Salgado	2013	1	1	1	1	1	0.5	1	1	0.5	1	1	1	1	0.5	12.5
11965	Fujii	1996	1	1	0.5	1	1	0.5	0	1	0	1	0.5	1	0	0	8.5
12022	Kim	2018	1	1	0.5	1	1	1	0	1	0	0	1	1	1	1	9.5
12244	Doidge	2010	1	1	0.5	1	0.5	0.5	0	0	0	0	0.5	0.5	0	0.5	5

12491	Barbut	2013	1	1	0.5	1	1	0.5	0	1	0	0	0.5	1	0.5	0	7
12894	Biswal	2017	1	1	0	0	0	0	0	1	0	0	0	0	0	1	4
12952	Bates	2005	1	0.5	0.5	0.5	0.5	0	0	1	0	0	0	0.5	0	0	4.5
13449	Otter	2007	1	1	1	1	1	0.5	0.5	1	0	0	0.5	0.5	0.5	0	8.5
13703	Montero	2019	1	1	1	1	0	0.5		1	0	0	0.5	1	0.5	0	7.5
13718	Mitchell	2014	1	1	0.5	0	1	0.5	0	1	0	0	0	0.5	1	1	7.5
14089	Hardy	2007	1	1	0.5	1	1	1	1	1	0	0	0.5	0.5	0	1	8.5
14130	Manian	2011	1	1	1	1	1	1	1	1	0	0	0	1	1	0.5	10.5
14269	French	2004	1	1	1	0.5	1	1	1	1	0	0	1	0.5	0.5	0	9
14394	Kaatz	1988	1	1	0.5	1	1	0	0.5	1	0	0	1	0.5	0.5	1	9
14746	Bogusz	2013	1	1	0.5	0.5	1	0	0.5	1	0	0	0.5	0.5	0.5	1	7.5
14850	Aucella	2000	1	0.5	1	1	1	0	1	1	0	0	0.5	1	0	0	7
14913	Alfa	2015	1	1	0.5	1	0.5	1	1	1	0	0	1	1	0.5	0.5	10

Table S8: Cohen's kappa and raw percent agreement for inter-rater variability

	**Study Quality Indictor**	**Study Quality Bias Type**	**Cohen's Kappa**	**Raw Percent Agreement**
**Study Design and Setting**	1	Study Type	0.71	0.93
	2	Setting Description	0.61	0.83
	3	Contemporary Groups	0.51	0.71
	4	Baseline equivalence	0.62	0.83
	5	Baseline outcome prevalence	0.65	0.83
**Intervention Methods**	6	Intervention description/ methods	0.67	0.78
	7	Low bias due to deviation from intervention protocol	0.59	0.73
	8	Outcome description/methods	0.62	0.88
	9	Blind evaluation	0.84	0.95
**Reporting and Analysis**	10	Low bias due to missing data	0.57	0.90
	11	Correction for Confounding	0.68	0.80
	12	Reporting based on aim	0.66	0.83
	13	Analysis	0.73	0.83
	14	Funding	0.88	0.93

Supplementary Material 3: PRISMA Checklists

Table of Contents

Table S9: PRISMA 2020 Checklist
Table S10: PRISMA 2020 Checklist Abstract

Table S9: PRISMA Checklist

Section and Topic	Item #	Checklist item	Location where item is reported
**TITLE**			
Title	1	Identify the report as a systematic review.	title
**ABSTRACT**			
Abstract	2	See the PRISMA 2020 for Abstracts checklist.	
**INTRODUCTION**			
Rationale	3	Describe the rationale for the review in the context of existing knowledge.	Introduction, page 2-3
Objectives	4	Provide an explicit statement of the objective(s) or question(s) the review addresses.	Introduction, page 3
**METHODS**			
Eligibility criteria	5	Specify the inclusion and exclusion criteria for the review and how studies were grouped for the syntheses.	Materials and Methods – Inclusion Criteria
Information sources	6	Specify all databases, registers, websites, organisations, reference lists and other sources searched or consulted to identify studies. Specify the date when each source was last searched or consulted.	Materials and Methods: Search Strategy and Machine Learning; Supplementary material 1
Search strategy	7	Present the full search strategies for all databases, registers and websites, including any filters and limits used.	Supplementary material 1
Selection process	8	Specify the methods used to decide whether a study met the inclusion criteria of the review, including how many reviewers screened each record and each report retrieved, whether they worked independently, and if applicable, details of automation tools used in the process.	Materials and Methods: Inclusion Criteria
Data collection process	9	Specify the methods used to collect data from reports, including how many reviewers collected data from each report, whether they worked independently, any processes for obtaining or confirming data from study investigators, and if applicable, details of automation tools used in the process.	Materials and Methods: Data Extraction and Risk of Bias
Data items	10a	List and define all outcomes for which data were sought. Specify whether all results that were compatible with each outcome domain in each study were sought (e.g. for all measures, time points, analyses), and if not, the methods used to decide which results to collect.	Materials and Methods: Data Extraction and Risk of Bias
	10b	List and define all other variables for which data were sought (e.g. participant and intervention characteristics, funding sources). Describe any assumptions made about	Materials and Methods: Data Extraction and

		any missing or unclear information.	Risk of Bias
Study risk of bias assessment	11	Specify the methods used to assess risk of bias in the included studies, including details of the tool(s) used, how many reviewers assessed each study and whether they worked independently, and if applicable, details of automation tools used in the process.	Materials and Methods: Data Extraction and Risk of Bias; Supplementary Material 4
Effect measures	12	Specify for each outcome the effect measure(s) (e.g. risk ratio, mean difference) used in the synthesis or presentation of results.	Supplementary Material 4 (e.g. Figure S1)
Synthesis methods	13a	Describe the processes used to decide which studies were eligible for each synthesis (e.g. tabulating the study intervention characteristics and comparing against the planned groups for each synthesis (item #5)).	None
	13b	Describe any methods required to prepare the data for presentation or synthesis, such as handling of missing summary statistics, or data conversions.	None
	13c	Describe any methods used to tabulate or visually display results of individual studies and syntheses.	Supplementary Material 4 (e.g. Figure S1)
	13d	Describe any methods used to synthesize results and provide a rationale for the choice(s). If meta-analysis was performed, describe the model(s), method(s) to identify the presence and extent of statistical heterogeneity, and software package(s) used.	Supplementary Material 4 (e.g. Figure S1)
	13e	Describe any methods used to explore possible causes of heterogeneity among study results (e.g. subgroup analysis, meta-regression).	None
	13f	Describe any sensitivity analyses conducted to assess robustness of the synthesized results.	Materials and Methods: Data Extraction and Risk of Bias
Reporting bias assessment	14	Describe any methods used to assess risk of bias due to missing results in a synthesis (arising from reporting biases).	None
Certainty assessment	15	Describe any methods used to assess certainty (or confidence) in the body of evidence for an outcome.	None
**RESULTS**			
Study selection	16a	Describe the results of the search and selection process, from the number of records identified in the search to the number of studies included in the review, ideally using a flow diagram.	Results; Figure 2
	16b	Cite studies that might appear to meet the inclusion criteria, but which were excluded, and explain why they were excluded.	None
Study characteristics	17	Cite each included study and present its characteristics.	Results; Supplementary Material 4
Risk of bias in studies	18	Present assessments of risk of bias for each included study.	Results: Study Quality
Results of individual studies	19	For all outcomes, present, for each study: (a) summary statistics for each group (where appropriate) and (b) an effect estimate and its precision (e.g. confidence/credible interval), ideally using structured tables or plots.	Supplementary Material 2
Results of	20a	For each synthesis, briefly summarise the characteristics	Results: Study

syntheses		and risk of bias among contributing studies.	Quality
	20b	Present results of all statistical syntheses conducted. If meta-analysis was done, present for each the summary estimate and its precision (e.g. confidence/credible interval) and measures of statistical heterogeneity. If comparing groups, describe the direction of the effect.	Results: Study Quality
	20c	Present results of all investigations of possible causes of heterogeneity among study results.	Discussion: Strengths and weaknesses, Disinfection Efficacy
	20d	Present results of all sensitivity analyses conducted to assess the robustness of the synthesized results.	None
Reporting biases	21	Present assessments of risk of bias due to missing results (arising from reporting biases) for each synthesis assessed.	None
Certainty of evidence	22	Present assessments of certainty (or confidence) in the body of evidence for each outcome assessed.	Supplementary Material 2
**DISCUSSION**			
Discussion	23a	Provide a general interpretation of the results in the context of other evidence.	Discussion: Strengths and Weaknesses
	23b	Discuss any limitations of the evidence included in the review.	Discussion: Strengths and Weaknesses; Disinfection Efficacy
	23c	Discuss any limitations of the review processes used.	Conclusions
	23d	Discuss implications of the results for practice, policy, and future research.	Conclusions
**OTHER INFORMATION**			
Registration and protocol	24a	Provide registration information for the review, including register name and registration number, or state that the review was not registered.	Materials and Methods
	24b	Indicate where the review protocol can be accessed, or state that a protocol was not prepared.	Materials and Methods
	24c	Describe and explain any amendments to information provided at registration or in the protocol.	None
Support	25	Describe sources of financial or non-financial support for the review, and the role of the funders or sponsors in the review.	Funding statement
Competing interests	26	Declare any competing interests of review authors.	Funding statement
Availability of data, code and other materials	27	Report which of the following are publicly available and where they can be found: template data collection forms; data extracted from included studies; data used for all analyses; analytic code; any other materials used in the review.	Supplementary materials

Table S10: PRISMA Abstract Checklist

Section and Topic	Item #	Checklist item	Reported (Yes/No)
**TITLE**			
Title	1	Identify the report as a systematic review.	yes
**BACKGROUND**			
Objectives	2	Provide an explicit statement of the main objective(s) or question(s) the review addresses.	yes
**METHODS**			
Eligibility criteria	3	Specify the inclusion and exclusion criteria for the review.	Not in abstract
Information sources	4	Specify the information sources (e.g. databases, registers) used to identify studies and the date when each was last searched.	Not in abstract
Risk of bias	5	Specify the methods used to assess risk of bias in the included studies.	Not in abstract
Synthesis of results	6	Specify the methods used to present and synthesise results.	Not in abstract
**RESULTS**			
Included studies	7	Give the total number of included studies and participants and summarise relevant characteristics of studies.	yes
Synthesis of results	8	Present results for main outcomes, preferably indicating the number of included studies and participants for each. If meta-analysis was done, report the summary estimate and confidence/credible interval. If comparing groups, indicate the direction of the effect (i.e. which group is favoured).	yes
**DISCUSSION**			
Limitations of evidence	9	Provide a brief summary of the limitations of the evidence included in the review (e.g. study risk of bias, inconsistency and imprecision).	yes
Interpretation	10	Provide a general interpretation of the results and important implications.	yes
**OTHER**			
Funding	11	Specify the primary source of funding for the review.	Not in abstract
Registration	12	Provide the register name and registration number.	No

From: Page MJ, McKenzie JE, Bossuyt PM, Boutron I, Hoffmann TC, Mulrow CD, et al. The PRISMA 2020 statement: an updated guideline for reporting systematic reviews. BMJ 2021;372:n71. doi: 10.1136/bmj.n71

Supplementary Material 4: Summary of Disinfection Intervention Efficacy

Table of Contents

Figure S1-Harvest plot of manually applied alcohol-based disinfection interventions with rows identifying outcome pathogen or HAI, columns representing effect

Figure S2-Harvest plot of manually applied peroxygen-based disinfection interventions with rows identifying outcome pathogen or HAI, columns representing effect

Figure S3-Harvest plot of manually applied quaternary ammonium compound-based disinfection interventions with rows identifying outcome pathogen or HAI, columns representing effect

Figure S4-Harvest plot of manually applied sodium hypochlorite disinfection interventions with rows identifying outcome pathogen or HAI, columns representing effect

Figure S5-Harvest plot of manually applied other chlorine-based disinfection interventions with rows identifying outcome pathogen or HAI, columns representing effect

Figure S6-Harvest plot of all other manually applied disinfection interventions with rows identifying outcome pathogen or HAI, columns representing effect

Figure S7-Harvest plot of copper surface disinfection interventions with rows identifying outcome pathogen or HAI, columns representing effect

Figure S8-Harvest plot of antimicrobial surface disinfection interventions (excepting copper) with rows identifying outcome pathogen or HAI, columns representing effect

Figure S9-Harvest plot of vaporized hydrogen peroxide disinfection interventions with rows identifying outcome pathogen or HAI, columns representing effect

Figure S10-Harvest plot of other vaporized disinfection interventions (excepting hydrogen peroxide vapor) with rows identifying outcome pathogen or HAI, columns representing effect

Manually Applied Disinfectants

Alcohol

For the purposes of this report, alcohols mixed with a QAC (Attaway et al. 2012; Bokulich et al. 2013; Schmidt et al. 2019) or with chlorhexidine gluconate (Casini et al. 2017; Fujii 1996; Jones et al. 2015) were categorized as alcohols when alcohol had the highest percentage among active ingredients.

Results

There were 20 studies included with interventions of manually-applied alcohol-based disinfectants. The majority of studies were conducted in hospitals, including academic/university, government, or community, and were often teaching hospitals. One study was conducted in a chiropractic outpatient teaching facility (Evans 2007). The critical care settings included an operating room, different types of ICUs, an outpatient urgent care clinic, a dermatological ward, a department of geriatrics, a regional burns center, a respiratory care center, a chiropractic outpatient teaching facility, and multiple unspecified hospital wards. Sixteen of the studies were conducted within countries with high-income economies including Australia (Doidge et al. 2010), Israel (Codish et al. 2015), Italy (Casini et al. 2017), Japan (Fujii 1996; Fukada et al. 2008, 2015; Oie et al. 2005), Norway (Andersen et al. 2008), Taiwan (Sui et al. 2012), the UK (Jones et al. 2015), and USA (Alhmidi et al. 2017; Bokulich et al. 2013; Evans et al. 2007; Reynolds et al. 2019; Schmidt et al. 2019). Two studies were conducted in countries with upper-middle-income economies in Brazil (Ferreira et al. 2015) and South Africa (Dramowski et al. 2016) and two in countries with lower-middle income economies in India (Biswal et al. 2017) and Pakistan (Zubair et al. 2018). The surfaces within critical care settings were predominantly high-touch surfaces in the near-patient environment, including surfaces like bed rails, bedside tables, floors, and door handles, though high-touch equipment surfaces, including keyboards, computer mice, telephones, and ventilator surfaces, were also assessed.

The efficacy of alcohol-based manually applied disinfectants in reducing bacterial load or percent surfaces positive are summarized by outcome: gram-positive organisms (bacilli and cocci), gram-negative organisms, fungi, viruses, all viable organisms, and HAIs.

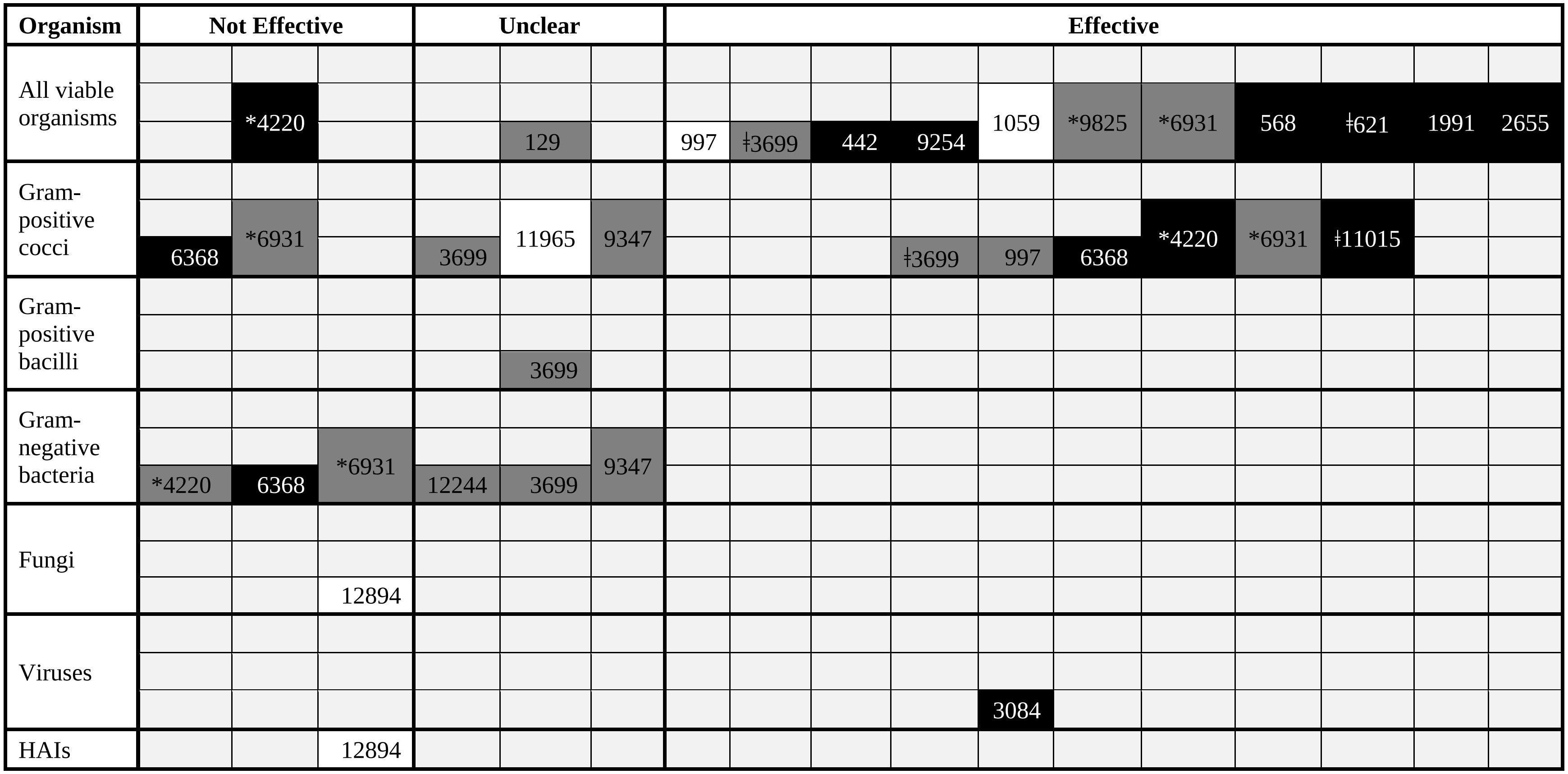

Figure S1. Harvest plot of manually applied alcohol-based disinfection interventions with rows identifying outcome pathogen or HAI, columns representing effect (not effective = disinfectant was significantly less effective or not significantly different; unclear = intervention significance or confidence intervals not specified; effective = at least one metric with significant reduction or any metric >90% or > 1 log10 reduction). ǂ denotes that study was defined as effective due to any metric reported > 90% or > 1 log10 reduction. Higher bar height represents better study design (three = controlled crossover; two = cohort or controlled before-after; one=no simultaneous control). Color represents outcome metric (black=concentration; grey = percent surfaces; white = ATP or qualitative). The number identifies the study ID (see table D1 for complete reference). * denotes the disinfectant was compared to another disinfectant rather than comparing before and after disinfection.

Gram-positive organisms

Eight studies assessed the effect of manually applied alcohol disinfectants on surfaces on gram-positive organisms including *Bacillus* spp., *Enterococcus* spp., *Micrococcus* spp., *Staphylococcus* spp., MRSA, and *Streptococcus* spp.

One study assessed gram-positive bacilli. *Bacillus* spp. was detected from 1 sample after disinfection with 70% methanol compared to 3 samples before disinfection (Zubair et al. 2018).

Eight studies assessed gram-positive cocci. For *Staphylococcus* spp., studies had significant reductions or non-detectable bacteria with alcohol-based disinfection. Though significance wasn't specified, disinfection with 80% ethyl alcohol had up to 99.99% reduction in MSSA count (range of initial mean=6.5 - 13897 CFU) with no detection of MSSA after disinfection (Oie et al. 2005). Disinfection of two ventilator surfaces with 75% ethanol with air drying had significantly lower *S. aureus* counts (no detection) compared to 75% ethanol with tissue drying or the control group (count=12 – 16 CFU) (Sui et al. 2012). One study found non-significant reduction of 60% in mean relative gene abundance (initial mean=0.05, p=0.07) after disinfection with 55% isopropyl alcohol and 0.5% QAC (Bokulich et al. 2013). There was also significant reduction in surfaces positive for *S. aureus* of 71% (initial=42.5%) after disinfection with 70% ethyl alcohol (Ferreira et al. 2015) and no detection of *S. aureus* (initial=17%) or coagulase-negative Staphylococci (initial=28%) after disinfection with 70% methanol, though significance was not specified (Zubair et al. 2018).

For MRSA, studies found reductions or significant reductions with alcohol-based disinfectants. Disinfection with 80% ethyl alcohol wipe had up to 99.99% reduction in MRSA mean count though significance was not specified (range of initial mean= 48 – 7366 CFU) (Oie et al. 2005). A 30% ethanol spray significantly reduced the percent of surfaces positive by 83% compared to control surfaces (control= 7.6%) (Alhmidi et al. 2017) and 70% ethyl alcohol had significant reductions of 59% in surfaces positive after compared to before disinfection (initial=22%) (Ferreira et al. 2015). MRSA was not detected after disinfection with 0.5% chlorhexidine digluconate but was detected after disinfection with 0.2% concentration (Fujii 1996). There was no significant difference between 30% ethanol and 0.65% sodium hypochlorite on MRSA (Alhmidi et al. 2017).

For other gram-positive cocci, efficacy was mixed, possibly due to low initial burden. Disinfection with 30% ethanol found non-significant (p=0.07) reduction of 87% in percent surfaces positive for VRE (initial=4.5%) compared to the water control (Alhmidi et al. 2017). Disinfection with 55% isopropyl alcohol and 0.05% QAC resulted in no change (p>0.1) in the mean relative abundance of *Enterococcus* spp. (initial mean= 0.0006) and significant decrease in the mean relative abundance of *Streptococcus* spp. (initial mean= 0.025) (Bokulich et al. 2013). *Micrococcus* spp. was not detected after disinfection with 70% methanol compared to 2 samples before disinfection (Zubair et al. 2018). Unspecified gram-positive organisms were not detected on five tables after disinfection with 70% isopropyl alcohol and 10% acetone (Evans et al. 2007).

Gram-negative organisms

Six studies assessed the effect of manually applied alcohol-based disinfectants on surfaces on gram-negative organisms including *Acinetobacter* spp., CRAB, *Citrobacter* spp., *Escherichia* spp., *Klebsiella* spp., *Proteus* spp., *Pseudomonas* spp., and gram-negative bacilli.
None of the studies found significant effects of alcohol-based disinfectants on gram-negative organisms, although some studies found reductions with significance not specified due to missing analysis.

For gram-negative organisms generally, disinfection with 30% ethanol found non-significant ($p=0.07$) reduction of 99% in percent surfaces positive for gram-negative bacteria (initial=4.5% surfaces positive) compared to the water control (Alhmidi et al. 2017). Two of five tables were positive for gram-negative organisms. Disinfection with 70% isopropyl alcohol and 10% acetone had no detection of gram-negative bacteria (initial=two of five tables positive) (Evans et al. 2007).

For *Acinetobacter* spp., alcohol-based disinfection found reductions in percent surfaces positive. Disinfection with 70% alcohol wipes found reduction of 82% (significance not specified) in percent surfaces positive for CRAB (initial=8% surfaces positive) (Doidge et al. 2010). Disinfection with 70% methanol found reduction of 90% in percent surfaces positive for *Acinetobacter* spp. (initial=19% surfaces positive) (Zubair et al. 2018).

For other gram-negative organism, there were reductions (significance not specified) in surfaces positive after disinfection with 70% methanol for *Klebsiella* spp. (before=22%, after=0%), *Pseudomonas* spp. (before=4%, after=2%), *Citrobacter* spp. (before=9%, after=0%), *Escherichia coli* (before=9%, after=0%), and *Proteus* spp. (before=2%, after=0%) (Zubair et al. 2018). There was no difference in efficacy of 75% ethanol compared to water control on *Pseudomonas aeruginosa* detection on 3 ventilator surfaces (Sui et al. 2012). Disinfection with 55% isopropyl alcohol and 0.5% QAC had no effect ($p>0.1$) on the mean relative abundance of *Acinetobacter* spp. (initial mean= 0.066), *Escherichia* spp. (initial mean= 0.0003), *Klebsiella* spp. (initial mean= 0.0005), and a significant increase ($p=0.023$) in the mean relative abundance of *Pseudomonas* spp. (initial mean= 0.016) (Bokulich et al. 2013).

Fungi

One study examined the efficacy of 70% alcohol against *Candida auris* in an outbreak setting, qualitatively finding that surfaces remained contaminated after disinfection (Biswal et al. 2017).

Virus

One study examined the efficacy of 29% ethanol against the MS2 bacteriophage finding a 94% reduction in the geometric mean viral count after disinfection compared to baseline (initial mean= ~60 PFU/cm^2) (Reynolds et al. 2019).

All viable organisms

Thirteen studies assessed alcohol-based disinfectants on all viable organisms, with three adding chlorhexidine gluconate and two adding a quaternary ammonium compound.

Of studies assessing the mean concentration of all viable organisms, there was mixed efficacy. Alcohol significantly reduced mean concentration by 88.3% (before=300 CFU/ml, after=35 CFU/ml) (Fukada et al. 2008), mean count by 64.3% with 5% propanol wet mopping (before=99 CFU, after= 35 CFU) (Andersen et al. 2008) and mean count by 61.5% with 70% alcohol (before=39 CFU, after=15 CFU) (Dramowski et al. 2016). However, one controlled cohort study found that 75% alcohol disinfection with air drying and with tissue drying did not have significantly different bacterial counts compared to the control using water (range of median= 36- > 500 CFU) (Sui et al. 2012).

Alcohol-based disinfection had significantly lower percent surfaces positive for bacterial growth by 85% compared to sterile water control surfaces (control=17% surfaces positive) (Alhmidi et al. 2017) and by 91% reduction compared to before disinfection with 70% methanol (initial=96% surfaces positive) (Zubair et al. 2018). There were significant reductions in mean ATP after disinfection with 70% ethyl alcohol (range of initial mean= 692 – 21850 RLU) (Ferreira et al. 2015) and 77% ethanol (range of initial mean= 691-5167 RLU) (Fukada et al. 2015). Alcohol-based disinfectants combined with chlorhexidine gluconate were effective. Median counts were significantly lower after disinfection intervention (median= 0 CFU) compared to the baseline before the intervention (median > 500 CFU) (Jones et al. 2015). Disinfection with 70% isopropyl alcohol, 0.5% chlorhexidine, and 0.45% hydrogen peroxide had significantly fewer surfaces with high-risk pathogens than did a QAC (Codish et al. 2015). The percent surfaces deemed unacceptable (>20 CFU/100cm^2) was less after cleaning with a chlorhexidine-alcohol combined disinfectant compared to before and compared to hypochlorite cleaning (significance not specified) (Casini et al. 2017).

For surfaces disinfected with alcohol combined with a quaternary ammonium compound, 70% ethanol and QAC significantly reduced median concentrations 1 h, 6 h, and 24 h after disinfection (initial=6.75 CFU/cm^2 and 3.6 CFU/cm^2*)* (Schmidt et al. 2019). Disinfection with 17% isopropanol and QAC had significantly reduced median concentrations after 1 h and 6 h after disinfection (initial=9.9 CFU/cm^2) (Schmidt et al. 2019). A second study assessing disinfection with 17% isopropanol and QAC had a mean relative reduction of the bacterial population on bed rails of 99% at 30 minutes after disinfection (initial mean=580 CFU/cm^2) (Attaway et al. 2012). The 17% isopropanol combined with QAC had significantly lower concentration than a QAC alone (Attaway et al. 2012).

<u>HAIs</u>

One study assessed 70% alcohol disinfection in an outbreak setting in a trauma ICU on *C. auris* infections finding that after disinfection of ECG leads and blood pressure monitoring cuffs, all 10 patients without *C. auris* colonization at admission acquired *C. auris* by the fourth day of being in the ICU (Biswal et al. 2017).

Peroxygen

Peroxygen disinfectants include hydrogen peroxide, peracetic acid, and peroxymonosulfate disinfectants.

Results

A total of 17 articles relating to peroxygen intervention methods in healthcare facilities were analyzed and reviewed. Interventions included peracetic acid wipes, hydrogen peroxide wipes, improved or accelerated hydrogen peroxide, combined peracetic acid and hydrogen peroxide, and peroxymonosulfate of varied concentrations applied with wipes, mops, cotton cloths, and microfiber cloths. All but one of the studies were from high-income countries including Australia (Doidge et al. 2010; Mitchell et al. 2014), Canada (Alfa et al. 2015), Japan (Fukada et al. 2015), Sweden (Sjöberg et al. 2014), Switzerland (Dharan et al. 1999), the UK (Doan et al. 2012; Saha et al. 2016; Siani et al. 2018; Yui et al. 2017), and the USA (Armellino et al. 2020; Boyce et al. 2017; Boyce and Havill 2013; Carling et al. 2014; Deshpande et al. 2014; Wiemken et al. 2014). One study was in a lower-middle income country in India (Biswal et al. 2017). The settings of the reviewed studies included intensive care units (ICUs), isolation rooms, children’s wards, women’s wards, surgical wards, elderly care wards, and operating rooms in hospitals and healthcare facilities around the world. The types of environmental surfaces ranged from a variety of common, high-touch locations in hospital environments. These surfaces include, but are not limited to, bed rails, door handles, light switches, nurse call bells, bed table, phones, tray tables, bathroom doors, bathroom handles, toilet seats, and toilet handles.

The efficacy of peroxide acid-based disinfectants in reducing bacterial load or percent surfaces positive are summarized by outcome: gram-positive organisms (bacilli and cocci), gram-negative organisms, fungi, all viable organisms, and HAIs.

Organism	Not Effective	Unclear		Effective							
All viable organisms			*4540	ǂ3858	ǂ809	1059	*111	606	*393	*808	*4540
Gram-positive cocci						13718	7047				
Gram-positive bacilli		4505	3444			7047	ǂ414				
Gram-negative bacteria						*3190					
Fungi			12894								
HAIs	3190		*808				12244	14913	13718		

Figure S2. Harvest plot of manually applied peroxygen-based disinfection interventions with rows identifying outcome pathogen or HAI, columns representing effect (not effective = disinfectant was significantly less effective or not significantly different; unclear = intervention significance or confidence intervals not specified; effective = at least one metric with significant reduction or any metric >90% or > 1 log10 reduction). ǂ denotes that study was defined as effective due to any metric reported > 90% or > 1 log10 reduction. Higher bar height represents better study design (three = controlled crossover; two = cohort or controlled before-after; one=no simultaneous control). Color represents outcome metric (black = concentration; grey = percent surfaces; white = ATP or qualitative). The number identifies the study ID (see table D1 for complete reference). * denotes the disinfectant was compared to another disinfectant rather than comparing before and after disinfection.

Gram-positive organisms

Five studies assessed the effect of manually applied peroxygen disinfectants on surfaces against gram-positive organisms including *C. difficile*, MRSA, and VRE.

Four studies assessed gram-positive bacilli. There was a 2.1 log reduction in concentration of inoculated *C. difficile* after disinfection with peracetic acid wipes (Doan et al. 2012). There was a significant reduction in percent surfaces positive for *C. difficile* after the use of a combined peracetic acid-hydrogen peroxide wipe compared to a QAC product (Deshpande et al. 2014). In single-isolation rooms with known *C. difficile* colonized patients there was a 76% reduction in mean count after terminal cleaning the mean count (initial mean=87 CFU) (Yui et al. 2017) though significance was not specified and contemporary control not included in the study design. There was a 78% reduction to 3% surfaces positive for *C. difficile* (initial=23% positive) after disinfection with 21.4% potassium monopersulfate-based disinfectant (Sjöberg et al. 2014). Two studies assessed gram-positive cocci. There were significant reductions in percent surfaces positive for MRSA or VRE with no recovery after the use of peracetic acid-hydrogen peroxide wipes (initial=22%) (Deshpande et al. 2014) and a 22% reduction in MRSA after the use of either manually applied or vaporized hydrogen peroxide (results not disaggregated; initial=24.7%) (Mitchell et al. 2014).

Gram-negative organisms

One study assessed the efficacy of peracetic acid wipes compared to routine cleaning on two different wards with similar baseline bioburden finding that finding that there were significantly more positive surfaces in wards implementing routine cleaning with a QAC and isopropyl alcohol (17%) compared to the intervention ward (Saha et al. 2016).

Fungi

One study did not report recovery of *Candida auris* after floors were cleaned with mops soaked in hydrogen peroxide with silver nitrate, however number of samples before and after disinfection were unspecified (Biswal et al. 2017).

Virus

No studies assessed effects of peroxygen interventions on viruses.

All viable organisms

Eight studies assessed peroxygen efficacy on all viable organisms across environmental surfaces in healthcare facilities. All studies found a significant effect of the peroxygen intervention.

A crossover trial with improved hydrogen peroxide had significantly lower mean count per surface compared to a QAC (control mean= 22.2 CFU) (Boyce et al. 2017). Additionally, a crossover study reported a significant reduction in total aerobic count with a peracetic acid/hydrogen peroxide wipe compared with detergent followed by chlorine-based disinfectant (Siani et al. 2018). The percent of samples positive for MDROs (VRE, CRE, ESBL) was higher on wards using detergent and chlorine-based disinfectant (1.3-3%) compared to wards using peracetic acid/hydrogen peroxide wipes (0.6-1%) (Siani et al. 2018). A cohort study found that a potassium peroxymonosulfate disinfectant had significantly lower bacterial load on floors by 95% compared to a QAC and compared to a detergent (Dharan et al. 1999). Hydrogen peroxide/peracetic acid was 1.93 times more effective than QAC (initial=15-17 CFU) (Carling et al. 2014) and there was similar efficacy when comparing hydrogen peroxide to 77% ethanol (Fukada et al. 2015).

Comparing before to after disinfection with hydrogen peroxide wipes, there was a 38% reduction in mean count per operating room compared to before disinfection (initial 87 CFU) (Armellino et al. 2020), no recovery from 75% of surfaces (initial median=63.1 CFU) (Boyce and Havill 2013), 84-96% reduction in ATP (Wiemken et al. 2014), and significant reduction in ATP in two of five surfaces (initial range=573-2970 RLU) (Fukada et al. 2015).

HAIs

Five studies assessed HAI outcomes from disinfection interventions using manually-applied peroxygen disinfectants.

In a 12-month crossover trial, incidence density rates for HAIs due to VRE (5.49 vs. 6.6 cases/1000 patient-days), *C. difficile* (0.56 vs. 1.0 cases/1000 patient-days), MRSA (1.96 vs. 2.79 cases/1000 patient-days), and composite incidence density rates (8.0 vs. 10.3 cases/1000 patient-days, p=0.068) were lower on wards implementing daily cleaning with 0.5% improved hydrogen peroxide compared to wards cleaning with a QAC (Boyce et al. 2017).
MRSA incidence was significantly lower in the 3-year period using hydrogen peroxide vapor or manually applied hydrogen peroxide (results not disaggregated by mode of application) terminal cleaning compared to the 4-year period using only detergent (5.3 vs. 9.0/10,000 patient-days, p<0.001) (Mitchell et al. 2014).

One study introduced disinfection of high-touch surfaces with hydrogen peroxide wipes compared to a hospital that did not introduce the new disinfection step finding a significant reduction in HAIs due to *C. difficile* (3.0 vs. 6.04 cases/10,000 patient-days), MRSA (11.43 vs. 2.5 cases/10,000 patient-days), and VRE (25 vs. 14 cases/10,000 patient-days when cleaning compliance was high in the intervention hospital compared to a hospital (Alfa et al. 2015).
One study implemented a new disinfection product with active ingredient potassium peroxymonosulfate to replace routine cleaning with alcohol-based wipes during an outbreak of CRAB, and there was temporal association between the introduction of the new product and end of the outbreak (Doidge et al. 2010).

Finally, one study found that HAIs due to gram-negative organisms were not significantly different in wards with routine cleaning using QAC wipes and 70% isopropyl alcohol wipes compared to peracetic acid wipes (Saha et al. 2016).

Quaternary Ammonium Compounds

For the purposes of this report, disinfectants were categorized as QACs if the QAC was the active ingredient with the highest percent concentration. Among the products included in the articles reviewed, didecyl dimethyl ammonium chloride and benzyl ammonium chloride were common active ingredients for QACs.

Results

There were 45 studies that included QAC products for disinfection on environmental surfaces in healthcare facilities. These include studies assessing efficacy of disinfectants when the QAC was used as the control in standard or routine cleaning as well as when the QAC was explicitly studied as part of the intervention.

The majority of studies were in countries with high-income economies, including France (Blazejewski et al. 2015; Butin et al. 2019; Le Coutour and Oblin 1991; Roux et al. 2013), Hong Kong (Yuen et al. 2015), Israel (Codish et al. 2015), Italy (Casini et al. 2018a; Fattorini et al. 2018), Japan (Fujii 1996; Kitagawa et al. 2020; Suzuki et al. 1984), Romania (Strat 1971), Switzerland (Dharan et al. 1999), the UK (Bogusz et al. 2013; Garvey et al. 2018; Otter et al. 2007, 2016; Saha et al. 2016), and the USA (Anderson et al. 2017, 2018; Attaway et al. 2012; Boyce et al. 2014, 2017; Byers et al. 2020; Carling et al. 2014; Deshpande et al. 2014; Eckstein et al. 2007; Evans et al. 2007; Fitton et al. 2017; Hacek et al. 2010a; Hinsa-Leasure et al. 2016; Lewis et al. 2015; Mayfield et al. 2019; Passaretti et al. 2013; Rutala et al. 2018; Schmidt et al. 2019; Sigler and Hensley 2013; Strassle et al. 2012; Vesley et al. 1987). There were six studies from upper-middle income countries including Brazil (Santos-Junior et al. 2018), Indonesia (Styaningsih et al. 2019), Mexico (Gonzalez et al. 2012), and Russia (Panknin 2014). Among the most commonly sampled environmental surfaces were high-touch points such as bedrails, door handles, tray tables, bedside tables, call buttons, light switches, toilets, and computer equipment. Some studies also focused specifically on floors and incubators. The surfaces were measured in critical care settings which included *Clostridium difficile* (CDI) and MRSA isolation rooms, an elder-care ward, intensive care units (ICUs), internal medicine wards, a medical-surgical suite, medical ICUs (MICUs), surgical ICUs, cardiac ICUs, neonatal ICUs (NICUs), a neurosurgery ward, an outpatient clinic, a walk-in emergency clinic, and several Veterans' Affairs medical clinics (VAs).

The efficacy of manually applied quaternary ammonium compound disinfectants in reducing bacterial load or percent surfaces positive are summarized by outcome: gram-positive organisms (bacilli and cocci), gram-negative organisms, fungi, all viable organisms, and HAIs.

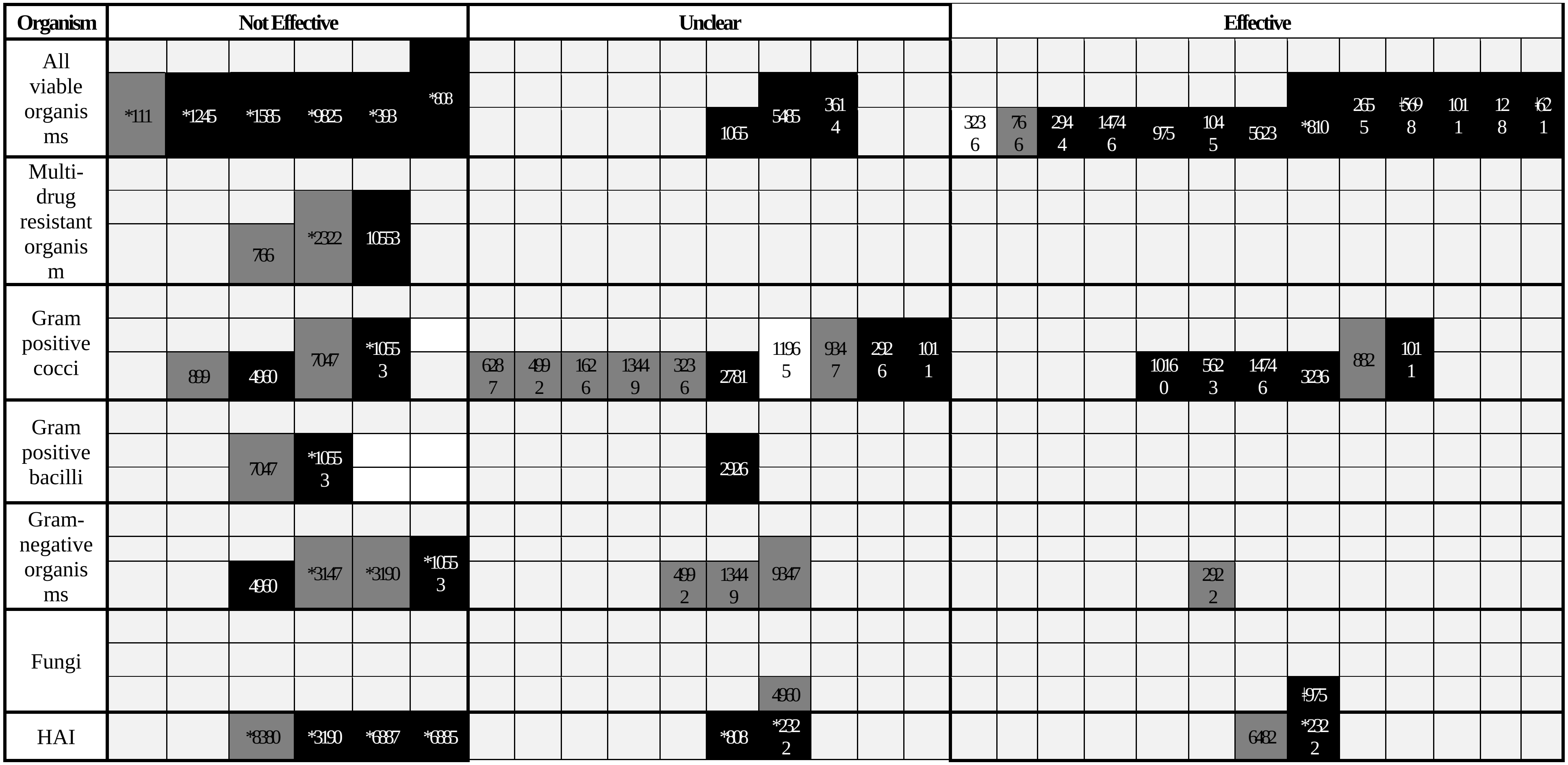

Figure S3. Harvest plot of manually applied quaternary ammonium compound-based disinfection interventions with rows identifying outcome pathogen or HAI, columns representing effect (not effective = disinfectant was significantly less effective or not significantly different; unclear = intervention significance or confidence intervals not specified; effective = at least one metric with significant reduction or any metric >90% or > 1 log10 reduction). ǂ denotes that study was defined as effective due to any metric reported > 90% or > 1 log10 reduction. Higher bar height represents better study design (three=controlled crossover; two = cohort or controlled before-after; one=no simultaneous control). Color represents outcome metric (black = concentration; grey = percent surfaces; white = ATP or qualitative). The number identifies the study ID (see table D1 for complete reference). * denotes the disinfectant was compared to another disinfectant rather than comparing before and after disinfection.

<u>Gram-positive organisms</u>

There were 18 studies assessing efficacy of QACs on gram-positive organisms including *Bacillus* spp., *C. difficile, Enterococcus* spp., VRE, *Staphylococcus* spp., MRSA, and *Streptococcus* spp. Results were mixed for QAC efficacy on different identified gram-positive pathogens and no studies implemented crossover trials.

Three studies assessed gram-positive bacilli and did not find significant effects of QACs on gram-positive bacilli reductions. One study found that QACs did not significantly reduce surfaces positive for *C. difficile* (Deshpande et al. 2014). One study found similar concentrations of *C. difficile* when using a QAC compared to bleach, finding no significantly different mean concentration of *C. difficile* following disinfection (mean=4.48 CFU) (Rutala et al. 2018). *B. subtillis* was not recovered after disinfection with QACs, though there was low initial concentration (2.33CFU/cm^2) (Styaningsih et al. 2019). QACs did not significantly reduce recovery of *C. difficile,* while bleach and peracetic acid significantly recuded recovery of *C. difficile* (Deshpande et al. 2014).

Eighteen studies assessed gram-positive cocci. A few studies found significant reductions in *Staphylococcus* spp. after disinfection. One study reported a significant reduction in total *Staphylococcus* concentration when using a QAC and when using a placebo saline solution (Fitton et al. 2017). Additionally, there were significantly higher reductions when using the QAC compared to the placebo (Fitton et al. 2017). Compared to before disinfection, significant reductions were reported on one of five surfaces for *S. aureus* by 67% immediately after disinfection (initial median=10.5 CFU) (Santos-Junior et al. 2018), reductions of 83% for MSSA and MRSA 2-4 hours after disinfection (initial total count=12 CFU) (Bogusz et al. 2013), and reduction in median MRSA concentration immediately after disinfection (initial median=4.0 CFU) (Kitagawa et al. 2020). Some studies found reductions but did not report significance for *S. aureus* up to 52% (initial=18-56%) (Gonzalez et al. 2012), for MRSA by 33% (initial=60%) (Otter et al. 2007), 50% (initial=22%) (Santos-Junior et al. 2018), and 33% (initial=60%) (Otter et al. 2016), and for *S. capitis* in a NICU by 75% (initial=44%) (Butin et al. 2019). There was no recovery of gram-positive organisms after disinfection (initial=5 tables positive) in one study (Evans et al. 2007), and no recovery of *S. aureus* (initial=6 CFU/cm^2) or *S. epidermidis* (initial=7.7 CFU/cm^2) (Styaningsih et al. 2019) after disinfection. A qualitative study recovered MRSA after disinfection with 0.2% benzalkonium chloride but not after increasing the concentration to 0.5% (Fujii 1996). There were higher average MRSA counts in rooms using QAC (8.5 CFU) compared to rooms using sodium hypochlorite (4.4 CFU), though not significantly so (Rutala et al. 2018).

No studies assessed the concentration of VRE before compared to after QAC disinfection; however, QACs reduced surfaces positive for VRE by 19% (initial=71%, significance not specified) (Eckstein et al. 2007), and significantly reduced surfaces positive with no recovery for VRE after 10 min contact time bucket disinfection (Byers et al. 2020). One study reported that there was not a significant reduction in percent surfaces positive for MRSA or VRE after disinfection with QACs (initial=22%) (Deshpande et al. 2014). Three studies did not find reductions due to low initial prevalence of VRE (Fitton et al. 2017; Otter et al. 2007, 2016).

There were not significantly different mean concentrations of VRE in rooms cleaned with QACs (mean=39.6 CFU) compared to bleach (mean=2.4 CFU) (Rutala et al. 2018).

Two studies assessed gene concentration for gram-positive cocci generally finding no difference before compared to after disinfection with QACs. Average gene density was not significantly different before compared to after disinfection for *Enterococcus* spp. and *Staphylococcus* spp., though there was a significant reduction in *Streptococcus* spp. genes after disinfection (Panknin 2014). Most samples had multiple *Staphylococcus* spp. gene markers before and after disinfection (Sigler and Hensley 2013). QACs significantly decreased the average gene density of *Streptococcus* when comparing before and after terminal disinfection (Panknin 2014).

<u>Gram-negative organisms</u>

There were 8 studies assessing the efficacy of QACs on gram-negative organisms including *Acinetobacter* spp., *Escherichia* spp, *Klebsiella* spp., *Pseudomonas* spp., and others.
One study reported significant reductions in gram-negative organisms after disinfection with QACs. Compared to before disinfection, there were significantly lower number of rooms with surfaces positive for MDR *Acinetobacter* (Strassle et al. 2012), 67% fewer surfaces positive for gram-negative rods (significance not assessed) (Otter et al. 2007), and 67% fewer surfaces positive for MRSA (significance not assessed) (Otter et al. 2016) after terminal cleaning with QACs. One study reported no recovery of gram-negative organisms after disinfection (initial=2 surfaces positive) (Evans et al. 2007).

When compared to other disinfectants, QACs were not significantly better than another disinfectant for gram-negative organisms. Compared to terminal cleaning with sodium hypochlorite, there was a significantly higher concentration of MDR *Acinetobacter* spp. using QACs (mean=8.95 CFU) compared to using sodium hypochlorite (mean=0.39 CFU) (Rutala et al. 2018). Wards cleaned with sodium hypochlorite had significantly lower detection of gram-negative organisms (4%) compared to wards with QAC (17%) (Saha et al. 2016) and sinks disinfected with sodium hypochlorite had significantly lower detection of ESBL-*Enterobacteriaceae* (0%) than sinks disinfected with QAC (36%) (Roux et al. 2013).
Following disinfection with QACs, no significant reduction was found in the mean relative abundance of *Acinetobacter* spp., *Escherichia* spp. or *Klebsiella* spp. and a significant increase was recorded in the mean relative abundance of *Pseudomonas* following disinfection when compared to before (p=0.02) (Panknin 2014).

<u>Fungi</u>

Two studies assessed efficacy of QACs on fungi. One study assessed yeast concentration in a neonatal pediatric unit and found a 98.5% mean reduction after QAC disinfection with 10-minute contact time (Fattorini et al. 2018). A second study assessed gene abundance of *C. albicans* but did not recover genes before or after disinfection (Panknin 2014).

<u>Virus</u>

There were no studies assessing efficacy of QACs on virus outcomes.

All viable organisms

There were 24 studies that assessed the effect of QACs on all viable bacteria. Most studies found reductions in bacterial concentration after disinfection with QAC product compared to before disinfection. However, when QACs were compared to other disinfectant products, QACs usually were significantly less effective.

Compared to before disinfection with a QAC, there was a significantly lower mean concentration of 92% (mean reduction=205 CFU) (Fattorini et al. 2018), 93% reduction in median concentration (initial load median: 2.7 CFU/cm^2) (Frota et al. 2017), 90% reduction in mean concentration after QAC wipe and QAC aerosolization (Strat 1971), 77% reduction in mean concentration (initial load: 7.5 CFU) on surfaces of equipment and 92% (initial load: 2.6 CFU) on operating room floors (Suzuki et al. 1984), 72% reduction in mean concentration in the first hour after disinfection (initial concentration: 480 CFU/100 cm^2) (Schmidt et al. 2019),71% reduction in mean concentration (initial mean= 52 CFU/25 cm^2) after 0.5 hours which remained comparatively low up to 6.5 hours after disinfection (Casini et al. 2018a), 66% reduction in mean concentration and significantly more effective compared to placebo saline solution (Fitton et al. 2017), 52% reduction in mean concentration of bacterial load (initial load average: 29.8 CFU) (Kitagawa et al. 2020), and 49% reduction in mean concentration (initial mean/site=6.7 CFU/cm^2) 4 hours after disinfection (Bogusz et al. 2013). There were also significant reductions in other measures of bacteria including a significant reduction of 51% in the number of rooms positive for at least one bacterium (initial=77%) (Blazejewski et al. 2015) and a significant reduction in ATP for two of five high-touch surfaces (range of initial median=358-946 RLU) (Santos-Junior et al. 2018).

While significance was not assessed comparing before and after disinfection with QACs, there were reductions in concentration of 80% (initial mean: 47 CFU/cm^2) (Furlan et al. 2019), 83.1% in floors with higher reductions in winter compared to summer (Vesley et al. 1987), a 45% decrease in concentration (initial mean: 3,711 CFU/100 cm^2) for one QAC and a 99% decrease in concentration for a second QAC (initial mean= 5,800 CFU/ 100 cm2) (Attaway et al. 2012), and log reductions ranging from 0.53-0.79 log CFU (Le Coutour and Oblin 1991).

A few studies found minimal reductions after disinfection with QACs with a mean reduction of 0.6 CFU/24 cm^2 (significance not specified) (Dharan et al. 1999). There was not a significant reduction in number of rooms with surfaces positive for MDROs after disinfection with QAC (initial=8%) (Blazejewski et al. 2015).

Nine studies compared efficacy of QACs on all viable organisms to another disinfectant finding that QACs were significantly less effective in seven of the studies, the same as sodium hypochlorite in one study, and significantly better than one antimicrobial surface treatment.

Three studies found that surfaces disinfected with manually applied peroxygen disinfectants were significantly more effective than QACs. Improved hydrogen peroxide had significantly lower mean concentration (14 CFU) compared to surfaces disinfected with QAC (mean=22 CFU) (Boyce et al. 2017). Hydrogen peroxide/peracetic acid disinfectant was 1.93 times more effective at removing bacteria compared to QAC ($p<0.001$) (Carling et al. 2014). Potassium

peroxymonosulfate had significantly higher reductions in mean concentration (111 CFU/24 cm^2) on ward floors compared to QAC (0.6 CFU/24 cm^2) (Dharan et al. 1999). There was a significantly higher percent of surfaces negative for high-risk pathogens after disinfection with alcohol-based disinfectant compared to QAC (Codish et al. 2015). Disinfection with sodium hypochlorite also had higher reductions in concentrations compared to QACs, though results were not significant, with average concentration of target pathogens (including MDROs) at 61 CFU/room using QACs compared to 12 CFU/room when using sodium hypochlorite (Rutala et al. 2018). Among vaporized disinfectants, there were significantly more surfaces positive for MDROs in rooms using standard cleaning with QACs compared to rooms using HPV (Passaretti et al. 2013). In three studies assessing efficacy of antimicrobial surface applications, QAC disinfection was used for routine cleaning on treated and untreated surfaces. There were mixed results. After application of antimicrobial isopropyl alcohol/organofunctional silane spray and continued standard cleaning with QACs, mean concentration among surfaces positive for bacteria was significantly higher on untreated surfaces (14.3 CFU) compared to treated surfaces (1.7 CFU) (Lewis et al. 2015). With routine cleaning using QACs, mean concentration during the 12-month intervention period for control components was significantly higher at 6,172 CFU/100 cm^2 compared to rooms with copper components at 117 CFU/100 cm^2 (Hinsa-Leasure et al. 2016). Control sites cleaned with QACs had a significantly lower mean colony count compared to the test products (Boyce et al. 2014).

<u>HAIs</u>

Eight studies assessed the effect of a QAC disinfection on HAI outcomes.

One study found significant reductions in HAI-outcome with the use of QAC. The average MRSA acquisition was 9.4 per 100,000 patient-bed days during implementation of QAC wipe intervention compared to 20.7 per 100,000 patient-bed days with baseline cleaning ($p<0.05$) (Garvey et al. 2018). However, this study was unable to determine if improvements in HAI incidence were due to the QAC product or due to the implementation of a simplified protocol (i.e. one wipe vs. two wipe).

Six other studies compared standard cleaning with QAC with a different disinfection intervention. While most studies did find significant differences on most outcomes between QACs and the disinfection intervention, four studies found that QAC performed significantly worse than the comparator for VRE (Anderson et al. 2017; Passaretti et al. 2013) and *C. difficile* (Hacek et al. 2010b; Mayfield et al. 2019).

In a nine-hospital multicenter trial, standard terminal cleaning with QACs was not significantly different than terminal cleaning using bleach for overall target HAI pathogens including hospital-wide incidence (Anderson et al. 2018) and exposed patient incidence (Anderson et al. 2017). However, there was a significant reduction in incidence of VRE by 57% ($p=0.049$) for patients exposed to terminally-cleaned rooms with bleach compared to QACs (Anderson et al. 2017), though the efficacy did not hold true for hospital-wide incidence of VRE. Another study found that *C. difficile*-associated disease incidence rate decreased significantly from 8.6 to 3.3 cases per 1000 patient-days when switching from QAC to sodium hypochlorite and incidence rates increased from 3.2 to 8.1 cases per 1000 patient-days when switching back to QAC from sodium

hypochlorite disinfection (Mayfield et al. 2019). The number of patients with *C. difficile* infections were significantly lower after replacing QAC disinfection with bleach for terminal cleaning (0.85 patients per 1000 patient-days to 0.45 patients per 1000 patient-days) (Hacek et al. 2010b). One study compared HAI incidence of patients in rooms with standard cleaning plus additional HPV cleaning compared concurrently to patients in rooms with standard cleaning alone and found reductions, but not significant differences, in HAIs due to MRSA (risk ratio=0.53, 95% CI=0.16 – 1.79), *C. difficile* (risk ratio= 0.49, 95% CI= 0.16 – 1.47), and MDR-gram-negative rods (risk ratio=0.55, 95% CI=0.20 – 1.57) (Passaretti et al. 2013). However, the study did find significant reductions in VRE HAIs (risk ratio=0.25, 95% CI=0.10 – 0.60). In a 12-month crossover trial, incidence density rates for HAIs due to VRE (5.49 vs. 6.6 cases/1000 patient-days), *C. difficile* (0.56 vs. 1.0 cases/1000 patient-days), MRSA (1.96 vs. 2.79 cases/1000 patient-days), and composite incidence density rates (8.0 vs. 10.3 cases/1000 patient-days, p=0.068) were lower on wards implementing daily cleaning with 0.5% improved hydrogen peroxide compared to wards cleaning with a QAC (Boyce et al. 2017). Finally, one study found that HAIs due to gram-negative organisms were not significantly different in wards with routine cleaning using QAC wipes and 70% isopropyl alcohol wipes compared to wards using peracetic acid wipes (Saha et al. 2016).

Sodium Hypochlorite

For the purposes of this report, disinfectants were categorized as sodium hypochlorite (i.e. bleach) if the disinfectant was specified as sodium hypochlorite. Unspecified hypochlorite disinfectants were categorized as other chlorine disinefectants.

Results

There were 34 studies that included sodium hypochlorite for disinfection on environmental surfaces in healthcare facilities. The majority of studies were in countries with high-income economies, including France (Barbut et al. 2009; Roux et al. 2013), Hong Kong (Yuen et al. 2015), Israel (Lerner et al. 2019), Italy (Aucella et al. 2000; Casini et al. 2017, 2018a, 2018b, 2019; Mosci et al. 2017), Spain (Simon Garcia et al. 2009), Taiwan (Ho et al. 2016a; Huang et al. 2015), the UK (Patel et al. 2007; Wilcox et al. 2003), and the USA (Alhmidi et al. 2017; Anderson et al. 2017, 2018; Coppin et al. 2017; Deshpande et al. 2014; Eckstein et al. 2007; Ghantoji et al. 2015; Hacek et al. 2010a; Jinadatha et al. 2014; Kaatz et al. 1988; Manian et al. 2011, 2013a; Orenstein et al. 2011; Rathod et al. 2019; Rutala et al. 2018; Zhang et al. 2013). There were two studies from upper-middle income countries including Indonesia (Styaningsih et al. 2019) and Mexico (Galván Contreras et al. 2017). One study was from a low income country in Sierra Leone (Youkee et al. 2015). Among the most commonly sampled environmental surfaces were bedrails, bedside tables, call buttons, floors, and other high-touch surfaces. The surfaces were in critical care settings such as a burns center, operating rooms, veterans affairs hospital, and patient isolation rooms with known CRAB, *C. difficile*, MRSA, or VRE colonization.

The efficacy of sodium hypochlorite disinfectants in reducing bacterial load or percent surfaces positive are summarized by outcome: gram-positive organisms (bacilli and cocci), gram-negative organisms, fungi, all viable organisms, and HAIs.

Organism	Not effective			Unclear					Effective							
All viable organisms	1079	128	*280	7960	129	131	1416	10553	*2323	1311	ǂ130	3666	1886	6931	3507	6163
Gram positive cocci	*10160	*10553	6931			6163	2926			14130	899	*2323	6931	7047	ǂ1416	
Gram positive bacilli		1171	*10553		130	2926	3854				899	14394	686	1886	7047	
Gram-negative bacteria					130	1574	6931				14130	*3147	6163	*10553		
Virus						3432										
HAI	*5113	*6885	*6887			14850				*5852	3854	*6885	*6887	7960	8042	

Figure S4. Harvest plot of manually applied sodium hypochlorite disinfection interventions with rows identifying outcome pathogen or HAI, columns representing effect (not effective = disinfectant was significantly less effective or not significantly different; unclear = intervention significance or confidence intervals not specified; effective = at least one metric with significant reduction or any metric >90% or > 1 log10 reduction). ǂ denotes that study was defined as effective due to any metric reported > 90% or > 1 log10 reduction. Higher bar height represents better study design (three = controlled crossover; two = cohort or controlled before-after; one = no simultaneous control). Color represents outcome metric (black = concentration; grey = percent surfaces; white=ATP or qualitative). The number identifies the study ID (see table D1 for complete reference). * denotes the disinfectant was compared to another disinfectant rather than comparing before and after disinfection.

<u>Gram-positive organisms</u>

There were 18 studies assessing efficacy of manually-applied sodium hypochlorite on gram-positive organisms including *C. difficile*, *Bacillus subtilis*, VRE, *S. epidermis*, *S. aureus*, MRSA

There were 12 studies that assessed gram-positive bacilli. When compared to before disinfection with sodium hypochlorite, there were significant reductions in *C. difficile* concentration by 79% (initial mean = 5.1 CFU) (Kaatz et al. 1988), and percent surfaces positive by 90% (initial=50% positive) (Deshpande et al. 2014), 85% (initial=20% positive) (Mosci et al. 2017), and 50% (initial=24% positive) (Barbut et al. 2009). One study found significantly lower percent rooms positive for *C. difficile* after two rounds of bleach disinfection (Eckstein et al. 2007). Three studies did not assess significance with one study finding a 75% reduction after disinfection among four surfaces positive for *C. difficile* (Casini et al. 2019). In a crossover trial comparing detergent to sodium hypochlorite in two wards, significance was not assessed for percent surfaces positive for *C. difficile* and there were not evident reductions in both wards (Wilcox et al. 2003). One study reported a non-significant 70% reduction in *C. difficile* count (initial=2.39 CFU) (Ghantoji et al. 2015). One study reported no growth of *Bacillus* spp. after disinfection (initial = 4.00 CFU/cm^2) (Styaningsih et al. 2019). When comparing sodium hypochlorite to QACs, one study found that sodium hypochlorite significantly reduced percent surfaces positive while a QAC did not (Deshpande et al. 2014). Another study found that *C. difficile* concentration (mean=3.76 CFU) was not significantly different than when using a QAC, although initial concentration was not assessed (Rutala et al. 2018). When disinfection was randomized to HPV or sodium hypochlorite, while both studies found fewer samples positive for *C. difficile* in HPV rooms, one study found that disinfectant efficacy was not significantly different (Mosci et al. 2017) and a second study found that that HPV had significantly higher reductions in percent surfaces positive than sodium hypochlorite (Barbut et al. 2009).

There were 10 studies that assessed gram-positive cocci. After disinfection there was a 91% reduction (initial medan=127 CFU) in MRSA concentration (Jinadatha et al. 2014), significantly lower number of sites positive for MRSA on 3 of 16 surface types (Patel et al. 2007) and at least 92% reduction in percent surfaces positive for MRSA or VRE (initial=24% positive) (Deshpande et al. 2014). Compared to control surfaces there were significantly fewer surfaces positive for MRSA when using sodium hypochloirite (Alhmidi et al. 2017). After four rounds of bleach disinfection, 2% samples were positive for MRSA (Manian et al. 2011). The addition of bleach to disinfection with QAC significantly reduced percent surfaces positive for VRE (Eckstein et al. 2007). Four studies did not find significant effects from sodium hypochlorite finding no samples positive for *S. aureus* (initial mean = 7.00 CFU/cm^2) (Styaningsih et al. 2019), low median concentration of *S. aureus* (initial median=1.1 CFU/cm^2) (Ho et al. 2016a), low median concentration of VRE (initial median=0.98 CFU/cm^2) (Ho et al. 2016a), and no samples positive for *S. epidermidis* (initial mean = 8.33 CFU/cm^2) (Styaningsih et al. 2019) after sodium hypochlorite disinfection. While not significant (p=0.07), there was reduced percent surfaces positive for VRE after sodium hypochlorite disinfection compared to control surfaces (Alhmidi et al. 2017). When sodium hypochlorite was compared to a QAC disinfectant, sodium hypochlorite significantly reduced recovery of MRSA and/or VRE (Deshpande et al. 2014), concentration of VRE (mean=2.43 CFU) (Rutala et al. 2018), and concentration of MRSA (mean=4.39 CFU) (Rutala et al. 2018). Mean staphylococcal contamination increased

significantly by 80% 5 h after disinfection with sodium hypochlorite while it decreased significantly with the application of a QAC spray (Yuen et al. 2015). There was not a significant difference in percent surfaces positive for MRSA when disinfecting with 30% ethanol spray or sodium hypochlorite (Alhmidi et al. 2017).

Gram-negative organisms

There were 7 studies assessing efficacy of manually-applied sodium hypochlorite on gram-negative organisms including MDR *Acinetobacter* spp. including CRAB and *A. baumannii* complex, ESBL-producing *Enterobacteriaceae*, *K. pneumonia*, and gram-negative bacilli. Median concentration of CRAB was significantly lower after disinfection compared to before disinfction (initial median=102 CFU) (Ho et al. 2016a), 85% reduction in percent surfaces positive for CRAB after disinfection (initial=41% surfaces positive, significance not specified) (Lerner et al. 2019), and 87% reduction in percent surfaces positive for gram-negative bacilli (initial=4.5% surfaces positive) (Alhmidi et al. 2017). Of three surfaces positive for ESBL K. pneumoni, one surface remained positive after disinfection with sodium hypochlorite (Casini et al. 2019) (Casini 2019). There was not a significant difference between sodium hypochlorite and 30% ethanol spray in reducing percent surfaces positive for gram-negative bacilli (Alhmidi et al. 2017). Compared to QAC, bleach was significantly more effective at reducing mean concentration per room of MDR Acinetobacter spp. (Rutala et al. 2018), however another study found that although there was a significant reduction in percent surfaces positive for *A. baumannii* complex, there were still 16% of surfaces positive after four rounds of bleach disinfection (Manian et al. 2011). There were more sinks positive for ESBL Enterobacteriaceae when using QAC compared to using bleach (Roux et al. 2013).

Fungi

There were no studies assessing efficacy of manually-applied sodium hypochlorite on fungi.

Virus

One study assessed Ebola virus RNA finding a 34% reduction in percent surfaces positive after deep cleaning with a disinfectant identified as reconstituted sodium hypochlorite from powder (initial=29% surfaces positive) (Youkee et al. 2015). One study assessed viruses as an HAI-outcome but did not take environmental samples (see HAI section below, (Aucella et al. 2000)).

All viable organisms

There were 15 studies assessing efficacy of manually-applied sodium hypochlorite on all viable organisms.

Most studies found reductions following disinfection with with sodium hypochlorite. There was a significant 99% reduction in total bacterial count (initial=160 CFU)(Zhang et al. 2013), 98% reduction in bacteria (concentration and significance not reported) (Rathod et al. 2019), 86-92% reduction in mean bacterial count (initial range= 7-58 CFU/24 cm^2) (Casini et al. 2019), 76%

reduction in mean concentration (initial=255 CFU) (Jinadatha et al. 2014), 38-59% reduction in mean bacterial count initial range = 284-995 CFU) (Casini et al. 2018b), and significant reduction in median count (initial=0.25 CFU/cm^2)(Huang et al. 2015) compared to before disinfection.

Studies also found reductions in percent rooms positive for bacteria (initial=11 rooms positive) (Mosci et al. 2017) and percent samples with presence of pathogenic organism(Simon Garcia et al. 2009) compared to before disinfection. Compared to control surfaces, surfaces disinfected with sodium hypochlorite had fewer surfaces positive for organisms (Alhmidi et al. 2017). Some surfaces had significant reductions on some surface types but not others. Two of eleven surface types had significantly lower concentration after disinfection (Ho et al. 2016a) and in another study, 13% of sites remained above unacceptable threshold of 20 CFU/100 cm^2 (Casini et al. 2017). Two-step disinfection with sodium hypochlorite did not significantly reduce concentration compared to baseline (Casini et al. 2018a) and sodium hypochlorite did not reduce percent surfaces positive after disinfection in one study (Galván Contreras et al. 2017)

Detergent with sodium hypochlorite had significantly lower total counts compared with detergent alone (Patel et al. 2007). Concentration of MDROs was 81% lower when using sodium hypochlorite at 12 CFU/room compared to using a QAC (Rutala et al. 2018). Compard to other disinfectants, there was no significant difference between copper surfaces and control surfaces cleaned with sodium hypochlorite up to 6 h after disinfection, however copper surfaces had lower concentrations between 24-30 h after disinfection (Coppin et al. 2017). Sodium hypochlorite was not significantly different than BCDMH (Galván Contreras et al. 2017), 30% ethanol (Alhmidi et al. 2017), or hydrogen peroxide vapor (Mosci et al. 2017) in percent surfaces positive.

<u>HAIs</u>

Eight studies assessed the effect of sodium hypochlorite disinfection on HAI outcomes due to *C. difficile*, MRSA, MDR-*Acinetobacter* spp., VRE, Hepatitis C, and other pathogens.

In a multi-site 2-year crossover trial compring standard disinfection with a QAC to bleach disinfection, hospital-wide HAI incidence was not significantly different for VRE, MRSA, or all target pathogens combined (Anderson et al. 2018). Hospital-wide HAI incidence was significantly lower during the bleach period for MDR *Acinetobacter* spp. (Anderson et al. 2018). Among exposed patients to a prior room occupant with target pathogen, incidence was not significantly different for MRSA, MDR *Acinetobacter* spp., or all target pathogens combined (Anderson et al. 2017). However, exposed patient incicence was signicantly lower during the bleach period for VRE (Anderson et al. 2017). An uncontrolled before-after study found that the intervention period using bleach and QAC disinfection had significantly lower incidence of nosocomial infection compared to the pre-intervention period (Simon Garcia et al. 2009).

Four studies specifically assessed *C. difficile* infections. In a single-site crossover trial comparing detergent cleaning to bleach, *C. difficile* infections significantly decreased in one of two wards that used bleach compared to using detergent (Wilcox et al. 2003). *C. difficile* infections were significantly associated with percent surfaces positive for *C. difficile* (Wilcox et

al. 2003). In two uncontrolled before-after study, following a pre-intervention period using QAC for terminal disinfection, there was a significant reduction in *C. difficile* infections compared to the intervention period using bleach (Hacek et al. 2010b; Orenstein et al. 2011). In an uncontrolled before-after study, a two-year pre-intervention period using bleach for terminal disinfection had significantly higher *C. difficile* infetctions compared to a one-year intervention period with bleach and HPV terminal disinfection (Manian et al. 2013a).

One study assessed hepatitis C HAIs at four dialysis centers over five years to determine whether disinfection of shared monitors or monitor separation prevented hepatitis C positivity in patients (Aucella et al. 2000). Compared to three years of historical data without disinfection use, prevalence of hepatitis C virus infection and incidence reduced after implementing disinfection, though significance not reported (Aucella et al. 2000).

Other Chlorine

In this report, other chlorine disinfectants include demand-release chlorine such as sodium dichloroisocyanurate (NaDCC), chloramine, chlorine dioxide, and bromo-chloro-dimethyl-hydantoin (BCDMH). Other chlorine disinfectants include electrolyzed water. For the purposes of this report, if a chlorine-based disinfectant was not identified as bleach or sodium hypochlorite, the disinfectant was considered other chlorine.

<u>Results</u>

This report includes reviews of a total of 25 articles covering interventions involving NaDCC (Best et al. 2014; Casey et al. 2010; Doan et al. 2012; Frabetti et al. 2009; Garvey et al. 2016; Goldenberg et al. 2012; Hall et al. 2011; Ho et al. 2016a; Hosein et al. 2016a; Karpanen et al. 2012; Ojajärvi and Mäkelä 1976; Oztoprak et al. 2019; Shelly et al. 2011), chlorine dioxide (Allen et al. 2019; Goldenberg et al. 2012; Johnson et al. 2016; Jones et al. 2015), chloramine (Andersen et al. 2006a), BCDMH (Galván Contreras et al. 2017), calcium hypochlorite (Turner et al. 1974), and electrolyzed water (Stewart et al. 2014). Though less specific, other interventions included unspecified hypochlorite and unspecified chlorine disinfectants (Al-Hamad and Maxwell 2008; Chen et al. 2017; Gan et al. 2017; Mayfield et al. 2019; Siani et al. 2018).

The critical care settings in which the interventions were assessed across these studies included acute medical wards, intensive care units, a cystic fibrosis outpatient clinic, a chronic infection outpatient clinic, a rehabilitation unit, surgical wards, a cardiovascular ward, wards with elderly care patients, an infectious disease ward, a neonatal intensive care unit (NICU), and an emergency department. Twenty-three of the studies were conducted in countries with high-income economies in Australia (Chan et al. 2011), Finland (Ojajärvi and Mäkelä 1976), Ireland (Shelly et al. 2011), Italy (Frabetti et al. 2009), Norway (Andersen et al. 2006a), Taiwan (Chen et al. 2017; Ho et al. 2016a), the UK (Al-Hamad and Maxwell 2008; Allen et al. 2019; Best et al. 2014; Casey et al. 2010; Doan et al. 2012; Garvey et al. 2016; Goldenberg et al. 2012; Hall et al. 2011; Hosein et al. 2016b; Johnson et al. 2016; Jones et al. 2015; Karpanen et al. 2012; Siani et al. 2018; Stewart et al. 2014), and the USA (Mayfield et al. 2019; Turner et al. 1974). Three studies were in the upper-middle income countries in China (Gan et al. 2017), Mexico (Galván Contreras et al. 2017) and Turkey (Oztoprak et al. 2019).

The setting included patient rooms, operating rooms, patient washrooms, isolation rooms, and samples from the open ward. Specific surfaces on which sampling occurred generally included bedrails or bedside tables, handles, call buttons, floors, walls, and other high-touch surfaces within the patient environment.

The efficacy of chlorine-based disinfections, excepting sodium hypochlorite, on reducing bacterial load or percent surfaces positive are summarized by outcome: gram-positive organisms (bacilli and cocci), gram-negative organisms, all viable organisms, and HAIs. None of the studies assessed fungi or viruses following disinfectant interventions.

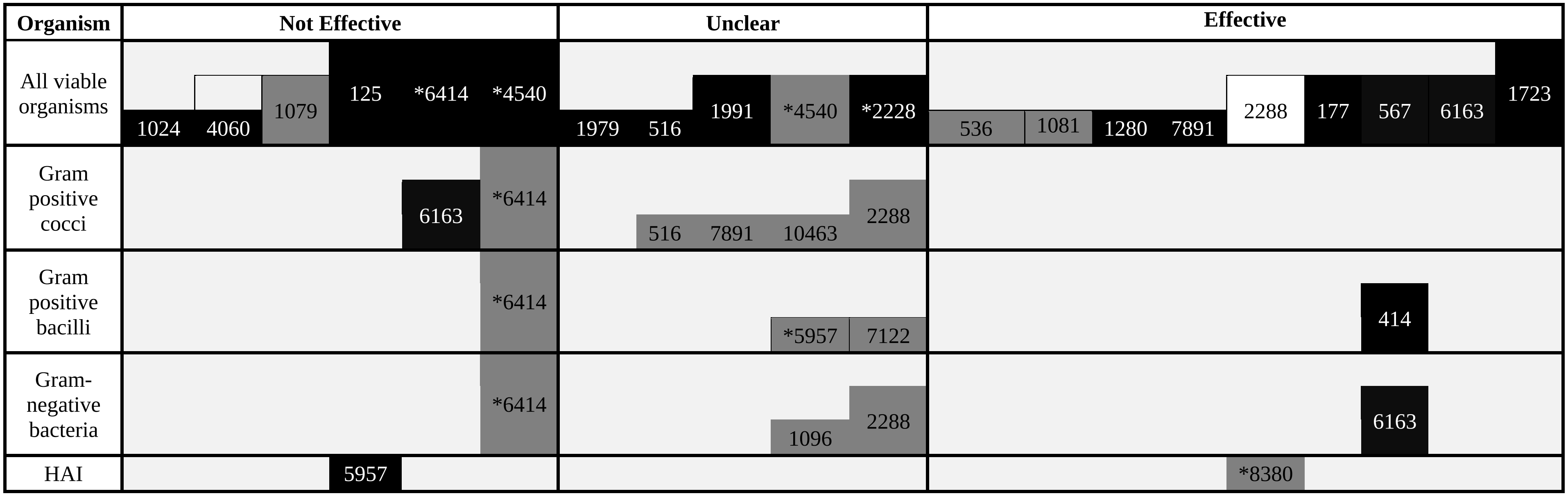

Figure S5. Harvest plot of manually applied chlorine-based disinfection interventions (excepting sodium hypochlorite) with rows identifying outcome pathogen or HAI, columns representing effect (not effective = disinfectant was significantly less effective or not significantly different; unclear = intervention significance or confidence intervals not specified; effective = at least one metric with significant reduction or any metric >90% or > 1 log10 reduction). ǂ denotes that study was defined as effective due to any metric reported > 90% or > 1 log10 reduction. Higher bar height represents better study design (three = controlled crossover; two = cohort or controlled before-after; one = no simultaneous control). Color represents outcome metric (black = concentration; grey = percent surfaces; white =ATP or qualitative). The number identifies the study ID (see table D1 for complete reference). * denotes the disinfectant was compared to another disinfectant rather than comparing before and after disinfection.

Gram-positive organisms

Nine studies included findings from NaDCC, chlorine dioxide, electrolyzed water, and unspecified hypochlorite disinfectant efficacy against gram-positive organisms including *Clostridium difficile*, MSSA, MRSA, and VRE.

Four studies assessed efficacy on gram-positive bacilli. All four studies assessed efficacy of NaDCC and one study assessed chlorine-dioxide on gram-positive bacilli on environmental surfaces for *C. difficile*. One study (Doan et al. 2012) found significant reductions in concentration of inoculated *C. difficile* spores onto environmental surfaces after disinfection (standardized median log10 reduction= 2.301 CFU/mL, IQR= 0.935, 2.301) (Doan et al. 2012). This study assessed eight disinfectant interventions and NaDCC was the second-most effective of eight interventions assessed (Doan et al. 2012). A second study found a 43% reduction in percent surfaces positive for *C. difficile* (10.8% before, 6.1% after, significance not assessed) after a seven-day deep clean with NaDCC (Best et al. 2014). In a crossover trial, there was not a significant difference in percent surfaces positive for *C. difficile* for copper and standard surfaces disinfected with 1000 ppm NaDCC (Karpanen et al. 2012). Chlorine dioxide was not more effective (significance not specified) than standard disinfection with NaDCC demand-release chlorine in reducing percent of surfaces positive (compared to standard disinfection = 9/120 surfaces positive) (Goldenberg et al. 2012).

Six studies assessed efficacy of NaDCC, electrolyzed water, and unspecified chlorine on gram-positive cocci including MRSA, MSSA, and VRE. While some studies found reductions after disinfection, no studies found significant reductions after disinfection with other chlorine-based disinfectants. Daily NaDCC disinfection had a non-significant reduction in MRSA count before (median= 0.9 CFU/cm^2) compared to after disinfection (median=0 CFU/cm^2) (Ho et al. 2016b). After recovery of MRSA (concentration not specified) from the bore of an MRI unit, MRSA was not recovered after disinfection with 1000 ppm NaDCC (Shelly et al. 2011). Electrolyzed water disinfection had 48% reduction in percent surfaces positive for MRSA and at 1 h after disinfection and up to 71% reduction at 4 h after disinfection (Stewart et al. 2014). MRSA was not recovered (Oztoprak et al. 2019) or infrequently recovered (Al-Hamad and Maxwell 2008) after unspecified hypochlorite disinfection. A crossover trial comparing copper and standard surfaces both disinfected with 1000 ppm NaDCC found similar percent surfaces positive for MRSA and significantly higher percent surfaces positive for MSSA on standard surfaces compared to copper surfaces (Karpanen et al. 2012).

For VRE, NaDCC disinfection had a non-significant reduction in median VRE concentration after disinfection (not detected) compared to before disinfection (median=1.5 CFU/100 cm^2) (Ho et al. 2016b). VRE was not recovered (Oztoprak et al. 2019) after unspecified hypochlorite disinfection. A crossover trial comparing copper and standard surfaces both disinfected with 1000 ppm NaDCC found significantly higher percent surfaces positive for MSSA on standard surfaces compared to copper surfaces (Karpanen et al. 2012).

Gram-negative organisms

Four studies assessed the effect of NaDCC on coliforms, carbapenamase-producing coliforms, multi-drug resistant *A. baumannii*, and carbapenem-resistant *P. aeruginosa*. Only one study found significant reductions, finding a ~94% reduction in median concentration of CRAB after disinfection with NaDCC compared to before disinfection (median = 8.5 CFU/cm^2) (Ho et al. 2016b). Other studies found unclear effects. After disinfection with unspecified hypochlorite, carbapenem-resistant *P. aeruginosa* and MDR *A. baumannii* were not recovered (initial concentration not reported) (Oztoprak). After terminal disinfection with 1000 ppm NaDCC and 6% HPV, some surfaces remained positive for carbapenemase-producing coliforms, however these organisms were not recovered after increasing concentrations of NaDCC to 2000 ppm and 12% HPV (Garvey et al. 2016). A crossover trial comparing copper and standard surfaces both disinfected with 1000 ppm NaDCC found significantly higher percent surfaces positive for coliforms on standard surfaces compared to copper surfaces (Karpanen et al. 2012).

Fungi

No studies assessed effects of non-sodium hypochlorite chlorine interventions on fungi.

Virus

No studies assessed effects of non-sodium hypochlorite chlorine interventions on viruses.

All viable organisms

There were 21 studies that assessed efficacy of non-sodium hypochlorite chlorine interventions on all viable organisms, which included MDROs. Seven studies reported significant reductions in bacterial concentration in at least one type of surface. Four (Hall et al. 2011; Ho et al. 2016b; Hosein et al. 2016a; Oztoprak et al. 2019) of the eight studies assessing NaDCC disinfection found significant reductions in at least one surface type. One (Allen et al. 2019) of the three studies involving chlorine dioxide found significant reductions. Other studies that found significant reductions involved chloramine (Andersen, 2006), electrolyzed water (Stewart et al. 2014), and unspecified hypochlorite (Chen et al. 2017; Gan et al. 2017).

Eight studies assessed all viable organisms following NaDCC disinfection. In one crossover study, there was a significant median reduction in bacterial concentration of ~48% (initial count=100 CFU) across surfaces following NaDCC disinfection (Hall et al. 2011). Another controlled before-after study found significant reductions in median bacterial concentration after NaDCC disinfection compared to before in three of eleven surfaces studied (range of initial median= 0.5-28.6 CFU/cm^2) (Ho et al. 2016b) and significant reductions of 97% in ATP (initial =651 RLU) (Oztoprak et al. 2019). There was a significant reduction in mean bacterial count of 61% (initial mean= 19.5 CFU) (Hosein et al. 2016b). Four studies (Casey et al. 2010; Frabetti et al. 2009; Karpanen et al. 2012; Ojajärvi and Mäkelä 1976) did not find significant reductions. In one crossover trial of copper and control surfaces, there was no significant difference in microbial count on control items before daily cleaning with NaDCC and detergent compared to after (median before=3.6 vs. after=2.1 CFU/cm^2, p=0.97). However, most of the copper surfaces treated with the same daily cleaning had significantly lower bacterial concentration (Casey et al. 2010). One uncontrolled before-after study found insignificant reductions in bacterial

concentration (range=8.3%-79.6%) compared to initial loads (range of initial mean=1.5-5.98 CFU/cm^2) (Frabetti et al. 2009). In a cohort study, initial burden was not measured, however reported mean bacterial concentration was 39% lower at 17 CFU/plate following NaDCC disinfection compared to phenol-based disinfection (significance not assessed) (Ojajärvi and Mäkelä 1976). A crossover trial comparing copper and standard surfaces both disinfected with 1000 ppm NaDCC found significantly higher total bacterial counts on standard materials compared to copper (largest median difference =80.3 CFU/cm^2, initial concentration= 110 CFU/cm^2) (Karpanen et al. 2012).

Three studies assessed all viable organisms following chlorine dioxide. There was a significant reduction in an uncontrolled study up to 80% in mean bacterial count following chlorine dioxide across five surface types (range of initial load=30-250 CFU) (Allen et al. 2019). The second study was a controlled cohort study which reported high baseline concentration (initial median= >500 CFU) and low bacterial count immediately after chlorine-dioxide disinfection (median=2.54 CFU) and after 24 h (median=7.5 CFU) compared to before (Jones et al. 2015). Chlorhexidine gluconate disinfectant was significantly more effective than chlorine dioxide at all time points throughout the study (Jones et al. 2015). Finally, one uncontrolled study found a 30% reduction in total count following disinfection (initial total count =27.4 CFU) (Johnson et al. 2016).

One controlled before-after study assessed all viable organisms after chloramine disinfection finding significant reductions up of ~96% in mean bacterial concentration (initial mean= 30.9 CFU) after chloramine disinfection and ~87% reduction (initial mean=30.9 CFU) after chloramine disinfection with rinse (Andersen et al. 2006a).

One study assessed all viable organisms after disinfection with electrolyzed water. There was a significant reduction in mean bacterial concentration of ~62% (initial mean= 4.3 CFU/cm^2) after 1 h from disinfection compared to before (Stewart et al. 2014).

One controlled before-after study assessed all viable organisms following bromo-chloro-dimethyl-hydantoin (BCDMH) disinfection. Following disinfection, there were no surfaces positive for bacterial burden compared to before (initial=13/21 surfaces positive), although results did not reach significance (Galván Contreras et al. 2017). Additionally, there was no significant difference in reductions of percent surfaces positive between BCDMH and sodium hypochlorite (Galván Contreras et al. 2017)

In a burns unit immersion tub, one uncontrolled study found that manual scrubbing had significant reductions in total bacterial counts (initial mean=372 CFU) but that following cleaning with calcium hypochlorite disinfection had non-significant reduction of 50% in bacterial counts (initial mean= 22 CFU) (Turner et al. 1974).

Four studies had unspecified chlorine disinfectants. One crossover trial on two wards assessed peracetic acid/hydrogen peroxide wipes compared to detergent followed by chlorine-containing disinfectant of known chlorine concentration (but unknown active ingredients) on all viable organisms and MDROs (VRE, CRE, and/or ESBL) (Siani et al. 2018). There were significantly lower reductions in aerobic organism count when using detergent followed by chlorine compared

to peracetic acid/hydrogen peroxide wipes during both periods of the crossover trial. The total anaerobic count and ATP were not significantly different for one ward, but significantly higher in another ward with the use of detergent and chlorine compared to the use of peracetic acid/hydrogen peroxide wipes. Percent samples positive for MDROs was higher (significance not specified) on wards using detergent and chlorine (3% and 1.3%) compared to wards using peracetic acid/hydrogen peroxide wipes (1% and 0.6%) (Siani et al. 2018). After disinfection with an unspecified hypochlorite, there were reductions in bacterial concentration (initial mean= 7.5 CFU/cm^2) though significance was not specified (Al-Hamad and Maxwell 2008) and significant reduction of 83% in percent surfaces positive for MDROs (Gan et al. 2017). One study assessed gene abundance and bacterial species diversity finding that a ward disinfected daily with 500 ppm unspecified hypochlorite had lower species diversity and (sometimes) significantly lower gene abundance when compared to a ward only conducting terminal disinfection (Chen et al. 2017).

<u>HAIs</u>

Two studies assessed HAI outcomes associated with other chlorine-based disinfection interventions. A pre-intervention period with standard cleaning with NaDCC demand-release chlorine compared to intervention period with chlorine-dioxide-based disinfection did not have significantly different incidence of *C. difficile* infections (pre-intervention incidence=11.8) or significantly different rate of *C. difficile* infection (pre-intervention rate= 0.42 per 1000 occupied bed-days) (Goldenberg et al. 2012). Among bone marrow transplant patients (n=293), *C. difficile*-associated disease (CDAD) incident rate decreased significantly from 8.6 to 3.3 cases per 1000 patient days from a period using QAC to a period using unspecified hypochlorite solution, though CDAD rates were not significantly different in other units (Mayfield et al. 2019). The CDAD rate for bone marrow transplant patients increased to 8.1 cases from 3.2 cases per 1000 patient –days after replacing hypochlorite with quaternary ammonium disinfectant (Mayfield et al. 2019).

Other Manually Applied

There is a variety of other manually applied not categorized above as alcohol, peroxygen, sodium hypochlorite, or other chlorine.

Results

There were 18 studies that assessed the efficacy of other manually-applied chemical disinfectants were reviewed. We identified manually-applied disinfectants comprised of phenols (Biswal et al. 2017; Danforth et al. 1987; Dunklin and Lester 1959; Gable 1966; Ojajärvi and Mäkelä 1976; Smith et al. 1998; Stibich et al. 2011; Tekin et al. 2013), hydrochlorides(Fujii 1996; Hedin et al. 2010; Oie et al. 2005), aldehydes (Daschner et al. 1980; Exner et al. 1982; Meinke et al. 2012), copper (Hall et al. 2011; Hamilton et al. 2010), triethylene glycol (Strat 1971), and grapefruit seed extract (Ogino et al. 1995). Many of the samples collected in these studies were from ICUs, transplant units, surgical recovery wards, outpatient clinics and operating rooms in larger hospitals, no matter the geographic location. The majority of the studies were in high-income countries, including Canada (Danforth et al. 1987), Finland (Ojajärvi and Mäkelä 1976), Germany (Daschner et al. 1980; Exner et al. 1982), Japan (Fujii 1996; Ogino et al. 1995; Oie et al. 2005), Romania (Strat 1971), Sweden (Hedin et al. 2010), Switzerland (Meinke et al. 2012), the UK (Hall et al. 2011; Hamilton et al. 2010), and the USA (Biswal et al. 2017; Dunklin and Lester 1959; Gable 1966; Smith et al. 1998; Stibich et al. 2011; Tekin et al. 2013). One study was from an upper-middle income country in Turkey (Tekin et al. 2013) and one study was in a lower-middle income country in India (Biswal et al. 2017). Specifically, samples were collected from floors, tables, beds, windows and any other furniture or fixture in the rooms.

The efficacy of all other manually applied disinfectants in reducing bacterial load or percent surfaces positive are summarized by outcome: gram-positive organisms (bacilli and cocci), gram-negative organisms, fungi, all viable organisms, and HAIs.

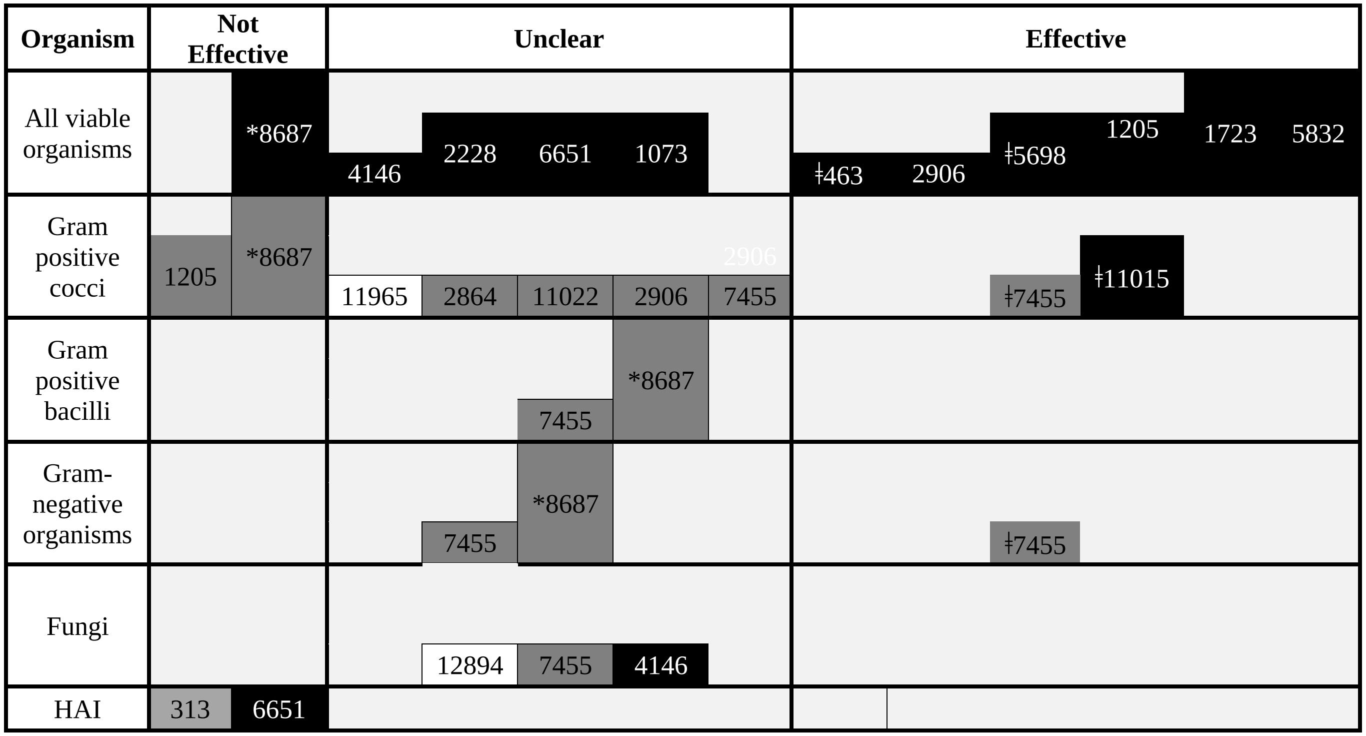

Figure S6. Harvest plot of all other manually applied disinfection interventions with rows identifying outcome pathogen or HAI, columns representing effect (not effective = disinfectant was significantly less effective or not significantly different; unclear = intervention significance or confidence intervals not specified; effective = at least one metric with significant reduction or any metric >90% or > 1 log10 reduction). ǂ denotes that study was defined as effective due to any metric reported > 90% or > 1 log10 reduction. Higher bar height represents better study design (three = controlled crossover; two = cohort or controlled before-after; one = no simultaneous control). Color represents outcome metric (black = concentration; grey = percent surfaces; white = ATP or qualitative). The number identifies the study ID (see table D1 for complete reference). * denotes the disinfectant was compared to another disinfectant rather than comparing before and after disinfection.

Gram-positive organisms

Nine studies included findings on efficacy of manually applied phenol, aldehyde, hydrochloride and other disinfectants against gram-positive organisms including *Clostridium difficile,* spore-forming organisms, *Staphylococcus aureus*, MRSA, *Enterococcus* spp., VRE, and green streptococci.

Two studies assessed gram-positive bacilli. *C. difficile* was not detected in one crossover trial comparing aldehyde-based and glucoprotamin-based disinfection (Meinke et al. 2012). Spore-forming bacteria were still detected in 15-25% surfaces after disinfection (initial=19-41% positive) with aldehyde-based disinfectant (Exner et al. 1982).

Eight studies assessed gram-positive cocci.

While no studies assessed significance for efficacy against *Staphylococcus* spp., there were two studies that showed high reductions following disinfection. Disinfection with 0.2% solution of alkyldiaminoethyl glycine for ten minutes had up to 99.99% reduction in methicillin-sensitive *S. aureus* (MSSA) count (range of initial mean=6.5-13897 CFU) and MRSA count (range of initial mean= 48 – 7366 CFU) with no detection of MSSA or MRSA after disinfection (Oie et al. 2005). Though significance was not assessed, there was up to a 91% reduction in surfaces positive for *S. aureus* (initial=56% positive, n=40/71) after disinfection with aldehyde-based disinfectant (Exner et al. 1982) and a 67% reduction in surfaces positive for MRSA after disinfection with grapefruit seed extract (initial=75% positive). MRSA was detected following disinfection with alkyldiaminioethyl glycine, 0.2% chlorhexidine digluconate, and 0.2% benzalkonium chloride but MRSA was not detected when concentration was increased to 0.5% benzalkonium chloride and 0.5% chlorhexidine digluconate (Fujii 1996). A crossover study compared two disinfectants (aldehyde-based and glucoprotamin-based) but found low prevalence of *S. aureus* for both (Meinke et al. 2012).

There were no studies that found significant reductions in *Enterococcus* spp. or other cocci due to other manually applied disinfectants. In one crossover trial, there was not a significant difference in percent surfaces positive for *Enterococcus* after disinfection with an aldehyde-based or glucoprotamin-based disinfectant (Meinke et al. 2012). There was no significant difference in surfaces positive for *Enterococcus faecalis* on surfaces sprayed daily with polyhexamethylene biguanide compared to control surfaces (Hedin et al. 2010). For VRE, there was a 76% reduction in percent surfaces positive (initial=23%) after disinfection with a phenol-based disinfectant in one study (significance not specified) (Stibich et al. 2011) and after deep cleaning with phenol-based disinfectant VRE was not recovered from two rooms positive for VRE (Smith et al. 1998). There was low to no recovery for green *Streptococcus* (Exner et al. 1982) and VRE (Meinke et al. 2012) in some studies.

Gram-negative organisms

Two studies assessed the efficacy of manually applied aldehyde disinfectants against gram-negative bacteria, including *Citrobacter* spp., *Enterbacteriaceae*, *Proteus* spp., and others. One crossover study tested for gram-negative bacteria but had low prevalence throughout the study

(Meinke et al. 2012). A second study did not recover some gram-negative bacteria immediately after disinfection for *Klebsiella* spp. (initial=11%-35%), *E. coli* (initial=8-14%), *Citrobacter* spp. (initial=1%), *Proteus* spp. (initial=2%), and *P. aeruginosa* (initial=2.5%) but still had recovery of *Enterobacter* spp. (initial=3-14%) after disinfection (Exner et al. 1982).

Fungi

Three studies assessed efficacy of manually applied phenol or aldehyde disinfectants on fungi including *Candida auris.* All three studies did not have a contemporary control and compared efficacy to initial measurements before disinfection. There was an 86% reduction in average concentration of fungi after disinfection with 20% orthophenylphenol (initial mean= 2.9 CFU/cm^2) though significance was not specified (Tekin et al. 2013). *Candida auris* was present on surfaces after cleaning with a 5% phenol solution up to four days after cleaning (Biswal et al. 2017). Yeast were not recovered immediately following disinfection with an aldehyde-based disinfectant (initial=2-14% positive) (Exner et al. 1982).

Virus

No studies assessed effects of other manually applied disinfectant interventions on viruses.

All viable organisms

Eleven studies assessed the efficacy of other manually applied disinfectants against all viable bacteria on environmental surfaces.

All but one study found that when compared to a non-disinfectant control, disinfectants had reductions in bacterial concentrations. Two crossover studies found that a **copper biocide** was effective against all viable bacteria. The disinfection with copper biocide using ultramicrofiber mop had significant reduction in median concentration (initial= 78 CFU) after disinfection while cleaning with an ultramicrofibre mop with water alone was not effective (Hall et al. 2011). Another crossover study found significant reductions in bacterial count after cleaning with ultramicrofibre mops and water (reduction=30%) as well as after using copper biocide (reduction=56%), however the median bacterial count was significantly lower with the copper biocide compared to water alone one and four hours after disinfection (Hamilton et al. 2010). Bedside tables sprayed daily with **polyhexamethylene biguanide** (PHMB) had significantly lower median concentration (0-9 CFU/50 cm^2) than surfaces not sprayed with PHMB (20-22 CFU/50 cm^2) (Hedin et al. 2010). Disinfection with aerosolized hydrochloride solution followed by wiping with **hydrochloride wipes** (dodecyldiaminoethylglycine) was 22 times more effective after 1.5 h and 9.8 times more effective after 12.5 h than cleaning with water and soda alone disinfectant (Strat 1971). Bacterial reductions on floors ranged between 70-96% after disinfection with **phenol-based** disinfectant (2.5% orthophenylphenol, range=423-12,280 CFU/ft^2) compared to cleaning periods only using detergent (range=11,100-169,800 CFU/ft^2) (Dunklin and Lester 1959). Floor disinfection using 1% **aldehyde disinfectant** had reductions for four different methods of application of 84% using a two-bucket system (initial=44,200 CFU/m2), by 60% using a disposable wet wipe (initial=30,700 CFU/m2), by 55% using rotating disc blockers (initial=7,800 CFU/m2), and by 50% using a dry mop (initial=15,800 CFU/m^2)

when compared to no disinfection (significance not specified) (Daschner et al. 1980). One study did not find bacterial reductions after cleaning with a general detergent or when disinfecting floors with **a phenolic detergent-disinfectant** (initial range=36-210 CFU) (Gable 1966). When comparing to before disinfection, two studies found reductions in bacterial concentration when using phenol-based disinfectants. A **phenol-based** disinfectant (ortho-phenylphenol 3.4%, ortho-benzyl-para-chlorophenol 3.03%) had significant 18% reduction in mean concentration (initial=33 CFU/cm^2) after compared to before disinfection (Stibich et al. 2011). A **phenol-based** disinfectant (20% orthophenylphenol) had lower average concentration of bacteria by 87% after compared to before disinfection (initial=12.1 CFU/cm^2) though significance was not assessed (Tekin et al. 2013).

A few studies explicitly compared disinfectant interventions. One cohort study found that disinfection with **phenol-based** (0.5-1% arylated and halogenated phenols) disinfectant had higher average concentration of bacteria on patient floors (28 CFU/plate) in an infectious disease ward compared to cleaning with 0.25% NaDCC (17 CFU/plate) or chlorinated trisodium phosphate and potassium bromide (15 CFU/plate) though significance was not specified (Ojajärvi and Mäkelä 1976). A crossover study compared the effectiveness of an **aldehyde-based** disinfectant (Deconex 50 FF) and a **glucoprotamin based** disinfectant (0.5% Incidin Plus) over 8 weeks finding no significant difference in percent surfaces positive for bacteria nor in bacterial concentration (Meinke et al. 2012), however reductions compared to baseline concentrations were not reported.

HAIs

Two studies assessed the efficacies of other manually applied disinfectants on floors for HAI outcomes, however no studies had a high-touch surface intervention.
A controlled crossover study of HAI outcomes in wards cleaning floors with soap detergent or a stabilized chlorinated phenol germicidal cleaning agent with ortho-benzyl parachlorophenol finding no significant difference after the 6-month trial (Danforth et al. 1987)d. A second study did not show a significant difference in HAI outcomes in the 6-month intervention period using an aldehyde disinfectant compared to the prior 6-month period without disinfection (Daschner et al. 1980).

Antimicrobial Surfaces and Coatings

Copper Surfaces

Results

We identified 17 studies that assessed the biocidal effect of copper-alloy surfaces in critical care settings. Study settings ranged from small, rural community hospitals or outpatient infectious disease clinic to large university, veterans' affairs, and cancer hospitals. Studies were conducted in different hospital wards including acute care, intensive care (ICU), pediatric intensive care (PICU), and the medical-surgical ward. Sixteen studies were conducted in high-income countries including Chile (Montero et al. 2019; Schmidt et al. 2016; von Dessauer et al. 2016), Finland (Inkinen et al. 2017), Greece (Souli et al. 2017), the UK (Casey et al. 2010; Karpanen et al. 2012), and the USA (Coppin et al. 2017; Esolen et al. 2018; Hinsa-Leasure et al. 2016; Rai et al. 2012; Salgado et al. 2013; Schmidt et al. 2012, 2020, 2013; Sifri et al. 2016). One study was conducted in an upper-middle income country in South Africa (Marais et al. 2010). Samples were taken of surfaces in patients' rooms, bathrooms, nursing stations, and other parts of the hospital ward.

Interventions included surfaces that were copperized and sampled such as bed rails, tables, IV poles, door push plates, faucet handles, toilet seats, flush levers, grab bars, light switches, keyboards, chairs, cupboards, call buttons, and countertops. The copper composition of surfaces tested in the different studies ranged from containing 16% copper oxide to 99.9% pure copper. Many surfaces were coated with a type of copper/resin composite. Resins act as a glue to hold the copper fibers together and protect them from any mechanical or environmental damage. Many of the studies used copper alloys for intervention surfaces in their healthcare facilities. These metal alloys have copper as their principal component and mixed with, e.g., nickel or silver. These surfaces were compared with non-copper control surfaces to assess copper's ability to reduce environmental contamination.

The efficacy of copper surfaces in reducing bacterial load or percent surfaces positive are summarized by outcome: gram-positive organisms (bacilli and cocci), gram-negative organisms, fungi, all viable organisms, and HAIs.

Organism	Not Effective	Unclear	Effective
All viable organisms	7468 4655	8890	2653 2654 5106 2656 *280 8312 11135 1245 13703 125 7928 6414
Gram positive cocci	2654 6414 8890 7928		2654 13703 6414 7928
Gram positive bacilli	6414		
Gram-negative organisms	8890 7928	7928	6414 7928
Fungi	13703		
HAI	7917 10314		11135 7917

Figure S7. Harvest plot of copper surface interventions with rows identifying outcome pathogen or HAI, columns representing effect (not effective = disinfectant was significantly less effective or not significantly different; unclear = intervention significance or confidence intervals not specified; effective = at least one metric with significant reduction or any metric >90% or > 1 log10 reduction). ǂ denotes that study was defined as effective due to any metric reported > 90% or > 1 log10 reduction. Higher bar height represents better study design (three = controlled crossover; two = cohort or controlled before-after; one = no simultaneous control). Color represents outcome metric (black = concentration; grey = percent surfaces; white = ATP or qualitative). The number identifies the study ID (see table D1 for complete reference). * denotes the disinfectant was compared to another disinfectant rather than comparing before and after disinfection.

Gram-positive organisms

Five studies assessed the effect of copper-alloy surfaces on gram-positive organisms including *C. difficile, Staphylococcus* spp., MRSA, *Enterococcus* spp., and VRE.
One study assessed gram-positive bacilli. No studies assessed concentration. Compared to control surfaces, there was no significant difference in percent surfaces positive for *C. difficile* (Karpenan 2012).

Five studies assessed gram-positive cocci. All five assessed *Staphylococcus* spp. and two assessed MRSA. There was a significant reduction in *Staphylococcus* spp. concentration on copper bed rails (median=0 CFU/100cm^2) compared to control bed rails (median=300 CFU/100cm^2) (Montero) as well as significant reduction in percent surfaces positive for *Staphylococcus aureus* (Karpenen). Two studies found significant reductions in surfaces positive for S. *aureus* (Inkinen, Souli) and MRSA (Karpenen, Schmidt 2012)

Four studies assessed *Enterococcus* spp. and two assessed VRE. No studies assessed *Enterococcus* concentration. There were mixed results for *Enterococcus* spp. with one study finding significant reduction in surfaces positive (4.5% for control, 1.3% for copper) in two rooms (n=685) of ICU (Souli) and another study finding no significant reductions on surfaces from 8 rooms in pediatric ward (n=42) (Inkinen). Copper-alloy surfaces significantly reduced surfaces positive VRE (Schmidt (2012), Karpanen (2012)) compared to analogous non-copper-alloy control surfaces.

Gram-negative bacteria

Three studies assessed the effect of copper-alloy surfaces on gram-negative bacteria and report mixed results. Only one study (Souli) assessed concentration finding significant reductions in concentration on copper surfaces (mean=261 CFU/100 cm^2) compared to control surfaces (mean=1,226 CFU/100 cm^2) for all gram-negative bacteria. There was a significant reduction in percent surfaces positive for gram-negative bacteria (13.8% on copper, 22.7 %for control) in one study (Souli 2017) but no significant reduction in another study (Inkinen 2017). There was a significant reduction in surfaces positive for coliforms (odds ratio=0.398, 95% CI=0.229 – 0.692) (Karpanen 2012). When assessing specific gram-negative organisms, there was not a significant reduction for *K. pneumonia* (1.3% for control, 0.3% for copper) (Souli 2017) or *A. baumannii* (13.6% for control, 9% for copper) (Souli 2017), but there was a non-significant reduction in MDR gram-negative organisms (80% for control, 28% for copper, p=0.058).

Fungi

One study assessed the effect of copper-alloy surfaces on fungus finding no significant reduction in fungus concentration on three different copper-alloy surfaces (median=0 CFU/100 cm^2) compared to non-copper control surfaces (median=0 CFU/100 cm^2) (Montero 2019).

Virus

No studies assessed effects of copper surface interventions on viruses.

All viable organisms

Fifteen studies assessed the effect of copper-alloy surfaces on all viable organisms. Most studies reviewed found that copper surfaces reduced total bacterial burden when compared to the control. All but one study reported significant reductions in bacterial concentration on copper surfaces compared to control surfaces in at least one surface type or for one time point studied. Reported median reduction in bacterial concentration was 90-100% (control median range=0.6 – 87.6) (Casey et al. 2010), 88-90% (median range=1290-1305 CFU/100 cm^2) (Rai et al. 2012), and up to approximately 70% (initial median= ~110 CFU/cm^2) (Karpanen et al. 2012) when copper compared to standard surfaces. Reported mean reduction in bacterial concentration was up to 98% (control mean=6,172CFU/100 cm^2) (Hinsa-Leasure et al. 2016), 94% (Schmidt et al. 2020), 83% (control mean= 2,674 CFU/100 cm^2) (Schmidt et al. 2012), ~73% (control mean=1,100 CFU/100 cm^2), 71% (control mean=2,000 CFU/100 cm^2) (Marais et al. 2010), 66% (control mean range=338 – 3323 CFU/100 cm^2) (Montero et al. 2019), and 63% (control mean= 7,631 CFU/100 cm^2) (Souli et al. 2017). One study reported a 0.76 log10 reduction (Salgado) and 1.94 log10 reduction (Schmidt et al. 2016) in total bacteria recovered on copper surfaces compared to control surfaces. There were significantly lower surfaces > 250 CFU/100 cm^2 on copper surfaces compared to control (control mean range=338 – 3323 CFU/100 cm^2)(Montero et al. 2019) and significantly lower concentration of bacteria on copper beds compared to control beds regardless of length of patient stay (Schmidt et al. 2020). The one study that did not report significant reductions reported lower concentrations on copper surfaces but did not assess significance (Inkinen et al. 2017).

Four studies assessed reductions across time. Notably, two studies (Esolen et al. 2018; Schmidt et al. 2013)found significantly higher initial burden for control bedrails compared to copper bedrails and found that control surfaces had significantly more reductions compared to copper surfaces over 6.5 h (Schmidt et al. 2013) and that both control and copper surfaces had significant reductions over 15 days (Esolen et al. 2018). One study found similar concentrations immediately after routine cleaning, but significantly lower concentration on copper surfaces compared to non-copper surfaces 24 h later (Coppin et al. 2017). Copper beds had significantly lower concentration than control beds regardless of length of patient stay (Schmidt et al. 2020).

HAIs

There were 3 studies that measured the effect of installing copper surfaces in critical care settings on the incidence rate of HAIs. One study did find a significant reduction in rate of all HAIs among infants in rooms with copper fixtures compared to rooms without (relative risk reduction of 0.81 (90% CI: 0.50 – 1.32) (von Dessauer et al. 2016). Another study built a new hospital wing with copper surfaces and compared HAI incidence to the older hospital wing without copper surfaces finding a significant reduction in HAIs due to *C. difficile* and an insignificant reduction in HAIs due to MDROs . Finally, one study found significant reduction in HAIs among patients randomly assigned rooms with copper-containing surfaces compared to rooms without (Salgado et al. 2013).

Other Surfaces

Results

We reviewed 15 studies that tested the effectiveness of other, non-copper surface applications on environmental contamination in acute care settings. Antimicrobial surfaces identified included coatings incorporating metals such as titanium oxide(de Jong et al. 2018; Kim et al. 2018; Özpolat et al. 2011b; Prindis et al. 2018; Reid et al. 2018) and silver ions (Ortí-Lucas and Muñoz-Miguel 2017; Taylor et al. 2009). Other coatings were made of isopropyl alcohol and organofunctional silane (Edmiston et al. 2020; Lewis et al. 2015), organosilane (Boyce et al. 2014), silicon nano-coating (Karunanayake et al. 2019), inorganic metal and organic quaternary ammonium (Lee et al. 2017), silicone quaternary amine (Thom et al. 2014), and quaternary ammonium silyl oxide and titanyl oxide moieties (Tamimi et al. 2014). One study used a probiotics-based cleaning product (Afinogenova et al. 2017). Products were applied using cloths or sprays and allowed to dry to form a surface coating. Coatings that were applied more than once a week were considered manually-applied products rather than surface interventions (e.g. (Yuen et al. 2015)).

Study locations were primarily in high-income countries including the Czech Republic (Prindis et al. 2018), the Netherlands (de Jong et al. 2018), South Korea (Kim et al. 2018), Spain (Ortí-Lucas and Muñoz-Miguel 2017), Taiwan (Lee et al. 2017), the UK (Reid et al. 2018; Taylor et al. 2009), and the USA (Boyce et al. 2014; Edmiston et al. 2020; Lewis et al. 2015; Tamimi et al. 2014; Thom et al. 2014). Two studies were conducted in upper-middle income countries in Russia (Afinogenova et al. 2017) and Turkey (Özpolat et al. 2011b). One study was conducted in a lower-middle income country in Sri Lanka (Karunanayake et al. 2019). Healthcare facilities included community, government, and university-affiliated hospitals. Most surfaces were high-touch, near-patient surfaces on internal medicine wards or in ICUs, where staff equipment such as a computer keyboards and a stethoscope were also included.

The efficacy of non-copper antimicrobial surfaces in reducing bacterial load or percent surfaces positive are summarized by outcome: gram-positive organisms (bacilli and cocci), gram-negative organisms, all viable organisms, and HAIs. No fungus or virus outcomes were assessed. No crossover studies were identified.

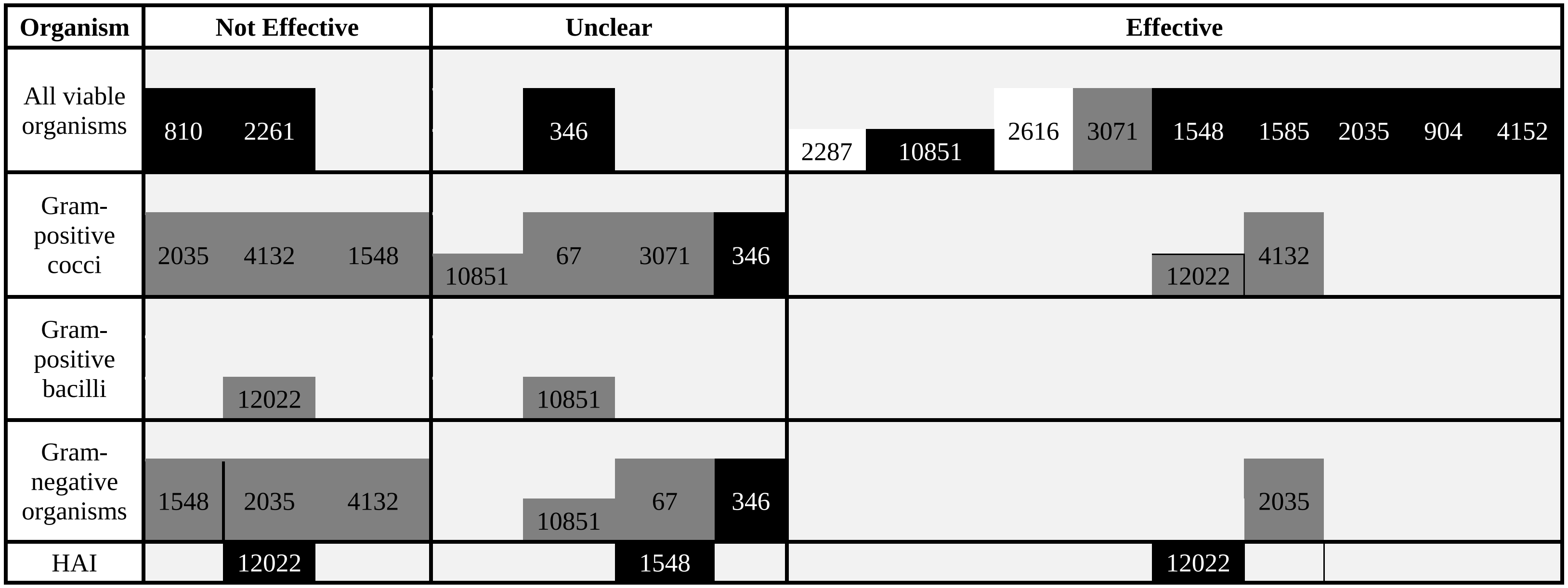

Figure S8. Harvest plot of other, non-copper surface interventions with rows identifying outcome pathogen or HAI, columns representing effect (not effective = disinfectant was significantly less effective or not significantly different; unclear = intervention significance or confidence intervals not specified; effective = at least one metric with significant reduction or any metric >90% or > 1 log10 reduction). ǂ denotes that study was defined as effective due to any metric reported > 90% or > 1 log10 reduction. Higher bar height represents better study design (three = controlled crossover; two = cohort or controlled before-after; one = no simultaneous control). Color represents outcome metric (black = concentration; grey = percent surfaces; white = ATP or qualitative). The number identifies the study ID (see table D1 for complete reference). * denotes the disinfectant was compared to another disinfectant rather than comparing before and after disinfection.

Gram-positive organisms

Eight studies included findings on efficacy of non-copper antimicrobial surfaces against gram-positive organisms including *Bacillus* spp., *Clostridium difficile, Enterococcus* spp., VRE, *Micrococcus* spp., *Staphylococcus* spp., and MRSA.

Two studies assessed gram-positive bacilli. One study did not find high prevalence of *C. difficile* so reductions in concentration could not be ascertained (Tamimi et al. 2014). Surfaces positive for *Bacillus* spp. were not significantly different during the intervention period compared to the pre-intervention period, though initial prevalence was low (initial=4% surfaces positive) (Kim et al. 2018).

Eight studies assessed gram-positive cocci, primarily finding no significant effect.
Only one study reported significant differences, finding reductions in percent surfaces positive for coagulase-negative *Staphylococcus* (initial=26%) comparing 5 months before and after a titanium-dioxide-based intervention (Kim et al. 2018); however, there was no contemporary control. Two other studies of titanium oxide on *S. aureus* reported no significant effects. There was no significant difference in average count of *S. aureus* four months after intervention (initial mean = 116 CFUs) (de Jong et al. 2018) nor in number of isolates recovered twelve weeks (Reid et al. 2018) after coating surfaces with titanium oxide. A study that assessed a silicone quaternary amine surface polymer (Thom et al. 2014) and another that assessed a silicon nano-coating surface (Lee et al. 2017) did not find differences in the percent surfaces positive for *S. aureus* in treated rooms compared to control rooms. There was no difference in recovery of *Staphylococcus* spp. due to low recovery in three studies (Afinogenova et al. 2017; Karunanayake et al. 2019; Tamimi et al. 2014).

For other gram-positive cocci, while one study did have near significant reductions (p=0.054) following the use of silicone quaternary amine antimicrobial surface polymer in percent rooms positive for VRE, all other organism outcomes in this study did not have significant differences between treated and untreated rooms (Thom et al. 2014), and the authors concluded that the antimicrobial surface was not effective. No other studies reported significant effects. There was no significant difference in percent surfaces positive for *Micrococcus* spp. following intervention with a silicon nano-coating surface compared to untreated surfaces (Karunanayake et al. 2019), for VRE following inorganic metal and organic quaternary ammonium surface treatment (Lee et al. 2017), nor for *Enterococcus* spp. following quaternary ammonium silyl oxide and titanyl oxide moieties (Tamimi et al. 2014). There was no difference in recovery of *Enterococcus* spp. (Afinogenova et al. 2017), due to low recovery.

Gram-negative organisms

Six studies included findings on efficacy of non-copper antimicrobial surfaces against gram-positive organisms including *Acinetobacter baumannii*, coliforms, *Enterobacteriaceae* spp.,CRE, and *Pseudomonas aeruginosa.*

Only one study found significant effect of coated surfaces on gram-negative bacteria. There were significantly fewer percent surfaces positive for *A. baumannii* on surfaces coated with

silicon nano-coating compared to control surfaces (Karunanayake et al. 2019). However, another study found no difference in reductions in CRAB prevalence when assessing baseline conditions for both treated and untreated surfaces with similar reductions between control and treated surfaces (Lee et al. 2017).

All other studies did not find significant effects of coated surfaces. Prevalence of coliforms (Karunanayake et al. 2019), *Enterobacteriaceae* spp. (Afinogenova et al. 2017; Karunanayake et al. 2019) and *P. aeruginosa* (Thom et al. 2014) were not significantly different between treated and untreated surfaces or rooms. There was no difference in recovery of *Enterobacteriaceae* spp. (initial mean=0 CFU) (deJong), CRE (baseline prevalence=3%) (Tamimi et al. 2014), *E. coli* (Thom et al. 2014), *K. pneumonia* (Thom et al. 2014), and *A. baumannii* (Thom et al. 2014) due to low recovery.

Fungi

There were no studies that assessed the effect of non-copper antimicrobial surfaces on fungi.

Virus

There were no studies that assessed the effect of non-copper antimicrobial surfaces on viruses.

All viable organisms

Twelve studies assessed efficacy of non-copper antimicrobial surfaces on all viable organisms. One study comparing bacterial concentrations on treated and untreated surfaces to baseline concentrations found significant reductions on surfaces treated with inorganic metal and organic quaternary ammonium by 81% (initial mean=3,252 CFU/100 cm^2) (Lee et al. 2017) compared to control surfaces. Other studies found significant differences between treated and untreated surfaces without comparing concentrations to baseline concentrations. There were significantly lower concentrations on treated surfaces compared to untreated by 88% (control mean=14.3 CFU) (Lewis et al. 2008), 36-56% (Karunanayake et al. 2019), and 62-98% (range of mean for control=96-1140 CFU) (Taylor et al. 2009). Treated surfaces had lower range of bacterial count on treated surfaces after 6 weeks compared to untreated surfaces (Edmiston et al. 2020). A 3-log concentration reduction was observed four weeks after application with quaternary ammonium product, containing titanium oxide moieties, with percent of sites with >10,000 CFUs falling from 71.5% to 11.1% over the 15-week study period (P<0.0005) (Tamimi et al. 2014).

For titanium dioxide treatments, there was a significant reduction in particle count compared to before intervention (Özpolat et al. 2011a), significantly lower ATP values on treated compared to control surfaces (Prindis et al. 2018), and sustained antimicrobial effects over time (Reid et al. 2018). Odds of growing less than 2.5 CFU/cm^2 fell by 2.5% per day for coated surfaces (OR=0.95; 95% CI: 0.925 to 0.977; P<0.001) compared to increasing 2.6% per day for uncoated (OR=1.026; 95% CI 1.009 to 1.043; P=0.003; (Reid et al. 2018)).

However, concentration was not lower compared to baseline (mean baseline=161 CFU/room) (de Jong et al. 2018) among surfaces treated with titanium dioxide compared to untreated surfaces.

Surfaces treated with two different organosilane test products did not have significantly different concentrations over 4-week period (mean range for control= 15-115 CFU) (Boyce et al. 2014). One silver-ion containing antimicrobial coating had significantly higher concentration on treated bedside surfaces compared to control surfaces (control mean=0.10 CFU/cm^2) (Ortí-Lucas and Muñoz-Miguel 2017).

One study compared reductions in in percent samples positive for all MDROs (MRSA, VRE, CRAB, CRE) before and after coating treatment with higher reductions in treated surfaces of 46%-83% (initial=12-20%) compared to control surfaces at 35%-65% (initial=12-20%) (Lee et al. 2017). Notably, when MDROs were analyzed separately, there were not significant differences.

HAIs

Two studies assessed HAI outcomes. Percent change in incidence of new-onset sepsis pre- vs post- intervention were significant in both treated and untreated rooms over a 3-month period, with a 28.5% decline in rooms with treated surfaces (initial incidence=33.3%) and a 63.6% increase in control rooms (initial incidence=25%) (Lee et al. 2017). However, the difference was not significant when post-intervention incidences were compared and the authors concluded that the antimicrobial surface did not effectively reduce the incidence of HAIs in their study (Lee et al. 2017). Another study compared incidence rates of HAIs 5 months before and after an intervention that coated high-touch surfaces with titanium dioxide and found significant reduction in MRSA incidence (initial=9.3/1000 patient-days) and hospital-acquired pneumonia (initial=16.1/1000 patient-days) but no reduction in bloodstream infections, UTIs, CDAD, VRE, or MDR *A. baumannii* (Kim et al. 2018). This study did not include a control group during the intervention period.

Vaporized Disinfectants

Hydrogen Peroxide Vapor

<u>Results</u>

This review identified 33 studies assessing efficacy of HPV on environmental surfaces. The majority of studies were conducted in high-income countries including Australia (Chan et al. 2011; Mitchell et al. 2014; Oon et al. 2020), France (Barbut et al. 2009, 2013; Blazejewski et al. 2015), Israel (Lerner et al. 2019), Italy (Mosci et al. 2017), Kingdom of Saudi Arabia (Humayun et al. 2019), Netherlands (Otter et al. 2010), Norway (Andersen et al. 2008), Sweden (Holmdahl et al. 2016), the UK (Ali et al. 2016; Bates and Pearse 2005; Best et al. 2014; Doan et al. 2012; French et al. 2004; Garvey et al. 2016; Hardy et al. 2007; Otter et al. 2007, 2016; Shapey et al. 2008; Yui et al. 2017), and the USA (Boyce et al.; Havill et al. 2012; Manian et al. 2011, 2013b; Mccord et al. 2016; Passaretti et al. 2013; Ray et al. 2010). One study was conducted in an upper-middle income country in Russia (Popov and Anuchina 2016), and two in a lower-middle income country in India (Singh et al. 2017; Taneja et al. 2011). Studies included university, secondary, tertiary care, teaching, small district, and acute care hospitals. Critical care settings included burns unit, CU, surgical ward, cardiovascular ward, long-term care ward, isolation rooms, operation rooms, treatment rooms, and equipment rooms. Environmental surfaces included high-touch surfaces, floors, toilets, bedrails, computer keyboards, telephones, door handles, nurse call bells, mattresses, curtain rails, chairs, other surfaces near to patient’s bed, and others.

The efficacy of HPV in reducing bacterial load or percent surfaces positive are summarized by outcome: gram-positive organisms (bacilli and cocci), gram-negative organisms, fungi, all viable organisms, and HAIs.

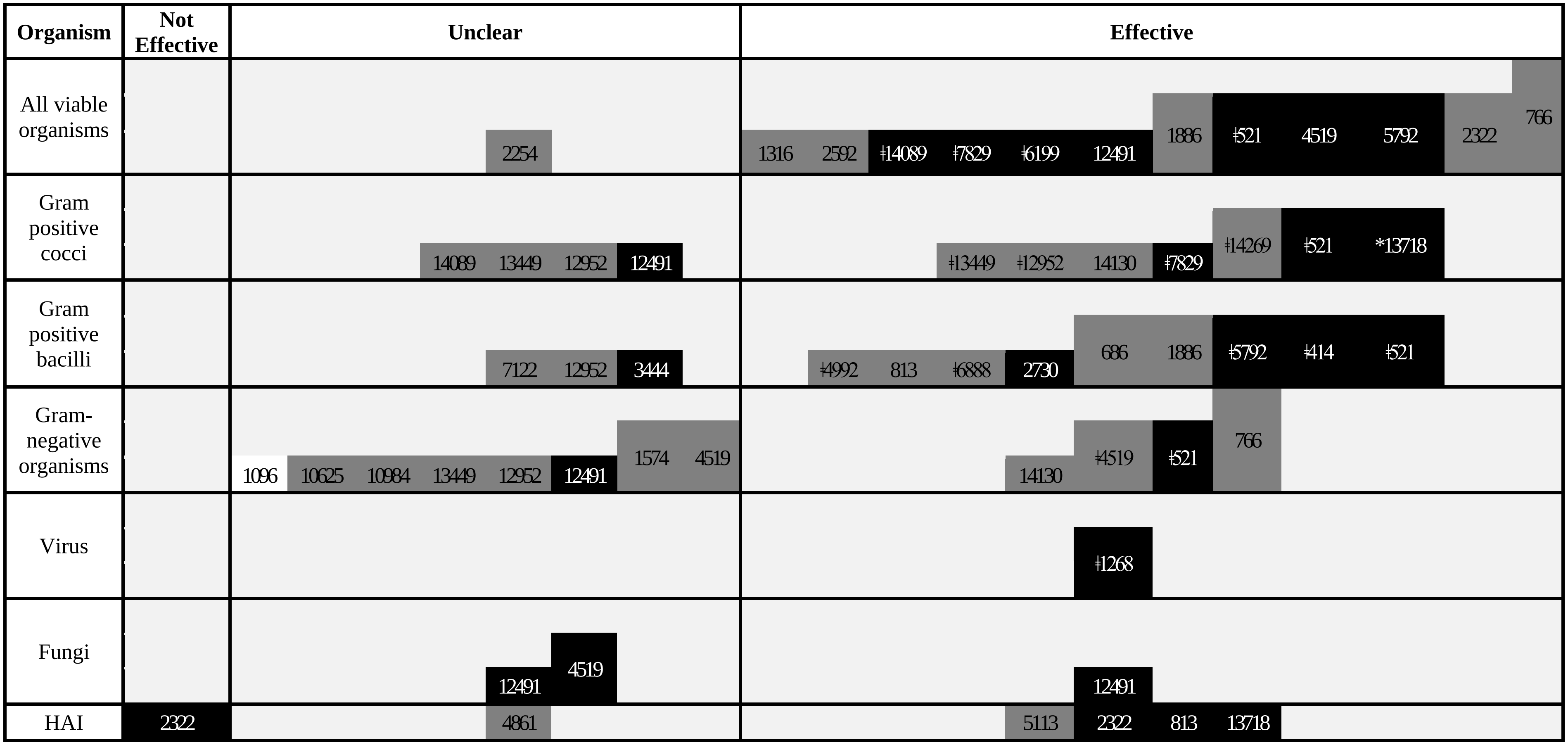

Figure S9. Harvest plot of vaporized hydrogen peroxide disinfection with rows identifying outcome pathogen or HAI, columns representing effect (not effective = disinfectant was significantly less effective or not significantly different; unclear = intervention significance or confidence intervals not specified; effective = at least one metric with significant reduction or any metric >90% or > 1 log10 reduction). ǂ denotes that study was defined as effective due to any metric reported > 90% or > 1 log10 reduction. Higher bar height represents better study design (three = controlled crossover; two = cohort or controlled before-after; one = no simultaneous control). Color represents outcome metric (black = concentration; grey = percent surfaces; white = ATP or qualitative). The number identifies the study ID (see table D1 for complete reference). * denotes the disinfectant was compared to another disinfectant rather than comparing before and after disinfection.

<u>Gram-positive organisms</u>

There were 19 studies assessing efficacy of HPV on gram-positive organisms including *Bacillus* spp., *C. difficile*, *Staphylococcus* spp., MRSA, and VRE.

Twelve studies assessed gram-positive bacilli with nine studies specifically assessing *C. difficile*. Four studies reported significant reductions for *C. difficile* following HPV (Barbut et al. 2009; Boyce et al.; Mosci et al. 2017; Shapey et al. 2008). Studies found 6-log reduction in concentration on all site types (Havill et al. 2012), 5.1 log10 reductions relative to control reductions (Ali et al. 2016), median log10 reduction of 2.3 CFU (Doan et al. 2012), 94% significant reduction in concentration (initial mean=13.8 CFU) (Shapey et al. 2008) and 67% reduction in concentration (initial mean = 21.2 CFU) (Yui et al. 2017). There was a 4-log reduction (Havill et al. 2012) and 6-log reduction (Otter et al. 2016) in all biological indicators (*G. stearothermophilus*). Following terminal cleaning with HPV, samples > 6 CFU significantly decreased from 25% to 0% after HPV (Boyce et al.) and had significantly lower percent surfaces positive (Barbut et al. 2009; Mosci et al. 2017). Additionally, biological indicators were negative after three HPV cycles (Andersen et al. 2006b) and unspecified spore-bearers were not found after HPV (initial=7% surfaces positive) (Bates and Pearse 2005). Two studies assessed addition of HPV following terminal disinfection with sodium hypochlorite. There was a 45% reduction in surfaces positive (initial=11% surfaces) following sodium hypochlorite with further reductions of 86% following HPV (after hypochlorite=6%) (Best et al. 2014) for combined 92% reduction using both. Following terminal disinfection with peracetic acid-based manual disinfection, there was a 76% reduction in concentration (initial=86.9 CFU) with further reductions of 67% following HPV for combined 92% reduction in concentration and combined 83% reduction in surfaces positive (Yui et al. 2017). Three studies compared efficacy of HPV on *C. difficile* to other disinfectants. One study found that HPV had significantly fewer surfaces positive compared to sodium hypochlorite (Barbut et al. 2009) while another did not find significant differences between the disinfection interventions (Mosci et al. 2017). In another study, HPV along with 1000 ppm chlorine-releasing agent and peracetic wipes were the most effective interventions among eight interventions assessed (Doan et al. 2012).

Nine studies found reductions in gram-positive cocci following HPV. Two studies reported significant reductions in MRSA due to HPV (Manian et al. 2011; Mitchell et al. 2014). There was a 6.3 log10 reduction of inoculated MRSA after HPV relative to control (Ali et al. 2016). An uncontrolled study with high contamination found over 99% reduction in total *Staphylococcus* (initial=2343 CFU), *S. aureus* (initial=891 CFU), and MRSA (initial=379 CFU) (Taneja et al. 2011). There was a significant 32% reduction (initial=25%) following either manual or vaporized hydrogen peroxide disinfection (Mitchell et al. 2014). Though significance was not specified, there was a 98% reduction in percent surfaces positive (initial=72%) for MRSA following HPV (French et al. 2004) and a 93% reduction in percent surfaces coagulase-negative Staphylococci (initial=63% surfaces positive) (Bates and Pearse 2005). Following terminal cleaning with a QAC, there was a 33% reduction in surfaces positive for MRSA (initial=60%) followed by 92% reduction (after QAC=40%) following HPV for combined 94% reduction in surfaces positive (Otter et al. 2007). Following terminal cleaning with sodium hypochlorite there was not a significant reduction, however the addition of HPV had a significant reduction in sites positive for MRSA (Manian et al. 2011). Three studies found no surfaces

positive after HPV but had low prevalence of *S. aureus* at 1 CFU/100 cm^2 from one sample (Barbut et al. 2013), 4 sites positive for *S. aureus* prior to HPV (Bates and Pearse 2005), and 5 sites positive for MRSA (Hardy et al. 2007) before HPV. One study assessed efficacy on VRE found low prevalence of VRE prior to disinfection (initial= 1 surface positive) (Otter et al. 2007).

Gram-negative organisms

There were 11 studies assessing efficacy of HPV on gram-negative organisms including *Acinetobacter* spp., CRAB, *Escherichia coli, Klebsiella* spp., *Pseudomonas* spp., *Serratia marcescens*, antibiotic-resistant gram-negative rods, carbapenamase-producing coliforms, ESBL-producing gram-negative bacilli. Most studies found reductions, though only two specified significance (Blazejewski et al. 2015; Manian et al. 2011).

HPV reduced prevalence of *A. baumannii*. There was a 78% reduction (initial=80%) of surfaces positive for CRAB after HPV (Lerner et al. 2019). With the addition of HPV to terminal cleaning with sodium hypochlorite, there was a significant reduction in surfaces positive for *A. baumannii* complex (Manian et al. 2011). MDR *A. baumannii* was not recovered immediately after and one week after HPV (initial=7 rooms positive) (Ray et al. 2010). After HPV, *A. baumannii* was not found (initial= 4 CFU/100 cm^2 on one surface) (Barbut et al. 2013).
A crossover trial found significant reductions of 86% reduction (initial=0.96%) in ESBL gram-negative bacilli after HPV (Blazejewski et al. 2015). For all other studies, while significance wasn't assessed, there was a 6.3 log10 reduction after HPV compared to control for inoculated *K. pneumonia* (Ali et al. 2016) and a 67% reduction (initial=56%) surfaces positive for *Klebsiella* spp.(Singh et al. 2017). After HPV, surfaces were not positive for *E. coli* (initial=1%-33%) (Barbut et al. 2013; Singh et al. 2017), *Pseudomonas* spp. (initial=11%) (Singh et al. 2017), *Serratia* spp. (initial=5%) (Bates and Pearse 2005), coliforms (initial=17%) (Bates and Pearse 2005), gram-negative rods (initial=10-48%) (Otter et al. 2007, 2010). After terminal disinfection with 1000 ppm NaDCC and 6% HPV, some surfaces remained positive for carbapenemase-producing coliforms, however these organisms were not recovered after increasing concentrations of NaDCC to 2000 ppm and 12% HPV (Garvey et al. 2016).

Fungi

There were two studies assessing efficacy of HPV on fungi. One study found significant reductions in fungal concentrations with no fungus detected after HPV (initial mean=3.5 CFU/100 cm^2) (Barbut et al. 2013). Other studies did not report significance not recovering fungus after HPV (initial= 0-2 CFU) (Singh et al. 2017). *Aspergillus* spp. were not recovered from surfaces after HPV (initial=4.5%) (Barbut et al. 2013).

Virus

One study assessed the efficacy of HPV on viruses using inoculated feline calicivirus and murine norovirus as models for human norovirus (Holmdahl et al. 2016). This study found that no viable virus was recovered and that HPV demonstrated at least a 3.65 log reduction for feline calicivirus and 4.67 log reduction for murine norovirus (Holmdahl et al. 2016).

<u>All viable organisms</u>

There were 13 studies assessing efficacy of HPV on all viable organisms. Eight studies reported significant reductions (Barbut et al. 2013; Blazejewski et al. 2015; Havill et al. 2012; Humayun et al. 2019; Mosci et al. 2017; Passaretti et al. 2013; Popov and Anuchina 2016; Singh et al. 2017).

Significant reductions in concentration were reported with initial concentration of 16 CFU (Singh), range of initial mean concentration 12 – 53 CFU (Havill et al. 2012) and 2.9 – 4 CFU/100 cm^2 (Barbut et al. 2013). Reductions over 90% without significance assessment were reported with initial range of median concentration 21 – 28 CFU/25 cm^2 (Ali et al. 2016), average concentration 2 – 6.5 log10 CFU (Taneja et al. 2011), and counts 25 – 230 CFU (Hardy et al. 2007)and 11 – 531 CFU (Chan et al. 2011).

Significant reductions were also observed in percent surfaces positive for bacteria after HPV with 70% reduction (initial=80%) (Humayun et al. 2019), 60% (initial=85%) (Popov and Anuchina 2016), 33% (initial=38%) (Blazejewski et al. 2015), and no surfaces positive (initial=13%) (Mosci et al. 2017). MDROs were significantly lower in patient rooms using HPV compared to control rooms (relative risk=0.65) (Passaretti et al. 2013). One study did not report significance but found that intervention period with low (0.02 ppm) concentration of HPV had higher number of samples with bacterial concentrations > 2.5 CFU/cm^2 compared to control periods using manually applied disinfectants (Oon et al. 2020).

<u>HAIs</u>

There were 5 studies that assessed the effect of HPV disinfection of environmental surfaces on HAI outcomes.

One study (Passaretti et al. 2013) compared HAI incidence of patients in rooms with HPV addition to standard cleaning compared concurrently to patients in rooms standard cleaning alone and did not find significant differences in HAIs due to MRSA (risk ratio=0.53, 95% CI=0.16 – 1.79), *C. difficile* (risk ratio= 0.49, 95% CI= 0.16 – 1.47), or MDR-gram-negative rods (risk ratio=0.55, 95% CI=0.20 – 1.57). However, Passaretti et al. did find significant reductions in VRE HAIs (risk ratio=0.25, 95% CI=0.10 – 0.60).

Four studies compared a prior period without HPV to an intervention using HPV. Two studies reported a significant reduction of *C. difficile* HAIs with initial rate of 0.88/1000 patient-days (rate ratio=0.63, 95% CI=0.50 – 0.79) after addition of HPV to terminal cleaning (Manian et al. 2013b) and 2.28/1000-patient-days (rate ratio=0.52) (Boyce et al.). Another study found insignificant 60% reductions of *C. difficile* HAIs after the introduction of HPV (initial rate=1.0 cases/1000 patient-days) (Mccord et al. 2016). MRSA HAIs also decreased from 9.0/10,000 patient-days using detergent for terminal cleaning (rate ratio=0.59) after introduction of hydrogen peroxide vapor or manually-applied hydrogen peroxide for terminal cleaning (Mitchell et al. 2014).

Other Vapor

Other vaporized disinfectants include all other disinfection interventions applied through vapor, mist or fog excepting HPV and included chlorine dioxide, sodium hypochlorite, essential oils, formalin, QACs, glutaral, beta-propiolactone, steam, acidic electrolytic water, ozone, and steam.

Results

This review identified 18 articles assessing vaporized disinfectants (excepting HPV) on environmental surfaces at tertiary care hospitals, community hospitals, university hospitals, and acute care facilities including ICU, surgical center, biocontainment patient care unit, and isolation rooms. Eleven studies were conducted in high-income countries including France (Ory et al. 2019), Italy (Gelmini et al. 2016), Japan (Aimiya et al. 1989; Nagai et al. 1983; Nakata et al. 2001), Romania (Strat 1971), the UK (Doan et al. 2012; Dyas et al. 1983), and the USA (Barbeito 1966; Lowe et al. 2013a, 2013b; Munster and Ostrander 1974; Sexton et al. 2011). Three studies were conducted in upper-middle income countries including Bosnia and Herzegovina (Čamdžić et al. 2019), Russia (Alekseeva et al. 1969), and Turkey (Oztoprak et al. 2019). Two studies were conducted in India, a lower-middle income country (Shekhawat et al. 1992; Singh et al. 2017). Surfaces sampled included bed rails, call bells, bedside tables, bedside lockers, patient chairs, handrails, floors, sink taps, and others.

The efficacy of vaporized disinfectants (excepting HPV) for reducing bacterial load or percent surfaces positive are summarized by outcome: gram-positive organisms (bacilli and cocci), gram-negative organisms, fungi, all viable organisms, and HAIs.

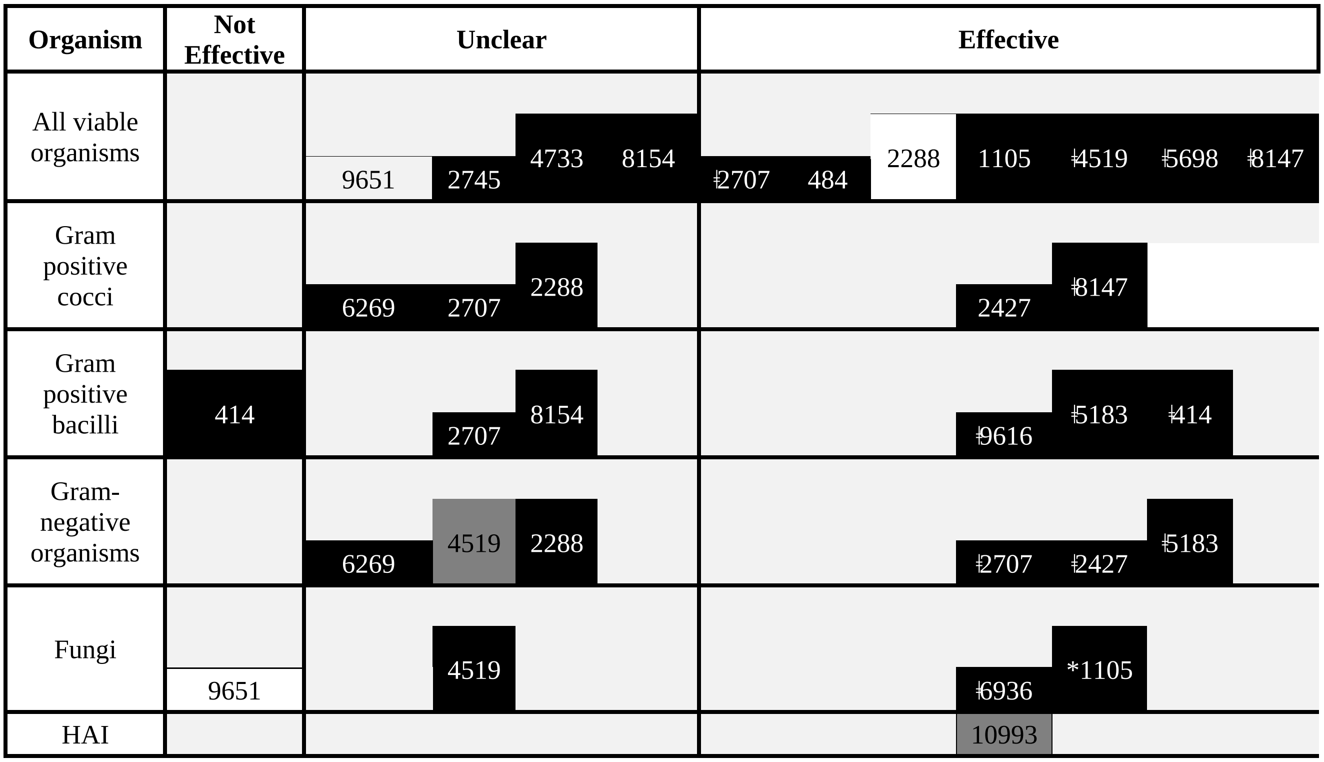

Figure S10. Harvest plot of other vaporized disinfection (excepting hydrogen peroxide vapor) with rows identifying outcome pathogen or HAI, columns representing effect (not effective = disinfectant was significantly less effective or not significantly different; unclear = intervention significance or confidence intervals not specified; effective = at least one metric with significant reduction or any metric >90% or > 1 log10 reduction). ǂ denotes that study was defined as effective due to any metric reported > 90% or > 1 log10 reduction. Higher bar height represents better study design (three = controlled crossover; two = cohort or controlled before-after; one = no simultaneous control). Color represents outcome metric (black=concentration; grey = percent surfaces; white = ATP or qualitative). The number identifies the study ID (see table D1 for complete reference). * denotes the disinfectant was compared to another disinfectant rather than comparing before and after disinfection.

<u>Gram-positive organisms</u>

Nine studies assessed the effect of vaporized disinfectants on surfaces on gram-positive organisms including *Bacillus* spp., *C. difficile, Enterococcus* spp., VRE, *Staphylococcus* spp., and MRSA.

Five studies assessed gram-positive bacilli. For *Bacillus* spp. indicator organisms, two studies assessed efficacy of beta-propiolactone (BPL) or chlorine dioxide vapor. After BPL disinfection, *B. subtilis* was not recovered from any surfaces (initial concentration 104 spores/mL) (Barbeito 1966). After spraying with chlorhexidine gluconate, *B. subtilis* was not recovered from floors, however 67% of samples from walls and ceilings remained positive after spraying for 60 min (Nagai et al. 1983). After chlorine dioxide vapor disinfection, *B. atrophaeus* was inactivated in

all but two sites which were behind a closed door (initial concentration=10^6 spores) and *B. anthracis* spores had 7.8-10.0 log10 reductions (initial concentration=10^{10} CFU) (Lowe et al. 2013a).

For *C. difficile*, two studies assessed efficacy of dry ozone, dry atomized steam cleaning, and steam vapor. In one study that inoculated *C. difficile* into rooms, reductions were larger for dry ozone intervention (median log10 reduction=1.3) compared to steam cleaning (0.56) and dry atomized steam cleaning (0.53) (Doan et al. 2012). Dry ozone was not significantly different than manually applied chlorine-based or peracetic acid disinfectants, but was less effective than manually applied hydrogen peroxide disinfectant. Both steam cleaning interventions were significantly less effective than manually applied hydrogen peroxide, chlorine-based, and peracetic acid disinfectants (Doan et al. 2012). There was low initial burden in one study assessing effect of saturated steam vapor with no surfaces positive after disinfection (initial=1 of 48 surfaces positive) (Sexton et al. 2011).

Five studies assessed reductions in gram-positive cocci due to steam, ozone, chlorine dioxide, and other fogging chemicals. For *Staphylococcus* spp., after disinfection with saturated steam vapor devise, no MRSA were detected with up to 0.35 log10 reduction in average concentration (range of initial mean=<4.0 – 9.0 CFU/in2) and up to 0.24 log10 reduction in average concentration of methicillin-intermediate *S. aureus* (MISA) after disinfection (range of initial mean = 4.5 – 7.0 CFU/in2) (Sexton et al. 2011). After steam disinfection, no MRSA were recovered (initial concentration unspecified) (Oztoprak et al. 2019). Chlorine dioxide resulted in a mean log10 reduction of 8.75 (99.6%) (Lowe et al. 2013b). Significance not specified since complete inactivation for most samples. After standard cleaning two surfaces were positive for MRSA (maximum concentration 100 CFU/cm^2) but no MRSA were recovered following subsequent ozone disinfection (Čamdžić et al. 2019). One study reported *S. aureus* concentration reductions (initial concentrations not specified) of 89% for fogging with a hydrochloride (alkyldiaminoethylglycine), 90% for fogging with acidic electrolytic water, 95% for fogging with a QAC (benzalkonium chloride), and 95% for fogging with sodium hypochlorite, 97% for fogging with glutaral (Nakata et al. 2001).

For *Enterococcus* spp., chlorine dioxide resulted in mean log10 reduction of 9.02 (99.5%) for *E. faecalis* (Lowe et al. 2013b). After steam disinfection, no VRE were recovered (initial concentration unspecified) (Oztoprak et al. 2019).

<u>Gram-negative organisms</u>

Six studies assessed the effect of vaporized disinfectants on surfaces for gram-negative organisms including *Acinetobacter* spp., *Escherichia* spp., *Francisella tularensis*, *Klebsiella* spp., *Pseudomonas* spp., *Yersinia pestis*, and total coliforms.
For *Acinetobacter* spp., three studies assessed efficacy of steam, ozone, and chlorine dioxide. After steam disinfection, no surfaces were positive for MDR *A. baumannii* (initial concentration not specified) (Oztoprak et al. 2019). After standard cleaning three surfaces were positive for *A. baumannii* (maximum concentration 100 CFU/cm^2) but no *A. baumannii* were recovered following subsequent ozone disinfection (Čamdžić et al. 2019). After use of chlorine dioxide vapor, the mean $\log_{10}$ reduction for inoculated MDR *A. baumannii* was 8.55 (99.3%) and for inoculated *M. smegmatis* was 9.32 (99.3%) (Lowe et al. 2013b).

For other gram-negative bacteria, four studies assessed efficacy of steam, QAC vapor, and chlorine dioxide vapor. After steam disinfection, no surfaces were positive for carbapenem-resistant *Pseudomonas aeruginosa* (initial concentration not specified) (Oztoprak et al. 2019). There was a reduction of 88% in percent surfaces positive and up to 96% reduction in concentration on bedrails (initial mean= 106 CFU/in2) for total coliforms (initial=81% surfaces positive) following steam vapor disinfection (Sexton et al. 2011). One study implemented two separate QAC fogging disinfectants finding reductions in *Klebsiella* spp. of up to 46% (initial=89% surfaces positive) (Singh et al. 2017) and no recovery of *Pseudomonas* spp. (initial maximum=33% surfaces positive) or *E. coli* (initial maximum=11% surfaces positive) following both QAC vapors (Singh et al. 2017). Following disinfection with chlorine dioxide vapor, the mean log10 reduction for two strains of inoculated *E. coli* (two strains) was 9.02 (99.2%) (Lowe et al. 2013b), range of percent inactivation of inoculated *Yersinia pestis* spores was 100% with range of average $\log_{10}$ reduction of 6.9 – 8.8 (Lowe et al. 2013a), and range of percent inactivation of *Francisella tularensis* spores was 100% with range of average log10 reduction of 8.8 – 9.6 (Lowe et al. 2013a).

Fungi

Four studies assessed the effect of ozone, formaldehyde, two vaporized QACs, and vaporized essential oils on surfaces for fungi. A small ozone generator produced ozone at < 0.001 ppm in a hospital room and did not have an effect on inoculated fungal concentrations (Dyas et al. 1983). After disinfection with 40% aqueous solution of formaldehyde intervention followed by a neutralization with 25% ammonia, only one surface was positive of 144 samples (range of initial total contamination=$182x10^6$-$233x10^7$ CFU/cm^2) (Alekseeva et al. 1969). One study implemented two separate QAC fogging disinfectants with no recovery after disinfection with one QAC (initial=3 CFU) and 50% reduction with the second QAC (initial=4 CFU) (Singh et al. 2017). One intervention compared standard cleaning to disinfection with 0.2% essential oil vapor finding a significant reduction in fungi on tables and cabinets compared to control sites throughout the study (Gelmini et al. 2016).

Virus

No studies assessed effects of other vaporized disinfection interventions on viruses.

All viable organisms

Eleven studies assessed the effect of vaporized disinfectants on surfaces for all viable organisms. Vapors included steam (Oztoprak et al. 2019; Sexton et al. 2011), formalin (Shekhawat et al. 1992), QACs (Munster and Ostrander 1974; Nakata et al. 2001; Singh et al. 2017), alcohol (Nagai et al. 1983; Strat 1971), hydrochloride (Aimiya et al. 1989; Nagai et al. 1983; Nakata et al. 2001; Strat 1971), ozone (Dyas et al. 1983), essential oils (Gelmini et al. 2016), sodium hypochlorite (Nakata et al. 2001), glutaral (Nakata et al. 2001), and electrolytic water (Nakata et al. 2001).

After steam disinfection, there was 1.2 log10 reduction in concentration on bedrails (initial mean=1590 CFU) (Sexton et al. 2011) and also significant reductions of 98%in ATP (initial= 578 RLU) (Oztoprak et al. 2019). One intervention compared standard cleaning to disinfection with 0.2% essential oil vapor finding a significant reduction of up to 90% in total bacteria concentration compared to control sites throughout the study (Gelmini et al. 2016).

One study saw a floor bacterial samples reduced from 57-66% (initial=320-388 CFU/cm^2) after fumigation with formalin (Shekhawat et al. 1992). After fogging with a vaporized QAC**,** there was a 76% mean reduction in bacterial concentration (initial mean=26 CFU) (Munster and Ostrander 1974) and one of two QAC vapors had significantly lower number of samples positive after disinfection and at least 95% reduction in bacterial concentration (range of initial count= 40-50 CFU) (Singh et al. 2017). Vaporized alcohol-based disinfectant followed by detergent or hydrochloride disinfection had significantly lower concentration compared to standard cleaning and reduced bacterial load by 90% with detergent and by 95% with hydrochloride disinfection (Strat 1971)When using a spray method to apply chlorhexidine gluconate or alkylpolyaminoethyl glycine chloride, efficacy was better for floors compared to walls and ceilings (initial concentrations not specified) (Nagai et al. 1983). One study reported reductions for in bacterial concentration for five disinfectants (initial concentrations not specified) of 90% for fogging with a hydrochloride (0.5% alkyldiaminoethylglycine), 77% for fogging with acidic electrolytic water, 91% for fogging with 0.5% glutaral, 93% for fogging with a QAC (0.2% benzalkonium chloride), and 93% for fogging with 0.2% acidic electrolytic water sodium hypochlorite (Nakata et al. 2001). There were also significant reductions in median bacterial concentration for fogging with hydrochloride (0.5% alkyldiaminoethylglycine, initial=10-40 CFU/10 cm^2) (Aimiya et al. 1989). A small ozone generator produced ozone at < 0.001 ppm in a hospital room and did not have an effect on all viable bacteria (Dyas et al. 1983).

An uncontrolled cohort study by Aimiya et al. compared the bacterial count after cleaning with 0.2% and 0.5% QAC benzalkonium chloride (Osvan), 0.2% hydrochloride solution of alkyldiaminoethyl glycine (Tego-51) and 0.2% and 0.5% chlorhexidine digluconate (Hibitane). A significant reduction in bacterial concentration was found ($p<0.001$), with counts before cleaning ranging from 10-45 CFU/10cm^2 as compared to 0-1 CFU/10cm^2 after cleaning (Aimiya et al. 1989). The bacterial counts were not specified separately for the three specific disinfectants (Aimiya et al. 1989)

<u>HAIs</u>

One study assessed HAIs in the NICU was compared retrospectively for four periods using or not using steam cleaning finding that the periods with steam cleaning had significantly lower incidence compared to periods without (Ory et al. 2019). The incidence of infection or colonization with *S. capitis* was 1.04% before use, 0.55% with use of steam cleaning, 3.95% when steam cleaning was out-of-order, and 0.0% when steam cleaning returned (Ory et al. 2019).

Supplementary Material 5: Tables for Disinfection Efficacy by Disinfection Intervention and Outcome

Table of Contents:

Table S11: Study results for manually applied alcohol interventions ordered by outcome organism
Table S12: Study results for manually applied peroxygen interventions ordered by outcome organism
Table S13: Study results for manually applied quaternary ammonium compound interventions ordered by outcome organism
Table S14: Study results for manually applied sodium hypochlorite interventions ordered by outcome organism
Table S15: Study results for manually applied chlorine interventions (excepting sodium hypochlorite) ordered by outcome organism
Table S16: Study results for other manually applied interventions ordered by outcome organism
Table S17: Study results for copper antimicrobial surface interventions ordered by outcome organism
Table S18: Study results for other, non-copper surface interventions ordered by outcome organism
Table S19: Study results for vaporized hydrogen peroxide interventions ordered by outcome organism
Table S20: Study results for other vapor interventions ordered by outcome organism

Applied Manually

Table S11: Study results for manually applied alcohol interventions ordered by outcome organism

Reference (Study ID)	Outcome	Study Design	Setting	Intervention	Results
Casini 2017 (129)	All viable organisms	Single-site, Quasi-experimental, uncontrolled before-after study over 6 months	206 samples were taken from high-touch surfaces (mobile and office telephones, tablets, keyboards and mouses, touchscreen monitors, bed rails, and patient tables) surrounding patients in a 7-bed regional burns center at a tertiary-care teaching hospital. Pisa, Italy.	Standard cleaning with chlorine sodium hypochlorite (1400 mg/L available chlorine) compared to enhanced cleaning with addition of twice-daily wiping all high-touch surfaces with 0.5% chlorhexidine-60% isopropyl alcohol solution. Samples were taken from every cleaned surface one a week in late morning after cleaning	Initial environmental monitoring found number (percent) of surfaces with unacceptable (> 20 CFU/100 cm2) bacterial count at 13/103 (12.6%). During standard hypochlorite, 3/23 (13%) surfaces were unacceptable. During improved cleaning with addition of CHG, 2/50 (4%) surfaces were unacceptable. Also, during improved cleaning 8/30 (27%) surfaces were unacceptable possibly due to low adherence to protocol. No significance reported.
Dramowski 2016 (442)	All viable organisms	Single-site, Quasi-experimental, uncontrolled before-after study over 15 months	250 samples from 5 surfaces (bedrail, bedside table, sink, door handle, and mattress) were sampled in 25 pediatric isolation rooms in a 300-bed children's hospital, in a 1,384-bed academic hospital. Cape Town, South Africa	Routine cleaning consisted of water and detergent. Terminal cleaning was usually conducted with 70% alcohol except when sodium hypochlorite was used for patients with *Clostridium difficile* infections. Samples were taken after routine cleaning/before terminal cleaning and after terminal cleaning.	Mean bacterial count was significantly lower after terminal cleaning compared to before (15 ± 30 after, 39 ± 41 before; p <0.001). Mean ATP bioluminescence was low before terminal cleaning and decreased significantly after terminal cleaning (72 ± 40 before, 23 ± 11 after; p < 0.001) and the number of surfaces considered clean (<100 RLU) after terminal cleaning significantly increased compared to before (p < 0.001).
Andersen 2009 (568)	All viable organisms	Single-site, Quasi-experimental controlled cohort, over 4 days	192 surface samples were taken from 3 different positions within one randomly selected area of a 1x1 m floor area in each of four two-bed patient rooms in the Department of Geriatrics at university hospital. Oslo, Norway	Dry mopping and moist mopping with no disinfectant were compared to spray mopping and wet mopping with Allrent (1-5% 2-propanol,1-5% tensides, 60-100% water). Samples were taken before and within 10 minutes after each surface area was cleaned	The mean bacterial count for all four methods decreased after cleaning (Dry: 85.1 CFU before to 35.4 CFU after, p=0.011; spray: 86.0 CFU before to 61.9 CFU after; moist: 67.4 CFU before to 25.7 CFU after, p=0.002; wet: 98.9 CFU before to 35.3 CFU, p=0.007) with significant reduction observed for dry, moist, and wet methods. Wet mopping reduced bacterial count by 64%. Wet mopping significantly reduced ATP bioluminescence compared to dry mopping (p<0.001) and spray mopping (p<0.011).

Reference (Study ID)	Outcome	Study Design	Setting	Intervention	Results
Attaway 2012 (621)	All viable organisms	Single-site, quasi-experimental, controlled before-after study over a period of 6 months	A total of 18 microbiological samples were collected from bedrails in 3 rounds of sampling of 6 patient rooms each; sampling was done in the MICU of a teaching hospital, SC, USA	Baseline samples were taken immediately prior to disinfection. Routine cleaning was done with two products: (1) 1:256 dilution of low-alcohol QAC (final concentration of 660 ppm 0.07% n-alkyl dimethyl benzyl ammonium chloride and 0.07% didecyl dimethyl ammonium chloride; Virex II 256; Diversey); (2) high-alcohol (17.2% isopropanol) QAC in a ready-to-use, pre-diluted spray bottle (final concentration: 0.28% diisobutyl phenoxyethoxyethyl dimethyl benzyl ammonium chloride; Cavicide; Metrex). Products had a wet application time of 30 minutes. Measurements were taken at 0.5, 2.5, 4.5, and 6.5 hours after cleaning.	Mean concentration before cleaning with low-alcohol QAC Virex II 256 was 3,711 CFU/100 cm2 compared to 2,057 CFU/100 cm2 at 30 minutes after cleaning, for a mean (median) relative reduction of 45% (95%). Within 2.5 hours after cleaning, mean concentration exceeded 250 CFU/100 cm2 (significance not specified). Mean concentration before cleaning with high-alcohol QAC CaviCide was 5,800 CFU/100 cm2 compared to 58 CFU/100 cm2 at 30 minutes after cleaning, for a mean (median) relative reduction of 99% (98%). Within 2.5 hours after cleaning, mean concentration exceeded 250 CFU/100 cm2 (significance not specified). The mean relative reduction of the bacterial population on bed rails after 30 minutes was significantly higher (p=0.017) for CaviCide (99%) than for Virex II 256 (45%).
Ferreira 2015 (997)	All viable organisms	Single-site, Quasi-experimental, uncontrolled before-after study over 4 weeks	320 samples from 5 surfaces (bed rails, bedside tables, infusion pumps, nurse's counter, prescription tables) in a medical-surgical ICU at a general hospital linked to the Brazilian Unified Health System (SUS). Tres Lagoas, Brazil	Samples were taken before and 10 minutes after disinfection with ethyl alcohol (70% w/v). Cloth was dampened with hydrated ethyl alcohol and each surface was three times for at least 15 seconds. ATP readings were measured, 10 minutes passed before the after samples	There were significant reductions in ATP (P<0.001) after cleaning on all surfaces when compared to the readings before cleaning. Average RLU ranged from 692 – 21850 before cleaning to 249 – 1712 after cleaning. Percent unacceptable (> 500 RLU) surfaces was 72.5% before cleaning compared to 20.6% after cleaning.
Fukada 2015 (1059)	All viable organisms	Single-site, Quasi-experimental, controlled before-after study	~240 samples from 5 surfaces (keyboard, mouse, APL valve, control knob, and syringe pump) in the anesthetist's working environment in an OR at a	Samples taken after 12 surgeries were completed were compared to samples taken approximately 30 minutes after post-surgery cleaning with either 76.9%-81.4% ethanol (Shodokku®	All surfaces had a reduction of the mean ATP bioluminescence before and after disinfection. For both ethanol and hydrogen peroxide disinfectants, two surfaces (mouse and control knob) had significantly lower ATP values (p<0.05). Average ATP among

Reference (Study ID)	Outcome	Study Design	Setting	Intervention	Results
			women's medical university. Tokyo, Japan.	Super) or accelerated hydrogen peroxide (6% hydrogen peroxide, <5% linear alkyaryl sulfonic acid, Hyprox Accele Wipes). Surfaces were allowed to dry. Unspecified contact time	5 surfaces disinfected with ethanol ranged from 691-5167 RLU before and 454-980 RLU after. For hydrogen peroxide, average ATP among 5 surfaces ranged from 573-2970 RLU before and 533 – 1311 RLU after disinfection. There was not a significant difference in the number of sites with > 500 RLU after disinfection between disinfectants.
Jones 2015 (1991)	All viable organisms	Single-site, Quasi-experimental, controlled cohort study over 16 days	399 samples from bedside keyboards in intensive care unit at NHS Foundation Trust hospital. Norfolk, UK	Intervention compared daily disinfection with chlorine dioxide spray (Tristel Fuse, 5 min contact time) to daily disinfection with 2% chlorhexidine gluconate spray (70% alcohol, Hydrex Pink, unspecified contact time). Samples were taken from keyboards 0 h, 4-6 h, and 24 h after they were sprayed with either CHG spray or Tristel Fuse spray. Baseline samples were taken before intervention and 2 weeks after CHG intervention only.	During the intervention period, mean ± standard deviation (median) concentration after each of the disinfectants increased over time, although CHG was more effective than Tristel Fuse at all time points ($p=0.002$). For CHG, concentration increased from 0±0 (0) CFU after 0 h to 0.13±43 (0) CFU after 4-6 h to 4.21±10.72 (0) CFU after 24 h. For chlorine-dioxide, concentration increased from 2.54±6.78 (0) CFU after 0 h to 7.75±14.90 (2) CFU after 4-6 h to 68.23±133.24 (7.5) CFU after 24 h. There was a 60-fold reduction in bacterial burden at 4-6 hours after chlorhexidine cleaning compared with Tristel Fuse, and a 16-fold reduction at 24. Baseline samples had significantly higher ($p<0.001$) median CFU count >500 compared to 2 weeks after CHG intervention with median of 0. hours.
Schmidt 2019 (2655)	All viable organisms	Single-site, quasi-experimental, controlled cohort study over an unspecified period of time	A total of 129 samples were taken from bedrails in 132 beds of the ICU of a teaching hospital in SC, USA	Baseline measurements were taken prior to disinfection. A trial product and persistent disinfectant containing 70% ethanol and <1% mixed QAC along with proprietary agents designed to increase longevity on surfaces (active QAC not specified; Firebird F130; Microban), was tested against	Trial product (Firebird F130) had significantly lower ($p<0.05$) median concentration after 1 h, 6 h, and 24 h after disinfection compared to before when compared to each of the control disinfectants. Each of the three disinfectants had significantly lower concentration after 1 h compared to before. Virex II 256 had significantly lower concentration ($p<0.05$) only after 1 h (135 CFU/100 cm^2) and not

Reference (Study ID)	Outcome	Study Design	Setting	Intervention	Results
				two control products. The first was a low-alcohol QAC solution (8.2% n-alkyl dimethyl benzyl ammonium chloride, 8.7% didecyl dimethyl ammonium chloride, and 2.9% ethyl alcohol; Virex II 256; Diversey) at an unspecified final dilution. The second was a high-alcohol QAC solution (17.2% isopropanol and 0.28% diisobutyl phenoxyethoxyethyl dimethyl benzyl ammonium chloride; CaviCide; Metrex) at an unspecified final dilution. All products were applied consistent with manufacturer instructions and allowed to air dry. Measurements were taken at 1, 6, and 24 hours post disinfection.	after 6 h (540 CFU/100 cm^2) and 24 h (735 CFU/100 cm^2) after disinfection compared to before (480 CFU/100 cm^2). CaviCide had significantly lower concentration ($p<0.05$) after 1 h (30 CFU/100 cm^2) and 6 h (450 CFU/100 cm^2), but not 24 h (630 CFU/100 cm^2) after disinfection compared to before (990 CFU/100 cm^2). The median bacterial burden following use of control was significantly higher at all three time points (for Virex II 256) and at 1 h and 6 h (for CaviCide) when compared to Firebird F130 ($p<0.05$).
Zubair 2018 (3699)	All viable organisms	Single-site, Quasi-experimental, uncontrolled before-after study, over 5 months	108 samples from surfaces (patient's bedside tables, patient's beds, nursing counters, door handles, walls, windows in hospital wards) at a children's hospital. Lahore, Pakistan	Samples were taken before and after surfaces were disinfected with 70% methanol (unspecified contact time). Sample collection relative to disinfection unspecified.	There was a 90.7% reduction in the percent surfaces positive for bacterial growth after disinfection (3/54, 5.5%) compared to before disinfection (52/54, 96.2%) were positive). Significance not specified.
Sui 2012 (4220)	All viable organisms	Single-site, Quasi-experimental, controlled cohort study	Samples from surfaces (faceplates, Y-pieces, and water traps) from 9 in-use ventilators in a 15-bed respiratory care center. Taipei City, Taiwan.	Samples were taken from all 9 surfaces 0.5 h, 8 h, and 24 h after initial disinfection with 0.5% sodium hypochlorite. Ventilators were randomly assigned to one of three groups 24 h after initial disinfection: disinfection with 75% alcohol aerosol with air drying (E1), disinfection with 75% alcohol with tissue trying (E2), and	Detection rate was not significantly different between control group and either of the alcohol disinfectants. There was not a significant difference between the alcohol disinfectant methods. Median total bacteria across the three study groups was 10-36 on faceplates, 146-> 500 on Y-pieces, and > 500 on water traps.

Reference (Study ID)	Outcome	Study Design	Setting	Intervention	Results
				control group with no second disinfection. Samples were taken at 0.5 h, 8 h, 24 after the second disinfection with ethanol.	
Alhmidi 2017 (6931)	All viable organisms	Single-site, Quasi-experimental, controlled before-after study	471 samples were collected from 100 hard surfaces (bed rails, beside tables, and physical therapy hand rails) and 57 soft surfaces (chairs, mattresses, and cushions) in hospital wards at veterans affair medical center. Ohio, USA	Control samples were taken 30 s after a surface was sprayed 5 times with sterile water compared to two disinfectants. Experimental samples were taken 30 s after surface was sprayed 5 times with 30% ethanol spray (Purell Healthcare Surface Disinfectant) or after surface was sprayed 5 times with 0.65% sodium hypochlorite spray (Clorox Healthcare Bleach Germicidal Cleaner).	Percent surfaces positive was significantly lower ($p < 0.01$) for surfaces sprayed with Purell at 2.5% and Clorox at 1.3% compared to control surfaces at 16.6%. There was not a significant difference between the two disinfectants.
Fukada 2008 (9254)	All viable organisms	Single-site, quasi-experimental, uncontrolled before-after study	Samples from the keys of all keyboards in the operating room at women's hospital. Tokyo, Japan. Total number of samples not specified.	Before (unspecified routine cleaning) compared to after disinfection with ethyl alcohol (concentration not specified). Samples were collected after healthcare procedure and after 1 hour after they were cleaned by a cotton cellulose sheet dampened with ethyl alcohol, unspecified contact time.	Mean concentration (standard deviation) was significantly higher ($p<0.05$) before cleaning at 300 CFU/ml) compared to after cleaning at 35 CFU/ml (67).
Codish 2015 (9825)	All viable organisms	Single-site, quasi-experimental, controlled cohort study over 2 weeks	A total of 324samples were taken from the keyboards and computer mice from 6 internal medicine wards and 2 at a large (>1000-bed) teaching hospital. Israel.	Baseline measurements were taken prior to decontamination. Simultaneous trials were run using two disinfectant products. Product 1 was an alcohol-free QAC wipe with proprietary ingredients (<1% polymeric biguanide hydrochloride, <1% alkyl dimethyl benzyl ammonium chloride, and dodecyl dimethyl ammonium	No. of rooms (%) with improved pathogenicity (i.e. more surfaces negative for high-risk pathogens after disinfection) was significantly ($p<0.001$) higher for alcohol-based disinfection at 32 rooms (42.1%) compared to alcohol-free QAC - based disinfection at 16 rooms (18.6%). Other pathogenicity categories were not different between products. A higher odds ratio (1.77, 95% CI, 1.36-2.89) of achieving lower pathogenicity by decontamination

Reference (Study ID)	Outcome	Study Design	Setting	Intervention	Results
				chloride; TriGene Advance; MediChem International). Product 2 was a wipe containing ethanol, 70% isopropyl alcohol, 0.5% chlorhexidine, and 0.45% hydrogen peroxide 0.45% (MEDIWIPES; Albaad). Wet contact time, time to measurement not specified. Surfaces were decontaminated three times a day. Samples were collected 2 weeks after the beginning of the trial.	was associated with alcohol-based decontamination when compared to quaternary ammonium-based decontamination ($p<0.001$)
Biswal 2017 (12894)	Fungi (*Candida auris*)	Single-site, Quasi-experimental uncontrolled before-after study, 3 months total	ECG leads and blood pressure monitoring cuffs in the trauma ICU of a tertiary care multi-specialty hospital. Chandigarh, India.	Surveillance was conducted to stop *Candida auris* outbreak. Surfaces were disinfected with 70% alcohol and left to dry. 10 patients admitted after the disinfection were screened for C. auris daily. Surface sample number not specified.	All 10 patients acquired C. auris yeast at one or more sites by the fourth day of being in the ICU after all 10 were found to have no colonization on the day of admission. Surfaces were found to be contaminated and disinfection was deemed suboptimal.
Evans 2007 (9347)	Gram-negative bacteria	Single-site, Quasi-experimental, controlled before-after study	5 treatment tables from chiropractic outpatient teaching facility. Texas, USA.	Each table received two sterilizing agents: the left half received treatment with a pre-packaged alcohol wipe containing 70% isopropyl alcohol and 10% acetone while the right side received treatment with QAC (Lysol Brand, <1% 80% benzalkonium chloride) sanitizing wipes. Once treated, each side was allowed to completely dry before a sample was taken. Baseline samples were taken prior to disinfection.	Prior to disinfection, two of five tables were positive for gram-negative organisms. After disinfection, none reported. Bacterial counts were not reported.
Doidge 2010 (12244)	Gram-negative bacteria (*Acinetobacter*	Single-site, Quasi-experimental,	149 total surface samples were taken from environmental sites	Contamination of environmental sites with CRAB before initial intervention (unspecified routine	Before the initial intervention CRAB was recovered from 11 of 137 environmental sites sampled, after the intervention CRAB

Reference (Study ID)	Outcome	Study Design	Setting	Intervention	Results
	baumannii-carbapenem resistant (CRAB))	uncontrolled before-after study over one month	(mattress, vital signs monitor, horizontal surfaces at patient bedside, computer keyboard, glucometer) in a 19-bed long-stay ICU at a women's hospital. Brisbane, Australia	cleaning) is compared to contamination of environmental sites with CRAB after the ward was closed for 3 days for cleaning 3 times a day with a 1% neutral detergent water detergent and then cleaning with 70% alcohol-impregnated wipes (>1 minute for contact time)	was recovered from 2 of 12 environmental sample cultures from horizontal surfaces at the patient bedside
Zubair 2018 (3699)	Gram-negative bacteria (*Acinetobacter* spp.)	Single-site, Quasi-experimental, uncontrolled before-after study, over 5 months	108 samples from surfaces (patient's bedside tables, patient's beds, nursing counters, door handles, walls, windows in hospital wards) at a children's hospital. Lahore, Pakistan	Samples were taken before and after surfaces were disinfected with 70% methanol (unspecified contact time). Sample collection relative to disinfection unspecified.	*Acinetobacter* spp. was isolated from fewer samples after disinfection (1/54 samples) compared to before disinfection (10/54 samples). Significance not specified.
Bokulich 2013 (6368)	Gram-negative bacteria (*Acinetobacter* spp.)	Single-site, quasi-experimental, uncontrolled study over a 5-month period	A total of 128 samples were collected from isolettes, radiant warmers, and ventilators, in an unspecified number of rooms in the NICU of a children's hospital in the USA	Routine cleaning twice daily (control) was done with high-alcohol wipes containing 55% isopropyl alcohol and 0.5% QAC (0.25% n-alkyl dimethyl ethylbenzyl ammonium chloride, 0.25% n-alkyl dimethyl benzyl ammonium chloride; Super Sani-Cloth; PDI); this was compared to intensive cleaning (trial) with alcohol-free QAC (13% n-alkyl dimethyl ethylbenzyl ammonium chloride and 13% n-alkyl dimethyl benzyl ammonium chloride; HB Quat Disinfectant Cleaner Concentrate 25H; 3M) on soft cloth, dilution not specified. Contact times and time until measurement were not specified. Samples were taken before (control) and after intensive cleaning (trial).	Mean relative abundance ± standard deviation unchanged, from 0.0655 ± 0.0240 to 0.0696 ± 0.0199 following intensive cleaning ($p>0.1$).

Reference (Study ID)	Outcome	Study Design	Setting	Intervention	Results
Zubair 2018 (3699)	Gram-negative bacteria (*Citrobacter* spp.)	Single-site, Quasi-experimental, uncontrolled before-after study, over 5 months	108 samples from surfaces (patient's bedside tables, patient's beds, nursing counters, door handles, walls, windows in hospital wards) at a children's hospital. Lahore, Pakistan	Samples were taken before and after surfaces were disinfected with 70% methanol (unspecified contact time). Sample collection relative to disinfection unspecified.	*Citrobacter* spp. was isolated from fewer samples after disinfection (0/54 samples) compared to before disinfection (5/54 samples). Significance not specified.
Zubair 2018 (3699)	Gram-negative bacteria (*Escherichia* coli)	Single-site, Quasi-experimental, uncontrolled before-after study, over 5 months	108 samples from surfaces (patient's bedside tables, patient's beds, nursing counters, door handles, walls, windows in hospital wards) at a children's hospital. Lahore, Pakistan	Samples were taken before and after surfaces were disinfected with 70% methanol (unspecified contact time). Sample collection relative to disinfection unspecified.	*E. coli* was isolated from fewer samples after disinfection (0/54 samples) compared to before disinfection (5/54 samples). Significance not specified.
Bokulich 2013 (6368)	Gram-negative bacteria (*Escherichia* spp.)	Single-site, quasi-experimental, uncontrolled study over a 5-month period	A total of 128 samples were collected from isolettes, radiant warmers, and ventilators, in an unspecified number of rooms in the NICU of a children's hospital in the USA	Routine cleaning twice daily (control) was done with high-alcohol wipes containing 55% isopropyl alcohol and 0.5% QAC (0.25% n-alkyl dimethyl ethylbenzyl ammonium chloride, 0.25% n-alkyl dimethyl benzyl ammonium chloride; Super Sani-Cloth; PDI); this was compared to intensive cleaning (trial) with alcohol-free QAC (13% n-alkyl dimethyl ethylbenzyl ammonium chloride and 13% n-alkyl dimethyl benzyl ammonium chloride; HB Quat Disinfectant Cleaner Concentrate 25H; 3M) on soft cloth, dilution not specified. Contact times and time until measurement not specified. Samples were taken before (control) and after intensive cleaning (trial).	Mean relative abundance ± standard deviation was unchanged from 0.0003 ± 0.0005 to 0.0004 ± 0.0006 following disinfection ($p>0.1$).

Reference (Study ID)	Outcome	Study Design	Setting	Intervention	Results
Alhmidi 2017 (6931)	Gram-negative bacteria (Gram-negative bacilli)	Single-site, Quasi-experimental, controlled before-after study	471 samples were collected from 100 hard surfaces (bed rails, beside tables, and physical therapy hand rails) and 57 soft surfaces (chairs, mattresses, and cushions) in hospital wards at veteran's affair medical center. Ohio, USA	Control samples were taken 30 s after a surface was sprayed 5 times with sterile water compared to two disinfectants. Experimental samples were taken 30 s after surface was sprayed 5 times with 30% ethanol spray (Purell Healthcare Surface Disinfectant) or after surface was sprayed 5 times with 0.65% sodium hypochlorite spray (Clorox Healthcare Bleach Germicidal Cleaner).	Percent surfaces positive was lower (p=0.07) for surfaces sprayed with Purell at 0.6% and Clorox at 0.6% compared to control surfaces at 4.5%. There was not a significant difference between the two disinfectants.
Zubair 2018 (3699)	Gram-negative bacteria (*Klebsiella* spp.)	Single-site, Quasi-experimental, uncontrolled before-after study, over 5 months	108 samples from surfaces (patient's bedside tables, patient's beds, nursing counters, door handles, walls, windows in hospital wards) at a children's hospital. Lahore, Pakistan	Samples were taken before and after surfaces were disinfected with 70% methanol (unspecified contact time). Sample collection relative to disinfection unspecified.	*Klebsiella* spp. was not isolated from any samples after disinfection (0/54) compared to 22.4% (15/54) before disinfection. Significance not specified.
Bokulich 2013 (6368)	Gram-negative bacteria (*Klebsiella* spp.)	Single-site, quasi-experimental, uncontrolled study over a 5-month period	A total of 128 samples were collected from isolettes, radiant warmers, and ventilators, in an unspecified number of rooms in the NICU of a children's hospital in the USA	Routine cleaning twice daily (control) was done with high-alcohol wipes containing 55% isopropyl alcohol and 0.5% QAC (0.25% n-alkyl dimethyl ethylbenzyl ammonium chloride, 0.25% n-alkyl dimethyl benzyl ammonium chloride; Super Sani-Cloth; PDI); this was compared to intensive cleaning (trial) with alcohol-free QAC (13% n-alkyl dimethyl ethylbenzyl ammonium chloride and 13% n-alkyl dimethyl benzyl ammonium chloride; HB Quat Disinfectant Cleaner Concentrate 25H; 3M) on soft cloth, dilution not specified. Contact times and	Mean relative abundance ± standard deviation was unchanged from 0.0005 ± 0.0003 to 0.0005 ± 0.0004 following disinfection (p>0.1).

Reference (Study ID)	Outcome	Study Design	Setting	Intervention	Results
				time until measurement not specified. Samples were taken before (control) and after intensive cleaning (trial).	
Zubair 2018 (3699)	Gram-negative bacteria (*Proteus* spp.)	Single-site, Quasi-experimental, uncontrolled before-after study, over 5 months	108 samples from surfaces (patient's bedside tables, patient's beds, nursing counters, door handles, walls, windows in hospital wards) at a children's hospital. Lahore, Pakistan	Samples were taken before and after surfaces were disinfected with 70% methanol (unspecified contact time). Sample collection relative to disinfection unspecified.	*Proteus* spp. was isolated from fewer samples after disinfection (0/54 samples) compared to before disinfection (1/54 samples). Significance not specified.
Sui 2012 (4220)	Gram-negative bacteria (*Pseudomonas aeruginosa*)	Single-site, Quasi-experimental, controlled cohort study	Samples from surfaces (faceplates, Y-pieces, and water traps) from 9 in-use ventilators in a 15-bed respiratory care center. Taipei City, Taiwan.	Samples were taken from all 9 surfaces 0.5 h, 8 h, and 24 h after initial disinfection with 0.5% sodium hypochlorite. Ventilators were randomly assigned to one of three groups 24 h after initial disinfection: disinfection with 75% alcohol aerosol with air drying (E1), disinfection with 75% alcohol with tissue trying (E2), and control group with no second disinfection. Samples were taken at 0.5 h, 8 h, 24 after the second disinfection with ethanol.	The *Pseudomonas aeruginosa* detection rate on faceplates was 0% for all groups. There was no difference in detection (p>0.05) on Y-pieces. Group E1 (air-dry) had higher concentration on water traps compared to control (median 24 compared to median of 0 CFU).
Zubair 2018 (3699)	Gram-negative bacteria (*Pseudomonas* spp.)	Single-site, Quasi-experimental, uncontrolled before-after study, over 5 months	108 samples from surfaces (patient's bedside tables, patient's beds, nursing counters, door handles, walls, windows in hospital wards) at a children's hospital. Lahore, Pakistan	Samples were taken before and after surfaces were disinfected with 70% methanol (unspecified contact time). Sample collection relative to disinfection unspecified.	*Pseudomonas* spp. was isolated from fewer samples after disinfection (1/54 samples) compared to before disinfection (2/54 samples). Significance not specified.
Bokulich 2013 (6368)	Gram-negative bacteria (*Pseudomonas* spp.)	Single-site, quasi-experimental, uncontrolled study over a	A total of 128 samples were collected from isolettes, radiant warmers, and ventilators, in an unspecified number of	Routine cleaning twice daily (control) was done with high-alcohol wipes containing 55% isopropyl alcohol and 0.5% QAC (0.25% n-alkyl dimethyl	Mean relative abundance ± standard deviation significantly increased from 0.0166 ± 0.0054 to 0.0199 ± 0.0065 following disinfection (p=0.023).

Reference (Study ID)	Outcome	Study Design	Setting	Intervention	Results
		5-month period	rooms in the NICU of a children's hospital in the USA	ethylbenzyl ammonium chloride, 0.25% n-alkyl dimethyl benzyl ammonium chloride; Super Sani-Cloth; PDI); this was compared to intensive cleaning (trial) with alcohol-free QAC (13% n-alkyl dimethyl ethylbenzyl ammonium chloride and 13% n-alkyl dimethyl benzyl ammonium chloride; HB Quat Disinfectant Cleaner Concentrate 25H; 3M) on soft cloth, dilution not specified. Contact times and time until measurement not specified. Samples were taken before (control) and after intensive cleaning (trial).	
Zubair 2018 (3699)	Gram-positive bacilli (Bacillus spp.)	Single-site, Quasi-experimental, uncontrolled before-after study, over 5 months	108 samples from surfaces (patient's bedside tables, patient's beds, nursing counters, door handles, walls, windows in hospital wards) at a children's hospital. Lahore, Pakistan	Samples were taken before and after surfaces were disinfected with 70% methanol (unspecified contact time). Sample collection relative to disinfection unspecified.	Bacillus spp. was isolated from fewer samples after disinfection (1/54 samples) compared to before disinfection (3/54 samples). Significance not specified.
Alhmidi 2017 (6931)	Gram-positive cocci (*Enterococcus* spp.-VRE)	Single-site, Quasi-experimental, controlled before-after study	471 samples were collected from 100 hard surfaces (bed rails, beside tables, and physical therapy hand rails) and 57 soft surfaces (chairs, mattresses, and cushions) in hospital wards at veterans affair medical center. Ohio, USA	Control samples were taken 30 s after a surface was sprayed 5 times with sterile water compared to two disinfectants. Experimental samples were taken 30 s after surface was sprayed 5 times with 30% ethanol spray (Purell Healthcare Surface Disinfectant) or after surface was sprayed 5 times with 0.65% sodium hypochlorite spray (Clorox Healthcare Bleach Germicidal Cleaner).	Percent surfaces positive for VRE was lower (p=0.07) for surfaces sprayed with Purell at 0.6% and Clorox at 0.6% compared to control surfaces at 4.5%. There was not a significant difference between the two disinfectants.

Reference (Study ID)	Outcome	Study Design	Setting	Intervention	Results
Bokulich 2013 (6368)	Gram-positive cocci (*Enterococcus* spp.)	Single-site, quasi-experimental, uncontrolled study over a 5-month period	A total of 128 samples were collected from isolettes, radiant warmers, and ventilators, in an unspecified number of rooms in the NICU of a children's hospital in the USA	Routine cleaning twice daily (control) was done with high-alcohol wipes containing 55% isopropyl alcohol and 0.5% QAC (0.25% n-alkyl dimethyl ethylbenzyl ammonium chloride, 0.25% n-alkyl dimethyl benzyl ammonium chloride; Super Sani-Cloth; PDI); this was compared to intensive cleaning (trial) with alcohol-free QAC (13% n-alkyl dimethyl ethylbenzyl ammonium chloride and 13% n-alkyl dimethyl benzyl ammonium chloride; HB Quat Disinfectant Cleaner Concentrate 25H; 3M) on soft cloth, dilution not specified. Contact times and time until measurement not specified. Samples were taken before (control) and after intensive cleaning (trial).	Mean relative abundance ± standard deviation was unchanged from 0.0006 ± 0.0019 to 0.0007 ± 0.0020 following disinfection ($p>0.1$).
Evans 2007 (9347)	Gram-positive cocci (Gram-positive bacteria)	Single-site, Quasi-experimental, controlled before-after study	5 treatment tables from chiropractic outpatient teaching facility. Texas, USA.	Each table received two sterilizing agents: the left half received treatment with a pre-packaged alcohol wipe containing 70% isopropyl alcohol and 10% acetone while the right side received treatment with QAC (Lysol Brand, <1% 80% benzalkonium chloride) sanitizing wipes. Once treated, each side was allowed to completely dry before a sample was taken. Baseline samples were taken prior to disinfection.	Prior to disinfection, all (n=5) tables were positive for gram-positive organisms including *Staphylococcus* spp. After disinfection, no *Staphylococcus* spp or MRSA were reported. Bacterial counts were not reported.

Reference (Study ID)	Outcome	Study Design	Setting	Intervention	Results
Zubair 2018 (3699)	Gram-positive cocci (*Micrococcus* spp.)	Single-site, Quasi-experimental, uncontrolled before-after study, over 5 months	108 samples from surfaces (patient's bedside tables, patient's beds, nursing counters, door handles, walls, windows in hospital wards) at a children's hospital. Lahore, Pakistan	Samples were taken before and after surfaces were disinfected with 70% methanol (unspecified contact time). Sample collection relative to disinfection unspecified.	*Micrococcus* was isolated from fewer samples after disinfection (0/54 samples) compared to before disinfection (2/54 samples). Significance not specified.
Ferreira 2015 (997)	Gram-positive cocci (*Staphylococcus aureus*-MRSA)	Single-site, Quasi-experimental, uncontrolled before-after study over 4 weeks	320 samples from 5 surfaces (bed rails, bedside tables, infusion pumps, nurse's counter, prescription tables) in a medical-surgical ICU at a general hospital linked to the Brazilian Unified Health System (SUS). Tres Lagoas, Brazil	Samples were taken before and 10 minutes after disinfection with ethyl alcohol (70% w/v). Cloth was dampened with hydrated ethyl alcohol and each surface was three times for at least 15 seconds. ATP readings were measured, 10 minutes passed before the after samples	There was a significant reduction ($p<0.05$) in the percent of samples positive for MRSA after cleaning at 9% (14/160) compared to before cleaning at 22% (35/160).
Alhmidi 2017 (6931)	Gram-positive cocci (*Staphylococcus aureus*-MRSA)	Single-site, Quasi-experimental, controlled before-after study	471 samples were collected from 100 hard surfaces (bed rails, beside tables, and physical therapy hand rails) and 57 soft surfaces (chairs, mattresses, and cushions) in hospital wards at veterans affair medical center. Ohio, USA	Control samples were taken 30 s after a surface was sprayed 5 times with sterile water compared to two disinfectants. Experimental samples were taken 30 s after surface was sprayed 5 times with 30% ethanol spray (Purell Healthcare Surface Disinfectant) or after surface was sprayed 5 times with 0.65% sodium hypochlorite spray (Clorox Healthcare Bleach Germicidal Cleaner).	Percent surfaces positive was significantly lower ($p < 0.01$) for surfaces sprayed with Purell at 1.3% and Clorox at 0.0% compared to control surfaces at 7.6%. There was not a significant difference between the two disinfectants.
Oie 2005 (11015)	Gram-positive cocci (*Staphylococcus aureus*-MRSA)	Single-site, quasi-experimental, controlled before-after study. 3-month period.	32 samples were taken from smooth, non-porous surfaces (immersion bathtub, foot washbowl, examination tables) in a 37-bed dermatological ward in the hydrotherapy unit and ointment treatment unit of a	Baseline sampling before disinfection was compared to sampling non-porous surfaces after two disinfection methods: wiping with 0.2% solution (alkyldiaminoethyl glycine, Tego-51) or wiping with 80% ethyl alcohol (Kenei Pharm).	Mean (standard deviation) MRSA count before disinfection ranged from 48 (119) to 7366 (16555) CFU to no detection after QAC or ethyl alcohol disinfection.

Reference (Study ID)	Outcome	Study Design	Setting	Intervention	Results
			university hospital. Yamaguchi, Japan.	Contact time for QAC was 10 min; unspecified contact time for alcohol. This study compared efficacy among porous and non-porous surfaces.	
Fujii 1996 (11965)	Gram-positive cocci (*Staphylococcus aureus*-MRSA)	Single-site, quasi-experimental, controlled before-after study.	An unstated number of samples were taken from the floors of patient rooms in a neurosurgery ward at a university hospital. Yamaguchi, Japan.	Baseline measurements were taken prior to mopping. Floors were mopped with QAC benzalkonium chloride (Osvan) in concentrations of 0.2% and 0.5%. Product was compared to hydrochloride solution of alkyldiaminoethyl glycine (zwitterionic surfactant; Tego-51) at 0.2% concentration, as well as chlorhexidine digluconate (Hibitane) in concentrations of 0.2% and 0.5%. This was a cohort study and no products were designated as the control. Contact time, time until measurement after disinfection were not specified.	MRSA was detected following disinfection with alkyldiaminioethyl glycine, 0.2% chlorhexidine digluconate , and 0.2% benzalkonium chloride; it was not detected when concentration was increased to 0.5% benzalkonium chloride and 0.5% chlorhexidine digluconate (significance not specified).
Oie 2005 (11015)	Gram-positive cocci (*Staphylococcus aureus*-MSSA)	Single-site, quasi-experimental, controlled before-after study. 3-month period.	32 samples were taken from smooth, non-porous surfaces (immersion bathtub, foot washbowl, examination tables) in a 37-bed dermatological ward in the hydrotherapy unit and ointment treatment unit of a university hospital. Yamaguchi, Japan.	Baseline sampling before disinfection was compared to sampling non-porous surfaces after two disinfection methods: wiping with 0.2% solution (alkyldiaminoethyl glycine, Tego-51) or wiping with 80% ethyl alcohol (Kenei Pharm). Contact time for QAC was 10 min; unspecified contact time for alcohol. This study compared efficacy among porous and non-porous surfaces.	Mean (standard deviation) MSSA bacterial count before disinfection ranged from 6.5 (16) to 13897 (37721) CFU to no detection after QAC or ethyl alcohol disinfection .
Ferreira 2015 (997)	Gram-positive cocci	Single-site, Quasi-	320 samples from 5 surfaces (bed rails, bedside	Samples were taken before and 10 minutes after disinfection	There were significantly lower surfaces positive for S. *aureus* after cleaning

Reference (Study ID)	Outcome	Study Design	Setting	Intervention	Results
	(*Staphylococcus aureus*)	experimental, uncontrolled before-after study over 4 weeks	tables, infusion pumps, nurse's counter, prescription tables) in a medical-surgical ICU at a general hospital linked to the Brazilian Unified Health System (SUS). Tres Lagoas, Brazil	with ethyl alcohol (70% w/v). Cloth was dampened with hydrated ethyl alcohol and each surface was three times for at least 15 seconds. ATP readings were measured, 10 minutes passed before the after samples	compared to before on all surfaces except one (nursing tables, p=0.072). Percent surfaces positive was 42.5% (92/160) before cleaning compared to 12.5% after cleaning.
Zubair 2018 (3699)	Gram-positive cocci (*Staphylococcus aureus*)	Single-site, Quasi-experimental, uncontrolled before-after study, over 5 months	108 samples from surfaces (patient's bedside tables, patient's beds, nursing counters, door handles, walls, windows in hospital wards) at a children's hospital. Lahore, Pakistan	Samples were taken before and after surfaces were disinfected with 70% methanol (unspecified contact time). Sample collection relative to disinfection unspecified.	S. *aureus* was isolated from fewer samples after disinfection (0/54 samples) compared to before disinfection (9/54 samples). Significance not specified.
Sui 2012 (4220)	Gram-positive cocci (*Staphylococcus aureus*)	Single-site, Quasi-experimental, controlled cohort study	Samples from surfaces (faceplates, Y-pieces, and water traps) from 9 in-use ventilators in a 15-bed respiratory care center. Taipei City, Taiwan.	Samples were taken from all 9 surfaces 0.5 h, 8 h, and 24 h after initial disinfection with 0.5% sodium hypochlorite. Ventilators were randomly assigned to one of three groups 24 h after initial disinfection: disinfection with 75% alcohol aerosol with air drying (E1), disinfection with 75% alcohol with tissue trying (E2), and control group with no second disinfection. Samples were taken at 0.5 h, 8 h, 24 after disinfection with ethanol.	Percent surfaces positive was significantly lower with 75% alcohol compared to control on faceplates and Y-pieces. 75% ethanol with air drying had significantly lower bacterial concentration and percent surfaces positive compared to 75% ethanol with tissue drying (for faceplates p<0.001, for Y-pieces, p=0.01). Concentration on surfaces of faceplates (4 CFU, E2 group; 16 CFU, control) and Y-pieces (20 CFU, E2 group; 12 CFU, control) were significantly higher than E1 group (alcohol with air-dry) (0 CFU, 0 CFU)
Zubair 2018 (3699)	Gram-positive cocci (*Staphylococcus* spp.)	Single-site, Quasi-experimental, uncontrolled before-after study, over 5 months	108 samples from surfaces (patient's bedside tables, patient's beds, nursing counters, door handles, walls, windows in hospital wards) at a children's hospital. Lahore, Pakistan	Samples were taken before and after surfaces were disinfected with 70% methanol (unspecified contact time). Sample collection relative to disinfection unspecified.	Coagulase-negative Staphylococci were isolated from fewer samples after disinfection (0/54 samples) compared to before disinfection (15/54 samples). Significance not specified.

Reference (Study ID)	Outcome	Study Design	Setting	Intervention	Results
Bokulich 2013 (6368)	Gram-positive cocci (*Staphylococcus* spp.)	Single-site, quasi-experimental, uncontrolled study over a 5-month period	A total of 128 samples were collected from isolettes, radiant warmers, and ventilators, in an unspecified number of rooms in the NICU of a children's hospital in the USA	Routine cleaning twice daily (control) was done with high-alcohol wipes containing 55% isopropyl alcohol and 0.5% QAC (0.25% n-alkyl dimethyl ethylbenzyl ammonium chloride, 0.25% n-alkyl dimethyl benzyl ammonium chloride; Super Sani-Cloth; PDI); this was compared to intensive cleaning (trial) with alcohol-free QAC (13% n-alkyl dimethyl ethylbenzyl ammonium chloride and 13% n-alkyl dimethyl benzyl ammonium chloride; HB Quat Disinfectant Cleaner Concentrate 25H; 3M) on soft cloth, dilution not specified. Contact times and time until measurement not specified. Samples were taken before (control) and after intensive cleaning (trial).	Mean relative abundance ± standard deviation decreased from 0.0549 ± 0.0804 to 0.0353 ± 0.0671 following disinfection (p=0.072).
Bokulich 2013 (6368)	Gram-positive cocci (*Streptococcus* spp.)	Single-site, quasi-experimental, uncontrolled study over a 5-month period	A total of 128 samples were collected from isolettes, radiant warmers, and ventilators, in an unspecified number of rooms in the NICU of a children's hospital in the USA	Routine cleaning twice daily (control) was done with high-alcohol wipes containing 55% isopropyl alcohol and 0.5% QAC (0.25% n-alkyl dimethyl ethylbenzyl ammonium chloride, 0.25% n-alkyl dimethyl benzyl ammonium chloride; Super Sani-Cloth; PDI); this was compared to intensive cleaning (trial) with alcohol-free QAC (13% n-alkyl dimethyl ethylbenzyl ammonium chloride and 13% n-alkyl dimethyl benzyl ammonium chloride; HB Quat Disinfectant Cleaner Concentrate 25H; 3M)	Mean relative abundance ± standard deviation significantly decreased from 0.0250 ± 0.0445 to 0.0119 ± 0.0217 following disinfection (p=0.0022).

Reference (Study ID)	Outcome	Study Design	Setting	Intervention	Results
				on soft cloth, dilution not specified. Contact times and time until measurement not specified. Samples were taken before (control) and after intensive cleaning (trial).	
Biswal 2017 (12894)	HAI (HAI – C. auris)	Single-site, Quasi-experimental, 3 months total	ECG leads and blood pressure monitoring cuffs in the trauma ICU of a tertiary care multi-specialty hospital. Chandigarh, India.	Surveillance was conducted to stop *Candida auris* outbreak. Surfaces were disinfected with 70% alcohol and left to dry. 10 patients admitted after the disinfection were screened for C. auris daily. Surface sample number not specified.	All 10 patients acquired C. auris yeast at one or more sites by the fourth day of being in the ICU after all 10 were found to have no colonization on the day of admission. Surfaces were found to be contaminated and disinfection was deemed suboptimal.
Reynolds 2019 (3084)	Virus (MS2 bacteriophage)	Single-site, Quasi-experimental, uncontrolled before-after study over 6 h	Samples were taken from 19 high-touch surfaces (bathroom door handle, bathroom faucet, computer mouse, waiting room counter, patient seat arm, nurse station chair arm, patient room countertop, patient room door handle) in an outpatient, urgent care clinic. Arizona, USA	After seeding a viral tracer MS2 bacteriophage onto patient door handle and front desk pen, high-touch surfaces were cleaned with ethanol-based spray disinfectant (Purell Surface Disinfectant, 29.4% ethanol). Surfaces were sprayed until thoroughly wet for 30 s and wiped with dry paper towels. Samples were collected 2 h after cleaning and at baseline (before disinfection and 6 h after seeding of viral tracer).	The geometric mean viral count was 94.1% lower (95% CI: 71.4 -98.8; p = 0.001) for the spray disinfectant compared to the baseline. Virus concentrations decreased on most surfaces after disinfection except for the bathroom door handle and the bathroom faucet. Mean concentration was ~60 PFU/cm^2 at baseline compared to < 1 PFU/cm^2 after intervention.

Table S12: Study results for manually applied peroxygen interventions ordered by outcome organism

Reference (Study ID)	Outcome	Study Design	Setting	Intervention	Results
Carling 2014 (111)	All viable organisms	Single-site, quasi-experimental, controlled cohort study over several months	A total of 571 samples were collected from 12 high-touch surfaces (door, bedrail, call button, telephone, tray table, room chair, bathroom inner door, bathroom light switch, bathroom sink, bathroom grab bar, toilet handle, and toilet seat) in 48 rooms of an ICU of a general acute care hospital in the USA	Baseline samples were taken prior to disinfection (before) with one of two disinfectants: (1) low-alcohol QAC (final dilution: 0.10% octyl decyl dimethyl ammonium chloride, 0.04% dioctyl dimethyl ammonium chloride, 0.06% didecyl dimethyl ammonium chloride, and 0.14% alkyl dimethyl benzyl ammonium chloride; Quaternary Disinfectant Cleaner; Ecolab); (2) a product containing peracetic acid and hydrogen peroxide (Oxycide, 0.63% hydrogen peroxide, 0.13% peroxyactic acid). Disinfectant applied with pre-saturated cloth (after). Wet contact times, time until measurement not specified.	Median bioburden was 17 CFU/slide for QAC surfaces and 15 CFU/slide for Oxycide surfaces (before). The disinfection intervention (after) had complete removal of bacterial bioburden in 40% (93/237) of samples with QAC and 77% (211/274) with Oxycide (significance not specified). Hydrogen peroxide/peracetic acid disinfectant was 1.93 times more effective at removing bacteria compared to QAC ($p<0.001$).
Dharan 1999 (393)	All viable organisms	Single-site, Quasi-experimental, controlled cohort study for a 4-month period.	A total of 1356 surface (floors, furniture) samples were collected weekly in patient areas from two wings of a 106-bed medical unit at a tertiary-care hospital. Geneva, Switzerland.	Routine cleaning and disinfection was compared for floors, furniture, and bathroom/toilet floors on two wards using a QAC (0.5% ISEQUAT®, unknown active ingredients), detergent (1% TASKI® R50), and/or active oxygen based (AOB) compound (1% PERFORM, pentapotassium-bis-(peroxymonosulphate) bis(sulphate). Weekly samples were taken after surfaces were dry (10-15 minutes later).	For ward floors, using QAC had minimal decrease in bacterial count with average decrease 0.6 CFU/24 cm2; 95% CI: 26-27) compared to detergent which introduced bacteria averaging additional 103.6 CFU/24 cm2, 95% CI: 73–134. Active oxygen-based compound had a larger reduction averaging 111.1 CFU/24 cm2 (CI 95 87-133). Similar results were seen for bathroom/toilet floors. The AOB was significantly more effective than the QAC on floors ($p<0.001$).
Armellino 2020 (606)	All viable organisms	Single-site, quasi-experimental, controlled before-after study prospective study	3,300 samples were collected from 165 equipment surfaces (anesthesia carts, medical carts, Bovie machines, etc.) from 6 operating rooms at a 222-bed community	Standard terminal disinfection protocol consisted of 1-step EPA-registered hydrogen peroxide wipe on equipment surfaces (contact time, concentration not specified). A separate arm of the study used focused multivector ultraviolet (FUMV) light as the disinfection. Sampling was conducted as soon as operating room was available after standard cleaning and	There was significant reduction after disinfection in four of six operating rooms ($p<0.05$) with significant reduction ($p<0.001$) in average count per operating room from 87 CFU to 54 CFU (38.4% reduction). There was also a significant reduction ($p<0.001$) in total count per object from 2,871

Reference (Study ID)	Outcome	Study Design	Setting	Intervention	Results
			hospital. New York, USA.	again after manual disinfection or FUMV disinfection.	CFU to 1,769 CFU. Simultaneous FUMV arm displayed 96.5% reduction (p<0.001) with average count per operating room from 79 CFU to 2.73 CFU.
Boyce 2017 (808)	All viable organisms	Single-site, quasi-experimental controlled crossover study; 12-months	1061 samples prior to cleaning & 1092 samples after cleaning from 5-8 high-touch surfaces (bedside rails, remote control module, overbed tables, toilet seats, toilet grab bars, counters, supply cart keyboards, and workstations on wheels) from 2 general wards in a MICU at Yale New-Haven Hospital in New Haven. Connecticut, USA.	Daily and discharge cleaning using QAC disinfectant (Hyperfect 256; Genesan, Gorham, ME) with dry wipes made of melt blown polypropylene. 12-month crossover trial compared QAC with 0.5% improved hydrogen peroxide (IHP) disinfectant. Contact time was not specified. Monthly measurements were taken at unspecified time relative to disinfection.	Mean aerobic colony count per high-touch surface after cleaning was significantly higher (p=0.003) with QAC at 22.2 CFU compared to 14.0 CFU with improved hydrogen peroxide. Percent surfaces with no growth after cleaning with QAC was significantly lower at 35% (182/517) compared to 48% (240/500) with IHP (p < 0.001). Percent surfaces with < 2.5 CFU/cm2 was significantly lower with QAC at 88.4% (457/517) compared to IHP at 92.4% (462/500) (p=0.03).
Boyce 2013 (809)	All viable organisms	Single-site quasi experimental uncontrolled before-after study over unspecified amount of time	704 samples from 10 high-touch surfaces (bedside rails, over-bed table, television remote control, telephone, bedside panel, chair arm, blood pressure cuff, toilet seat, grab bar, faucet handles) in 72 patient rooms at a 500-bed university hospital. CT, USA	Convenience samples were taken before and after cleaning with a hydrogen peroxide wipe (activated hydrogen peroxide, Clorox Healthcare). Pre-cleaning not specified. Samples collected before and after cleaning with measurement time not specified.	The median count before disinfection was 63.1 CFU compared to 0 CFU after disinfection. Percent of surfaces with no growth was 75% (528/704) high-touch surfaces after disinfection, ranging from 53%-89% depending on surface type. Percent of surfaces with counts <2.5 CFU/cm2 was 99% (698/704) and with counts < 0.4 CFU/cm2 was 96% (679/704) after disinfection. Percent of surfaces with ATP bioluminescence < 250 RLU was 69.7% (388/557) after disinfection.

Reference (Study ID)	Outcome	Study Design	Setting	Intervention	Results
Fukada 2015 (1059)	All viable organisms	Single-site, Quasi-experimental, controlled before-after study	~240 samples from 5 surfaces (keyboard, mouse, APL valve, control knob, and syringe pump) in the anesthetist's working environment in an OR at a women's medical university. Tokyo, Japan.	Samples taken after 12 surgeries were completed were compared to samples taken approximately 30 minutes after post-surgery cleaning with either 76.9%-81.4% ethanol (Shodokku® Super) or accelerated hydrogen peroxide (6% hydrogen peroxide, <5% linear alkyaryl sulfonic acid, Hyprox Accele Wipes). Surfaces were allowed to dry. Unspecified contact time	All surfaces had a reduction of the mean ATP bioluminescence before to after disinfection. For both ethanol and hydrogen peroxide disinfectants, two surfaces (mouse and control knob) had significantly lower ATP values ($p<0.05$). Average ATP among 5 surfaces disinfected with ethanol ranged from 691-5167 RLU before and 454-980 RLU after. For hydrogen peroxide, average ATP among 5 surfaces ranged from 573-2970 RLU before and 533 – 1311 RLU after disinfection. There was not a significant difference in the number of sites with > 500 RLU after disinfection between disinfectants.
Wiemken 2014 (3858)	All viable organisms	Single-site, quasi-experimental, uncontrolled study over 8 days	480 samples from10 high-touch surfaces (overhead light handle, cart drawer, intravenous fluid pole knob, IV pole hanger, chair seat, EKG patch drawer, operating table, mayo stands, telephone, bedside table) were sampled in an operating room. Kentucky, USA	Terminal cleaning of surgery consisted of 1-step sodium hypochlorite (Clorox Healthcare) before the first surgery of the day. After each surgery, the room was cleaned and disinfected using an improved hydrogen peroxide product (Clorox Healthcare). This was repeated for 3 consecutive surgeries during the same day for 8 days. Samples were collected after terminal cleaning, after the first surgery, and after disinfection with improved hydrogen peroxide. Disinfectant concentration and time of measurements relative to disinfection unspecified.	Percent ATP reduction before to after disinfection with hydrogen peroxide (termed cleaning efficacy) was 96%; (95% CI, 91.6%-100.0%), 85.5%; (95% CI, 77.5%-93.4%), 84.4%; (95% CI, 76.2%-92.7%) for the first, second, and third surgery of the day respectively. No colonies were observed after the disinfection (unreported before disinfection).
Siani 2018 (4540)	All viable organisms	Single-site, Quasi-experimental, controlled crossover study, over 29 weeks	1,566 environmental samples from 11 high-touch surfaces (bed control, bed rails, tray table, call button, patient chair, drug locker, commode top, bathroom	Samples were collected during 5-week baseline period with standard disinfection. Standard disinfection consisted of cleaning with detergent followed by disinfection with 1,000 ppm chlorine (active ingredient not specified) soaked in a cotton cloth. The crossover intervention compared standard disinfection with modified	During intervention, all sites had < 2.5 CFU/cm2 in both wings of the experiment indicating training beforehand (between baseline and crossover trial) reduced bacterial load. Reduction in total aerobic count was significantly higher

Reference (Study ID)	Outcome	Study Design	Setting	Intervention	Results
			door handle, flush handle, toilet grab rail, toilet seat) in two identical surgical and cardiovascular wards at a 1,000-bed teaching hospital. Cardiff, UK.	disinfection, which consisted of peracetic acid/hydrogen peroxide wipe when activated with water. Intervention was 12 weeks of either standard or modified disinfection on one ward followed by 12 weeks with disinfection interchanged on the ward. Both wards received training. Contact time, manufacturer unspecified. Samples were collected weekly before daily disinfection.	($p<0.001$) in both crossover periods with peracetic wipe compared with detergent + chlorine. The reintroduction of detergent+chlorine following the period using peracetic acid wipe had significant increase ($p<0.001$) in total aerobic count in 3/11 surface types. Total anaerobic count and ATP were not significantly different in one ward, but significantly higher ($p<0.001$) in another ward with the use of detergent+chlorine compared to the use of peracetic acid.
Siani 2018 (4540)	All viable organisms (MDRO-VRE, CRE, EBSL)	Single-site, Quasi-experimental, controlled crossover study, over 29 weeks	1,566 environmental samples from 11 high-touch surfaces (bed control, bed rails, tray table, call button, patient chair, drug locker, commode top, bathroom door handle, flush handle, toilet grab rail, toilet seat) in two identical surgical and cardiovascular wards at a 1,000-bed teaching hospital. Cardiff, UK.	Samples were collected during 5-week baseline period with standard disinfection. Standard disinfection consisted of cleaning with detergent followed by disinfection with 1,000 ppm chlorine (active ingredient not specified) soaked in a cotton cloth. The crossover intervention compared standard disinfection with modified disinfection, which consisted of peracetic acid/hydrogen peroxide wipe when activated with water. Intervention was 12 weeks of either standard or modified disinfection on one ward followed by 12 weeks with disinfection interchanged on the ward. Both wards received training. Contact time, manufacturer unspecified. Samples were collected weekly before daily disinfection.	Percent samples positive for VRE, CRE, or ESBL during baseline period on wards 1 and 2, respectively, (7%, 35/522; 2.5%, 13/522 samples) was higher compared to hydrogen peroxide wipes (1%, 5/522; and 0.6% 3/522) and compared to detergent + chlorine (3%, 14/522; 1.3%7/522). Reductions compared to baseline could be due to training. Percent samples positive for MDROs was higher on wards using detergent + chlorine (3% and 1.3%) compared to wards using peracetic wipes (1% an d 0.6%). Significance not specified.
Biswal 2017 (12894)	Fungi (*Candida auris*)	Single site, quasi-experimental, uncontrolled case study, 4 days	Environmental sampling (unspecified number) of surfaces (bed, trolley , ventilator, refrigerator, railing, etc.)in the ICU at the Postgraduate Institute	Following mop with water, decontamination of the MICU environment was carried out with mop soaked in stabilized hydrogen peroxide 11% with silver nitrate (Ecoshield).	Prior to decontamination environmental samples (unspecified number) were positive. Following decontamination, none of the environmental samples

Reference (Study ID)	Outcome	Study Design	Setting	Intervention	Results
			of Medical Education and Research, a tertiary care, multi-specialty hospital in Chandigarh, India		(unspecified number) were positive.
Saha 2016 (3190)	Gram-negative bacteria	Single-site, quasi-experimental, controlled cohort study over a 6-week period	An unspecified number of samples were taken from 20 identical frequent touch points in two wards, including patient beds, tables, chairs and curtain rails, telephone keypad, computer keyboard and mouse; toilet washbasin rims, taps, and nozzles; shower handles, commodes, and door handles and light switches). The samples were taken from 2 matched 29-bed elderly care wards in a hospital in London, UK	Baseline measurement was taken the week prior to trial period of the study. Routine cleaning (control) on one ward with alcohol-free QAC wipes (≤0.5% cocoalkyl dimethylbenzyl ammonium chloride; Tuffie 5 Wipes) on patient equipment and 70% isopropyl alcohol wipes (Sani-Cloth 70; PDI) on nursing station equipment was compared to an intervention cleaning on a second ward with wipes producing peracetic acid when wet (sodium percarbonate ≤50% by weight, and citric acid ≤20% by weight; Clinell sporicidal wipes; GAMA Healthcare) on patient and nursing station equipment. Samples were collected within one hour of intervention. Contact time not specified.	Detection of gram-negative indicator organism in both wards were similarly low during baseline period ($p=0.31$). After intervention, the control ward (alcohol-free QAC wipes) had significantly higher surfaces positive at 17/100 (17%) compared to intervention with 4/100 (4%) surfaces positive ($p=0.003$).
Doan 2012 (414)	Gram-positive bacilli (*Clostridium difficile*)	Single-site, quasi-experimental, controlled before-after cohort study over 3 months	53 samples collected per intervention from high frequency contact surfaces from hospital environment (bedrails, door handles, light switches, nurse call bell, toilet, bed table, floor) in isolation rooms at Derby Hospital Foundation Trust. Derby, UK	*C. difficile* inoculated into rooms for 72 h. Samples taken prior to disinfection. Disinfection interventions (HPV, dry ozone, 1000 ppm chlorine, dry atomized steam, steam cleaning, peracetic acid wipes) were tested each in separate rooms to determine concentration reduction of with known concentration of *C. difficile* spores placed in rooms. Intervention peracetic acid Clinell sporicidal wipes (< 50% sodium percarbonate, < 20% citric acid, < 25% tetra acetyl ethylene diamine) had 22-minute cleaning time. Measurements taken after “designated time period specified by company guidelines.”	Log10 reductions (in CFU/mL) were highest for hydrogen peroxide, 1000 ppm chlorine-releasing agent, and peracetic acid wipes at 2.303, 2.223, and 2.134 respectively. Standardized median log10 reductions were 2.301 (IQR: 2.151, 2.301) following disinfection with peracetic acid wipes

Reference (Study ID)	Outcome	Study Design	Setting	Intervention	Results
Yui 2017 (3444)	Gram-positive bacilli (*Clostridium difficile*)	Single-site quasi-experimental uncontrolled before-after study, 1 year	2,529 samples from 16 high-touch surfaces (floor, bed rail, bed control, nurse call button, bedside table, chair arm, bin lid, door handle, ceiling vent, bathroom floor, toilet assist bar, toilet flush, toilet seat, tap handle, door handle) from 146 single-isolation rooms and 44 bed-bay areas at a large teaching hospital. London, UK	Routine and terminal cleaning consisted of peracetic acid-based disinfectant (40% acetic acid, 35.5% peracetic acid, 6.5% hydrogen peroxide, DiffX) using microfiber cloths on surfaces and microfiber mops on floors. Concentration was 1,000 ppm for surfaces and 750 ppm for floors. Hydrogen peroxide vapor (HPV) decontamination followed terminal cleaning when patient had known infection due to *C. difficile* or other HAI (Deprox system, hydrogen peroxide 29-46 ppm). Samples were collected immediately before and immediately after terminal cleaning, and immediately after hydrogen peroxide decontamination.	Number (percent) of surfaces positive for *C. difficile* before terminal cleaning was 131 of 572 surfaces (22.9%) compared to after terminal cleaning with 105 of 959 surfaces (10.6%) and after hydrogen peroxide decontamination with 43 of 967 surfaces (4.4%). In single-isolation rooms with known *C. difficile* colonized patient mean count (standard deviation) was 86.9 (98.8) CFU before terminal cleaning, 21.2 (38.7) CFU after terminal cleaning, and 7.1 (17.9) CFU after terminal cleaning + decontamination (significance not specified).
Sjoberg 2014 (4505)	Gram-positive bacilli (*Clostridium difficile*)	Single site, quasi-experimental, uncontrolled study over 2 years	A total of 640 samples were taken from 10 beds(bedrail, bedfoot) and high-touch surfaces (bed table, call button, toilet seat, etc.) in 10 hospital rooms from different wards with a CDI patient at Orebro University Hospital, Sweden	Samples were taken from rooms occupied by patients with *C. difficile*. Beds occupied by C. diff patients were cleaned with 21.4% potassium monopersulfate-based disinfectant (0.5% Virkon, Antec International) between patient use/at terminal cleaning. Samples were collected once a week for four weeks	The percent of samples positive for *C. difficile* was 23% (34/150) from 100% of rooms before disinfection compared to 3% (6/150) samples from 30% of rooms after disinfection. Specifically for beds, *C. difficile* was found in 30% (3/10) of beds before disinfection and 20% of beds after 1 week from disinfection. Significance not specified.
Deshpande 2014 (7047)	Gram-positive bacilli (*Clostridium difficile*)	Single-site, quasi-experimental, controlled cohort study over a 1-month period	A total of 888 samples were taken from the floors and high-touch surfaces (bed rails, bedside tables) of an unspecified number of CDI and MRSA isolation rooms in a hospital in the USA	Baseline measurements were not specified. Routine cleaning (control) with alcohol-free QAC (<1% n-alkyl dimethyl benzyl ammonium chloride and <1% n-alkyl dimethyl ethylbenzyl ammonium chloride; Virex; Diversey) was mopped on half of floors, and sodium hypochlorite (1:10 dilution of household bleach) was wiped onto high-touch surfaces. The product was compared to enhanced (trial)	OxyCide and bleach significantly reduced ($p<0.05$) the recovery of *C. difficile* while the QAC did not significantly reduce recovery of *C. difficile* ($p>0.05$). In bedside tables and bed rails, there was no recovery of *C. difficile* after OxyCide or bleach disinfection (from 8/50 to 0/50 surfaces

Reference (Study ID)	Outcome	Study Design	Setting	Intervention	Results
				cleaning with peracetic acid (0.13%) and hydrogen peroxide (0.63%) based sporicidal product (Oxycide; Ecolab), applied in the same fashion to the second half of floors and high-touch surfaces. All surfaces were allowed to air-dry 10-15 min. Dilutions and time until measurement were not specified.	positive for OxyCide and from 7/50 to 0/50 surfaces positive for bleach). On floors, there was 5% surfaces positive after disinfection compared to 50% before disinfection with OxyCide (from 41/82 to 4/82 surfaces positive) compared to 54% surfaces positive after QAC and 55% positive before QAC disinfection (from 45/82 to 44/82 surfaces positive).
Deshpande 2014 (7047)	Gram-positive cocci (Gram positive organisms- *Staphylococcus aureus* (MRSA) or *Enterococcus* spp (VRE))	Single-site, quasi-experimental, controlled cohort study over a 1-month period	A total of 888 samples were taken from the floors and high-touch surfaces (bed rails, bedside tables) of an unspecified number of CDI and MRSA isolation rooms in a hospital in the USA	Baseline measurements were not specified. Routine cleaning (control) with alcohol-free QAC (<1% n-alkyl dimethyl benzyl ammonium chloride and <1% n-alkyl dimethyl ethylbenzyl ammonium chloride; Virex; Diversey) was mopped on half of floors, and sodium hypochlorite (1:10 dilution of household bleach) was wiped onto high-touch surfaces. The product was compared to enhanced (trial) cleaning with peracetic acid (0.13%) and hydrogen peroxide (0.63%) based sporicidal product (Oxycide; Ecolab), applied in the same fashion to the second half of floors and high-touch surfaces. All surfaces were allowed to air-dry 10-15 min. Dilutions and time until measurement were not specified.	OxyCide and bleach significantly reduced ($p<0.05$) recovery of MRSA and/or VRE, but not QAC ($p>0.05$). In bedside tables and bed rails, there was no recovery of MRSA/VRE after OxyCide or bleach disinfection (from 11/50 to 0/50 surfaces positive for OxyCide and from 12/50 to 0/50 surfaces positive for bleach). On floors, there was no recovery after OxyCide disinfection compared to 18% positive before (from 7/40 to 0/40 surfaces positive) compared to 18% surfaces positive after QAC and 25% surfaces positive before from 10/40 to 7/40 surfaces positive).
Mitchell 2014 (13718)	Gram-positive cocci (*Staphylococcus aureus*-MRSA)	Single-site uncontrolled before-after study over 7 years	32,661 samples from 9 environmental surfaces (e.g. ceiling vent, sink, console, bed, patient/visitor chair, patient table, bedside locker, mattress, pillow) from rooms occupied by MRSA patients at 300-	MRSA patient rooms were cleaned after discharge with pH-neutral detergent from Jan 1 2006 to Oct 30 2009. From Nov 1 2009 to Dec 31 2012, terminal cleaning was switched to hydrogen peroxide. In single rooms, HP (6%) vapor decontamination using the dry hydrogen vapor room decontamination system (Nocospray). In shared rooms, HP was applied to surfaces using a cloth (Oxivir TB 0.5%). 9	MRSA was isolated from 24.7% (473/1917) rooms following detergent cleaning and from 18.8% (322/1712) of rooms after HP (349 cleaned manually and 1363 cleaned with HPV) ($p<0.001$).

Reference (Study ID)	Outcome	Study Design	Setting	Intervention	Results
			bed public acute care hospital. Tasmania, Australia	environmental samples were taken after terminal cleaning.	
Boyce 2017 (808)	HAI (HAI – All (VRE, MRSA, *C. difficile*))	Single-site, quasi-experimental controlled crossover study; 12-months	11,490 patient-days for QAC disinfectant; 10,741 patient-days for IHP disinfectant from 2 general wards in a Medical intensive care unit (MICU) in Yale New-Haven Hospital in New Haven. Connecticut, USA.	Daily and discharge cleaning using QAC disinfectant (Hyperfect 256; Genesan, Gorham, ME) with dry wipes made of melt blown polypropylene. 12-month crossover trial compared QAC with 0.5% improved hydrogen peroxide (IHP) disinfectant. Contact time was not specified. Surveillance of HAIs based on clinical data from hospital.	Composite incidence density rate for MRSA, VRE, *C. difficile* was 8.0 cases per 1000 patient-days on IHP wards compared to 10.3 cases per 1000 patient-days on QAC wards (p=0.068). Incidence rate ratio = 0.77 (95% confidence interval = 0.579-1.029).
Boyce 2017 (808)	HAI (HAI-*C. difficile*)	Single-site, quasi-experimental controlled crossover study; 12-months	11,490 patient-days for QAC disinfectant; 10,741 patient-days for IHP disinfectant from 2 general wards in a Medical intensive care unit (MICU) in Yale New-Haven Hospital in New Haven. Connecticut, USA.	Daily and discharge cleaning using QAC disinfectant (Hyperfect 256; Genesan, Gorham, ME) with dry wipes made of melt blown polypropylene. 12-month crossover trial compared QAC with 0.5% improved hydrogen peroxide (IHP) disinfectant. Contact time was not specified. Surveillance of HAIs based on clinical data from hospital.	Incidence density rates were lower (significance not specified) on IHP wards compared to QAC wards with 0.56 cases per 1000 patient-days on IHP wards compared to 1.0 cases per 1000 patient-days on QAC wards.
Alfa 2015 (14913)	HAI (HAI – *C. difficile*)	Multi-site, quasi-experimental controlled before-after study, 52 weeks	All patients admitted to medicine, cardiac, surgery, women and child wards during the study at two acute care tertiary hospitals. Manitoba, Canada.	Non-disinfectant cleaning agent (PERdiem) applied with cotton cloths was used daily at two hospitals for floors and non-patient care areas. The intervention was introduced to one hospital consisting of disinfectant wipe containing a 0.5% accelerated hydrogen peroxide disinfectant and cleaner (Accel INTERvention) on a disposable wipe with a 1-minute contact time on high-touch surfaces. Monitoring of cleaning was conducted on intervention hospital. HAI rate was compared between intervention period and 3-year period before the intervention at the intervention hospital.	There was a significant reduction (p=0.0005) in *C. difficile* cases from 6.04 cases/10,000 patient days before the intervention to approximately 3 cases/10,000 patient days after the intervention when cleaning compliance was high (>80%).

Reference (Study ID)	Outcome	Study Design	Setting	Intervention	Results
Saha 2016 (3190)	HAI (HAI – gram negative organisms)	Single-site, quasi-experimental, controlled cohort study over a 6-week period	Number of patients and HAI surveillance not specified at 2 matched 29-bed elderly care wards in a hospital in London, UK	Weekly HAI data collected. Routine cleaning (control) on one ward with alcohol-free QAC wipes (≤0.5% cocoalkyl dimethylbenzyl ammonium chloride; Tuffie 5 Wipes) on patient equipment and 70% isopropyl alcohol wipes (Sani-Cloth 70; PDI) on nursing station equipment was compared to an intervention cleaning on a second ward with wipes producing peracetic acid when wet (sodium percarbonate ≤50% by weight, and citric acid ≤20% by weight; Clinell sporicidal wipes; GAMA Healthcare) on nursing station and patient equipment. Contact time not specified.	No significant decrease in weekly HAI rate was observed in intervention ward (p=0.31) and control ward (p=0.23). HAIs were lower in the control ward than in the study ward, but these results were not statistically significant.
Boyce 2017 (808)	HAI (HAI-MRSA)	Single-site, quasi-experimental controlled crossover study; 12-months	11,490 patient-days for QAC disinfectant; 10,741 patient-days for IHP disinfectant from 2 general wards in a Medical intensive care unit (MICU) in Yale New-Haven Hospital in New Haven. Connecticut, USA.	Daily and discharge cleaning using QAC disinfectant (Hyperfect 256; Genesan, Gorham, ME) with dry wipes made of melt blown polypropylene. 12-month crossover trial compared QAC with 0.5% improved hydrogen peroxide (IHP) disinfectant. Contact time was not specified. Surveillance of HAIs based on clinical data from hospital.	Incidence density rates were lower (significance not specified) on IHP wards compared to QAC wards with 1.96 cases per 1000 patient-days on IHP wards compared to 2.79 cases per 1000 patient-days on QAC wards.
Mitchell 2014 (13718)	HAI (HAI-MRSA)	Single-site uncontrolled before-after study over 7 years	32,661 samples from 9 environmental surfaces (e.g. ceiling vent, sink, console, bed, patient/visitor chair, patient table, bedside locker, mattress, pillow) from rooms occupied by MRSA patients at 300-bed public acute care hospital. Tasmania, Australia	MRSA patient rooms were cleaned after discharge with pH-neutral detergent from Jan 1 2006 to Oct 30 2009. From Nov 1 2009 to Dec 31 2012, terminal cleaning was switched to hydrogen peroxide. In single rooms, HP (6%) vapor decontamination using the dry hydrogen vapor room decontamination system (Nocospray). In shared rooms, HP was applied to surfaces using a cloth (Oxivir TB 0.5%). MRSA screening was conducted on some patients prior to 2010 and all patients after 2010.	Incidence of MRSA colonization and infection decreased from 9.0/10,000 patient days during detergent period to 5.3/10,000 patient days during the HP disinfectant period (p<0.001).
Alfa 2015 (14913)	HAI (HAI-MRSA)	Multi-site, quasi-experimental	All patients admitted to medicine, cardiac, surgery, women and	Non-disinfectant cleaning agent (PERdiem) applied with cotton cloths was used daily at two hospitals for floors and non-patient care areas.	There was a significant reduction (p=0.0071) in MRSA cases from 11.43 cases/10,000 patient days

Reference (Study ID)	Outcome	Study Design	Setting	Intervention	Results
		controlled before-after study, 52 weeks	child wards during the study at two acute care tertiary hospitals. Manitoba, Canada.	The intervention was introduced to one hospital consisting of disinfectant wipe containing a 0.5% accelerated hydrogen peroxide disinfectant and cleaner (Accel INTERvention) on a disposable wipe. with a 1-minute contact time on high-touch surfaces. Monitoring of cleaning was conducted on intervention hospital. HAI rate was compared between intervention period and 3-year period before the intervention at the intervention hospital.	before the intervention to approximately 2.5 cases/10,000 patient days after the intervention when cleaning compliance was high (>80%).
Boyce 2017 (808)	HAI (HAI-VRE)	Single-site, quasi-experimental controlled crossover study; 12-months	11,490 patient-days for QAC disinfectant; 10,741 patient-days for IHP disinfectant from 2 general wards in a Medical intensive care unit (MICU) in Yale New-Haven Hospital in New Haven. Connecticut, USA.	Daily and discharge cleaning using QAC disinfectant (Hyperfect 256; Genesan, Gorham, ME) with dry wipes made of melt blown polypropylene. 12-month crossover trial compared QAC with 0.5% improved hydrogen peroxide (IHP) disinfectant. Contact time was not specified. Surveillance of HAIs based on clinical data from hospital.	Incidence density rates were lower (significance not specified) on IHP wards compared to QAC wards with 5.49 cases per 1000 patient-days on IHP wards compared to 6.6 cases per 1000 patient-days on QAC wards.
Alfa 2015 (14913)	HAI (HAI-VRE)	Multi-site, quasi-experimental controlled before-after study, 52 weeks	All patients admitted to medicine, cardiac, surgery, women and child wards during the study at two acute care tertiary hospitals. Manitoba, Canada.	Non-disinfectant cleaning agent (PERdiem) applied with cotton cloths was used daily at two hospitals for floors and non-patient care areas. The intervention was introduced to one hospital consisting of disinfectant wipe containing a 0.5% accelerated hydrogen peroxide disinfectant and cleaner (Accel INTERvention) on a disposable wipe with a 1-minute contact time on high-touch surfaces. Monitoring of cleaning was conducted on intervention hospital. HAI rate was compared between intervention period and 3-year period before the intervention at the intervention hospital.	There was a significant reduction ($p<0.0001$) before compared to after the intervention for VRE cases from 25 cases/10,000 patient days to approximately 14 cases/10,000 patient days when cleaning compliance was high (>80%). For any cleaning compliance, there was also significant reduction ($p=0.0358$) from 25 cases/10,000 patient days before the intervention to approximately 16 cases/10,000 patient days after the intervention.
Doidge 2010 (12244)	HAI (HAI-Carbanapem resistant	Single site, quasi-experimental, uncontrolled	HAI colonization/infection of patients with CR-AB were reported over 4	A new disinfection product was introduced to replace routine cleaning, which did not completely remove CR-AB from environmental surfaces. Routine cleaning was 1% neutral	Before the introduction of the new disinfectant and during routine cleaning, 41 patients were newly colonized with CR-AB in prior 6

Reference (Study ID)	Outcome	Study Design	Setting	Intervention	Results
	Acinetobacter baumanii)	study over 4 years	years in a 19-bed long-stay ICU in a large teaching hospital in Brisbane, Australia	detergent water and 70% alcohol wipes (> 1-minute contact time) 3 times a day for 3 days. The new disinfection product was oxidizing disinfectant (Virkon S; potassium peroxomonosulphate 50%, sodium alkyl benzene sulphonate 15% and sulphamic acid 5%) applied for at least 10 minutes.	months. The introduction of the new disinfection product controlled the outbreak and only 6 patients were found to have CR-AB in the next two years of follow-up. A temporal association between the introduction of Virkon S and outbreak end was seen (significance not specified).

Table S13: Study results for manually applied quaternary ammonium compound interventions ordered by outcome organism

Reference (Study ID)	Outcome	Study Design	Setting	Intervention	Results
Carling 2014 (111)	All viable organisms	Single-site, quasi-experimental, controlled cohort study over several months	A total of 571 samples were collected from 12 high-touch surfaces (door, bedrail, call button, telephone, tray table, room chair, bathroom inner door, bathroom light switch, bathroom sink, bathroom grab bar, toilet handle, and toilet seat) in 48 rooms of an ICU of a general acute care hospital in the USA	Baseline samples were taken prior to disinfection (before) with one of two disinfectants: (1) low-alcohol QAC (final dilution: 0.10% octyl decyl dimethyl ammonium chloride, 0.04% dioctyl dimethyl ammonium chloride, 0.06% didecyl dimethyl ammonium chloride, and 0.14% alkyl dimethyl benzyl ammonium chloride; Quaternary Disinfectant Cleaner; Ecolab); (2) a product containing peracetic acid and hydrogen peroxide (Oxycide, 0.63% hydrogen peroxide, 0.13% peroxyactic acid). Disinfectant applied with pre-saturated cloth (after). Wet contact times, time until measurement not specified.	Median bioburden 17 CFU/slide for QAC surfaces and 15 CFU/slide for Oxycide surfaces (p=0.06) (before). The disinfection intervention (after) had complete removal of bacterial bioburden in 40% (93/237) of samples with QAC and 77% (211/274) with Oxycide. Hydrogen peroxide/peracetic acid disinfectant was 1.93 times more effective at removing bacteria compared to QAC (p<0.001).
Casini 2018 (128)	All viable organisms	Single-site, quasi-experimental, controlled cohort study over a 2-month period	560 samples were taken from 5 high-touch inanimate surfaces per room (bedrails, overbed tables, worktop, infusion pump, monitor). Samples were taken from four patient units in a 12-bed ICU at a university hospital. Pisa, Italy.	A new single-wipe disinfection protocol with alcohol-free QAC-impregnated disposable wipes with proprietary ingredients (≤0.5% benzalkonium chloride, ≤0.5% didecyl dimethyl ammonium chloride, ≤0.10% polyhexamethylene biguanide (PHMB); Clinell Universal Sanitising Wipes; GAMA Healthcare Limited) was compared to a two-step protocol (control) of cloth application of alcohol-based detergent (Keradet-Aktiv; Kiehl, unspecified dilution) followed by a chlorine-based disinfectant (sodium hypochlorite, Antisapril 2%; Angelini, active chlorine 540 mg/L). Baseline samples were taken immediately prior to disinfection with the new or two-step protocol. Measurements were taken at	Average concentration ± standard deviation (CFU/25 cm^2) decreased significantly (71.2%, p=0.005) from 52 ± 63 prior to disinfection with alcohol-free QAC to 15 ± 24 after 0.5 hours. The two-step protocol with hypochlorite did not have a significant reduction (38.2%, p=0.32) compared to baseline (pre-protocol) measurements. Average concentration at 2.5, 4.5, and 6.5 hours after QAC disinfection were 20, 17, and 13 CFUs/25 cm^2, respectively (significance not specified).

Reference (Study ID)	Outcome	Study Design	Setting	Intervention	Results
				0.5, 2.5, 4.5, and 6 hours after disinfection. Wet contact time not specified.	
Dharan 1999 (393)	All viable organisms	Single-site, Quasi-experimental, controlled cohort study for a 4-month period.	A total of 1356 surface (floors, furniture) samples were collected weekly in patient areas from two wings of a 106-bed medical unit at a tertiary-care hospital. Geneva, Switzerland.	Routine cleaning and disinfection was compared for floors, furniture, and bathroom/toilet floors on two wards using a QAC (0.5% ISEQUAT®, unknown active ingredients), detergent (1% TASKI® R50), and/or active oxygen based (AOB) compound (1% PERFORM, pentapotassium-bis-(peroxymonosulphate) bis(sulphate). Weekly samples were taken after surfaces were dry (10-15 minutes later).	For ward floors, using QAC had minimal decrease in bacterial count with average decrease 0.6 CFU/24 cm^2; 95% CI: 26-27) compared to detergent which introduced bacteria averaging additional 103.6 CFU/24 cm^2, 95% CI: 73–134. Active oxygen-based compound had a larger reduction averaging 111.1 CFU/24 cm2 (CI 95 87-133). Similar results were seen for bathroom/toilet floors. The AOB was significantly more effective than the QAC on floors ($p<0.001$).
Attaway 2012 (621)	All viable organisms	Single-site, quasi-experimental, controlled cohort study over a period of 6 months	A total of 18 microbiological samples were collected from bedrails in 3 rounds of sampling of 6 patient rooms each; sampling was done in the MICU of a teaching hospital, SC, USA	Baseline samples were taken immediately prior to disinfection. Routine cleaning was done with two products: (1) 1:256 dilution of low-alcohol QAC (final concentration of 660 ppm 0.07% n-alkyl dimethyl benzyl ammonium chloride and 0.07% didecyl dimethyl ammonium chloride; Virex II 256; Diversey) ;(2) high-alcohol (17.2% isopropanol) QAC in a ready-to-use, pre-diluted spray bottle (final concentration: 0.28% diisobutyl phenoxyethoxyethyl dimethyl benzyl ammonium chloride; Cavicide; Metrex). Products had a wet application time of 30 minutes. Measurements were taken at 0.5, 2.5, 4.5, and 6.5 hours after cleaning.	Mean (median) concentration before cleaning with low-alcohol QAC Virex II 256 was 3,711 CFU/100 cm2 compared to 2,057 CFU/100 cm2 at 30 minutes after cleaning, for a mean (median) relative reduction of 45% (95%). Within 2.5 hours after cleaning, mean concentration exceeded 250 CFU/100 cm2 (significance not specified). Mean concentration before cleaning with high-alcohol QAC CaviCide was 5,800 CFU/100 cm2 compared to 58 CFU/100 cm2 at 30 minutes after cleaning, for a mean (median) relative reduction of 99% (98%). Within 2.5 hours after cleaning, mean concentration exceeded 250 CFU/100 cm2 (significance not specified). The mean relative reduction of the bacterial population on bed rails after 30 minutes was significantly higher ($p=0.017$) for CaviCide (99%) than for Virex II 256 (45%).

Reference (Study ID)	Outcome	Study Design	Setting	Intervention	Results
Blazejewski 2015 (766)	All viable organisms	Single-site, quasi-experimental, uncontrolled study over a 3-month period	A total of 546 samples were taken from 8 surfaces in 182 rooms including inside lateral part of mattress, ventilator, monitor, underside of overbed table, room door handle, sink, bedrail, and keyboard for 13 computerized rooms or storage box for other rooms. Samples were taken at the ICU of a university hospital in France.	Baseline measurements were taken after patient discharge. A low-alcohol QAC was used for terminal cleaning (control) to clean the floors once daily (2.5-10% didecyl dimethyl ammonium chloride and 2.5-10% propan-2-ol; Aniosurf; Anios) at an unspecified final dilution, as well as surfaces using two applications of the solution, each with a wet contact time of five minutes. The sink was first cleaned by a detergent, rinsed with clear water, then cleaned and disinfected with bleach. Enhanced cleaning (trial) was performed with either 30% hydrogen peroxide vapor (HPV) or aerosolized hydrogen peroxide (7% H_2O_2 solution, 0.25% peracetic acid, 30% acetic acid). Time until measurement "after terminal cleaning".	The number of rooms contaminated with at least one bacterium prior to disinfection with Ansiosurf were 141/182 (77%), compared to 70/182 (38%) following disinfection, a significant reduction ($p<0.001$).
Boyce 2017 (808)	All viable organisms	Single-site, quasi-experimental controlled crossover study; 12-months	1061 samples prior to cleaning & 1092 samples after cleaning from 5-8 high-touch surfaces (bedside rails, remote control module, overbed tables, toilet seats, toilet grab bars, counters, supply cart keyboards, and workstations on wheels) from 2 general wards in a MICU at Yale New-Haven Hospital in New Haven. Connecticut, USA.	Daily and discharge cleaning using QAC disinfectant (Hyperfect 256; Genesan, Gorham, ME) with dry wipes made of melt blown polypropylene. 12-month crossover trial compared QAC with 0.5% improved hydrogen peroxide (IHP) disinfectant. Contact time was not specified. Monthly measurements were taken at unspecified time relative to disinfection.	Mean aerobic colony count per high-touch surface after cleaning was significantly higher ($p=0.003$) with QAC at 22.2 CFU compared to 14.0 CFU with IHP. Percent surfaces with no growth after cleaning with QAC was significantly lower at 35% (182/517) compared to 48% (240/500) with IHP ($p < 0.001$). Percent surfaces with < 2.5 CFU/cm2 was significantly lower with QAC at 88.4% (457/517) compared to IHP at 92.4% (462/500) ($p=0.03$).

Reference (Study ID)	Outcome	Study Design	Setting	Intervention	Results
Boyce 2014 (810)	All viable organisms	Single site, quasi-experimental controlled cohort study over 4-week period	1587 samples from high-touch surfaces (bedside rail, overbed table, TV remote, telephone, door handle, dresser, toilet seat, bathroom grab bar, sink handles) in 9 patient rooms on rehabilitation ward at 500-bed community teaching hospital. Connecticut, USA.	Daily cleaning consisted of QAC (Virex 256). In three intervention rooms, 9 high-touch sites were allowed to dry after daily cleaning and then coated with one of two organosilate test products with microfiber cloth: (i) Eco Antimicrobial, (ii) Bio-Protect AM500. Test products were not applied to surfaces in three control rooms. Samples were collected from each site daily before daily cleaning.	Neither test product had lower mean colony counts than control rooms for most sites. Mean colony count ranged from approximately 15-115 for control sites, ~25-130 for test product 1, and ~15-115 for test product 2 depending on site type. Control sites had significantly lower mean colony count compared to the test products.
Fattorini 2018 (975)	All viable organisms	Single-site, quasi-experimental, uncontrolled study over a 7-month period	A total of 237 samples, were taken from 13 matched surfaces (porthole, wall, mattress, humidity chamber, control panel, cover) of 20 incubators from the neonatal pediatric unit of a teaching hospital in Italy.	Baseline samples were taken prior to disinfection. The trial product was an alcohol-free QAC (n-benzy-n-dodecyl-n,n-dimethyl-ammonium chloride and n-benzyl-n,n-dimethyl-n-tetradecyl-ammonium chloride; UMONIUM38 Neutralis; Huckerts) at a manufacturer recommended concentration and contact time of 2.5% and 10 min, respectively. Sampling was performed within 30 minutes of disinfection. Application matrix not specified. No comparison group.	Mean reduction in concentration (95% CI) following the use of alcohol-free QAC was 205 CFU (–99-330) for all viable organisms. Maximum reduction in concentration was 5730 CFU. Average (95% CI) reduction in concentration was 91.6% (86.6 – 95.7). A second non-selective media was used with similar results. Concentration reduction was significantly higher on surfaces inside incubator (97%) compared to surfaces outside incubator (88%) ($p<0.05$).
Fitton 2017 (1011)	All viable organisms	Single-site, quasi-experimental, controlled cohort study over a 5-month period	A total of 1382 microbiological samples were collected from bedrails, patient call pad, patient tray table, and bedside table drawer handle in 342 patient rooms in the MICU of a community teaching hospital in the USA	Baseline measurements were taken for 7 consecutive days prior to intervention period. A saline solution (control) or alcohol-free QAC (treatment) (0.75% 3-trihydroxysilylpropyldimethyl-octadecylammonium chloride; Goldshield 75; AP Goldshield) were applied to rooms selected for control or treatment as ready-to-use spray. Wet contact time not specified. Product was applied every 30 days or after terminal cleaning with bleach, with sampling	Mean 5-month reduction in total bioburden was 65.9% from baseline following the use of alcohol-free QAC compared to 30.8% reduction for placebo surfaces. Reduction was both significant compared to baseline ($p<0.001$) and to placebo ($p=0.02$). Baseline concentration not reported.

Reference (Study ID)	Outcome	Study Design	Setting	Intervention	Results
				performed weekly. Sample measurement time relative to disinfection not specified.	
Frota 2017 (1045)	All viable organisms	Single-site, quasi-experimental, uncontrolled study over a 4-month period	A total of 720 samples were collected from surfaces (medication preparation areas, heat monitor, dressing trolley, and mattress). Samples were taken from the emergency room, medication room, bandaging room, and observation room of a walk-in emergency clinic in Brazil.	Samples were taken before and 10 minutes after cleaning and disinfection which consisted of routine cleaning with detergent followed by disinfection with alcohol-free QAC with proprietary ingredients (12.4% glucoprotamin and 15% alkyl-dimethyl-benzyl-ammonium chloride; Ecolab Deutschland), at an unspecified final dilution. Products were allowed to air dry for 10 minutes prior to sampling.	The median concentration of aerobic colony counts (ACC; CFU/cm^2) for all surfaces was 2.7 (range: 0.1-81.9) prior to disinfection with alcohol-free QAC, with a total of 67 samples (61.4%) <2.5 CFU/cm^2. Following disinfection, median ACC for all surfaces was significantly lower ($p<0.001$) at 0.2 (range 0-68.5) with 14 samples (11.7%) <2.5 CFU/cm^2.
Furlan 2019 (1065)	All viable organisms	Single-site, quasi-experimental, uncontrolled study over a period of 6 months	A total of 240 samples were taken from 5 surfaces (dressing trolley, stretcher, reception desk, support table, operating table). Samples were taken from an unspecified number of rooms in multiple wards of an outpatient clinic in Mato Grosso du Sul, Brazil.	Baseline measurements were taken immediately prior to standard cleaning. Standard cleaning was performed with a combination detergent and disinfectant, alcohol-free QAC with proprietary ingredients (12.4% glucoprotamin and 15% alkyl-dimethyl-benzyl-ammonium chloride; Ecolab Deutschland) at an unspecified final dilution. Product was allowed to air dry. Measurements were taken within 10 minutes of standard cleaning. Application matrix not specified. No comparison group.	Median concentration (range) of aerobic colony counts (ACC) for all surfaces was 47 CFU/cm^2 (0-300) before standard cleaning and 9.5 CFU/cm^2 (0-178) after. Median (range) ATP bioluminescence for all surfaces was 250 RLU/cm^2 (23-9,597) before standard cleaning and 59 RLU/cm^2 (11-2,083) after.
Hinsa-Leasure 2016 (1245)	All viable organisms	Single-site, quasi-experimental, controlled cohort study over a 12-month period	A total of 665 samples were taken from 20 high-touch surfaces (outside patient rooms: sinks and faucet handles,	Baseline samples were taken for 10 weeks prior to installation of copper surfaces. Afterward, samples were taken weekly (time until measurement relative to cleaning unspecified). Routine cleaning was comprised of a	Mean (median) concentration during 12-month intervention period for control components was significantly higher at 6,172 at CFU/100 cm^2 (364) compared to rooms with copper components at 117 CFU/100 cm^2 (0). After routine

Reference (Study ID)	Outcome	Study Design	Setting	Intervention	Results
			keyboards, door opener push plates, toilet flush lever, grab bars, door handles, light switches, bed tables, bed rails) in 18 occupied and unoccupied patient rooms from the medical-surgical suite of a 49-bed rural hospital in Iowa, USA	low-alcohol QAC (10-30% dicapryl/dicaprylyl dimonium chloride, 5-10% alkyl dimethylbenzyl ammonium chloride, 1-5% alcohol, and 1-5% tetrasodium EDTA; High Dilution Disinfectant 256; Spartan Green Solutions) at an unspecified final dilution. Routine cleaning in control rooms was compared to rooms with a copper nickel alloy (C706; trial arm) that contained 90% copper by weight for surfaces, plus daily and terminal cleaning with product (OxivirTB) containing benzyl alcohol (1-5%), hydrogen peroxide (<1%), and dodecylbenzene sulfonic acid (<1%). Rooms previously housed by patients with *C. difficile*, in both arms of the study, were subject to cleaning with 0.65% bleach product (Clorox Bleach Germicidal Cleaner; Clorox). Application matrix, wet contact time not specified.	cleaning, 59% of samples fell below recommended 250 CFU/100 cm^2 threshold in control rooms near patients compared to 91% in rooms with copper.
Lewis 2015 (1585)	All viable organisms	Single site, quasi-experimental, controlled cohort study over a 6-week period	A total of 720 samples were collected from surfaces (telephone handpieces, computer keyboards, physician workstations, door handles, outer surface blood pressure cuffs, bed tables) in 4 operating rooms (one hybrid, one transplant, and two general surgical). Wisconsin, USA.	Control surfaces had terminal cleaning with quaternary disinfectant (unspecified product, active ingredient, and contact time) twice a week. Treated surfaces had antimicrobial isopropyl alcohol/organofunctional silane (IOS) solution applied and allowed to dry at beginning of intervention. Baseline samples were taken after QAC disinfection. During intervention period, samples were taken 3 times a week for 6 weeks after terminal cleaning.	Baseline samples had between 29.9-57.8%, surfaces in surfaces designated as dirty (>46 RLUs) by ATP bioluminescence assay in operating rooms. During the intervention phase, the average adenosine triphosphate bioluminescence (ATB) for untreated sites was significantly higher (p=0.048) (242.0 RLU, range 19.4-2872.6 RLU) compared to IOS treatment (67.6 RLU, range 0-297.5 RLU) for treated sites. Percent of surfaces negative was 20% for untreated surfaces compared to 82.5% for treated surfaces. The mean concentration among culture-positive

Reference (Study ID)	Outcome	Study Design	Setting	Intervention	Results
					surfaces for untreated sites was significantly (p<0.001) higher at 14.3 CFU compared to 1.7 CFU for treated sites.
Schmidt 2019 (2655)	All viable organisms	Single-site, quasi-experimental, controlled cohort study over an unspecified period of time	A total of 129 samples were taken from bedrails in 132 beds of the ICU of a teaching hospital in SC, USA	Baseline measurements were taken prior to disinfection. A trial product and persistent disinfectant containing 70% ethanol and <1% mixed QAC along with proprietary agents designed to increase longevity on surfaces (active QAC not specified; Firebird F130; Microban), was tested against two control products. The first was a low-alcohol QAC solution (8.2% n-alkyl dimethyl benzyl ammonium chloride, 8.7% didecyl dimethyl ammonium chloride, and 2.9% ethyl alcohol; Virex II 256; Diversey) at an unspecified final dilution. The second was a high-alcohol QAC solution (17.2% isopropanol and 0.28% diisobutyl phenoxyethoxyethyl dimethyl benzyl ammonium chloride; CaviCide; Metrex) at an unspecified final dilution. All products were applied consistent with manufacturer instructions and allowed to air dry. Measurements were taken at 1, 6, and 24 hours post disinfection.	Trial product (Firebird F130) had significantly lower (p<0.05) median concentration after 1 h, 6 h, and 24 h after disinfection compared to before when compared to each of the control disinfectants. Each of the three disinfectants had significantly lower concentration after 1 h compared to before. Virex II 256 had significantly lower concentration (p<0.05) only after 1 h (135 CFU/100 cm2) and not after 6 h (540 CFU/100 cm2) and 24 h (735 CFU/100 cm2) after disinfection compared to before (480 CFU/100 cm2). CaviCide had significantly lower concentration (p<0.05) after 1 h (30 CFU/100 cm2) and 6 h (450 CFU/100 cm2), but not 24 h (630 CFU/100 cm2) after disinfection compared to before (990 CFU/100 cm2). The median bacterial burden following use of control was significantly higher at all three time points (for Virex II 256) and at 1 h and 6 h (for CaviCide) when compared to Firebird F130 (p<0.05).
Suzuki 1984 (2944)	All viable organisms	Single-site, quasi-experimental study, uncontrolled before-after study for a 5-year period.	28 samples were taken from floor surfaces and 184 samples were taken from surfaces of equipment from 10 operating rooms in Nagoya University	After every operation, floors were cleaned with a wet mop soaked in 0.1% benzethonium chloride and water daily. Samples were taken during period of cleaning with soap and water (before) and during period using disinfection (after) at unspecified time relative to disinfection.	Mean bacterial count (standard deviation) on operating room floors was 2.6 CFU (2.2) without vs 0.2 CFU (0.5) with disinfection (p=0.005). Mean bacterial count (standard deviation) on equipment surfaces was 7.5 CFU (14.3) without vs 1.7 CFU (2.5) with disinfection (p=0.001). The most common bacterial species recovered was

Reference (Study ID)	Outcome	Study Design	Setting	Intervention	Results
			Hospital. Nagoya, Japan.		coagulase-negative staphylococci followed by spore-bearing bacilli.
Santos-Junior 2018 (3236)	All viable organisms	Single-site, quasi-experimental, uncontrolled before-after study for a 4-week period.	80 samples from 5 high-touch surfaces (bed rails, bedside table, inner bathroom door handle, toilet bowl rim, toilet flush handle) from 8 rooms in an internal medicine and surgical nursing ward at 45-bed hospital. Mato Grosso do Sul, Brazil.	Concurrent cleaning was performed once a day in the morning. Disinfectant used was a QAC based in combination with polymeric biguanide (35-40% dimethyl benzyl ammonium chloride; bigunide polyhexamethylene hydrochloride 4-5%, Nippo-Bac Plus, Nippon Chemical Inc.). Surfaces were rubbed with 100% cotton cloths that had been soaked in previously diluted disinfectant solution. Samples were collected twice a week before and after ten minutes of disinfection allowing samples to dry.	The range of the median ATP readings on all surface types was 358-946 RLU before compared to 47-176 RLU after. Two surface types had significant reduction in ATP ($p<0.05$) including bathroom door handles and toilet bowls. Sides of bed, bedside tables, and toilet flush handles all had reductions but not significant reductions.
Vesley 1987 (3614)	All viable organisms	Single-site, quasi-experimental cohort study over a period of 4 months	120 samples were taken from floors covered in resilient vinyl tiles, from 6 rooms in a large metropolitan hospital in the MN, USA	Baseline samples were collected prior to treatment with dry mop. Following dry mop, floors were wet mopped with a low-alcohol QAC (undiluted concentration: 5.8% didecyl dimethyl ammonium chloride, 4.7% benzyl alkyldimethyl chlorides, 1-5% ethanol, and 1-5% alcohols; SaniMaster III; Ecolab) QAC was allowed to air dry. Time until measurement after cleaning not specified.	Percent reduction of mean concentration was 83.1% with higher reduction in winter (85.8%) compared to summer (80.3%) following implementation of low-alcohol QAC (significance not specified).
Le Coutour 1991 (5485)	All viable organisms	Single-sited, quasi-experimental, controlled before-after study	15 samples were taken from surfaces in hospital clinical hematology and neonatology departments of hospital. Caen, France.	Comparison of three different products: Phenol and quaternary ammonium, nonionic surfactant; Phenol, benzalkonium chloride, nonionic surfactant; Aldehydes, nonionic surfactant. Contact time was 5 min for each disinfectant. Samples were taken before disinfection and 15 minutes after disinfection after a period of 10 days using the product.	There was no difference in the efficacy between the three products. The log reduction after disinfection ranged from 0.53-0.79 log CFU corresponding to a reduction by a factor of 3.3 to 6.1. No difference was observed between the disinfectants (significance not specified).

Reference (Study ID)	Outcome	Study Design	Setting	Intervention	Results
Kitagawa 2020 (5623)	All viable organisms	Single-site, quasi-experimental, uncontrolled before-after study over a 5-month period	204 samples from high -touch surfaces (bed rail, bed control panel, overbed table, vital sign monitor control panel, infusion pump control panel, bedside table, door handle, sink counter) from 11 rooms in the ICU, emergency ICU, and high care unit of a 740-bed tertiary care hospital. Hiroshima, Japan.	Baseline samples were collected after patient discharge, prior to daily standard manual cleaning. Manual cleaning of surfaces was performed with wipes containing 0.5% benzalkonium chloride (Seifukipu; Kao Corporation, Tokyo, Japan). Samples were collected after cleaning once surfaces were dry.	Percent surfaces positive was significantly lower ($p<0.001$) at 58.8% after cleaning compared to before at 82.4%. Mean concentration was lower after manual cleaning compared to before from 29.8 ± 58.6 CFU to 14.4 ± 38.7 CFU. Median concentration was significantly lower after cleaning compared to before.
Strat 1971 (5698)	All viable organisms	Single-site, Quasi-experimental, controlled before-after study for a 3-day period.	581 surface samples (operating tables, instruments, walls, floors) from 7 rooms in the Bucharest Institute of Hygiene. Bucharest, Romania.	A hydrochloride solution (1% Ampholytic detergent with dodecyldiaminoethylglycine; Tego 103G), 1% cationic detergent (cetylpyridinium chloride) (BCP), and alcohol-based disinfectant (triethylene glycol; TEG) were used to simultaneously aerosolize/disinfect air and manually wipe/disinfect surfaces. Samples taken at least 10-15 minutes after cleaning as well as 1.5, 6, and 12 h after cleaning. This values were compared to standard cleaning (water with soda).	On average, the efficiency of each disinfectant for decreasing the bacterial load is 90% for BCP (80-98.5%), 95% for Tego 103G (88-99%), 96% for TEG aerosolization and BCP wipes (80-98.5%) and 99% for TEG aerosolization and Tego 103G wipes (98.8-100%) compared to standard cleaning. The efficiency of these disinfectants changed throughout the day as well. After 1.5 h, BCP is 13.8 times more effective, Tego 103G is 22 times more effective at reducing bacterial count. After 6 h, efficiency lowers with BCP is 3.6 times more effective and Tego 103G is 5.6 times more effective at reducing bacterial load. After 12.5 h, BCP was 5.3 times more effective and Tego 103G is 9.8 times more effective at reducing bacterial load. Bacterial load included *Staphylococcus*, P. *aeruginosa*, *Proteus* mirabilis. *E. coli* were not isolated.

Reference (Study ID)	Outcome	Study Design	Setting	Intervention	Results
Codish 2015 (9825)	All viable organisms	Single-site, quasi-experimental, controlled cohort study over 2 weeks	A total of 86 samples were taken from the keyboards and mice of 8 wards (internal medicine and ICU), in a large (>1000 bed) teaching hospital in Israel.	Baseline measurements were taken prior to decontamination. Simultaneous trials were run using two decontamination products. Product 1 was an alcohol-free QAC wipe with proprietary ingredients (<1% polymeric biguanide hydrochloride, <1% alkyl dimethyl benzyl ammonium chloride, and dodecyl dimethyl ammonium chloride; TriGene Advance; MediChem International). Product 2 was a wipe containing ethanol, 70% isopropyl alcohol, 0.5% chlorhexidine, and 0.45% hydrogen peroxide 0.45% (MEDIWIPES; Albaad). Wet contact time, time to measurement not specified.	No. of rooms (%) with improved pathogenicity (i.e. more surfaces negative for high-risk pathogens after disinfection) was significantly (p<0.001) higher for alcohol-based at 32 rooms (42.1%) compared to QAC-based cleaning at 16 room (18.6%). Other pathogenicity categories were not different between products. For QAC product, 15 surfaces (17.4%) had initial low-risk pathogenicity, which was unchanged by decontamination; 20 (23.35%) had worsened pathogenicity following decontamination; and 35 (40.69%) had high-risk pathogenicity, unchanged by decontamination. Alcohol-based compared with quaternary ammonium-based decontamination had higher odds of surfaces negative for high-risk pathogen groups (odds ratio of 1.77 (95% CI, 1.36- 2.89; p<0.001).
Bogusz 2013 (14746)	All viable organisms	Single-site, quasi-experimental study over a period of 3 months	A total of 360 samples were taken from 4 surface types, including bedside locker, left bedrail, overbed table, and right bedrail. Samples were taken from 30-bed elder care ward in an NHS hospital in the UK.	Tuffie detergent wipes, Vernacare, Bolton, UK were studied for their efficacy in reducing ACC, MSSA, and MRSA. Samples were taken 1, 2, 4, 8, 12, 24, and 48 hours after intervention.	Mean ACC/cm2 per site was 6.72 prior to disinfection, 5.55 at 1 hour after disinfection, then 4.54, 3.46, 4.25, 3.67, 4.89 and 5.27 at 2, 4, 8, 12, 24, and 48 hours respectively. Results were significant (p<0.0001) 4 hours after disinfection compared to before disinfection.
Rutala 2018 (10553)	All viable organisms (MDRO- MDR *Acinetobacter*, MRSA, VRE, *C. difficile*)	Multi-site, controlled cohort study over 27-month period	7,360 samples from environmental surfaces (bed rail, over-bed table, supply or medicine	Compared standard cleaning with quaternary ammonium compound disinfection in 21 randomly selected rooms with standard cleaning with bleach in 20 randomly selected rooms.	Average concentration of pathogen was 60.8 CFU per room using quaternary ammonium compounds compared to 11.7 CFU per room when using bleach (81% reduction).

Reference (Study ID)	Outcome	Study Design	Setting	Intervention	Results
			cart, chair sink, toilet seat, shower floor, side counter, linen hamper lid, bathroom floor) in 92 rooms of 3 university-affiliated hospitals in North Carolina, USA	Active ingredients and contact time were not specified. Samples were collected after disinfection (not specified).	
Blazejewski 2015 (766)	All viable organisms (MDRO-ESBL gram negative, MRSA, IRAB, resistant P. *aeruginosa*)	Single-site, quasi-experimental, uncontrolled study over a 3-month period	A total of 546 samples were taken from 8 surfaces in 182 rooms including inside lateral part of mattress, ventilator, monitor, underside of overbed table, room door handle, sink, bedrail, and keyboard for 13 computerized rooms or storage box for other rooms. Samples were taken at the ICU of a university hospital in France.	Baseline measurements were taken after patient discharge. A low-alcohol QAC was used for terminal cleaning (control) to clean the floors once daily (2.5-10% didecyl dimethyl ammonium chloride and 2.5-10% propan-2-ol; Aniosurf; Anios) at an unspecified final dilution, as well as surfaces using two applications of the solution, each with a wet contact time of five minutes. The sink was first cleaned by a detergent, rinsed with clear water, then cleaned and disinfected with bleach. Enhanced cleaning (trial) was performed with either 30% hydrogen peroxide vapor (HPV) or aerosolized hydrogen peroxide (7% H2O2 solution, 0.25% peracetic acid, 30% acetic acid). Time until measurement “after terminal cleaning”.	The number of rooms contaminated with at least one MDRO prior to disinfection with Aniosurf were 15/182 (8%), compared to 11/182 (6%) following disinfection, an insignificant reduction (p=0.371).
Passaretti 2013 (2322)	All viable organisms (MDRO- MRSA, VRE, MDR GNR, *C. difficile*)	Single-site, quasi-experimental controlled study over 9 months	1039 room surfaces (bedrail, keyboard, monitoring equipment in patient room) in 6 high-risk units (ICU, surgical unit) at 994-bed tertiary referral hospital. Baltimore, USA	3-month pre-intervention phase followed by 6-month intervention phase with HPV (Bioquell, no specific concentration, 1.5 – 3 h) after standard cleaning on 3 units compared to standard cleaning alone with quaternary ammonium compound (active ingredient not specified, 3M) on 3 units. Samples were taken monthly.	Significant reduction for patient rooms positive for > 1 MDRO in HPV units during intervention compared to non-HPV units (relative risk 0.65, p=0.03)

Reference (Study ID)	Outcome	Study Design	Setting	Intervention	Results
Fattorini 2018 (975)	Fungi	Single-site, quasi-experimental, uncontrolled study over a 7-month period	A total of 76 samples, were taken from 13 matched surfaces (porthole, wall, mattress, humidity chamber, control panel, cover) of 20 incubators from the neonatal pediatric unit of a teaching hospital in Italy.	Baseline samples were taken prior to disinfection. The trial product was an alcohol-free QAC (n-benzy-n-dodecyl-n,n-dimethyl-ammonium chloride and n-benzyl-n,n-dimethyl-n-tetradecyl-ammonium chloride; UMONIUM38 Neutralis; Huckerts) at a manufacturer recommended concentration and contact time of 2.5% and 10 min, respectively. Sampling was performed within 30 minutes of disinfection. Application matrix not specified. No comparison group.	Mean reduction in concentration (95% CI) following the use of alcohol-free QAC was 170 CFU (80-274). Maximum reduction in concentration was 2,880 CFU, with an average (95% CI) reduction of 96.8% (93.8–99.2).
Panknin 2014 (4960)	Fungi (Candida albicans)	Single site, quasi-experimental uncontrolled before-after study over 2 months	147 samples collected from flat surfaces (incubator, heater, ventilator) in patient rooms in the neonatal intensive care unit at a university children’s hospital. Sacramento, CA, USA.	Samples were collected before and after intensive terminal disinfection with 3% final concentration of high-alcohol QAC (Super Sani-Cloth, PDI, 55.5% isopropyl alcohol, 0.25% n-alkyl-dimethyl-ethylbenzyl-ammonium chloride and 0.25% n-alkyl-dimethyl-benzyl-ammonium chloride).	Not detected before or after disinfection.
Saha 2016 (3190)	Gram-negative bacteria	Single-site, quasi-experimental, controlled cohort study over a 6-week period	An unspecified number of samples were taken from 20 identical frequent touch points in two wards, including patient beds, tables, chairs and curtain rails, telephone keypad, computer keyboard and mouse; toilet washbasin rims, taps, and nozzles; shower handles,	Baseline measurement was taken the week prior to trial period of the study. Routine cleaning (control) on one ward with alcohol-free QAC wipes (≤0.5% cocoalkyl dimethylbenzyl ammonium chloride; Tuffie 5 Wipes) and 70% isopropyl alcohol wipes (Sani-Cloth 70; PDI) was compared to an intervention cleaning on a second ward with wipes producing peracetic acid when wet (sodium percarbonate ≤50% by weight, and citric acid ≤20% by weight; Clinell sporicidal wipes; GAMA Healthcare). Samples were collected within one	Detection of gram-negative indicator organism in both wards were similarly low during baseline period (p=0.31). After intervention, the control ward (alcohol-free QAC wipes) had significantly higher surfaces positive at 17/100 (17%) compared to intervention with 4/100 (4%) surfaces positive (p=0.003).

Reference (Study ID)	Outcome	Study Design	Setting	Intervention	Results
			commodes, and door handles and light switches). The samples were taken from 2 matched 29-bed elderly care wards in a hospital in London, UK	hour of intervention. Contact time not specified.	
Evans 2007 (9347)	Gram-negative bacteria	Single-site, Quasi-experimental, controlled before-after study	5 treatment tables from chiropractic outpatient teaching facility. Texas, USA.	Each table received two sterilizing agents: the left half received treatment with a pre-packaged alcohol wipe containing 70% isopropyl alcohol and 10% acetone while the right side received treatment with QAC (Lysol Brand, <1% 80% benzalkonium chloride) sanitizing wipes. Once treated, each side was allowed to completely dry before a sample was taken. Baseline samples were taken prior to disinfection.	Prior to disinfection, two of five tables were positive for gram-negative organisms. After disinfection, none reported. Bacterial counts were not reported.
Strassle 2012 (2922)	Gram-negative bacteria (*Acinetobacter baumannii*-MDR)	Single-site, quasi-experimental study over a 48-month period	A total of 487 samples were taken from 10 surfaces in 31 rooms, including the sink drain and edge of basin, buttons on bed rails, bedside table handle, vital sign monitor buttons, floor on either side of the bed, in the medical, surgical, and cardiac surgery ICUs of a hospital in MD, USA	Baseline measurements were taken after patient discharge, prior to cleaning with saturated wipe, and after (time not specified) cleaning. The study examined the efficacy of a wipe saturated with low-alcohol QAC (8.2% n-alkyl dimethyl benzyl ammonium chloride, 8.7% didecyl dimethyl ammonium chloride, and 2.9% ethyl alcohol; Virex II 256; Diversey) with a wet contact time of 8-10 minutes. Final dilution not specified.	The number of rooms positive for MDR-*A. baumannii* was significantly lower in 8/32 rooms (25.0%) after cleaning with low-alcohol QAC vs. 15/32 rooms (46.9%) before cleaning (p=0.01). 41/268 total samples (15.3%) were positive before cleaning vs 12/219 samples (5.5%) positive after (p>0.01). Sites with persistent contamination included the floor, bedside table, call button, door handles, and supply cart. Bedrails and ventilators had no positive samples following cleaning.
Rutala 2018 (10553)	Gram-negative bacteria	Multi-site, controlled cohort	7,360 samples from environmental surfaces (bed rail,	Compared standard cleaning with quaternary ammonium compound disinfection in 21 randomly selected	Mean concentration per room for MDR *Acinetobacter* was significantly higher

Reference (Study ID)	Outcome	Study Design	Setting	Intervention	Results
	(*Acinetobacter* spp.-MDR)	study over 27-month period	over-bed table, supply or medicine cart, chair sink, toilet seat, shower floor, side counter, linen hamper lid, bathroom floor) in 92 rooms of 3 university-affiliated hospitals in North Carolina, USA	rooms with standard cleaning with bleach in 20 randomly selected rooms. Active ingredients and contact time were not specified. Samples were collected after disinfection (not specified).	(p=0.035) using QAC compared to bleach with 8.95 compared to 0.39 CFU.
Panknin 2014 (4960)	Gram-negative bacteria (*Acinetobacter* spp.)	Single site, quasi-experimental uncontrolled before-after study over 2 months	147 samples collected from flat surfaces (incubator, heater, ventilator) in patient rooms in the neonatal intensive care unit at a university children's hospital. Sacramento, CA, USA.	Samples were collected before and after intensive terminal disinfection with 3% final concentration of high-alcohol QAC (Super Sani-Cloth, PDI, 55.5% isopropyl alcohol, 0.25% n-alkyl-dimethyl-ethylbenzyl-ammonium chloride and 0.25% n-alkyl-dimethyl-benzyl-ammonium chloride).	Average gene density was not significantly different before compared to after disinfection.
Roux 2013 (3147)	Gram-negative bacteria (*Enterobacteriaceae*-Extended-spectrum beta-lactamase-producing *Enterobacteriaceae* (ESBLE))	Multi-site, cross-sectional cohort study, 4 weeks	185 samples (handwashing sink drains) from 13 ICUs of 7 hospitals and 1 surgical clinic in Tours France.	Routine disinfection was reported for daily disinfection with bleach compared to daily disinfection with quaternary ammonium compounds. Products, active ingredients, contact times not specified. Volume of disinfecting product varied from 25mL of pure product to several liters of variously diluted solutions.	The number of sinks positive for ESBL *Enterobacteriaceae* was significantly higher (p=0.002) when using QAC (20/56) compared to using bleach (0/19).
Panknin 2014 (4960)	Gram-negative bacteria (*Escherichia* spp.)	Single site, quasi-experimental uncontrolled before-after study over 2 months	147 samples collected from flat surfaces (incubator, heater, ventilator) in patient rooms in the neonatal intensive care unit at a university children's	Samples were collected before and after intensive terminal disinfection with 3% final concentration of high-alcohol QAC (Super Sani-Cloth, PDI, 55.5% isopropyl alcohol, 0.25% n-alkyl-dimethyl-ethylbenzyl-ammonium chloride and 0.25% n-alkyl-dimethyl-benzyl-ammonium chloride).	Average gene density was not significantly different before compared to after disinfection.

Reference (Study ID)	Outcome	Study Design	Setting	Intervention	Results
			hospital. Sacramento, CA, USA.		
Otter 2007 (13449)	Gram-negative bacteria (Gentamicin-resistant gram-negative rod)	Single-site, quasi-experimental, uncontrolled before-after study for 19 days	90 samples were taken from 15 sites (floor, beside the bed, floor corner, bed-frame, bed-elevation control panel, bedside chair, bedside locker, over-bed table, remote control, door handle, etc.) in one room in a 500-bed teaching hospital. London, UK.	Terminal cleaning included a QAC disinfectant-detergent (HP800, PVA Hygiene Ltd, unknown active ingredient). HPV was implemented as an adjunct decontamination in one room (Bioquell, 30 min at 20 g/min for two cycles, peak HPV 530-540 ppm). Sampling was taken before and after terminal cleaning and after HPV decontamination.	Number (percent) surfaces positive before vs. after terminal cleaning vs. after HPV: 9 (30%) vs. 3 (10.0%) vs. 0. GNR remained undetected after 1, 2, 5, and 6 days after HPV. Most of the GNR cultured were *Acinetobacter* spp or *Klebsiella* spp. Significance not assessed.
Otter 2016 (4992)	Gram-negative bacteria (Gram-negative rods)	Single site, uncontrolled before-after study over 19 days	225 samples from 15 sites (floor, bedframe, chair, overbed table, door handle, toilet floor, remote control, etc.) in single occupancy room with same patient at university hospital. London, UK.	Terminal cleaning consisted of QAC disinfectant (HP800, PVA Hygiene, active ingredient, contact time not specified). HPV decontamination (BIOQUELL, peak 530-540 ppm) conducted twice in patient room. Samples were collected before terminal cleaning, after terminal cleaning, after HPV decontamination, and subsequent days (up to 19 days) after HPV.	Before terminal cleaning 30% (9/30) samples were positive for MRSA compared to 10% (3/30) after terminal cleaning and 0% (0/30) after HPV. Low prevalence (< 1 sample positive) remained one, two, five, six days after decontamination.
Panknin 2014 (4960)	Gram-negative bacteria (*Klebsiella* spp.)	Single site, quasi-experimental uncontrolled before-after study over 2 months	147 samples collected from flat surfaces (incubator, heater, ventilator) in patient rooms in the neonatal intensive care unit at a university children's hospital. Sacramento, CA, USA.	Samples were collected before and after intensive terminal disinfection with 3% final concentration of high-alcohol QAC (Super Sani-Cloth, PDI, 55.5% isopropyl alcohol, 0.25% n-alkyl-dimethyl-ethylbenzyl-ammonium chloride and 0.25% n-alkyl-dimethyl-benzyl-ammonium chloride).	Average gene density was not significantly different before compared to after disinfection.

Reference (Study ID)	Outcome	Study Design	Setting	Intervention	Results
Panknin 2014 (4960)	Gram-negative bacteria (*Pseudomonas* spp.)	Single site, quasi-experimental uncontrolled before-after study over 2 months	147 samples collected from flat surfaces (incubator, heater, ventilator) in patient rooms in the neonatal intensive care unit at a university children's hospital. Sacramento, CA, USA.	Samples were collected before and after intensive terminal disinfection with 3% final concentration of high-alcohol QAC (Super Sani-Cloth, PDI, 55.5% isopropyl alcohol, 0.25% n-alkyl-dimethyl-ethylbenzyl-ammonium chloride and 0.25% n-alkyl-dimethyl-benzyl-ammonium chloride).	Average gene density significantly higher after disinfection compared to before (p=0.02)
Styaningsih 2019 (2926)	Gram-positive bacilli (*Bacillus subtilis*)	Single-site, quasi-experimental, controlled before-after study.	36 samples were collected from the floor of operating room of surgical unit at a hospital. Kudus City, Indonesia.	The efficacy of two disinfectants was compared: quaternary ammonium derivative (unspecified product or concentration) or sodium hypochlorite (unspecified product or concentration). Sampling was conducted prior to disinfection, after 20 minutes, and after 2 h from disinfection. Efficacy was compared between surgical rooms with centralized or split air conditioning (AC) systems.	Average Bacillus spp before disinfection was 4.00 CFU/cm2 in split AC for sodium hypochlorite and 2.33CFU/cm2 in split AC for QAC. Samples were not positive after disinfection with QAC or sodium hypochlorite (20 min or 2 h). Central AC had lower mean bacterial count.
Deshpande 2014 (7047)	Gram-positive bacilli (*Clostridium difficile*)	Single-site, quasi-experimental, controlled cohort study over a 1-month period	A total of 888 samples were taken from the floors and high-touch surfaces (bed rails, bedside tables) of an unspecified number of CDI and MRSA isolation rooms in a hospital in the USA	Baseline measurements were not specified. Routine cleaning (control) with alcohol-free QAC (<1% n-alkyl dimethyl benzyl ammonium chloride and <1% n-alkyl dimethyl ethylbenzyl ammonium chloride; Virex; Diversey) was mopped on half of floors, and sodium hypochlorite (1:10 dilution of household bleach) was wiped onto high-touch surfaces. The product was compared to enhanced (trial) cleaning with peracetic acid (0.13%) and hydrogen peroxide (0.63%) based sporicidal product (Oxycide; Ecolab), applied in the same fashion to the second half of floors and high-touch	OxyCide and bleach significantly reduced (p<0.05) the recovery of *C. difficile* while the QAC did not significantly reduce recovery of *C. difficile* (p>0.05). In bedside tables and bed rails, there was no recovery of *C. difficile* after OxyCide or bleach disinfection (from 8/50 to 0/50 surfaces positive for OxyCide and from 7/50 to 0/50 surfaces positive for bleach). On floors, there was 5% surfaces positive after disinfection compared to 50% before disinfection with OxyCide (from 41/82 to 4/82 surfaces positive) compared to 54% surfaces positive after QAC and 55% positive before QAC

Reference (Study ID)	Outcome	Study Design	Setting	Intervention	Results
				surfaces. All surfaces were allowed to air-dry 10-15 min. Dilutions and time until measurement were not specified.	disinfection (from 45/82 to 44/82 surfaces positive).
Rutala 2018 (10553)	Gram-positive bacilli (*Clostridium difficile*)	Multi-site, controlled cohort study over 27-month period	7,360 samples from environmental surfaces (bed rail, over-bed table, supply or medicine cart, chair sink, toilet seat, shower floor, side counter, linen hamper lid, bathroom floor) in 92 rooms of 3 university-affiliated hospitals in North Carolina, USA	Compared standard cleaning with quaternary ammonium compound disinfection in 21 randomly selected rooms with standard cleaning with bleach in 20 randomly selected rooms. Active ingredients and contact time were not specified. Samples were collected after disinfection (not specified).	Mean concentration per room for *C. difficile* was not different using QAC compared to bleach at 3.76 compared to 4.48 CFU (p>0.05).
Byers 1998 (882)	Gram-positive cocci (*Enterococcus* spp.-Vancomycin-resistant enterococci (VRE))	Single-sited, quasi experimental, controlled before-after study.	501 sample surfaces (bed rails, telephones, electronic thermometers, IV poles, counters, and floors) 14 hospital rooms with patient with VRE infection from a hospital. Virginia, USA.	Conventional terminal disinfection (before) compared to alternate terminal disinfection (after). Conventional disinfection wiped all surfaces with sprayed cleaning rag with 0.03-0.05% QAC (1:128 dilution with water of alkyl cl12-16 dimethyl benzyl ammonium chloride, dioxyl dimethyl ammonium chloride, and di-decyl dimethyl ammonium chloride, Sanimaster III). Alternate (after) method dipped cleaning rag into a bucket containing the same QAC as the conventional terminal disinfection method and drenching all surfaces. The surfaces were left wet for 10 minutes before being wiped dry with a clean towel. Samples were collected 2 hours after room disinfection.	After one, two, three, and four consecutive conventional disinfection, number (percent) samples positive was 60 (15.9%), 8/82 (9.8%), 3/28 (10.7%) 0/10 (0%) samples, respectively. After switching to the bucket cleaning method, none of 135 sites samples in four hospital rooms grew VRE following a single disinfection (p<0.001).
Eckstein 2007 (899)	Gram-positive cocci (*Enterococcus* spp.-Vancomycin-	Single-site, quasi-experimental, uncontrolled study	A total of 102 microbiological samples were	Baseline measurements were taken within 3 days of patient discharge. Terminal disinfection of rooms	Number (%) rooms positive for environmental cultures was 16/17 (94%) before routine cleaning with low-alcohol

Reference (Study ID)	Outcome	Study Design	Setting	Intervention	Results
	resistant enterococci (VRE))	over a 6-week period	collected from bedrail, bedside table, phone, call button, toilet, and door handle in 26 rooms of patients with either *C. difficile* or VRE colonization in an acute care Veterans Affairs Medical Center in Ohio, USA	(control) using low-alcohol QAC (7-13% didecyldimethyl ammonium chloride, 3-7% alkyl dimethylbenzyl ammonium chloride, 1-5% alcohol, and 1-5% tetrasodium EDTA; Super HDQ Neutral; Spartan) was compared to additional effect of cleaning procedure using 10% bleach solution. Researchers implemented the 10% bleach prior to admission of another patient. Application methods were cloth or mop and disinfectants were allowed to air dry. Dilutions, time until measurement unspecified.	QAC versus 12/17 (71%) after (p=0.125). 72 of 102 total samples (71%) were positive for VRE before cleaning with low-alcohol QAC compared to 58 of 102 (57%) were positive after (significance not specified). Additional cleaning with bleach significantly reduced % surfaces positive to 0 (p<0.001).
Fitton 2017 (1011)	Gram-positive cocci (*Enterococcus* spp.- Vancomycin-resistant enterococci (VRE))	Single-site, quasi-experimental, controlled cohort study over a 5-month period	A total of 1,382 microbiological samples were collected from bedrails, patient call pad, patient tray table, and bedside table drawer handle in 342 patient rooms in the MICU of a community teaching hospital in the USA	Baseline measurements were taken for 7 consecutive days prior to intervention period. A saline solution (control) or alcohol-free QAC (treatment) (0.75% 3-trihydroxysilylpropyldimethyl-octadecylammonium chloride; Goldshield 75; AP Goldshield) were applied to rooms selected for control or treatment as ready-to-use spray. Wet contact time not specified. Product was applied every 30 days, with sampling performed weekly. Sample measurement time relative to disinfection not specified.	Although a reduction was noted during the intervention period with alcohol-free QAC, counts of VRE colonies were too low to observe trends.
Otter 2016 (4992)	Gram-positive cocci (*Enterococcus* spp.- Vancomycin-resistant enterococci (VRE))	Single site, uncontrolled before-after study over 19 days	225 samples from 15 sites (floor, bedframe, chair, overbed table, door handle, toilet floor, remote control, etc.) in single occupancy room with same patient at university	Terminal cleaning consisted of QAC disinfectant (HP800, PVA Hygiene, active ingredient, contact time not specified). HPV decontamination (BIOQUELL, peak 530-540 ppm) conducted twice in patient room. Samples were collected before terminal cleaning, after terminal cleaning, after HPV decontamination, and subsequent days (up to 19 days) after HPV.	One sample was positive for VRE before (1/30) and after (1/30) terminal cleaning. Immediately after HPV, no samples (0/30) were positive for VRE up to 19 days after decontamination.

Reference (Study ID)	Outcome	Study Design	Setting	Intervention	Results
			hospital. London, UK.		
Rutala 2018 (10553)	Gram-positive cocci (*Enterococcus* spp.- Vancomycin-resistant enterococci (VRE))	Multi-site, controlled cohort study over 27-month period	7,360 samples from environmental surfaces (bed rail, over-bed table, supply or medicine cart, chair sink, toilet seat, shower floor, side counter, linen hamper lid, bathroom floor) in 92 rooms of 3 university-affiliated hospitals in North Carolina, USA	Compared standard cleaning with quaternary ammonium compound disinfection in 21 randomly selected rooms with standard cleaning with bleach in 20 randomly selected rooms. Active ingredients and contact time were not specified. Samples were collected after disinfection (not specified).	Mean concentration per room for VRE was higher ($p>0.05$) using QAC compared to bleach with 39.57 compared to 2.43 CFU.
Otter 2007 (13449)	Gram-positive cocci (*Enterococcus* spp.- Vancomycin-resistant enterococci (VRE))	Single-site, quasi-experimental, uncontrolled before-after study for 19 days	90 samples were taken from 15 sites (floor, beside the bed, floor corner, bed-frame, bed-elevation control panel, bedside chair, bedside locker, over-bed table, remote control, door handle, etc.) in one room in a 500-bed teaching hospital. London, UK.	Terminal cleaning included a QAC disinfectant-detergent (HP800, PVA Hygiene Ltd, unknown active ingredient). HPV was implemented as an adjunct decontamination in one room (Bioquell, 30 min at 20 g/min for two cycles, peak HPV 530-540 ppm). Sampling was taken before and after terminal cleaning and after HPV decontamination.	Number (percent) surfaces positive before vs after terminal cleaning vs. after HPV: 1 (6.7%) vs. 1 (6.7%) vs. 0. Significance not assessed.
Panknin 2014 (4960)	Gram-positive cocci (*Enterococcus* spp.)	Single site, quasi-experimental uncontrolled before-after study over 2 months	147 samples collected from flat surfaces (incubator, heater, ventilator) in patient rooms in the neonatal intensive care unit at a university children's hospital.	Samples were collected before and after intensive terminal disinfection with 3% final concentration of high-alcohol QAC (Super Sani-Cloth, PDI, 55.5% isopropyl alcohol, 0.25% n-alkyl-dimethyl-ethylbenzyl-ammonium chloride and 0.25% n-alkyl-dimethyl-benzyl-ammonium chloride).	Average gene density was not significantly different before compared to after disinfection.

Reference (Study ID)	Outcome	Study Design	Setting	Intervention	Results
			Sacramento, CA, USA.		
Deshpande 2014 (7047)	Gram-positive cocci (Gram positive organisms- *Staphylococcus aureus* (MRSA) or *Enterococcus* spp (VRE))	Single-site, quasi-experimental, controlled cohort study over a 1-month period	A total of 888 samples were taken from the floors and high-touch surfaces (bed rails, bedside tables) of an unspecified number of CDI and MRSA isolation rooms in a hospital in the USA	Baseline measurements were not specified. Routine cleaning (control) with alcohol-free QAC (<1% n-alkyl dimethyl benzyl ammonium chloride and <1% n-alkyl dimethyl ethylbenzyl ammonium chloride; Virex; Diversey) was mopped on half of floors, and sodium hypochlorite (1:10 dilution of household bleach) was wiped onto high-touch surfaces. The product was compared to enhanced (trial) cleaning with peracetic acid (0.13%) and hydrogen peroxide (0.63%) based sporicidal product (Oxycide; Ecolab), applied in the same fashion to the second half of floors and high-touch surfaces. All surfaces were allowed to air-dry 10-15 min. Dilutions and time until measurement were not specified.	OxyCide and bleach significantly reduced ($p<0.05$) recovery of MRSA and/or VRE, but not QAC ($p>0.05$). In bedside tables and bed rails, there was no recovery of MRSA/VRE after OxyCide or bleach disinfection (from 11/50 to 0/50 surfaces positive for OxyCide and from 12/50 to 0/50 surfaces positive for bleach). On floors, there was no recovery after OxyCide disinfection compared to 18% positive before (from 7/40 to 0/40 surfaces positive) compared to 18% surfaces positive after QAC and 25% surfaces positive before from 10/40 to 7/40 surfaces positive).
Evans 2007 (9347)	Gram-positive cocci (Gram-positive bacteria)	Single-site, Quasi-experimental, controlled before-after study	5 treatment tables from chiropractic outpatient teaching facility. Texas, USA.	Each table received two sterilizing agents: the left half received treatment with a pre-packaged alcohol wipe containing 70% isopropyl alcohol and 10% acetone while the right side received treatment with QAC (Lysol Brand, <1% 80% benzalkonium chloride) sanitizing wipes. Once treated, each side was allowed to completely dry before a sample was taken. Baseline samples were taken prior to disinfection.	Prior to disinfection, all (n=5) tables were positive for gram-positive organisms including *Staphylococcus* spp. After disinfection, no *Staphylococcus* spp or MRSA were reported. Bacterial counts were not reported.
Santos-Junior 2018 (3236)	Gram-positive cocci (*Staphylococcus aureus*-MRSA)	Single-site, quasi-experimental, uncontrolled before-after study	80 samples from 5 high-touch surfaces (bed rails, bedside table, inner bathroom door handle, toilet	Concurrent cleaning was performed once a day in the morning. Disinfectant used was a QAC based in combination with polymeric biguanide (35-40% dimethyl benzyl ammonium chloride;	The percent (number) of samples positive for MRSA before disinfection was 21.5% (7/28) compared after 10.7% (3/28). Significance not assessed.

Reference (Study ID)	Outcome	Study Design	Setting	Intervention	Results
		for a 4-week period.	bowl rim, toilet flush handle) from 8 rooms in an internal medicine and surgical nursing ward at 45-bed hospital. Mato Grosso do Sul, Brazil.	bigunide polyhexamethylene hydrochloride 4-5%, Nippo-Bac Plus, Nippon Chemical Ind). Surfaces were rubbed with 100% cotton cloths that had been soaked in previously diluted disinfectant solution. Samples were collected twice a week before and after ten minutes of disinfection allowing samples to dry.	
Otter 2016 (4992)	Gram-positive cocci (*Staphylococcus aureus*-MRSA)	Single site, uncontrolled before-after study over 19 days	225 samples from 15 sites (floor, bedframe, chair, overbed table, door handle, toilet floor, remote control, etc.) in single occupancy room with same patient at university hospital. London, UK.	Terminal cleaning consisted of QAC disinfectant (HP800, PVA Hygiene, active ingredient, contact time not specified). HPV decontamination (BIOQUELL, peak 530-540 ppm) conducted twice in patient room. Samples were collected before terminal cleaning, after terminal cleaning, after HPV decontamination, and subsequent days (up to 19 days) after HPV.	Before terminal cleaning 60% (18/30) samples were positive for MRSA compared to 40% (12/30) after terminal cleaning and 3.3% (1/30) after HPV. Low prevalence (< 1 sample positive) remained one and two days after decontamination.
Kitagawa 2020 (5623)	Gram-positive cocci (*Staphylococcus aureus*-MRSA)	Single-site, quasi-experimental, uncontrolled before-after study over a 5-month period	204 samples from high -touch surfaces (bed rail, bed control panel, overbed table, vital sign monitor control panel, infusion pump control panel, bedside table, door handle, sink counter) from 11 rooms in the ICU, emergency ICU, and high care unit of a 740-bed tertiary care hospital. Hiroshima, Japan.	Baseline samples were collected after patient discharge, prior to daily standard manual cleaning. Manual cleaning of surfaces was performed with wipes containing 0.5% benzalkonium chloride (Seifukipu; Kao Corporation, Tokyo, Japan). Samples were collected after cleaning once surfaces were dry.	Percent surfaces positive was significantly lower ($p<0.001$) at 19.6% after manual cleaning compared to before at 42.2%. Compared with the baseline, manual cleaning reduced average MRSA counts by 80.7%. Overall, mean concentration was lower after manual cleaning from 5.7 ± 2.1 CFU to 1.1 ± 3.9 CFU. Median (range) concentration was significantly lower after cleaning compared to before from 4.0 CFU (0-245) to 1.0 CFU (0-200).

Reference (Study ID)	Outcome	Study Design	Setting	Intervention	Results
Rutala 2018 (10553)	Gram-positive cocci (*Staphylococcus aureus*-MRSA)	Multi-site, controlled cohort study over 27-month period	7,360 samples from environmental surfaces (bed rail, over-bed table, supply or medicine cart, chair sink, toilet seat, shower floor, side counter, linen hamper lid, bathroom floor) in 92 rooms of 3 university-affiliated hospitals in North Carolina, USA	Compared standard cleaning with quaternary ammonium compound disinfection in 21 randomly selected rooms with standard cleaning with bleach in 20 randomly selected rooms. Active ingredients and contact time were not specified. Samples were collected after disinfection (not specified).	Mean count per room for MRSA was higher ($p>0.05$) using QAC compared to bleach at 8.52 CFU compared to 4.39 CFU.
Fujii 1996 (11965)	Gram-positive cocci (*Staphylococcus aureus*-MRSA)	Single-site, quasi-experimental, controlled before-after study.	An unstated number of samples were taken from the floors of patient rooms in a neurosurgery ward at a university hospital. Yamaguchi, Japan.	Baseline measurements were taken prior to mopping. Floors were mopped with QAC benzalkonium chloride (Osvan) in concentrations of 0.2% and 0.5%. Product was compared to hydrochloride solution of alkyldiaminoethyl glycine (zwitterionic surfactant; Tego-51) at 0.2% concentration, as well as chlorhexidine digluconate (Hibitane) in concentrations of 0.2% and 0.5%. This was a cohort study and no products were designated as the control. Contact time, time until measurement after disinfection were not specified.	MRSA was detected following disinfection with alkyldiaminioethyl glycine, 0.2% chlorhexidine digluconate, and 0.2% benzalkonium chloride; it was not detected when concentration was increased to 0.5% benzalkonium chloride and 0.5% chlorhexidine digluconate (significance not specified).
Otter 2007 (13449)	Gram-positive cocci (*Staphylococcus aureus*-MRSA)	Single-site, quasi-experimental, uncontrolled before-after study for 19 days	90 samples were taken from 15 sites (floor, beside the bed, floor corner, bed-frame, bed-elevation control panel, bedside chair, bedside locker, over-bed table, remote control, door handle, etc.) in one	Terminal cleaning included a QAC disinfectant-detergent (HP800, PVA Hygiene Ltd, unknown active ingredient). HPV was implemented as an adjunct decontamination in one room (Bioquell, 30 min at 20 g/min for two cycles, peak HPV 530-540 ppm). Sampling was taken before and after terminal cleaning and after HPV decontamination.	Number (percent) surfaces positive before vs. after terminal cleaning vs. after HPV: 18 (60%) vs. 12 (40.0%) vs. 1 (3.3%). Surfaces had low MRSA after 1, 2 days after HPV but increased at 5 days post-HPV decontamination. Significance not assessed.

Reference (Study ID)	Outcome	Study Design	Setting	Intervention	Results
			room in a 500-bed teaching hospital. London, UK.		
Bogusz 2013 (14746)	Gram-positive cocci (*Staphylococcus aureus*-MSSA, MRSA)	Single-site, quasi-experimental study over a period of 3 months	A total of 360 samples were taken from 4 surface types, including bedside locker, left bedrail, overbed table, and right bedrail. Samples were taken from 30-bed elder care ward in an NHS hospital in the UK	Tuffie detergent wipes, Vernacare, Bolton, UK were studied for their efficacy in reducing ACC, MSSA, and MRSA. Samples were taken 1, 2, 4, 8, 12, 24, and 48 hours after intervention.	Total MSSA & MRSA count at all sites and all phases was 12 prior to disinfection, then 8, 2, 2, 3, 3, 4, and 9 at 1, 2, 4, 8, 12, 24, and 48 hours' post-intervention, respectively. Reduction in levels of MSSA/MRSA was significant 2-4 hours after disinfection (p=0.014).
Gonzalez 1963 (1626)	Gram-positive cocci (*Staphylococcus aureus*)	Single-sited, quasi experimental, uncontrolled before-after study for a 5-week period.	110 surface samples (floor, incubators, beds, mattresses, night tables, lamps, etc.) from the crib room of the maternity wing and 50 surface samples from staff areas and 13 patient rooms of pneumology wing of the General Hospital. Specific location not stated.	Efficacy of a 2:1000 germicide QAC solution (after) (Biomet 66, N-alkyl dimethyl chloride (benzyl ammonium chloride)-25%, Bis oxide (tri-n-butyl tin)-5%) was assessed. Biomet 66 was applied by spray pump for two consecutive days (maternity wing) or daily for 5 weeks (pneumology wing). Sampling was conducted at baseline (before) and then 3 days after intervention.	In the maternity wing, percent surfaces positive was 56% before the use of the germicide to 48% afterwards. In the pneomology wing's staff area percent surfaces positive was unchanged at 18% before and 18% after disinfection while in patient rooms was 46% before and 22% after disinfection. Significance not specified.
Styaningsih 2019 (2926)	Gram-positive cocci (*Staphylococcus aureus*)	Single-site, quasi-experimental, controlled before-after study.	36 samples were collected from the floor of operating room of surgical unit at a hospital. Kudus City, Indonesia.	The efficacy of two disinfectants was compared: quaternary ammonium derivative (unspecified product or concentration) or sodium hypochlorite (unspecified product or concentration). Sampling was conducted prior to disinfection, after 20 minutes, and after 2 h from disinfection. Efficacy was compared between surgical rooms with	Average S. *aureus* before disinfection was 7.00 CFU/cm2 in split AC for sodium hypochlorite and 6.33CFU/cm2 in split AC for QAC. Samples were not positive after disinfection with QAC or sodium hypochlorite (20 min or 2 h). Central AC had lower mean bacterial count.

Reference (Study ID)	Outcome	Study Design	Setting	Intervention	Results
				centralized or split air conditioning (AC) systems.	
Santos-Junior 2018 (3236)	Gram-positive cocci (*Staphylococcus aureus*)	Single-site, quasi-experimental, uncontrolled before-after study for a 4-week period.	80 samples from 5 high-touch surfaces (bed rails, bedside table, inner bathroom door handle, toilet bowl rim, toilet flush handle) from 8 rooms in an internal medicine and surgical nursing ward at 45-bed hospital. Mato Grosso do Sul, Brazil.	Concurrent cleaning was performed once a day in the morning. Disinfectant used was a QAC based in combination with polymeric biguanide (35-40% dimethyl benzyl ammonium chloride; bigunide polyhexamethylene hydrochloride 4-5%, Nippo-Bac Plus, Nippon Chemical Ind). Surfaces were rubbed with 100% cotton cloths that had been soaked in previously diluted disinfectant solution. Samples were collected twice a week before and after ten minutes of disinfection allowing samples to dry.	The range of the median counts on all surface types for S. *aureus* was 1 – 17.5 CFU before compared to 0 – 16 CFU after. Significant reduction was observed at toilet flush handles (initial median=10.5 CFU) p=0.04
Butin 2019 (6287)	Gram-positive cocci (*Staphylococcus* capitis (methicillin-resistant clone NRCS-A))	Single-site, quasi-experimental, uncontrolled study over a 6-week period	A total of 288 samples were taken from 9 sites (button, handles, window, scale, mattress) on 16 incubators, from the NICU of a neonatal hospital in France.	Baseline samples were taken before disinfection. The study examined the efficacy of a solution containing alcohol-free QAC with proprietary agents (didecyldimethyl ammonium chloride at 82 mg/g by weight, chlorhexidine digluconate at 5 mg/g by weight, and polyhexamethylene biguanide chlorhydrate at 0.24 mg/g by weight; ANIOSURF Premium; Anios) at a final dilution of 0.25%, in reducing methicillin-resistant *S. capitis* clone strain NRCS-A on incubators following an immersion time of 20 minutes. Items that could not be submerged were disinfected using wet wipes impregnated with the same disinfectant solution. Time until measurement not specified.	All 16/16 incubators (100%) in at least one site and 63 samples (44%) were positive for *S. capitis* prior to disinfection with alcohol-free QAC compared to 10/16 (62%) incubators and 16 (11%) samples positive after (significance not specified). The majority of samples positive after disinfection were from sites that could not be immersed in the disinfectant solution.
Styaningsih 2019 (2926)	Gram-positive cocci (*Staphylococcus epidermis*)	Single-site, quasi-experimental,	36 samples were collected from the floor of operating	The efficacy of two disinfectants was compared: quaternary ammonium derivative (unspecified product or	Average S. epidermidis before disinfection was 8.33 CFU/cm2 in split AC for sodium hypochlorite and 7.67

Reference (Study ID)	Outcome	Study Design	Setting	Intervention	Results
		controlled before-after study.	room of surgical unit at a hospital. Kudus City, Indonesia.	concentration) or sodium hypochlorite (unspecified product or concentration). Sampling was conducted prior to disinfection, after 20 minutes, and after 2 h from disinfection. Efficacy was compared between surgical rooms with centralized or split air conditioning (AC) systems.	CFU/cm2 in split AC for QAC. Samples were not positive after disinfection with QAC or sodium hypochlorite (20 min or 2 h). Central AC had lower mean bacterial count. Disinfectants were not significantly different.
Sigler 2013 (2781)	Gram-positive cocci (*Staphylococcus* spp.)	Single-site, quasi experimental, uncontrolled before-after study.	129 samples were collected before (n=81) and after (n=48) disinfection from 9 surfaces (sink area, bed rails, floor, overbed table, television button panel, call light, telephone, dry-erase erasers, and patient information binder) from each of ten, single-patient isolation rooms in a 250-bed university hospital. Ohio, USA.	Surfaces were sampled before, and within 15 min after the established hospital disinfection protocol which included an automatically dispensed, hospital-approved quaternary ammonia product (no other specifications). Microfiber cloths or mops were frequently changed and cloths were not returned to the cleaning solution.	24/81 samples (30%) were PCR positive for *Staphylococcus* spp. genetic marker before disinfection. With the exception of one surface, multiple *Staphylococcus* spp genes were detected in each surface sample before and after cleaning.
Yuen 2015 (10160)	Gram-positive cocci (*Staphylococcus* spp.- MSSA, MRSA, coagulase-negative *Staphylococcus*)	Single site, uncontrolled before-after study, over 6-week period	864 samples from 4 high-touch bedside surfaces in a 6-bed cubical of a medical ward at a 1500-bed teaching hospital. Hong Kong, China.	The control periods included weeks 1, 3, and 5 and consisted of routine disinfection with sodium hypochlorite wipes once per day. The intervention periods included weeks 2, 4, and 6 and included routine disinfection followed by an applied QAC spray (JUC spray) to all bed-units in the cubicle three times per week. Samples were always collected 1 h and 5 h after routine cleaning. During the intervention periods, the JUC spray was applied immediately after samples were	During control periods, 78% (14/18) beds were positive for staphylococcal bacteria. During the intervention periods, 11% (2/18) were positive (significance not specified). In the control period, 5 h after hypochlorite disinfection, mean staphylococcal contamination increased significantly 80% ($p<0.01$) while it decreased significantly in the intervention group with the application of the JUC spray ($p<0.001$). In the experimental group the mean staphylococcal concentration

Reference (Study ID)	Outcome	Study Design	Setting	Intervention	Results
				collected 1 h after routine disinfection. The same ward and 6-bed cubicle were used during the intervention and control periods.	of the bedside surfaces was 4.4 CFU/cm2 1 h after hypochlorite disinfection (before JUC spray) to 0.7 CFU/cm2 (5 h after hypochlorite and 4 h after JUC spray). In the control periods, mean concentration at 1 h after hypochlorite was ~1.5 CFU/cm2 and at 5 h after hypochlorite was ~4.1 CFU/cm2 (significant increase of 80%, p<0.01).
Fitton 2017 (1011)	Gram-positive cocci (*Staphylococcus* spp.)	Single-site, quasi-experimental, controlled cohort study over a 5-month period	A total of 1382 microbiological samples were collected from bedrails, patient call pad, patient tray table, and bedside table drawer handle in 342 patient rooms in the MICU of a community teaching hospital in the USA	Baseline measurements were taken for 7 consecutive days prior to intervention period. A saline solution (control) or alcohol-free QAC (treatment) (0.75% 3-trihydroxysilylpropyldimethyl-octadecylammonium chloride; Goldshield 75; AP Goldshield) were applied to rooms selected for control or treatment as ready-to-use spray. Wet contact time not specified. Product was applied every 30 days, with sampling performed weekly. Sample measurement time relative to disinfection not specified.	Mean 5-month reduction in total *Staphylococcus* was 76.3% from baseline following the use of alcohol-free QAC compared to 40.7% for placebo. A significant reduction was achieved between the treatment and placebo groups (p=0.02) for total *Staphylococcus*.
Panknin 2014 (4960)	Gram-positive cocci (*Staphylococcus* spp.)	Single site, quasi-experimental uncontrolled before-after study over 2 months	147 samples collected from flat surfaces (incubator, heater, ventilator) in patient rooms in the neonatal intensive care unit at a university children's hospital. Sacramento, CA, USA.	Samples were collected before and after intensive terminal disinfection with 3% final concentration of high-alcohol QAC (Super Sani-Cloth, PDI, 55.5% isopropyl alcohol, 0.25% n-alkyl-dimethyl-ethylbenzyl-ammonium chloride and 0.25% n-alkyl-dimethyl-benzyl-ammonium chloride).	Average gene density was not significantly different before compared to after disinfection.
Panknin 2014 (4960)	Gram-positive cocci (*Streptococcus* spp.)	Single site, quasi-experimental uncontrolled	147 samples collected from flat surfaces (incubator,	Samples were collected before and after intensive terminal disinfection with 3% final concentration of high-alcohol	Average gene density was significantly higher before disinfection compared to after (p=0.002)..

Reference (Study ID)	Outcome	Study Design	Setting	Intervention	Results
		before-after study over 2 months	heater, ventilator) in patient rooms in the neonatal intensive care unit at a university children's hospital. Sacramento, CA, USA.	QAC (Super Sani-Cloth, PDI, 55.5% isopropyl alcohol, 0.25% n-alkyl-dimethyl-ethylbenzyl-ammonium chloride and 0.25% n-alkyl-dimethyl-benzyl-ammonium chloride).	
Hacek 2010 (5852)	HAI (*Clostridium difficile*)	Multi-site, Quasi-experimental uncontrolled before-after study over 3 years	Monthly *C. difficile* infections were collected from the hospital databases and defined as having a positive stool test > 48 h after admission. The study was conducted in 3-hospital system in Illinois, USA	During the entire study period daily cleaning of patient rooms used a QAC (unspecified product, active ingredients). During the 10-month pre-intervention period, QAC was also used for terminal disinfection. The 2-year intervention period replaced QAC with 5000 ppm bleach during terminal cleaning.	There was a significant reduction (p<0.0001) in average number of CDI patients per 1000 patient-days during the intervention period with 0.85 in the pre-intervention period (QAC terminal cleaning) compared to 0.45 (bleach terminal cleaning).
Garvey 2018 (6482)	HAI (HAI-Methicillin-resistant *Staphylococcus aureus* (MRSA))	Single-site, quasi-experimental, controlled cohort study over a period of 4.5 years	The study examined 2,146,651 total patient bed days for the effect of environmental cleanings on HAIs related to MRSA. The study was performed in all wards of a tertiary care teaching hospital in the UK	Per protocol, the bed frame, nurse-call handset, door handles, bedside chair, and other surfaces were cleaned. Baseline (control arm) period consisted of 3 years and included two-wipe system consisting of a detergent wipe (<1% phenoxyethanol, <0.2% alkyl polyglycoside, <0.1% diethylene glycol, and <0.1% 2-octyl-2H-isothiazol-3-one; manufacturer not specified) followed by an alcohol wipe (50-80% propan-2-ol; manufacturer not specified). A single-wipe system (trial arm) was implemented and measured for 9 months, and included wipes containing alcohol-free QAC with proprietary ingredients (≤0.5% benzalkonium chloride, ≤0.5% didecyl dimethyl ammonium chloride, ≤0.10%	The number of MRSA acquisitions during the period of use of alcohol-free QAC wipes was 92 in 989,724 patient-bed days, representing a 6.3% reduction of the rate of MRSA infections each month, following implementation of wipes. The average MRSA acquisition was 9.4 per 100,000 patient-bed days during this time, compared to 20.7 per 100,000 patient-bed days with baseline cleaning (p<0.05).

Reference (Study ID)	Outcome	Study Design	Setting	Intervention	Results
				polyhexamethylene biguanide (PHMB); Clinell Universal Sanitising Wipes; GAMA Healthcare Limited). Wet contact time not specified.	
Anderson 2018 (6885)	HAI (HAI-Methicillin-resistant *Staphylococcus aureus* (MRSA))	Multisite, quasi-experimental, cohort study over a period of 28 months	Hospital-wide HAI acquisition over 271740 patients and 375918 admissions 9 hospitals including university, tertiary care, regional, community, and VA hospital settings, USA	Four experimental periods of 6 months each, with a 1-month "wash" period between each using different terminal disinfection. Standard terminal disinfection (reference) was performed using a low-alcohol solution QAC (6.5% octyl decyl dimethyl ammonium chloride, 2.6% dioctyl dimethyl ammonium chloride, 3.9% didecyl dimethyl ammonium chloride, 8.7% alkyl dimethyl benzyl ammonium chloride; Quaternary Disinfectant Cleaner; Ecolab) applied with microfiber cloth at an unspecified final dilution. Standard terminal cleaning was compared to 3 enhanced protocol methods including QAC disinfection followed by UV light disinfection; bleach disinfection (10% hypochlorite, Clorox Germicidal Wipes); and bleach disinfection followed by UV light disinfection. Wet contact time, time until measurement unspecified.	For standard disinfection period/reference period with QAC disinfection, hospital-wide incident cases of MRSA infections were 204 (0.27% of exposed admissions) with 5.66 per 10,000 patient-days. During the bleach-only disinfection period, incidence was 5.88 per 10,000 patient days and relative risk (95% CI) was not significantly different at 0.97 (0.76-1.24), p=0.82.
Anderson 2018 (6885)	HAI (HAI-All target pathogens)	Multisite, quasi-experimental, cohort study over a period of 28 months	Hospital-wide HAI acquisition over 271740 patients and 375918 admissions 9 hospitals including university, tertiary care, regional, community, and VA hospital settings, USA	Four experimental periods of 6 months each, with a 1-month "wash" period between each. Standard terminal disinfection (control) was performed using a low-alcohol solution QAC (6.5% octyl decyl dimethyl ammonium chloride, 2.6% dioctyl dimethyl ammonium chloride, 3.9% didecyl dimethyl ammonium chloride, 8.7% alkyl dimethyl benzyl ammonium chloride; Quaternary Disinfectant	For standard disinfection period/reference period with QAC disinfection, hospital-wide incident cases of all targeted organisms (MRSA, VRE, MDR *Acinetobacter* spp., *C. difficile*) infections were 626 (0.86% of exposed admissions) with 18.1 per 10,000 patient-days. During the bleach-only disinfection period, incidence was 17.5 per 10,000 patient days and relative

Reference (Study ID)	Outcome	Study Design	Setting	Intervention	Results
				Cleaner; Ecolab) applied with microfiber cloth at an unspecified final dilution. Standard terminal cleaning was compared to 3 enhanced protocol methods including QAC disinfection followed by UV light disinfection; bleach disinfection; and bleach disinfection followed by UV light disinfection. Wet contact time, time until measurement unspecified.	risk (95% CI) was not significantly different at 0.92 (0.79-1.08), p=0.32.
Anderson 2017 (6887)	HAI (HAI-All target pathogens)	Multi-site, controlled cohort study. 27- month period	21,395 patients met inclusion criteria as an exposed patient who was admitted to room with prior occupant with proven infection or colonization with organism. 4916 patients from reference group, 5438 in bleach group. Conducted at nine hospitals including tertiary, community, Veterans Affairs hospitals in the southeastern USA.	Four experimental periods of 6 months each, with a 1-month "wash" period between each. Standard terminal disinfection (control) was performed using a low-alcohol solution QAC (6.5% octyl decyl dimethyl ammonium chloride, 2.6% dioctyl dimethyl ammonium chloride, 3.9% didecyl dimethyl ammonium chloride, 8.7% alkyl dimethyl benzyl ammonium chloride; Quaternary Disinfectant Cleaner; Ecolab) applied with microfiber cloth at an unspecified final dilution. Standard terminal cleaning was compared to 3 enhanced protocol methods including QAC disinfection followed by UV light disinfection; bleach disinfection; and bleach disinfection followed by UV light disinfection. Wet contact time, time until measurement unspecified.	Incidence for reference/QAC was 115 cases among exposed patients (2.3%) compared to incidence among bleach group of 101 (1.9%). Rate was 51.3/10,000 patient-days for QAC compared to 41.6/10,000 patient-days for bleach. Incidence was not significantly different with rate ratio (95% CI) was 0.85 (0.69-1.04) for disinfection with bleach compared to QAC for all target organisms (*C. difficile*, MRSA, VRE, MDR *Acinetobacter*).
Boyce 2017 (808)	HAI (HAI-*Clostridium difficile*)	Single-site, quasi-experimental controlled crossover study; 12-months	11,490 patient-days for QAC disinfectant; 10,741 patient-days for IHP disinfectant from 2 general wards in a Medical intensive care unit	Daily and discharge cleaning using QAC disinfectant (Hyperfect 256; Genesan, Gorham, ME) with dry wipes made of melt blown polypropylene. 12-month crossover trial compared QAC with 0.5% improved hydrogen peroxide (IHP) disinfectant. Contact time was	Incidence density rates were lower (significance not specified) on IHP wards compared to QAC wards with 0.56 cases per 1000 patient-days on IHP wards compared to 1.0 cases per 1000 patient-days on QAC wards.

Reference (Study ID)	Outcome	Study Design	Setting	Intervention	Results
			(MICU) in Yale New-Haven Hospital in New Haven. Connecticut, USA.	not specified. Surveillance of HAIs based on clinical data from hospital.	
Mayfield 2000 (8380)	HAI (HAI-*Clostridium difficile*)	Single site, quasi-experimental, uncontrolled before-after study for a 20-month period.	All patients in the bone marrow transplantation unit (n=293), the neurosurgical intensive care unit (ICU) (n=1278), and a general medicine unit (n=2881) at Barnes-Jewish Hospital, a 2-campus 1287-bed tertiary care university-affiliated facility. Missouri, USA.	Routine baseline cleaning with quaternary ammonium solution for 9 months (period 1) was switched to routine cleaning with unbuffered 1:10 hypochlorite solution for 9 months (contact time not specified) (period 1) and switched back to routine daily cleaning with a quaternary ammonium solution with a 5-min contact time, followed by vigorous rubbing (period 3)	Among the bone marrow transplant patients (n=293), CDAD incident rate decreased significantly from 8.6 to 3.3 cases per 1000 patient days (period 1 to period 2). No significant reduction in CDAD rates were seen in the other units from period 1 to period 2: neurosurgical ICU (from 3.0 to 2.7 cases per 1000 patient-days) and general medicine unit (from 1.3 to 1.5 cases per 1000 patient-days). CDAD rate for bone marrow transplant patients increased to 8.1 cases from 3.2 cases per 1000 patient –days from period 2 to period 3 after replacing hypochlorite with quaternary ammonium disinfectant.
Anderson 2017 (6887)	HAI (HAI-MDR *Acinetobacter* spp)	Multi-site, controlled cohort study. 27- month period	21,395 patients met inclusion criteria as an exposed patient who was admitted to room with prior occupant with proven infection or colonization with organism. 4916 patients from reference group, 5438 in bleach group. Conducted at nine hospitals including tertiary, community, Veterans Affairs hospitals in the southeastern USA.	Four experimental periods of 6 months each, with a 1-month "wash" period between each. Standard terminal disinfection (control) was performed using a low-alcohol solution QAC (6.5% octyl decyl dimethyl ammonium chloride, 2.6% dioctyl dimethyl ammonium chloride, 3.9% didecyl dimethyl ammonium chloride, 8.7% alkyl dimethyl benzyl ammonium chloride; Quaternary Disinfectant Cleaner; Ecolab) applied with microfiber cloth at an unspecified final dilution. Standard terminal cleaning was compared to 3 enhanced protocol methods including QAC disinfection followed by UV light disinfection; bleach disinfection; and bleach	Only one case was observed. No significant difference between bleach and QAC disinfection for MDR Acintenobacter.

Reference (Study ID)	Outcome	Study Design	Setting	Intervention	Results
				disinfection followed by UV light disinfection. Wet contact time, time until measurement unspecified.	
Passaretti 2013 (2322)	HAI (HAI-MDR-gram-negative rods)	Single-site, quasi-experimental controlled study over 30 months	5378 patients at-risk of MDRO acquisition due to prior occupant with MDRO. No surveillance specifically for MDR-GNR in 6 high-risk units (ICU, surgical unit) at 994-bed tertiary referral hospital. Baltimore, USA	3-month pre-intervention phase followed by 6-month intervention phase with HPV (Bioquell, no specific concentration, 1.5 – 3 h) after standard cleaning on 3 units compared to standard cleaning alone with quaternary ammonium compound (active ingredient not specified, 3M) on 3 units. Samples were taken monthly.	Risk of acquisition was lower in HPV cohort compared to non-HPV units, though not statistically significant with risk ratio (95% confidence interval) 0.55 (0.20 – 1.57). MDR-GNR acquisition was 1.2% in HPV units compared to 1.8% in non-HPV units.
Anderson 2017 (6887)	HAI (HAI-MRSA)	Multi-site, controlled cohort study. 27- month period	21,395 patients met inclusion criteria as an exposed patient who was admitted to room with prior occupant with proven infection or colonization with organism. 4916 patients from reference group, 5438 in bleach group. Conducted at nine hospitals including tertiary, community, Veterans Affairs hospitals in the southeastern USA.	Four experimental periods of 6 months each, with a 1-month "wash" period between each. Standard terminal disinfection (control) was performed using a low-alcohol solution QAC (6.5% octyl decyl dimethyl ammonium chloride, 2.6% dioctyl dimethyl ammonium chloride, 3.9% didecyl dimethyl ammonium chloride, 8.7% alkyl dimethyl benzyl ammonium chloride; Quaternary Disinfectant Cleaner; Ecolab) applied with microfiber cloth at an unspecified final dilution. Standard terminal cleaning was compared to 3 enhanced protocol methods including QAC disinfection followed by UV light disinfection; bleach disinfection; and bleach disinfection followed by UV light disinfection. Wet contact time, time until measurement unspecified.	Incidence for reference/QAC was 73 cases among exposed patients (2.2%) compared to incidence among bleach group of 74 (2.0%). Rate was 50.3/10,000 patient-days for QAC compared to 48.2/10,000 patient-days for bleach. Incidence was not significantly different with rate ratio (95% CI) was 1.00 (0.82-1.21) for disinfection with bleach compared to QAC for MRSA.
Boyce 2017 (808)	HAI (HAI-MRSA)	Single-site, quasi-experimental	11,490 patient-days for QAC disinfectant;	Daily and discharge cleaning using QAC disinfectant (Hyperfect 256;	Incidence density rates were lower (significance not specified) on IHP

Reference (Study ID)	Outcome	Study Design	Setting	Intervention	Results
		controlled crossover study; 12-months	10,741 patient-days for IHP disinfectant from 2 general wards in a Medical intensive care unit (MICU) in Yale New-Haven Hospital in New Haven. Connecticut, USA.	Genesan, Gorham, ME) with dry wipes made of melt blown polypropylene. 12-month crossover trial compared QAC with 0.5% improved hydrogen peroxide (IHP) disinfectant. Contact time was not specified. Surveillance of HAIs based on clinical data from hospital.	wards compared to QAC wards with 1.96 cases per 1000 patient-days on IHP wards compared to 2.79 cases per 1000 patient-days on QAC wards.
Passaretti 2013 (2322)	HAI (HAI-MRSA)	Single-site, quasi-experimental controlled study over 30 months	5378 patients at-risk of MDRO acquisition due to prior occupant with MDRO. Weekly surveillance for MRSA, VRE in 6 high-risk units (ICU, surgical unit) at 994-bed tertiary referral hospital. Baltimore, USA	3-month pre-intervention phase followed by 6-month intervention phase with HPV (Bioquell, no specific concentration, 1.5 – 3 h) after standard cleaning on 3 units compared to standard cleaning alone with quaternary ammonium compound (active ingredient not specified, 3M) on 3 units. Samples were taken monthly.	Risk of acquisition was lower in HPV cohort compared to non-HPV units, though not statistically significant with risk ratio (95% confidence interval) 0.53 (0.16 – 1.79). MRSA acquisition was 0.9% in HPV units compared to 2.8% in non-HPV units.
Anderson 2018 (6885)	HAI (HAI-Multidrug-resistant *Acinetobacter* spp. (MDR-A))	Multisite, quasi-experimental, cohort study over a period of 28 months	Hospital-wide HAI acquisition over 271740 patients and 375918 admissions 9 hospitals including university, tertiary care, regional, community, and VA hospital settings, USA	Four experimental periods of 6 months each, with a 1-month "wash" period between each. Standard terminal disinfection (control) was performed using a low-alcohol solution QAC (6.5% octyl decyl dimethyl ammonium chloride, 2.6% dioctyl dimethyl ammonium chloride, 3.9% didecyl dimethyl ammonium chloride, 8.7% alkyl dimethyl benzyl ammonium chloride; Quaternary Disinfectant Cleaner; Ecolab) applied with microfiber cloth at an unspecified final dilution. Standard terminal cleaning was compared to 3 enhanced protocol methods including QAC disinfection followed by UV light disinfection; bleach disinfection; and bleach	For standard disinfection period/reference period with QAC disinfection, hospital-wide incident cases MDR *Acinetobacter* spp. infections were 6 cases (0.01% of exposed admissions) with incidence of 0.18 per 10,000 patient-days. During the bleach-only disinfection period, incidence was 0.11 per 10,000 patient days and relative risk (95% CI) was significantly different ($p<0.05$) at 0.07 (-0.12-0.26).

Reference (Study ID)	Outcome	Study Design	Setting	Intervention	Results
				disinfection followed by UV light disinfection. Wet contact time, time until measurement unspecified.	
Boyce 2017 (808)	HAI (HAI – Total (VRE, MRSA, *C. difficile*))	Single-site, quasi-experimental controlled crossover study; 12-months	11,490 patient-days for QAC disinfectant; 10,741 patient-days for IHP disinfectant from 2 general wards in a Medical intensive care unit (MICU) in Yale New-Haven Hospital in New Haven. Connecticut, USA.	Daily and discharge cleaning using QAC disinfectant (Hyperfect 256; Genesan, Gorham, ME) with dry wipes made of melt blown polypropylene. 12-month crossover trial compared QAC with 0.5% improved hydrogen peroxide (IHP) disinfectant. Contact time was not specified. Surveillance of HAIs based on clinical data from hospital.	Composite incidence density rate for MRSA, VRE, *C. difficile* was 8.0 cases per 1000 patient-days on IHP wards compared to 10.3 cases per 1000 patient-days on QAC wards (p=0.068). Incidence rate ratio = 0.77 (95% confidence interval = 0.579-1.029).
Anderson 2018 (6885)	HAI (HAI-Vancomycin-resistant *Enterococcus* (VRE))	Multisite, quasi-experimental, cohort study over a period of 28 months	Hospital-wide HAI acquisition over 271740 patients and 375918 admissions 9 hospitals including university, tertiary care, regional, community, and VA hospital settings, USA	Four experimental periods of 6 months each, with a 1-month "wash" period between each. Standard terminal disinfection (control) was performed using a low-alcohol solution QAC (6.5% octyl decyl dimethyl ammonium chloride, 2.6% dioctyl dimethyl ammonium chloride, 3.9% didecyl dimethyl ammonium chloride, 8.7% alkyl dimethyl benzyl ammonium chloride; Quaternary Disinfectant Cleaner; Ecolab) applied with microfiber cloth at an unspecified final dilution. Standard terminal cleaning was compared to 3 enhanced protocol methods including QAC disinfection followed by UV light disinfection; bleach disinfection; and bleach disinfection followed by UV light disinfection. Wet contact time, time until measurement unspecified.	For standard disinfection period/reference period with QAC disinfection, hospital-wide incident cases of VRE infections were 121 (0.16% of exposed admissions) with 3.24 per 10,000 patient-days. During the bleach-only disinfection period, incidence was 4.62 per 10,000 patient days and relative risk (95% CI) was not significantly different at 0.87 (0.65-1.17), p=0.35.
Boyce 2017 (808)	HAI (HAI-VRE)	Single-site, quasi-experimental controlled	11,490 patient-days for QAC disinfectant; 10,741 patient-days	Daily and discharge cleaning using QAC disinfectant (Hyperfect 256; Genesan, Gorham, ME) with dry wipes	Incidence density rates were lower (significance not specified) on IHP wards compared to QAC wards with

Reference (Study ID)	Outcome	Study Design	Setting	Intervention	Results
		crossover study; 12-months	for IHP disinfectant from 2 general wards in a Medical intensive care unit (MICU) in Yale New-Haven Hospital in New Haven. Connecticut, USA.	made of melt blown polypropylene. 12-month crossover trial compared QAC with 0.5% improved hydrogen peroxide (IHP) disinfectant. Contact time was not specified. Surveillance of HAIs based on clinical data from hospital.	5.49 cases per 1000 patient-days on IHP wards compared to 6.6 cases per 1000 patient-days on QAC wards.
Passaretti 2013 (2322)	HAI (HAI-VRE)	Single-site, quasi-experimental controlled study over 30 months	5378 patients at-risk of MDRO acquisition due to prior occupant with MDRO. Weekly surveillance for MRSA, VRE in 6 high-risk units (ICU, surgical unit) at 994-bed tertiary referral hospital. Baltimore, USA	3-month pre-intervention phase followed by 6-month intervention phase with HPV (Bioquell, no specific concentration, 1.5 – 3 h) after standard cleaning on 3 units compared to standard cleaning alone with quaternary ammonium compound (active ingredient not specified, 3M) on 3 units. Samples were taken monthly.	MDRO reduction was driven by significant reduction in VRE in HPV units ($p<0.01$) compared to non-HPV units (risk ratio=0.25, 95% confidence interval =0.10, 0.60) between HPV and combined treatment. VRE acquisition was1.7% in HPV units compared to 8.1% in non-HPV units.
Anderson 2017 (6887)	HAI (HAI-VRE)	Multi-site, controlled cohort study. 27- month period	21,395 patients met inclusion criteria as an exposed patient who was admitted to room with prior occupant with proven infection or colonization with organism. 4916 patients from reference group, 5438 in bleach group. Conducted at nine hospitals including tertiary, community, Veterans Affairs hospitals in the southeastern USA.	Four experimental periods of 6 months each, with a 1-month "wash" period between each. Standard terminal disinfection (control) was performed using a low-alcohol solution QAC (6.5% octyl decyl dimethyl ammonium chloride, 2.6% dioctyl dimethyl ammonium chloride, 3.9% didecyl dimethyl ammonium chloride, 8.7% alkyl dimethyl benzyl ammonium chloride; Quaternary Disinfectant Cleaner; Ecolab) applied with microfiber cloth at an unspecified final dilution. Standard terminal cleaning was compared to 3 enhanced protocol methods including QAC disinfection followed by UV light disinfection; bleach disinfection; and bleach disinfection followed by UV light	Incidence for reference/QAC was 37 cases among exposed patients (3.5%) compared to incidence among bleach group of 24 (1.6%). Rate was 63.4/10,000 patient-days for QAC compared to 31.9/10,000 patient-days for bleach. Incidence was significantly different ($p=0.049$) with rate ratio (95% CI) was 0.43 (0.19-1.00) for disinfection with bleach compared to QAC for VRE.

Reference (Study ID)	Outcome	Study Design	Setting	Intervention	Results
				disinfection. Wet contact time, time until measurement unspecified.	
Saha 2016 (3190)	HAI (HAI- Gram-negative organisms)	Single-site, quasi-experimental, controlled cohort study over a 6-week period	Number of patients and HAI surveillance not specified at 2 matched 29-bed elderly care wards in a hospital in London, UK	Weekly HAI data collected. Routine cleaning (control) on one ward with alcohol-free QAC wipes (≤0.5% cocoalkyl dimethylbenzyl ammonium chloride; Tuffie 5 Wipes) on patient equipment and 70% isopropyl alcohol wipes (Sani-Cloth 70; PDI) on nursing station equipment was compared to an intervention cleaning on a second ward with wipes producing peracetic acid when wet (sodium percarbonate ≤50% by weight, and citric acid ≤20% by weight; Clinell sporicidal wipes; GAMA Healthcare) on nursing station and patient equipment. Contact time not specified.	No significant decrease in weekly HAI rate was observed in intervention ward (p=0.31) and control ward (p=0.23). HAIs were lower in the control ward than in the study ward, but these results were not statistically significant.

Table S14: Study results for manually applied sodium hypochlorite interventions ordered by outcome organism

Reference (Study ID)	Outcome	Study Design	Setting	Intervention	Results
Casini 2018 (128)	All viable organisms	Single-site, quasi-experimental, controlled cohort study over a 2-month period	560 samples were taken from 5 high-touch inanimate surfaces per room (bedrails, overbed tables, worktop, infusion pump, monitor). Samples were taken from four patient units in a 12-bed ICU at a university hospital. Pisa, Italy.	A new single-wipe disinfection protocol with alcohol-free QAC-impregnated disposable wipes with proprietary ingredients (≤0.5% benzalkonium chloride, ≤0.5% didecyl dimethyl ammonium chloride, ≤0.10% polyhexamethylene biguanide (PHMB); Clinell Universal Sanitising Wipes; GAMA Healthcare Limited) was compared to a two-step protocol (control) of cloth application of alcohol-based detergent (Keradet-Aktiv; Kiehl, unspecified dilution) followed by a chlorine-based disinfectant (sodium hypochlorite, Antisapril 2%; Angelini, active chlorine 540 mg/L). Baseline samples were taken immediately prior to disinfection with the new or two-step protocol. Measurements were taken at 0.5, 2.5, 4.5, and 6 hours after disinfection. Wet contact time not specified.	Average concentration ± standard deviation (CFU/25 cm^2) decreased significantly (71.2%, p=0.005) from 52 ± 63 prior to disinfection with alcohol-free QAC to 15 ± 24 after 0.5 hours. The two-step protocol with hypochlorite did not have a significant reduction (38.2%, p=0.32) compared to baseline (pre-protocol) measurements. Average concentration at 2.5, 4.5, and 6.5 hours after QAC disinfection were 20, 17, and 13 CFUs/25 cm^2, respectively (significance not specified).
Casini 2017 (129)	All viable organisms	Single-site, Quasi-experimental, uncontrolled before-after study over 6 months	206 samples were taken from high-touch surfaces (mobile and office telephones, tablets, keyboards and mice, touchscreen monitors, bed rails, and patient tables) surrounding patients in a 7-bed regional burns center at a tertiary-care teaching hospital. Pisa, Italy.	Standard cleaning with chlorine sodium hypochlorite (1400 mg/L available chlorine) compared to enhanced cleaning with addition of twice-daily wiping all high-touch surfaces with 0.5% chlorhexidine-60% isopropyl alcohol solution. Samples were taken from every cleaned surface one a week in late morning after cleaning	Initial environmental monitoring found number (percent) of surfaces with unacceptable (> 20 CFU/100 cm2) bacterial count at 13/103 (12.6%). During standard hypochlorite, 3/23 (13%) surfaces were unacceptable. During improved cleaning with addition of CHG, 2/50 (4%) surfaces were unacceptable. Also, during improved cleaning 8/30 (27%) surfaces were unacceptable possibly due to low adherence to protocol. No significance reported.
Casini 2019 (130)	All viable organisms	Single-site, Quasi-experimental uncontrolled before-after	A total of 345 samples collected from five specific high-touch surfaces (table, infusion	Standard cleaning with chlorine-based detergent (sodium hypochlorite, Antisapril Detergent 10%, Angelini) followed by 2800 mg/L active chlorine chlorine-based disinfectant (Antisapril Disinfectant 10%, Angelini). Disinfectant conc.	There was a decrease in mean (standard deviation) bacterial count with reductions of 86% (58±54 to 8±13 CFUs/24cm2) in patient rooms, 92% (25±19 to 2±4

Reference (Study ID)	Outcome	Study Design	Setting	Intervention	Results
		study , over 4 months	pump, tray table, patient be, tray table, call button, etc.) in sixteen critical areas including 5 patient rooms, 2 ICUs, and 9 operating theaters at a 1158-bed teaching hospital. Genoa, Italy.	raised to 18% in patient rooms with *C. difficile* infection. Unspecified conc. for detergent and unspecified contact times. Samples measured after patient discharge or surgical activity (baseline) and after standard disinfection.	CFUs/24cm2) in ICUs, and 86% (7±12 to 1±1 CFUs/24cm2) in high-turnover operating rooms following standard cleaning. Only the low-turnover OR samples experienced an average increase following standard cleaning from 6±10 to 11±18 CFUs/24cm2. Significance not specified.
Casini 2018 (131)	All viable organisms	Single-site, Quasi-experimental study, uncontrolled before-after, over 7 months	560 surface samples (unspecified) collected from two high patient turnover units-an ambulatory care visit unit and a wound care unit-of a hospital. Tuscany, Italy.	All samples collected one hour before and one hour after the disinfection. Disinfection included alcohol-based detergent pre-cleaning followed by a 540 mg/L chlorine-based disinfectant (sodium hypochlorite, 2% Antisapril, Angelini) properly diluted and sprayed. Unspecified contact times. Samplings performed every three days throughout study period.	There was a reduction in mean bacterial count from 995 (1494) to 462 (486) in ambulatory ward and from 404 (474) to 284 (336) in wound care ward corresponding to 59.4% and 38.5% reduction, respectively. Additionally, percent decrease in ATP bioluminescence was 59.4% in the ambulatory care unit and 38.5% in the wound care unit after disinfection. No significance reported.
Coppin 2017 (280)	All viable organisms	Single-site, quasi-experimental, controlled cohort study design with simultaneous control over a 2 day period	132 samples were taken from bedside tables 22 patient rooms (half isolation, half non-isolation rooms) at a120- bed veterans affairs hospital. Texas, USA.	Copper oxide (SSSCu) impregnated bedside tray tables (EOS^{Cu} Surfaces LLC) and non-copper tray tables were placed in 11 occupied patient rooms. All tables were cleaned with 10% sodium hypochlorite wipes (Clorox Healthcare) immediately prior to sampling. Samples were collected three times per day 0, 3, 6, 24, 27, and 30 h after cleaning.	There was no statistically significant difference in bacterial count between the copper and the non-copper surfaces at hours 0, 3, and 6. However, at hours 24, 27, and 30, there was a statistically higher concentration in non-copper sites compared to copper sites ($p = 0.002$). At hour 0, mean count (95% CI) was lower at 0.2 CFU/25 cm^2 (0-0.4) on copper surfaces compared to 1.9 CFU/25 cm^2 (0 – 4.9) on non-copper surfaces. At hour 30, mean count (95% CI) was lower at 18.9 CFU/25 cm^2 (11.0 – 32.5) on copper surfaces compared

Reference (Study ID)	Outcome	Study Design	Setting	Intervention	Results
					to 98.2 CFU/25 cm^2 (56.2-176.3) on non-copper surfaces.
Galván Contreras 2016 (1079)	All viable organisms	Single-site, Quasi-experimental study, controlled before-after study	84 samples from 21 surfaces (floors, walls, and ceilings) were sampled across adult ICU, neonatal ICU, emergency department, surgical unit, operating room of a hospital environment. Mexico City, Mexico.	6% sodium hypochlorite solution was compared to bromo-chloro-dimethyl-hydantoin (BCDMH) disinfectant (BCDMH Sanitizing Solution, GV-GERM) (diluted to 1-part sanitizer 3 parts water) applied through either sprayer or directly with flannel material. Sodium hypochlorite was 200 ppm in non-critical areas, 500 ppm in semi-critical areas and 5,000 ppm in critical areas. Unspecified contact times. Samples measured before disinfection interventions and 15 minutes after.	Percent surfaces positive was 13/ 21 surfaces before compared to 0/21 surfaces after BCDMH. Percent surfaces positive was 9/21 surfaces before disinfection and 2/21 surfaces after hypochlorite disinfection. There was no difference in disinfectant type (p=0.4). Reductions before compared to after were not significantly lower (p=0.15).
Huang 2015 (1311)	All viable organisms	Single site, uncontrolled before-after study, over 8 months	A total of 85 samples; 10-12 samples per room were collected from high-touch surfaces (door knob, light switch, windowsill, bedside rails, bedside cabinets, couch, toilet seats, hand rails, refrigerator, kettle, closet handles) from 8 rooms in the medical, surgical, and MICU wards at 2,200-bed tertiary care center. Scotland, UK.	Terminal cleaning consisted of 600 ppm sodium hypochlorite (contact time unspecified, pre-cleaning unspecified). Samples were collected ten minutes before and after terminal cleaning.	Before terminal cleaning, 20% of surfaces had aerobic colony counts >2.5 CFU/cm2 and 18.8% of surfaces had no growth compared to 5.9% of surfaces after terminal cleaning > 2.5 CFU/cm2 and 68.2% with no growth. Median (range) count was 0.25 (0.05-2.21) CFU/cm2 before terminal cleaning compared to 0 (0-0.5) CFU/cm2 after. There was a significant reduction after cleaning (p<0.001) compared to before for both colony count and ATP. The majority of isolated bacteria were coagulase negative staphylococci and gram-positive and gram-negative bacilli
Jinadatha 2014 (1416)	All viable organisms	Single site, controlled before-after study over two months	100 samples were taken from 5 high-touch surfaces (bedrail, toilet seat, bathroom handrail, call button, tray	Standard manual cleaning consisted of soak and wipe with 10% bleach solution (Dispatch®, contact time 1 min) with cotton rags. In the control group, standard manual cleaning was applied to visibly soiled and unsoiled areas of 20 room. In the intervention group, standard	Before cleaning, initial mean (median) concentration was 255 (278) CFU for control compared to 449 (365) in intervention. After cleaning there was a 76% reduction in control group

Reference (Study ID)	Outcome	Study Design	Setting	Intervention	Results
			table) in 40 rooms at 120-bed acute care veterans hospital in Texas, USA	manual cleaning was applied only to visibly soiled areas and was followed by PPX-UV device in 20 rooms. Samples were collected before cleaning and after standard cleaning in the control group and after portable pulsed ultraviolet (PPX-UV) in the intervention period.	(mean=60 CFU) and 98% reduction in intervention group (mean= 8 CFU). Significance was not assessed before compared to after. While controlling for significant differences in initial concentration, the intervention had significantly lower concentration of bacteria compared to control group ($p<0.01$).
Mosci 2017 (1886)	All viable organisms	Multi-site, Controlled before-after cohort study over 9 months	448 samples were collected from 28 rooms (medicine, orthopedics, long-term care, recovery and functional rehabilitation) 4 public and private health facilities in Emilia-Romaga Region, Italy	Rooms randomized to terminal cleaning with 0.5% sodium hypochlorite or with HPV (99MS system, < 8% hydrogen peroxide concentration and silver ions, 130-minute cycle time). All rooms received standard cleaning to remove visible dirt prior to disinfection intervention. Samples were taken before and after intervention.	Number of rooms positive for bacteria significantly decreased from 7 (50%) to 0 (0%) with HPV and from 11 (79%) to 2 (14%) with hypochlorite. Number of samples positive decreased from 13/112 (13%) to 0/112 (0%) with HPV vs. 22/112 (20%) to 3/112 (3%) with hypochlorite. Methods were similar ($p=0.497$).
Patel 2007 (2323)	All viable organisms	Single site, uncontrolled before-after study over 18 weeks	567 samples from frequent hand touch sites (armchair arm, bedside table, locker top, zimmer frame, door handle, bed frame, wall-mounted patient drug box, overhead lamp) in two isolation rooms at a 300-bed district general hospital in southern England, UK.	Initial 6-week period had routine cleaning practices with detergent alone. In the second 6-week period, the intervention added sodium hypochlorite Unichem, 1,000 ppm) after routine cleaning. This study also reports on an enhanced training intervention. Samples were collected twice a week from each site within 3 h of cleaning.	Detergent followed by sodium hypochlorite had significantly lower total counts than when cleaning with detergent alone on 10 of 16 surface types
Rathod 2019 (3507)	All viable organisms	Single site, controlled	150 samples from five high-touch surfaces (bed rails,	15 rooms cleaned with bleach wipe and 15 rooms cleaned with bleach and a liquid color additive (Highlight ®) to improve surface	Bleach alone resulted in 98% reduction of bacteria after disinfection. Bleach with the

Reference (Study ID)	Outcome	Study Design	Setting	Intervention	Results
		before-after study over	tray tables, room light switches, bathroom inner door knobs, and toilet seats) in 30 patient rooms in a medical oncology/hematology -oncology/medical ICU at Connecticut, USA	coverage. Samples were collected pre- and post-cleaning (time relative to disinfection unspecified).	additive resulted in 58% reduction of bacteria after disinfection (concentration data not reported).
Zhang 2013 (3666)	All viable organisms	Single site, uncontrolled before-after study	60 surfaces (bedside tables, bed rails, call buttons, telephones, trash can lids) from 12 medical and surgical ward rooms at a veterans affairs hospital in Ohio, USA	Disinfection consisted of 10% bleach (contact time 10 min) on visibly soiled and clean surfaces. Samples were collected before and after disinfection, but before terminal cleaning.	There was a significant decrease in the mean (standard deviation) of total bacterial count recovered after hypochlorite disinfection from 160 (50) to 0.8 (20) ($p<0.001$) for both visibly cleaned and stained surfaces. Among only visibly clean surfaces, there was a significant reduction in mean (standard deviation) bacterial concentration from 140 (20) to 0.03 (1) after disinfection ($p<0.001$).
Ho 2016 (6163)	All viable organisms	Single-site, Quasi-experimental study, controlled before-after study	121 total samples were collected from 11 specific high-touch surfaces in 22 rooms of the hospital environment. Hualien, Taiwan.	Standard daily cleaning (before) with 0.06% sodium hypochlorite detergent disinfectant on microfiber cloths compared to modified daily cleaning (after) with demand-release chlorine sodium dichloroisocyanurate (NaDCC) tablets (Medentech, Wexford, Ireland) on microfiber cloths (concentration of active chlorine equivalent to that of 0.05% NaOCl). Unspecified contact time. Samples measured “before and after daily cleaning” for each intervention	Median aerobic colony counts before disinfection with sodium hypochlorite ranged from 0.8 – 51.9 CFU/cm2 compared to after sodium hypochlorite disinfection ranging from 0.1 – 23.9 CFU/cm2. Median count before disinfection with NaDCC ranged from 0.5 – 28.6 CFU/cm2 before compared to 0.0 – 9.2 CFU/cm2 after disinfection. 3 of 11 surface types had significantly lower concentration after disinfection with NaDCC compared to 2 of 11 surfaces types with significantly

Reference (Study ID)	Outcome	Study Design	Setting	Intervention	Results
					lower concentration after disinfection with sodium hypochlorite.
Alhmidi 2017 (6931)	All viable organisms	Single-site, Quasi-experimental, controlled before-after study	471 samples were collected from 100 hard surfaces (bed rails, bedside tables, and physical therapy hand rails) and 57 soft surfaces (chairs, mattresses, and cushions) in hospital wards at veterans affair medical center. Ohio, USA	Control samples were taken 30 s after a surface was sprayed 5 times with sterile water compared to two disinfectants. Experimental samples were taken 30 s after surface was sprayed 5 times with 30% ethanol spray (Purell Healthcare Surface Disinfectant) or after surface was sprayed 5 times with 0.65% sodium hypochlorite spray (Clorox Healthcare Bleach Germicidal Cleaner).	Percent surfaces positive was significantly lower ($p < 0.01$) for surfaces sprayed with Purell at 2.5% and Clorox at 1.3% compared to control surfaces at 16.6%. There was not a significant difference between the two disinfectants.
Simon Garcia 2009 (7960)	All viable organisms	Single site, uncontrolled before-after study over 2 years	290 samples from surfaces (furniture, clinical equipment) in two ICUs at a hospital in Madrid, Spain.	A one-year pre-intervention period and one-year post-intervention period were compared. Samples were collected before and after disinfection intervention to control an outbreak. The disinfection consisted of QAC disinfection on furniture and equipment and 5% bleach solution on floors and walls.	During the pre-intervention period 29% samples (41/144) had pathogenic organisms and 4% (5/144) had bacterial counts > 100 CFU. During the post-intervention period, fewer samples were positive for pathogenic flora at 5% (7/146) and none (0/146) had bacterial counts > 100 CFU. Significance not specified.
Rutala 2018 (10553)	All viable organisms (MDRO- MDR *Acinetobacter*, MRSA, VRE, *C. difficile*)	Multi-site, controlled cohort study over 27-month period	7,360 samples from environmental surfaces (bed rail, over-bed table, supply or medicine cart, chair sink, toilet seat, shower floor, side counter, linen hamper lid, bathroom floor) in 92 rooms of 3 university-affiliated hospitals in North Carolina, USA	Compared standard cleaning with quaternary ammonium compound disinfection in 21 randomly selected rooms with standard cleaning with bleach in 20 randomly selected rooms. Active ingredients and contact time were not specified. Samples were collected after disinfection (not specified).	Average concentration of pathogen was 60.8 CFU per room using quaternary ammonium compounds compared to 11.7 CFU per room when using bleach (81% reduction).

Reference (Study ID)	Outcome	Study Design	Setting	Intervention	Results
Lerner 2019 (1574)	Gram-negative bacteria (*Acinetobacter baumannii*-CRAB)	Single-site, quasi-experimental, controlled before-after study for a 6-month period	253 samples from environmental objects (bedrail, IV pole, bed linen, electrical outlet, infusion bottle hook, medical tray, medical trolley, arm chair, chair, curtain, doorknob, counter, cupboard shelf, monitor, IV pump, ventilator, stethoscope, hemodialysis machine, mattress pump, warming unit) in 7 single-patient rooms of known CRAB-carriers in the MICU at 1450-bed tertiary-care hospital. Tel Aviv, Israel.	Terminal cleaning was with manually-applied sodium hypochlorite in 3 rooms (concentration, contact time unspecified) compared to aerosolized hydrogen peroxide (aHP) in 4 rooms (GLOSAIRTM 400, unspecified concentration, cycle time). Samples were collected before and immediately after terminal disinfection.	Before disinfection, 41% (24/59) were positive compared to 6% (3/52) after disinfection with manual sodium hypochlorite, an 85% reduction. Before disinfection with aHP, 80% (59/74) samples were positive compared to 18% (12/68) after disinfection, a 78% reduction. Significance not specified.
Ho 2016 (6163)	Gram-negative bacteria (*Acinetobacter baumannii*-CRAB)	Single-site, Quasi-experimental study, controlled before-after study	121 total samples were collected from 11 specific high-touch surfaces in 22 rooms of the hospital environment. Hualien, Taiwan.	Standard daily cleaning (before) with 0.06% sodium hypochlorite detergent disinfectant on microfiber cloths compared to modified daily cleaning (after) with demand-release chlorine sodium dichloroisocyanurate (NaDCC) tablets (Medentech, Wexford, Ireland) on microfiber cloths (concentration of active chlorine equivalent to that of 0.05% NaOCl). Unspecified contact time. Samples measured “before and after daily cleaning” for each intervention	Median (range) aerobic colony count in CFU/cm2 of CRAB across surfaces was 8.5 (0.0-16.1 range) before NaDCC disinfection compared to 0.5 (0.0 -0.6) after disinfection (~94% decrease, $p<0.01$.). Median (range) was 101.7 (0.0 – 201.8) before disinfection with sodium hypochlorite compared to not detectable after disinfection ($p<0.01$).
Manian 2011 (14130)	Gram-negative bacteria (*Acinetobacter*	Single site, uncontrolled before-after	7140 samples (bedside table, chair, TV, door, sink,	Routine terminal cleaning and disinfection by rooms vacated by antibiotic-resistant ABC-positive patients consisted of disinfection with	After 4 rounds of bleach disinfection, 27% rooms (83/312) and 16% (51/5705) sites were

Reference (Study ID)	Outcome	Study Design	Setting	Intervention	Results
	baumannii complex (ABC))	study, over 5 years	bedrail, telephone, lift, cabinet, countertop, etc.) in 384 rooms from all wards at suburban 900-bed community teaching medical center. Missouri, USA.	QAC followed by 0.525% sodium hypochlorite solution. During room occupation, at least daily disinfection was conducted with sodium hypochlorite. HPV (Bioquell) treatment was conducted following newly-vacated room following terminal disinfection with bleach.	positive for ABC. After 1 round of bleach disinfection, there was a significant reduction in number of sites positive (n=700) for ABC (OR=0.25, 95% CI: 0.045-0.93, p=0.04). After 1 round of bleach disinfection and addition of HPV, there was a near-significant reduction in ABC-positive sites (odds ratio=0, 95% CI: 0-0.08, p=0.04).
Rutala 2018 (10553)	Gram-negative bacteria (*Acinetobacter* spp.-MDR)	Multi-site, controlled cohort study over 27-month period	7,360 samples from environmental surfaces (bed rail, over-bed table, supply or medicine cart, chair sink, toilet seat, shower floor, side counter, linen hamper lid, bathroom floor) in 92 rooms of 3 university-affiliated hospitals in North Carolina, USA	Compared standard cleaning with quaternary ammonium compound disinfection in 21 randomly selected rooms with standard cleaning with bleach in 20 randomly selected rooms. Active ingredients and contact time were not specified. Samples were collected after disinfection (not specified).	Mean concentration per room for MDR *Acinetobacter* was significantly higher (p=0.035) using QAC compared to bleach with 8.95 compared to 0.39 CFU.
Roux 2013 (3147)	Gram-negative bacteria (*Enterobacteriaceae*-Extended-spectrum beta-lactamase-producing *Enterobacteriaceae* (ESBLE))	Multi-site, cross-sectional cohort study, 4 weeks	185 samples (handwashing sink drains) from 13 ICUs of 7 hospitals and 1 surgical clinic in Tours France.	Routine disinfection was reported for daily disinfection with bleach compared to daily disinfection with quaternary ammonium compounds. Products, active ingredients, contact times not specified. Volume of disinfecting product varied from 25mL of pure product to several liters of variously diluted solutions.	The number of sinks positive for ESBL *Enterobacteriaceae* was significantly higher (p=0.002) when using QAC (20/56) compared to using bleach (0/19).
Alhmidi 2017 (6931)	Gram-negative bacteria (Gram-negative bacilli)	Single-site, Quasi-experimental, controlled	471 samples were collected from 100 hard surfaces (bed rails, beside tables, and physical therapy	Control samples were taken 30 s after a surface was sprayed 5 times with sterile water compared to two disinfectants. Experimental samples were taken 30 s after surface was sprayed 5 times with 30% ethanol spray (Purell Healthcare	Percent surfaces positive was lower (p=0.07) for surfaces sprayed with Purell at 0.6% and Clorox at 0.6% compared to control surfaces at 4.5%. There

Reference (Study ID)	Outcome	Study Design	Setting	Intervention	Results
		before-after study	hand rails) and 57 soft surfaces (chairs, mattresses, and cushions) in hospital wards at veterans affair medical center. Ohio, USA	Surface Disinfectant) or after surface was sprayed 5 times with 0.65% sodium hypochlorite spray (Clorox Healthcare Bleach Germicidal Cleaner).	was not a significant difference between the two disinfectants.
Casini 2019 (130)	Gram-negative bacteria (*K. pneumoniae*)	Single-site, Quasi-experimental uncontrolled before-after study , over 4 months	A total of 345 samples collected from five specific high-touch surfaces (table, infusion pump, tray table, patient be, tray table, call button, etc.) in sixteen critical areas including 5 patient rooms, 2 ICUs, and 9 operating theaters at a 1158-bed teaching hospital. Genoa, Italy.	Standard cleaning with chlorine-based detergent (sodium hypochlorite, Antisapril Detergent 10%, Angelini) followed by 2800 mg/L active chlorine chlorine-based disinfectant (Antisapril Disinfectant 10%, Angelini). Disinfectant conc. raised to 18% in patient rooms with *C. difficile* infection. Unspecified conc. for detergent and unspecified contact times. Samples measured after patient discharge or surgical activity (baseline) and after standard disinfection.	The number of surfaces with ESBL-*K. pneumoniae* in a room where a patient with ESBL-*K. pneumoniae* was admitted was reduced from 3 out of 5 surfaces (60%) to 1 out of 5 surfaces (20%) following standard cleaning (a 66.67% decrease). The number of surfaces with KPC-producing *K. pneumoniae* in a room where a patient with KPC-producing *K. pneumoniae* patient was admitted was reduced from 1 out of 5 surfaces (20%) to 0 out of 5 surfaces (0%) following standard cleaning.
Styaningsih 2019 (2926)	Gram-positive bacilli (*Bacillus subtilis*)	Single-site, quasi-experimental, controlled before-after study.	36 samples were collected from the floor of operating room of surgical unit at a hospital. Kudus City, Indonesia.	The efficacy of two disinfectants was compared: quaternary ammonium derivative (unspecified product or concentration) or sodium hypochlorite (unspecified product or concentration). Sampling was conducted prior to disinfection, after 20 minutes, and after 2 h from disinfection. Efficacy was compared between surgical rooms with centralized or split AC systems.	Average Bacillus spp before disinfection was 4.00 CFU/cm2 in split AC for sodium hypochlorite and 2.33CFU/cm2 in split AC for QAC. Samples were not positive after disinfection with QAC or sodium hypochlorite (20 min or 2 h). Central AC had lower mean bacterial count.
Casini 2019 (130)	Gram-positive bacilli (*Clostridium difficile* spores)	Single-site, Quasi-experimental uncontrolled before-after	A total of 345 samples collected from five specific high-touch surfaces (table, infusion pump, tray table,	Standard cleaning with chlorine-based detergent (sodium hypochlorite, Antisapril Detergent 10%, Angelini) followed by 2800 mg/L active chlorine chlorine-based disinfectant (Antisapril Disinfectant 10%, Angelini). Disinfectant conc. raised to 18% in patient rooms with *C. difficile*	The number of surfaces with *C. difficile* in a room where a *C. difficile* patient was admitted reduced from 4 out of 5 surfaces (80%) to 1 out of 5 surfaces (20%)

Reference (Study ID)	Outcome	Study Design	Setting	Intervention	Results
		study , over 4 months	patient be, tray table, call button, etc.) in sixteen critical areas including 5 patient rooms, 2 ICUs, and 9 operating theaters at a 1158-bed teaching hospital. Genoa, Italy.	infection. Unspecified conc. for detergent and unspecified contact times. Samples measured after patient discharge or surgical activity (baseline) and after standard disinfection.	following standard cleaning (a 75% decrease).
Barbut 2009 (686)	Gram-positive bacilli (*Clostridium difficile*)	Multisite, Quasi-experimental, controlled cohort study over 5 months	748 total samples collected from 12 high-touch surfaces (bathroom floor, bedside table, care table, door handle, windowsill, etc.) from 31 rooms following patient with CDI discharge at 2 university hospitals. Creteil, France	Terminal cleaning in rooms following discharge of patient with *C. difficile* infection. Patient randomized to either HPV (hydrogen peroxide, phosphoric acid < 50ppm, silver cations < 50ppm, gum Arabic <1 ppm, 95% biosmotic water; Sterinis-Sterusil), 1-hour contact time, or control group with sodium hypochlorite solution (0.5%, 5,000 ppm available chlorine). Before each, floors and surfaces cleaned with detergent and rinsed with water. Samples collected before cleaning and after hypochlorite dried or 1 h exposure for HPV.	% positive surfaces and rooms were significantly lower after compared to before disinfection. Before cleaning, *C. difficile* spores were detected in 21% (80/374) samples and 74% (23/31) rooms. After hypochlorite, 12% (23/194) samples were positive ($p<0.002$) and 2% (4/180) surfaces were positive after HPV ($p<0.001$). Percent reduction of *C. difficile* positive samples was higher at 91% in HPV group vs 50% in hypochlorite group ($p<0.005$)
Eckstein 2007 (899)	Gram-positive bacilli (*Clostridium difficile*)	Single-site, quasi-experimental, uncontrolled study over a 6-week period	A total of 102 microbiological samples were collected from bedrail, bedside table, phone, call button, toilet, and door handle in 26 rooms of patients with either *C. difficile* or VRE colonization in an acute care Veterans Affairs Medical Center in Ohio, USA	Baseline measurements were taken within 3 days of patient discharge. Terminal disinfection of rooms with CDAD patients was a 10% bleach solution. Researchers implemented an additional disinfection with 10% bleach prior to admission of another patient. Application methods were cloth or mop and disinfectants were allowed to air dry. Dilutions, time until measurement unspecified.	Percent of room surface positive for *C. difficile* was 100% (9/9) prior to disinfection, lower ($p=0.50$) after the first round of bleach disinfection at 78% (7/9) and significantly lower after two rounds of bleach disinfection ($p=0.031$) at 11% (1/9). Total samples positive prior to disinfection was 56% (30/54) compared to after one round of bleach at 44% (24/54).

Reference (Study ID)	Outcome	Study Design	Setting	Intervention	Results
Ghantoji 2015 (1171)	Gram-positive bacilli (*Clostridium difficile*)	Single site, controlled before-after study over	298 samples from five high-touch surfaces (bathroom handrail, horizontal surface, bed control panel, bedrail, bedside table, IV pump control panel) in 30 rooms previously occupied by *C. difficile*-infected patients at a comprehensive cancer center in Texas, USA.	15 rooms were cleaned with standard protocol (10% sodium hypochlorite, unspecified contact time or product). 15 rooms were visually cleaned without bleach followed by PX-UV (5 min). Samples were collected after patient discharge but before and after disinfection with 10% sodium hypochlorite and before and after PX-UV treatment.	Bleach reduced mean count by 70% from 2.39 CFU before disinfection to 0.71 CFU after (p=0.14). UV reduced mean count from 22.97 CFU before disinfection to 1.19 CFU after disinfection (p=0.0017). PX-UV disinfection was not significantly better than bleach alone.
Mosci 2017 (1886)	Gram-positive bacilli (*Clostridium difficile*)	Multi-site, Controlled before-after cohort study over 9 months	448 samples were collected from 28 rooms (medicine, orthopedics, long-term care, recovery and functional rehabilitation) 4 public and private health facilities in Emilia-Romaga Region, Italy	Rooms randomized to terminal cleaning with 0.5% sodium hypochlorite or with HPV (99MS system, < 8% hydrogen peroxide concentration and silver ions, 130-minute cycle time). All rooms received standard cleaning to remove visible dirt prior to disinfection intervention. Samples were taken before and after intervention.	Percent of samples contaminated with *C. difficile* significantly decreased from 13% to 0% in HPV disinfection (p=0.002) and from 20% to 3% with sodium hypochlorite (p=0.006). Methods were similar (p=0.267).
Wilcox 2003 (3854)	Gram-positive bacilli (*Clostridium difficile*)	Single site, crossover study over 2 years	1128 environmental samples (floor, radiator, bedframe, toilet floor, sluice floor, cleaner floor, commode, side room floors, side room curtain rails) at two elderly medicine wards in Leeds, UK	Two similar wards were cleaned with one or the other cleaning regiment for 6-12 month periods with either neutral liquid detergent (Hospec) or detergent followed by 1000 ppm hypochlorite (Saniclor, 12.5% sodium hypochlorite). Environmental samples were collected as part of surveillance. Sample collection relative to disinfection time was not reported.	The percent surfaces positive for *C. difficile* in wards using detergent was 35.4% and 37.7%. The percent of surfaces positive for *C. difficile* in wards using sodium hypochlorite was 26.4% and 37.3%. Significance was not assessed comparing percent surfaces positive. Commodes, toilet floors, and bed frames had high prevalence of surfaces positive.

Reference (Study ID)	Outcome	Study Design	Setting	Intervention	Results
Deshpande 2014 (7047)	Gram-positive bacilli (*Clostridium difficile*)	Single-site, quasi-experimental, controlled cohort study over a 1-month period	A total of 888 samples were taken from the floors and high-touch surfaces (bed rails, bedside tables) of an unspecified number of CDI and MRSA isolation rooms in a hospital in the USA	Baseline measurements were not specified. Routine cleaning (control) with alcohol-free QAC (<1% n-alkyl dimethyl benzyl ammonium chloride and <1% n-alkyl dimethyl ethylbenzyl ammonium chloride; Virex; Diversey) was mopped on half of floors, and sodium hypochlorite (1:10 dilution of household bleach) was wiped onto high-touch surfaces. The product was compared to enhanced (trial) cleaning with peracetic acid (0.13%) and hydrogen peroxide (0.63%) based sporicidal product (Oxycide; Ecolab), applied in the same fashion to the second half of floors and high-touch surfaces. All surfaces were allowed to air-dry 10-15 min. Dilutions and time until measurement were not specified.	OxyCide and bleach significantly reduced (p<0.05) the recovery of *C. difficile* while the QAC did not significantly reduce recovery of *C. difficile* (p>0.05). In bedside tables and bed rails, there was no recovery of *C. difficile* after OxyCide or bleach disinfection (from 8/50 to 0/50 surfaces positive for OxyCide and from 7/50 to 0/50 surfaces positive for bleach). On floors, there was 5% surfaces positive after disinfection compared to 50% before disinfection with OxyCide (from 41/82 to 4/82 surfaces positive) compared to 54% surfaces positive after QAC and 55% positive before QAC disinfection (from 45/82 to 44/82 surfaces positive).
Rutala 2018 (10553)	Gram-positive bacilli (*Clostridium difficile*)	Multi-site, controlled cohort study over 27-month period	7,360 samples from environmental surfaces (bed rail, over-bed table, supply or medicine cart, chair sink, toilet seat, shower floor, side counter, linen hamper lid, bathroom floor) in 92 rooms of 3 university-affiliated hospitals in North Carolina, USA	Compared standard cleaning with quaternary ammonium compound disinfection in 21 randomly selected rooms with standard cleaning with bleach in 20 randomly selected rooms. Active ingredients and contact time were not specified. Samples were collected after disinfection (not specified).	Mean concentration per room for *C. difficile* was not different using QAC compared to bleach at 3.76 compared to 4.48 CFU (p>0.05).
Kaatz 1988 (14394)	Gram-positive bacilli (*Clostridium difficile*)	Single site, uncontrolled before-after study	1085 environmental samples (floors, walls, windows, bathrooms, bed frames, doors) in a	Outbreak ward was disinfected by spray-application with 500 ppm unbuffered sodium hypochlorite. Some rooms were also disinfected with buffered solutions (1600 ppm available	Significant reduction in percent surfaces positive and mean count of *C. difficile* resulting in a 79% reduction in CFU after disinfection (from 31% (81/258) to 17%

Reference (Study ID)	Outcome	Study Design	Setting	Intervention	Results
			medical ward at a hospital in Michigan, USA	chlorine). Samples were collected before and 24 h after disinfection.	(40/243) surfaces positive and from a mean of 5.1 CFU to 2.0 CFU after disinfection) (p<0.001). Using phsophate buffered hypochlorite, pre-disinfection had 14% (11/78) samples positive compared to 1% (1/78) positive after disinfection (p<0.001).
Eckstein 2007 (899)	Gram-positive cocci (*Enterococcus* spp.-Vancomycin-resistant enterococci (VRE))	Single-site, quasi-experimental, uncontrolled study over a 6-week period	A total of 102 microbiological samples were collected from bedrail, bedside table, phone, call button, toilet, and door handle in 26 rooms of patients with either *C. difficile* or VRE colonization in an acute care Veterans Affairs Medical Center in Ohio, USA	Baseline measurements were taken within 3 days of patient discharge. Terminal disinfection of rooms (control) using low-alcohol QAC (7-13% didecyldimethyl ammonium chloride, 3-7% alkyl dimethylbenzyl ammonium chloride, 1-5% alcohol, and 1-5% tetrasodium EDTA; Super HDQ Neutral; Spartan) was compared to additional effect of cleaning procedure using 10% bleach solution. Researchers implemented the 10% bleach prior to admission of another patient. Application methods were cloth or mop and disinfectants were allowed to air dry. Dilutions, time until measurement unspecified.	Number (%) rooms positive for environmental cultures was 16/17 (94%) before routine cleaning with low-alcohol QAC versus 12/17 (71%) after (p=0.125). 72 of 102 total samples (71%) were positive for VRE before cleaning with low-alcohol QAC compared to 58 of 102 (57%) were positive after (significance not specified). Additional cleaning with bleach significantly reduced % surfaces positive to 0 (p<0.001).
Rutala 2018 (10553)	Gram-positive cocci (*Enterococcus* spp.-Vancomycin-resistant enterococci (VRE))	Multi-site, controlled cohort study over 27-month period	7,360 samples from environmental surfaces (bed rail, over-bed table, supply or medicine cart, chair sink, toilet seat, shower floor, side counter, linen hamper lid, bathroom floor) in 92 rooms of 3 university-affiliated hospitals in North Carolina, USA	Compared standard cleaning with quaternary ammonium compound disinfection in 21 randomly selected rooms with standard cleaning with bleach in 20 randomly selected rooms. Active ingredients and contact time were not specified. Samples were collected after disinfection (not specified).	Mean concentration per room for VRE was higher (p>0.05) using QAC compared to bleach with 39.57 compared to 2.43 CFU.
Ho 2016 (6163)	Gram-positive cocci	Single-site, Quasi-experimental	121 total samples were collected from 11 specific high-	Standard daily cleaning (before) with 0.06% sodium hypochlorite detergent disinfectant on microfiber cloths compared to modified daily	Median (range) aerobic colony count in CFU/cm2 of VRE across surfaces was reduced from 1.5 (0.0

Reference (Study ID)	Outcome	Study Design	Setting	Intervention	Results
	(*Enterococcus* spp.-VRE)	study, controlled before-after study	touch surfaces in 22 rooms of the hospital environment. Hualien, Taiwan.	cleaning (after) with demand-release chlorine sodium dichloroisocyanurate (NaDCC) tablets (Medentech, Wexford, Ireland) on microfiber cloths (concentration of active chlorine equivalent to that of 0.05% NaOCl). Unspecified contact time. Samples measured "before and after daily cleaning" for each intervention	-8.4) before disinfection with NaDCC to undetectable level after disinfection. Median (range) was 0.98 (0.0 – 32.1) before disinfection with sodium hypochlorite compared to 0.0 (0.0 – 0.1) after disinfection.
Alhmidi 2017 (6931)	Gram-positive cocci (*Enterococcus* spp.-VRE)	Single-site, Quasi-experimental, controlled before-after study	471 samples were collected from 100 hard surfaces (bed rails, beside tables, and physical therapy hand rails) and 57 soft surfaces (chairs, mattresses, and cushions) in hospital wards at veterans affair medical center. Ohio, USA	Control samples were taken 30 s after a surface was sprayed 5 times with sterile water compared to two disinfectants. Experimental samples were taken 30 s after surface was sprayed 5 times with 30% ethanol spray (Purell Healthcare Surface Disinfectant) or after surface was sprayed 5 times with 0.65% sodium hypochlorite spray (Clorox Healthcare Bleach Germicidal Cleaner).	Percent surfaces positive for VRE was lower (p=0.07) for surfaces sprayed with Purell at 0.6% and Clorox at 0.6% compared to control surfaces at 4.5%. There was not a significant difference between the two disinfectants.
Deshpande 2014 (7047)	Gram-positive cocci (Gram positive organisms- *Staphylococcus aureus* (MRSA) or *Enterococcus* spp (VRE))	Single-site, quasi-experimental, controlled cohort study over a 1-month period	A total of 888 samples were taken from the floors and high-touch surfaces (bed rails, bedside tables) of an unspecified number of CDI and MRSA isolation rooms in a hospital in the USA	Baseline measurements were not specified. Routine cleaning (control) with alcohol-free QAC (<1% n-alkyl dimethyl benzyl ammonium chloride and <1% n-alkyl dimethyl ethylbenzyl ammonium chloride; Virex; Diversey) was mopped on half of floors, and sodium hypochlorite (1:10 dilution of household bleach) was wiped onto high-touch surfaces. The product was compared to enhanced (trial) cleaning with peracetic acid (0.13%) and hydrogen peroxide (0.63%) based sporicidal product (Oxycide; Ecolab), applied in the same fashion to the second half of floors and high-touch surfaces. All surfaces were allowed to air-dry 10-15 min. Dilutions and time until measurement were not specified.	OxyCide and bleach significantly reduced (p<0.05) recovery of MRSA and/or VRE, but not QAC (p>0.05). In bedside tables and bed rails, there was no recovery of MRSA/VRE after OxyCide or bleach disinfection (from 11/50 to 0/50 surfaces positive for OxyCide and from 12/50 to 0/50 surfaces positive for bleach). On floors, there was no recovery after OxyCide disinfection compared to 18% positive before (from 7/40 to 0/40 surfaces positive) compared to 18% surfaces positive after QAC and 25% surfaces positive before from 10/40 to 7/40 surfaces positive).

Reference (Study ID)	Outcome	Study Design	Setting	Intervention	Results
Jinadatha 2014 (1416)	Gram-positive cocci (*Staphylococcus aureus*-MRSA)	Single site, controlled before-after study over two months	100 samples were taken from 5 high-touch surfaces (bedrail, toilet seat, bathroom handrail, call button, tray table) in 40 rooms at 120-bed acute care veterans hospital in Texas, USA	Standard manual cleaning consisted of soak and wipe with 10% bleach solution (Dispatch®, contact time 1 min) with cotton rags. In the control group, standard manual cleaning was applied to visibly soiled and unsoiled areas of the room. In the intervention group, standard manual cleaning was applied only to visibly soiled areas and was followed by PPX-UV device. Samples were collected before cleaning and after standard cleaning in the control group and after portable pulsed ultraviolet (PPX-UV) in the intervention period.	Before cleaning, initial mean (median) concentration was 127 (28.5) CFU for control compared to 108 (123) in intervention. After cleaning there was a 91% reduction in control group (mean=11 CFU) and 99% reduction in intervention group (mean= 1 CFU). Significance was not assessed before compared to after. While controlling for significant differences in initial concentration, the intervention had significantly lower concentration of bacteria compared to control group (p<0.03).
Patel 2007 (2323)	Gram-positive cocci (*Staphylococcus aureus*-MRSA)	Single site, uncontrolled before-after study over 18 weeks	567 samples from frequent hand touch sites (armchair arm, bedside table, locker top, zimmer frame, door handle, bed frame, wall-mounted patient drug box, overhead lamp) in two isolation rooms at a 300-bed district general hospital in southern England, UK.	Initial 6-week period had routine cleaning practices with detergent alone. In the second 6-week period, the intervention added sodium hypochlorite Unichem, 1,000 ppm) after routine cleaning. This study also reports on an enhanced training intervention. Samples were collected twice a week from each site within 3 h of cleaning.	Of the 16 surfaces, detergent followed by sodium hypochlorite had significantly lower number of sites positive for MRSA on 3 of the surfaces.
Ho 2016 (6163)	Gram-positive cocci (*Staphylococcus aureus*-MRSA)	Single-site, Quasi-experimental study, controlled before-after study	121 total samples were collected from 11 specific high-touch surfaces in 22 rooms of the hospital environment. Hualien, Taiwan.	Standard daily cleaning (before) with 0.06% sodium hypochlorite detergent disinfectant on microfiber cloths compared to modified daily cleaning (after) with demand-release chlorine sodium dichloroisocyanurate (NaDCC) tablets (Medentech, Wexford, Ireland) on microfiber cloths (concentration of active chlorine equivalent to that of 0.05% NaOCl). Unspecified	Median (range) aerobic colony count in CFU/cm2 of MRSA across surfaces was reduced from 0.9 (0.0-447.3 range) before disinfection with NaDCC to 0.0 (0.0-5.4) after disinfection. Median (range) was 1.1 (0.0 – 50.0) before disinfection with

Reference (Study ID)	Outcome	Study Design	Setting	Intervention	Results
				contact time. Samples measured "before and after daily cleaning" for each intervention	sodium hypochlorite) compared to 0.0 (0.0 – 6.2) after disinfection.
Alhmidi 2017 (6931)	Gram-positive cocci (*Staphylococcus aureus*-MRSA)	Single-site, Quasi-experimental, controlled before-after study	471 samples were collected from 100 hard surfaces (bed rails, beside tables, and physical therapy hand rails) and 57 soft surfaces (chairs, mattresses, and cushions) in hospital wards at veterans affair medical center. Ohio, USA	Control samples were taken 30 s after a surface was sprayed 5 times with sterile water compared to two disinfectants. Experimental samples were taken 30 s after surface was sprayed 5 times with 30% ethanol spray (Purell Healthcare Surface Disinfectant) or after surface was sprayed 5 times with 0.65% sodium hypochlorite spray (Clorox Healthcare Bleach Germicidal Cleaner).	Percent surfaces positive was significantly lower ($p < 0.01$) for surfaces sprayed with Purell at 1.3% and Clorox at 0.0% compared to control surfaces at 7.6%. There was not a significant difference between the two disinfectants.
Rutala 2018 (10553)	Gram-positive cocci (*Staphylococcus aureus*-MRSA)	Multi-site, controlled cohort study over 27-month period	7,360 samples from environmental surfaces (bed rail, over-bed table, supply or medicine cart, chair sink, toilet seat, shower floor, side counter, linen hamper lid, bathroom floor) in 92 rooms of 3 university-affiliated hospitals in North Carolina, USA	Compared standard cleaning with quaternary ammonium compound disinfection in 21 randomly selected rooms with standard cleaning with bleach in 20 randomly selected rooms. Active ingredients and contact time were not specified. Samples were collected after disinfection (not specified).	Mean count per room for MRSA was higher ($p>0.05$) using QAC compared to bleach at 8.52 CFU compared to 4.39 CFU.
Manian 2011 (14130)	Gram-positive cocci (*Staphylococcus aureus*-MRSA)	Single site, uncontrolled before-after study, over 5 years	7140 samples (bedside table, chair, TV, door, sink, bedrail, telephone, lift, cabinet, countertop, etc.) in 384 rooms from all wards at suburban 900-bed community teaching medical	Routine terminal cleaning and disinfection by rooms vacated by antibiotic-resistant ABC-positive patients consisted of disinfection with QAC followed by 0.525% sodium hypochlorite solution. During room occupation, at least daily disinfection was conducted with sodium hypochlorite. HPV (Bioquell) treatment was conducted following newly-vacated room following terminal disinfection with bleach.	After 4 rounds of bleach disinfection, 14% rooms (44/312) and 2% (108/5705) sites were positive for MRSA. After 1 round of bleach disinfection, there was not a significant reduction in number of sites positive ($p=0.45$. After 1 round of bleach disinfection and addition of HPV, there was a significant reduction in

Reference (Study ID)	Outcome	Study Design	Setting	Intervention	Results
			center. Missouri, USA.		MRSA-positive sites (odds ratio=0, 95% CI: 0-0.85, p=0.04)
Styaningsih 2019 (2926)	Gram-positive cocci (*Staphylococcus aureus*)	Single-site, quasi-experimental, controlled before-after study.	36 samples were collected from the floor of operating room of surgical unit at a hospital. Kudus City, Indonesia.	The efficacy of two disinfectants was compared: quaternary ammonium derivative (unspecified product or concentration) or sodium hypochlorite (unspecified product or concentration). Sampling was conducted prior to disinfection, after 20 minutes, and after 2 h from disinfection. Efficacy was compared between surgical rooms with centralized or split air conditioning (AC) systems.	Average S. *aureus* before disinfection was 7.00 CFU/cm2 in split AC for sodium hypochlorite and 6.33CFU/cm2 in split AC for QAC. Samples were not positive after disinfection with QAC or sodium hypochlorite (20 min or 2 h). Central AC had lower mean bacterial count.
Styaningsih 2019 (2926)	Gram-positive cocci (*Staphylococcus epidermis*)	Single-site, quasi-experimental, controlled before-after study.	36 samples were collected from the floor of operating room of surgical unit at a hospital. Kudus City, Indonesia.	The efficacy of two disinfectants was compared: quaternary ammonium derivative (unspecified product or concentration) or sodium hypochlorite (unspecified product or concentration). Sampling was conducted prior to disinfection, after 20 minutes, and after 2 h from disinfection. Efficacy was compared between surgical rooms with centralized or split air conditioning (AC) systems.	Average S. epidermidis before disinfection was 8.33 CFU/cm2 in split AC for sodium hypochlorite and 7.67 CFU/cm2 in split AC for QAC. Samples were not positive after disinfection with QAC or sodium hypochlorite (20 min or 2 h). Central AC had lower mean bacterial count. Disinfectants were not significantly different.
Yuen 2015 (10160)	Gram-positive cocci (*Staphylococcus* spp.- MSSA, MRSA, coagulase-negative *Staphylococcus*)	Single site, uncontrolled before-after study, over 6-week period	864 samples from 4 high-touch bedside surfaces in a 6-bed cubical of a medical ward at a 1500-bed teaching hospital. Hong Kong, China.	The control periods included weeks 1, 3, and 5 and consisted of routine disinfection with sodium hypochlorite wipes once per day. The intervention periods included weeks 2, 4, and 6 and included routine disinfection followed by an applied QAC spray (JUC spray) to all bed-units in the cubicle three times per week. Samples were always collected 1 h and 5 h after routine cleaning. During the intervention periods, the JUC spray was applied immediately after samples were collected 1 h after routine disinfection. The same ward and 6-bed cubicle were used during the intervention and control periods.	During control periods, 78% (14/18) beds were positive for staphylococcal bacteria. During the intervention periods, 11% (2/18) were positive (significance not specified). In the control period, 5 h after hypochlorite disinfection, mean staphylococcal contamination increased significantly 80% (p<0.01) while it decreased significantly in the intervention group with the application of the JUC spray (p<0.001). In the experimental group the mean staphylococcal concentration of the bedside surfaces was 4.4 CFU/cm2 1 h

Reference (Study ID)	Outcome	Study Design	Setting	Intervention	Results
					after hypochlorite disinfection (before JUC spray) to 0.7 CFU/cm2 (5 h after hypochlorite and 4 h after JUC spray). In the control periods, mean concentration at 1 h after hypochlorite was ~1.5 CFU/cm2 and at 5 h after hypochlorite was ~4.1 CFU/cm2 (significant increase of 80%, $p<0.01$).
Orenstein 2011 (8042)	HAI (*C. difficile*)	Single site, uncontrolled before-after study over 2 years	Patients in 2 medical units with high endemic CDI at a 1249-bed hospital in Minnesota, USA	During the pre-intervention period for one year, daily cleaning and terminal disinfection consisted of a QAC (HB-Quat). For the second year, an intervention replaced the QAC with 0.55% sodium hypochlorite wipes (Clorox, 10 min contact time). All CDI cases were reviewed and validated by an expert. High compliance was observed in both periods.	In the pre-intervention period, there were 16 and 15 cases/10,000 patient-days in each of the units, respectively. During the intervention period the incidence on both units was reduced to 3.5 and 3.7 cases/10,000 patient-days on each of the units, respectively. The hospital-acquired CDI over a 12-month period decreased by 85% (from 24.2 to 3.6 cases/10,000 patient-days, $p<0.001$).
Hacek 2010 (5852)	HAI (*Clostridium difficile*)	Multi-site, Quasi-experimental uncontrolled before-after study over 3 years	Monthly *C. difficile* infections were collected from the hospital databases and defined as having a positive stool test > 48 h after admission. The study was conducted in 3-hospital system in Illinois, USA	During the entire study period daily cleaning of patient rooms used a QAC (unspecified product, active ingredients). During the 10-month pre-intervention period, QAC was also used for terminal disinfection. The 2-year intervention period replaced QAC with 5000 ppm bleach during terminal cleaning.	There was a significant reduction ($p<0.0001$) in average number of CDI patients per 1000 patient-days during the intervention period with 0.85 in the pre-intervention period (QAC terminal cleaning) compared to 0.45 (bleach terminal cleaning).
Anderson 2018 (6885)	HAI (HAI-Methicillin-resistant *Staphylococcus aureus* (MRSA))	Multisite, quasi-experimental, cohort study	Hospital-wide HAI acquisition over 271740 patients and 375918 admissions 9 hospitals including	Four experimental periods of 6 months each, with a 1-month "wash" period between each using different terminal disinfection. Standard terminal disinfection (reference) was performed using a low-alcohol solution QAC (6.5% octyl	For standard disinfection period/reference period with QAC disinfection, hospital-wide incident cases of MRSA infections were 204 (0.27% of exposed

Reference (Study ID)	Outcome	Study Design	Setting	Intervention	Results
		over a period of 28 months	university, tertiary care, regional, community, and VA hospital settings, USA	decyl dimethyl ammonium chloride, 2.6% dioctyl dimethyl ammonium chloride, 3.9% didecyl dimethyl ammonium chloride, 8.7% alkyl dimethyl benzyl ammonium chloride; Quaternary Disinfectant Cleaner; Ecolab) applied with microfiber cloth at an unspecified final dilution. Standard terminal cleaning was compared to 3 enhanced protocol methods including QAC disinfection followed by UV light disinfection; bleach disinfection (10% hypochlorite, Clorox Germicidal Wipes); and bleach disinfection followed by UV light disinfection. Wet contact time, time until measurement unspecified.	admissions) with 5.66 per 10,000 patient-days. During the bleach-only disinfection period, incidence was 5.88 per 10,000 patient days and relative risk (95% CI) was not significantly different at 0.97 (0.76-1.24), p=0.82.
Anderson 2018 (6885)	HAI (HAI-All target pathogens)	Multisite, quasi-experimental, cohort study over a period of 28 months	Hospital-wide HAI acquisition over 271740 patients and 375918 admissions 9 hospitals including university, tertiary care, regional, community, and VA hospital settings, USA	Four experimental periods of 6 months each, with a 1-month "wash" period between each. Standard terminal disinfection (control) was performed using a low-alcohol solution QAC (6.5% octyl decyl dimethyl ammonium chloride, 2.6% dioctyl dimethyl ammonium chloride, 3.9% didecyl dimethyl ammonium chloride, 8.7% alkyl dimethyl benzyl ammonium chloride; Quaternary Disinfectant Cleaner; Ecolab) applied with microfiber cloth at an unspecified final dilution. Standard terminal cleaning was compared to 3 enhanced protocol methods including QAC disinfection followed by UV light disinfection; bleach disinfection; and bleach disinfection followed by UV light disinfection. Wet contact time, time until measurement unspecified.	For standard disinfection period/reference period with QAC disinfection, hospital-wide incident cases of all targeted organisms (MRSA, VRE, MDR *Acinetobacter* spp., *C. difficile*) infections were 626 (0.86% of exposed admissions) with 18.1 per 10,000 patient-days. During the bleach-only disinfection period, incidence was 17.5 per 10,000 patient days and relative risk (95% CI) was not significantly different at 0.92 (0.79-1.08), p=0.32.
Anderson 2017 (6887)	HAI (HAI-All target pathogens)	Multi-site, controlled cohort study. 27- month period	21,395 patients met inclusion criteria as an exposed patient who was admitted to room with prior occupant with proven infection or	Four experimental periods of 6 months each, with a 1-month "wash" period between each. Standard terminal disinfection (control) was performed using a low-alcohol solution QAC (6.5% octyl decyl dimethyl ammonium chloride, 2.6% dioctyl dimethyl ammonium chloride, 3.9% didecyl dimethyl ammonium chloride,	Incidence for reference/QAC was 115 cases among exposed patients (2.3%) compared to incidence among bleach group of 101 (1.9%). Rate was 51.3/10,000 patient-days for QAC compared to 41.6/10,000 patient-days for

Reference (Study ID)	Outcome	Study Design	Setting	Intervention	Results
			colonization with organism. 4916 patients from reference group, 5438 in bleach group. Conducted at nine hospitals including tertiary, community, Veterans Affairs hospitals in the southeastern USA.	8.7% alkyl dimethyl benzyl ammonium chloride; Quaternary Disinfectant Cleaner; Ecolab) applied with microfiber cloth at an unspecified final dilution. Standard terminal cleaning was compared to 3 enhanced protocol methods including QAC disinfection followed by UV light disinfection; bleach disinfection; and bleach disinfection followed by UV light disinfection. Wet contact time, time until measurement unspecified.	bleach. Incidence was not significantly different with rate ratio (95% CI) was 0.85 (0.69-1.04) for disinfection with bleach compared to QAC for all target organisms (*C. difficile*, MRSA, VRE, MDR *Acinetobacter*).
Manian 2013 (5113)	HAI (HAI-*C. difficile*)	Single site, uncontrolled before-after study, 3 years	Patients with nosocomial *C. difficile* infection following 72 h after admission with positive test for cytotoxin A or B. suburban 900-bed community teaching medical center. Missouri, USA.	The pre-intervention period spanned two years and included terminal cleaning with 0.525% bleach when formerly occupied by patient colonized with antibiotic-resistant pathogen (VRE, MRSA, A. baumannii, gram-negative bacilli) or *C. difficile*. HPV disinfection (Bioquell, cycle time 3-4 h) was added to terminal cleaning for intervention period (1 year).	There was a significant reduction in *C. difficile* associated diarrhea rates from 322 cases, 0.88/1000 patient-days during the pre-intervention period compared to 109 cases, 0.55/1000 patient-days during the intervention period (rate ratio=0.63, 95% CI: 0.50-0.79, p<0.001)
Wilcox 2003 (3854)	HAI (HAI-*Clostridium difficile*)	Single site, crossover study over 2 years	1128 environmental samples (floor, radiator, bedframe, toilet floor, sluice floor, cleaner floor, commode, side room floors, side room curtain rails) at two elderly medicine wards in Leeds, UK	Two similar wards were cleaned with one or the other cleaning regiment for 6-12 month periods with either neutral liquid detergent (Hospec) or detergent followed by 1000 ppm hypochlorite (Saniclor, 12.5% sodium hypochlorite). CDI was diagnosed by laboratory and confounding variables measured included hand contamination and antibiotics use.	In ward X there was a significant decrease of CDI from 8.9 to 5.3 cases/100 admissions (p<0.05) using sodium hypochlorite, however there was not a significant decrease in the second ward. CDI was significantly associated with the proportion of culture-positive sites in ward X.
Simon Garcia 2009 (7960)	HAI (HAI-drug resistant organisms-ESBL, *Pseudomonas aeruginosa*, MRSA,	Single site, uncontrolled before-after study over 2 years	Nosocomial infections were assessed (method unspecified) among 3,556 patients in the pre-intervention	A one-year pre-intervention period and one-year post-intervention period were compared. Samples were collected before and after a disinfection intervention to control an outbreak. The disinfection consisted of QAC disinfection	In the pre-intervention period, the incidence of all nosocomial infection was 3.2 episodes/100 patients or 9.2 infections/1000 patient-days compared to 1.6 episodes/100 patients or 5.0

Reference (Study ID)	Outcome	Study Design	Setting	Intervention	Results
	Stenotrophomona maltophilia)		period and 3,662 patients in the post-intervention period.	on furniture and equipment and 5% bleach solution on floors and walls.	infections/1000 patient-days in the post-intervention period. MDR HAI incidence rate decreased significantly in the post-intervention period at 1.83. Incidence ratio (95% confidence interval) of nosocomial infections indicated significantly higher ratio in pre-intervention period compared to post-intervention period for all MDROs at 1.83 (1.34-2.50). Additionally, looking at specific MDR organisms, incidence ratio (95% confidence interval) for HAIs due to P. *aeruginosa* was 2.36 (1.41-3.96), ESBL *Enterobacteriaceae* was 2.31 (1.11-4.82), Stenotrophomono maltophilia was 2.77 (1.10-6.99). Incidence ratio for MRSA was not significantly different between periods at 0.92 (0.52-1.65)
Anderson 2017 (6887)	HAI (HAI-MDR *Acinetobacter* spp)	Multi-site, controlled cohort study. 27- month period	21,395 patients met inclusion criteria as an exposed patient who was admitted to room with prior occupant with proven infection or colonization with organism. 4916 patients from reference group, 5438 in bleach group. Conducted at nine hospitals including tertiary, community, Veterans Affairs	Four experimental periods of 6 months each, with a 1-month "wash" period between each. Standard terminal disinfection (control) was performed using a low-alcohol solution QAC (6.5% octyl decyl dimethyl ammonium chloride, 2.6% dioctyl dimethyl ammonium chloride, 3.9% didecyl dimethyl ammonium chloride, 8.7% alkyl dimethyl benzyl ammonium chloride; Quaternary Disinfectant Cleaner; Ecolab) applied with microfiber cloth at an unspecified final dilution. Standard terminal cleaning was compared to 3 enhanced protocol methods including QAC disinfection followed by UV light disinfection; bleach disinfection; and bleach disinfection followed by UV light	Only one case was observed. No significant difference between bleach and QAC disinfection for MDR Acintenobacter.

Reference (Study ID)	Outcome	Study Design	Setting	Intervention	Results
			hospitals in the southeastern USA.	disinfection. Wet contact time, time until measurement unspecified.	
Anderson 2017 (6887)	HAI (HAI-MRSA)	Multi-site, controlled cohort study. 27- month period	21,395 patients met inclusion criteria as an exposed patient who was admitted to room with prior occupant with proven infection or colonization with organism. 4916 patients from reference group, 5438 in bleach group. Conducted at nine hospitals including tertiary, community, Veterans Affairs hospitals in the southeastern USA.	Four experimental periods of 6 months each, with a 1-month "wash" period between each. Standard terminal disinfection (control) was performed using a low-alcohol solution QAC (6.5% octyl decyl dimethyl ammonium chloride, 2.6% dioctyl dimethyl ammonium chloride, 3.9% didecyl dimethyl ammonium chloride, 8.7% alkyl dimethyl benzyl ammonium chloride; Quaternary Disinfectant Cleaner; Ecolab) applied with microfiber cloth at an unspecified final dilution. Standard terminal cleaning was compared to 3 enhanced protocol methods including QAC disinfection followed by UV light disinfection; bleach disinfection; and bleach disinfection followed by UV light disinfection. Wet contact time, time until measurement unspecified.	Incidence for reference/QAC was 73 cases among exposed patients (2.2%) compared to incidence among bleach group of 74 (2.0%). Rate was 50.3/10,000 patient-days for QAC compared to 48.2/10,000 patient-days for bleach. Incidence was not significantly different with rate ratio (95% CI) was 1.00 (0.82-1.21) for disinfection with bleach compared to QAC for MRSA.
Anderson 2018 (6885)	HAI (HAI-Multidrug-resistant *Acinetobacter* spp. (MDR-A))	Multisite, quasi-experimental, cohort study over a period of 28 months	Hospital-wide HAI acquisition over 271740 patients and 375918 admissions 9 hospitals including university, tertiary care, regional, community, and VA hospital settings, USA	Four experimental periods of 6 months each, with a 1-month "wash" period between each. Standard terminal disinfection (control) was performed using a low-alcohol solution QAC (6.5% octyl decyl dimethyl ammonium chloride, 2.6% dioctyl dimethyl ammonium chloride, 3.9% didecyl dimethyl ammonium chloride, 8.7% alkyl dimethyl benzyl ammonium chloride; Quaternary Disinfectant Cleaner; Ecolab) applied with microfiber cloth at an unspecified final dilution. Standard terminal cleaning was compared to 3 enhanced protocol methods including QAC disinfection followed by UV light disinfection; bleach disinfection; and bleach disinfection followed by UV light disinfection. Wet contact time, time until measurement unspecified.	For standard disinfection period/reference period with QAC disinfection, hospital-wide incident cases MDR *Acinetobacter* spp. infections were 6 cases (0.01% of exposed admissions) with incidence of 0.18 per 10,000 patient-days. During the bleach-only disinfection period, incidence was 0.11 per 10,000 patient days and relative risk (95% CI) was significantly different ($p<0.05$) at 0.07 (-0.12-0.26).

Reference (Study ID)	Outcome	Study Design	Setting	Intervention	Results
Anderson 2018 (6885)	HAI (HAI-Vancomycin-resistant *Enterococcus* (VRE))	Multisite, quasi-experimental, cohort study over a period of 28 months	Hospital-wide HAI acquisition over 271740 patients and 375918 admissions 9 hospitals including university, tertiary care, regional, community, and VA hospital settings, USA	Four experimental periods of 6 months each, with a 1-month "wash" period between each. Standard terminal disinfection (control) was performed using a low-alcohol solution QAC (6.5% octyl decyl dimethyl ammonium chloride, 2.6% dioctyl dimethyl ammonium chloride, 3.9% didecyl dimethyl ammonium chloride, 8.7% alkyl dimethyl benzyl ammonium chloride; Quaternary Disinfectant Cleaner; Ecolab) applied with microfiber cloth at an unspecified final dilution. Standard terminal cleaning was compared to 3 enhanced protocol methods including QAC disinfection followed by UV light disinfection; bleach disinfection; and bleach disinfection followed by UV light disinfection. Wet contact time, time until measurement unspecified.	For standard disinfection period/reference period with QAC disinfection, hospital-wide incident cases of VRE infections were 121 (0.16% of exposed admissions) with 3.24 per 10,000 patient-days. During the bleach-only disinfection period, incidence was 4.62 per 10,000 patient days and relative risk (95% CI) was not significantly different at 0.87 (0.65-1.17), p=0.35.
Anderson 2017 (6887)	HAI (HAI-VRE)	Multi-site, controlled cohort study. 27- month period	21,395 patients met inclusion criteria as an exposed patient who was admitted to room with prior occupant with proven infection or colonization with organism. 4916 patients from reference group, 5438 in bleach group. Conducted at nine hospitals including tertiary, community, Veterans Affairs hospitals in the southeastern USA.	Four experimental periods of 6 months each, with a 1-month "wash" period between each. Standard terminal disinfection (control) was performed using a low-alcohol solution QAC (6.5% octyl decyl dimethyl ammonium chloride, 2.6% dioctyl dimethyl ammonium chloride, 3.9% didecyl dimethyl ammonium chloride, 8.7% alkyl dimethyl benzyl ammonium chloride; Quaternary Disinfectant Cleaner; Ecolab) applied with microfiber cloth at an unspecified final dilution. Standard terminal cleaning was compared to 3 enhanced protocol methods including QAC disinfection followed by UV light disinfection; bleach disinfection; and bleach disinfection followed by UV light disinfection. Wet contact time, time until measurement unspecified.	Incidence for reference/QAC was 37 cases among exposed patients (3.5%) compared to incidence among bleach group of 24 (1.6%). Rate was 63.4/10,000 patient-days for QAC compared to 31.9/10,000 patient-days for bleach. Incidence was significantly different (p=0.049) with rate ratio (95% CI) was 0.43 (0.19-1.00) for disinfection with bleach compared to QAC for VRE.
Aucella 2000 (14850)	HAI (Hepatitis C Virus)	Multi-site, prospective	135 patients enrolled for the prospective	During Period A (24 months) positive and negative patients shared the same monitors and systematic monitor disinfection was performed.	When compared to historical data (3 years without disinfection use), prevalence of HCV infection

Reference (Study ID)	Outcome	Study Design	Setting	Intervention	Results
		study over 5 years	study at 4 dialysis centers in Italy	In period B (36 months), 3 units continued the same strategy while one unit had an intervention that included strict separation for machines for anti-HCV positive subjects and no dialyzer was reused. Disinfection consisted of 7% sodium hypochlorite solution after each dialysis session. Patients were tested monthly for HCV.	reduced 35% to 31% over periods A and B and incidence reduced from 2.8% to 0.4%. This study concluded that monitor disinfection was more effective than the use of separate machines for anti-HCV-positive patients, however statistical analysis not reported.
Youkee 2015 (3432)	Virus (Ebola virus RNA)	Single-site, uncontrolled before-after study	173 samples collected from surfaces (bed, bedframe, mattress, latrine, wall, IV pole, bedside table, sharps bin, door handle, etc.) to assess decontamination procedures in an Ebola holding unit which is a 16-bed facility in an adult medical tertiary referral hospital in Freetown, Sierra Leone.	The unit was cleaned 5 times every 24 h which consisted of spraying and mopping all surfaces and floors. Terminal cleaning was also conducted. Cleaning fluid was 0.5% sodium hypochlorite reconstituted 6 times a day from sodium hypochlorite powder. Samples were collected immediately after Ebola PCR-positive patient, 30 min after terminal cleaning, and 60-min after terminal cleaning.	Of 14 non-porous surface types assessed, there were 29% (8/28) surfaces positive prior to terminal disinfection. After 30 min or 60 min of disinfection, 19% (5/28) surfaces were positive for the viral RNA. After a re-training intervention, 2/28 surfaces were positive before disinfection and no surfaces were positive after disinfection. Significance not assessed.

Table S15: Study results for manually applied chlorine interventions (excepting sodium hypochlorite) ordered by outcome organism

Reference (Study ID)	Outcome	Study Design	Setting	Intervention	Results
Casey 2010 (125)	All viable organisms	Single-site, Quasi-experimental crossover study, over 10 weeks	400 samples from 5 high-touch surfaces (door pushplate, faucet handles, toilet seat) from an open ward and patient washrooms of an acute medical ward in a university hospital. Queen Elizabeth Hospital, Birmingham, UK	Coated surfaces were compared to equivalent items with plastic, chrome-plated, or aluminum surfaces. Coated surfaces were pure copper/resin composite (~70% copper) toilet seat; brass (~60 Cu) faucet handles; brass (~70% Cu) door pushplate. Standard cleaning product used was demand-release chlorine Chlor-clean (sodium dichloroisocyanurate with 1000 ppm available chlorine and detergent). All surfaces disinfected 4 times every 2 h. Samples were collected once a week before daily cleaning began and after daily cleaning ended. Copper-containing and non-copper containing items were interchanged after 5 weeks.	The range of median total aerobic CFU/cm2 for copper-containing surfaces was 0 – 2.1 with maximum of 38.4 compared to 0.6 – 87.6 with maximum of 266.4 for control surfaces. All copper surfaces had significantly lower concentration compared to control surfaces ($p<0.05$) except for one of the comparisons. There was no significant difference in microbial count on control items before daily cleaning compared to after daily cleaning (median=3.6 vs. 2.1 CFU/cm2, $p=0.97$). Median numbers of microorganisms on copper surfaces were 90% to 100% lower than their control equivalents at both time points. 50% of control sample points and 0% of copper points had median counts > 5 CFU/cm2.
Chen 2017 (177)	All viable organisms	Single site, controlled cohort study over two weeks	72 samples from high-touch surfaces (bed, monitor, ventilator, stethoscope, keyboard, computer mouse) near 16 beds in a medical intensive care unit (MICU) and 12 beds in a respiratory care center (RCC) at a regional teaching hospital. Changhua City, Taiwan.	In one ward in the RCC, disinfection was not daily but only terminal. In a second ward in the MICU, there was daily and terminal disinfection. Disinfection consisted of 500 ppm hypochlorite wipes (unspecified hypochlorite, 30 min contact time for terminal disinfection only). Samples were collected 30 min after terminal disinfection but before new patient admission, and on days 3, 7, and 14 with hospitalized patient. In the MICU, sampling on days 3,7, and 14 was conducted 20 h after daily cleaning. Gene abundance was assessed using 16s ribosomal RNA metagenomics.	There was a significantly higher abundance of *Acinetobacter* spp., *Streptococcus* spp., and *Pseudomonas* spp. in the RCC compared to the MICU. There was not a significantly higher alpha-diversity on days 0, 3, and 7, but significantly higher alpha-diversity in the RCC on day 14
Al-Hamad 2008 (516)	All viable organisms	Single site, uncontrolled before-after	Samples were collected from clinical patient areas	Daily cleaning consisted of detergent on floors, door handles, and sinks. Terminal cleaning consisted of	Total aerobic count on clinical surfaces ranged from a mean of < 1 CFU/cm2 to a mean of ~7.5 CFU/cm2 before cleaning.

Reference (Study ID)	Outcome	Study Design	Setting	Intervention	Results
		study, over 4 weeks	(bed frame, cabinet horizontal surface, door handle, monitor and control panel, cabinet handle, soap dispenser, chart table, sink tap handle) from a seven-bedded ICU and five-bedded high dependency unit (HDU) at large district general hospital in the UK. Non-clinical areas were also sampled.	detergent followed by hypochlorite (unspecified hypochlorite, concentration, product, contact time) on surfaces except electrical equipment (70% alcohol). Clinical areas samples were randomly collected before cleaning and immediately after terminal cleaning.	After cleaning, the total aerobic count ranged from a mean of < 1 CFU/cm2 to ~2 CFU/cm2. Bed frames, cabinet horizontal surfaces, and door handles had the highest mean count prior to cleaning.
Allen 2019 (536)	All viable organisms	Single-site, Quasi-experimental, uncontrolled before-after study	50 from 5 surfaces (desk, patient chair, sink tap and sink upper surface, door and handle, spirometer equipment) in cystic fibrosis and chronic infection outpatient clinic rooms. Cambridge, UK.	Samples were taken after the patient left the room and after manual cleaning with demand-release chlorine-dioxide disinfectant (Tristel, Tristel Solutions Ltd). Unspecified concentrations or contact times. Surfaces were left to dry before sampling.	When initial load was > 50 CFU, disinfection with Tristel showed significant (p<0.025) reduction. Mean bacterial count (CFU) decreased from ~25 to ~20 on spirometers, ~250 to ~50 on sinks, ~30 to ~25 on door handles, ~100 to ~20 on desks, and from ~130 to ~40 on chair surfaces.
Andersen 2006 (567)	All viable organisms	Single-site, Quasi-experimental study, controlled before-after study	207 samples from 26 surface locations (floor, wall, windowsill, and other high-touch surfaces) in four patient isolation units at a university hospital. Oslo, Norway.	Daily pre-cleaning with soap and water to remove visible organic materials and soils. Terminal cleaning consisted of disinfection with demand-release 5% chloramine over 1h contact time or 5% chloramine over 1h contact time + rinse with soap and water. Samples taken within 10 minutes of before cleaning, after cleaning, and after disinfection with chloramine.	Mean (standard deviation) bacterial count before cleaning or disinfection was 30.9 (11.9) CFU/plate. There was a significant decrease to 1.3 (2.6) with chloramine only and to 4.1 (6.0) with cleaning followed by chloramine (p<0.001). However there was not a significant reduction before compared to after cleaning with soap and water.

Reference (Study ID)	Outcome	Study Design	Setting	Intervention	Results
Frabetti 2009 (1024)	All viable organisms	Single-site, Quasi-experimental, uncontrolled before-after study, over 2 months	A total of 2124 samples collected from 12 surfaces (wall, floor, and furnishing) Surfaces were within six operating rooms in a hospital. Ferrara, Italy.	Dry dust mopping with microfiber mop (before) was compared to wet cleaning (after) with demand-release 400 ppm available chlorine (sodium dichloroisocyanurate) on microfiber cloths. Unspecified contact time. Surfaces sampled before wet cleaning, 30 minutes after wet cleaning, and 12 hours after wet cleaning.	Mean total microbial concentration (SD) after dry cleaning alone compared to 30 min after disinfection had non-significant (p>0.05) reductions ranging from 8.3%-79.6%. Average of the initial concentration on different surfaces ranged from 1.5 – 5.98 CFU/100 cm2 before disinfection compared to and 0.82 – 2.21 CFU/100 cm2 after 30 min from disinfection. After 12 hours from disinfection, mean concentration ranged from 1.82 – 9.28 CFU/100 cm2. Higher bacterial counts were observed on horizontal surfaces compared to vertical surfaces.
Galván Contreras 2016 (1079)	All viable organisms	Single-site, Quasi-experimental study, controlled before-after study	84 samples from 21 surfaces (floors, walls, and ceilings) were sampled across adult ICU, neonatal ICU, emergency department, surgical unit, operating room of a hospital environment. Mexico City, Mexico.	6% sodium hypochlorite solution was compared to bromo-chloro-dimethyl-hydantoin (BCDMH) disinfectant (BCDMH Sanitizing Solution, GV-GERM) (diluted to 1-part sanitizer 3 parts water) applied through either sprayer or directly with flannel material. Sodium hypochlorite was 200 ppm in non-critical areas, 500 ppm in semi-critical areas and 5,000 ppm in critical areas. Unspecified contact times. Samples measured before disinfection interventions and 15 minutes after.	Percent surfaces positive was 13/ 21 surfaces before compared to 0/21 surfaces after BCDMH. Percent surfaces positive was 9/21 surfaces before disinfection and 2/21 surfaces after hypochlorite disinfection. There was no difference in disinfectant type (p=0.4). Reductions before compared to after were not significantly lower (p=0.15).
Hosein 2016 (1280)	All viable organisms	Single-site, Quasi-experimental, uncontrolled before-after study over 5 months	368 samples from 5 specific high-touch surfaces (bedrail, bathroom handrail, tray table, toilet seat, bathroom faucet) were sampled in 40 single-occupancy isolation rooms of	Standard terminal cleaning consisted of cleaning with demand-release hypochlorous acid disinfectant solution (1,000 ppm (0.1%) chlorine, sodium dichloroisocyanurate, Actichlor Plus). Unspecified contact time for terminal cleaning. Samples were collected after patient discharge but before standard terminal cleaning (baseline) and after	Mean bacterial count (standard deviation) averaged among all surfaces in rooms was reduced from 19.5 (26.1) CFU at baseline sampling to 7.6 (16.8) CFU following terminal cleaning (~61% decrease). Mean (standard deviation) reductions in bacterial count was significantly lower after baseline cleaning at 11.9 (24.6) CFU (p<0.01).

Reference (Study ID)	Outcome	Study Design	Setting	Intervention	Results
			acute medical assessment units in the 700-bed hospital. London, UK.	terminal cleaning when surfaces were dry	Additionally, percent (number) of samples positive was significantly higher (p<0.01) at baseline (86%, 158/184) compared to after terminal cleaning (56%, 103/184)
Hall 2011 (1723)	All viable organisms	Single site, quasi-experimental, controlled crossover study for 12 weeks	Samples (unspecified number) were collected from ten surfaces in the bathroom or patient area (e.g. floor, table, locker top, chair arm) in four wards comprising elderly care and surgical patients at Mayday Healthcare NHS Trust Hospital, UK	Standard daily cleaning with 1,000 ppm demand-release chlorine agent (sodium dichloroisocyanurate, Actichlor Plus) was compared to daily cleaning with ultramicrofiber (UMF) cloths/mops (Vikan Ltd) with or without a copper-based biocide (ICICS Ltd, CuWB50, 300 ppm). Samples were taken one hour before and one hour after cleaning. After 4 weeks, cleaning was interchanged for another 4 weeks.	CuWB50 and standard cleaning significantly reduced concentration; UMF with water only did not significantly reduce bacterial concentration. CuWB50 showed a significant (p=0.003) reduction of median concentration from 78 total viable count to 50 total viable count. In multivariate analysis, UMF + CuWB50 showed a 69% reduction of mean concentration (p<0.001) and 51% reduction in RLU (p<0.001), which was due to the direct and residual effects of CuWB50 (25% reduction, p=0.001) and residual effect which lasted for nearly a week (12%, p=0.001). Following standard cleaning, there was a significant 47.7% reduction (p<0.001) in median count from 100 to 71. Median ATP bioluminescence also had significant reduction from 500 to 250 RLU after standard cleaning.
Johnson 2016 (1979)	All viable organisms	Single-Site, quasi-experimental, uncontrolled study	160 samples from 8 touchpoints (bedrail, mattress, call bell, bedside table, handrail, etc.) in 20 hospital rooms. Hereford, UK.	Terminal cleaning included chlorine-dioxide disinfectant (Tristel Fuse, unspecified concentration/contact time). Samples collected before and after cleaning.	Average total count before cleaning was 27.4 CFU compared to after chlorine-dioxide cleaning at 19.3 CFU. Significance not specified.
Jones 2015 (1991)	All viable organisms	Single-site, Quasi-experimental, controlled	399 samples from bedside keyboards in intensive care unit at NHS Foundation	Intervention compared daily disinfection with demand-release chlorine dioxide spray (Tristel Fuse, 5 min contact time) to daily disinfection	During the intervention period, mean ± standard deviation (median) concentration after each of the disinfectants increased over time,

Reference (Study ID)	Outcome	Study Design	Setting	Intervention	Results
		cohort study over 16 days	Trust hospital. Norfolk, UK	with 2% chlorhexidine gluconate spray (70% alcohol, Hydrex Pink, unspecified contact time). Samples were taken from keyboards 0 h, 4-6 h, and 24 h after they were sprayed with either CHG spray or Tristel Fuse spray. Baseline samples were taken before intervention and 2 weeks after CHG intervention only.	although CHG was more effective than Tristel Fuse at all time points (p=0.002). For CHG, concentration increased from 0±0 (0) CFU after 0 h to 0.13±43 (0) CFU after 4-6 h to 4.21±10.72 (0) CFU after 24 h. For chlorine-dioxide, concentration increased from 2.54±6.78 (0) CFU after 0 h to 7.75±14.90 (2) CFU after 4-6 h to 68.23±133.24 (7.5) CFU after 24 h. There was a 60-fold reduction in bacterial burden at 4-6 hours after chlorhexidine cleaning compared with Tristel Fuse, and a 16-fold reduction at 24. Baseline samples had significantly higher (p<0.001) median CFU count >500 compared to 2 weeks after CHG intervention with median of 0. hours.
Ojajärvi 1976 (2228)	All viable organisms	Single site, controlled cohort study over 6 weeks	720 samples were taken from floors, patient rooms, and non-vertical environmental surfaces in infectious disease ward and ICU at the Children's Hospital in Helsinki University Central Hospital, Finland	Disinfectant was applied on the floors of ICUs three times a day and floors of patient rooms in infectious disease ward once a day. Other surfaces were wiped with the disinfectant. Surfaces were not rinsed with water. After two weeks using the disinfectant, samples were taken four hours after cleaning for three consecutive days. Samples were not taken prior to disinfection. Disinfectants included K 644 (1.2% chlorinated trisodium phosphate and potassium bromide), Panasept (0.25% sodium dichloroisocyanurate) and Gevisol (0.5-1% arylated and halogenated phenols). Contact times not specified.	Higher average concentration was found on patient room floors and other surfaces after disinfection using Givisol (28 CFU/plate) compared to Panasept (17 CFU/plate) and K 644 (15CFU/plate) (significance unspecified). The lowest colony count on patient floors was due to K644 in one ward and due to Panasept in the other.
Oztoprak 2019 (2288)	All viable organisms	Single-site quasi experimental, controlled	5 high-touch surfaces (buttons, bedside table, bed rail, floor) from 3 rooms in 43-	Each of the following disinfectants was used in one of three rooms: steam technology (Tecnovap Evo 304) compared to two-step cleaning with	The following differences in average (standard deviation) ATP bioluminescence (RLU) was significantly lower (98%) after steam disinfection

Reference (Study ID)	Outcome	Study Design	Setting	Intervention	Results
		before-after study over one month	bed ICU at tertiary care hospital. Turkey	detergent and water on microfiber cloths followed by 1,000 ppm, or 5,000 ppm hypochlorite solution (sodium dichloroisocyanurate) wipes. Known organism concentration inoculated onto pre-cleaned surface. Samples taken 10 minutes after inoculation and after disinfection.	from 578 (76) to 9.5 (2.3) ($p < 0.001$). Steam cleaning had significantly lower ATP compared to hypochlorite solutions ($p<0.05$). Chlorine interventions also had significant reductions ($p<0.001$) in ATP from 651 (66) to 22 (5.2) with 1000 ppm and from 632 (64) to 14 (2.9) with 5000 ppm chlorine.
Turner 1974 (4060)	All viable organisms	Single site, uncontrolled before-after study over 1 day	45 surface samples from one hydrotherapy immersion tank used in a hospital burn unit at North Carolina, USA.	After patient use, tank was emptied, and tank items soaked in pails of detergent (Vesphene), scrubbed, and rinsed. Tank was refilled to 1,355 L and disinfected using 28 g calcium hypochlorite (contact time 20 minutes). Samples were collected from tank surfaces after first drain/prior to detergent cleaning, after scrubbing and rinsing, and after disinfection draining.	After emptying the tank, mean bacterial count was 372 CFU per contact plate. After mechanical scrubbing, mean count was significantly lower (94% reduction) at 22 CFU. After chlorine disinfection, mean count was 11 CFU.
Siani 2018 (4540)	All viable organisms	Single-site, Quasi-experimental, controlled crossover study, over 29 weeks	1,566 environmental samples from 11 high-touch surfaces (bed control, bed rails, tray table, call button, patient chair, drug locker, commode top, bathroom door handle, flush handle, toilet grab rail, toilet seat) in two identical surgical and cardiovascular wards at a 1,000-bed teaching hospital. Cardiff, UK.	Samples were collected during 5-week baseline period with standard disinfection. Standard disinfection consisted of cleaning with detergent followed by disinfection with 1,000 ppm chlorine (active ingredient not specified) soaked in a cotton cloth. The crossover intervention compared standard disinfection with modified disinfection, which consisted of peracetic acid/hydrogen peroxide wipe when activated with water. Intervention was 12 weeks of either standard or modified disinfection on one ward followed by 12 weeks with disinfection interchanged on the ward. Both wards received training. Contact time, manufacturer unspecified. Samples were collected weekly before daily disinfection.	During intervention, all sites had < 2.5 CFU/cm2 in both indicating training reduced bacterial load when compared to baseline samples. Reduction in total aerobic count was significantly higher ($p<0.001$) in both crossover periods with peracetic wipe compared with detergent + chlorine. The reintroduction of detergent+chlorine following the period using peracetic acid wipe had significant increase ($p<0.001$) in total aerobic count in 3/11 surface types. Total anaerobic count and ATP were not significantly different in one ward, but significantly higher ($p<0.001$) in another ward with the use of detergent+chlorine compared to the use of peracetic acid.

Reference (Study ID)	Outcome	Study Design	Setting	Intervention	Results
Ho 2016 (6163)	All viable organisms	Single-site, Quasi-experimental study, controlled before-after study	121 total samples were collected from 11 specific high-touch surfaces in 22 rooms of the hospital environment. Hualien, Taiwan.	Standard daily cleaning (before) with 0.06% sodium hypochlorite detergent disinfectant on microfiber cloths compared to modified daily cleaning (after) with demand-release chlorine sodium dichloroisocyanurate (NaDCC) tablets (Medentech, Wexford, Ireland) on microfiber cloths (concentration of active chlorine equivalent to that of 0.05% NaOCl). Unspecified contact time. Samples measured "before and after daily cleaning" for each intervention	Median aerobic colony counts before disinfection with sodium hypochlorite ranged from 0.8 – 51.9 CFU/cm2 compared to after sodium hypochlorite disinfection ranging from 0.1 – 23.9 CFU/cm2. Median count before disinfection with NaDCC ranged from 0.5 – 28.6 CFU/cm2 before compared to 0.0 – 9.2 CFU/cm2 after disinfection. 3 of 11 surface types had significantly lower concentration after disinfection with NaDCC compared to 4 of 11 surfaces types with significantly lower concentration after disinfection with sodium hypochlorite.
Karpanen 2012 (6414)	All viable organisms	Single-site, controlled cross-over study over a 24 week period	~672 samples from frequent-touch items (door handles and push plates, toilet seats and flush handles, grab rails, light switches and pull cord toggles, sockets, overbed tables, dressing trolleys, commodes, taps, and sink fittings) in acute care medical ward at a large university hospital. UK.	14 types of copper items were installed 3 months before beginning of the study and include surfaces with copper-alloy (58-99.5% copper). Comparator items were composed of anodized aluminum, steel, plastic, chromium plated brass, etc. Sampled weekly before afternoon cleaning which consisted of detergent, hot water, and 1000 ppm chlorine (sodium dichloroisocyanurate, product unspecified, contact time unspecified). Copper and control items were switched after 12 weeks.	Eight of 14 item types had significantly lower CFU counts on the copper surfaces than on the standard materials ($p<0.0001$). The other six items had reduced (but insignificant) microbial counts on copper surfaces compared to control. The largest median difference in total aerobic microbial load on copper vs standard door pull handles was 80.3 CFU/cm^2 on toilet flush lever handles from ~110 CFU/cm^2 to ~25 CFU/cm^2
Stewart 2014 (7891)	All viable organisms	Single-site, Quasi-experimental, uncontrolled before-after study, over 4 months	360 total samples collected from 4 sites (lockers, left and right cotsides, overbed tables) in 30 rooms in an acute care elderly ward at	Surfaces were sprayed with 1.5 mL of electrolyzed water (Salvesan, Aqualution, 10-15 s contact time) and subsequent wiping clean with a new detergent wipe (Tuffie detergent wipes; Vernacare) then allowed to dry. Samples were taken before (12-22 h	Average of total aerobic colony count (CFU/cm2) across all surfaces types was 4.3 CFU/cm2 and reduced to 1.65 (61.63% reduction) 1 h after cleaning ($p<0.0001$), remaining below baseline: 1.66 at 2h, 1.75 at 4h, 2.58 at 8h, 2.63 at 12 h, 3.53 at 24 h, and 3.68 at 48

Reference (Study ID)	Outcome	Study Design	Setting	Intervention	Results
			450-bed general hospital. Lanarkshire, UK.	after daily detergent disinfection) and after disinfection: 1, 2, 4, 8, 12, 24, and 48 h after disinfection. Experiment repeated 3 times.	h. There was a return to baseline at 24 h for 2 sites.
Siani 2018 (4540)	All viable organisms (MDRO-VRE, CRE, or ESBL)	Single-site, Quasi-experimental, controlled crossover study, over 29 weeks	1,566 environmental samples from 11 high-touch surfaces (bed control, bed rails, tray table, call button, patient chair, drug locker, commode top, bathroom door handle, flush handle, toilet grab rail, toilet seat) in two identical surgical and cardiovascular wards at a 1,000-bed teaching hospital. Cardiff, UK.	Samples were collected during 5-week baseline period with standard disinfection. Standard disinfection consisted of cleaning with detergent followed by disinfection with 1,000 ppm chlorine (active ingredient not specified) soaked in a cotton cloth. The crossover intervention compared standard disinfection with modified disinfection, which consisted of peracetic acid/hydrogen peroxide wipe when activated with water. Intervention was 12 weeks of either standard or modified disinfection on one ward followed by 12 weeks with disinfection interchanged on the ward. Both wards received training. Contact time, manufacturer unspecified. Samples were collected weekly before daily disinfection.	Percent samples positive for VRE, CRE, or ESBL during baseline period on wards 1 and 2, respectively, (7%, 35/522; 2.5%, 13/522 samples) was higher compared to hydrogen peroxide wipes (1%, 5/522; and 0.6% 3/522) and compared to detergent + chlorine (3%, 14/522; 1.3%7/522). Reductions compared to baseline could be due to training. Percent samples positive for MDROs was higher on wards using detergent + chlorine (3% and 1.3%) compared to wards using peracetic wipes (1% an d 0.6%). Significance not specified.
Gan 2017 (1081)	All viable organisms (MDROs-combined MRSA, VRE, ESBL-producing *Enterobacteriaceae*)	Single site, uncontrolled before-after study over 17-months	4577 samples from 10 high-touch surfaces (bed rail, bedside, supply cart rail, bedside table, chair arm, etc.) around the patient in a 25-bed general ICU at 1200-bed teaching hospital in traditional Chinese medicine in Hangzhou, China.	The disinfectant used throughout was 500 mg/L hypochlorite (unspecified product). The 3-month baseline period with conventional cleaning methods with polyester cloths re-used after 30 min disinfection in hypochlorite. First 3-month intervention changed the cloth to microfiber cloth with one cloth per patient zone; cloths not re-used. Second 3-month intervention period added fluorescent markers to ensure cleaning. Third 3-month intervention used three cloths for each patient zone. Finally, the fourth 3-month period removed the	Mean prevalence of positive surfaces decreased throughout the study period from 16.1% (112/695) during baseline to 9.5% (91/954) during first intervention to 4.7% (31/66) during the second intervention, to 2.8% (30/1068) during the third intervention, and 4.7% (56/1200) after the fourth intervention. Mean percent of MDRO-positive surfaces was significantly reduced by up to 83% during the third intervention period ($p<0.001$). Use of 3 clean cloths per patient zone significantly increased fluorescent marker removal. Baseline

Reference (Study ID)	Outcome	Study Design	Setting	Intervention	Results
				fluorescent marker feedback. Samples were collected monthly within 1 h of cleaning.	percent surfaces positive for MRSA was up to ~25%, for ESBL-producing organisms was up to ~32%, for VRE was up to 12%.
Ho 2016 (6163)	Gram-negative bacteria (*Acinetobacter baumannii*-CRAB)	Single-site, Quasi-experimental study, controlled before-after study	121 total samples were collected from 11 specific high-touch surfaces in 22 rooms of the hospital environment. Hualien, Taiwan.	Standard daily cleaning (before) with 0.06% sodium hypochlorite detergent disinfectant on microfiber cloths compared to modified daily cleaning (after) with demand-release chlorine sodium dichloroisocyanurate (NaDCC) tablets (Medentech, Wexford, Ireland) on microfiber cloths (concentration of active chlorine equivalent to that of 0.05% NaOCl). Unspecified contact time. Samples measured "before and after daily cleaning" for each intervention	Median (range) aerobic colony count in CFU/cm2 of CRAB across surfaces was 8.5 (0.0-16.1 range) before NaDCC disinfection compared to 0.5 (0.0 -0.6) after disinfection (~94% decrease, p<0.01.). Median (range) was 101.7 (0.0 – 201.8) before disinfection with sodium hypochlorite compared to not detectable after disinfection (p<0.01).
Oztoprak 2019 (2288)	Gram-negative bacteria (*Acinetobacter baumannii*-MDR)	Single-site quasi experimental, controlled before-after study over one month	5 high-touch surfaces (buttons, bedside table, bed rail, floor) from 3 rooms in 43-bed ICU at tertiary care hospital. Turkey	Each of the following disinfectants was used in one of three rooms: steam technology (Tecnovap Evo 304) compared to two-step cleaning with detergent and water on microfiber cloths followed by 1,000 ppm, or 5,000 ppm hypochlorite solution (sodium dichloroisocyanurate) wipes. Known organism concentration inoculated onto pre-cleaned surface. Samples taken 10 minutes after inoculation and after disinfection.	No bacterial growth after steam or hypochlorite disinfection. Initial concentration not reported.
Garvey 2016 (1096)	Gram-negative bacteria (Coliforms-carbapenamse producing organisms)	Single site, uncontrolled before-after study over a period < 1 week	30 samples were collected from macroscopically clean touch-points in vicinity of patient (bed frame, ventilator, drip stand, extract vent, floor) and in communal	Decontamination of a burns shock room was described using standard terminal cleaning with 1000 ppm hypochlorite (NaDCC Chlor Clean) followed by hydrogen peroxide misting (6%). A modified second clean comprised steam-cleaning, 2000 ppm hypochlorite (NaDCC Chlor Clean) and hydrogen peroxide misting (12%)	After terminal disinfection with 1000 ppm NaDCC and 6% HPV, some surfaces remained positive for carbapenemase-producing coliforms, however these organisms were not recovered after increasing concentrations of NaDCC to 2000 ppm and 12% HPV

Reference (Study ID)	Outcome	Study Design	Setting	Intervention	Results
			areas (trolley, sink tap, sink, window sill, door handle, etc.) in a burns unit at a university NHS Foundation Trust tertiary referral teaching hospital in Birmingham, UK.		
Karpanen 2012 (6414)	Gram-negative bacteria (Coliforms)	Single-site, controlled cross-over study over a 24 week period	~672 samples from frequent-touch items (door handles and push plates, toilet seats and flush handles, grab rails, light switches and pull cord toggles, sockets, overbed tables, dressing trolleys, commodes, taps, and sink fittings) in acute care medical ward at a large university hospital. UK.	14 types of copper items were installed 3 months before beginning of the study and include surfaces with copper-alloy (58-99.5% copper). Comparator items were composed of anodized aluminum, steel, plastic, chromium plated brass, etc. Sampled weekly before afternoon cleaning which consisted of detergent, hot water, and 1000 ppm chlorine (sodium dichloroisocyanurate, product unspecified, contact time unspecified). Copper and control items were switched after 12 weeks.	Coliforms were more often found on control surfaces compared to copper surfaces. Odds ratio (95% confidence ratio) of copper surface positive for Coliforms compared to control surface was 0.398 (0.229 – 0.692), p=0.001.
Oztoprak 2019 (2288)	Gram-negative bacteria (*Pseudomonas aeruginosa*- Carbapenem-resistant *Pseudomonas aeruginosa*)	Single-site quasi experimental, controlled before-after study over one month	5 high-touch surfaces (buttons, bedside table, bed rail, floor) from 3 rooms in 43-bed ICU at tertiary care hospital. Turkey	Each of the following disinfectants was used in one of three rooms: steam technology (Tecnovap Evo 304) compared to two-step cleaning with detergent and water on microfiber cloths followed by 1,000 ppm, or 5,000 ppm hypochlorite solution (sodium dichloroisocyanurate) wipes. Known organism concentration inoculated onto pre-cleaned surface. Samples taken 10 minutes after inoculation and after disinfection.	No bacterial growth after steam or hypochlorite disinfection. Initial concentration not reported.

Reference (Study ID)	Outcome	Study Design	Setting	Intervention	Results
Doan 2012 (414)	Gram-positive bacilli (*Clostridium difficile*)	Single-site, quasi-experimental, controlled before-after cohort study over 3 months	53 samples collected per intervention from high frequency contact surfaces from hospital environment (bedrails, door handles, light switches, nurse call bell, toilet, bed table, floor) in isolation rooms at Derby Hospital Foundation Trust. Derby, UK	*C. difficile* inoculated into rooms for 72 h. Samples taken prior to disinfection. Disinfection interventions (HPV, dry ozone, 1000 ppm chlorine, dry atomized steam, steam cleaning, peracetic acid wipes) were tested each in separate rooms to determine concentration reduction of with known concentration of *C. difficile* spores placed in rooms. Intervention with 1000 ppm demand-release chlorine (sodium dichloroisocyanurate, Actichlor Plus tablets, Ecolab, Swindon, UK) had over 30-minute contact time. Measurements taken after "designated time period specified by company guidelines."	Log10 reductions (in CFU/mL) were highest for hydrogen peroxide, 1000 ppm chlorine-releasing agent, and peracetic acid wipes at 2.303, 2.223, and 2.134 respectively. Standardized median log10 reductions were 2.301 (IQR: 0.935, 2.301) following disinfection with Actichlor.
Goldenberg 2012 (5957)	Gram-positive bacilli (*Clostridium difficile*)	Single-site quasi experimental, uncontrolled study over 4 months	332 surfaces (bed rails, call buttons; patient chairs,, bathrooms, toilets, side rooms, door handles, trays and handles) from 13 wards (general medical, surgery, elderly care, acute admissions, etc.) a hospital, UK	A pre-intervention period with standard cleaning (microfiber system with no chemical disinfectant; NaDCC 1000 ppm chlorine, Chlor-Clean when in contact with infectious patient) was compared to intervention period with chlorine-dioxide-based disinfection (Difficil-S, 2 min contact time). Samples were taken over 2 months during pre-intervention period and over 2 months during intervention period after introduction of chlorine dioxide. Sample time relative to disinfection not specified.	The percent surfaces positive was unaffected with 7.5% (9/120) during pre-intervention period compared to 8.0% (17/212) during intervention period. Significance not specified.
Karpanen 2012 (6414)	Gram-positive bacilli (*Clostridium difficile*)	Single-site, controlled cross-over study over a 24 week period	~672 samples from frequent-touch items (door handles and push plates, toilet seats and flush handles, grab rails, light switches and	14 types of copper items were installed 3 months before beginning of the study and include surfaces with copper-alloy (58-99.5% copper). Comparator items were composed of anodized aluminum, steel, plastic, chromium plated brass, etc. Sampled weekly before afternoon	*C. difficile* was found similarly on copper and control surfaces. Odds ratio (95% confidence ratio) of copper surface positive for *C. difficile* compared to control surface was 3.920 (0.828 – 18.551), p=0.108.

Reference (Study ID)	Outcome	Study Design	Setting	Intervention	Results
			pull cord toggles, sockets, overbed tables, dressing trolleys, commodes, taps, and sink fittings) in acute care medical ward at a large university hospital. UK.	cleaning which consisted of detergent, hot water, and 1000 ppm chlorine (sodium dichloroisocyanurate, product unspecified, contact time unspecified). Copper and control items were switched after 12 weeks.	
Best 2014 (7122)	Gram-positive bacilli (*Clostridium difficile*)	Single site, Quasi-experimental, before and after, uncontrolled study over 10 months	342 samples from high, medium, and low-touch sites (beds, glove dispensers, bins, tables, chairs, handwash basins, curtain tracks, wall trunking, bases of beds, floor, bases of tables, other equipment) from day rooms and bathroom facilities in male and female section of 30-bed stroke rehabilitation unit at large ~2000 bed Teaching Hospital NHS Trust in Leeds, UK	A 7-day deep cleaning with 1000 ppm demand-release chlorine sporicidal disinfectant (Chlor-Clean, sodium dichloroisocyanurate) with unspecified contact time was conducted on ward. Immediately following, hydrogen peroxide vapor decontamination with 87 ppm atomized HPV (Deprox) ~2 h cycle time. Samples taken before deep cleaning, immediately after deep cleaning, the day after HPV, 19 days after HPV, and 20 weeks post-HPV.	Number of sites (% sites) positive for *C. difficile* decreased from 37/342 (10.8%) to 21/342 (6.1%) after deep cleaning with the chlorine-based sporicidal disinfectant to 0.9% (3) sites positive after HPV. After 19 days, no surfaces were positive. After 20 weeks, 3.5% (12) sites were positive. 92% overall reduction in sites positive for *C. difficile* using chlorine-based disinfectant and HPV. Deep cleaning reduced number of sites positive by 43% compared to HPV further reduced sites positive by 86%. (significance not specified).
Oztoprak 2019 (2288)	Gram-positive cocci (*Enterococcus* spp.-Vancomycin-resistant enterococci (VRE))	Single-site quasi experimental, controlled before-after study over one month	5 high-touch surfaces (buttons, bedside table, bed rail, floor) from 3 rooms in 43-bed ICU at tertiary care hospital. Turkey	Each of the following disinfectants was used in one of three rooms: steam technology (Tecnovap Evo 304) compared to two-step cleaning with detergent and water on microfiber cloths followed by 1,000 ppm, or 5,000 ppm hypochlorite solution (sodium dichloroisocyanurate) wipes. Known	No bacterial growth after steam or hypochlorite disinfection. Initial concentration not reported.

Reference (Study ID)	Outcome	Study Design	Setting	Intervention	Results
				organism concentration inoculated onto pre-cleaned surface. Samples taken 10 minutes after inoculation and after disinfection.	
Karpanen 2012 (6414)	Gram-positive cocci (*Enterococcus* spp.- Vancomycin-resistant enterococci (VRE))	Single-site, controlled cross-over study over a 24 week period	~672 samples from frequent-touch items (door handles and push plates, toilet seats and flush handles, grab rails, light switches and pull cord toggles, sockets, overbed tables, dressing trolleys, commodes, taps, and sink fittings) in acute care medical ward at a large university hospital. UK.	14 types of copper items were installed 3 months before beginning of the study and include surfaces with copper-alloy (58-99.5% copper). Comparator items were composed of anodized aluminum, steel, plastic, chromium plated brass, etc. Sampled weekly before afternoon cleaning which consisted of detergent, hot water, and 1000 ppm chlorine (sodium dichloroisocyanurate, product unspecified, contact time unspecified). Copper and control items were switched after 12 weeks.	VRE was more often found on control surfaces compared to copper surfaces. Odds ratio (95% confidence ratio) of copper surface positive for VRE compared to control surface was 0.095 (0.012 – 0.748), p=0.005.
Ho 2016 (6163)	Gram-positive cocci (*Enterococcus* spp.- VRE)	Single-site, Quasi-experimental study, controlled before-after study	121 total samples were collected from 11 specific high-touch surfaces in 22 rooms of the hospital environment. Hualien, Taiwan.	Standard daily cleaning (before) with 0.06% sodium hypochlorite detergent disinfectant on microfiber cloths compared to modified daily cleaning (after) with demand-release chlorine sodium dichloroisocyanurate (NaDCC) tablets (Medentech, Wexford, Ireland) on microfiber cloths (concentration of active chlorine equivalent to that of 0.05% NaOCl). Unspecified contact time. Samples measured "before and after daily cleaning" for each intervention	Median (range) aerobic colony count in CFU/cm2 of VRE across surfaces was reduced from 1.5 (0.0 -8.4) before disinfection with NaDCC to undetectable level after disinfection. Median (range) was 0.98 (0.0 – 32.1) before disinfection with sodium hypochlorite compared to 0.0 (0.0 – 0.1) after disinfection. Reductions were not significantly lower with either of the disinfectants.
Al-Hamad 2008 (516)	Gram-positive cocci (*Staphylococcus aureus*-MRSA)	Single site, uncontrolled before-after study, over 4 weeks	49 samples were collected from clinical patient areas (bed frame, cabinet horizontal surface,	Daily cleaning consisted of detergent on floors, door handles, and sinks. Terminal cleaning consisted of detergent followed by hypochlorite (unspecified hypochlorite,	No MRSA detected in ICU samples after cleaning. One sample was positive for MRSA in HDU samples after cleaning.

Reference (Study ID)	Outcome	Study Design	Setting	Intervention	Results
			door handle, monitor and control panel, cabinet handle, soap dispenser, chart table, sink tap handle) from a seven-bedded ICU and five-bedded high dependency unit (HDU) at large district general hospital in the UK. Non-clinical areas were also sampled.	concentration, product, contact time) on surfaces except electrical equipment (70% alcohol). Clinical areas samples were randomly collected before cleaning and immediately after terminal cleaning.	
Oztoprak 2019 (2288)	Gram-positive cocci (*Staphylococcus aureus*-MRSA)	Single-site quasi experimental, controlled before-after study over one month	5 high-touch surfaces (buttons, bedside table, bed rail, floor) from 3 rooms in 43-bed ICU at tertiary care hospital. Turkey	Each of the following disinfectants was used in one of three rooms: steam technology (Tecnovap Evo 304) compared to two-step cleaning with detergent and water on microfiber cloths followed by 1,000 ppm, or 5,000 ppm hypochlorite solution (sodium dichloroisocyanurate) wipes. Known organism concentration inoculated onto pre-cleaned surface. Samples taken 10 minutes after inoculation and after disinfection.	No bacterial growth after steam or hypochlorite disinfection. Initial concentration not reported.
Ho 2016 (6163)	Gram-positive cocci (*Staphylococcus aureus*-MRSA)	Single-site, Quasi-experimental study, controlled before-after study	121 total samples were collected from 11 specific high-touch surfaces in 22 rooms of the hospital environment. Hualien, Taiwan.	Standard daily cleaning (before) with 0.06% sodium hypochlorite detergent disinfectant on microfiber cloths compared to modified daily cleaning (after) with demand-release chlorine sodium dichloroisocyanurate (NaDCC) tablets (Medentech, Wexford, Ireland) on microfiber cloths (concentration of active chlorine equivalent to that of 0.05% NaOCl). Unspecified contact time. Samples measured “before and	Median (range) aerobic colony count in CFU/cm2 of MRSA across surfaces was reduced from 0.9 (0.0-447.3 range) before disinfection with NaDCC to 0.0 (0.0-5.4) after disinfection. Median (range) was 1.1 (0.0 – 50.0) before disinfection with sodium hypochlorite) compared to 0.0 (0.0 – 6.2) after disinfection. Reductions were not significantly lower with either of the disinfectants.

Reference (Study ID)	Outcome	Study Design	Setting	Intervention	Results
				after daily cleaning" for each intervention	
Karpanen 2012 (6414)	Gram-positive cocci (*Staphylococcus aureus*-MRSA)	Single-site, controlled cross-over study over a 24 week period	~672 samples from frequent-touch items (door handles and push plates, toilet seats and flush handles, grab rails, light switches and pull cord toggles, sockets, overbed tables, dressing trolleys, commodes, taps, and sink fittings) in acute care medical ward at a large university hospital. UK.	14 types of copper items were installed 3 months before beginning of the study and include surfaces with copper-alloy (58-99.5% copper). Comparator items were composed of anodized aluminum, steel, plastic, chromium plated brass, etc. Sampled weekly before afternoon cleaning which consisted of detergent, hot water, and 1000 ppm chlorine (sodium dichloroisocyanurate, product unspecified, contact time unspecified). Copper and control items were switched after 12 weeks.	MRSA was found similarly on copper and control surfaces. Odds ratio (95% confidence ratio) of copper surface positive for MRSA compared to control surface was 0.621 (0.306 – 1.262), p=0.217.
Shelly 2011 (10463)	Gram-positive cocci (*Staphylococcus aureus*-MRSA)	Single site, uncontrolled before-after study over 2 weeks	125 environmental and patient equipment samples from high-risk sites in the department of radiology including areas with direct patient contact with equipment (e.g. sink, floor, imaging equipment, MRI, collimators, etc.), areas with high throughput of staff (e.g. conference room, radiographer workstation), and patient transport areas (e.g. conference room,	Initial environmental sampling was conducted as surveillance. When samples were positive, 1000 ppm NaDCC (Chlor-Clean) was applied with long-handled brush to bore of MRI unit. Samples were collected again after 2 weeks of the initial screen and disinfection and again biannually.	The initial screening found that one sample (1/125) was positive for MRSA. The MRI unit was not positive after disinfection.

Reference (Study ID)	Outcome	Study Design	Setting	Intervention	Results
			waiting area, trolley) at a 550-bed tertiary referral hospital in Dublin, Ireland.		
Stewart 2014 (7891)	Gram-positive cocci (*Staphylococcus aureus*-MSSA & MRSA)	Single-site, Quasi-experimental, uncontrolled before-after study, over 4 months	360 total samples collected from 4 sites (lockers, left and right cotsides, overbed tables) in 30 rooms in an acute care elderly ward at 450-bed general hospital. Lanarkshire, UK.	Surfaces were sprayed with 1.5 mL of electrolyzed water (Salvesan, Aqualution, 10-15 s contact time) and subsequent wiping clean with a new detergent wipe (Tuffie detergent wipes; Vernacare) then allowed to dry. Samples were taken before (12-22 h after daily detergent disinfection) and after disinfection: 1, 2, 4, 8, 12, 24, and 48 h after disinfection. Experiment repeated 3 times.	The number of MSSA & MRSA isolates detected across all rooms was 34 before disinfection and reduced to 18 (48% decrease) 1 h after disinfection. It reached a minimum at 4 h (71% reduction) and a maximum at 24 h (155% compared to baseline): 14 at 2 h, 10 at 4 h, 14 at 8 h, 31 at 12 h, 53 at 24 h, and 36 at 48h.
Al-Hamad 2008 (516)	Gram-positive cocci (*Staphylococcus aureus*-MSSA)	Single site, uncontrolled before-after study, over 4 weeks	49 samples were collected from clinical patient areas (bed frame, cabinet horizontal surface, door handle, monitor and control panel, cabinet handle, soap dispenser, chart table, sink tap handle) from a seven-bedded ICU and five-bedded high dependency unit (HDU) at large district general hospital in the UK. Non-clinical areas were also sampled.	Daily cleaning consisted of detergent on floors, door handles, and sinks. Terminal cleaning consisted of detergent followed by hypochlorite (unspecified hypochlorite, concentration, product, contact time) on surfaces except electrical equipment (70% alcohol). Clinical areas samples were randomly collected before cleaning and immediately after terminal cleaning.	In the ICU, MSSA was isolated from 3/10 samples after cleaning compared to 1/10 samples before cleaning. In the HDU, no S. *aureus* was isolated before cleaning , with no MSSA isolated after cleaning.
Karpanen 2012 (6414)	Gram-positive cocci (*Staphylococcus aureus*-MSSA)	Single-site, controlled cross-over study over a	~672 samples from frequent-touch items (door handles and push plates, toilet	14 types of copper items were installed 3 months before beginning of the study and include surfaces with copper-alloy (58-99.5% copper). Comparator items	MSSA was found more often on control surfaces compared to copper surfaces. Odds ratio (95% confidence ratio) of copper surface positive for MSSA

Reference (Study ID)	Outcome	Study Design	Setting	Intervention	Results
		24 week period	seats and flush handles, grab rails, light switches and pull cord toggles, sockets, overbed tables, dressing trolleys, commodes, taps, and sink fittings) in acute care medical ward at a large university hospital. UK.	were composed of anodized aluminum, steel, plastic, chromium plated brass, etc. Sampled weekly before afternoon cleaning which consisted of detergent, hot water, and 1000 ppm chlorine (sodium dichloroisocyanurate, product unspecified, contact time unspecified). Copper and control items were switched after 12 weeks.	compared to control surface was 0.262 (0.112 – 0.612), p=0.001.
Goldenberg 2012 (5957)	HAI (HAI – *C. difficile*)	Single-site quasi experimental, uncontrolled study over 4 months	Patients with positive sample on a monthly basis from 13 wards (general medical, surgery, elderly care, acute admissions, etc.) a hospital, UK	A pre-intervention period with standard cleaning (microfiber system with no chemical disinfectant; 1000 ppm chlorine, Chlor-Clean when in contact with infectious patient) was compared to intervention period with chlorine-dioxide-based disinfection (Difficil-S, 2 min contact time). CDI rates based on central reporting of CDI.	The average number (95% confidence interval) of CDIs was not significantly different between pre-intervention and intervention periods with 11.8 (6.33-17.17) in pre-intervention and 12.7 (8.86 – 18.57) during intervention. Similarly, the average rate of infection (95% CI) was not significantly different with 0.42 (0.23 – 0.62) per 1000 occupied bed-days (OBD) compared to 0.50 (0.31 – 0.69) per 1000 OBD during intervention.
Mayfield 2000 (8380)	HAI (HAI-*Clostridium difficile*)	Single site, quasi-experimental, uncontrolled before-after study for a 20-month period.	All patients in the bone marrow transplantation unit (n=293), the neurosurgical intensive care unit (ICU) (n=1278), and a general medicine unit (n=2881) at Barnes-Jewish Hospital, a 2-campus 1287-bed tertiary care university-affiliated facility. Missouri, USA.	Routine baseline cleaning with quaternary ammonium solution for 9 months (period 1) was switched to routine cleaning with unbuffered 1:10 hypochlorite solution for 9 months (contact time not specified) (period 1) and switched back to routine daily cleaning with a quaternary ammonium solution with a 5-min contact time, followed by vigorous rubbing (period 3)	Among the bone marrow transplant patients (n=293), CDAD incident rate decreased significantly from 8.6 to 3.3 cases per 1000 patient days (period 1 to period 2). No significant reduction in CDAD rates were seen in the other units from period 1 to period 2: neurosurgical ICU (from 3.0 to 2.7 cases per 1000 patient-days) and general medicine unit (from 1.3 to 1.5 cases per 1000 patient-days). CDAD rate for bone marrow transplant patients increased to 8.1 cases from 3.2 cases per 1000 patient –days from period 2 to period 3 after replacing

Reference (Study ID)	Outcome	Study Design	Setting	Intervention	Results
					hypochlorite with quaternary ammonium disinfectant.

Table S16: Study results for other manually applied interventions ordered by outcome organism

Reference (Study ID)	Outcome	Study Design	Setting	Intervention	Results
Dunklin 1959 (463)	All viable organisms	Single site, quasi-experimental, uncontrolled study over 33 days	Samples (unspecified number)taken from the, floor tiles, hall, one recovery room of the Department of Surgery, University of Chicago teaching hospital, USA	Standard daily cleaning with detergent for 13 days was compared to 20 days of 2.5% orthophenylphenol solution (O-Syl; orthophenylphenol, 12%); potassium ricinoleate, 25.8%; ethyl alcohol (denatured), 7.2%; propylene glycol, 7.5%] glycerol, 2.5%; water, 45 %. Manufactured by Lehn and Fink Products Corporation, Bloomfield, New Jersey) applied and allowed to dry. Surfaces were treated at 7 AM and samples were taken within two hours of cleaning as well as 7-8 h later.	An average (range) of 87.4% (70-96%) and 95% (90 – 98%) reduction was observed for two test periods after application of orthophenylphenol solution compared to standard daily cleaning on floors. The range of average concentration after cleaning from detergent (11,100 – 169,800) was higher than after cleaning with orthophenylphenol solution (423-12,280 CFU/ft2. Significance not specified.
Gable 1966 (1073)	All viable organisms	Single site, quasi-experimental, controlled before-after study	100 samples were taken from the floors of an operating room from three wards at the University of Michigan Hospital, USA	The usefulness of phenolic detergent-disinfectant (Mikro-Bac, 1.25 oz/gal) was compared to general purpose cleaner (Soilax, 1 oz/gal) with five different methods of application: wet mopping with wet vacuum pickup (1), wet mopping without wet vacuum pickup (2), sprayer with wet vacuum pickup (3), automatic scrub machine with wet vacuum pickup (4), dry mopping with chemically treated dust-cloth (5). Samples were collected 5 minutes after completion of cleaning.	Methods including wet vacuum pickup were preferable to dry or wet mopping alone. Detergent-disinfectant produced slightly improved results (significance not specified, summary average not provided across corridor, operating room, patient room floors across 5 application methods). Initial concentration before cleaning ranged from 36 – 210 CFU compared to after disinfection with phenolic (4 – 293 CFU) and after cleaning with general (8 – 248 CFU)
Hedin 2010 (1205)	All viable organisms	Single-site, quasi-experimental cohort study with simultaneous control over 3 weeks	12 bedside tables, each sampled once a week for 3 weeks in an Infectious Diseases clinical ward in Falun Hospital, Falun, Sweden; 31 samples were analyzed	Active polymer A-200, polyhexamethylene biguanide (PHMB) combined with a surfactant solution Appeartex (Appeartex AB, Göteborg, Sweden) vs. control (untreated half of each surface). Treatment was applied to one half of each table daily following cleaning of the entire table surface with an alcohol-based disinfectant; sampling occurred one day after treatment, just prior to the next cleaning. Treated half	Median (range) concentration of viable bacteria in Appeartex significantly lower than control for two sample methods. (1) contact agar: 9 (0-58) CFU/50cm^2 vs. 22 (0-348) CFU/50cm^2, P< 0.05; (2) swab rinse: 0 (0-90) CFU/50cm^2 vs. 20 (0-960), CFU/50cm^2 , P<0.05

Reference (Study ID)	Outcome	Study Design	Setting	Intervention	Results
				was cleaned only with water between samplings.	
Hall 2011 (1723)	All viable organisms	Single site, quasi-experimental, controlled crossover study for 12 weeks	Samples (unspecified number) were collected from ten surfaces in the bathroom or patient area (e.g. floor, table, locker top, chair arm) in four wards comprising elderly care and surgical patients at Mayday Healthcare NHS Trust Hospital, UK	Standard daily cleaning with 1,000 ppm chlorine-releasing agent (sodium dichloroisocyanurate, Actichlor Plus) was compared to daily cleaning with ultramicrofiber (UMF) cloths/mops (Vikan Ltd) with or without a copper-based biocide (ICICS Ltd, CuWB50, 300 ppm). Samples were taken one hour before and one hour after cleaning. After 4 weeks, cleaning was interchanged for another 4 weeks.	CuWB50 and standard cleaning significantly reduced concentration; UMF with water only did not significantly reduce bacterial concentration. CuWB50 showed a significant ($p=0.003$) reduction of median concentration from 78 total viable count to 50 total viable count. In multivariate analysis, UMF + CuWB50 showed a 69% reduction of mean concentration ($p<0.001$) and 51% reduction in RLU ($p<0.001$), which was due to the direct and residual effects of CuWB50 (25% reduction, $p=0.001$) and residual effect which lasted for nearly a week (12%, $p=0.001$). Following standard cleaning, there was a significant 47.7% reduction ($p<0.001$) in median count from 100 to 71. Median ATP bioluminescence also had significant reduction from 500 to 250 RLU after standard cleaning.
Ojajärvi 1976 (2228)	All viable organisms	Single site, controlled cohort study over 6 weeks	720 samples were taken from floors, patient rooms, and non-vertical environmental surfaces in infectious disease ward and ICU at the Children's Hospital in Helsinki University Central Hospital, Finland	Disinfectant was applied on the floors of ICUs three times a day and floors of patient rooms in infectious disease ward once a day. Other surfaces were wiped with the disinfectant. Surfaces were not rinsed with water. After two weeks using the disinfectant, samples were taken four hours after cleaning for three consecutive days. Samples were not taken prior to disinfection. Disinfectants included K 644 (1.2% chlorinated trisodium phosphate and potassium bromide), Panasept (0.25% sodium dichlorisocyanurate) and Gevisol (0.5-1%	Higher average concentration was found on patient room floors and other surfaces after disinfection using Givisol (28 CFU/plate) compared to Panasept (17 CFU/plate) and K 644 (15CFU/plate) (significance unspecified). The lowest colony count on patient floors was due to K644 in one ward and due to Panasept in the other.

Reference (Study ID)	Outcome	Study Design	Setting	Intervention	Results
				arylated and halogenated phenols). Contact times not specified.	
Stibich 2011 (2906)	All viable organisms	Single-site, quasi-experimental, uncontrolled before-after study	239 environmental surface samples from 21 high-touch surfaces (bed rails, tray tables, chair arms, telephones, cabinets, intravenous infusion poles, door handles, remote controls, toilet seats, bathroom handrails, and computers) of 12 patient rooms which patients with VRE colonization and/or infection at a large comprehensive cancer center. Texas, USA.	Samples were collected before cleaning (n=73) and after standard terminal cleaning (n=91). Standard terminal cleaning consisted of use of germicide (ortho-phenylphenol 3.4%, ortho-benzyl-para-chlorophenol 3.03%, Wexcide; Wexford Labs) according to hospital guidelines.	Percent (number) surfaces positive 78.1% (57/75) with a mean of 33.0 CFU/cm^2 before and 63.7% (58/91) with a mean of 27.4 CFU/cm^2 after. Mean count was significantly lower after disinfection ($p<0.01$)
Tekin 2013 (4146)	All viable organisms	Single site, quasi-experimental, uncontrolled study	30 samples (operating table, handset, dressing and shower room) were taken from environmental surfaces in the operating room, burn center and clinical microbiology laboratory at Dicle University Hospital. Diyarbakır, Turkey	20% orthophenylphenol (Fumispore, 10% parahidroxifenilsalicilamida) was applied to environmental surfaces. Samples were taken before application and 6 hours after application of Fumispore.	Mean (range) concentration of total live microorganisms was higher at 12.1 (4.2-18.8) CFU/cm^2 before application compared to after at 1.6 (0.4 – 2.7) CFU/cm^2. Significance not specified.
Strat 1971 (5698)	All viable organisms	Single-site, Quasi-experimental, controlled before-after	581 surface samples (operating tables, instruments, walls, floors) from 7 rooms in the Bucharest	A hydrochloride solution (1% Ampholytic detergent with dodecyldiaminoethylglycine; Tego 103G), 1% cationic detergent (cetylpyridinium chloride) (BCP), and alcohol-based disinfectant (triethylene	On average, the efficiency of each disinfectant for decreasing the bacterial load is 90% for BCP (80-98.5%), 95% for Tego 103G (88-99%), 96% for TEG aerosolization and BCP wipes (80-98.5%) and 99% for TEG aerosolization and Tego

Reference (Study ID)	Outcome	Study Design	Setting	Intervention	Results
		study for a 3-day period.	Institute of Hygiene. Bucharest, Romania.	glycol; TEG) were used to simultaneously aerosolize/disinfect air and manually wipe/disinfect surfaces. Samples taken at least 10-15 minutes after cleaning as well as 1.5, 6, and 12 h after cleaning. These values were compared to standard cleaning (water with soda).	103G wipes (98.8-100%) compared to standard cleaning. The efficiency of these disinfectants changed throughout the day as well. After 1.5 h, BCP is 13.8 times more effective than standard cleaning, Tego 103G is 22 times more effective at reducing bacterial count. After 6 h, efficiency lowers with BCP is 3.6 times more effective and Tego 103G is 5.6 times more effective at reducing bacterial load. After 12.5 h, BCP was 5.3 times more effective and Tego 103G is 9.8 times more effective at reducing bacterial load. Bacterial load included *Staphylococcus*, P. *aeruginosa*, *Proteus* mirabilis. *E. coli* were not isolated.
Hamilton 2010 (5832)	All viable organisms	Single site, quasi-experimental crossover study over 7 weeks.	8,190 samples were taken from 10 surfaces (floor, shelf, chair, bed, dispenser) in four wards at Dumfries and Galloway Royal Infirmary, UK	The bactericidal efficiency of ultramicrofiber mops (UMF, Vikan) and water (before) were compared to the bactericidal efficiency of UMF and a 300 ppm copper biocide (CuWB50) (after). Samples were taken three times per week 1 h before cleaning and 1h and 4 h after cleaning. Half of the wards were cleaned with UMF and water for three weeks then UMF and CuWB50 for weeks 4 – 7; vice versa for other half of wards.	Overall, cleaning with UMF and just water decreased the bacterial count by 30% ($p<0.001$) but cleaning with CuWB50 decreased the bacterial count by 56%. Median bacterial count was significantly lower with UMF + CuWB50 compared to UMF + water before, 1 h after, and 4 h after disinfection. The 56% decrease was due to the direct and residual effects of UMF+CuWB50 (20% reduction, $p=0.024$) and residual effect which lasted for nearly two weeks (22% reduction, $p<0.001$).
Daschner 1980 (6651)	All viable organisms	Two site, quasi-experimental, controlled study over 6 months	Samples (unspecified number) from large floor areas in a university clinic, Freiburg, Germany	1% aldehyde disinfectant was used to clean the floor four ways: the two bucket system, a disposable wet wipe, rotating disc blockers and dry mopping. Samples were collected immediately before and 30-120 minutes after disinfection.	Listed from increasing to decreasing efficacy: the two bucket method decreased floor bacterial count by 84% (44,200 CFU/m^2 to 7,100 CFU/m^2), the wet wipe decreased bacterial load by 60% (30,700 CFU/m^2 to 12,500 CFU/m^2), blockers decreased bacteria load by 55% (7,800 CFU/m^2 to 3,500 CFU/m^2) and the dry mop decreased bacterial load by 50% (15,800 CFU/m^2 to 7,900 CFU/m^2)

Reference (Study ID)	Outcome	Study Design	Setting	Intervention	Results
					compared to no disinfection (significance not specified).
Meinke 2012 (8687)	All viable organisms	Single site, quasi-experimental, controlled crossover study over 8 weeks	A total of 3068 samples were collected from tables, faucets, bed control panels and floors in a hematologic transplant unit in University Hospital Basel, Basel, Switzerland	Two disinfectants were compared. The aldehyde based disinfectant (Deconex 50 FF; (12.0 g glyoxal (ethanedial), 0.5 g glutaraldehyde (pentanedial), and 7.5 g didecyldimethylammoniumchloride per 100 g) was used to disinfect half the study unit daily. The glucoprotamin based disinfectant (0.5% Incidin Plus; (26 g glucoprotamin per 100 g) (after) was used to disinfect another half of the study unit for one-hour contact time daily. After 4 weeks, products were switched on wards for another 4 weeks. Sampling was conducted every other day after disinfection. Baseline sampling was not conducted.	9.9% (152/1528) of surfaces disinfected with the aldehyde-based disinfectant showed growth compared to 12.0% (185/1540) of surfaces disinfected with the glucoprotamin-based disinfectant showed growth (p=0.067). The bacterial counts on positive surfaces were also not statistically significant (p=0.58). Baseline sample were not reported/collected.
Tekin 2013 (4146)	Fungi	Single site, quasi-experimental, uncontrolled study	30 samples were taken from environmental surfaces in the operating room, burn center and clinical microbiology laboratory at Dicle University Hospital.	20% orthophenylphenol (Fumispore) was applied to environmental surfaces. Samples were taken before application and 6 hours after application of Fumispore.	Mean concentration for fungi was higher at 2.9 (0.3 – 9.4) CFU/cm2 before application compared to after at 0.4 (0 – 0.9) CFU/cm^2. Significance not specified.
Biswal 2017 (12894)	Fungi (*Candida auris*)	Single site, quasi-experimental, uncontrolled case study, 4 days	Environmental sampling (unspecified number) of surfaces (bed, trolley, ventilator, refrigerator, railing, etc.)in the ICU at the Postgraduate Institute of Medical Education and Research, a tertiary care, multi-	Decontamination of the MICU environment was carried out with 5% phenol (carbolic acid, unspecified trade name/contact time) after *C. auris* had been detected from the environment.	Repeat environmental samples (unspecified number) four days post-disinfection were still positive for C. auris.

Reference (Study ID)	Outcome	Study Design	Setting	Intervention	Results
			specialty hospital in Chandigarh, India		
Exner 1982 (7455)	Fungi (Yeast)	Single site, quasi-experimental study over 9 weeks	186 samples were taken from the floor, shelves, windows and beds in an ICU	As an addition to unspecified standard cleaning, an aldehyde-based disinfectant was sprayed on surfaces of ICU and samples were collected 2 h after disinfection. In series 1, samples were also collected after 1 and 3 weeks. In a second series, samples were collected 1,2,3, and 6 weeks after disinfection.	Surfaces were negative for yeast immediately after disinfection and intermittently positive > 1 week. In series one, the percent of surfaces positive for yeast before disinfection was 1.7% and went down to 0% right after, one week after and three weeks after disinfection. In series two, the percent of surfaces positive for yeast before disinfection was 13.6% and went down to 0% right after. The percent of surfaces positive for yeast went up to 7% one week after and went down to 0% two weeks after and up to 2.6% three and six weeks after disinfection. Significance was not specified.
Meinke 2012 (8687)	Gram-negative bacteria	Single site, quasi-experimental, controlled crossover study over 8 weeks	A total of 3068 samples were collected from tables, faucets, bed control panels and floors in a hematologic transplant unit in University Hospital Basel, Basel, Switzerland	Two disinfectants were compared. The aldehyde based disinfectant (Deconex 50 FF; (12.0 g glyoxal (ethanedial), 0.5 g glutaraldehyde (pentanedial), and 7.5 g didecyldimethylammoniumchloride per 100 g) was used to disinfect half the study unit daily. The glucoprotamin based disinfectant (0.5% Incidin Plus; (26 g glucoprotamin per 100 g) (after) was used to disinfect another half of the study unit for one-hour contact time daily. After 4 weeks, products were switched on wards for another 4 weeks. Sampling was conducted every other day after disinfection. Baseline sampling was not conducted	0 surfaces were positive for gram-negative bacteria from aldehyde-based disinfectant compared to 1 sample positive for glucoprotamin-based disinfectant (floor, typed as *Enterobacter* aerogenes, 6 CFU/100 cm2).
Exner 1982 (7455)	Gram-negative bacteria (*Citrobacter* spp.)	Single site, quasi-experimental study over 9 weeks	186 samples were taken from the floor, shelves, windows and beds in an ICU	As an addition to unspecified standard cleaning, an aldehyde-based disinfectant (unspecified ingredients) was sprayed on surfaces of ICU and samples were collected 2 h after disinfection. In series	Surfaces were negative for *Citrobacter* immediately after disinfection. In series two, the percent of surfaces positive for *Citrobacter* before disinfection was 1% and went down to 0% directly after, one

Reference (Study ID)	Outcome	Study Design	Setting	Intervention	Results
				1, samples were also collected after 1 and 3 weeks. In a second series, samples were collected 1,2,3, and 6 weeks after disinfection.	week, two weeks and three weeks after disinfection. 1% of surfaces were positive for *Citrobacter* six weeks after disinfection. Significance was not specified.
Exner 1982 (7455)	Gram-negative bacteria (*Enterobacter* spp)	Single site, quasi-experimental, uncontrolled study over 9 weeks	186 samples were taken from the floor, shelves, windows and beds in an ICU. Germany	As an addition to unspecified standard cleaning, an aldehyde-based disinfectant (unspecified ingredients) was sprayed on surfaces of ICU and samples were collected 2 h after disinfection. In series 1, samples were also collected after 1 and 3 weeks. In a second series, samples were collected 1,2,3, and 6 weeks after disinfection.	*Enterobacter* was reduced but not eliminated after disinfection. 2.8% of surfaces were positive before disinfection, 5.6% after disinfection, 24% after a week of disinfection and 0% after 3 weeks. In series two, the percent of surfaces positive for before disinfection was 13.6%, 8.0% after disinfection, 19% a week after disinfection, 18.3% two weeks after, 2.6% three weeks after and 20% six weeks after disinfection. Significance not specified.
Exner 1982 (7455)	Gram-negative bacteria (*Escherichia* coli)	Single site, quasi-experimental study over 9 weeks	186 samples were taken from the floor, shelves, windows and beds in an ICU	As an addition to unspecified standard cleaning, an aldehyde-based disinfectant (unspecified ingredients) was sprayed on surfaces of ICU and samples were collected 2 h after disinfection. In series 1, samples were also collected after 1 and 3 weeks. In a second series, samples were collected 1,2,3, and 6 weeks after disinfection.	*E. coli* was not found on surfaces up to 3 weeks after disinfection. In series one, the percent of surfaces positive for *E. coli* before disinfection was 8.4% and 0% were present directly after, one week after and three weeks after disinfection. In series two, percent surfaces positive before disinfection was 13.6% and 0% was present directly after, one week after, two weeks after and three weeks after disinfection. However, there was 6.9% of *E. coli* present six weeks after disinfection. Significance was not specified.
Exner 1982 (7455)	Gram-negative bacteria (*Klebsiella* spp.)	Single site, quasi-experimental study over 9 weeks	186 samples were taken from the floor, shelves, windows and beds in an ICU	As an addition to unspecified standard cleaning, an aldehyde-based disinfectant (unspecified ingredients) was sprayed on surfaces of ICU and samples were collected 2 h after disinfection. In series 1, samples were also collected after 1 and 3 weeks. In a second series, samples were collected 1,2,3, and 6 weeks after disinfection.	Surfaces were negative for *Klebsiella* immediately after disinfection. In series one, the percent of surfaces positive before disinfection was 35% and 0% directly after, one week after and three weeks after disinfection. In series two, the percent of *Klebsiella* present before disinfection was 11%, 0% directly after, less than 1% one week after, 2.6% two weeks after, and 0%

Reference (Study ID)	Outcome	Study Design	Setting	Intervention	Results
					three and six weeks after disinfection. Significance was not specified.
Exner 1982 (7455)	Gram-negative bacteria (*Proteus* spp.)	Single site, quasi-experimental study over 9 weeks	186 samples were taken from the floor, shelves, windows and beds in an ICU	As an addition to unspecified standard cleaning, an aldehyde-based disinfectant (unspecified ingredients) was sprayed on surfaces of ICU and samples were collected 2 h after disinfection. In series 1, samples were also collected after 1 and 3 weeks. In a second series, samples were collected 1,2,3, and 6 weeks after disinfection.	Surfaces were negative for *Proteus* immediately after disinfection. In series one, the percent of surfaces positive for *Proteus* before disinfection was 1.7% and 0% directly after, one week after and three weeks after disinfection. In series two, the percent of surfaces positive for *Proteus* before disinfection was 1.7% and 0% directly after and one week after disinfection. There was 1.7% *Proteus* present two, three and six weeks after disinfection. Significance was not specified.
Exner 1982 (7455)	Gram-negative bacteria (*Pseudomonas aeruginosa*)	Single site, quasi-experimental study over 9 weeks	186 samples were taken from the floor, shelves, windows and beds in an ICU	As an addition to unspecified standard cleaning, an aldehyde-based disinfectant (unspecified ingredients) was sprayed on surfaces of ICU and samples were collected 2 h after disinfection. In series 1, samples were also collected after 1 and 3 weeks. In a second series, samples were collected 1,2,3, and 6 weeks after disinfection.	Surfaces were negative for P. *aeruginosa* immediately after disinfection. In series two, the percent of surfaces positive for P. *aeruginosa* before disinfection was 2.5% and went down to 0% directly after and one week after disinfection. There were 1.7% of surfaces positive for P. *aeruginosa* two and three weeks after disinfection. There were 9.5% of surfaces positive for P. *aeruginosa* six weeks after disinfection. Significance was not specified.
Meinke 2012 (8687)	Gram-positive bacilli (*Clostridium difficile*)	Single site, quasi-experimental, controlled crossover study over 8 weeks	A total of 3068 samples were collected from tables, faucets, bed control panels and floors in a hematologic transplant unit in University Hospital Basel, Basel, Switzerland	Two disinfectants were compared. The aldehyde based disinfectant (Deconex 50 FF; (12.0 g glyoxal (ethanedial), 0.5 g glutaraldehyde (pentanedial), and 7.5 g didecyldimethylammoniumchloride per 100 g) was used to disinfect half the study unit daily. The glucoprotamin based disinfectant (0.5% Incidin Plus; (26 g glucoprotamin per 100 g) (after) was used to disinfect another half of the study unit for one-hour contact time daily. After 4 weeks, products were switched on wards	*C. difficile* was not detected in the study, though there were clinical cases caused by this organism.

Reference (Study ID)	Outcome	Study Design	Setting	Intervention	Results
				for another 4 weeks. Sampling was conducted every other day after disinfection. Baseline sampling was not conducted.	
Exner 1982 (7455)	Gram-positive bacilli (Spore-forming)	Single site, quasi-experimental study over 9 weeks	186 samples were taken from the floor, shelves, windows and beds in an ICU	As an addition to unspecified standard cleaning, an aldehyde-based disinfectant was sprayed on surfaces of ICU and samples were collected 2 h after disinfection. In series 1, samples were also collected after 1 and 3 weeks. In a second series, samples were collected 1,2,3, and 6 weeks after disinfection.	The disinfection did not affect spore-forming bacteria and was detected at every time point. In series one, the % surfaces with spore-forming bacteria before disinfection was 19.7%, 25.3% after disinfection, 4.2% after 1 week, and 52.1% after 3 weeks. In the second series, surfaces positive before disinfection was 40.6%, 15.2% after disinfection, 52% one week after, 19.1% two weeks after, 18.3% three weeks after and 41.7% six weeks after disinfection. Significance was not specified.
Hedin 2010 (1205)	Gram-positive cocci (*Enterococcus faecalis*)	Single-site, quasi-experimental cohort study with simultaneous control over 3 weeks	12 bedside tables, sampled 3 times each, in an Infectious Diseases clinical ward in Falun Hospital, Falun, Sweden; 31 samples were analyzed	Active polymer A-200, polyhexamethylene biguanide (PHMB) combined with a surfactant solution Appeartex (Appeartex AB, Göteborg, Sweden) vs. control (untreated half of each surface). Treatment was applied to one half of each table daily following cleaning of the entire table surface with an alcohol-based disinfectant; sampling occurred one day after treatment, just prior to the next cleaning. Treated half was cleaned only with water between samplings.	Two (10 CFU/50 cm2 each) Appeartex-treated tables positive for *E. faecalis* vs. three (two tables w/10 CFU/50cm and one table w/220 CFU/50 cm2) untreated tables (P>0.05); 97 isolates of *S. aureus* from 635 treated surfaces
Smith 1998 (2864)	Gram-positive cocci (*Enterococcus* spp.- Vancomycin-resistant enterococci (VRE))	Single site, quasi-experimental, uncontrolled study	Samples (unknown) were taken from surfaces (treatment chair, table and sink handles) in seven cancer clinic examination rooms with VRE-positive	After superficial cleaning by spraying phenolic disinfectant (1:256 solution of Lilaphene in water, 5.5% potassium orthophenylphenate, 2.2% potassium 4-chloro-2-cyclopentylphenate, 2.2% potassium para-tertiary-amylphenate) on surfaces and wiping immediately with a paper towel (2 minutes) was compared to	No clinic room had VRE prior to patient entry. Before cleaning and after patient examination, 29% (2/7) clinic rooms had a surface positive for VRE. After superficial cleaning, one of the rooms was still contaminated with VRE and after deep cleaning, no rooms were found to

Reference (Study ID)	Outcome	Study Design	Setting	Intervention	Results
			patients at outpatient clinics. Nebraska, USA	the same solution used for deep cleaning by scrubbing the surfaces rigorously (20 minutes). Samples were collected before patient entry, after patient exit, after superficial cleaning, and after deep cleaning.	contaminated with VRE. Significance not specified.
Stibich 2011 (2906)	Gram-positive cocci (*Enterococcus* spp.- Vancomycin-resistant enterococci (VRE))	Single-site, quasi-experimental, uncontrolled before-after study	239 environmental surface samples from 21 high-touch surfaces (bed rails, tray tables, chair arms, telephones, cabinets, intravenous infusion poles, door handles, remote controls, toilet seats, bathroom handrails, and computers) of 12 patient rooms which patients with VRE colonization and/or infection at a large comprehensive cancer center. Texas, USA.	Samples were collected before cleaning (n=73) and after standard terminal cleaning (n=91). Standard terminal cleaning consisted of use of germicide (ortho-phenylphenol 3.4%, ortho-benzyl-para-chlorophenol 3.03%, Wexcide; Wexford Labs) according to hospital guidelines.	Percent (number) surfaces VRE positive was 23.3% (17/75) before and 8.2% (4/91) after. Significance not assessed.
Meinke 2012 (8687)	Gram-positive cocci (*Enterococcus* spp., VRE)	Single site, quasi-experimental, controlled crossover study over 8 weeks	A total of 3068 samples were collected from tables, faucets, bed control panels and floors in a hematologic transplant unit in University Hospital Basel, Basel, Switzerland	Two disinfectants were compared. The aldehyde based disinfectant (Deconex 50 FF; (12.0 g glyoxal (ethanedial), 0.5 g glutaraldehyde (pentanedial), and 7.5 g didecyldimethylammoniumchloride per 100 g) was used to disinfect half the study unit daily. The glucoprotamin based disinfectant (0.5% Incidin Plus; (26 g glucoprotamin per 100 g) (after) was used to disinfect another half of the study unit for one-hour contact time daily. After 4 weeks, products were switched on wards for another 4 weeks. Sampling was conducted every other day after	Enterococci (*E. faecalis*, *E. faecium*) was similarly (p=0.14) detected in 3% of surfaces disinfected with glucoprotamin-based disinfectant and 6% of samples disinfected with aldehyde-based disinfectant. High concentrations were reported (up to 8000 CFU/10cm2) 1 h after disinfection. VRE were not detected.

Reference (Study ID)	Outcome	Study Design	Setting	Intervention	Results
				disinfection. Baseline sampling was not conducted.	
Exner 1982 (7455)	Gram-positive cocci (Green *Streptococcus*)	Single site, quasi-experimental study over 9 weeks	186 samples were taken from the floor, shelves, windows and beds in an ICU	As an addition to unspecified standard cleaning, an aldehyde-based disinfectant was sprayed on surfaces of ICU and samples were collected 2 h after disinfection. In series 1, samples were also collected after 1 and 3 weeks. In a second series, samples were collected 1,2,3, and 6 weeks after disinfection.	Before disinfection, 1% surfaces were positive compared to 0 immediately after, 1 week after, and 2 weeks after. 4.3% surfaces were positive 3 weeks after and 0% positive 6 weeks after. Significance was not specified.
Oie 2005 (11015)	Gram-positive cocci (*Staphylococcus aureus*-MRSA)	Single-site, quasi-experimental, controlled before-after study. 3-month period.	32 samples were taken from smooth, non-porous surfaces (immersion bathtub, foot washbowl, examination tables) in a 37-bed dermatological ward in the hydrotherapy unit and ointment treatment unit of a university hospital. Yamaguchi, Japan.	Baseline sampling before disinfection was compared to sampling non-porous surfaces after two disinfection methods: wiping with 0.2% solution (alkyldiaminoethyl glycine, Tego-51) or wiping with 80% ethyl alcohol (Kenei Pharm). Contact time for Tego-51 was 10 min; unspecified contact time for alcohol. This study compared efficacy among porous and non-porous surfaces.	Mean MRSA count before disinfection ranged from 48 (119) to 7366 (16555) to no detection after Tego51 or ethyl alcohol disinfection.
Ogino 1995 (11022)	Gram-positive cocci (*Staphylococcus aureus*-MRSA)	Single site, quasi-experimental, uncontrolled study over three days.	Samples (unknown number) were taken from the floors of four rooms with patients positive for MRSA in Yamanashi Medical University Hospital, Japan	The bactericidal effect of 500 ppm disinfectant made from grapefruit seeds (DF-100, Puri Dyne) was observed. Samples were taken before and one hour after disinfection for three days.	9/12 sites were positive prior to disinfection compared to 6/12 sites positive after disinfection. Qualitative concentration reported as high (>100 CFU), medium (59-99 CFU), low (1-49 CFU) and negative. Of the 9 sites positive before disinfection, 4 sites had complete removal of MRSA with initial low concentration. 2 sites already had low concentration and showed no change after disinfection. 3 sites had reduced but not complete removal of MRSA concentration after disinfection. Significance not specified.

Reference (Study ID)	Outcome	Study Design	Setting	Intervention	Results
Fujii 1996 (11965)	Gram-positive cocci (*Staphylococcus aureus*-MRSA)	Single-site, quasi-experimental, controlled before-after study.	An unstated number of samples were taken from the floors of patient rooms in a neurosurgery ward at a university hospital. Yamaguchi, Japan.	Baseline measurements were taken prior to mopping. Floors were mopped with QAC benzalkonium chloride (Osvan) in concentrations of 0.2% and 0.5%. Product was compared to hydrochloride solution of alkyldiaminoethyl glycine (zwitterionic surfactant; Tego-51) at 0.2% concentration, as well as chlorhexidine digluconate (Hibitane) in concentrations of 0.2% and 0.5%. This was a cohort study and no products were designated as the control. Contact time, time until measurement after disinfection were not specified.	MRSA was detected following disinfection with alkyldiaminioethyl glycine, 0.2% chlorhexidine digluconate, and 0.2% benzalkonium chloride; it was not detected when concentration was increased to 0.5% benzalkonium chloride and 0.5% chlorhexidine digluconate (significance not specified).
Oie 2005 (11015)	Gram-positive cocci (*Staphylococcus aureus*-MSSA)	Single-site, quasi-experimental, controlled before-after study. 3-month period.	32 samples were taken from smooth, non-porous surfaces (immersion bathtub, foot washbowl, examination tables) in a 37-bed dermatological ward in the hydrotherapy unit and ointment treatment unit of a university hospital. Yamaguchi, Japan.	Baseline sampling before disinfection was compared to sampling non-porous surfaces after two disinfection methods: wiping with 0.2% solution (alkyldiaminoethyl glycine, Tego-51) or wiping with 80% ethyl alcohol (Kenei Pharm). Contact time for Tego-51 was 10 min; unspecified contact time for alcohol. This study compared efficacy among porous and non-porous surfaces.	Mean (standard deviation) MSSA bacterial count before disinfection ranged from 6.5 (16) to 13897 (37721) to no detection after Tego51 or ethyl alcohol disinfection.
Exner 1982 (7455)	Gram-positive cocci (*Staphylococcus aureus*)	Single site, quasi-experimental study over 9 weeks	186 samples were taken from the floor, shelves, windows and beds in an ICU	As an addition to unspecified standard cleaning, an aldehyde-based disinfectant was sprayed on surfaces of ICU and samples were collected 2 h after disinfection. In series 1, samples were also collected after 1 and 3 weeks. In a second series, samples were collected 1,2,3, and 6 weeks after disinfection.	S. *aureus* positive was reduced but not eliminated after disinfection. In series one, the percent surfaces positive for S. *aureus* before disinfection was 56% and went down to 5.6% directly after, 4.2% one week after and up to 8.4% three weeks after disinfection. In series two, % surfaces positive before disinfection was 5.1% and it went down to 0% right after disinfection. There was 4.3% S. *aureus* present one week after disinfection, 3.5%

Reference (Study ID)	Outcome	Study Design	Setting	Intervention	Results
					S. *aureus* two and three weeks after disinfection and 6.0% S. *aureus* six weeks after disinfection. Significance was not specified.
Meinke 2012 (8687)	Gram-positive cocci (*Staphylococcus aureus*)	Single site, quasi-experimental, controlled crossover study over 8 weeks	A total of 3068 samples were collected from tables, faucets, bed control panels and floors in a hematologic transplant unit in University Hospital Basel, Basel, Switzerland	Two disinfectants were compared. The aldehyde based disinfectant (Deconex 50 FF; (12.0 g glyoxal (ethanedial), 0.5 g glutaraldehyde (pentanedial), and 7.5 g didecyldimethylammoniumchloride per 100 g) was used to disinfect half the study unit daily. The glucoprotamin based disinfectant (0.5% Incidin Plus; (26 g glucoprotamin per 100 g) (after) was used to disinfect another half of the study unit for one-hour contact time daily. After 4 weeks, products were switched on wards for another 4 weeks. Sampling was conducted every other day after disinfection. Baseline sampling was not conducted.	Two floor surfaces were positive for S. *aureus* from aldehyde-based disinfectant compared to 2 floor surfaces, 2 bed control panel surfaces and 1 water faucet surface positive for glucoprotamin-based disinfectant.
Daschner 1980 (6651)	HAI (HAI-All viable bacteria)	Two site, quasi-experimental study over 6 months	Samples (unspecified number) from large floor areas in a university clinic, Freiburg, Germany	For 6 months, 0.5% aldehyde disinfectant was applied three times a day at the medical, surgical and neurosurgical ICU floors and the amount of bacterial infections were observed as compared to no disinfection in the prior 6 months	No significant difference was seen in hospital infections when infections from time period with disinfection (15.6% patients with nosocomial infection) compared to period without disinfection (15.5%).
Danforth 1987 (313)	HAI (HAI-all viable bacteria)	Single site, quasi-experimental, controlled crossover study over 6 months	442 of 5,883 patients discharged with nosocomial infection among eight acute care (medical, surgical, pediatric) wards in a 930-bed tertiary care teaching hospital, Calgary, Canada	Two cleaning agents applied to floors were compared: soap detergent (monoethanolamine, sodium trisolyphosphate, triethanolude and coconut aekansiamide soap; Power-solv) (diluted 20:1) was compared to a disinfection agent (stabilized chlorinated phenol germicidal cleaning agent with ortho-benzyl parachlorophenol; Biofex). Every ward was randomly chosen to have either the soap or disinfection agent applied for three months. Then the	No difference in infection rate (p>0.05) during disinfectant use (8.0 per 100 patient discharges) compared to the soap cleaning (7.1 per 100 patient discharges).

Reference (Study ID)	Outcome	Study Design	Setting	Intervention	Results
				intervention was switched for the second three months. Samples were taken at the end of each period (3 months, 6 months) 4 h after floor cleaning.	

Antimicrobial Surfaces and Coatings

Table S17: Study results for copper antimicrobial surface interventions ordered by outcome organism

Reference (Study ID)	Outcome	Study Design	Setting	Intervention	Results
Casey 2010 (125)	All viable organisms	Single-site, controlled crossover study design over a 10 week period	400 samples from 5 high-touch surfaces (door pushplate, faucet handles, toilet seat) from an open ward and patient washrooms of an acute medical ward in a university hospital. Queen Elizabeth Hospital, Birmingham, UK	Coated surfaces were compared to equivalent items with plastic, chrome-plated, or aluminum surfaces. Coated surfaces were pure copper/resin composite (~70% copper) toilet seat; brass (~60 Cu) faucet handles; brass (~70% Cu) door pushplate. Standard cleaning product used was Chlor-clean (sodium dichloroisocyanurate with 1000 ppm available chlorine and detergent). All surfaces disinfected 4 times every 2 h. Samples were collected once a week before daily cleaning began and after daily cleaning ended. Copper-containing and non-copper containing items were interchanged after 5 weeks.	Median numbers of microorganisms on copper surfaces were 90% to 100% lower than their control equivalents. Initial concentration not reported. The range of median total aerobic CFU/cm2 for copper-containing surfaces was 0 – 2.1 with maximum of 38.4 compared to 0.6 – 87.6 with maximum of 266.4 for control surfaces. All copper surfaces had significantly lower concentration compared to control surfaces ($p<0.05$) except for one of the comparisons. 50% of control sample points and 0% of copper points had median counts > 5 CFU/cm2.
Coppin 2017 (280)	All viable organisms	Single-site, quasi-experimental, controlled cohort study design with simultaneous control over a 2 day period	132 samples were taken from bedside tables 22 patient rooms (half isolation, half non-isolation rooms) at a120- bed veterans affairs hospital. Texas, USA.	Copper oxide (SSSCu) impregnated bedside tray tables (EOS^{Cu} Surfaces LLC) and non-copper tray tables were placed in 11 occupied patient rooms. All tables were cleaned with 10% sodium hypochlorite wipes (Clorox Healthcare) immediately prior to sampling. Samples were collected three times per day 0, 3, 6, 24, 27, and 30 h after cleaning.	There was no statistically significant difference in bacterial count between the copper and the non-copper surfaces at hours 0, 3, and 6. However, at hours 24, 27, and 30, there was a statistically higher concentration in non-copper sites compared to copper sites ($p = 0.002$). At hour 0, mean count (95% CI) was lower at 0.2 CFU/25 cm^2 (0-0.4) on copper surfaces compared to 1.9 CFU/25 cm^2 (0 – 4.9) on non-copper surfaces. At hour 30, mean count (95% CI) was lower at 18.9 CFU/25 cm^2 (11.0 – 32.5) on copper surfaces compared to 98.2 CFU/25 cm^2 (56.2-176.3) on non-copper surfaces.
Hinsa-Leasure 2016 (1245)	All viable organisms	Single-site, quasi-experimental, controlled	A total of 665 samples were taken from 20 high-touch surfaces (outside	Baseline samples were taken for 10 weeks prior to installation of copper surfaces. Afterward, samples were taken weekly (time until measurement relative	Mean (median) concentration during 12-month intervention period for control components was significantly higher at 6,172 CFU/100 cm^2 (364) compared to

Reference (Study ID)	Outcome	Study Design	Setting	Intervention	Results
		cohort study over a 12-month period	patient rooms: sinks and faucet handles, keyboards, door opener push plates, toilet flush lever, grab bars, door handles, light switches, bed tables, bed rails) in 18 occupied and unoccupied patient rooms from the medical-surgical suite of a 49-bed rural hospital in Iowa, USA	to cleaning unspecified). Routine cleaning was comprised of a low-alcohol QAC (10-30% dicapryl/dicaprylyl dimonium chloride, 5-10% alkyl dimethylbenzyl ammonium chloride, 1-5% alcohol, and 1-5% tetrasodium EDTA; High Dilution Disinfectant 256; Spartan Green Solutions) at an unspecified final dilution. Routine cleaning in control rooms was compared to rooms with a copper nickel alloy (C706; trial arm) that contained 90% copper by weight for surfaces, plus daily and terminal cleaning with product (OxivirTB) containing benzyl alcohol (1-5%), hydrogen peroxide (<1%), and dodecylbenzene sulfonic acid (<1%). Rooms previously housed by patients with *C. difficile*, in both arms of the study, were subject to cleaning with 0.65% bleach product (Clorox Bleach Germicidal Cleaner; Clorox). Application matrix, wet contact time not specified.	rooms with copper components at 117 CFU/100 cm^2 (0) After routine cleaning, 59% of samples fell below recommended 250 CFU/100 cm^2 threshold in control rooms near patients compared to 91% in rooms with copper.
Schmidt 2019 (2653)	All viable organisms	Single-site, uncontrolled before-after study over a 23 month period	558 samples from 5 high-touch surfaces on occupied beds (bed rail, inside patient facing surface of bed rail, elevation control panel, bed rail lift, and footboard) in the medical ICU at a 62-bed acute care hospital. Indiana, USA.	Control beds were polypropylene (Hill-Rom TotalCare SpO2RT) were monitored for 15 months. The intervention introduced modified control beds such that 100% of the near-patient bed surfaces were encapsulated with antimicrobial copper material (LuminOre CopperTouch) and monitored for 9 months. Daily cleaning conducted with QAC was conducted between 7AM and 11 AM. Samples were collected between 11AM and 2PM.	Overall cumulative average count on copper beds was 94% lower than control beds and had significantly lower concentration of bacteria (p<0.0001) than control beds. Copper surfaces had significantly lower (p<0.001) bacterial count than control surfaces regardless of length of patient stay (< 5 days, 5-10 days or > 10 days).

Reference (Study ID)	Outcome	Study Design	Setting	Intervention	Results
Schmidt 2012 (2654)	All viable organisms	Multisite, quasi-experimental, controlled before-after study over a 43 month period	6 frequent-touch surfaces (side rails of patient bed, over-bed tray table, IV pole, arm rest of chair, call button, computer) in 16 ICU rooms at 3 hospitals: 660-bed academic facility, 432-bed cancer hospital, 98-bed veterans hospital. New York and South Carolina, USA.	At month 23 of 43, copper-alloy surfaces were installed onto six frequent-touch surfaces in half of rooms. The other half of rooms had control surfaces that were not copper-alloy (e.g. wood, plastic, stainless steel). Samples were collected at the same time each week, however unspecified relative to cleaning schedule.	Microbial count was significantly lower for both control and copper surfaces compared to pre-intervention. Copper surfaces had 83% reduction ($p<0.0001$) in average microbial count at 465 CFU/100 cm^2 compared to control surfaces at 2,674 CFU/100 cm^2. Majority of the microorganisms were *Staphylococcus* spp.
Schmidt 2016 (2656)	All viable organisms	Single-site, quasi-experimental, controlled before-after study over a 12 month period	1320 samples from 3 frequent-touch surfaces (bed rails, cradle rails, and faucet handles) in 16 rooms of hospital pediatric intensive care unit (PICU) or intermediate pediatric care unit (PIMCU) at 249-bed tertiary hospital. Santiago, Chile.	Rooms had items with or without copper alloyed surfaces (bed rails, IV pole, sink handles, and work surface). Copper alloys used to surface the objects were brass (C27200 and C23000, DUAM S.A.; Chase Brass) or Eco Brass (C69300). Samples were collected prior to the intervention and then once every two weeks (not specified relative to cleaning schedule).	The microbial burden on copper alloyed surfaces was significantly lower than on the control surfaces by 88% (log10 reduction 1.94; $p<0.0001$). Compared to before the intervention, there was a 99% reduction in microbial burden (log10 reduction 2.00) on bedrails in copper rooms. In control rooms, there was a 73% reduction in CFUs (log10 1.86) on bedrails, before vs after intervention
Schmidt 2013 (4655)	All viable organisms	Single-site, quasi-experimental, controlled cohort over a 3 month period	Three patient-occupied beds with plastic rails (controls) and 3 with copper rails were sampled on each occasion,	Occupied patient beds were compared. Three beds were fitted with copper surface caps on rails (UNS# C110, 99.9% metallic copper). Control beds had plastic bedrail covers. All surfaces were cleaned at least daily using QAC (Virex II 256, 0.07% n-alkyl dimethyl	Initial mean bacterial burden prior to cleaning significantly lower ($p=0.006$) on copper compared to control bedrails. Mean count on copper rails remained significantly lower than that on plastic rails at hours 0.5 ($p=0.069$), 2.5 ($p=0.012$), and 6.5 ($p=0.002$). There was an immediate

Reference (Study ID)	Outcome	Study Design	Setting	Intervention	Results
			resulting in evaluation of 30 beds at 660- bed academic hospital. South Carolina, USA.	benzyl ammonium chloride, 0.07% dodecyl dimethyl ammonium chloride). Samples were collected immediately before cleaning, 30 min, 2.5 h, 4.5, and 6.5 h after cleaning.	decrease in bacterial burden following cleaning regardless of bed rail surface. Mean reduction was 82% for control surfaces compared to 48% for copper surfaces. Percent reduction for control vs. copper surfaces after 0.5 h was 82% vs. 48% ($p=0.069$), after 2.5 h was 74% to 24% ($p=0.012$), after 4.5 h was 61% to 68% ($p=0.013$) and after 6.5 h 15% to 38% ($p=0.002$) respectively.
Marais 2010 (5106)	All viable organisms	Single-site, quasi-experimental controlled cohort study over a six month period	12 high-touch and low touch surfaces (desk, trolleys, top of cupboard, windowsill) in two patient rooms at a primary healthcare clinic. Grabouw, Western Cape, South Africa.	In two identical, adjacent rooms, one was fitted with copper sheets (BS 2870, Alloy C101: 99.9% pure copper) on surfaces and the other was the control with original surfaces (wood, stainless steel, etc.). Samples were collected before cleaning, up to 1 h post-cleaning, and ~8 h post-cleaning. Cleaning consisted of liquid dish soap without disinfectant.	There was a 71% reduction and significantly lower mean total colony counts ($p < 0$ 0.001) for all copper surfaces compared to control surfaces with mean total colony count 5.9 x 10^4 CFU/dm^2 on copper surfaces compared to 2.0 x 10^5 CFU/dm^2 on control surfaces.
Karpanen 2012 (6414)	All viable organisms	Single-site, controlled cross-over study over a 24 week period	~672 samples from frequent-touch items (door handles and push plates, toilet seats and flush handles, grab rails, light switches and pull cord toggles, sockets, overbed tables, dressing trolleys, commodes, taps, and sink fittings) in acute care medical ward at a large university hospital. UK.	14 types of copper items were installed 3 months before beginning of the study and include surfaces with copper-alloy (58-99.5% copper). Comparator items were composed of anodized aluminum, steel, plastic, chromium plated brass, etc. Sampled weekly before afternoon cleaning which consisted of detergent, hot water, and 1000 ppm chlorine (sodium dichloroisocyanurate, product unspecified, contact time unspecified). Copper and control items were switched after 12 weeks.	Eight of 14 item types had significantly lower CFU counts on the copper surfaces than on the standard materials ($p<0.0001$). The other six items had reduced (but insignificant) microbial counts on copper surfaces compared to control. The largest median difference in total aerobic microbial load on copper vs standard door pull handles was 80.3 CFU/cm^2 on toilet flush lever handles from ~110 CFU/cm^2 to ~25 CFU/cm^2

Reference (Study ID)	Outcome	Study Design	Setting	Intervention	Results
Esolen 2018 (7468)	All viable organisms	Single-site, quasi-experimental, controlled cohort design over a 15 day period	272 samples from bedrail covers from 34 beds at the medical–surgical intensive care unit at 550-bed tertiary care academic hospital. Pennsylvania, USA.	Disposable bedrail covers (Aionx Inc.) made of a copper and silver polymer were applied to intervention arm beds. In the control arm, bedrails were made of standard polypropylene. Samples were obtained on days 0, 1, 2, 3, 4, 5, 10, and 15 after application of copper bedrail covers. Daily cleaning protocols were not specified.	Total bacterial count over 15 days was significantly higher ($p<0.001$) at 54,480 CFU/100cm^2 on control bedrails compared to 9,540 CFU/100cm^2 found on copper bedrails. Initial concentration at day 0 on control bedrails was 10,590 CFU/100cm^2 compared to 1,750 CFU/100cm^2 on study bedrails. On day 15, concentration was 1,990 CFU/100cm^2 on control bedrails compared to 950 CFU/100cm^2 on copper bedrails. Both control and copper bedrails had significant reduction over time ($p<0.001$).
Souli 2017 (7928)	All viable organisms	Single-site, controlled, crossover study over a 16 month period	685 samples from surfaces (bed rails, side table, IV pole, side cart, etc.) in two rooms of ICU at 650-bed tertiary care academic hospital. Athens, Greece.	Intervention period 1: Copper-alloy-coated bed and accessories (Hellenic Copper Development Institute) were placed next to non-coated bed and accessories in the same ICU compartment for 5 months. Intervention period 2: copper and non-copper items were placed in separate ICU compartments for 9 months. Cleaning protocol used spray alcohol-based disinfectant (Bacillol) and twice daily cleaning of floors with chlorine solution (1,000 ppm/L. Samples were collected prior to daily cleaning.	Copper coating significantly reduced the percent of surfaces positive when compared to control (55.6% vs 72.5%; $p< 0.0001$). Copper coating also had significantly lower ($p=0.008$) mean bacterial count (standard deviation) with 2,858 CFU/100 cm^2 (8,662) compared to 7,631 CFU/100 cm^2 (30,642).
Rai 2012 (8312)	All viable organisms	Single-site, quasi-experimental controlled cohort study, over 15-week period	194 samples were collected from high-touch surfaces (arm tops, arm sides, and tray tops) of phlebotomy chairs at outpatient infectious disease clinic in South Carolina, USA.	Solid copper alloy metal (90% copper, 10% nickel) was inlaid on phlebotomy chair surfaces. Non-copper, wood/composite phlebotomy chairs were the control. All chairs received daily standard cleaning with QAC wipe (PDI Sani-Cloth) at end of day. Samples were taken twice per week in midafternoon (prior to cleaning).	An 88%-90% ($p<0.0001$) median reduction was observed for the total aerobic bacteria on copper surfaces compared to non-copper surfaces. Median bacterial count was between 1290-1305 CFU/100cm^2 on non-copper components compared to 135-150 CFU/100cm^2 on copper components. The percent of surfaces with <2.5 CFU/cm^2 was 62% of copper compared to 10% of non-copper surfaces. Majority of isolated organisms were staphylococci.

Reference (Study ID)	Outcome	Study Design	Setting	Intervention	Results
Inkinen 2017 (8890)	All viable organisms	Single-site, quasi-experimental, controlled cohort study over a 2 month period	42 samples from toilet surfaces (flush button, support rail) in 8 rooms at a hospital. Satakunta, Finland.	Copper and copper alloy touch surface products (99.90% Cu-DHP) were used on the treatment surfaces. The control group was plastic toilet buttons and chromed support rails. Samples were collected weekly before daily cleaning.	Average (standard deviation) bacterial count was lower on copper flush buttons and support rails compared to control: 5 CFU/cm^2 (6) for copper flush buttons compared to 73 CFU/cm^2 (190) control; 0.1 CFU/cm^2 (0.2) copper support rails compared to 2 CFU/cm^2 (3) compared to control. Significance was not specified
Salgado 2013 (11135)	All viable organisms	Multi-site, quasi-experimental, controlled cohort study over 11 month period	Six high-touch surfaces (bed rails, overbed tables, IV poles, arms of visitor's chair, nurses call button, computer mouse, etc.) with high burden per room in 8 rooms of ICU at 660-bed tertiary care academic hospital, 460-bed academic cancer hospital, and a 98-bed veterans affairs hospital. South Carolina, USA.	Copper alloy surfaces were placed onto 6 common, highly touched objects in ICU rooms. The control rooms were adjacent to copper rooms and used QAC (Virex 256) for routine (at least daily) and terminal cleaning as well as hypochlorite disinfectant (Dispatch) for rooms housing patients with C difficile. Samples were collected weekly (unspecified time relative to routine cleaning).	17% of total bacterial burden was recovered from rooms with copper objects which corresponds to a 0.76 log reduction of total bacteria recovered, copper vs control ($p<0.001$).
Montero 2019 (13703)	All viable organisms	Single-site, quasi-experimental, controlled before-after study over 2-month period	158 samples were collected from 4 high-touch surfaces (bed rails, overbed table, bedside table and IV pole) in two patient rooms in an adult intensive care unit at university-affiliated hospital. Santiago, Chile.	Surfaces were coated with 60% copper, 40% agglomerate resin (Copper Armour™, copper particles in a methyl methacrylate resin DEGADUR 527, Evonik). Uncoated surfaces from adjacent patient room was the control. Samples were collected at the same time and before morning cleaning every week.	Copper surfaces had significantly lower number of surfaces > 250 CFU/100 cm^2 compared to control surfaces ($p<0.001$). Average concentration ranged from 157.5 to 1793 CFU/100 cm^2 on copper-coated surfaces compared to 337.5 – 3323 CFU/100cm^2 on control surfaces. There was a 66% reduction of aerobic microbial burden on copper-coated bed rails ($p=0.018$), 56.5% reduction for the overbed table ($p=0.045$), 14.9% reduction for bedside table ($p=0.303$), 53.5% reduction

Reference (Study ID)	Outcome	Study Design	Setting	Intervention	Results
					on the IV pole (p=0.195) when compared to control.
Montero 2019 (13703)	Fungi	Single-site, quasi-experimental, controlled before-after study over 2-month period	158 samples were collected from 4 high-touch surfaces (bed rails, overbed table, bedside table and IV pole) in two patient rooms in an adult intensive care unit at university-affiliated hospital. Santiago, Chile.	Surfaces were coated with 60% copper, 40% agglomerate resin (Copper Armour™, copper particles in a methyl methacrylate resin DEGADUR 527, Evonik). Uncoated surfaces from adjacent patient room was the control. Samples were collected at the same time and before morning cleaning every week.	There was not a reduction in the average burden of yeast/fungi on coated (median=0 CFU/100 cm2) compared to uncoated surfaces (median= 0 CFU/100 cm2).
Souli 2017 (7928)	Gram-negative bacteria	Single-site, controlled, crossover study over a 16 month period	685 samples from surfaces (bed rails, side table, IV pole, side cart, etc.) in two rooms of ICU at 650-bed tertiary care academic hospital. Athens, Greece.	Intervention period 1: Copper-alloy-coated bed and accessories (Hellenic Copper Development Institute) were placed next to non-coated bed and accessories in the same ICU compartment for 5 months. Intervention period 2: copper and non-copper items were placed in separate ICU compartments for 9 months. Cleaning protocol used spray alcohol-based disinfectant (Bacillol) and twice daily cleaning of floors with chlorine solution (1,000 ppm/L. Samples were collected prior to daily cleaning.	Copper-coated surfaces had significantly lower (p=0.003) percent surfaces positive at 13.8% compared to non-copper surfaces at 22.7%. Copper coating also had significantly lower (p=0.049) mean bacterial count (standard deviation) with 261 CFU/100 cm^2 (1,380) compared to 1,226 CFU/100 cm^2 (8.893).
Inkinen 2017 (8890)	Gram-negative bacteria	Single-site, quasi-experimental, controlled cohort study over a 2 month period	42 samples from toilet surfaces (flush button, support rail) in 8 rooms at a hospital. Satakunta, Finland.	Copper and copper alloy touch surface products (99.90% Cu-DHP) were used on the treatment surfaces. The control group was plastic toilet buttons and chromed support rails. Samples were collected weekly before daily cleaning.	Number of surfaces positive for gram-negative organisms was not lower for copper surfaces compared to control: 3/15 copper compared to 3/12 for control on flush buttons; 1/8 for copper compared to 2/7 for control on support rails.
Souli 2017 (7928)	Gram-negative bacteria (*Acinetobacter baumannii*)	Single-site, controlled, crossover study over a	685 samples from surfaces (bed rails, side table, IV pole, side cart, etc.) in	Intervention period 1: Copper-alloy-coated bed and accessories (Hellenic Copper Development Institute) were placed next to non-coated bed and	Copper-coated surfaces had lower (p=0.07) percent surfaces positive at 9% compared to non-copper surfaces at 13.6%.

Reference (Study ID)	Outcome	Study Design	Setting	Intervention	Results
		16 month period	two rooms of ICU at 650-bed tertiary care academic hospital. Athens, Greece.	accessories in the same ICU compartment for 5 months. Intervention period 2: copper and non-copper items were placed in separate ICU compartments for 9 months. Cleaning protocol used spray alcohol-based disinfectant (Bacillol) and twice daily cleaning of floors with chlorine solution (1,000 ppm/L. Samples were collected prior to daily cleaning.	
Karpanen 2012 (6414)	Gram-negative bacteria (Coliforms)	Single-site, controlled cross-over study over a 24 week period	~672 samples from frequent-touch items (door handles and push plates, toilet seats and flush handles, grab rails, light switches and pull cord toggles, sockets, overbed tables, dressing trolleys, commodes, taps, and sink fittings) in acute care medical ward at a large university hospital. UK.	14 types of copper items were installed 3 months before beginning of the study and include surfaces with copper-alloy (58-99.5% copper). Comparator items were composed of anodized aluminum, steel, plastic, chromium plated brass, etc. Sampled weekly before afternoon cleaning which consisted of detergent, hot water, and 1000 ppm chlorine (sodium dichloroisocyanurate, product unspecified, contact time unspecified). Copper and control items were switched after 12 weeks.	Coliforms were more often found on control surfaces compared to copper surfaces. Odds ratio (95% confidence ratio) of copper surface positive for Coliforms compared to control surface was 0.398 (0.229 – 0.692), p=0.001.
Souli 2017 (7928)	Gram-negative bacteria (*Klebsiella pneumonia*)	Single-site, controlled, crossover study over a 16 month period	685 samples from surfaces (bed rails, side table, IV pole, side cart, etc.) in two rooms of ICU at 650-bed tertiary care academic hospital. Athens, Greece.	Intervention period 1: Copper-alloy-coated bed and accessories (Hellenic Copper Development Institute) were placed next to non-coated bed and accessories in the same ICU compartment for 5 months. Intervention period 2: copper and non-copper items were placed in separate ICU compartments for 9 months. Cleaning protocol used spray alcohol-based disinfectant (Bacillol) and twice daily	Copper-coated surfaces had somewhat reduced (p=0.156) percent surfaces positive at 0.3% compared to non-copper surfaces at 1.3%.

Reference (Study ID)	Outcome	Study Design	Setting	Intervention	Results
				cleaning of floors with chlorine solution (1,000 ppm/L. Samples were collected prior to daily cleaning.	
Souli 2017 (7928)	Gram-negative bacteria (MDR)	Single-site, controlled, crossover study over a 16 month period	685 samples from surfaces (bed rails, side table, IV pole, side cart, etc.) in two rooms of ICU at 650-bed tertiary care academic hospital. Athens, Greece.	Intervention period 1: Copper-alloy-coated bed and accessories (Hellenic Copper Development Institute) were placed next to non-coated bed and accessories in the same ICU compartment for 5 months. Intervention period 2: copper and noncopper items were placed in separate ICU compartments for 9 months. Cleaning protocol used spray alcohol-based disinfectant (Bacillol) and twice daily cleaning of floors with chlorine solution (1,000 ppm/L. Samples were collected prior to daily cleaning.	Copper-coated surfaces had lower (p=0.058) percent surfaces positive for MDR gram-negative bacteria with 27.5% of copper surfaces compared to 80% of non-copper surfaces in first period of intervention 41% on copper surfaces compared to 70% on non-copper surfaces in second period of intervention (p=0.185).
Karpanen 2012 (6414)	Gram-positive bacilli (*Clostridium difficile*)	Single-site, controlled cross-over study over a 24 week period	~672 samples from frequent-touch items (door handles and push plates, toilet seats and flush handles, grab rails, light switches and pull cord toggles, sockets, overbed tables, dressing trolleys, commodes, taps, and sink fittings) in acute care medical ward at a large university hospital. UK.	14 types of copper items were installed 3 months before beginning of the study and include surfaces with copper-alloy (58-99.5% copper). Comparator items were composed of anodized aluminum, steel, plastic, chromium plated brass, etc. Sampled weekly before afternoon cleaning which consisted of detergent, hot water, and 1000 ppm chlorine (sodium dichloroisocyanurate, product unspecified, contact time unspecified). Copper and control items were switched after 12 weeks.	*C. difficile* was found similarly on copper and control surfaces. Odds ratio (95% confidence ratio) of copper surface positive for *C. difficile* compared to control surface was 3.920 (0.828 – 18.551), p=0.108.
Schmidt 2012 (2654)	Gram-positive cocci (*Enterococcus* spp.-	Multisite, quasi-experimental, controlled	6 frequent-touch surfaces (side rails of patient bed, over-bed tray table, IV	At month 23 of 43, copper-alloy surfaces were installed onto six frequent-touch surfaces in half of rooms. The other half of rooms had control surfaces that were	Percent surfaces positive for VRE was significantly higher at 3% (91/3004) on control surfaces compared to 0.3% (9/2781) on copper surfaces (p<0.001).

Reference (Study ID)	Outcome	Study Design	Setting	Intervention	Results
	Vancomycin-resistant enterococci (VRE))	before-after study over a 43 month period	pole, arm rest of chair, call button, computer) in 16 ICU rooms at 3 hospitals: 660-bed academic facility, 432-bed cancer hospital, 98-bed veterans hospital. New York and South Carolina, USA.	not copper-alloy (e.g. wood, plastic, stainless steel). Samples were collected at the same time each week, however unspecified relative to cleaning schedule.	
Karpanen 2012 (6414)	Gram-positive cocci (*Enterococcus* spp.- Vancomycin-resistant enterococci (VRE))	Single-site, controlled cross-over study over a 24 week period	~672 samples from frequent-touch items (door handles and push plates, toilet seats and flush handles, grab rails, light switches and pull cord toggles, sockets, overbed tables, dressing trolleys, commodes, taps, and sink fittings) in acute care medical ward at a large university hospital. UK.	14 types of copper items were installed 3 months before beginning of the study and include surfaces with copper-alloy (58-99.5% copper). Comparator items were composed of anodized aluminum, steel, plastic, chromium plated brass, etc. Sampled weekly before afternoon cleaning which consisted of detergent, hot water, and 1000 ppm chlorine (sodium dichloroisocyanurate, product unspecified, contact time unspecified). Copper and control items were switched after 12 weeks.	VRE was more often found on control surfaces compared to copper surfaces. Odds ratio (95% confidence ratio) of copper surface positive for VRE compared to control surface was 0.095 (0.012 – 0.748), p=0.005.
Souli 2017 (7928)	Gram-positive cocci (*Enterococcus* spp.)	Single-site, controlled, crossover study over a 16 month period	685 samples from surfaces (bed rails, side table, IV pole, side cart, etc.) in two rooms of ICU at 650-bed tertiary care academic hospital. Athens, Greece.	Intervention period 1: Copper-alloy-coated bed and accessories (Hellenic Copper Development Institute) were placed next to non-coated bed and accessories in the same ICU compartment for 5 months. Intervention period 2: copper and noncopper items were placed in separate ICU compartments for 9 months. Cleaning	Copper-coated surfaces had significantly lower (p=0.014) percent surfaces positive at 1.3% compared to non-copper surfaces at 4.5%.

Reference (Study ID)	Outcome	Study Design	Setting	Intervention	Results
				protocol used spray alcohol-based disinfectant (Bacillol) and twice daily cleaning of floors with chlorine solution (1,000 ppm/L). Samples were collected prior to daily cleaning.	
Inkinen 2017 (8890)	Gram-positive cocci (*Enterococcus* spp.)	Single-site, quasi-experimental, controlled cohort study over a 2 month period	42 samples from toilet surfaces (flush button, support rail) in 8 rooms at a hospital. Satakunta, Finland.	Copper and copper alloy touch surface products (99.90% Cu-DHP) were used on the treatment surfaces. The control group was plastic toilet buttons and chromed support rails. Samples were collected weekly before daily cleaning.	Number of surfaces positive for Enterococci was not lower for copper surfaces compared to control: 5/15 copper compared to 2/12 for control on flush buttons; 2/8 for copper compared to 1/7 for control on support rails.
Schmidt 2012 (2654)	Gram-positive cocci (*Staphylococcus aureus*-MRSA)	Multisite, quasi-experimental, controlled before-after study over a 43 month period	6 frequent-touch surfaces (side rails of patient bed, over-bed tray table, IV pole, arm rest of chair, call button, computer) in 16 ICU rooms at 3 hospitals: 660-bed academic facility, 432-bed cancer hospital, 98-bed veterans hospital. New York and South Carolina, USA.	At month 23 of 43, copper-alloy surfaces were installed onto six frequent-touch surfaces in half of rooms. The other half of rooms had control surfaces that were not copper-alloy (e.g. wood, plastic, stainless steel). Samples were collected at the same time each week, however unspecified relative to cleaning schedule.	Percent surfaces positive for MRSA was higher at 0.63% (19/3004) on control surfaces compared to 0.3% (8/2781) on copper surfaces (p=0.0804).
Karpanen 2012 (6414)	Gram-positive cocci (*Staphylococcus aureus*-MRSA)	Single-site, controlled cross-over study over a 24 week period	~672 samples from frequent-touch items (door handles and push plates, toilet seats and flush handles, grab rails, light switches and pull cord toggles, sockets, overbed tables,	14 types of copper items were installed 3 months before beginning of the study and include surfaces with copper-alloy (58-99.5% copper). Comparator items were composed of anodized aluminum, steel, plastic, chromium plated brass, etc. Sampled weekly before afternoon cleaning which consisted of detergent, hot water, and 1000 ppm chlorine (sodium dichloroisocyanurate, product	MRSA was found similarly on copper and control surfaces. Odds ratio (95% confidence ratio) of copper surface positive for MRSA compared to control surface was 0.621 (0.306 – 1.262), p=0.217.

Reference (Study ID)	Outcome	Study Design	Setting	Intervention	Results
			dressing trolleys, commodes, taps, and sink fittings) in acute care medical ward at a large university hospital. UK.	unspecified, contact time unspecified). Copper and control items were switched after 12 weeks.	
Karpanen 2012 (6414)	Gram-positive cocci (*Staphylococcus aureus*-MSSA)	Single-site, controlled cross-over study over a 24 week period	~672 samples from frequent-touch items (door handles and push plates, toilet seats and flush handles, grab rails, light switches and pull cord toggles, sockets, overbed tables, dressing trolleys, commodes, taps, and sink fittings) in acute care medical ward at a large university hospital. UK.	14 types of copper items were installed 3 months before beginning of the study and include surfaces with copper-alloy (58-99.5% copper). Comparator items were composed of anodized aluminum, steel, plastic, chromium plated brass, etc. Sampled weekly before afternoon cleaning which consisted of detergent, hot water, and 1000 ppm chlorine (sodium dichloroisocyanurate, product unspecified, contact time unspecified). Copper and control items were switched after 12 weeks.	MSSA was found more often on control surfaces compared to copper surfaces. Odds ratio (95% confidence ratio) of copper surface positive for MSSA compared to control surface was 0.262 (0.112 – 0.612), p=0.001.
Souli 2017 (7928)	Gram-positive cocci (*Staphylococcus aureus*)	Single-site, controlled, crossover study over a 16 month period	685 samples from surfaces (bed rails, side table, IV pole, side cart, etc.) in two rooms of ICU at 650-bed tertiary care academic hospital. Athens, Greece.	Intervention period 1: Copper-alloy-coated bed and accessories (Hellenic Copper Development Institute) were placed next to non-coated bed and accessories in the same ICU compartment for 5 months. Intervention period 2: copper and noncopper items were placed in separate ICU compartments for 9 months. Cleaning protocol used spray alcohol-based disinfectant (Bacillol) and twice daily cleaning of floors with chlorine solution (1,000 ppm/L. Samples were collected prior to daily cleaning.	Copper-coated surfaces did not have reduced (p=0.446) percent surfaces positive at 0.6% compared to non-copper surfaces at 0.3%.

Reference (Study ID)	Outcome	Study Design	Setting	Intervention	Results
Inkinen 2017 (8890)	Gram-positive cocci (*Staphylococcus aureus*)	Single-site, quasi-experimental, controlled cohort study over a 2 month period	42 samples from toilet surfaces (flush button, support rail) in 8 rooms at a hospital. Satakunta, Finland.	Copper and copper alloy touch surface products (99.90% Cu-DHP) were used on the treatment surfaces. The control group was plastic toilet buttons and chromed support rails. Samples were collected weekly before daily cleaning.	Number of surfaces positive for S. *aureus* was not lower for copper surfaces compared to control: 0/15 copper compared to 2/12 for control on flush buttons; 0/8 for copper compared to 1/7 for control on support rails.
Montero 2019 (13703)	Gram-positive cocci (*Staphylococcus* spp.)	Single-site, quasi-experimental, controlled before-after study over 2-month period	158 samples were collected from 4 high-touch surfaces (bed rails, overbed table, bedside table and IV pole) in two patient rooms in an adult intensive care unit at university-affiliated hospital. Santiago, Chile.	Surfaces were coated with 60% copper, 40% agglomerate resin (Copper Armour™, copper particles in a methyl methacrylate resin DEGADUR 527, Evonik). Uncoated surfaces from adjacent patient room was the control. Samples were collected at the same time and before morning cleaning every week.	There was an 88.9% reduction (p=0.001) of *Staphylococcus* spp. concentration on bed rails, 35.7% reduction (p=0.106) on overbed table, 72.9% on the bedside table (p=0.289), 62.5% reduction on the IV pole when compared to control (p=0.231). Average concentration ranged from 22.5 to 463 CFU/100 cm^2 on copper-coated surfaces compared to 60 to 2445 CFU/100cm^2 on control surfaces.
Sifri 2016 (7971)	HAI (HAI – (*C. difficile* + MDROs))	Single-site, quasi-experimental, controlled before-after study over a 26 month period	23,889 patients in unmodified with non-copper surfaces or new hospital wing with copper-containing surfaces (countertops, high-touch surfaces, bedrails, etc.). Patients were in single-occupancy beds at a community hospital. Virginia, USA.	A new hospital wing was constructed and outfitted with 16% copper oxide impregnated composite countertops and molded surfaces (Cupron Enhanced EOS Solid Surfaces). Copper-impregnated linens were also deployed. The control wing was an unmodified wing without copper surfaces or linens. HAI outcomes compared acute care hospital beds of original hospital to acute care hospital beds of new wing and to the unmodified wing.	There was 78% fewer HAIs due to MDROs or *C. difficile* relative to baseline. Incidence rate on wing without copper (8.34 events per 10,000 patient-days) was not significantly different (p=0.352) from baseline period (6.25 events per 10,000 patient days). The new wing with copper had 1.38 events per 10,000 patient days which was significantly different (p=0.023) from the baseline period.
Sifri 2016 (7971)	HAI (HAI – *C. difficile*)	Single-site, quasi-experimental, controlled before-after	23,889 patients in unmodified with non-copper surfaces or new hospital wing with copper-	A new hospital wing was constructed and outfitted with 16% copper oxide impregnated composite countertops and molded surfaces (Cupron Enhanced EOS Solid Surfaces). Copper-impregnated	There were 83% fewer cases of *C. difficile* infection compared to historic data. Incidence rate on wing without copper (4.69 events per 10,000 patient-days) was not significantly different (p=0.736) from

Reference (Study ID)	Outcome	Study Design	Setting	Intervention	Results
		study over a 26 month period	containing surfaces (countertops, high-touch surfaces, bedrails, etc.). Patients were in single-occupancy beds at a community hospital. Virginia, USA.	linens were also deployed. The control wing was an unmodified wing without copper surfaces or linens. HAI outcomes compared acute care hospital beds of original hospital to acute care hospital beds of new wing and to the unmodified wing.	baseline period (4.10 events per 10,000 patient days). The new wing with copper had 0.69 events per 10,000 patient days which was significantly different (p=0.048) from the baseline period.
Sifri 2016 (7971)	HAI (HAI – MDROs (all but *C. difficile*))	Single-site, quasi-experimental, controlled before-after study over a 26 month period	23,889 patients in unmodified with non-copper surfaces or new hospital wing with copper-containing surfaces (countertops, high-touch surfaces, bedrails, etc.). Patients were in single-occupancy beds at a community hospital. Virginia, USA.	A new hospital wing was constructed and outfitted with 16% copper oxide impregnated composite countertops and molded surfaces (Cupron Enhanced EOS Solid Surfaces). Copper-impregnated linens were also deployed. The control wing was an unmodified wing without copper surfaces or linens. HAI outcomes compared acute care hospital beds of original hospital to acute care hospital beds of new wing and to the unmodified wing.	There were 68% fewer MDRO infections compared to baseline. Incidence rate on wing without copper (3.65 events per 10,000 patient-days) was not significantly different (p=0.28) from baseline period (2.16 events per 10,000 patient days). The new wing with copper had 0.69 events per 10,000 patient days which was also not significantly different (p= 0.252) from the baseline period.
Salgado 2013 (11135)	HAI (HAI)	Multi-site randomized control trial over a 11 month period	650 patients were admitted to ICU in 16 rooms of ICU at Medical University of South Carolina	Patients were randomly assigned room with or without copper-containing surfaces (bed rails, overbed tables, IV poles, visitor chair arm, etc.). The control group used QAC (Virex 256) for routine (at least daily) and terminal cleaning as well as hypochlorite disinfectant (Dispatch) for rooms housing patients with C difficile. All patients were screened for MRSA and two sites screened for VRE.	The proportion of patients who developed an HAI in the control rooms was significantly higher at 0.081 compared to 0.034 in rooms with copper items (p=0.013). There was a significant reduction in new HAI or colonization in the copper rooms vs the control rooms (p=0.020).
von Dessauer	HAI (HAI)	Single-site, randomized	515 infants > 72 h stay in hospital	Infants were sequentially assigned to a room with or without items with copper	There was decreased HAI rate in rooms with copper items compared to rooms

Reference (Study ID)	Outcome	Study Design	Setting	Intervention	Results
2016 (10314)		controlled trial over 12 month period	pediatric intensive care unit (PICU) or intermediate pediatric care unit (PIMCU) at 249-bed tertiary hospital. Santiago, Chile.	alloyed surfaces (bed rails, bed rail levers, intravenous pole, sink handles, and nurses' workstation). HAI was assessed by diagnosis. Surveillance not indicated.	without. HAI incidence rate was 13.0 per 1000 patient-days in control rooms compared to 10.6 per 1000 patient days in rooms with copper corresponding to relative risk reduction of 0.81 (90% CI: 0.50 – 1.32). This corresponded to a moderate reduction (19% crude, 25% adjusted) of HAI incidence compared to control though not statistically significant.

Table S18: Study results for other, non-copper surface interventions ordered by outcome organism

Reference (Study ID)	Outcome	Study Design	Setting	Intervention	Results
de Jong 2018 (346)	All viable organisms	Single-site, quasi-experimental over 12 weeks: 2-week wash-in, 4-week control measurement (pre-intervention), 2-week wash-in, 4-week intervention measurement	192 sets of samples from 6 high-touch, near-patient surfaces (wall, floor, bed rail, door and door handle, ceiling, computer keyboard, bedside table, monitor arm, medical equipment cabinet) in 4 isolation rooms in a Level III ICU in university-affiliated Gelderse Vallei Hospital in Ede, The Netherlands	Titanium dioxide coating (Miracle Titanium MVX, Maeda Kougyou, Kitakyushu, Japan) vs. uncoated surfaces (pre-intervention); cleaning of both intervention and control surfaces with alcohol-based disinfectants continued, per hospital protocol. Surfaces were sampled at baseline (after wash-in, before coating) then once a week during the 4-week intervention period. Samples were collected at 8 PM, four hours after the most recent cleaning at 4 PM.	Mean CFUs per room was 161 pre-intervention vs. 121 post- intervention. Mean (standard deviation) ratio of CFUs per room post- vs. pre-intervention was 0.94 (0.64). Significance not reported.
Boyce 2014 (810)	All viable organisms	Single site, quasi-experimental controlled cohort study over 4-week period	1587 samples from high-touch surfaces (bedside rail, overbed table, TV remote, telephone, door handle, dresser, toilet seat, bathroom grab bar, sink handles) in 9 patient rooms on rehabilitation ward at 500-bed community teaching hospital. Connecticut, USA.	Daily cleaning consisted of QAC (Virex 256). In three intervention rooms, 9 high-touch sites were allowed to dry after daily cleaning and then coated with one of two organosilane test products with microfiber cloth: (i) Eco Antimicrobial, (ii) Bio-Protect AM500. Test products were not applied to surfaces in three control rooms. Samples were collected from each site daily before daily cleaning.	Neither test product had lower mean colony counts than control rooms for most sites. Mean colony count ranged from approximately 15-115 for control sites, ~25-130 for test product 1, and ~15-115 for test product 2 depending on site type. Control sites had significantly lower mean colony count compared to the test products.
Edmiston 2020 (904)	All viable organisms	Single-site, Quasi-experimental, controlled before-after study, over 8 weeks	400 samples from high-touch surfaces (telephone handpieces, computer keyboards, physician workspaces, patient room door handles, and patient tables) in	Daily baseline sampling over 2 weeks was conducted. Surfaces were assigned randomly to a control or intervention group. Intervention surfaces received surface treatment with antimicrobial/ disinfectant coating (IOS, quaternary	Baseline ATP range before intervention was 202-870 RLU. There was a significant reduction in the mean ATP bioluminescence and bacterial count at 1 and 6 weeks after the intervention on treatment surfaces compared to control surfaces ($p < 0.001$). Mean ATP ranged from 13 – 82 RLU on treatment surfaces 1 week after the

Reference (Study ID)	Outcome	Study Design	Setting	Intervention	Results
			a 26-bed ICU in a tertiary 505-bed health care facility. Wisconsin, USA	ammonium compound coating, isopropyl alcohol/organofunctional silane solution) applied with microfiber cloth and allowed to dry. Samples were taken daily on week 1 and week 6 after intervention. Sample collection relative to routine cleaning not specified.	intervention compared to 67-743 RLU on control surfaces. The range of average bacterial count 1 week after the intervention was 15 – 119 CFU on control surfaces and 0 – 2.5 CFU on treatment surfaces. After 6 weeks, ATP ranged from 36-52 on treated surfaces compared to 93-717 on control surfaces. After 6 weeks, range of the average bacterial count was 11 – 89 CFU on control surfaces compared to 0 – 2.3 CFU on treatment surfaces.
Lee 2017 (1548)	All viable organisms	Single-site, quasi-experimental cohort study with 3-month period of simultaneous intervention and control with comparison to a 3-month pre-intervention period	1496 samples from 17 sites, including fomites (direct patient contact: mattress, bed clothes, bed rails) and patient surroundings (wall, curtains, ECG monitor. dining table, chart, cupboard) in 2 ICUs (MICU and SICU) in Wan Fang Medical Center, Taipei, Taiwan	Nanomaterial consisting of inorganic metal and organic quaternary ammonium (Bio-Kil, Cargico Group) vs control (untreated surface); cleaning of both intervention and control surfaces continued with 500-ppm sodium hypochlorite, per hospital protocol. Surfaces were sampled twice a week for 3 months after treatment. Sample collection relative to cleaning unspecified.	Mean concentration reduction treated vs control surfaces was 75.3% (initial load 12,962 CFUs) vs 78.3% (initial load 11,098 CFUs) for fomites, and 80.5% (initial load 3,252 CFUs) vs 3.3% (initial load 1,835 CFUs) for patient surroundings ($P<0.001$)
Lewis 2015 (1585)	All viable organisms	Single site, quasi-experimental, controlled cohort study over a 6-week period	A total of 720 samples were collected from surfaces (telephone handpieces, computer keyboards, physician workstations, door handles, outer surface blood pressure cuffs, bed tables) in 4 operating rooms (one hybrid, one transplant,	Control surfaces had terminal cleaning with quaternary disinfectant (unspecified product, active ingredient, and contact time) twice a week. Treated surfaces had antimicrobial isopropyl alcohol/organofunctional silane (IOS) solution applied and allowed to dry at beginning of intervention. Baseline samples were taken after QAC	Baseline samples had between 29.9-57.8%, surfaces in surfaces designated as dirty (>46 RLUs) by ATP bioluminescence assay in operating rooms. During the intervention phase, the average adenosine triphosphate bioluminescence (ATB) for untreated sites was significantly higher ($p=0.048$) (242.0 RLU, range 19.4-2872.6 RLU) compared to IOS treatment (67.6 RLU, range 0-297.5 RLU) for treated sites. Percent of surfaces negative was 20% for untreated surfaces compared to 82.5% for treated surfaces. The

Reference (Study ID)	Outcome	Study Design	Setting	Intervention	Results
			and two general surgical). Wisconsin, USA.	disinfection. During intervention period, samples were taken 3 times a week for 6 weeks after terminal cleaning.	mean concentration among culture-positive surfaces for untreated sites was significantly (p<0.001) higher at 14.3 CFU compared to 1.7 CFU for treated sites.
Karunanayake 2019 (2035)	All viable organisms	Single-site, quasi-experimental cohort study with simultaneous control over 12 weeks	5 surfaces in an ICU (telephone, bedrail, doorknob, nurse's table, stethoscope) and 1 surface (bedrail)[a] in a general medicine ward in Base Hospital Homagama, Sri Lanka (identical type and number of coated and uncoated surfaces)	Silicon nano-coating surface (Bacterlon, Nanopool GmbH) vs. control (uncoated surfaces); cleaning of both intervention and control surfaces continued, with general purpose detergents (ward, ICU) and 70% alcohol (stethoscope, trolley), per hospital protocol. Surfaces were sampled once a week for 12 weeks after application of coating	Baseline bioburden was < 1 CFU/cm2 on coated and uncoated surfaces. Mean concentration reduction of bioburden was 56.43% in the ICU and 36.15% in the ward when compared to control. All 6 coated surfaces showed significant reductions in bioburden compared to corresponding uncoated surfaces, after 12 weeks.
Ortí-Lucas 2017 (2261)	All viable organisms	Single site, quasi-experimental controlled cohort study over a 3-month period	615 samples from surfaces (tables, sinks, beds, walls) in two wards of a recovery unit (PACU) at NHS Foundation Trust hospital. Valencia, Spain	In two similar wards, one ward was treated with antimicrobial application while the other was left untreated. The application was composed of laminar nanoclay-based antimicrobial additives containing silver ions (BB635A1 0.3%BactiBlock 101 R4.47 and 0.3% Zink Pyrithione) on bedside tables, walls, beds. A Monolayer polyurethane coating (BB655A0 0.3% BactiBock 101 R4.47) was applied to floors and sinks. Cleaning consisted of QAC-based disinfectant on surfaces and disinfection of floors (15.05% alkyl dimethyl ammonium and 1.5% bis-(3-aminopropyl)-dodecylamine) in both wards. Samples were	Percent samples positive were not significantly different among treated and untreated surfaces with non-treated surfaces somewhat less contaminated (< 0.03 CFU/cm2) (rate ratio=1.15, 95% CI=0.90-1.47, p=0.26). Mean counts were significantly higher on bedside tables that were treated (0.16 CFU/cm2) compared to untreated surfaces (0.10 CFU/cm2).

Reference (Study ID)	Outcome	Study Design	Setting	Intervention	Results
				collected weekly at the same time.	
Özpolat 2011 (2287)	All viable organisms	Single site, quasi-experimental uncontrolled before-after study over 1-month period	Surfaces in operating room, ICU, physician room, wards, and hospital kitchens at a hospital. Kirikkale, Turkey	The intervention consisted of coating surfaces with a photocatalyst consisting of TiO2 and iron: apatite coated iron titante (Easycoat). Samples were collected before and one month after surfaces were coated. Sample collection relative to cleaning not specified.	After one month, particle count from lumimeter on treated surfaces was significantly reduced 97.15% in operating room, 95.61% in ICU, 98.30 in physicians' room, 94.13% in wards and 97.04% in hospital kitchen compared to before treatment.
Prindis 2018 (2616)	All viable organisms	Single-site, quasi-experimental cohort study with simultaneous control over 4 weeks	279 samples from 3 surfaces (bed handles, bed temples, inside of entrance doors) in 2 patient rooms (one coated, one uncoated), unknown ward, in University Hospital Hradec Kralove, Kladno, Czech Republic	Titanium dioxide nanoparticles (SmartCoat) vs. control (uncoated surface); cleaning of both intervention and control surfaces continued (unknown protocol or disinfectant type). Surfaces were sampled twice a day for 4 weeks after application of coating. Sample collection relative to cleaning unspecified.	Decreased level of ATP bioluminescence contamination, relative to control, in all monitored places (P=0.041): bed handles, 28% (-1060 RLU), P =0.04; Bed temples, 90% (-1890 RLU), P<0.0001); inside of entrance doors,30% (-70RLU), P =0.016
Reid 2018 (3071)	All viable organisms	Single-site, quasi-experimental cohort study with simultaneous control over 12 weeks	102 samples from high-touch surfaces on beds (rails, control panel, table, floorboard, locker) in a general medicine ward and a stroke unit (control surfaces only) in New Cross Hospital, Wolverhampton, UK.	Titanium dioxide-based (1.5% Titania and 0.1% silver zeolite diluted solution) photocatalytic coating (MVX Hitech Co. Ltd, Kitakyushu, Japan) vs. control (uncoated surfaces); cleaning of both intervention and control surfaces with alcohol-based detergents continued, per hospital protocol. Surfaces were sampled twice a week for 12 weeks after application of coating. Sample collection relative to cleaning unspecified.	Mean proportion of surfaces with <2.5 CFU/cm^2 was 80.4% for coated surfaces vs. 52.9% for uncoated, P<0.001; OR: 0.95; 95% CI: 0.925 to 0.977. Odds of >2.5 CFU/cm^2 fell by 2.5% per day for coated surfaces (OR=0.95; 95% CI: 0.925 to 0.977; P < 0.001) vs. increasing 2.6% per day for uncoated (OR=1.026; 95%CI 1.009 to 1.043; P = 0.003
Taylor 2009 (4152)	All viable organisms	Single site, quasi-	Surfaces (door, door handle, electrical	Both units had daily cleaning with wet mopping of floors with	Mean bacterial count on untreated sites ranged from 96-1140 CFU compared to 12-

Reference (Study ID)	Outcome	Study Design	Setting	Intervention	Results
		experimental cohort study over 4-month period	switch, blinds, chair, treatment couch, sign, waste bin, tiles) in two outpatient units at a major acute NHS trust.	detergent, damp dusting with detergent. Treated products in recently refurbished unit were treated 12 months before the initiation of sampling. Treatment consisted of BioCote (silver ion-treated). Untreated products in the second similar unit were composed of the same materials as the treated products but were not identical. Sample collection frequency not specified. Sample collection relative to routine cleaning not specified.	119 at treated sites. Bacterial counts were reduced between 62-98% on treated products compared to untreated products. A subset of untreated samples were in the same refurbished unit with treated samples and had 44% less bacterial counts compared to untreated samples in the non-refurbished unit.
Tamimi 2014 (10851)	All viable organisms	Single-site, quasi-experimental uncontrolled before-after over a 15-week period	6 sets of samples from 95 sites on surfaces in patient rooms (bed rails, bed controls, tray table, and wall), nurses station, and lobby (countertops, phones, computer keyboards, chair armrests, end table) in an ICU in a community hospital in Los Angeles, CA	Surface coating composed of quaternary ammonium silyl oxide and titanyl oxide moieties (ABS-G2015, Allied BioScience, Point Roberts, WA) vs control (baseline); cleaning of intervention surfaces continued with bleach and/or quaternary ammonium wipes. Surfaces were sampled at 1, 2, 4, 8, and 15 weeks after application of coating. Sample collection relative to cleaning unspecified.	Average concentration reduction from initial load (233,064 CFU/100cm^2, all sites) was 3 logs at 4 weeks, 2 logs at 8 weeks, and ~2 logs at 15 weeks; percent sites with >10,000 CFU/100 cm2 was 71.5% at baseline, 0% at 1,2,4, and 8 weeks, and 11.1% at 15 weeks (P<0.0005)
Lee 2017 (1548)	All viable organisms (MDRO)	Single-site, quasi-experimental cohort study with simultaneous intervention and control over 3 months, with	1496 samples from 17 sites, including fomites (direct patient contact: mattress, bed clothes, bed rails) and patient surroundings (wall, curtains, ECG monitor. dining table, chart, cupboard) in 2 ICUs (MICU and	Nanomaterial consisting of inorganic metal and organic quaternary ammonium (Bio-Kil, Cargico Group) vs control (untreated surface); cleaning of both intervention and control surfaces with 500-ppm sodium hypochlorite continued, per hospital protocol. Surfaces were sampled twice a week for 3	Percent reduction of prevalence (percent positive samples) of MDRO, treated vs control was 45.8% (19.5% to 10.6%) vs 34.6% (19.5% to 12.8%) for fomites (P=0.002) and 83.1% (12.2% to 2.1%) vs 65.1% (12.2% to 4.3%) for surroundings (P=0.004).

Reference (Study ID)	Outcome	Study Design	Setting	Intervention	Results
		comparison to a 3-month pre-intervention period	SICU) in Wan Fang Medical Center, Taipei, Taiwan	months after treatment. Sample collection relative to cleaning unspecified.	
Lee 2017 (1548)	Gram-negative bacteria (*Acinetobacter baumannii*- Carbapenem-resistant *Acinetobacter calcoaceticus baumannii* complex (CRAB))	Single-site, quasi-experimental cohort study with simultaneous control over 3 months, with comparison to a 3-month pre-intervention period	1496 samples from 17 sites, including fomites (direct patient contact: mattress, bed clothes, bed rails) and patient surroundings (wall, curtains, ECG monitor. dining table, chart, cupboard) in 2 ICUs (MICU and SICU) in Wan Fang Medical Center, Taipei, Taiwan	Nanomaterial consisting of inorganic metal and organic quaternary ammonium (Bio-Kil, Cargico Group) in two rooms vs control (untreated surface) in two rooms; cleaning of both intervention and control surfaces with 500-ppm sodium hypochlorite continued, per hospital protocol. Surfaces were sampled twice a week for 3 months after treatment. Sample collection after daily routine cleaning.	Percent reduction of prevalence (percent positive samples) for CRAB on fomite surfaces was 34.9% (9.8% to 6.4%) for treated vs 100% (9.8% to 0%) for control (p=0.135). Prevalence on patient surrounding surfaces was not significantly different for treated (2.4% to 2.1%) and untreated (2.4% to 4.3%) surfaces (p=0.135).
Thom 2014 (4132)	Gram-negative bacteria (*Acinetobacter baumannii*)	Single site, controlled cohort study over 2 months	343 samples from surfaces (sink basin, counter, call button, bedside table, bedside patient vital signs monitor, telephone, supply cart, door handle, floor) in 10-bed adult surgical intermediate care unit at university hospital. Maryland, USA.	Prior to treatment application, all rooms were cleaned and HPV was used to decontaminate surfaces. The silicone quaternary amine antimicrobial surface polymer (MSDS Poly, active ingredient 3-(trimethoxysily)-propyl dimethyl actadecyl ammonium chloride) was applied to all surfaces (e.g. floors, walls, equipment, furniture) in five treated rooms using electrostatic sprayer. Five control rooms did not receive the treatment. Samples were collected 2 days per week from all rooms with patient occupancy. Sample collection	There was no difference (p=1.00) between percent rooms positive with 5% (1/20) in treated rooms and 4% (1/23) in untreated rooms.

Reference (Study ID)	Outcome	Study Design	Setting	Intervention	Results
				relative to cleaning protocol not specified.	
Karunanayake 2019 (2035)	Gram-negative bacteria (*Acinetobacter* spp.)	Single-site, quasi-experimental cohort study with simultaneous control over 12 weeks	5 surfaces in an ICU (telephone, bedrail, doorknob, nurse's table, stethoscope) and 1 surface (bedrail)[a] in a general medicine ward in Base Hospital Homagama, Sri Lanka (identical type and number of coated and uncoated surfaces)	Silicon nano-coating surface (Bacterlon, Nanopool GmbH) vs. control (uncoated surfaces); cleaning of both intervention and control surfaces continued, with general purpose detergents (ward, ICU) and 70% alcohol (stethoscope, trolley), per hospital protocol. Surfaces were sampled once a week for 12 weeks after application of coating. Sample collection relative to cleaning unspecified.	Frequency of isolated *Acinetobacter* spp showed significant reduction on coated surfaces compared to uncoated surfaces (8 vs 31, p < 0.01).
Karunanayake 2019 (2035)	Gram-negative bacteria (Coliforms)	Single-site, quasi-experimental cohort study with simultaneous control over 12 weeks	5 surfaces in an ICU (telephone, bedrail, doorknob, nurse's table, stethoscope) and 1 surface (bedrail)[a] in a general medicine ward in Base Hospital Homagama, Sri Lanka (identical type and number of coated and uncoated surfaces)	Silicon nano-coating surface (Bacterlon, Nanopool GmbH) vs. control (uncoated surfaces); cleaning of both intervention and control surfaces continued, with general purpose detergents (ward, ICU) and 70% alcohol (stethoscope, trolley), per hospital protocol. Surfaces were sampled once a week for 12 weeks after application of coating	Frequency of isolated coliforms did not show significant reduction on coated (n=7) surfaces compared to uncoated (n=10) surfaces.
Thom 2014 (4132)	Gram-negative bacteria (*E. coli*)	Single site, controlled cohort study over 2 months	343 samples from surfaces (sink basin, counter, call button, bedside table, bedside patient vital signs monitor, telephone, supply cart, door handle, floor) in 10-bed adult surgical intermediate care unit	Prior to treatment application, all rooms were cleaned and HPV was used to decontaminate surfaces. The silicone quaternary amine antimicrobial surface polymer (MSDS Poly, active ingredient 3-(trimethoxysily)-propyl dimethyl actadecyl ammonium chloride) was applied to all surfaces (e.g.	*E. coli* were not detected in treated or untreated rooms

Reference (Study ID)	Outcome	Study Design	Setting	Intervention	Results
			at university hospital. Maryland, USA.	floors, walls, equipment, furniture) in five treated rooms using electrostatic sprayer. Five control rooms did not receive the treatment. Samples were collected 2 days per week from all rooms with patient occupancy. Sample collection relative to cleaning protocol not specified.	
Karunanayake 2019 (2035)	Gram-negative bacteria (*Enterobacter* spp)	Single-site, quasi-experimental cohort study with simultaneous control over 12 weeks	5 surfaces in an ICU (telephone, bedrail, doorknob, nurse's table, stethoscope) and 1 surface (bedrail)[a] in a general medicine ward in Base Hospital Homagama, Sri Lanka (identical type and number of coated and uncoated surfaces)	Silicon nano-coating surface (Bacterlon, Nanopool GmbH) vs. control (uncoated surfaces); cleaning of both intervention and control surfaces continued, with general purpose detergents (ward, ICU) and 70% alcohol (stethoscope, trolley), per hospital protocol. Surfaces were sampled once a week for 12 weeks after application of coating	Frequency of isolated *Enterobacter* spp did not show significant reduction on coated surfaces (n=35) compared to uncoated surfaces (n=47).
Tamimi 2014 (10851)	Gram-negative bacteria (*Enterobacteriaceae*-Carbapenemase-resistant *Enterobacteriaceae* (CRE))	Single-site, quasi-experimental uncontrolled before-after over a 15-week period	6 sets of samples from 95 sites on surfaces in patient rooms (bed rails, bed controls, tray table, and wall), nurses station, and lobby (countertops, phones, computer keyboards, chair armrests, end table) in an ICU in a community hospital in Los Angeles, CA	Surface coating composed of quaternary ammonium silyl oxide and titanyl oxide moieties (ABS-G2015,Allied BioScience, Point Roberts, WA) vs baseline; cleaning of intervention surfaces continued with bleach and/or quaternary ammonium wipes. Surfaces were sampled at 1, 2, 4, 8, and 15 weeks after application of coating.	Prevalence (percent positive sites) of CRE was 3% at baseline and 0 through week 15

Reference (Study ID)	Outcome	Study Design	Setting	Intervention	Results
Afinogenova 2017 (67)	Gram-negative bacteria (*Enterobacteriaceae*)	Single-site, cohort study with simultaneous control and intervention over 35 days; partially seeded (*E. faecalis*)	Floors in 2 treatment rooms of research hospital in Russia (unknown number of samples)	Probiotics-based cleaning products (unspecified) compared to unspecified conventional disinfection methods	No growth after day 4 (with daily probiotic cleaning); significance not reported
de Jong 2018 (346)	Gram-negative bacteria (*Enterobacteriaceae*)	Single-site, quasi-experimental over 12 weeks: 2-week wash-in, 4-week control measurement (pre-intervention), 2-week wash-in, 4 week-intervention measurement	192 sets of samples from 6 high-touch, near-patient surfaces (wall, floor, bed rail, door and door handle, ceiling, computer keyboard, bedside table, monitor arm, medical equipment cabinet) in 4 isolation rooms in a Level III ICU in university-affiliated Gelderse Vallei Hospital in Ede, The Netherlands	Titanium dioxide coating (Miracle Titanium MVX, Maeda Kougyou, Kitakyushu, Japan) vs. uncoated surfaces (pre-intervention); cleaning of both intervention and control surfaces with alcohol-based disinfectants continued, per hospital protocol. Surfaces were sampled at baseline (after wash-in, before coating) then once a week during the 4-week intervention period	Mean count was 0 pre-intervention and 0 post-intervention; ratio of mean number of CFUs per room post- vs. pre-intervention (SD): 0.25 (0.50); Significance not reported.
Thom 2014 (4132)	Gram-negative bacteria (*Klebsiella pneumoniae*)	Single site, controlled cohort study over 2 months	343 samples from surfaces (sink basin, counter, call button, bedside table, bedside patient vital signs monitor, telephone, supply cart, door handle, floor) in 10-bed adult surgical intermediate care unit at university hospital. Maryland, USA.	Prior to treatment application, all rooms were cleaned and HPV was used to decontaminate surfaces. The silicone quaternary amine antimicrobial surface polymer (MSDS Poly, active ingredient 3-(trimethoxysily)-propyl dimethyl actadecyl ammonium chloride) was applied to all surfaces (e.g. floors, walls, equipment, furniture) in five treated rooms	*K. pneumoniae* were not detected in treated or untreated rooms

Reference (Study ID)	Outcome	Study Design	Setting	Intervention	Results
				using electrostatic sprayer. Five control rooms did not receive the treatment. Samples were collected 2 days per week from all rooms with patient occupancy. Sample collection relative to cleaning protocol not specified.	
Thom 2014 (4132)	Gram-negative bacteria (*Pseudomonas aeruginosa*)	Single site, controlled cohort study over 2 months	343 samples from surfaces (sink basin, counter, call button, bedside table, bedside patient vital signs monitor, telephone, supply cart, door handle, floor) in 10-bed adult surgical intermediate care unit at university hospital. Maryland, USA.	Prior to treatment application, all rooms were cleaned and HPV was used to decontaminate surfaces. The silicone quaternary amine antimicrobial surface polymer (MSDS Poly, active ingredient 3-(trimethoxysily)-propyl dimethyl actadecyl ammonium chloride) was applied to all surfaces (e.g. floors, walls, equipment, furniture) in five treated rooms using electrostatic sprayer. Five control rooms did not receive the treatment. Samples were collected 2 days per week from all rooms with patient occupancy. Sample collection relative to cleaning protocol not specified.	There was no difference (p=0.76) between percent rooms positive with 65% (13/20) in treated rooms and 43% (13/23) in untreated rooms.
Kim 2018 (12022)	Gram-positive bacilli (*Bacillus* spp.)	Single site, uncontrolled before-after study over 5 months	30 high-touch surfaces (bedside rails, tabletops, nursing trolley tops, door handles, faucet handler, computer keyboards) at 630-bed secondary care teaching hospital. South Korea.	5-month pre-intervention compared to 5-month post-intervention. High-touch surfaces coated with titanium dioxide-based photocatalyst. Standard cleaning of high-touch surfaces with chlorhexidine cloth and floors with detergent or sodium hypochlorite when known VRE or CDAD patient.	Percent surfaces positive was 4% (4/90) during the pre-intervention period compared to 2 after the intervention (2/90), p=0.41

Reference (Study ID)	Outcome	Study Design	Setting	Intervention	Results
Tamimi 2014 (10851)	Gram-positive bacilli (*Clostridium difficile*)	Single-site, quasi-experimental uncontrolled before-after over a 15-week period	6 sets of samples from 95 sites on surfaces in patient rooms (bed rails, bed controls, tray table, and wall), nurses station, and lobby (countertops, phones, computer keyboards, chair armrests, end table) in an ICU in a community hospital in Los Angeles, CA	Surface coating composed of quaternary ammonium silyl oxide and titanyl oxide moieties (ABS-G2015, Allied BioScience, Point Roberts, WA) vs baseline; cleaning of intervention surfaces continued with bleach and/or quaternary ammonium wipes. Surfaces were sampled at 1, 2, 4, 8, and 15 weeks after application of coating.	C difficile was not recovered from any of sites during the study period (prevalence was 0 at baseline through week 15)
Kim 2018 (12022)	Gram-positive cocci (Coagulase-negative *Staphylococcus* spp)	Single site, uncontrolled before-after study over 5 months	30 high-touch surfaces (bedside rails, tabletops, nursing trolley tops, door handles, faucet handler, computer keyboards) at 630-bed secondary care teaching hospital. South Korea.	5-month pre-intervention compared to 5-month post-intervention. High-touch surfaces coated with titanium dioxide-based photocatalyst. Standard cleaning of high-touch surfaces with chlorhexidine cloth and floors with detergent or sodium hypochlorite when known VRE or CDAD patient.	Significant decrease in organisms on high-touch surfaces was observed (23/90-26% vs 8/90-9%) in the intervention period compared to the pre-intervention period; p<0.01
Afinogenova 2017 (67)	Gram-positive cocci (*Enterococcus faecium*)	Single-site, cohort study with simultaneous control and intervention over 35 days ; partially seeded (E *faecalis*)	Floors in 2 treatment rooms of research hospital in Russia	Probiotics-based cleaning products (unspecified) compared to unspecified conventional disinfection methods	No growth after day 3 with daily probiotic cleaning; initial loads and significance not reported
Thom 2014 (4132)	Gram-positive cocci (*Enterococcus* spp-VRE and VSE)	Single site, controlled cohort study over 2 months	343 samples from surfaces (sink basin, counter, call button, bedside table, bedside patient vital signs	Prior to treatment application, all rooms were cleaned and HPV was used to decontaminate surfaces. The silicone quaternary amine antimicrobial	There were fewer (p=0.054) percent rooms positive with 35% (7/20) in treated rooms and 48% (11/23) in untreated rooms.

Reference (Study ID)	Outcome	Study Design	Setting	Intervention	Results
			monitor, telephone, supply cart, door handle, floor) in 10-bed adult surgical intermediate care unit at university hospital. Maryland, USA.	surface polymer (MSDS Poly, active ingredient 3-(trimethoxysily)-propyl dimethyl actadecyl ammonium chloride) was applied to all surfaces (e.g. floors, walls, equipment, furniture) in five treated rooms using electrostatic sprayer. Five control rooms did not receive the treatment. Samples were collected 2 days per week from all rooms with patient occupancy. Sample collection relative to cleaning protocol not specified.	
Lee 2017 (1548)	Gram-positive cocci (*Enterococcus* spp.- Vancomycin-resistant enterococci (VRE))	Single-site, quasi-experimental cohort study with simultaneous control over 3 months, with comparison to a 3-month pre-intervention period	1496 samples from 17 sites, including fomites (direct patient contact: mattress, bed clothes, bed rails) and patient surroundings (wall, curtains, ECG monitor. dining table, chart, cupboard) in 2 ICUs (MICU and SICU) in Wan Fang Medical Center, Taipei, Taiwan	Nanomaterial consisting of inorganic metal and organic quaternary ammonium (Bio-Kil, Cargico Group) vs control (untreated surface); cleaning of both intervention and control surfaces with 500-ppm sodium hypochlorite continued, per hospital protocol. Surfaces were sampled twice a week for 3 months after treatment. Sample collection relative to cleaning unspecified.	Percent reduction of prevalence (percent positive samples) of VRE was 56.6% (9.8% to 4.2%) for treated vs 118.1% (4.9% to 10.6%) for control, fomite surfaces(P=0.261). For patient surrounding surfaces, reductions were similar for treated (2.4% to 0%) and control (7.3% to 0%) rooms (p=0.135).
Tamimi 2014 (10851)	Gram-positive cocci (*Enterococcus* spp.- Vancomycin-resistant enterococci (VRE))	Single-site, quasi-experimental uncontrolled before-after over a 15-week period	6 sets of samples from 95 sites on surfaces in patient rooms (bed rails, bed controls, tray table, and wall), nurses station, and lobby (countertops, phones, computer keyboards, chair	Surface coating composed of quaternary ammonium silyl oxide and titanyl oxide moieties (ABS-G2015, Allied BioScience, Point Roberts, WA) vs baseline; cleaning of intervention surfaces continued with bleach and/or quaternary ammonium wipes. Surfaces were	Prevalence (percent positive sites) of VRE was 14% at baseline; 0 at weeks 1,2,4; 1% at week 8; and 0 at week 15. Significance not specified.

Reference (Study ID)	Outcome	Study Design	Setting	Intervention	Results
			armrests, end table) in an ICU in a community hospital in Los Angeles, CA	sampled at 1, 2, 4, 8, and 15 weeks after application of coating.	
Karunanayake 2019 (2035)	Gram-positive cocci (*Micrococcus* spp.)	Single-site, quasi-experimental cohort study with simultaneous control over 12 weeks	5 surfaces in an ICU (telephone, bedrail, doorknob, nurse's table, stethoscope) and 1 surface (bedrail)[a] in a general medicine ward in Base Hospital Homagama, Sri Lanka (identical type and number of coated and uncoated surfaces)	Silicon nano-coating surface (Bacterlon, Nanopool GmbH) vs. control (uncoated surfaces); cleaning of both intervention and control surfaces continued, with general purpose detergents (ward, ICU) and 70% alcohol (stethoscope, trolley), per hospital protocol. Surfaces were sampled once a week for 12 weeks after application of coating	Frequency of isolated *Micrococcus* spp did not show significant reduction on coated surfaces (n=3) compared to uncoated surfaces (n=10).
Lee 2017 (1548)	Gram-positive cocci (*Staphylococcus aureus*-MRSA)	Single-site, quasi-experimental cohort study with simultaneous intervention and control over 3 months, with comparison to a 3-month pre-intervention period	1496 samples from 17 sites, including fomites (direct patient contact: mattress, bed clothes, bed rails) and patient surroundings (wall, curtains, ECG monitor. dining table, chart, cupboard) in 2 ICUs (MICU and SICU) in Wan Fang Medical Center, Taipei, Taiwan	Nanomaterial consisting of inorganic metal and organic quaternary ammonium (Bio-Kil, Cargico Group) vs control (untreated surface); cleaning of both intervention and control surfaces with 500-ppm sodium hypochlorite continued, per hospital protocol. Surfaces were sampled twice a week for 3 months after treatment. Sample collection relative to cleaning unspecified.	Percent reduction of prevalence (percent positive samples) of MRSA on fomite surfaces was 100% (2.4% to 0%) for treated vs 78.2% (9.8% to 2.1%) for control (P=0.261). For patient surrounding surfaces reductions were similar for treated (4.9% to 0%) and control (4.9% to 0%) rooms (p=0.333)
Karunanayake 2019 (2035)	Gram-positive cocci (*Staphylococcus aureus*-MRSA)	Single-site, quasi-experimental cohort study with simultaneous	5 surfaces in an ICU (telephone, bedrail, doorknob, nurse's table, stethoscope) and 1 surface (bedrail)[a] in a general	Silicon nano-coating surface (Bacterlon, Nanopool GmbH) vs. control (uncoated surfaces); cleaning of both intervention and control surfaces continued, with general purpose detergents	Frequency of isolated MRSA did not show significant reduction on coated surfaces (n=5) compared to uncoated surfaces (n=3).

Reference (Study ID)	Outcome	Study Design	Setting	Intervention	Results
		control over 12 weeks	medicine ward in Base Hospital Homagama, Sri Lanka (identical type and number of coated and uncoated surfaces)	(ward, ICU) and 70% alcohol (stethoscope, trolley), per hospital protocol. Surfaces were sampled once a week for 12 weeks after application of coating	
Tamimi 2014 (10851)	Gram-positive cocci (*Staphylococcus aureus*-MRSA)	Single-site, quasi-experimental uncontrolled before-after over a 15-week period	6 sets of samples from 95 sites on surfaces in patient rooms (bed rails, bed controls, tray table, and wall), nurses station, and lobby (countertops, phones, computer keyboards, chair armrests, end table) in an ICU in a community hospital in Los Angeles, CA	Surface coating composed of quaternary ammonium silyl oxide and titanyl oxide moieties (ABS-G2015, Allied BioScience, Point Roberts, WA) vs baseline; cleaning of intervention surfaces continued with bleach and/or quaternary ammonium wipes. Surfaces were sampled at 1, 2, 4, 8, and 15 weeks after application of coating	Prevalence (percent positive sites) of MRSA was 7% at baseline and 0 through week 15
Thom 2014 (4132)	Gram-positive cocci (*Staphylococcus aureus*-MSSA, MRSA)	Single site, controlled cohort study over 2 months	343 samples from surfaces (sink basin, counter, call button, bedside table, bedside patient vital signs monitor, telephone, supply cart, door handle, floor) in 10-bed adult surgical intermediate care unit at university hospital. Maryland, USA.	Prior to treatment application, all rooms were cleaned and HPV was used to decontaminate surfaces. The silicone quaternary amine antimicrobial surface polymer (MSDS Poly, active ingredient 3-(trimethoxysily)-propyl dimethyl actadecyl ammonium chloride) was applied to all surfaces (e.g. floors, walls, equipment, furniture) in five treated rooms using electrostatic sprayer. Five control rooms did not receive the treatment. Samples were collected 2 days per week from all rooms with patient occupancy. Sample collection	There was no difference (p=0.53) between percent rooms positive with 40% (8/20) in treated rooms and 26% (6/23) in untreated rooms.

Reference (Study ID)	Outcome	Study Design	Setting	Intervention	Results
				relative to cleaning protocol not specified.	
de Jong 2018 (346)	Gram-positive cocci (*Staphylococcus aureus*)	Single-site, quasi-experimental over 12 weeks: 2-week wash-in, 4-week control measurement (pre-intervention), 2-week wash-in, 4 week-intervention measurement	192 sets of samples from 6 high-touch, near-patient surfaces (wall, floor, bed rail, door and door handle, ceiling, computer keyboard, bedside table, monitor arm, medical equipment cabinet) in 4 isolation rooms in a Level III ICU in university-affiliated Gelderse Vallei Hospital in Ede, The Netherlands	Titanium dioxide coating (Miracle Titanium MVX, Maeda Kougyou, Kitakyushu, Japan) vs. uncoated surfaces (pre-intervention); cleaning of both intervention and control surfaces with alcohol-based disinfectants continued, per hospital protocol. Surfaces were sampled at baseline (after wash-in, before coating) then once a week during the 4-week intervention period	Mean number of CFUs per room was 116 CFUs pre-intervention vs. 65 CFUs post-intervention; mean ratio/room of CFUs post- vs. pre-intervention (SD): 0.71 (0.38); Significance not reported.
Reid 2018 (3071)	Gram-positive cocci (*Staphylococcus aureus*)	Single-site, quasi-experimental cohort study with simultaneous control over 12 weeks	102 samples from high-touch surfaces on beds (rails, control panel, table, floorboard, locker) in a general medicine ward and a stroke unit (control surfaces only) in New Cross Hospital, Wolverhampton, UK	Titanium dioxide-based (1.5% Titania and 0.1% silver zeolite diluted solution) photocatalytic coating (MVX Hitech Co. Ltd, Kitakyushu, Japan) vs. control (uncoated surfaces); cleaning of both intervention and control surfaces with alcohol-based detergents continued, per hospital protocol. Surfaces were sampled twice a week for 12 weeks after application of coating. Sample collection relative to cleaning unspecified.	Low recovery of *S. aureus* (~10% surfaces positive). A total of 97 isolates recovered from 635 treated surfaces vs. 68 isolates from 655 control surfaces. Significance not reported.
Afinogenova 2017 (67)	Gram-positive cocci (*Staphylococcus* spp.)	Single-site, cohort study with simultaneous control and intervention	Floors in 2 treatment rooms of research hospital in Russia	Probiotics-based cleaning products compared to unspecified conventional disinfection methods	Minimal growth during study period

Reference (Study ID)	Outcome	Study Design	Setting	Intervention	Results
		over 35 days; partially seeded (*E. faecalis*)			
Kim 2018 (12022)	HAI (HAI-MDR *Acinetobacter baumannii*)	Single site, prospective cohort study, over 5 months	621 patients included who were admitted to medical ICU (excluding patients hospitalized < 72 h and < 18 y) Photocatalyst on high-touch surfaces and walls at 630-bed secondary care teaching hospital. South Korea.	5-month pre-intervention compared to 5-month post-intervention. High-touch surfaces coated with titanium dioxide-based photocatalyst. Standard cleaning of high-touch surfaces with chlorhexidine cloth and floors with detergent or sodium hypochlorite when known VRE or CDAD patient.	MDR A. baumannii also did not decrease significance (after at 3.09 from 3.20/1000 patient-days before) p=0.76
Kim 2018 (12022)	HAI (HAI-MRSA)	Single site, prospective cohort study, over 5 months	621 patients included who were admitted to medical ICU (excluding patients hospitalized < 72 h and < 18 y) Photocatalyst on high-touch surfaces and walls at 630-bed secondary care teaching hospital. South Korea.	5-month pre-intervention compared to 5-month post-intervention. High-touch surfaces coated with titanium dioxide-based photocatalyst. Standard cleaning of high-touch surfaces with chlorhexidine cloth and floors with detergent or sodium hypochlorite when known VRE or CDAD patient.	Incidence rate reduced from 9.3/1000 patient-days before intervention to 2.6/1000 patient-days after (p=0.03). After adjusting for other variables, risk of acquiring MRSA was 0.37 (95% CI 0.14-0.99, p=0.04). Longer length of stay corresponded with higher risk of MRSA acquisition.
Kim 2018 (12022)	HAI (HAI-pneumonia, BSI, UTI, CDAD)	Single site, prospective cohort study, over 5 months	621 patients included who were admitted to medical ICU (excluding patients hospitalized < 72 h and < 18 y) Photocatalyst on high-touch surfaces and walls at 630-bed secondary care	5-month pre-intervention compared to 5-month post-intervention. High-touch surfaces coated with titanium dioxide-based photocatalyst. Standard cleaning of high-touch surfaces with chlorhexidine cloth and floors with detergent or sodium hypochlorite when known VRE or CDAD patient.	Significant reduction in incidence of hospital-acquired pneumonia from 16.12/1000 before to 7.70/1000 patient-days after p=0.03. Acquisition during intervention compared to baseline was 0.47 (95% CI: 0.23-0.94, p=0.03). No statistically significant reduction in BSI, UTI, CDAD observed.

Reference (Study ID)	Outcome	Study Design	Setting	Intervention	Results
			teaching hospital. South Korea.		
Kim 2018 (12022)	HAI (HAI-VRE)	Single site, prospective cohort study, over 10 months	621 patients included who were admitted to medical ICU (excluding patients hospitalized < 72 h and < 18 y) Photocatalyst on high-touch surfaces and walls at 630-bed secondary care teaching hospital. South Korea.	51month pre-intervention compared to 5-month post-intervention. High-touch surfaces coated with titanium dioxide-based photocatalyst. Standard cleaning of high-touch surfaces with chlorhexidine cloth and floors with detergent or sodium hypochlorite when known VRE or CDAD patient.	VRE incidence was low across the study with no significant reduction after 0.62 compared to before at 1.28/1000 patient-days. p=0.54
Lee 2017 (1548)	HAI (Healthcare-associated infections (HAIs): sepsis)	Single-site, quasi-experimental cohort study with simultaneous control over 3 months, with comparison to a 3-month pre-intervention period	1496 samples from 17 sites, including fomites (direct patient contact: mattress, bed clothes, bed rails) and patient surroundings (wall, curtains, ECG monitor. dining table, chart, cupboard) in 2 ICUs (MICU and SICU) in Wan Fang Medical Center, Taipei, Taiwan	Nanomaterial consisting of inorganic metal and organic quaternary ammonium (Bio-Kil, Cargico Group) vs control (untreated surface); cleaning of both intervention and control surfaces continued with 500-ppm sodium hypochlorite, per hospital protocol. Surfaces were sampled twice a week for 3 months after treatment. Sample collection relative to cleaning unspecified.	Percent change in incidence of new-onset sepsis pre vs post intervention was -28.5% (incidence fell from 33.3% to 23.8%; P<0.001) in intervention rooms and +63.6% (incidence increased from 25% to 40.9%; P<0.001) in control rooms; P=0.232 for intervention vs control in post-intervention period (23.8% vs 40.9%)

Vapors

Table S19: Study results for vaporized hydrogen peroxide interventions ordered by outcome organism

Reference (Study ID)	Outcome	Study Design	Setting	Intervention	Results
Ali 2016 (521)	All viable organisms	Single-site, Quasi-experimental controlled before-after study, time period unspecified	220 samples collected from high frequency touch surfaces (bed frame, footboard, patient chair arm)in 10 isolation rooms at University of London Hospital, London, UK	Compared 2 whole-room hydrogen peroxide decontamination systems after terminal clean with 1000 ppm peracetic acid: (1) 30% hydrogen peroxide heated to 130º C (Bioquell Q10). (2) 4.9% Hydrogen peroxide solution (2 h cycle time, Deprox, Hygiene Solutions) Contact time was 2 – 2.5 h. All surfaces were sampled before exposure to hydrogen peroxide and immediately after HPV decontamination.	Percent surfaces positive for bacteria after HPV method 1 and 2 compared to before HPV was 50.2% (109/217) and 49.5% (106/215) compared to 96% (414/431), respectively. Median CFU of all surfaces decreased from 21.0 CFU/25 cm2 to 0.25 CFU/25 cm2 and from 28.0 CFU/25 cm2 to 0.5 CFU/25 cm2 after HPV methods 1 and 2, respectively (significance not specified). No difference between two HPV methods (p>0.05).
Blazejewski 2015 (766)	All viable organisms	Single-site, quasi-experimental, controlled crossover over a 3-month period	A total of 546 samples were taken from 8 surfaces (lateral part of mattress, ventilator, monitor, underside of overbed table, room door handle, sink, bedrail, keyboard, storage box) in 182 rooms in 5 medical and surgical ICUs of a university hospital. Lille, France.	Crossover trial compared two HPV technologies with the same terminal cleaning. Terminal cleaning consisted of a low-alcohol QAC for floors once daily and other surfaces twice daily (Aniosurf). After terminal cleaning, HPV (30% liquid H2O2, 30-minute contact time, 1 h 40 min cycle time) or aerosolized hydrogen peroxide (7% H2O2, 0.25% peracetic acid, 30% acetic acid, 30 min contact time, 3 h cycle time) was implemented. Time until measurement was "after terminal cleaning" and "after H2O2" disinfection.	There was a significant reduction in percent surfaces positive for at least one bacterium from 70 (38%) before H2O2/after terminal cleaning to 10 (5%) after H2O2 (p<0.001). After vaporized or aerosolized H2O2, there was a significant reduction compared to routine terminal cleaning with 10 (5%) surfaces positive. The H2O2 technologies were not significantly different. Crossover period was 6 weeks for each H2O2 technology than rooms were inverted.
Humayun 2019 (1316)	All viable organisms	Single-site, quasi-experimental before-after, uncontrolled study over one year	600 samples from high-touch, surfaces (bedside table, bed, bed rail, mattress, sink, etc.) in 20 rooms of ICU, general medical	Samples collected at the same time after patient discharge, after terminal cleaning (1:10 hypochlorite three times daily), and after HPV (Bioxeco, 100 ppm, 12.5% hydrogen peroxide, 2 h cycle time).	Proportion of rooms contaminated with one or more bacteria decreased from 19/20 (95%) after patient discharge to 16/20 (80%) after terminal cleaning to 2/20 (10%) after HPV intervention. HPV significantly lowered number of rooms with

Reference (Study ID)	Outcome	Study Design	Setting	Intervention	Results
			ward, operating rooms in hospital. Arar, Saudi Arabia		positive sample (70% reduction, p<0.001).
Mosci 2017 (1886)	All viable organisms	Multi-site, Controlled before-after cohort study over 9 months	448 samples were collected from 28 rooms (medicine, orthopedics, long-term care, recovery and functional rehabilitation) 4 public and private health facilities in Emilia-Romaga Region, Italy	Rooms randomized to terminal cleaning with 0.5% hypochlorite or with HPV (99MS system, < 8% hydrogen peroxide concentration and silver ions, 130-minute cycle time). All rooms received standard cleaning to remove visible dirt prior to disinfection intervention. Samples were taken before and after intervention.	Number of rooms positive for bacteria significantly decreased from 7 (50%) to 0 (0%) with HPV and from 11 (79%) to 2 (14%) with hypochlorite. Number of samples positive decreased from 13/112 (13%) to 0/112 (0%) with HPV vs. 22/112 (20%) to 3/112 (3%) with hypochlorite. Methods were similar (p=0.497).
Oon 2020 (2254)	All viable organisms	Single-site, uncontrolled before-after study over a 4 month period	429 samples collected from high-touch sites within patient bed zone (bed rail, overbed table, bed end, hand basin tap handle, IV pole, monitor button, toilet flush button) in five bed areas in 10-bed critical care unit at 160-bed rural hospital. New South Wales, Australia.	Standard cleaning consisted of detergent, NaDCC for patients with *C. difficile*, or alcohol wipes. Intervention with continuous dilute hydrogen peroxide (CIMR Tech) at 0.02 ppm hydrogen peroxide gas in ventilation system. Samples were collected for five consecutive days during a control period 4 weeks after DHP had been turned off, an intervention period after DHP had been turned on for 4 weeks, and a second control period 4 weeks after DHP had been turned off. Samples were collected 21-23 h after routine cleaning or 6-29 h after discharge clean.	Failure consisted of surfaces with aerobic colony counts> 2.5 CFU/cm2. During the intervention period there were 7.7% samples with failure compared to 2.2% in the first control phase and 3.4% in the second control phase. Significance not assessed.
Popov 2016 (2592)	All viable organisms	Single site, uncontrolled before-after study	60 samples from working surfaces (furniture, equipment, table, drug box, drip stand, shadowless lamp, containers,	Disinfection was conducted after routine cleaning without patients or personnel (GLOSAIR 400, 5-6% hydrogen peroxide with 50 ppm silver cations, ~3 h cycle time). Samples were collected before and	Percent (number) of objects positive for bacteria before decontamination was 83% (25/30) compared to 37% (11/30) after decontamination (p=0.0006).

Reference (Study ID)	Outcome	Study Design	Setting	Intervention	Results
			dispensers for soap, sinks, etc.) in cardiovascular surgical dressing room or patient ward at hospital. Russia.	after the completion of the working cycle.	
Singh 2017 (4519)	All viable organisms	Single-site quasi experimental, controlled before-after study	27 surface samples (wall, bed) from three high risk areas (ICU, HDU, and isolation rooms) at hospital. Amritsar, India	Fogging was conducted with three different disinfectants: (1) 20% solution for fogging (hydrogen peroxide-10%, silver solution-0.01%); (2) 0.39% QAC solution (Octyldecyl-dimethyl-ammonium-chloride 6.5%, dioctyldimethyl-ammonium-chloride 2.6%, dodecyl-dimethylammonium-chloride 3.9%, alkylkimethyl-benzylammonium-chloride 8.7%); (3) 1% QAC solution (N-alkyldimethyl-benzylammonium chloride-13.6%, didecyldimethyl-ammonium-chloride 13%, polymeric biguanide hydro-chloride 5%). Also compared efficacy with and without pre-cleaning with detergent.	All samples were positive for bacteria prior to disinfection compared to 44% with disinfectant 1, 33% with disinfectant 2, and 67% with disinfectant 3. Disinfectants 1 and 2 had significantly lower ($p<0.05$) number of samples positive. 100% reduction in bacterial concentration with disinfectants 1 (initial= 16 CFU) and 2 (initial=52 CFU), 95% reduction in bacterial concentration with disinfectant 3 (initial=6 CFU) when pre-cleaning was conducted. % reduction is lower without pre-cleaning
Havill 2012 (5792)	All viable organisms	Single site, controlled before-after study over two months	150 samples were collected from 5 high-touch surfaces (bedside rail, overbed table, television remote, bathroom grab bar, toilet seat) from 15 patient rooms on 8 wards in 500-bed teaching hospital. Connecticut, USA	Rooms were cleaned with QAC (Virex 256) or 10% bleach wipe (Dispatch) and bathroom doors opened prior to decontamination. Then, HPV (Bioquell) decontamination conducted converting 30% hydrogen peroxide liquid into HPV over average of 153 minutes. Samples were collected before and after (unspecified time) decontamination. HPV decontamination was compared to UVC (Tru-D, 22,000 microW sec/cm2) decontamination concurrently.	Concentration was significantly lower after room decontamination ($p<0.001$). Range of mean before decontamination was 12.0-53.4 CFU/plate compared to 0.1-0.3 CFU/plate after decontamination. Percent surfaces negative was significantly ($p<0.001$) higher after HPV decontamination at 93% compared to before at 7%. After decontamination, five sites were positive ranging from 1-4 CFU/plate. HPV decontamination had significantly fewer sites positive for

Reference (Study ID)	Outcome	Study Design	Setting	Intervention	Results
					bacteria after decontamination compared to UVC (p<0.0001)
Chan 2011 (6199)	All viable organisms	Single site, uncontrolled before-after study	Samples from four high-touch surfaces (toilet flush button, bedside table, handset) in each of four rooms in unspecified wards at 300-bed teaching hospital. Melbourne, Australia.	Terminal cleaning consisted of neutral detergent followed by hypochlorite product (either 500ppm bleach or NaDCC-Det-Sol 500) or by HPV with dry hydrogen vapor room decontamination spray (Nocospray). Samples were collected after patient discharge, after detergent cleaning, and after HPV disinfection. Samples were also collected after detergent + chlorine terminal disinfection (but not collected before).	After HPV, 33% of surfaces were not positive for bacteria and counts were all < 3 CFU/cm2 (median=1 CFU/cm2). Total counts after detergent cleaning ranged between 11 and 531 CFU. Significance not specified.
Taneja 2011 (7829)	All viable organisms	Single site, uncontrolled before-after study, over ~12 hours	252 samples from 126 surfaces (floor, trolley, cabinet, door handle, air-conditioning grill, electrical switch, mattress, IV stand, etc.) in clinical area and rooms in emergency medical and surgical wards and other wards in emergency complex. India.	Air ducts were contaminated with MRSA. Rooms were washed with detergent and water. Air duct openings were sealed. Fogging conducted with 115% w/v hydrogen peroxide with 0.015% silver nitrate (Ecoshield, Fogmaster ULV 2401). Samples were collected before and after fogging.	All post-fogging samples had 99.7-100% reduction in counts with average of initial counts ranging from 2.05-6.52 log10 CFU depending on surface type.
Barbut 2013 (12491)	All viable organisms	Single-site, Quasi-experimental, before and after uncontrolled study, 3 years	165 samples from 28 high-touch surfaces (electric rail, bedside table, dining table, sink, plinth, bathtub, bench, etc.) in burns unit (10 single-bed rooms, operating theater, treatment	HPV disinfection of entire unit and also patient rooms (concentration not specified, ~1.5 h cycle time). Standard cleaning included detergent disinfectant. Surface samples were taken after standard cleaning but before HPV and after HPV (time not specified)	Average bacterial surfaces count decreased from 2.9 CFU/100 cm^2 to 0.1 in patient rooms (p<0.001) and from 4 CFU/100 cm^2 to 0.7 in burns unit after HPV (p<0.02).

Reference (Study ID)	Outcome	Study Design	Setting	Intervention	Results
			room) at hospital. Paris, France		
Hardy 2007 (14089)	All viable organisms	Single site, uncontrolled before-after study, over five months	Unspecified number of samples were collected (2 from each bedspace) from environmental surfaces (underside of bed frame, workstation alongside bed) in 9-bedded open-plan ICU at a hospital. Birmingham, UK.	Baseline environmental sampling was conducted for three months prior to deep clean intervention. All patients were removed from ICU prior to deep clean. Terminal clean consisted of washing walls, surfaces, and equipment with detergent and water. HPV (Bioquell) was run overnight at up to 280 ppm. Samples were collected immediately before patient removal, immediately after terminal cleaning, 1 h before HPV, immediately after HPV but before patient readmission, and 24 h and 48 h after patient re-admission. Additional samples were collected weekly for 8 weeks.	Before the intervention, mean count underneath bed was between ~150 - 230 CFU and mean count for workstations was ~25-75 CFU. Counts were reduced to < 10 CFU following HPV and increased somewhat at 24 h and 48 h to ~ 30 CFU underneath bed and ~15 CFU for workstations. Significance not specified.
Blazejewski 2015 (766)	All viable organisms (MDRO-ESBL gram negative, MRSA, IRAB, resistant *P. aeruginosa*)	Single-site, quasi-experimental, controlled cohort study over a 3-month period	A total of 546 samples were taken from 8 surfaces (lateral part of mattress, ventilator, monitor, underside of overbed table, room door handle, sink, bedrail, keyboard, storage box) in 182 rooms in 5 medical and surgical ICUs of a university hospital. Lille, France.	Crossover trial compared two HPV technologies with the same terminal cleaning. Terminal cleaning consisted of a low-alcohol QAC for floors once daily and other surfaces twice daily (Aniosurf). After, HPV (30% liquid H2O2, 30-minute contact time, 1 h 40 min cycle time) or aerosolized hydrogen peroxide (7% H2O2, 0.25% peracetic acid, 30% acetic acid, 30 min contact time, 3 h cycle time) was implemented. Time until measurement was "after terminal cleaning" and "after H2O2" disinfection.	2 (0.13%) MDRO (ESBL gram-negative rods, MRSA, IRAB, Resistant P. *aeruginosa*) samples identified on room surfaces after H2O2 intervention was significantly lower (p<0.001) compared to 14 (0.96%) after terminal cleaning. Routine terminal cleaning did not have a significant reduction (2%, p=0.371) in number of rooms positive for MDRO compared to reduction after HPV (5.5%, p=0.004). H2O2 technologies were not significantly different. Reductions were primarily due to detection of ESBL organisms (low prevalence of MRSA, IRAB, and resistant P. *aeruginosa* prior to terminal cleaning)

Reference (Study ID)	Outcome	Study Design	Setting	Intervention	Results
Passaretti 2013 (2322)	All viable organisms (MDRO-MRSA, VRE, MDR GNR, *C. difficile*)	Single-site, quasi-experimental controlled study over 9 months	1039 room surfaces (bedrail, keyboard, monitoring equipment in patient room) in 6 high-risk units (ICU, surgical unit) at 994-bed tertiary referral hospital. Baltimore, USA	3-month pre-intervention phase followed by 6-month intervention phase with HPV (Bioquell, no specific concentration, 1.5 – 3 h) after standard cleaning on 3 units compared to standard cleaning alone with quaternary ammonium compound (active ingredient not specified, 3M) on 3 units. Samples were taken monthly.	Significant reduction for patient rooms positive for > 1 MDRO in HPV units during intervention compared to non-HPV units (relative risk 0.65, p=0.03)
Singh 2017 (4519)	Fungi	Single-site quasi experimental, controlled before-after study	27 surface samples (wall, bed) from three high risk areas (ICU, HDU, and isolation rooms) at hospital. Amritsar, India	Fogging was conducted with three different disinfectants: (1) 20% solution for fogging (hydrogen peroxide-10%, silver solution-0.01%); (2) 0.39% QAC solution (Octyldecyl-dimethyl-ammonium-chloride 6.5%, dioctyldimethyl-ammonium-chloride 2.6%, didecyldimethyl-ammonium-chloride 3.9%, alkyl-kimethylbenzylammonium-chloride 8.7%); (3) 1% QAC solution (N-alkyldimethyl-benzylammonium chloride-13.6%, didecyldimethyl-ammonium-chloride 13%, polymeric biguanide hydro-chloride 5%). Also compared efficacy with and without pre-cleaning with detergent.	100% reduction in fungal concentration with disinfectants 1 (from 2 CFU to 0 CFU) and disinfectant 2 (from 3 CFU to 0 CFU). 50% reduction in fungal concentration with disinfectant 3 (from 4 CFU to 2 CFU) when pre-cleaning was conducted. % reduction is lower without pre-cleaning.
Barbut 2013 (12491)	Fungi	Single-site, Quasi-experimental, before and after uncontrolled study, 3 years	165 samples from 28 high-touch surfaces (electric rail, bedside table, dining table, sink, plinth, bathtub, bench, etc.) in burns unit (10 single-bed rooms, operating theater, treatment	HPV disinfection of entire unit and also patient rooms (concentration not specified, ~1.5 h cycle time). Standard cleaning included detergent disinfectant. Surface samples were taken after standard cleaning but before HPV and after HPV (time not specified)	Average fungal surfaces count decreased from 1 CFU/100 cm^2 to 0 in patient rooms (p<0.01) and from 3.5 CFU/100 cm^2 to 0 (p<0.001) in burns unit before compared to after disinfection with HPV

Reference (Study ID)	Outcome	Study Design	Setting	Intervention	Results
			room) at hospital. Paris, France		
Barbut 2013 (12491)	Fungi (*Aspergillus* spp.)	Single-site, Quasi-experimental, before and after uncontrolled study, 3 years	165 samples from 28 high-touch surfaces (electric rail, bedside table, dining table, sink, plinth, bathtub, bench, etc.) in burns unit (10 single-bed rooms, operating theater, treatment room) at hospital. Paris, France	HPV disinfection of entire unit and also patient rooms (concentration not specified, ~1.5 h cycle time). Standard cleaning included detergent disinfectant. Surface samples were taken after standard cleaning but before HPV and after HPV (time not specified)	Aspergillus percentage surfaces positive in burns unit decreased from 4.5% (3/66) after standard cleaning to 0 after HPV. No significance reported.
Lerner 2019 (1574)	Gram-negative bacteria (*Acinetobacter baumannii*-CRAB)	Single-site, quasi-experimental, controlled before-after study for a 6-month period	253 samples from environmental objects (bedrail, IV pole, bed linen, electrical outlet, infusion bottle hook, medical tray, medical trolley, arm chair, chair, curtain, doorknob, counter, cupboard shelf, monitor, IV pump, ventilator, stethoscope, hemodialysis machine, mattress pump, warming unit) in 7 single-patient rooms of known CRAB-carriers in the MICU at 1450-bed tertiary-care hospital. Tel Aviv, Israel.	Terminal cleaning was with manually-applied sodium hypochlorite in 3 rooms (concentration, contact time unspecified) compared to aerosolized hydrogen peroxide (aHP) in 4 rooms (GLOSAIRTM 400, unspecified concentration, cycle time). Samples were collected before and immediately after terminal disinfection.	Before disinfection, 41% (24/59) were positive compared to 6% (3/52) after disinfection with manual sodium hypochlorite, an 85% reduction. Before disinfection with aHP, 80% (59/74) samples were positive compared to 18% (12/68) after disinfection, a 78% reduction. Significance not specified.
Ray 2010 (10625)	Gram-negative bacteria	Single-site, quasi-experimental	93 samples from high-touch objects	Vaporized hydrogen peroxide intervention (VaproSure Steris, 240	Number of rooms positive decreased from 7 rooms (before intervention) to

Reference (Study ID)	Outcome	Study Design	Setting	Intervention	Results
	(*Acinetobacter baumannii*-MDR)	uncontrolled before-after study over 3 weeks.	(call bell, bedside table, bedrails) inside 2 wards with mostly single occupancy rooms at 54-bed long-term acute care hospital. Ohio, USA	ppm peak concentration, 90 min contact time, cycle time 8 h) measured immediately, 24 h, 1 week, 2 weeks, and 3 weeks after intervention.	0 room immediately after and 1 week after intervention. 1 room was positive 2 weeks after intervention and 2 rooms positive 3 weeks after intervention (significance not specified).
Manian 2011 (14130)	Gram-negative bacteria (*Acinetobacter baumannii* complex (ABC))	Single site, uncontrolled before-after study, over 5 years	7140 samples (bedside table, chair, TV, door, sink, bedrail, telephone, lift, cabinet, countertop, etc.) in 384 rooms from all wards at suburban 900-bed community teaching medical center. Missouri, USA.	Routine terminal cleaning and disinfection by rooms vacated by antibiotic-resistant ABC-positive patients consisted of disinfection with QAC followed by 0.525% sodium hypochlorite solution. During room occupation, at least daily disinfection was conducted with sodium hypochlorite. HPV (Bioquell) treatment was conducted following newly-vacated room following terminal disinfection with bleach.	After 4 rounds of bleach disinfection, 27% rooms (83/312) and 16% (51/5705) sites were positive for ABC or MRSA. After 1 round of bleach disinfection, there was a significant reduction in number of sites positive (n=700) for ABC (OR=0.25, 95% CI: 0.045-0.93, p=0.04). After 1 round of bleach disinfection and addition of HPV, there was a significant reduction in ABC-positive sites (odds ratio=0, 95% CI: 0-0.08, p=0.04).
Barbut 2013 (12491)	Gram-negative bacteria (*Acinetobacter* spp.)	Single-site, Quasi-experimental, before and after uncontrolled study, 3 years	165 samples from 28 high-touch surfaces (electric rail, bedside table, dining table, sink, plinth, bathtub, bench, etc.) in burns unit (10 single-bed rooms, operating theater, treatment room) at hospital. Paris, France	HPV disinfection of entire unit and also patient rooms (concentration not specified, ~1.5 h cycle time). Standard cleaning included detergent disinfectant. Surface samples were taken after standard cleaning but before HPV and after HPV (time not specified)	*Acinetobacter* percent surfaces positive in patient rooms decreased from 1% (1/102) at 4 CFU/100 cm^2 to 0. No significance reported.
Garvey 2016 (1096)	Gram-negative bacteria (Coliforms-carbapenemase producing organisms)	Single site, uncontrolled before-after study over a period < 1 week	30 samples were collected from macroscopically clean touch-points in vicinity of patient (bed frame, ventilator, drip stand,	Decontamination of a burns shock room was described using standard terminal cleaning with 1000 ppm hypochlorite (NaDCC Chlor Clean) followed by hydrogen peroxide misting (6%). A modified second clean comprised steam-cleaning,	After terminal disinfection with 1000 ppm NaDCC and 6% HPV, 24/30 samples remained positive for carbapenemase-producing coliforms, however these organisms were not recovered from any surface after

Reference (Study ID)	Outcome	Study Design	Setting	Intervention	Results
			extract vent, floor) and in communal areas (trolley, sink tap, sink, window sill, door handle, etc.) in a burns unit at a university NHS Foundation Trust tertiary referral teaching hospital in Birmingham, UK.	2000 ppm hypochlorite (NaDCC Chlor Clean) and hydrogen peroxide misting (12%). Samples were collected after the first terminal clean and after the second enhanced clean.	increasing concentrations of NaDCC to 2000 ppm and 12% HPV.
Bates 2005 (12952)	Gram-negative bacteria (Coliforms)	Single-site, Quasi-experimental, before and after uncontrolled study over 2 months	66 samples were taken from incubator surfaces in two rooms of the neonatal ICU at a General and Teaching Hospital, Sheffield, UK	An environmental survey was conducted pre-HPV. In addition to standard cleaning with detergent sanitizer, implemented HPV 30% vaporized liquid hydrogen peroxide (Bioquell) overnight (cycle time not stated). Samples taken before and after HPV cycle overnight (time not specified).	0 (0%) sites contaminated with Coliforms after HPV intervention compared to 7 (17%) after detergent sanitizer, no significance reported
Blazejewski 2015 (766)	Gram-negative bacteria (ESBL gram-negative bacilli)	Single-site, quasi-experimental, controlled cohort study over a 3-month period	A total of 546 samples were taken from 8 surfaces (lateral part of mattress, ventilator, monitor, underside of overbed table, room door handle, sink, bedrail, keyboard, storage box) in 182 rooms in 5 medical and surgical ICUs of a university hospital. Lille, France.	Crossover trial compared two HPV technologies with the same terminal cleaning. Terminal cleaning consisted of a low-alcohol QAC for floors once daily and other surfaces twice daily (Aniosurf). After, HPV (30% liquid H_2O_2, 30-minute contact time, 1 h 40 min cycle time) or aerosolized hydrogen peroxide (7% H_2O_2, 0.25% peracetic acid, 30% acetic acid, 30 min contact time, 3 h cycle time) was implemented. Time until measurement was "after terminal cleaning" and "after H_2O_2" disinfection.	2 (0.13%) samples identified on room surfaces were positive for ESBL gram-negative bacilli after H_2O_2 intervention and significantly lower ($p<0.001$) compared to 14 (0.96%) samples identified on room surfaces after terminal cleaning.
Singh 2017 (4519)	Gram-negative bacteria (*Escherichia coli*)	Single-site quasi experimental,	27 surface samples (wall, bed) from three high risk areas	Fogging was conducted with three different disinfectants: (1) 20% solution for fogging (hydrogen	Surfaces positive for *Pseudomonas* before compared to after disinfection was from 11% to 0% with disinfectant

Reference (Study ID)	Outcome	Study Design	Setting	Intervention	Results
		controlled before-after study	(ICU, HDU, and isolation rooms) at hospital. Amritsar, India	peroxide-10%, silver solution-0.01%); (2) 0.39% QAC solution (Octyldecyl-dimethyl-ammonium-chloride 6.5%, dioctyldimethyl-ammonium-chloride 2.6%, didecyldimethyl-ammonium-chloride 3.9%, alkyl-kimethylbenzylammonium-chloride 8.7%); (3) 1% QAC solution (N-alkyldimethyl-benzylammonium chloride-13.6%, didecyldimethyl-ammonium-chloride 13%, polymeric biguanide hydro-chloride 5%).	1, from 0% to 0% with disinfectant 2, and from 33% to 0% with disinfectant 3.
Barbut 2013 (12491)	Gram-negative bacteria (*Escherichia coli*)	Single-site, Quasi-experimental, before and after uncontrolled study, 3 years	165 samples from 28 high-touch surfaces (electric rail, bedside table, dining table, sink, plinth, bathtub, bench, etc.) in burns unit (10 single-bed rooms, operating theater, treatment room) at hospital. Paris, France	HPV disinfection of entire unit and also patient rooms (concentration not specified, ~1.5 h cycle time). Standard cleaning included detergent disinfectant. Surface samples were taken after standard cleaning but before HPV and after HPV (time not specified)	*E. coli* percentage surfaces positive in patient rooms decreased from 1% (1/102) at 1 CFU/100 cm^2 to 0 (not detected, not compared to infection control bundle). No significance reported.
Otter 2007 (13449)	Gram-negative bacteria (Gentamicin-resistant Gram-negative rod (GNR))	Single-site, quasi-experimental, uncontrolled before-after study for 19 days	90 samples were taken from 15 sites (floor, beside the bed, floor corner, bed-frame, bed-elevation control panel, bedside chair, bedside locker, over-bed table, remote control, door handle, etc.) in one room in a 500-bed teaching hospital. London, UK.	Terminal cleaning included a QAC disinfectant-detergent (HP800, PVA Hygiene Ltd, Weston-super-Mare, Somerset, UK). HPV was implemented as an adjunct decontamination in one room (Bioquell, 30 min at 20 g/min for two cycles, peak HPV 530-540 ppm). Sampling was taken before and after terminal cleaning and after HPV decontamination.	Number (percent) surfaces positive before vs. after terminal cleaning vs. after HPV: 9 (30%) vs. 3 (10.0%) vs. 0. GNR remained undetected after 1, 2, 5, and 6 days after HPV. Most of the GNR cultured were *Acinetobacter* spp or *Klebsiella* spp. Significance not assessed.

Reference (Study ID)	Outcome	Study Design	Setting	Intervention	Results
Otter 2010 (10984)	Gram-negative bacteria (Gram-negative rods-MDR)	Single-site, Quasi-experimental, uncontrolled before-after study over six months	84 samples from all hand-contact surfaces (bed rail, medical equipment) adjacent to bed from 12-bed, 3-room ICU at a hospital. Ede, The Netherlands	Condensing HPV decontamination (Bioquel) of the whole unit was conducted after cleaning with 2000 ppm sodium hypochlorite or 70% alcohol wipes. Decontamination took ~ 12 h. Samples were taken after cleaning but before HPV and after HPV.	Number of areas positive for gram-negative rods decreased from 10 out of 21 (47.6%) to 0 out of 63 (0%) after HPV compared to before (significance not specified).
Ali 2016 (521)	Gram-negative bacteria (*Klebsiella pneumonia*)	Single-site, Quasi-experimental controlled before-after study, time period unspecified	220 samples collected from high frequency touch surfaces (bed frame, footboard, patient chair arm)in 10 isolation rooms at University of London Hospital, London, UK	Compared 2 whole-room hydrogen peroxide decontamination systems after terminal clean with 1000 ppm peracetic acid: (1) 30% hydrogen peroxide heated to 130° C (Bioquell Q10). (2) 4.9% Hydrogen peroxide solution (2 h cycle time, Deprox, Hygiene Solutions) Contact time was 2 – 2.5 h. All surfaces were sampled before exposure to hydrogen peroxide and immediately after HPV decontamination. Coupons inoculated with 10^{6} CFU bacterial suspension and placed on surfaces prior to decontamination. Coupons removed after decontamination and compared to non-exposed (control) coupons.	~6.3 log10-reduction with HPV compare to non-exposed. No difference in efficacy by decontamination system ($p>0.05$).
Singh 2017 (4519)	Gram-negative bacteria (*Klebsiella* spp.)	Single-site quasi experimental, controlled before-after study	27 surface samples (wall, bed) from three high risk areas (ICU, HDU, and isolation rooms) at hospital. Amritsar, India	Fogging was conducted with three different disinfectants: (1) 20% solution for fogging (hydrogen peroxide-10%, silver solution-0.01%); (2) 0.39% QAC solution (Octyldecyl-dimethyl-ammonium-chloride 6.5%, dioctyldimethyl-ammonium-chloride 2.6%, didecyldimethylammonium-chloride 3.9%, alkyl-kimethylbenzylammonium-chloride 8.7%); (3) 1% QAC solution (N-	Surfaces positive for *Klebsiella* had 67% reduction from 56% to 33% surfaces positive with disinfectant 1, 46% reduction from 89% to 22% surfaces positive with disinfectant 2, and 0% reduction from 67% to 67% surfaces positive with disinfectant 3.

Reference (Study ID)	Outcome	Study Design	Setting	Intervention	Results
				alkyldimethyl-benzylammonium chloride-13.6%, didecyldimethyl-ammonium-chloride 13%, polymeric biguanide hydro-chloride 5%).	
Singh 2017 (4519)	Gram-negative bacteria (*Pseudomonas* spp.)	Single-site quasi experimental, controlled before-after study	27 surface samples (wall, bed) from three high risk areas (ICU, HDU, and isolation rooms) at hospital. Amritsar, India	Fogging was conducted with three different disinfectants: (1) 20% solution for fogging (hydrogen peroxide-10%, silver solution-0.01%); (2) 0.39% QAC solution (Octyldecyl-dimethyl-ammonium-chloride 6.5%, dioctyldimethyl-ammonium-chloride 2.6%, didecyldimethyl-ammonium-chloride 3.9%, alkyl-kimethylbenzylammonium-chloride 8.7%); (3) 1% QAC solution (N-alkyldimethyl-benzylammonium chloride-13.6%, didecyldimethyl-ammonium-chloride 13%, polymeric biguanide hydro-chloride 5%).	Surfaces positive for *E. coli* had 17% reduction from 33% to 0% surfaces positive with disinfectant 1, 4% reduction from 11% to 0% surfaces positive with disinfectant 2, and 0% reduction from 0% to 0% surfaces positive with disinfectant 3.
Bates 2005 (12952)	Gram-negative bacteria (*Serratia marcescens*)	Single-site, Quasi-experimental, before and after uncontrolled study over 2 months	66 samples were taken from incubator surfaces in two rooms of the neonatal ICU at a General and Teaching Hospital, Sheffield, UK	An environmental survey was conducted pre-HPV. In addition to standard cleaning with detergent sanitizer, implemented HPV 30% vaporized liquid hydrogen peroxide (Bioquell) overnight (cycle time not stated). Samples taken before and after HPV cycle overnight (time not specified).	0 (0%) sites contaminated with Serratia spp. after HPV intervention compared to 2 (5%) after detergent sanitizer, no significance reported
Andersen 2006 (6888)	Gram-positive bacilli (*Bacillus atrophaeus*)	Single-site, Quasi-experimental uncontrolled before-after study over one month	Spore strips placed on walls, tables, floors, etc. in 17 rooms in operating department at university hospital. Oslo, Norway	No pre-cleaning conducted prior to HPV (5% hydrogen peroxide, phosphoric acid <50 ppm, silver cations <50 ppm, gum Arabica < 1ppm, and biosmotic water 95%; Sterusil) with 0.5, 1 h, and 2 h cycle times. Biological indicator with known concentration (2.5e6 CFU/strip) placed in rooms and	48/48 biological indicator tests had no growth after 3 cycles, 6/6 positive after 2 cycles, and 12/12 positive after 1 cycle. No significance reported.

Reference (Study ID)	Outcome	Study Design	Setting	Intervention	Results
				removed 18-20 h after the last cycle to determine efficacy	
Havill 2012 (5792)	Gram-positive bacilli (*C. difficile*)	Single site, controlled before-after study over two months	75 spiked carrier disks with known concentration were placed on 5 surfaces (overbed table, chair, floor under bed, toilet seat, shower floor) in 15 patient rooms on 8 wards in 500-bed teaching hospital. Connecticut, USA	Rooms were cleaned with QAC (Virex 256) or 10% bleach wipe (Dispatch) and bathroom doors opened prior to decontamination. Then, HPV (Bioquell) decontamination conducted converting 30% hydrogen peroxide liquid into HPV over average of 153 minutes. Disk carriers with spores at concentration of 10^6 were placed in five sites in each of the rooms before decontamination. Carriers exposed to decontamination were compared to disks carriers unexposed to the decontamination process. HPV decontamination was compared to UVC (Tru-D, 22,000 microW sec/cm2) decontamination concurrently.	There was a 6-log reduction in *C. difficile* spores in all samples from all sites.
Doan 2012 (414)	Gram-positive bacilli (*Clostridium difficile*)	Single-site, quasi-experimental, controlled before-after cohort study over 3 months	53 samples collected per intervention from high frequency contact surfaces from hospital environment (bedrails, door handles, light switches, nurse call bell, toilet, bed table, floor) in isolation rooms at Derby Hospital Foundation Trust. Derby, UK	*C. difficile* inoculated into rooms for 72 h. Samples taken prior to disinfection. Disinfection interventions (HPV, dry ozone, 1000 ppm chlorine (sodium dichloroisocyanurate), dry atomized steam, steam cleaning, peracetic acid wipes) were tested each in separate rooms to determine concentration reduction of with known concentration of *C. difficile* spores placed in rooms. HPV (Bioquell Q10) at 350-700ppm, 255-minute cycle time). Measurements taken after "designated time period specified by company guidelines."	Median log10 reduction (interquartile range) was 2.3 CFU (1.9-2.3) and median concentration reduction was 200 CFU. Hydrogen peroxide vapor, 1000 ppm chlorine-releasing agent, and peracetic wipes were the most effective compared to the other interventions at 2.303, 2.223, and 2.134 log10 reduction respectively.

Reference (Study ID)	Outcome	Study Design	Setting	Intervention	Results
Ali 2016 (521)	Gram-positive bacilli (*Clostridium difficile*)	Single-site, Quasi-experimental controlled before-after study, time period unspecified	220 samples collected from high frequency touch surfaces (bed frame, footboard, patient chair arm)in 10 isolation rooms at University of London Hospital, London, UK	Compared 2 whole-room hydrogen peroxide decontamination systems after terminal clean with 1000 ppm peracetic acid: (1) 30% hydrogen peroxide heated to 130º C (Bioquell Q10). (2) 4.9% Hydrogen peroxide solution (2 h cycle time, Deprox, Hygiene Solutions) Contact time was 2 – 2.5 h. Coupons inoculated with 10^5 CFU spores and placed on surfaces prior to decontamination. Coupons removed after decontamination and compared to non-exposed (control) coupons.	5.1 log10-reduction in *C. difficile* observed with HPV relative to non-exposed. No difference in efficacy by decontamination system (p>0.05).
Barbut 2009 (686)	Gram-positive bacilli (*Clostridium difficile*)	Multisite, Quasi-experimental, controlled cohort study over 5 months	748 total samples collected from 12 high-touch surfaces (bathroom floor, bedside table, care table, door handle, windowsill, etc.) from 31 rooms following patient with CDI discharge at 2 university hospitals. Creteil, France	Terminal cleaning in rooms following discharge of patient with *C. difficile* infection. Patient randomized to either HPV (hydrogen peroxide, phosphoric acid < 50ppm, silver cations < 50ppm, gum Arabic <1 ppm, 95% biosmotic water; Sterinis-Sterusil), 1-hour contact time, or control group with sodium hypochlorite solution (0.5%, 5,000 ppm available chlorine). Before each, floors and surfaces cleaned with detergent and rinsed with water. Samples collected before cleaning and after hypochlorite dried or 1 h exposure for HPV.	% positive surfaces and rooms was significantly lower after compared to before disinfection. After hypochlorite, 12% (23/194) samples were positive (p<0.002) and 2% (4/180) surfaces were positive after HPV (p<0.001) compared to 21% (80/374) surfaces and 74% (23/31) rooms were positive before cleaning. Percent reduction of *C. difficile* positive samples was higher at 91% in HPV group vs 50% in hypochlorite group (p<0.005)
Boyce 2008 (813)	Gram-positive bacilli (*Clostridium difficile*)	Single-site, Quasi-experimental, uncontrolled before-after study over 10 months	80 samples from high-touch surfaces in bathroom, patient rooms, and open ward areas (bedrail, nurse call button, intravenous pumps, chair arm, dresser) in	Three of five high-incidence CDAD had ward decontamination with HPV. All wards had terminal decontamination of patient rooms with HPV. HPV consisted of 30 % hydrogen peroxide solution (Bioquell, 12 h cycle time for ward, 3-4 h cycle time per room). Samples	*C. difficile*-positive (> 6 CFU) samples decreased from 25.6% to 0% (p<0.001) after HPV.

Reference (Study ID)	Outcome	Study Design	Setting	Intervention	Results
			5 high-incidence wards at 500-bed hospital. Connecticut, USA	collected before and after HPV (time not specified).	
Mosci 2017 (1886)	Gram-positive bacilli (*Clostridium difficile*)	Multi-site, Controlled before-after cohort study over 9 months	448 samples were collected from 28 rooms (medicine, orthopedics, long-term care, recovery and functional rehabilitation) 4 public and private health facilities in Emilia-Romaga Region, Italy	Rooms randomized to terminal cleaning with 0.5% sodium hypochlorite or with HPV (99MS system, < 8% hydrogen peroxide concentration and silver ions, 130-minute cycle time). All rooms received standard cleaning to remove visible dirt prior to disinfection intervention. Samples were taken before and after intervention.	Percent of samples contaminated with *C. difficile* significantly decreased from 13% to 0% in HPV disinfection (p=0.002) and from 20% to 3% with hypochlorite (p=0.006). Methods were similar (p=0.267).
Shapey 2008 (2730)	Gram-positive bacilli (*Clostridium difficile*)	Single-sites, Quasi-experimental, uncontrolled before-after study over 3 months	406 samples collected from touch points (bedside table, bed frame, toilet, shelf, etc.) in 10 rooms from high risk elderly care wards. Nottingham, UK	Intervention was HPV (5% hydrogen peroxide, <50 ppm silver cations, <50 ppm orthophosphoric acid, Sterinis, 2 h cycle time) with traces of silver cations (<50 ppm) and orthophosphoric acid (<50 ppm) after standard terminal cleaning (detergent or 1% hypochlorite if prior occupant had *C. difficile*). Samples were taken before HPV but after standard terminal cleaning and after HPV.	Number and percentage of rooms positive for *C. difficile* decreased from 10 rooms (100%) to 5 rooms (50%) following HPV (p=0.033). Percent samples decreased from 48/203 (24%) to 7/203 (3%) after HPV (p<0.0001). Average (range) concentration per room decreased from 13.8 (1 – 33) to 0.8 (0 – 2) CFU per room (94% reduction).
Yui 2017 (3444)	Gram-positive bacilli (*Clostridium difficile*)	Single-site quasi-experimental uncontrolled before-after study, 1 year	2,529 samples from 16 high-touch surfaces (floor, bed rail, bed control, nurse call button, bedside table, chair arm, bin lid, door handle, ceiling vent, bathroom floor, toilet assist bar, toilet flush, toilet seat, tap handle, door handle)	Routine and terminal cleaning consisted of peracetic acid-based disinfectant (40% acetic acid, 35.5% peracetic acid, 6.5% hydrogen peroxide, DiffX) using microfiber cloths on surfaces and microfiber mops on floors. Concentration was 1,000 ppm for surfaces and 750 ppm for floors. Hydrogen peroxide vapor (HPV) decontamination followed terminal cleaning when patient had known infection due to *C. difficile* or	Number (percent) of surfaces positive for *C. difficile* before terminal cleaning was 131 of 572 surfaces (22.9%) compared to after terminal cleaning with 105 of 959 surfaces (10.6%) and after hydrogen peroxide decontamination with 43 of 967 surfaces (4.4%). In single-isolation rooms with known *C. difficile* colonized patient mean count (standard deviation) was 86.9 (98.8) CFU before terminal cleaning, 21.2

Reference (Study ID)	Outcome	Study Design	Setting	Intervention	Results
			from 146 single-isolation rooms and 44 bed-bay areas at a large teaching hospital. London, UK	other HAI (Deprox system, hydrogen peroxide 29-46 ppm). Samples were collected immediately before and immediately after terminal cleaning, and immediately after hydrogen peroxide decontamination.	(38.7) CFU after terminal cleaning, and 7.1 (17.9) CFU after terminal cleaning + HPV decontamination (significance not specified).
Best 2014 (7122)	Gram-positive bacilli (*Clostridium difficile*)	Single site, Quasi-experimental, before and after, uncontrolled study over 10 months	342 samples from high, medium, and low-touch sites (beds, glove dispensers, bins, tables, chairs, handwash basins, curtain tracks, wall trunking, bases of beds, floor, bases of tables, other equipment) from day rooms and bathroom facilities in male and female section of 30-bed stroke rehabilitation unit at large ~2000 bed Teaching Hospital NHS Trust in Leeds, UK	A 7-day deep cleaning with 1000 ppm chlorine-based sporicidal disinfectant (Chlor-Clean, sodium dichloroisocyanurate) with unspecified contact time was conducted on ward. Immediately following, hydrogen peroxide vapor decontamination with 87 ppm atomized HPV (Deprox) ~2 h cycle time. Samples taken before deep cleaning, immediately after deep cleaning, the day after HPV, 19 days after HPV, and 20 weeks post-HPV.	Number of sites (% sites) positive for *C. difficile* decreased from 37/342 (10.8%) to 21/342 (6.1%) after deep cleaning with the chlorine-based sporicidal disinfectant to 0.9% (3) sites positive after HPV. After 19 days, no surfaces were positive. After 20 weeks, 3.5% (12) sites were positive. 92% overall reduction in sites positive for *C. difficile* using chlorine-based disinfectant and HPV. Deep cleaning reduced number of sites positive by 43% compared to HPV further reduced sites positive by 86%. (significance not specified).
Otter 2016 (4992)	Gram-positive bacilli (*Geobacillus stearothermophilus*)	Single site, uncontrolled before-after study over 3 days	5 biological indicators placed outside air transport isolator, 19 placed internally, and 4 for room housing isolators. London, UK.	Biological indicators with known concentration (6-log spores dried onto metal disks) were measured after HPV (Bioquell) decontamination of irregular isolation space (air transport isolator)	All biological indicators were inactivated indicating 6-log reduction.
Havill 2012 (5792)	Gram-positive bacilli (*Geobacillus stearothermophilus*)	Single site, controlled before-	75 spiked carrier disks with known concentration were	Rooms were cleaned with QAC (Virex 256) or 10% bleach wipe (Dispatch) and bathroom doors	There was a 4-log reduction in all of the BIs from all 5 sites. HPV had significantly more sites achieving 4-

Reference (Study ID)	Outcome	Study Design	Setting	Intervention	Results
		after study over two months	placed on 5 surfaces (overbed table, chair, floor under bed, toilet seat, shower floor) in 15 patient rooms on 8 wards in 500-bed teaching hospital. Connecticut, USA	opened prior to decontamination. Then, HPV (Bioquell) decontamination conducted converting 30% hydrogen peroxide liquid into HPV over average of 153 minutes. Known concentration of 10^4-10^6 spores on carrier disks were placed in five sites in each of the rooms before decontamination and evaluated after decontamination (unspecified time). HPV decontamination was compared to UVC (Tru-D, 22,000 microW sec/cm2) decontamination concurrently.	log reduction compared to UVC (p<0.001)
Bates 2005 (12952)	Gram-positive bacilli (Spore-bearers)	Single-site, Quasi-experimental, before and after uncontrolled study over 2 months	66 samples were taken from incubator surfaces in two rooms of the neonatal ICU at a General and Teaching Hospital, Sheffield, UK	An environmental survey was conducted pre-HPV. In addition to standard cleaning with detergent sanitizer, implemented HPV 30% vaporized liquid hydrogen peroxide (Bioquell) overnight (cycle time not stated). Samples taken before and after HPV cycle overnight (time not specified).	0 (0%) sites contaminated with spore-bearing bacteria after HPV intervention compared to 3 (7%) after detergent sanitizer, no significance reported
Otter 2007 (13449)	Gram-positive cocci (*Enterococcus* spp.- Vancomycin-resistant enterococci (VRE))	Single-site, quasi-experimental, uncontrolled before-after study for 19 days	90 samples were taken from 15 sites (floor, beside the bed, floor corner, bed-frame, bed-elevation control panel, bedside chair, bedside locker, over-bed table, remote control, door handle, etc.) in one room in a 500-bed teaching hospital. London, UK.	Terminal cleaning included a QAC disinfectant-detergent (HP800, PVA Hygiene Ltd, Weston-super-Mare, Somerset, UK). HPV was implemented as an adjunct decontamination in one room (Bioquell, 30 min at 20 g/min for two cycles, peak HPV 530-540 ppm). Sampling was taken before and after terminal cleaning and after HPV decontamination.	Number (percent) surfaces positive before vs after terminal cleaning vs. after HPV: 1 (6.7%) vs. 1 (6.7%) vs. 0. Significance not assessed.

Reference (Study ID)	Outcome	Study Design	Setting	Intervention	Results
Ali 2016 (521)	Gram-positive cocci (*Staphylococcus aureus*-MRSA)	Single-site, Quasi-experimental controlled before-after study, time period unspecified	220 samples collected from high frequency touch surfaces (bed frame, footboard, patient chair arm)in 10 isolation rooms at University of London Hospital, London, UK	Compared 2 whole-room hydrogen peroxide decontamination systems after terminal clean with 1000 ppm peracetic acid: (1) 30% hydrogen peroxide heated to 130º C (Bioquell Q10). (2) 4.9% Hydrogen peroxide solution (2 h cycle time, Deprox, Hygiene Solutions) Contact time was 2 – 2.5 h. All surfaces were sampled before exposure to hydrogen peroxide and immediately after HPV decontamination. Coupons inoculated with 10^6 CFU bacterial suspension and placed on surfaces prior to decontamination. Coupons removed after decontamination and compared to non-exposed (control) coupons.	~6.3 log10-reduction with HPV compare to non-exposed. No difference in efficacy by decontamination system ($p>0.05$). MRSA persisted on 25.6% (40/150) and 25.3% (37/146) surfaces for HPV methods 1 and 2 respectively.
Otter 2007 (13449)	Gram-positive cocci (*Staphylococcus aureus*-MRSA)	Single-site, quasi-experimental, uncontrolled before-after study for 19 days	90 samples were taken from 15 sites (floor, beside the bed, floor corner, bed-frame, bed-elevation control panel, bedside chair, bedside locker, over-bed table, remote control, door handle, etc.) in one room in a 500-bed teaching hospital. London, UK.	Terminal cleaning included a QAC disinfectant-detergent (HP800, PVA Hygiene Ltd, Weston-super-Mare, Somerset, UK). HPV was implemented as an adjunct decontamination in one room (Bioquell, 30 min at 20 g/min for two cycles, peak HPV 530-540 ppm). Sampling was taken before and after terminal cleaning and after HPV decontamination.	Number (percent) surfaces positive before vs. after terminal cleaning vs. after HPV: 18 (60%) vs. 12 (40.0%) vs. 1 (3.3%). Surfaces had low MRSA after 1, 2 days after HPV but increased at 5 days post-HPV decontamination. Significance not assessed.
Mitchell 2014 (13718)	Gram-positive cocci (*Staphylococcus aureus*-MRSA)	Single-site controlled cohort study over 7 years	32,661 samples from 9 environmental surfaces (ceiling vent, sink, console, bed, patient/visitor chair, patient table,	MRSA patient rooms were cleaned after discharge with pH-neutral detergent from Jan 1 2006 to Oct 30 2009. From Nov 1 2009 to Dec 31 2012, terminal cleaning was switched to hydrogen peroxide. In single	MRSA was isolated from 24.7% (473/1917) rooms following detergent cleaning and from 18.8% (322/1712) of rooms after HP (349 cleaned manually and 1363 cleaned with

Reference (Study ID)	Outcome	Study Design	Setting	Intervention	Results
			bedside locker, mattress, pillow) from rooms occupied by MRSA patients at 300-bed public acute care hospital. Tasmania, Australia	rooms, HP (6%) vapor decontamination using the dry hydrogen vapor room decontamination system (Nocospray). In shared rooms, HP was applied to surfaces using a cloth (Oxivir TB 0.5%). 9 environmental samples were taken after terminal cleaning.	HPV). HP was more effective than detergent (p<0.001).
Hardy 2007 (14089)	Gram-positive cocci (*Staphylococcus aureus*-MRSA)	Single site, uncontrolled before-after study, over five months	Unspecified number of samples were collected (2 from each bedspace) from environmental surfaces (underside of bed frame, workstation alongside bed) in 9-bedded open-plan ICU at a hospital. Birmingham, UK.	Baseline environmental sampling was conducted for three months prior to deep clean intervention. All patients were removed from ICU prior to deep clean. Terminal clean consisted of washing walls, surfaces, and equipment with detergent and water. HPV (Bioquell) was run overnight at up to 280 ppm. Samples were collected immediately before patient removal, immediately after terminal cleaning, 1 h before HPV, immediately after HPV but before patient readmission, and 24 h and 48 h after patient re-admission. Additional samples were collected weekly for 8 weeks.	Three-month baseline prevalence of MRSA was between 0 and 7 sites positive. After terminal cleaning, 5 sites remained contaminated. After HPV/before re-admission, no MRSA were isolated. After re-opening, 24 h after MRSA was isolated from 5 sites.
Manian 2011 (14130)	Gram-positive cocci (*Staphylococcus aureus*-MRSA)	Single site, uncontrolled before-after study, over 5 years	7140 samples (bedside table, chair, TV, door, sink, bedrail, telephone, lift, cabinet, countertop, etc.) in 384 rooms from all wards at suburban 900-bed community teaching medical center. Missouri, USA.	Routine terminal cleaning and disinfection by rooms vacated by antibiotic-resistant ABC-positive patients consisted of disinfection with QAC followed by 0.525% sodium hypochlorite solution. During room occupation, at least daily disinfection was conducted with sodium hypochlorite. HPV (Bioquell) treatment was conducted following newly-vacated room	After 4 rounds of bleach disinfection, 27% rooms (83/312) and 16% (51/5705) sites were positive for ABC or MRSA. After 1 round of bleach disinfection, there was not significant reduction in number of sites positive (n=700) for MRSA (p=0.45). After 1 round of bleach disinfection and addition of HPV, there was a significant reduction in MRSA-positive sites (odds ratio=0, 95% CI: 0-0.85, p=0.04).

Reference (Study ID)	Outcome	Study Design	Setting	Intervention	Results
				following terminal disinfection with bleach.	
French 2004 (14269)	Gram-positive cocci (*Staphylococcus aureus*-MRSA)	Single site, controlled before-after study	607 samples from surfaces likely to be touched (floor corners, floor areas beside bed, over-bed tables, bed frames, bedside chairs and lockers, door handles, light switch, sink tap, television, remote control) from 18 ward side rooms, 2 four-bedded ward bays, 4 bathrooms recently used by MRSA patients in four ward types (vascular, lower GI, orthopedic, general surgical) at 1200-bed teaching hospital. London, UK.	Terminal cleaning was conducted with detergent sanitizer. HPV (Bioquell, 30% liquid hydrogen peroxide, ~5 h cycle time) was conducted in four single side rooms, shower room, and bathroom after patients with MRSA had left but before terminal cleaning.	90% (111/124) samples comprising ten rooms were positive for MRSA before terminal cleaning compared to 66% (82/124) after cleaning with detergent. 72% (61/85) sites comprising 6 rooms were positive before HPV compared to 1% (1/85) after HPV. Significance not specified.
Barbut 2013 (12491)	Gram-positive cocci (*Staphylococcus aureus*)	Single-site, Quasi-experimental, before and after uncontrolled study, 3 years	165 samples from 28 high-touch surfaces (electric rail, bedside table, dining table, sink, plinth, bathtub, bench, etc.) in burns unit (10 single-bed rooms, operating theater, treatment room) at hospital. Paris, France	HPV disinfection of entire unit and also patient rooms (concentration not specified, ~1.5 h cycle time). Standard cleaning included detergent disinfectant. Surface samples were taken after standard cleaning but before HPV and after HPV (time not specified)	S. *aureus* percentage surfaces positive in patient rooms decreased from 2% (2/102) to 0 and decreased from 1.1% (1/92) at 1 CFU/100 cm^2 to 0 in burns units (no detected, not compared to infection control bundle). No significance reported.
Taneja 2011 (7829)	Gram-positive cocci (*Staphylococcus*	Single site, uncontrolled	252 samples from 126 surfaces (floor, trolley, cabinet, door	Air ducts were contaminated with MRSA. Rooms were washed with detergent and water. Air duct	Before fogging, there were 2353 CFU of total staphylococci, 891 CFU S. *aureus*, 379 MRSA colonies. After

Reference (Study ID)	Outcome	Study Design	Setting	Intervention	Results
	spp. and S. *aureus*, MRSA)	before-after study, over ~12 hours	handle, air-conditioning grill, electrical switch, mattress, IV stand, etc.) in clinical area and rooms in emergency complex. India.	openings were sealed. Fogging conducted with 115% w/v hydrogen peroxide with 0.015% silver nitrate (Ecoshield, Fogmaster ULV 2401). Samples were collected before and after fogging.	fogging, there were 9 CFU total Staphylococci and S. *aureus* and MRSA were not recovered.
Bates 2005 (12952)	Gram-positive cocci (*Staphylococcus* spp.)	Single-site, Quasi-experimental, before and after uncontrolled study over 2 months	66 samples were taken from incubator surfaces in two rooms of the neonatal ICU at a General and Teaching Hospital, Sheffield, UK	An environmental survey was conducted pre-HPV. In addition to standard cleaning with detergent sanitizer, implemented HPV 30% vaporized liquid hydrogen peroxide (Bioquell) overnight (cycle time not stated). Samples taken before and after HPV cycle overnight (time not specified).	0 (0%) sites contaminated with S. *aureus* after HPV intervention compared to 4 (10%) after detergent sanitizer, no significance reported. 1 site (4%) site contaminated with coagulase negative Staphylococci after compared to before 15 (63%), no significance reported.
Passaretti 2013 (2322)	HAI (HAI-*C. difficile*)	Single-site, quasi-experimental controlled study over 30 months	5378 patients at-risk of MDRO acquisition due to prior occupant with MDRO in 6 high-risk units (ICU, surgical unit) at 994-bed tertiary referral hospital. Baltimore, USA	12-month pre-intervention phase followed by 18-month intervention phase with HPV (Bioquell, no specific concentration, 1.5 – 3 h) after standard cleaning on 3 units compared to standard cleaning alone (hydrogen peroxide liquid) on 3 units.	Risk of acquisition was lower in HPV cohort compared to non-HPV units, though not statistically significant with risk ratio (95% confidence interval) 0.49 (0.16 – 1.47). *C. difficile* acquisition was 0.7% in HPV units compared to 2.1% in non-HPV units.
McCord 2016 (4861)	HAI (HAI-*C. difficile*)	Single site, uncontrolled before-after study, over 4 years	Patients with healthcare-associated CDI (i.e. CDI detected after three days of admission) at hospital. Mississippi, USA.	Before HPV intervention, daily and terminal cleaning consisted of 6500 sodium hypochlorite (Dispatch) with 5 min contact time. During the 2-year period with HPV intervention, daily cleaning conducted with same sodium hypochlorite and terminal cleaning with QAC (Vie II 256). HPV consisted of terminal disinfection with 35% hydrogen peroxide (cycle time 1 h 45 min).	The CDI rate decreased 60% from 1.0 (258 cases) to 0.4 (123 cases) cases per 1000 patient-days before (24 months) compared to after (24 months) introduction of HPV. The analysis found significant reductions due to seasonal change as well as due to introduction of HPV, however 95% confidence interval included a portion of the period without HPV intervention

Reference (Study ID)	Outcome	Study Design	Setting	Intervention	Results
Manian 2013 (5113)	HAI (HAI-*C. difficile*)	Single site, uncontrolled before-after study, 3 years	Patients with nosocomial *C. difficile* infection following 72 h after admission with positive test for cytotoxin A or B. suburban 900-bed community teaching medical center. Missouri, USA.	The pre-intervention period spanned two years and included terminal cleaning with 0.525% bleach when formerly occupied by patient colonized with antibiotic-resistant pathogen (VRE, MRSA, A. baumannii, gram-negative bacilli) or *C. difficile*. HPV disinfection (Bioquell, cycle time 3-4 h) was added to terminal cleaning for intervention period (1 year).	There was a significant reduction in *C. difficile* associated diarrhea rates from 322 cases, 0.88/1000 patient-days during the pre-intervention period compared to 109 cases, 0.55/1000 patient-days during the intervention period (rate ratio=0.63, 95% CI: 0.50-0.79, p<0.001)
Boyce 2008 (813)	HAI (HAI – *C. difficile*-associated disease (CDAD))	Single-site, Quasi-experimental, uncontrolled before-after study over 10 months	CDAD considered positive for patient with diarrhea and positive lab result in 5 high-incidence wards and at 500 beds hospital of San Raphael, New Haven, USA	Incidence compared before HPV intervention but with infection control practices (June 2004 – April 2005) to intervention period (June 2005 –March 2006) Three of five high-incidence CDAD had ward decontamination with HPV. All wards had terminal decontamination of patient rooms with HPV. HPV consisted of had 30 % hydrogen peroxide solution (Bioquell, 12 h cycle time for ward, 3-4 h cycle time per room).	CDAD incidence was significantly lower on 5 high-incidence wards from 2.28 cases to 1.18 (p=0.047) cases per 1,000 patient-days after HPV cleaning. Hospital-wide CDAD incidence lower (p=0.26) at 0.84 compared to 1.36 cases per 1000 patient-days after HPV.
Passaretti 2013 (2322)	HAI (HAI-MDR-GNR)	Single-site, quasi-experimental controlled study over 30 months	5378 patients at-risk of MDRO acquisition due to prior occupant with MDRO. No surveillance specifically for MDR-GNR in 6 high-risk units (ICU, surgical unit) at 994-bed tertiary referral hospital. Baltimore, USA	3-month pre-intervention phase followed by 6-month intervention phase with HPV (Bioquell, no specific concentration, 1.5 – 3 h) after standard cleaning on 3 units compared to standard cleaning alone with quaternary ammonium compound (active ingredient not specified, 3M) on 3 units. Samples were taken monthly.	Risk of acquisition was lower in HPV cohort compared to non-HPV units, though not statistically significant with risk ratio (95% confidence interval) 0.55 (0.20 – 1.57). MDR-GNR acquisition was 1.2% in HPV units compared to 1.8% in non-HPV units.

Reference (Study ID)	Outcome	Study Design	Setting	Intervention	Results
Passaretti 2013 (2322)	HAI (HAI-MRSA)	Single-site, quasi-experimental controlled study over 30 months	5378 patients at-risk of MDRO acquisition due to prior occupant with MDRO. Weekly surveillance for MRSA, VRE in 6 high-risk units (ICU, surgical unit) at 994-bed tertiary referral hospital. Baltimore, USA	3-month pre-intervention phase followed by 6-month intervention phase with HPV (Bioquell, no specific concentration, 1.5 – 3 h) after standard cleaning on 3 units compared to standard cleaning alone with quaternary ammonium compound (active ingredient not specified, 3M) on 3 units. Samples were taken monthly.	Risk of acquisition was lower in HPV cohort compared to non-HPV units, though not statistically significant with risk ratio (95% confidence interval) 0.53 (0.16 – 1.79). MRSA acquisition was 0.9% in HPV units compared to 2.8% in non-HPV units.
Mitchell 2014 (13718)	HAI (HAI-MRSA)	Single-site uncontrolled before-after study over 7 years	32,661 samples from 9 environmental surfaces (e.g. ceiling vent, sink, console, bed, patient/visitor chair, patient table, bedside locker, mattress, pillow) from rooms occupied by MRSA patients at 300-bed public acute care hospital. Tasmania, Australia	MRSA patient rooms were cleaned after discharge with pH-neutral detergent from Jan 1 2006 to Oct 30 2009. From Nov 1 2009 to Dec 31 2012, terminal cleaning was switched to hydrogen peroxide. In single rooms, HP (6%) vapor decontamination using the dry hydrogen vapor room decontamination system (Nocospray). In shared rooms, HP was applied to surfaces using a cloth (Oxivir TB 0.5%). MRSA screening was conducted on some patients prior to 2010 and all patients after 2010.	Incidence of MRSA colonization and infection decreased from 9.0/10,000 patient days during detergent period to 5.3/10,000 patient days during the HP disinfectant period ($p<0.001$).
Passaretti 2013 (2322)	HAI (HAI- VRE)	Single-site, quasi-experimental controlled study over 30 months	5378 patients at-risk of MDRO acquisition due to prior occupant with MDRO. Weekly surveillance for MRSA, VRE in 6 high-risk units (ICU, surgical unit) at 994-bed tertiary referral hospital. Baltimore, USA	3-month pre-intervention phase followed by 6-month intervention phase with HPV (Bioquell, no specific concentration, 1.5 – 3 h) after standard cleaning on 3 units compared to standard cleaning alone with quaternary ammonium compound (active ingredient not specified, 3M) on 3 units. Samples were taken monthly.	MDRO reduction was driven by significant reduction in VRE in HPV units ($p<0.01$) compared to non-HPV units (risk ratio=0.25, 95% confidence interval =0.10, 0.60) between HPV and combined treatment. VRE acquisition was 1.7% in HPV units compared to 8.1% in non-HPV units.

Reference (Study ID)	Outcome	Study Design	Setting	Intervention	Results
Holmdahl 2016 (1268)	Virus (Feline calicivirus-human norovirus model)	Single site, controlled before-after study over 3 days	72 samples from surfaces (bed table, top of cupboard, bathroom, floor, behind toilet, behind door) in unoccupied patient room at a university hospital. Malmo, Sweden.	Dried virus stock on plastic plates placed in triplicate at six locations comprising three heights in intervention room and two locations in control room. The intervention with HPV (Bioquell Q10 Suite, peak 474-505 ppm, 3 h cycle time, 40-50 min gassing time) was conducted in non-occupied patient room on three occasions. Virus recovery was compared from plates in intervention room with HPV to virus recovery from plates placed in an untreated patient room.	No viable FCV was recovered from any sample in the treated room (<1.0 log 50% tissue culture infectious dose (TCID50)/100uL), but were recovered from the control room (mean 4.65 log TCID50/100uL) demonstrating at least a 3.65 log by TCID50 reduction.
Holmdahl 2016 (1268)	Virus (Murine norovirus-human norovirus model)	Single site, controlled before-after study over 3 days	72 samples from surfaces (bed table, top of cupboard, bathroom, floor, behind toilet, behind door) in unoccupied patient room at a university hospital. Malmo, Sweden.	Dried virus stock on plastic plates placed at six locations comprising three heights in intervention room and two locations in control room. The intervention with HPV (Bioquell Q10 Suite, peak 474-505 ppm, 3 h cycle time, 40-50 min gassing time) was conducted in non-occupied patient room on three occasions. Virus recovery was compared from plates in intervention room with HPV to virus recovery from plates placed in an untreated patient room.	No viable MNV was recovered from any sample in the treated room (<1.0 log 50% tissue culture infectious dose (TCID50)/100uL), but were recovered from the control room (mean 4.67 log TCID50/100uL) demonstrating at least a 3.67 log by TCID50 reduction. There was at least a 2.85 reduction in plaques with average plaque count in control room 3.35 PFU/100uL compared to below detection in intervention room (<0.5).

Table S20: Study results for other vapor interventions ordered by outcome organism

Reference (Study ID)	Outcome	Study Design	Setting	Intervention	Results
Aimiya 1989 (484)	All viable organisms	Single-site, quasi-experimental, uncontrolled before-after study over 6 months	Samples from the floor were taken from 4 rooms in the prematurity room, newborn room, ICU, and NCU of a new ward built at National Nagoya Hospital in Japan.	Baseline measurements were taken prior to mopping and after primary decontamination and fogging with 0.5% alkyldiaminoethylglycine hydrochloride. Routine cleaning then consisted of floors mopped with benzethonium chloride (0.1-0.2%, Hyamine t) or alkylpolyaminoethylglycine hydrochloride (TEGO-51, 0.1-0.2%). Samples were collected before and 1-2 h after primary decontamination/fogging. Samples were also collected three times (one month apart) six months after primary decontamination.	After primary decontamination with Tego-51 (0.5%), no samples were positive with significant reductions compared to before fogging (initial concentration median ~10-~40 CFU/10 cm2). Median count before and after decontamination with 0.5% alkyldiaminoethylglycine hydrochloride showed that there was a significant decrease (p<0.01) in all rooms. After six months, bacterial concentration remained low in operating room and prematurity room, had median ~50 CFU/10 cm2 for newborn room and NICU, and had ~100 CFU/10 cm2 for a normal room
Gelmini 2016 (1105)	All viable organisms	Single-Site Quasi-experimental, controlled cohort study over 5 months.	Samples from high-touch surfaces (tables, cabinet surfaces, handrails) from patient rooms of 114-bed residential health care house. Iseo, Italy.	Control rooms received standard cleaning. Intervention rooms had standard cleaning and essential oil nebulizers with water and 0.02% essential oil mixture (Lavandula angus-tifolia 24%, Melaleuca cajuputi 24%(Cajeput), Abiessiberica 20%, Myrtus communis 20%, Pelargoniumgraveolens (Geranium bourbon) 12%) dispersed with ultrasound vaporizers that were left working for 8 h. Samples taken before intervention and then every 30 days for 5 months	Significant reduction (p<0.05) in total organisms on tables (>90%) and cabinets (>75%) compared to control sites throughout the study. Handrails, located further/outside room of nebulizers did not have significant reduction (p>0.05) compared to control.
Oztoprak 2019 (2288)	All viable organisms	Single-site quasi experimental, controlled before-after study over one month	5 high-touch surfaces (buttons, bedside table, bed rail, floor) from 3 rooms in 43-bed ICU at tertiary care hospital. Turkey	Each of the following disinfectants was used in one of three rooms: steam technology (Tecnovap Evo 304) compared to two-step cleaning with detergent and water on microfiber cloths followed by 1,000 ppm, or 5,000 ppm hypochlorite solution (sodium dichloroisocyanurate) wipes. Known	The following differences in average (standard deviation) ATP bioluminescence (RLU) was significantly lower (98%) after steam disinfection from 578 (76) to 9.5 (2.3) (p< 0.001). Steam cleaning had significantly lower ATP compared to hypochlorite solutions (p<0.05). Chlorine interventions also had

Reference (Study ID)	Outcome	Study Design	Setting	Intervention	Results
				organism concentration inoculated onto pre-cleaned surface. Samples taken 10 minutes after inoculation and after disinfection.	significant reductions ($p<0.001$) in ATP from 651 (66) to 22 (5.2) with 1000 ppm and from 632 (64) to 14 (2.9) with 5000 ppm chlorine.
Sexton 2011 (2707)	All viable organisms	Single-site quasi experimental, uncontrolled study over 2 days	32 samples from high-touch surfaces (bedrails, tabletops, chair arms, sinks, doors) in 8 rooms from long-term care unit at hospital. Arizona, USA.	Portable saturated steam vapor device with tap water (VaporJet PC 2400) applied to surfaces with light pressure (~10-20 s per surface, 12-15 psi). Samples were taken before and after disinfection.	Bedrails had highest initial concentration. Average (standard deviation) concentration before compared to after disinfection on bedrails was 1,590 (3,190) CFU/in^2 before vs 103 (352) CFU/in^2 after disinfection which corresponded to a log10 reduction 1.19, >90% reduction. Maximum log10 reduction was 1.76 on bedside tables.
Shekhawat 1992 (2745)	All viable organisms	Single-Site, quasi-experimental, uncontrolled study	Floors of a neonatal unit at medical college. Jodhpur, India.	Study carried out in two parts. Routine cleaning (4% phenol solution) compared to formalin fumigation (34-38% formalin) Fumigation took 24h. Samples were taken after routine cleaning and just after fumigation. `	Concentration reduced from 320-388 CFU/cm^2 to 118-136 CFU/cm^2 after fumigation corresponding to reduction between 57-66%.
Singh 2017 (4519)	All viable organisms	Single-site quasi experimental, controlled before-after study	27 surface samples (wall, bed) from three high risk areas (ICU, HDU, and isolation rooms) at hospital. Amritsar, India	Fogging was conducted with three different disinfectants: (1) 20% solution for fogging (hydrogen peroxide-10%, silver solution-0.01%); (2) 0.39% QAC solution (Octyldecyl-dimethyl-ammonium-chloride 6.5%, dioctyldimethyl-ammonium-chloride 2.6%, dodecyl-dimethylammonium-chloride 3.9%, alkylkimethyl-benzylammonium-chloride 8.7%); (3) 1% QAC solution (N-alkyldimethyl-benzylammonium chloride-13.6%, didecyldimethyl-ammonium-chloride 13%, polymeric biguanide hydro-chloride 5%). Also compared efficacy with and without pre-cleaning with detergent.	All samples were positive for bacteria prior to disinfection compared to 44% with disinfectant 1, 33% with disinfectant 2, and 67% with disinfectant 3. Disinfectants 1 and 2 had significantly lower ($p<0.05$) number of samples positive. 100% reduction in bacterial concentration with disinfectants 1 (initial=30 CFU) and 2 (initial=50 CFU), 95% reduction in bacterial concentration with disinfectant 3 (initial=40 CFU) when pre-cleaning was conducted. % reduction is lower without pre-cleaning
Munster 1974 (4733)	All viable organisms	Single-site quasi	1440 surfaces (floor, walls,	Manual cleaning with a phenolic compound (Ves-phene One Stroke,	Average initial concentration on surfaces was 25.6 CFU. Mean percent (standard

Reference (Study ID)	Outcome	Study Design	Setting	Intervention	Results
		experimental, controlled crossover study	fixtures) from one room in surgical ICU at hospital. South Carolina, USA.	phenyl phenol, benze p-chlorophenol and p-tertiary aminophenol) was compared with quaternary ammonium compound fogging disinfectant (Micromist, ethanol, cetyl trimethyl ammonium-bromide, propylene glycol, cetyl pyridinium chloride, and dimethyl benzyl ammonium chloride). In one group, manual cleaning was followed by fogging. In second group, fogging was followed by manual cleaning. Samples taken after manual cleaning and after fogging.	deviation) change following fogging was decrease of 76% (13.2) compared to an increase of 293% (262) after manual cleaning. Significance not specified.
Strat 1971 (5698)	All viable organisms	Single-site, Quasi-experimental, controlled before-after study for a 3-day period.	581 surface samples (operating tables, instruments, walls, floors) from 7 rooms in the Bucharest Institute of Hygiene. Bucharest, Romania.	A hydrochloride solution (1% Ampholytic detergent with dodecyldiaminoethylglycine; Tego 103G), 1% cationic detergent (cetylpyridinium chloride) (BCP), and alcohol-based disinfectant (triethylene glycol; TEG) were used to simultaneously aerosolize/disinfect air and manually wipe/disinfect surfaces. Samples taken at least 10-15 minutes after cleaning as well as 1.5, 6, and 12 h after cleaning. These values were compared to standard cleaning (water with soda).	On average, the efficiency of each disinfectant for decreasing the bacterial load is 90% for BCP (80-98.5%), 95% for Tego 103G (88-99%), 96% for TEG aerosolization and BCP wipes (80-98.5%) and 99% for TEG aerosolization and Tego 103G wipes (98.8-100%) compared to standard cleaning. The efficiency of these disinfectants changed throughout the day as well. After 1.5 h, BCP is 13.8 times more effective, Tego 103G is 22 times more effective at reducing bacterial count. After 6 h, efficiency lowers with BCP is 3.6 times more effective and Tego 103G is 5.6 times more effective at reducing bacterial load. After 12.5 h, BCP was 5.3 times more effective and Tego 103G is 9.8 times more effective at reducing bacterial load. Bacterial load included *Staphylococcus*, P. *aeruginosa*, *Proteus* mirabilis. *E. coli* were not isolated.
Nakata 2001 (8147)	All viable organisms	Single-Site quasi-experimental,	8 easily touched surfaces (floor, wall, shelf,	A fogging disinfection unit compared five chemicals: 0.5% alkyldiaminoethylglycine (Ikeuchi),	Concentration percent reduction with fogging disinfectants (from highest to lowest) was 92.8% for benzalkonium

Reference (Study ID)	Outcome	Study Design	Setting	Intervention	Results
		controlled before-after study	ceilings operating table, surgical lights) in surgical center at Osaka University Medical School. Japan	0.2% benzalkonium chloride (Nihon), 0.2% acidic electrolytic water sodium hypochlorite (Nippon Shinyaku) and 0.5% glutaral (Maruishi Pharmaceutical), acidic electrolytic water (Bio Japan). Spray time 16-30 min. Samples taken before and ~ 30 min after fogging cycle.	chloride, 92.5% for sodium hypochlorite, 90.5% for glutaral, 90.2% for alkyldiaminoethylclycine, and 76.8% for acidic electrolytic water. Differences among disinfectants not assessed.
Nagai 1983 (8154)	All viable organisms	Single site, quasi-experimental controlled before-after study	48 samples (floor, wall, ceiling) from four operating rooms at a hospital. Japan.	Operating room was sprayed with either 0.2% chlorhexidine gluconate or 0.1% alkylpolyaminoethyl glycine using a tube sprayer. Samples were collected 1 h, 1 week, and 2 weeks after spraying	Average concentration after chlorhexidine gluconate spray was 0 at 1 h, 0.13-1.21 CFU/10 cm2 at 1 week and 0.15-5.96 CFU/10 cm2 at 2 weeks. Average concentration after alkylpolyaminoethyl glycine chloride spray was 3.66-4.02 CFU/10 cm2 at 1 h, 1.52-4.08 CFU/10 cm2 at 1 week and 0.88-2.02 CFU/10 cm2 at 2 weeks.
Dyas 1983 (9651)	All viable organisms	Single site, quasi-experimental uncontrolled before-after study	Inoculated organism measured after intervention in one hospital room at Queen Elizabeth Hospital, UK.	Ozone generator (0.001 ppm, Coronair Airbracer, 6 h cycle time) device. Known concentration of organism inoculated in room prior to ozone and measured after ozone intervention.	Ozone concentration in hospital room was 0.001 ppm and no bactericidal effect was seen. Significance not specified.
Gelmini 2016 (1105)	Fungi	Single-Site Quasi-experimental, controlled cohort study over 5 months.	Samples from high-touch surfaces (tables, cabinet surfaces, handrails) from patient rooms of 114-bed residential health care house. Iseo, Italy.	Control rooms received standard cleaning. Intervention rooms had standard cleaning and essential oil nebulizers with water and 0.02% essential oil mixture (Lavandula angus-tifolia 24%, Melaleuca cajuputi 24%(Cajeput), Abiessiberica 20%, Myrtus communis 20%, Pelargoniumgraveolens (Geranium bourbon) 12%) dispered with ultrasound vaporizers that were left working for 8 h. Samples taken before intervention and then every 30 days for 5 months	Significant reduction ($p<0.05$) in fungi on tables (>90%) and cabinets (>75%) compared to control sites throughout the study. Handrails, located further/outside room of nebulizers did not have significant reduction ($p>0.05$) compared to control.

Reference (Study ID)	Outcome	Study Design	Setting	Intervention	Results
Singh 2017 (4519)	Fungi	Single-site quasi experimental, controlled before-after study	27 surface samples (wall, bed) from three high risk areas (ICU, HDU, and isolation rooms) at hospital. Amritsar, India	Fogging was conducted with three different disinfectants: (1) 20% solution for fogging (hydrogen peroxide-10%, silver solution-0.01%); (2) 0.39% QAC solution (Octyldecyl-dimethyl-ammonium-chloride 6.5%, dioctyldimethyl-ammonium-chloride 2.6%, didecyldimethyl-ammonium-chloride 3.9%, alkyl-kimethylbenzylammonium-chloride 8.7%); (3) 1% QAC solution (N-alkyldimethyl-benzylammonium chloride-13.6%, didecyldimethyl-ammonium-chloride 13%, polymeric biguanide hydro-chloride 5%). Also compared efficacy with and without pre-cleaning with detergent.	100% reduction in fungal concentration with disinfectants 1 (from 2 CFU to 0 CFU) and disinfectant 2 (from 3 CFU to 0 CFU). 50% reduction in fungal concentration with disinfectant 3 (from 4 CFU to 2 CFU) when pre-cleaning was conducted. % reduction is lower without pre-cleaning.
Alekseeva 1969 (6936)	Fungi	Single-site, quasi-experimental, uncontrolled before-after study	216 samples taken from surfaces in dining room, rest room operating room, ward, corridor, etc. in an anti-tuberculous institution in Russia.	Indicator organism (non-pathogenic acid resistant saprophyte b-5) was seeded onto surfaces at 27×10^4 – 99×10^7 microbial bodies/cm2. Then, 40% aqueous solution of formaldehyde sprayed at rate of 20 mL/m^3 with 24 h exposure and followed by neutralization with 25% ammonia solution at rate of 10 mL/m^3. Samples were taken before and after disinfection.	Total contamination (standard deviation) averaged 182×10^6 (12×10^6) to 233×10^7 (102×10^7) microbial bodies/cm^2 before disinfection from 72 samples. After disinfection, only one surface was positive of 144 samples (concentration not specified). Significance not specified.
Dyas 1983 (9651)	Fungi	Single site, quasi-experimental uncontrolled before-after study	Inoculated organism measured after intervention in one hospital room at Queen Elizabeth Hospital, UK.	Ozone generator (0.001 ppm, Coronair Airbracer, 6 h cycle time) device. Known concentration of organism inoculated in room prior to ozone and measured after ozone intervention.	Ozone concentration in hospital room was <0.001 ppm and no fungicidal effect was seen. Significance not specified.
Oztoprak 2019 (2288)	Gram-negative bacteria (*Acinetobacter*	Single-site quasi experimental, controlled	5 high-touch surfaces (buttons, bedside table, bed rail, floor) from 3	Each of the following disinfectants was used in one of three rooms: steam technology (Tecnovap Evo 304) compared to two-step cleaning with	No bacterial growth after steam or hypochlorite disinfection. Unclear if inoculated surfaces were culture-positive.

Reference (Study ID)	Outcome	Study Design	Setting	Intervention	Results
	baumannii-MDR)	before-after study over one month	rooms in 43-bed ICU at tertiary care hospital. Turkey	detergent and water on microfiber cloths followed by 1,000 ppm, or 5,000 ppm hypochlorite solution (sodium dichloroisocyanurate) wipes. Known organism concentration inoculated onto pre-cleaned surface. Samples taken 10 minutes after inoculation and after disinfection.	
Lowe 2013 (2427)	Gram-negative bacteria (*Acinetobacter baumannii*)	Single-site quasi-experimental, uncontrolled repeated before-after study	60 samples (bed rails, mattresses, countertops, mounted light fixture, floor, sink) were taken from 6 rooms in biocontainment patient care unit at medical center. Nebraska, USA	The intervention was six repetitions (before-after) of ClO2 (351-385 ppm maintained < 3 h, with exposures of 667-890 ppm-h, Minidox-M Decontamination System) on known concentration ($9x10^9$ CFU) of organism inoculated onto 10 surfaces with samples taken after reduction of ClO2 gas to 0 ppm	The mean log10 reduction (%) for MDR A. baumannii (HDR BC 9782) was 8.55 (99.3%). Significance not specified since complete inactivation for most samples.
Čamdžić 2019 (6269)	Gram-negative bacteria (*Acinetobacter baumannii*)	Single site, quasi-experimental uncontrolled before-after study	7 samples were taken from surfaces (bed frame, console, respirator monitor, table, floor) in isolation room in tertiary care hospital. Sarajevo, Bosnia and Herzegovina	Intervention was ozone-producing disinfection device (Sterisafe Pro, cycle time 105-180 min). Samples were taken immediately after patient discharge, after standard cleaning, and after the disinfection cycle.	Number of samples positive was 0 after intervention compared to 3 samples positive prior to ozone disinfection. After standard cleaning, concentration ranged from 0 – 100 CFU/cm^2). After ozone, 0 CFU/cm^2 Significance not specified.
Lowe 2013 (2427)	Gram-negative bacteria (*Acinetobacter mycobacterium smegmatis*)	Single-site quasi-experimental, uncontrolled repeated before-after study	60 samples (bed rails, mattresses, countertops, mounted light fixture, floor, sink) were taken from 6 rooms in biocontainment	The intervention was six repetitions (before-after) of ClO2 (351-385 ppm maintained < 3 h, with exposures of 667-890 ppm-h, Minidox-M Decontamination System) on known concentration ($9x10^9$ CFU)of organism inoculated onto 10 surfaces with samples	The mean log10 reduction (%) for M. smegmatis (ATCC 14468) was 9.32 (99.3%). Significance not specified since complete inactivation for most samples.

Reference (Study ID)	Outcome	Study Design	Setting	Intervention	Results
			patient care unit at medical center. Nebraska, USA	taken after reduction of ClO2 gas to 0 ppm	
Lowe 2013 (2427)	Gram-negative bacteria (*Escherichia coli*)	Single-site quasi-experimental, uncontrolled repeated before-after study	60 samples (bed rails, mattresses, countertops, mounted light fixture, floor, sink) were taken from 6 rooms in biocontainment patient care unit at medical center. Nebraska, USA	The intervention was six repetitions (before-after) of ClO2 (351-385 ppm maintained < 3 h, with exposures of 667-890 ppm-h, Minidox-M Decontamination System) on known concentration of organism inoculated onto 10 surfaces with samples taken after reduction of ClO2 gas to 0 ppm	The mean log10 reduction (%) for *E. coli* (two strains) was 9.02 (99.2%). Significance not specified since complete inactivation for most samples..
Singh 2017 (4519)	Gram-negative bacteria (*Escherichia coli*)	Single-site quasi experimental, controlled before-after study	27 surface samples (wall, bed) from three high risk areas (ICU, HDU, and isolation rooms) at hospital. Amritsar, India	Fogging was conducted with three different disinfectants: (1) 20% solution for fogging (hydrogen peroxide-10%, silver solution-0.01%); (2) 0.39% QAC solution (Octyldecyl-dimethyl-ammonium-chloride 6.5%, dioctyldimethyl-ammonium-chloride 2.6%, didecyldimethyl-ammonium-chloride 3.9%, alkyl-kimethylbenzylammonium-chloride 8.7%); (3) 1% QAC solution (N-alkyldimethyl-benzylammonium chloride-13.6%, didecyldimethyl-ammonium-chloride 13%, polymeric biguanide hydro-chloride 5%).	Surfaces positive for *Pseudomonas* before compared to after disinfection was from 11% to 0% with disinfectant 1, from 0% to 0% with disinfectant 2, and from 33% to 0% with disinfectant 3.
Lowe 2013 (5183)	Gram-negative bacteria (*Francisella tularensis*)	Single-site quasi-experimental, controlled before-after study	80 surfaces (windows, metal cabinets, walls, bathroom sinks, floor, bed mattresses, light fixtures) in one patient care suite biocontainment	4 repetitions/trials compared chlorine dioxide decontamination (Mindox-M Decontamination System, gas concentration 377 to 385 ppm maintained to exposures of 767 ppm-h) on inactivation of control organism with non-exposed control organism placed in adjacent room without ClO2. Control organism with known concentration	Range of percent inactivation of spores was 100% with range of average log10 reduction of 8.8 – 9.6.

Reference (Study ID)	Outcome	Study Design	Setting	Intervention	Results
			patient care unit at 635-bed medical center. Nebraska, USA.	(10^{10} CFU) (before) compared to samples collected after ClO2	
Singh 2017 (4519)	Gram-negative bacteria (*Klebsiella* spp.)	Single-site quasi experimental, controlled before-after study	27 surface samples (wall, bed) from three high risk areas (ICU, HDU, and isolation rooms) at hospital. Amritsar, India	Fogging was conducted with three different disinfectants: (1) 20% solution for fogging (hydrogen peroxide-10%, silver solution-0.01%); (2) 0.39% QAC solution (Octyldecyl-dimethyl-ammonium-chloride 6.5%, dioctyldimethyl-ammonium-chloride 2.6%, didecyldimethylammonium-chloride 3.9%, alkyl-kimethylbenzylammonium-chloride 8.7%); (3) 1% QAC solution (N-alkyldimethyl-benzylammonium chloride-13.6%, didecyldimethyl-ammonium-chloride 13%, polymeric biguanide hydro-chloride 5%).	Surfaces positive for *Klebsiella* had 67% reduction from 56% to 33% surfaces positive with disinfectant 1, 46% reduction from 89% to 22% surfaces positive with disinfectant 2, and 0% reduction from 67% to 67% surfaces positive with disinfectant 3.
Oztoprak 2019 (2288)	Gram-negative bacteria (*Pseudomonas aeruginosa*-Carbapenem-resistant *Pseudomonas aeruginosa*)	Single-site quasi experimental, controlled before-after study over one month	5 high-touch surfaces (buttons, bedside table, bed rail, floor) from 3 rooms in 43-bed ICU at tertiary care hospital. Turkey	Each of the following disinfectants was used in one of three rooms: steam technology (Tecnovap Evo 304) compared to two-step cleaning with detergent and water on microfiber cloths followed by 1,000 ppm, or 5,000 ppm hypochlorite solution (sodium dichloroisocyanurate) wipes. Known organism concentration inoculated onto pre-cleaned surface. Samples taken 10 minutes after inoculation and after disinfection.	No bacterial growth after steam or hypochlorite disinfection. Initial concentration not reported.
Singh 2017 (4519)	Gram-negative bacteria (*Pseudomonas* spp.)	Single-site quasi experimental, controlled before-after study	27 surface samples (wall, bed) from three high risk areas (ICU, HDU, and isolation rooms) at hospital. Amritsar, India	Fogging was conducted with three different disinfectants: (1) 20% solution for fogging (hydrogen peroxide-10%, silver solution-0.01%); (2) 0.39% QAC solution (Octyldecyl-dimethyl-ammonium-chloride 6.5%, dioctyldimethyl-ammonium-chloride	Surfaces positive for *E. coli* had 17% reduction from 33% to 0% surfaces positive with disinfectant 1, 4% reduction from 11% to 0% surfaces positive with disinfectant 2, and 0% reduction from 0% to 0% surfaces positive with disinfectant 3.

Reference (Study ID)	Outcome	Study Design	Setting	Intervention	Results
				2.6%, didecyldimethyl-ammonium-chloride 3.9%, alkyl-kimethylbenzylammonium-chloride 8.7%); (3) 1% QAC solution (N-alkyldimethyl-benzylammonium chloride-13.6%, didecyldimethyl-ammonium-chloride 13%, polymeric biguanide hydro-chloride 5%).	
Sexton 2011 (2707)	Gram-negative bacteria (Total coliform bacteria)	Single-site quasi experimental, uncontrolled study over 2 days	96 samples from high-touch surfaces (bedrails, tabletops, chair arms, sinks, doors) in 8 rooms from long-term care unit at hospital. Arizona, USA.	Portable saturated steam vapor device with tap water (VaporJet PC 2400) applied to surfaces with light pressure (~10-20 s per surface, 12-15 psi). Samples were taken before and after disinfection.	Percent surfaces positive was 81% (29/48) before compared to 13% (6/48) after. Significance not specified. Bedrails had highest initial concentration with average (standard deviation) concentration 106 (183) CFU/in^2 before and was not detected (<4.0 CFU/in^2) after disinfection. This corresponded to log10 reduction of 1.42.
Lowe 2013 (5183)	Gram-negative bacteria (*Yersinia pestis*)	Single-site quasi-experimental, controlled before-after study	80 surfaces (windows, metal cabinets, walls, bathroom sinks, floor, bed mattresses, light fixtures) in one patient care suite biocontainment patient care unit at 635-bed medical center. Nebraska, USA.	4 repetitions/trials compared chlorine dioxide decontamination (Mindox-M Decontamination System, gas concentration 377 to 385 ppm maintained to exposures of 767 ppm-h) on inactivation of control organism with non-exposed control organism placed in adjacent room without ClO2. Control organism with known concentration (10^{10} CFU) (before) compared to samples collected after ClO2	Range of percent inactivation of spores was 100% with range of average log10 reduction of 6.9 – 8.8.
Lowe 2013 (5183)	Gram-positive bacilli (*Bacillus anthracis*)	Single-site quasi-experimental, controlled before-after study	80 surfaces (windows, metal cabinets, walls, bathroom sinks, floor, bed mattresses, light fixtures) in one patient care suite	4 repetitions/trials compared chlorine dioxide decontamination (Mindox-M Decontamination System, gas concentration 377 to 385 ppm maintained to exposures of 767 ppm-h) on inactivation of control organism with non-exposed control organism placed in adjacent room without ClO2. Control	Range of percent inactivation of spores was 93-100% with range of average log10 reduction of 7.8-10.0. Range of percent inactivation of vegetative was 99-100% with range of average log10 reduction of 7.9-8.5.

Reference (Study ID)	Outcome	Study Design	Setting	Intervention	Results
			biocontainment patient care unit at 635-bed medical center. Nebraska, USA.	organism with known concentration (10^{10} CFU) (before) compared to samples collected after ClO2	
Lowe 2013 (5183)	Gram-positive bacilli (*Bacillus atrophaeus*)	Single-site quasi-experimental, controlled before-after study	80 surfaces (windows, metal cabinets, walls, bathroom sinks, floor, bed mattresses, light fixtures) in one patient care suite biocontainment patient care unit at 635-bed medical center. Nebraska, USA.	4 repetitions/trials compared chlorine dioxide decontamination (Mindox-M Decontamination System, gas concentration 377 to 385 ppm maintained to exposures of 767 ppm-h) on inactivation of control organism with non-exposed control organism placed in adjacent room without ClO2. Control organism with known concentration (10^6 spores) on surfaces (before) compared to samples collected after ClO2	Biological indicator was inactivated on 58/60 sites after intervention. Sites with growth were due to closed door in hospital room. Significance not specified since complete inactivation for most samples.
Barbeito 1966 (9616)	Gram-positive bacilli (*Bacillus subtilis*)	Single-Site, Quasi-experimental, uncontrolled before-after study	24 samples from surfaces (wall, windowsill, floor sink, door handle, operating table, stands, anesthetizing machine, beds, chair, stool, etc.) from isolation ward and operation suite in community hospital. Maryland, USA	Biological indicator with known concentration (1 x 10^4 spores/mL) B. subtilis were seeded onto surfaces placed in rooms. To assess efficacy, beta-propiolactone (BPL) vapor (Tergisyl (R) from Lehn & Fink Products Corp with orthohydroxydiphenyl, paratertiary amylphenol, sodium sulfonates at 900 mL BPL/12,000-16,000 ft^3, 2 h wet contact time, 4.5 h total cycle time) implemented. Samples taken prior to BPL and after vapor was diffused for 30 minutes.	B. subtilis were not recovered from any surface after BPL disinfection. Significance not specified.
Nagai 1983 (8154)	Gram-positive bacilli (*Bacillus subtilis*)	Single site, quasi-experimental controlled	90 samples (floor, wall, ceiling) from an operating room at a hospital. Japan.	Operating room was sprayed with 0.2% chlorhexidine gluconate using a tube sprayer. Petri dishes were inoculated with biological indicator B. subtillis, placed in operating room floors, walls,	All samples from floors were not positive for any duration of spray (5 through 60 min). Samples placed on walls and ceilings showed growth after 5 min, 10 min, and 20 min. After 60 min spray,

Reference (Study ID)	Outcome	Study Design	Setting	Intervention	Results
		before-after study		and ceiling points, and collected after spraying for 5 min, 10 min, 20 min, 30 min, and 60 min.	8/12 wall and ceiling samples were positive for bacterial growth.
Doan 2012 (414)	Gram-positive bacilli (*Clostridium difficile*)	Single-site, quasi-experimental, controlled before-after cohort study over 3 months	53 samples collected per intervention from high frequency contact surfaces from hospital environment (bedrails, door handles, light switches, nurse call bell, toilet, bed table, floor) in isolation rooms at Derby Hospital Foundation Trust. Derby, UK	*C. difficile* inoculated into rooms for 72 h. Samples taken prior to disinfection. Disinfection interventions (HPV, dry ozone, 1000 ppm chlorine (sodium dichloroisocyanurate), dry atomized steam, steam cleaning, peracetic acid wipes) were tested each in separate rooms to determine concentration reduction of with known concentration of *C. difficile* spores placed in rooms. Dry ozone (25 ppm, Meditrox 100), high temperature over heated dry atomized steam cleaning (180º C, Polti steam), and steam cleaning (Osprey). Measurements taken after "designated time period specified by company guidelines."	Log10 reductions (in CFU/mL) were highest for hydrogen peroxide, 1000 ppm chlorine-releasing agent, and peracetic acid wipes at 2.303, 2.223, and 2.134 respectively. Log10 (interquartile range) reduction for dry ozone, steam cleaning, and dry atomized steam cleaning were 1.303 (0.805, 2.160), 0.556 (0, 1.161), and 0.527 (0.211, 1.142). Dry ozone was not significantly different than chlorine or peracetic acid but hydrogen peroxide was significantly better than dry ozone. Both steam cleaning interventions were significantly less effective than hydrogen peroxide, chlorine, and peracetic acid.
Sexton 2011 (2707)	Gram-positive bacilli (*Clostridium difficile*)	Single-site quasi experimental, uncontrolled study over 2 days	96 samples from high-touch surfaces (bedrails, tabletops, chair arms, sinks, doors) in 8 rooms from long-term care unit at hospital. Arizona, USA.	Portable saturated steam vapor device with tap water (VaporJet PC 2400) applied to surfaces with light pressure (~10-20 s per surface, 12-15 psi). Samples were taken before and after disinfection.	Percent surfaces positive for *C. difficile* was 2% (1/48) before compared to 0% after disinfection (<0.08 CFU/in^2).
Lowe 2013 (2427)	Gram-positive cocci (*Enterococcus faecalis*)	Single-site quasi-experimental, uncontrolled repeated before-after study	60 samples (bed rails, mattresses, countertops, mounted light fixture, floor, sink) were taken from 6 rooms in biocontainment patient care unit at	The intervention was six repetitions (before-after) of ClO2 (351-385 ppm maintained < 3 h, with exposures of 667-890 ppm-h, Minidox-M Decontamination System) on known concentration ($9x10^9$ CFU)of organism inoculated onto 10 surfaces with samples taken after reduction of ClO2 gas to 0 ppm	The mean log10 reduction (%) for E. *faecalis* (two strains) was 9.02 (99.5%). Significance not specified since complete inactivation for most samples..

Reference (Study ID)	Outcome	Study Design	Setting	Intervention	Results
			medical center. Nebraska, USA		
Oztoprak 2019 (2288)	Gram-positive cocci (*Enterococcus* spp.-Vancomycin-resistant enterococci (VRE))	Single-site quasi experimental, controlled before-after study over one month	5 high-touch surfaces (buttons, bedside table, bed rail, floor) from 3 rooms in 43-bed ICU at tertiary care hospital. Turkey	Each of the following disinfectants was used in one of three rooms: steam technology (Tecnovap Evo 304) compared to two-step cleaning with detergent and water on microfiber cloths followed by 1,000 ppm, or 5,000 ppm hypochlorite solution (sodium dichloroisocyanurate) wipes. Known organism concentration inoculated onto pre-cleaned surface. Samples taken 10 minutes after inoculation and after disinfection.	No bacterial growth after steam or hypochlorite disinfection. Initial concentration not reported.
Sexton 2011 (2707)	Gram-positive cocci (*Staphylococcus aureus*-MISA (Methicillin-intermediate S. *aureus*))	Single-site quasi experimental study over 2 days	32 samples from high-touch surfaces (bedrails, tabletops, chair arms, sinks, doors) in 8 rooms from long-term care unit at hospital. Arizona, USA.	Portable saturated steam vapor device with tap water (VaporJet PC 2400) applied to surfaces with light pressure (~10-20 s per surface, 12-15 psi). Samples were taken before and after disinfection.	Percent surfaces positive for MISA was 25% (12/48) before cleaning compared to 2% (1/48) after cleaning. Initial average concentration ranged from 4.5 to 7.0 CFU/in^2.
Oztoprak 2019 (2288)	Gram-positive cocci (*Staphylococcus aureus*-MRSA)	Single-site quasi experimental, controlled before-after study over one month	5 high-touch surfaces (buttons, bedside table, bed rail, floor) from 3 rooms in 43-bed ICU at tertiary care hospital. Turkey	Each of the following disinfectants was used in one of three rooms: steam technology (Tecnovap Evo 304) compared to two-step cleaning with detergent and water on microfiber cloths followed by 1,000 ppm, or 5,000 ppm hypochlorite solution (sodium dichloroisocyanurate) wipes. Known organism concentration inoculated onto pre-cleaned surface. Samples taken 10 minutes after inoculation and after disinfection.	No bacterial growth after steam or hypochlorite disinfection. Initial concentration not reported.
Sexton 2011 (2707)	Gram-positive cocci	Single-site quasi experimental,	96 samples from high-touch surfaces (bedrails,	Portable saturated steam vapor device with tap water (VaporJet PC 2400) applied to surfaces with light pressure	Percent surfaces positive for MRSA was 6% (3/48) before compared to 0% after.

Reference (Study ID)	Outcome	Study Design	Setting	Intervention	Results
	(*Staphylococcus aureus*-MRSA)	uncontrolled study over 2 days	tabletops, chair arms, sinks, doors) in 8 rooms from long-term care unit at hospital. Arizona, USA.	(~10-20 s per surface, 12-15 psi). Samples were taken before and after disinfection.	Initial concentration of MRSA was <4.0-9.0 CFU/in^2).
Lowe 2013 (2427)	Gram-positive cocci (*Staphylococcus aureus*)	Single-site quasi-experimental, uncontrolled repeated before-after study	60 samples (bed rails, mattresses, countertops, mounted light fixture, floor, sink) were taken from 6 rooms in biocontainment patient care unit at medical center. Nebraska, USA	The intervention was six repetitions (before-after) of ClO2 (351-385 ppm maintained < 3 h, with exposures of 667-890 ppm-h, Minidox-M Decontamination System) on known concentration (9x10^9 CFU)of organism inoculated onto 10 surfaces with samples taken after reduction of ClO2 gas to 0 ppm	The mean log10 reduction (%) for S. *aureus* (two strains) was 8.75 (99.6%). Significance not specified since complete inactivation for most samples.
Nakata 2001 (8147)	Gram-positive cocci (*Staphylococcus aureus*)	Single-Site quasi-experimental, controlled before-after study	~80 samples from easily touched surfaces per disinfectant (floor, wall, shelf, ceilings operating table, surgical lights) in surgical center at Osaka University Medical School. Japan	A fogging disinfection unit compared five chemicals: 0.5% alkyldiaminoethylglycine (Ikeuchi), 0.2% benzalkonium chloride (Nihon), 0.2% acidic electrolytic water sodium hypochlorite (Nippon Shinyaku) and 0.5% glutaral (Maruishi Pharmaceutical), acidic electrolytic water (Bio Japan). Spray time 16-30 min. Samples taken before and ~ 30 min after fogging cycle.	Concentration percent reduction with fogging disinfectants (from highest to lowest) was 96.5% for glutaral. 95.0% for sodium hypochlorite, 94.7% for benzalkonium chloride, 88.9% for acidic electrolytic water, and 88.6% for alkyldiaminoethylglycine. Differences among disinfectants not assessed.
Čamdžić 2019 (6269)	Gram-positive cocci (*Staphylococcus* spp)	Single site, quasi-experimental uncontrolled before-after study	7 samples were taken from surfaces (bed frame, console, respirator monitor, table, floor) in isolation room in tertiary care hospital. Sarajevo,	Intervention was ozone-producing disinfection device (Sterisafe Pro, cycle time 105-180 min). Samples were taken immediately after patient discharge, after standard cleaning, and after the disinfection cycle.	Number of samples positive was 0 after intervention compared to 2 samples positive prior to ozone disinfection. After standard cleaning, concentration ranged from 0 – 100 CFU/cm^2). After ozone, 0 CFU/cm^2 Significance not specified.

Reference (Study ID)	Outcome	Study Design	Setting	Intervention	Results
			Bosnia and Herzegovina		
Ory 2019 (10993)	HAI (HAI – *S. capitis*)	Single-Site, quasi-experimental, uncontrolled before-after study. 5 years	Total of 2518 admitted patients and 37 patient infections or colonizations in two NICUs at hospital. Nimes, France.	Steam cleaning (Sanivap SV2900) intervention. Infection incidence compared retrospectively for four periods: (1) before intervention, (2) with steam cleaning, (3) steam cleaning out of order, (4) with steam cleaning (repaired).	Incidence of infection or colonization was 1.04% before use, 0.55% with use, 3.95% when out-of-order, and 0.0% when steam cleaning returned. Periods with steam cleaning had significantly lower incidence compared to periods without ($p < 0.001$).

References

Afinogenova AG, Kraeva LA, Afinogenov GE, Veretennikov V V. 2017. Probiotic-based sanitation as alternatives to chemical disinfectants. Russ J Infect Immun 7:419–424; doi:10.15789/2220-7619-2017-4-419-424.

Aimiya K, Sato T, Hishida H, Yamaguchi K. 1989. Primary Decontamination Treatments and the Control of Microbial Contamination in a New Ward. J Antibact Antifung Agents 17: 53–56.

Al-Hamad A, Maxwell S. 2008. How clean is clean? Proposed methods for hospital cleaning assessment. J Hosp Infect 70:328–334; doi:10.1016/j.jhin.2008.08.006.

Alekseeva MI, Tsetlin VM, Savel'eva AR, Mal'kov OS, Zakomyrdin AA, Fediaev BP, et al. 1969. [Disinfection in antituberculous institutions]. Probl Tuberk 47.

Alfa MJ, Lo E, Olson N, Macrae M, Buelow-Smith L. 2015. Use of a daily disinfectant cleaner instead of a daily cleaner reduced hospital-Acquired infection rates. Am J Infect Control 43:141–146; doi:10.1016/j.ajic.2014.10.016.

Alhmidi H, Koganti S, Cadnum JL, Rai H, Jencson AL, Donskey CJ. 2017. Evaluation of a novel alcohol-based surface disinfectant for disinfection of hard and soft surfaces in healthcare facilities. Open Forum Infect Dis 4:8–10; doi:10.1093/ofid/ofx054.

Ali S, Muzslay M, Bruce M, Jeanes A, Moore G, Wilson APR. 2016. Efficacy of two hydrogen peroxide vapour aerial decontamination systems for enhanced disinfection of meticillin-resistant Staphylococcus aureus, Klebsiella pneumoniae and Clostridium difficile in single isolation rooms. J Hosp Infect 93:70–77; doi:10.1016/j.jhin.2016.01.016.

Allen O, Jadkauskaite L, Shafi NT, Jackson A, Athithan V, Chiu YD, et al. 2019. Microbiological evaluation of UV disinfection effectiveness in a specialist cystic fibrosis clinic. J Cyst Fibros 18:e37–e39; doi:10.1016/j.jcf.2019.04.019.

Andersen BM, Bånrud H, Bøe E, Bjordal O, Drangsholt F. 2006a. Comparison of UV C Light and Chemicals for Disinfection of Surfaces in Hospital Isolation Units. Infect Control Hosp Epidemiol 27:729–734; doi:10.1086/503643.

Andersen BM, Rasch M, Hochlin K, Jensen FH, Wismar P, Fredriksen JE. 2006b. Decontamination of rooms, medical equipment and ambulances using an aerosol of hydrogen peroxide disinfectant. J Hosp Infect 62:149–155; doi:10.1016/j.jhin.2005.07.020.

Andersen BM, Rasch M, Kvist J, Tollefsen T, Lukkassen R, Sandvik L, et al. 2008. Floor cleaning: effect on bacteria and organic materials in hospital rooms. J Hosp Infect 71:57–65; doi:10.1016/j.jhin.2008.09.014.

Anderson DJ, Chen LF, Weber DJ, Moehring RW, Lewis SS, Triplett PF, et al. 2017. Enhanced terminal room disinfection and acquisition and infection caused by multidrug-resistant organisms and Clostridium difficile (the Benefits of Enhanced Terminal Room Disinfection study): a cluster-randomised, multicentre, crossover study. Lancet 389:805–814; doi:10.1016/S0140-6736(16)31588-4.

Anderson DJ, Moehring RW, Weber DJ, Lewis SS, Chen LF, Schwab JC, et al. 2018. Effectiveness of targeted enhanced terminal room disinfection on hospital-wide acquisition and infection with multidrug-resistant organisms and Clostridium difficile: a secondary analysis of a multicentre cluster randomised controlled trial with crossover. Lancet Infect Dis 18:845–853; doi:10.1016/S1473-3099(18)30278-0.

Armellino D, Goldstein K, Thomas L, Walsh TJ, Petraitis V. 2020. Comparative evaluation of operating room terminal cleaning by two methods: Focused multivector ultraviolet (FMUV) versus manual-chemical disinfection. Am J Infect Control 48:147–152;

doi:10.1016/j.ajic.2019.10.009.
Attaway HH, Fairey S, Steed LL, Salgado CD, Michels HT, Schmidt MG. 2012. Intrinsic bacterial burden associated with intensive care unit hospital beds: Effects of disinfection on population recovery and mitigation of potential infection risk. Am J Infect Control 40:907–912; doi:10.1016/j.ajic.2011.11.019.
Aucella F, Vigilante M, Valente GL, Stallone C. 2000. Systematic monitor disinfection is effective in limiting HCV spread in hemodialysis. Blood Purif 18:110–114; doi:10.1159/000014433.
Barbeito M. 1966. Emergency disinfection of operating room and patient ward with beta-propiolactone. J Am Hosp Assoc 10.
Barbut F, Menuet D, Verachten M, Girou E. 2009. Comparison of the Efficacy of a Hydrogen Peroxide Dry-Mist Disinfection System and Sodium Hypochlorite Solution for Eradication of Clostridium difficile Spores . Infect Control Hosp Epidemiol 30:507–514; doi:10.1086/597232.
Barbut F, Yezli S, Mimoun M, Pham J, Chaouat M, Otter JA. 2013. Reducing the spread of Acinetobacter baumannii and methicillin-resistant Staphylococcus aureus on a burns unit through the intervention of an infection control bundle. Burns 39:395–403; doi:10.1016/j.burns.2012.07.007.
Bates CJ, Pearse R. 2005. Use of hydrogen peroxide vapour for environmental control during a Serratia outbreak in a neonatal intensive care unit. J Hosp Infect 61.
Best EL, Parnell P, Thirkell G, Verity P, Copland M, Else P, et al. 2014. Effectiveness of deep cleaning followed by hydrogen peroxide decontamination during high Clostridium difficile infection incidence. J Hosp Infect 87:25–33; doi:10.1016/j.jhin.2014.02.005.
Biswal M, Rudramurthy S, Jain N, Shamanth A, Sharma D, Jain K, et al. 2017. Controlling a possible outbreak of Candida auris infection: lessons learnt from multiple interventions. J Hosp Infect 97: 363–370.
Blazejewski C, Wallet F, Rouzé A, Le Guern R, Ponthieux S, Salleron J, et al. 2015. Efficiency of hydrogen peroxide in improving disinfection of ICU rooms. Crit Care 19:30; doi:10.1186/s13054-015-0752-9.
Bogusz A, Stewart M, Hunter J, Yip B, Reid D, Robertson C, et al. 2013. How quickly do hospital surfaces become contaminated after detergent cleaning? Healthc Infect 18:3–9; doi:10.1071/HI12063.
Bokulich NA, Mills DA, Underwood MA. 2013. Surface microbes in the neonatal intensive care unit: Changes with routine cleaning and over time. J Clin Microbiol 51:2617–2624; doi:10.1128/JCM.00898-13.
Boyce JM, Guercia KA, Sullivan L, Havill NL, Fekieta R, Kozakiewicz J, et al. 2017. Prospective cluster controlled crossover trial to compare the impact of an improved hydrogen peroxide disinfectant and a quaternary ammonium-based disinfectant on surface contamination and health care outcomes. Am J Infect Control 45:1006–1010; doi:10.1016/j.ajic.2017.03.010.
Boyce JM, Havill NL. 2013. Evaluation of a New Hydrogen Peroxide Wipe Disinfectant Author (s): John M . Boyce MD and Nancy L . Havill MT CIC Source : Infection Control and Hospital Epidemiology , Vol . 34 , No . 5 , Special Topic Issue : The Role of the Environment in Infection P. 34: 521–523.
Boyce JM, Havill NL, Guercia KA, Schweon SJ, Moore BA. 2014. Evaluation of two organosilane products for sustained antimicrobial activity on high-touch surfaces in patient

rooms. Am J Infect Control 42:326–328; doi:10.1016/j.ajic.2013.09.009.
Boyce JM, Havill NL, Otter JA, McDonald LC, Adams NM, Cooper T, et al. Impacts of hydrogen peroxide vapor room decontamination on Clostridium difficile environmental contamination and transmission in a healthcare setting. Infect Control Hosp Epidemiol.
Butin M, Dumont Y, Monteix A, Raphard A, Roques C, Martins Simoes P, et al. 2019. Sources and reservoirs of Staphylococcus capitis NRCS-A inside a NICU. Antimicrob Resist Infect Control 8:1–7; doi:10.1186/s13756-019-0616-1.
Byers KE, Durbin LJ, Simonton BM, Anglim AM, Adal A, Farr BM, et al. 2020. Disinfection of Hospital Rooms Contaminated with Vancomycin-Resistant Enterococcus faecium Published by : Cambridge University Press on behalf of The Society for Healthcare Epidemiology of America Stable URL : https://www.jstor.org/stable/30142418 REFEREN. 19.
Čamdžić A, Dedeić-Ljubović A, Madacki-Todorović K. 2019. Using desinfection devices in intensive care units. Acta Medica Salin 49:191–194; doi:10.5457/525.
Carling PC, Perkins J, Ferguson J, Thomasser A. 2014. Evaluating a New Paradigm for Comparing Surface Disinfection in Clinical Practice. Infect Control Hosp Epidemiol 35:1349–1355; doi:10.1086/678424.
Casey AL, Adams D, Karpanen TJ, Lambert PA, Cookson BD, Nightingale P, et al. 2010. Role of copper in reducing hospital environment contamination. J Hosp Infect 74:72–77; doi:10.1016/j.jhin.2009.08.018.
Casini B, Righi A, De Feo N, Totaro M, Giorgi S, Zezza L, et al. 2018a. Improving cleaning and disinfection of high-touch surfaces in intensive care during carbapenem-resistant acinetobacter baumannii endemo-epidemic situations. Int J Environ Res Public Health 15; doi:10.3390/ijerph15102305.
Casini B, Selvi C, Cristina ML, Totaro M, Costa AL, Valentini P, et al. 2017. Evaluation of a modified cleaning procedure in the prevention of carbapenem-resistant Acinetobacter baumannii clonal spread in a burn intensive care unit using a high-sensitivity luminometer. J Hosp Infect 95:46–52; doi:10.1016/j.jhin.2016.10.019.
Casini B, Tuvo B, Cristina ML, Spagnolo AM, Totaro M, Baggiani A, et al. 2019. Evaluation of an Ultraviolet C (UVC) Light-Emitting Device for Disinfection of High Touch Surfaces in Hospital Critical Areas. Int J Environ Res Public Health 16.
Casini B, Tuvo B, Totaro M, Aquino F, Baggiani A, Privitera G. 2018b. Evaluation of the cleaning procedure efficacy in prevention of nosocomial infections in healthcare facilities using cultural method associated with high sensitivity luminometer for ATP detection. Pathogens 7:1–9; doi:10.3390/pathogens7030071.
Chan HT, White P, Sheorey H, Cocks J, Waters MJ. 2011. Evaluation of the biological efficacy of hydrogen peroxide vapour decontamination in wards of an Australian hospital. J Hosp Infect 79:125–128; doi:10.1016/j.jhin.2011.06.009.
Chen CH, Tu CC, Kuo HY, Zeng RF, Yu CS, Lu HHS, et al. 2017. Dynamic change of surface microbiota with different environmental cleaning methods between two wards in a hospital. Appl Microbiol Biotechnol 101:771–781; doi:10.1007/s00253-016-7846-4.
Codish S, Toledano R, Novack V, Sherf M, Borer A. 2015. Effectiveness of stringent decontamination of computer input devices in the era of electronic medical records and bedside computing: A randomized controlled trial. Am J Infect Control 43:644–646; doi:10.1016/j.ajic.2014.09.011.
Coppin JD, Villamaria FC, Williams MD, Copeland LA, Zeber JE, Jinadatha C. 2017. Self-

sanitizing copper-impregnated surfaces for bioburden reduction in patient rooms. Am J Infect Control 45:692–694; doi:10.1016/j.ajic.2017.01.012.
Danforth D, Nicolle LE, Hume K, Alfieri N, Sims H. 1987. Nosocomial infections on nursing units with floors cleaned with a disinfectant compared with detergent. J Hosp Infect 10:229–235; doi:10.1016/0195-6701(87)90002-8.
Daschner F, Rabbenstein G, Langmaack H. 1980. Surface decontamination in the control of hospital infections: Comparison of different methods. Dtsch Medizinische Wochenschrift 105:325–329; doi:10.1055/s-2008-1070659.
de Jong B, Meeder AM, Koekkoek KWAC, Schouten MA, Westers P, van Zanten ARH. 2018. Pre–post evaluation of effects of a titanium dioxide coating on environmental contamination of an intensive care unit: the TITANIC study. J Hosp Infect 99:256–262; doi:10.1016/j.jhin.2017.04.008.
Deshpande A, Mba TSCM, Cadnum JL, Bs ACJ, Ms BS, Fertelli D, et al. 2014. Evaluation of a Sporicidal Peracetic Acid / Hydrogen Peroxide – Based Daily Disinfectant Cleaner Published by : Cambridge University Press on behalf of The Society for Healthcare Epidemiology of America Stable URL : https://www.jstor.org/stable/10.1086/67. 5–8.
Dharan S, Mourouga P, Copin P, Bessmer G, Tschanz B, Pittet D. 1999. Routine disinfection of patients' environmental surfaces. Myth or reality? J Hosp Infect 42:113–117; doi:10.1053/jhin.1999.0567.
Doan L, Forrest H, Fakis A, Craig J, Claxton L, Khare M. 2012. Clinical and cost effectiveness of eight disinfection methods for terminal disinfection of hospital isolation rooms contaminated with Clostridium difficile 027. J Hosp Infect 82:114–121; doi:10.1016/j.jhin.2012.06.014.
Doidge M, Allworth AM, Woods M, Marshall P, Terry M, Brien KO, et al. 2010. Control of an Outbreak of Carbapenem - Resistant Acinetobacter baumannii in Australia after Introduction of Environmental Cleaning with a Commercial Oxidizing Disinfectant Published by : Cambridge University Press on behalf of The Society for Healthcare E. 17–20.
Dramowski A, Whitelaw A, Cotton MF. 2016. Assessment of terminal cleaning in pediatric isolation rooms: Options for low-resource settings. Am J Infect Control 44:1558–1564; doi:10.1016/j.ajic.2016.05.026.
Dunklin EW, Lester W. 1959. Residual surface disinfection ii. the effect of orthophenylphenol treatment of the floor on bacterial contamination in a recovery room. J Infect Dis 104:41–55; doi:10.1093/infdis/104.1.41.
Dyas A, Boughton BJ, Das BC. 1983. Ozone killing action against bacterial and fungal species; microbiological testing of a domestic ozone generator. J Clin Pathol 36:1102–1104; doi:10.1136/jcp.36.10.1102.
Eckstein BC, Adams DA, Eckstein EC, Rao A, Sethi AK, Yadavalli GK, et al. 2007. Reduction of Clostridium Difficile and vancomycin-resistant Enterococcus contamination of environmental surfaces after an intervention to improve cleaning methods. BMC Infect Dis 7:1–6; doi:10.1186/1471-2334-7-61.
Edmiston CE, Spencer M, Lewis BD, Rossi PJ, Brown KR, Malinowski M, et al. 2020. Assessment of a novel antimicrobial surface disinfectant on inert surfaces in the intensive care unit environment using ATP-bioluminesence assay. Am J Infect Control 48:143–146; doi:10.1016/j.ajic.2019.08.026.
Esolen LM, Thakur L, Layon AJ, Fuller TA, Harrington DJ, Jha K, et al. 2018. The efficacy of

self-disinfecting bedrail covers in an intensive care unit. Am J Infect Control 46:417–419; doi:10.1016/j.ajic.2017.09.026.
Evans MW, Breshears J, Campbell A, Husbands C, Rupert R. 2007. Assessment and risk reduction of infectious pathogens on chiropractic treatment tables. Chiropr Osteopat 15:1–5; doi:10.1186/1746-1340-15-8.
Exner M, Vogel F, Hamann R. 1982. Surface disinfection in a medical intensive care unit. Intensivmedizin 19: 26–29.
Fattorini M, Buonocore G, Lenzi D, Burgassi S, Cardaci RMR, Biermann KP, et al. 2018. Public Health since the beginning: Neonatal incubators safety in a clinical setting. J Infect Public Health 11:788–792; doi:10.1016/j.jiph.2018.03.001.
Ferreira AM, de Andrade D, Rigotti MA, de Almeida MTG, Guerra OG, dos Santos Junior AG. 2015. Assessment of disinfection of hospital surfaces using different monitoring methods. Rev Lat Am Enfermagem 23:466–474; doi:10.1590/0104-1169.0094.2577.
Fitton K, Barber KR, Karamon A, Zuehlke N, Atwell S, Enright S. 2017. Long-acting water-stable organosilane agent and its sustained effect on reducing microbial load in an intensive care unit. Am J Infect Control 45:1214–1217; doi:10.1016/j.ajic.2017.06.014.
Frabetti A, Vandini A, Balboni P, Triolo F, Mazzacane S. 2009. Experimental evaluation of the efficacy of sanitation procedures in operating rooms. Am J Infect Control 37:658–664; doi:10.1016/j.ajic.2009.03.011.
French GL, Otter JA, Shannon KP, Adams NMT, Watling D, Parks MJ. 2004. Tackling contamination of the hospital environment by methicillin-resistant Staphylococcus aureus (MRSA): A comparison between conventional terminal cleaning and hydrogen peroxide vapour decontamination. J Hosp Infect 57:31–37; doi:10.1016/j.jhin.2004.03.006.
Frota OP, Ferreira AM, Guerra OG, Rigotti MA, de Andrade D, Borges NMA, et al. 2017. Efficiency of cleaning and disinfection of surfaces: correlation between assessment methods. Rev Bras Enferm 70: 1176–1183.
Fujii M. 1996. Prevention of MRSA infection in neurosurgery : Examination from the patient environment *.
Fukada T, Iwakiri H, Ozaki M. 2008. Anaesthetists' role in computer keyboard contamination in an operating room. J Hosp Infect 70:148–153; doi:10.1016/j.jhin.2008.05.023.
Fukada T, Tsuchiya Y, Iwakiri H, Ozaki M. 2015. Adenosine triphosphate bioluminescence assay for monitoring contamination of the working environment of anaesthetists and cleanliness of the operating room. J Infect Prev 16:8–13; doi:10.1177/1757177414553492.
Furlan MCR, Ferreira AM, Rigotti MA, Guerra OG, Frota OP, De Sousa AFL, et al. 2019. Correlation among monitoring methods of surface cleaning and disinfection in outpatient facilities. Acta Paul Enferm 32: 282–289.
Gable TS. 1966. Bactericidal effectiveness of floor cleaning methods in a hospital environment. Hospitals (Lond) 40: 107–111.
Galván Contreras R-, Tapia RAR, Cervantes ES, Aguilar RMAC. 2017. Comparative study on the effectiveness of 6% sodium hypochlorite solution vs a bromine-chloro-dimethylhydantoin solution for disinfecting hospital environments. Perinatol y Reprod Humana 30: 145–150.
Gan T, Xu H, Wu J, Zhu Y, Wang L, Jin H, et al. 2017. Sequential enhanced cleaning eliminates multidrug-resistant organisms in general intensive care unit of a traditional Chinese medicine hospital. J Crit Care 41:216–221; doi:10.1016/j.jcrc.2017.04.036.
Garvey MI, Bradley CW, Jumaa P. 2016. Environmental decontamination following

occupancy of a burns patient with multiple carbapenemase-producing organisms. J Hosp Infect 93:136–140; doi:10.1016/j.jhin.2016.01.006.

Garvey MI, Wilkinson MAC, Bradley CW, Holden KL, Holden E. 2018. Wiping out MRSA: Effect of introducing a universal disinfection wipe in a large UK teaching hospital. Antimicrob Resist Infect Control 7:1–9; doi:10.1186/s13756-018-0445-7.

Gelmini F, Belotti L, Vecchi S, Testa C, Beretta G. 2016. Air dispersed essential oils combined with standard sanitization procedures for environmental microbiota control in nosocomial hospitalization rooms. Complement Ther Med 25:113–119; doi:10.1016/j.ctim.2016.02.004.

Ghantoji SS, Stibich M, Stachowiak J, Cantu S, Adachi JA, Raad II, et al. 2015. Non-inferiority of pulsed xenon UV light versus bleach for reducing environmental Clostridium difficile contamination on high-touch surfaces in Clostridium difficile infection isolation rooms. J Med Microbiol 64:191–194; doi:10.1099/jmm.0.000004.

Goldenberg SD, Patel A, Tucker D, French GL. 2012. Lack of enhanced effect of a chlorine dioxide-based cleaning regimen on environmental contamination with Clostridium difficile spores. J Hosp Infect 82:64–67; doi:10.1016/j.jhin.2012.06.004.

Gonzalez S, Illescas A, Escarzaga E. 2012. Reduction of Bacterial Contamination of the Environment of a General Hospital, By the Use of a new Germicide Biomet 66. Современные Проблемы Науки И Образования.

Hacek DM, Ascp MT, Ogle AM, Fisher A, Ascp MT, Robicsek A. 2010a. Significant impact of terminal room cleaning with bleach on reducing nosocomial Clostridium difficile. Am J Infect Control 38:350–353; doi:10.1016/j.ajic.2009.11.003.

Hacek DM, Ogle AM, Fisher A, Robicsek A, Peterson LR. 2010b. Significant impact of terminal room cleaning with bleach on reducing nosocomial Clostridium difficile. Am J Infect Control 38:350–353; doi:10.1016/j.ajic.2009.11.003.

Hall TJ, Jeanes A, McKain LW, Jepson MJ, Coen PG, Hickok SS, et al. 2011. A UK district general hospital cleaning study: A comparison of the performance of ultramicrofibre technology with or without addition of a novel copper-based biocide with standard hypochlorite-based cleaning. J Infect Prev 12:232–236; doi:10.1177/1757177411411509.

Hamilton D, Foster A, Ballantyne L, Kingsmore P, Bedwell D, Hall TJ, et al. 2010. Performance of ultramicrofibre cleaning technology with or without addition of a novel copper-based biocide. J Hosp Infect 74:62–71; doi:10.1016/j.jhin.2009.08.006.

Hardy KJ, Gossain S, Henderson N, Drugan C, Oppenheim BA, Gao F, et al. 2007. Rapid recontamination with MRSA of the environment of an intensive care unit after decontamination with hydrogen peroxide vapour. J Hosp Infect 66:360–368; doi:10.1016/j.jhin.2007.05.009.

Havill NL, Moore BA, Boyce JM. 2012. Comparison of the Microbiological Efficacy of Hydrogen Peroxide Vapor and Ultraviolet Light Processes for Room Decontamination. Infect Control Hosp Epidemiol 33:507–512; doi:10.1086/665326.

Hedin G, Rynbäck J, Loré B. 2010. Reduction of bacterial surface contamination in the hospital environment by application of a new product with persistent effect. J Hosp Infect 75:112–115; doi:10.1016/j.jhin.2010.02.007.

Hinsa-Leasure SM, Nartey Q, Vaverka J, Schmidt MG. 2016. Copper alloy surfaces sustain terminal cleaning levels in a rural hospital. Am J Infect Control 44:e195–e203; doi:10.1016/j.ajic.2016.06.033.

Ho YH, Wang LS, Jiang HL, Chang CH, Hsieh CJ, Chang DC, et al. 2016a. Use of a sampling area-adjusted adenosine triphosphate bioluminescence assay based on digital image

quantification to assess the cleanliness of hospital surfaces. Int J Environ Res Public Health 13:1–12; doi:10.3390/ijerph13060576.

Ho YH, Wang LS, Jiang HL, Chang CH, Hsieh CJ, Chang DC, et al. 2016b. Use of a sampling area-adjusted adenosine triphosphate bioluminescence assay based on digital image quantification to assess the cleanliness of hospital surfaces. Int J Environ Res Public Health 13:1–12; doi:10.3390/ijerph13060576.

Holmdahl T, Walder M, Uzcátegui N, Odenholt I, Lanbeck P, Medstrand P, et al. 2016. Hydrogen peroxide vapor decontamination in a patient room using feline Calicivirus and Murine Norovirus as surrogate markers for human norovirus. Infect Control Hosp Epidemiol 37:561–566; doi:10.1017/ice.2016.15.

Hosein I, Madeloso R, Nagaratnam W, Villamaria F, Stock E, Jinadatha C. 2016a. Evaluation of a pulsed xenon ultraviolet light device for isolation room disinfection in a United Kingdom hospital. Am J Infect Control 44:e157–e161; doi:10.1016/j.ajic.2016.01.044.

Hosein I, Madeloso R, Nagaratnam W, Villamaria F, Stock E, Jinadatha C. 2016b. Evaluation of a pulsed xenon ultraviolet light device for isolation room disinfection in a United Kingdom hospital. Am J Infect Control 44:e157–e161; doi:10.1016/j.ajic.2016.01.044.

Huang YS, Chen YC, Chen ML, Cheng A, Hung IC, Wang JT, et al. 2015. Comparing visual inspection, aerobic colony counts, and adenosine triphosphate bioluminescence assay for evaluating surface cleanliness at a medical center. Am J Infect Control 43:882–886; doi:10.1016/j.ajic.2015.03.027.

Humayun T, Qureshi A, Al Roweily SF, Carig J, Humayun F. 2019. Efficacy Of Hydrogen Peroxide Fumigation In Improving Disinfection Of Hospital Rooms And Reducing The Number Of Microorganisms. J Ayub Med Coll Abbottabad 31 1): S646–S650.

Inkinen J, Mäkinen R, Keinänen-Toivola MM, Nordström K, Ahonen M. 2017. Copper as an antibacterial material in different facilities. Lett Appl Microbiol 64:19–26; doi:10.1111/lam.12680.

Jinadatha C, Quezada R, Huber TW, Williams JB, Zeber JE, Copeland LA. 2014. Evaluation of a pulsed-xenon ultraviolet room disinfection device for impact on contamination levels of methicillin-resistant Staphylococcus aureus. BMC Infect Dis 14:1–7; doi:10.1186/1471-2334-14-187.

Johnson A, Weston L, Grisewood L, Kyffin M. 2016. Evaluation of the Ultra-V™ (ultraviolet) decontamination system as an adjunct to cleaning in a district general hospital. J Hosp Infect 94:406–407; doi:10.1016/j.jhin.2016.07.002.

Jones R, Hutton A, Mariyaselvam M, Hodges E, Wong K, Blunt M, et al. 2015. Keyboard cleanliness: A controlled study of the residual effect of chlorhexidine gluconate. Am J Infect Control 43:289–291; doi:10.1016/j.ajic.2014.12.002.

Kaatz GW, Gitlin SD, Schaberg DR, Wilson KH, Kauffman CA, Seo SM, et al. 1988. Acquisition of Clostridium difficile from the hospital environment. Am J Epidemiol 127:1289–1294; doi:10.1093/oxfordjournals.aje.a114921.

Karpanen TJ, Casey AL, Lambert PA, Cookson BD, Nightingale P, Miruszenko L, et al. 2012. The Antimicrobial Efficacy of Copper Alloy Furnishing in the Clinical Environment: A Crossover Study. Infect Control Hosp Epidemiol 33:3–9; doi:10.1086/663644.

Karunanayake LI, Waniganayake YC, Nirmala Gunawardena KD, Danuka Padmaraja SA, Peter D, Jayasekera R, et al. 2019. Use of silicon nanoparticle surface coating in infection control: Experience in a tropical healthcare setting. Infect Dis Heal 24:201–207; doi:10.1016/j.idh.2019.06.006.

Kim MH, Lee SG, Kim KS, Heo YJ, Oh JE, Jeong SJ. 2018. Environmental disinfection with photocatalyst as an adjunctive measure to control transmission of methicillin-resistant Staphylococcus aureus: A prospective cohort study in a high-incidence setting 11 Medical and Health Sciences 1117 Public Health and Hea. BMC Infect Dis 18:1–10; doi:10.1186/s12879-018-3555-1.

Kitagawa H, Mori M, Kashiyama S, Sasabe Y, Ukon K, Shimokawa N, et al. 2020. Effect of pulsed xenon ultraviolet disinfection on methicillin-resistant Staphylococcus aureus contamination of high-touch surfaces in a Japanese hospital. Am J Infect Control 48:139–142; doi:10.1016/j.ajic.2019.08.033.

Le Coutour C, Oblin I. 1991. Disinfection of surfaces in hospital: Comparison between theoric and real efficiency of three commercial products.

Lee W Sen, Hsieh TC, Shiau JC, Ou TY, Chen FL, Liu YH, et al. 2017. Bio-Kil, a nano-based disinfectant, reduces environmental bacterial burden and multidrug-resistant organisms in intensive care units. J Microbiol Immunol Infect 50:737–746; doi:10.1016/j.jmii.2016.04.008.

Lerner AO, Abu-Hanna J, Carmeli Y, Schechner V. 2019. Environmental contamination by carbapenem-resistant Acinetobacter baumannii: The effects of room type and cleaning methods. Infect Control Hosp Epidemiol 41:166–171; doi:10.1017/ice.2019.307.

Lewis BD, Spencer M, Rossi PJ, Lee CJ, Brown KR, Malinowski M, et al. 2015. Assessment of an innovative antimicrobial surface disinfectant in the operating room environment using adenosine triphosphate bioluminescence assay. Am J Infect Control 43:283–285; doi:10.1016/j.ajic.2014.11.023.

Lewis T, Griffith C, Gallo M, Weinbren M. 2008. A modified ATP benchmark for evaluating the cleaning of some hospital environmental surfaces. J Hosp Infect 69:156–163; doi:10.1016/j.jhin.2008.03.013.

Lowe JJ, Gibbs SG, Iwen PC, Smith PW, Hewlett AL. 2013a. Decontamination of a hospital room using gaseous chlorine dioxide: Bacillus anthracis, francisella tularensis, and Yersinia pestis. J Occup Environ Hyg 10:533–539; doi:10.1080/15459624.2013.818241.

Lowe JJ, Gibbs SG, Iwen PC, Smith PW, Hewlett AL. 2013b. Impact of chlorine dioxide gas sterilization on nosocomial organism viability in a hospital room. Int J Environ Res Public Health 10:2596–2605; doi:10.3390/ijerph10062596.

Manian FA, Griesenauer S, Senkel D, Setzer JM, Doll SA, Perry AM, et al. 2011. Isolation of Acinetobacter baumannii Complex and Methicillin-Resistant Staphylococcus aureus from Hospital Rooms Following Terminal Cleaning and Disinfection: Can We Do Better? . Infect Control Hosp Epidemiol 32:667–672; doi:10.1086/660357.

Manian FA, Griesnauer S, Bryant A. 2013a. Implementation of hospital-wide enhanced terminal cleaning of targeted patient rooms and its impact on endemic Clostridium difficile infection rates. Am J Infect Control 41:537–541; doi:10.1016/j.ajic.2012.06.014.

Manian FA, Griesnauer S, Bryant A. 2013b. Implementation of hospital-wide enhanced terminal cleaning of targeted patient rooms and its impact on endemic Clostridium difficile infection rates. Am J Infect Control 41:537–541; doi:10.1016/j.ajic.2012.06.014.

Marais F, Mehtar S, Chalkley L. 2010. Antimicrobial efficacy of copper touch surfaces in reducing environmental bioburden in a South African community healthcare facility. J Hosp Infect 74:80–82; doi:10.1016/j.jhin.2009.07.010.

Mayfield JL, Leet T, Miller J, Mundy LM. 2019. Environmental Control to Reduce Transmission of Clostridium difficile Author (s): Jennie L . Mayfield , Terry Leet , James Miller and

Linda M . Mundy Published by : Oxford University Press Stable URL : https://www.jstor.org/stable/4461350 Environmental. Clin Infect Dis 31: 995–1000.
Mccord J, Prewitt M, Dyakova E, Mookerjee S, Otter JA. 2016. Reduction in Clostridium difficile infection associated with the introduction of hydrogen peroxide vapour automated room disinfection. J Hosp Infect 94:185–187; doi:10.1016/j.jhin.2016.05.014.
Meinke R, Meyer B, Frei R, Passweg J, Widmer AF. 2012. Equal Efficacy of Glucoprotamin and an Aldehyde Product for Environmental Disinfection in a Hematologic Transplant Unit: A Prospective Crossover Trial. Infect Control Hosp Epidemiol 33:1077–1080; doi:10.1086/668028.
Mitchell BG, Digney W, Locket P, Dancer SJ. 2014. Controlling methicillin-resistant Staphylococcus aureus (MRSA) in a hospital and the role of hydrogen peroxide decontamination: An interrupted time series analysis. BMJ Open 4:1–7; doi:10.1136/bmjopen-2013-004522.
Montero DA, Arellano C, Pardo M, Vera R, Gálvez R, Cifuentes M, et al. 2019. Antimicrobial properties of a novel copper-based composite coating with potential for use in healthcare facilities. Antimicrob Resist Infect Control 8:1–10; doi:10.1186/s13756-018-0456-4.
Mosci D, Marmo GW, Sciolino L, Zaccaro C, Antonellini R, Accogli L, et al. 2017. Automatic environmental disinfection with hydrogen peroxide and silver ions versus manual environmental disinfection with sodium hypochlorite: a multicentre randomized before-and-after trial. J Hosp Infect 97:175–179; doi:10.1016/j.jhin.2017.06.010.
Munster AM, Ostrander WE. 1974. Terminal disinfection of contaminated patient care areas: to fog or not to fog? Am Surg 40: 713–715.
Nagai I, Kadota M, Matsuoka K, Jitsukawa S. 1983. Evaluation of Chemical Aerosol Spray Disinfection in the Operating Room. Med J Osaka Univ 34: 27–31.
Nakata S, Ikeda T, Nakatani H, Sakamoto M, Higashidutsumi M, Honda T, et al. 2001. Evaluation of an automatic fogging disinfection unit. Environ Health Prev Med 6:160–164; doi:10.1265/ehpm.2001.160.
Ogino J, Fujimori I, Goto R, Hisamastu K, Murakami Y, Yamada T, et al. 1995. Efficacy of pyoktanin and DF-100 for prevention of nosocomial MRSA infection. Pract Otol Suppl 104–109; doi:10.5631/jibirinsuppl1986.1995.supplement79_104.
Oie S, Yanagi C, Matsui H, Nishida T, Tomita M, Kamiya A. 2005. Contamination of environmental surfaces by Staphylococcus aureus in a dermatological ward and its preventive measures. Biol Pharm Bull 28:120–123; doi:10.1248/bpb.28.120.
Ojajärvi J, Mäkelä P. 1976. Evaluation of Chlorine Compounds for Surface Disinfection by Laboratory and ln-use Testing. Scand J Infect Dis 8:267–270; doi:10.3109/inf.1976.8.issue-4.09.
Oon A, Reading E, Ferguson JK, Dancer SJ, Mitchell BG. 2020. Measuring environmental contamination in critical care using dilute hydrogen peroxide (DHP) technology: An observational cross-over study. Infect Dis Heal 25:107–112; doi:10.1016/j.idh.2019.12.005.
Orenstein R, Aronhalt KC, Rn MA, Jr JEM, Fedraw LA, Ma PMP, et al. 2011. A Targeted Strategy to Wipe Out Clostridium difficile Published by : Cambridge University Press on behalf of The Society for Healthcare Epidemiology of America Stable URL : https://www.jstor.org/stable/10.1086/662586 A Targeted Strategy to Wipe Out Clostr. 32: 1137–1139.
Ortí-Lucas RM, Muñoz-Miguel J. 2017. Effectiveness of surface coatings containing silver ions in bacterial decontamination in a recovery unit. Antimicrob Resist Infect Control 6:1–8;

doi:10.1186/s13756-017-0217-9.
Ory J, Cazaban M, Richaud-Morel B, Di Maio M, Dunyach-Remy C, Pantel A, et al. 2019. Successful implementation of infection control measure in a neonatal intensive care unit to combat the spread of pathogenic multidrug resistant Staphylococcus capitis. Antimicrob Resist Infect Control 8:1–7; doi:10.1186/s13756-019-0512-8.
Otter JA, Cummins M, Ahmad F, van Tonder C, Drabu YJ. 2007. Assessing the biological efficacy and rate of recontamination following hydrogen peroxide vapour decontamination. J Hosp Infect 67:182–188; doi:10.1016/j.jhin.2007.07.019.
Otter JA, Mepham S, Athan B, Mack D, Smith R, Jacobs M, et al. 2016. Terminal decontamination of the Royal Free London's high-level isolation unit after a case of Ebola virus disease using hydrogen peroxide vapor. Am J Infect Control 44:233–235; doi:10.1016/j.ajic.2015.08.025.
Otter JA, Yezli S, Schouten MA, Van Zanten ARH, Houmes-Zielman G, Nohlmans-Paulssen MKE. 2010. Hydrogen peroxide vapor decontamination of an intensive care unit to remove environmental reservoirs of multidrug-resistant gram-negative rods during an outbreak. Am J Infect Control 38:754–756; doi:10.1016/j.ajic.2010.03.010.
Özpolat B, Çavuşoğlu T, Yilmaz S, Büyükkoçak Ü, Günaydin S. 2011a. Clinical and laboratory evaluation of anti-microbial efficacy of photocataylsts. J Clin Anal Med 2:32–35; doi:10.4328/JCAM.262.
Özpolat B, Çavuşoğlu T, Yilmaz S, Büyükkoçak Ü, Günaydin S. 2011b. Fotokatalizörlerin anti-mikrobiyal etkinliğinin klinik ve laboratuar ortamda değerlendirilmesi. J Clin Anal Med 2:32–35; doi:10.4328/JCAM.262.
Oztoprak N, Kizilates F, Percin D. 2019. Comparison of steam technology and a two-step cleaning (water/detergent) and disinfecting (1,000 resp. 5,000 ppm hypochlorite) method using microfiber cloth for environmental control of multidrug-resistant organisms in an intensive care unit. GMS Hyg Infect Control 14:Doc15; doi:10.3205/dgkh000330.
Page MJ, McKenzie JE, Bossuyt PM, Boutron I, Hoffmann TC, Mulrow CD, et al. The PRISMA 2020 statement: an updated guideline for reporting systematic reviews. BMJ 2021;372:n71. doi: 10.1136/bmj.n71
Panknin H. 2014. Diversity of the ambient flora and effectiveness of surface disinfection measures in the neonatal unit. Hyg + Medizin 39: 245–247.
Passaretti CL, Otter JA, Reich NG, Myers J, Shepard J, Ross T, et al. 2013. An evaluation of environmental decontamination with hydrogen peroxide vapor for reducing the risk of patient acquisition of multidrug-resistant organisms. Clin Infect Dis 56:27–35; doi:10.1093/cid/cis839.
Patel SS, Pevalin DJ, Prosser R, Couchman A. 2007. Comparison of detergent-based cleaning, disinfectant-based cleaning, and detergent-based cleaning after enhanced domestic staff training within a source isolation facility. Br J Infect Control 8:20–25; doi:10.1177/1469044607008003070l.
Popov DA, Anuchina NM. 2016. Microbiological Efficacy of Hospital Environment Decontamination by Hydrogen Peroxide Aerosol. Biomed Eng (NY) 50:92–95; doi:10.1007/s10527-016-9595-1.
Prindis V, Michalek J, Kubatova I. 2018. Application of photocatalytic nanolayers SmartCoat in health care facility. EMBEC NBC 65:1089–1090; doi:10.1007/978-981-10-5122-7.
Rai S, Hirsch B, Attaway H, Nadan R, Fairey S, Hardy J, et al. 2012. Evaluation of the Antimicrobial Properties of Copper Surfaces in an Outpatient Infectious Disease Practice

Author (s): Seema Rai MD , Bruce E . Hirsch MD , Hubert H . Attaway MS MBA , Richard Nadan G . Schmidt PhD Published by : Cambridge University Pre. Infect Control Hosp Epidemiol 33: 200–201.

Rathod SN, Beauvais K, Sullivan LK, Sudikoff SN, Peaper DR, Martinello RA. 2019. The effectiveness of a novel colorant additive in the daily cleaning of patient rooms. Infect Control Hosp Epidemiol 40:721–723; doi:10.1017/ice.2019.67.

Ray A, Perez F, Beltramini AM, Jakubowycz M, Dimick P, Jacobs MR, et al. 2010. Use of Vaporized Hydrogen Peroxide Decontamination during an Outbreak of Multidrug-Resistant Acinetobacter baumannii Infection at a Long-Term Acute Care Hospital . Infect Control Hosp Epidemiol 31:1236–1241; doi:10.1086/657139.

Reid M, Whatley V, Spooner E, Nevill AM, Cooper M, Ramsden JJ, et al. 2018. How Does a Photocatalytic Antimicrobial Coating Affect Environmental Bioburden in Hospitals? Infect Control Hosp Epidemiol 39:398–404; doi:10.1017/ice.2017.297.

Reynolds KA, Sexton JD, Pivo T, Humphrey K, Leslie RA, Gerba CP. 2019. Microbial transmission in an outpatient clinic and impact of an intervention with an ethanol-based disinfectant. Am J Infect Control 47:128–132; doi:10.1016/j.ajic.2018.06.017.

Roux D, Aubier B, Cochard H, Quentin R, Van Der Mee-Marquet N. 2013. Contaminated sinks in intensive care units: An underestimated source of extended-spectrum beta-lactamase-producing Enterobacteriaceae in the patient environment. J Hosp Infect 85:106–111; doi:10.1016/j.jhin.2013.07.006.

Rutala WA, Kanamori H, Gergen MF, Knelson LP, Sickbert-Bennett EE, Chen LF, et al. 2018. Enhanced disinfection leads to reduction of microbial contamination and a decrease in patient colonization and infection. Infect Control Hosp Epidemiol 39:1118–1121; doi:10.1017/ice.2018.165.

Saha A, Botha SL, Weaving P, Satta G. 2016. A pilot study to assess the effectiveness and cost of routine universal use of peracetic acid sporicidal wipes in a real clinical environment. Am J Infect Control 44:1247–1251; doi:10.1016/j.ajic.2016.03.046.

Salgado CD, Sepkowitz KA, John JF, Cantey JR, Attaway HH, Freeman KD, et al. 2013. Copper Surfaces Reduce the Rate of Healthcare-Acquired Infections in the Intensive Care Unit. Infect Control Hosp Epidemiol 34:479–486; doi:10.1086/670207.

Santos-Junior AG, Ferreira AM, Frota OP, Rigotti MA, Barcelos L da S, Lopes de Sousa AF, et al. 2018. Effectiveness of Surface Cleaning and Disinfection in a Brazilian Healthcare Facility. Open Nurs J 12:36–44; doi:10.2174/1874434601812010036.

Schmidt MG, Attaway HH, Fairey SE, Howard J, Mohr D, Craig S. 2020. Self-disinfecting copper beds sustain terminal cleaning and disinfection effects throughout patient care. Appl Environ Microbiol 86:1–10; doi:10.1128/AEM.01886-19.

Schmidt MG, Attaway HH, Ms III, Bs SEF, Lisa L, Schmidt MG, et al. 2013. Copper Continuously Limits the Concentration of Bacteria Resident on Bed Rails within the Intensive Care Unit Steed PhD , Harold T . Michels PhD and Cassandra D . Salgado MD MS Source : Infection Control and Hospital Epidemiology , Vol . 34 , No . 5 , Spe. 34: 11–15.

Schmidt MG, Attaway HH, Sharpe PA, John J, Sepkowitz KA, Morgan A, et al. 2012. Sustained reduction of microbial burden on common hospital surfaces through introduction of copper. J Clin Microbiol 50:2217–2223; doi:10.1128/JCM.01032-12.

Schmidt MG, Fairey SE, Attaway HH. 2019. In situ evaluation of a persistent disinfectant provides continuous decontamination within the clinical environment. Am J Infect Control

47:732–734; doi:10.1016/j.ajic.2019.02.013.
Schmidt MG, Von Dessauer B, Benavente C, Benadof D, Cifuentes P, Elgueta A, et al. 2016. Copper surfaces are associated with significantly lower concentrations of bacteria on selected surfaces within a pediatric intensive care unit. Am J Infect Control 44:203–209; doi:10.1016/j.ajic.2015.09.008.
Sexton JD, Tanner BD, Maxwell SL, Gerba CP. 2011. Reduction in the microbial load on high-touch surfaces in hospital rooms by treatment with a portable saturated steam vapor disinfection system. Am J Infect Control 39:655–662; doi:10.1016/j.ajic.2010.11.009.
Shapey S, Machin K, Levi K, Boswell TC. 2008. Activity of a dry mist hydrogen peroxide system against environmental Clostridium difficile contamination in elderly care wards. J Hosp Infect 70:136–141; doi:10.1016/j.jhin.2008.06.008.
Shekhawat PS, Singh RN, Shekhawat R, Joshi KR. 1992. Fumigation of neonatal nursery: how effective in reducing the environmental pathogens? Indian Pediatr 29.
Shelly MJ, Scanlon TG, Ruddy R, Hannan MM, Murray JG. 2011. Meticillin-resistant Staphylococcus aureus (MRSA) environmental contamination in a radiology department. Clin Radiol 66:861–864; doi:10.1016/j.crad.2011.05.002.
Siani H, Wesgate R, Maillard JY. 2018. Impact of antimicrobial wipes compared with hypochlorite solution on environmental surface contamination in a health care setting: A double-crossover study. Am J Infect Control 46:1180–1187; doi:10.1016/j.ajic.2018.03.020.
Sifri CD, Burke GH, Enfield KB. 2016. Reduced health care-associated infections in an acute care community hospital using a combination of self-disinfecting copper-impregnated composite hard surfaces and linens. Am J Infect Control 44:1565–1571; doi:10.1016/j.ajic.2016.07.007.
Sigler V, Hensley S. 2013. Persistence of mixed staphylococci assemblages following disinfection of hospital room surfaces. J Hosp Infect 83:253–256; doi:10.1016/j.jhin.2012.12.009.
Simon Garcia MJ, Gonzalez Sanchez JA, Alcudia Perez F, Sanchez Sanchez C, Gomez Mayoral B, Merino Martinez MR. 2009. Evaluation of the effect of a cleaning disinfection intervention on the rate of multiresistant microorganism infections in the Intensive Care Unit. Enferm Intensiva 20: 27–34.
Singh H, Kumar R, Singh K, Attri J. 2017. Infection Control in Isolation Units/Hdus/Icus- a Comparative Study Using Three Different Disinfectants With Fogger for Environmental Decontamination. J Evol Med Dent Sci 6:3091–3096; doi:10.14260/jemds/2017/667.
Sjöberg M, Eriksson M, Andersson J, Norén T. 2014. Transmission of Clostridium difficile spores in isolation room environments and through hospital beds. Apmis 122:800–803; doi:10.1111/apm.12218.
Smith TL, Iwen PC, Olson SB, Rupp ME. 1998. Environmental contamination with vancomycin-resistant enterococci in an outpatient setting. Infect Control Hosp Epidemiol 19: 515–518.
Souli M, Antoniadou A, Katsarolis I, Mavrou I, Paramythiotou E, Papadomichelakis E, et al. 2017. Reduction of environmental contamination with multidrug-resistant bacteria by copper-alloy coating of surfaces in a highly endemic setting. Infect Control Hosp Epidemiol 38:765–771; doi:10.1017/ice.2017.52.
Stewart M, Bogusz A, Hunter J, Devanny I, Yip B, Reid D, et al. 2014. Evaluating Use of Neutral Electrolyzed Water for Cleaning Near-Patient Surfaces. Infect Control Hosp Epidemiol 35:1505–1510; doi:10.1086/678595.

Stibich M, Stachowiak J, Tanner B, Berkheiser M, Moore L, Raad I, et al. 2011. Evaluation of a Pulsed-Xenon Ultraviolet Room Disinfection Device for Impact on Hospital Operations and Microbial Reduction. Infect Control Hosp Epidemiol 32:286–288; doi:10.1086/658329.

Strassle P, Thom KA, Johnsonm JK, Leekha S, Lissauer M, Zhu J, et al. 2012. The effect of terminal cleaning on environmental contamination rates of multidrug-resistant Acinetobacter baumannii. Am J Infect Control 40:1005–1007; doi:10.1016/j.ajic.2012.05.027.

Strat E. 1971. Research on the effectiveness of disinfectants in the surfactant group o the degeneration of the hospital environment. Rev Med Chir Soc Med Nat Iasi 75: 957–966.

Styaningsih N, Suwundo A, Adi MS. 2019. Effectiveness of Disinfectant A and B on the Growth of Bacteria in the Area of Central Surgical Installation of Hospital X in Kudus City. Indian J Public Heal Res Dev 10: 795–804.

Sui YS, Wan GH, Chen YW, Ku HL, Li LP, Liu CH, et al. 2012. Effectiveness of bacterial disinfectants on surfaces of mechanical ventilator systems. Respir Care 57:250–256; doi:10.4187/respcare.01180.

Suzuki A, Namba Y, Matsuura M, Horisawa A. 1984. Bacterial contamination of floors and other surfaces in operating rooms: A five-year survey. J Hyg (Lond) 93:559–566; doi:10.1017/S002217240006513X.

Tamimi AH, Carlino S, Gerba CP. 2014. Long-term efficacy of a self-disinfecting coating in an intensive care unit. Am J Infect Control 42:1178–1181; doi:10.1016/j.ajic.2014.07.005.

Taneja N, Biswal M, Kumar A, Edwin A, Sunita T, Emmanuel R, et al. 2011. Hydrogen peroxide vapour for decontaminating air-conditioning ducts and rooms of an emergency complex in northern India: Time to move on. J Hosp Infect 78:200–203; doi:10.1016/j.jhin.2011.02.013.

Taylor L, Phillips P, Hastings R. 2009. Reduction of bacterial contamination in a healthcare environment by silver antimicrobial technology. J Infect Prev 10:6–12; doi:10.1177/1757177408099083.

Tekin A, Dal T, Selçuk CT, Deveci Ö, Tekin R, Mete M, et al. 2013. Orthophenylphenol in healthcare environments: A trial related to a new administration method and a review of the literature. Turkish J Med Sci 43:805–809; doi:10.3906/sag-1208-4.

Thom KA, Standiford HC, Johnson JK, Hanna N, Furuno JP. 2014. Effectiveness of an Antimicrobial Polymer to Decrease Contamination of Environmental Surfaces in the Clinical Setting. Infect Control Hosp Epidemiol 35:1060–1062; doi:10.1086/677159.

Turner AG, Hill C, Higgins MM, Craddock JG, Nc CH. 1974. Disinfection of Immersion Tanks (Hubbard) in a Hospital Burn Unit. Arch Env Heal 28: 101–105.

Vesley D, Klapes NA, Benzow K, Le CT. 1987. Microbiological evaluation of wet and dry floor sanitization systems in hospital patient rooms. Appl Environ Microbiol 53:1042–1045; doi:10.1128/aem.53.5.1042-1045.1987.

von Dessauer B, Navarrete MS, Benadof D, Benavente C, Schmidt MG. 2016. Potential effectiveness of copper surfaces in reducing health care–associated infection rates in a pediatric intensive and intermediate care unit: A nonrandomized controlled trial. Am J Infect Control 44:e133–e139; doi:10.1016/j.ajic.2016.03.053.

Wiemken TL, Curran DR, Kelley RR, Pacholski EB, Carrico RM, Peyrani P, et al. 2014. Evaluation of the effectiveness of improved hydrogen peroxide in the operating room. Am J Infect Control 42:1004–1005; doi:10.1016/j.ajic.2014.05.002.

Wilcox MH, Fawley WN, Wigglesworth N, Parnell P, Verity P, Freeman J. 2003. Comparison of

the effect of detergent versus hypochlorite cleaning on environmental contamination and incidence of Clostridium difficile infection. J Hosp Infect 54:109–114; doi:10.1016/S0195-6701(02)00400-0.

Youkee D, Brown CS, Lilburn P, Shetty N, Brooks T, Simpson A, et al. 2015. Assessment of Environmental Contamination and Environmental Decontamination Practices within an Ebola Holding Unit, Freetown, Sierra Leone. PLoS One 10:1–11; doi:10.1371/journal.pone.0145167.

Yuen JWM, Chung TWK, Loke AY. 2015. Methicillin-Resistant Staphylococcus aureus (MRSA) contamination in bedside surfaces of a hospital ward and the potential effectiveness of enhanced disinfection with an antimicrobial polymer surfactant. Int J Environ Res Public Health 12:3026–3041; doi:10.3390/ijerph120303026.

Yui S, Ali S, Muzslay M, Jeanes A, Wilson APR. 2017. Identification of Clostridium difficile Reservoirs in the Patient Environment and Efficacy of Aerial Hydrogen Peroxide Decontamination. Infect Control Hosp Epidemiol 38:1487–1492; doi:10.1017/ice.2017.227.

Zhang A, Nerandzic MM, Kundrapu S, Donskey CJ. 2013. Does Organic Material on Hospital Surfaces Reduce the Effectiveness of Hypochlorite and UV Radiation for Disinfection of Clostridium difficile ? . Infect Control Hosp Epidemiol 34:1106–1108; doi:10.1086/673148.

Zubair M, Imtiaz S, Zafar A, Javed H, Atif M, Abosalif KOAA, et al. 2018. Role of hospital surfaces in transmission of infectious diseases. Pakistan J Med Heal Sci 12: 857–859.
